# Employing an innovative underwater camera to improve electronic monitoring in the commercial Gulf of Mexico reef fish fishery

**DOI:** 10.1371/journal.pone.0298588

**Published:** 2024-03-08

**Authors:** Carole Neidig, Max Lee, Genevieve Patrick, Ryan Schloesser

**Affiliations:** 1 Center for Fisheries Electronic Monitoring, Mote Marine Laboratory, Sarasota, Florida, United States of America; 2 Fisheries Ecology & Enhancement, Mote Marine Laboratory, Sarasota, Florida, United States of America; Instituto Portugues do Mar e da Atmosfera, PORTUGAL

## Abstract

Vessel electronic monitoring (EM) systems used in fisheries around the world apply a variety of cameras to record catch as it is brought on deck and during fish processing activities. In EM work conducted by the Center for Fisheries Electronic Monitoring at Mote (CFEMM) in the Gulf of Mexico commercial reef fish fishery, there was a need to improve upon current technologies to enhance camera views for accurate species identification of large sharks, particularly those that were released while underwater at the vessel side or underneath the hull. This paper describes how this problem was addressed with the development of the first known EM system integrated underwater camera (UCAM) with a specialized vessel-specific deployment device on a bottom longline reef fish vessel. Data are presented based on blind video reviews from CFEMM trained reviewers of the resulting UCAM video footage compared with video from only the overhead EM cameras from 68 gear retrievals collected from eight fishing trips. Results revealed that the UCAM was a successful tool for capturing clear underwater video footage of released large (>2m) sharks to enable reviewers to improve individual species identification, determination, and fate by 34.4%. This was particularly important for obtaining data on incidental catches of large protected shark species. It also provided clear underwater imagery of the presence of potential predators such as marine mammals close to the vessel, more specifically bottlenose dolphin (*Tursiops truncatus*) during gear retrieval, which often damaged or removed catch. This information is intended to assist researchers in need of gathering critical data on bycatch in close proximity to a vessel in which conventional overhead EM cameras are limited.

## Introduction

Electronic monitoring (EM) system technology continues to evolve and gain momentum as an onboard fisheries dependent sampling tool for obtaining permanent video documentation of a vessel’s fishing efforts and catch as costs decrease [1,2]. The application of EM video technology has been used in a variety of fisheries around the world and in several U.S. regions [1–3] and has demonstrated that it can increase monitoring coverage and improve data robustness and compliance [3,4]. In many cases, EM is well suited for managers who require accurate estimates of target species catch, and incidental interactions with threatened species [1], particularly in fisheries challenged with low observer coverage [1,4]. A typical EM system allows for multiple areas of a vessel to be monitored simultaneously and near-continuously [3,5] through the use of activity sensors, a global positioning system (GPS), computer hardware, and overhead cameras [3,6]. Depending on the vessel application and camera configuration, conventional EM cameras are used to document catch as it is brought onboard, during processing, and when fish are discarded [2]. Detailed data on discards, especially in the case of incidental catches of regulated and or sensitive species are often rare and can be easily missed if reliant on a sampling program with less than full coverage [1,7]. The use of EM can enhance data collection in these circumstances by recording information about gear, targeted catch, incidental catch, and discards, along with catch handling, processing and /or fishing effort (location and time fished) to produce reliable and verifiable information for fisheries managers consideration in stock assessments [1,3,8] for improving sustainable fishing practices [3,7].

The Center for Fisheries Electronic Monitoring at Mote (CFEMM) has spearheaded EM pilot studies in the Gulf of Mexico commercial reef fishery since 2016 to primarily provide bycatch and discard data, as observer coverage in this fishery is limited. During this time, 22 volunteer bottom longline and vertical line vessels have applied overhead cameras to record fishing activities. In CFEMM studies, Saltwater Inc. (Anchorage, AK) EM systems, including sensors, GPS, non-proprietary software, and roof or aluminum boom mounted cameras were used for monitoring catch, bycatch, and most discarded fish activities. Though similar to observer experiences, the reviewers were limited in their ability to accurately identify and quantify incidental catches of large sharks when they were quickly discarded by vessel crew cutting the leader line when the hooked shark was underwater. These occurrences led CFEMM researchers to investigate solutions that could overcome this problem by improving upon the current EM technology limitations to provide better coverage, including the testing of underwater cameras and development of compatible camera deployment devices. Key criteria for an underwater camera was that it could be fully integrated with the Saltwater Inc. EM system processor and software, could be easily deployed, unobtrusive to the captain and crew’s fishing activities, durable in the marine environment, and provide clear and steady imagery under various sea conditions to improve the video reviewers ability to accurately identify shark species that were released underwater.

Underwater cameras of different types and levels of technological advancements have been used with increasing frequency to further research in long-term monitoring of the environment [9], *in situ* behavior observations of deep-water species [10], abundance surveys, including sharks and rays [11], fish habitat monitoring [12], and in fish farming net cage applications [13]. Valuable information has also been obtained using underwater cameras in studies related to commercial fishing gear, including fish and crab behavior with trawls and seines [14–17], and efficiency of catch with longlines [18]. One study [19] involving pelagic longline vessels describes a similar problem as observed in our work on bottom longline vessels, with not being able to confirm the identification of large sharks released while underwater. Though the commercial vessels fishing in the eastern Mediterranean basin did not have EM video systems; a submersible GoPro was used to document and confirm what had been suspected by the fishermen, that the incidental longline catches were of a scarce species of shark outside of its previously reported range, but whose presence was not previously confirmed due to limited observer coverage or photographic evidence [19]. The study presented in this paper describes the design, application, and test results of a novel approach for providing near-vessel views and permanent photographic documentation aligned with corresponding trip data of large sharks not hauled above the water surface. This was accomplished by using an underwater video camera and a specialized deployment device integrated with a standard EM system on a commercial bottom longline fishing vessel to provide views in which shark species could be identified and correlated with their location specific catch data.

## Methods

### Fishing vessel, effort, and area

**Fig 1:**
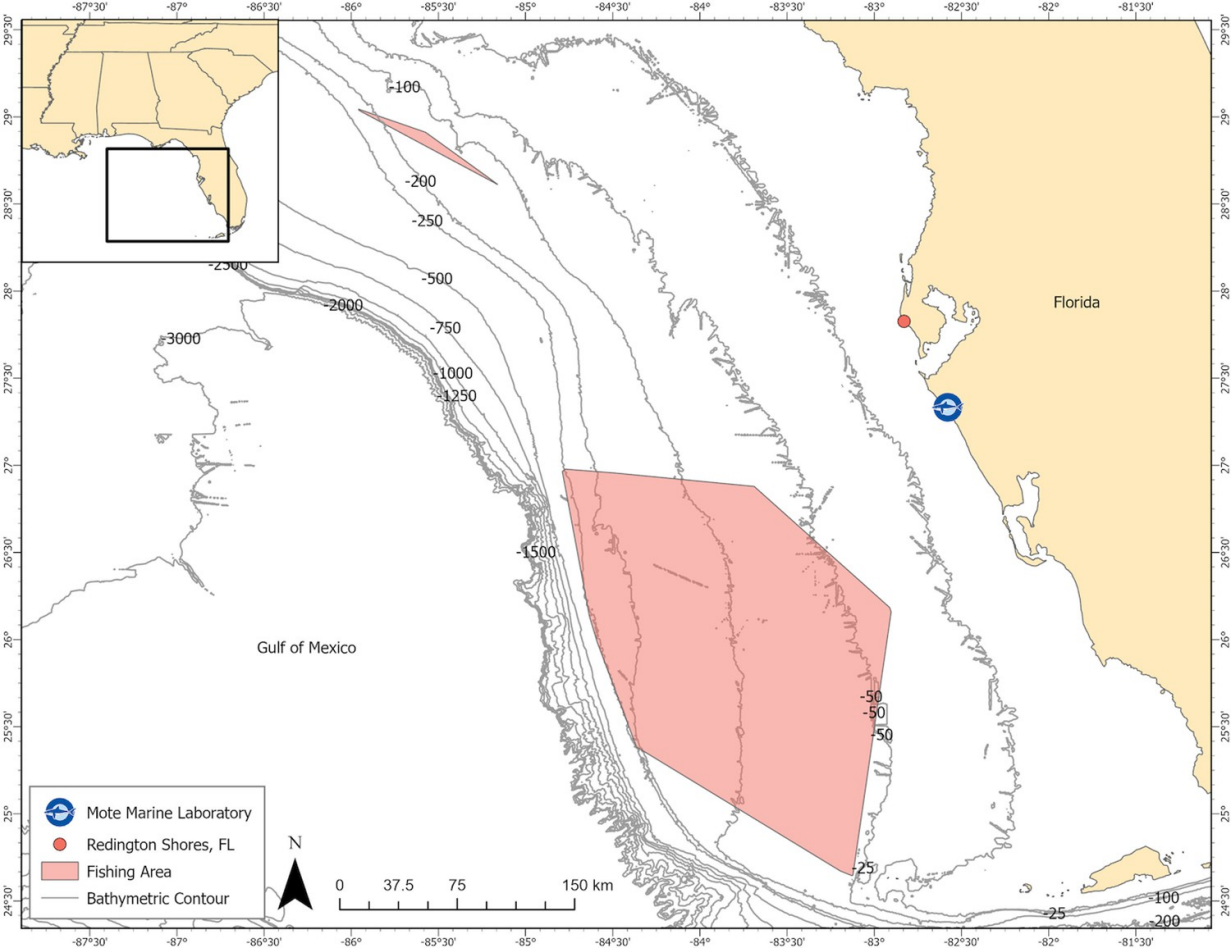
Areas fished in the eastern Gulf of Mexico as indicated by two polygons, during testing of the underwater camera device from February 2020 through November 2021. The orange circle indicates the vessel’s point of departure from Redington Shores, FL and the blue logo represents the location of the Center for Fisheries Electronic Monitoring at Mote Marine Laboratory, Sarasota, FL.

The participating bottom longline vessel was the F/V Amy Lynn, measuring 14.75 m in length, 4.8 m beam, and 2.7 m draft, located on the west coast of Florida in Redington Shores. The captain / owner fished with a crew of 2–3 on trips lasting up to 14 days. Fishing gear included a single braided stainless steel mainline with a deployment range of approximately 8,000–10,900 m (8–10.9 km) set with 13/0 circle hooks attached to 1.82 m monofilament leaders of ~ 113.3 kg (300/lb) test. Based on NOAA Gulf of Mexico federal regulations, up to 750 circle hooks were deployed at one time. Gear was typically set three times per day and soaked up to 6.3 hrs from the beginning of the set to the end of the haul. A total of eight trips were made for this evaluation with the fishing effort occurring in the eastern Gulf of Mexico on the West Florida Shelf, in approximate depths from 38 m to 286 m (Fig 1). The participating vessel did not require a federal permit for this work as the study was conducted during their normal fishing activities and did not involve the utilization of specialized fishing gear or the retention of specific fish species.

### Electronic monitoring equipment and software

The vessel wheel house was equipped with a Saltwater Inc. (Anchorage, AK) Electronic Monitoring Unit (EMU) consisting of a Lanner-LVC-5770 model processor (Lanner Electronics, Taiwan) affixed to a specialized metal mount with four rubber cushioned expansion bolts to reduce vibration, which on this vessel was fastened to the cabin ceiling. Two processor slots each held a one 1 terabyte hard drive in sliding metal caddies, secured in place with thumb screws. In addition, the EMU included a 25.4 cm liquid crystal display monitor and a waterproof keyboard with touchpad. A multi-port Power Over Ethernet (POE) switch connected marine grade Category 5 ethernet cable (CAT-5e) cables from the control processor center to each of four overhead Geovision-GV-EVD3100 (Geovision Inc., Taipei) model closed circuit fixed focal length digital Internet Protocol (IP) cameras with light emitting diodes (LEDs). The cameras were mounted for optimum coverage, including two underneath the deck roof to view the processing table and the bow work area, one on a roofline for viewing discards thrown overboard, and one on a roof mounted aluminum boom to provide coverage of the hauling area. The hauling camera provided wide viewing of the vessel’s starboard deck, gunwale, and a large area of the water’s surface proximate to the vessel.

The cameras were triggered to record video (no sound was recorded) based on software settings associated with a magnetic reverse polarity rotation sensor installed on the mainline drum, and an inline hydraulic sensor, which also logged data when gear was in use, indicating fishing activity. A GPS receiver mounted on the cabin roof recorded vessel position, speed, and heading. A Saltwater Inc. non-proprietary Linux software program encrypted all recorded data and imagery on the retrievable hard drives.

### Underwater camera and deployment device development

Early efforts to integrate an underwater camera with an EM system were restricted by Saltwater Inc.’s EMU “01” version processor software that was designed to link with only Geovision camera software. An underwater camera option with the specifications required to operate with this version of software was not available at the time, which led to several investigative trials. One included the use of a typical EM system Geovision camera encased in a waterproof enclosure attached to the end of a polyvinyl chloride (PVC) cylinder with fabricated aluminum stabilizing fins, that was bulky and could not be easily controlled, though it recorded to an EM system. In addition, a GoPro type HD 1080P (ODRVM, Taiwan) camera was tested, but it had limited battery life and the resulting video recordings could not be integrated with the EM Review software. A Saltwater Inc. upgraded “02” version software was developed and allowed for the use of any CAT-5e compatible camera with their EM system, and thus opened the door for exploring submersible cameras that could potentially provide optimum near vessel underwater coverage. The chosen option for testing was an underwater camera provided by SubAqua Imaging Systems (San Diego, CA).

SubAqua Imaging Systems’ Vivotek (VIVOTEK Inc., Taiwan) SAIS IP HD CAM advanced integrated security IP high definition underwater camera (Bullet Cam) was selected for this study based on feedback provided by researchers who had applied it in a variety of marine and freshwater monitoring situations. It was compact, measuring 60 mm L x 64 mm D, weighed 0.74 kg, had a rugged plastic exterior with no exposed metal parts, and could be reliably submersed for years at a time at depths up to 60 m. The camera had a horizontal viewing angle of 88 degrees, a resolution of 1920 x 1080 dpi, and a waterproof marine grade POE connection for attaching a single CAT-5e cable from the EM processor. A 6.1 m length of marine grade submersible, heavy-duty CAT-5e cable with a wet-pluggable break-away connector mated with the camera connector. The break-away connection was intended to prevent the camera from being damaged, but still secure and retrievable in the event that the vessel’s fishing gear became entangled with the deployment device.

**Fig 2:**
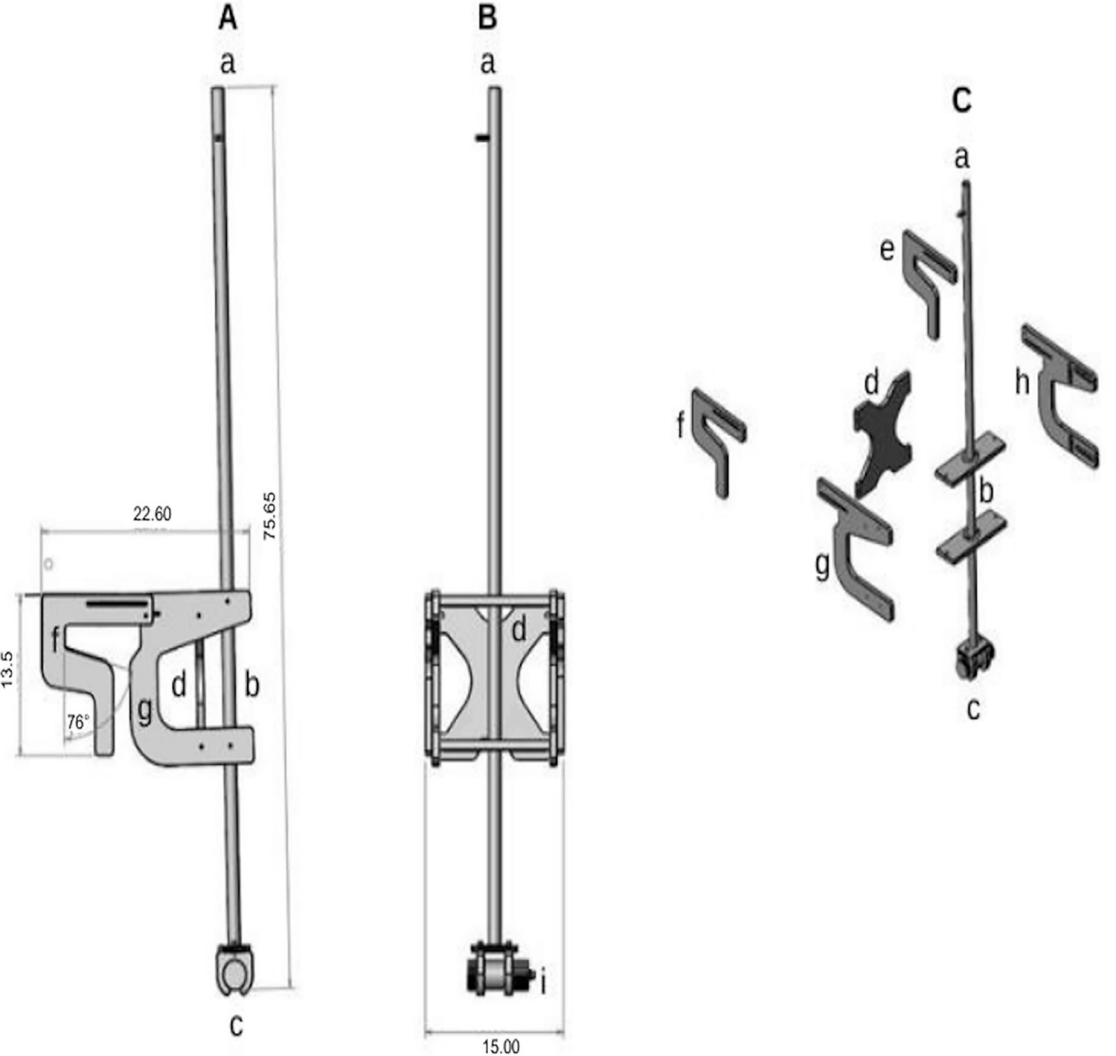
Computer aided designed (CAD) software schematic of the underwater camera deployment device and its components (measurements are in centimeters [cm]). Reprinted with permission from SeaSucker LLC (Bradenton, FL). (A) Left side view (a) aluminum pole, (b) pole mount location, (c) camera harness, (d) rear mount support, (f) one of two adjustable hinged arms, (g) mount side support. (B) Front view (a) pole, (d) rear mount support, (i) camera in harness showing female Cat5e connector. (C) Deployment device individual components (a) pole, (b) height adjustment tabs, (c) camera harness, (d) rear mount support, (e and f) right and left adjustment arms, (g and h) left and right supports.

The underwater camera (hereafter, known as the UCAM) was deployed using a detachable pole deployment device (Fig 2) mounted to the vessel’s starboard gunwale. The deployment device and camera cable specifications were designed through onsite vessel visits by a CFEMM volunteer citizen scientist and a SeaSucker LLC (Bradenton, FL) engineer to obtain measurements and gain captain and crew feedback for consideration in the design. The deployment device vessel mount (57.4 cm x 38.1 cm x 34.3 cm) (Fig 2A–2C) was then engineered and stamped through the use of computer-aided design (CAD) and drafting tools, and was fabricated out of 12.7 mm marine grade King Plastics Corporation StarBoard (Medford, OR) by SeaSucker LLC. An aluminum pole (1.92 m L x 2.5 cm D) extended through the middle of the vessel mount (Fig 2A and 2B). At the bottom end of the pole, a durable plastic composite camera harness from SubAqua Imaging System was secured with stainless steel bolts (Fig 2A).

A CAT-5e marine grade power cable connected to the EMU processor was secured along the cabin ceiling to the deck area underneath the outside deck roofline, where it was positioned to extend down the center of the UCAM deployment pole, and exited through a slot just above the bottom of the pole to connect to the camera. At the camera connection point, a waterproof MacArtney Inc. (Latin America) SubConn Micro Circular female connector was used. SubAqua Inc. applied a vacuum and baking process, using the same polyurethane material as the cable jacket, to chemically cross-link the two together for a strong bond.

The deployment pole was positioned to the outer side of two Starboard material adjustable hinged arms. These arms were loosened to fit over the gunwale and adjusted forward to the marked optimum location, where two hard plastic star knobs with stainless steel screws were hand-tightened to securely lock the device in position. Closed cell rubber pads (50.8 mm) were affixed on the inside at the end of each adjustable arm to prevent sliding on the smooth fiberglass gunwale and minimize camera vibration when the vessel traveled at 1.9 to 3.0 knots during gear retrieval.

**Fig 3:**
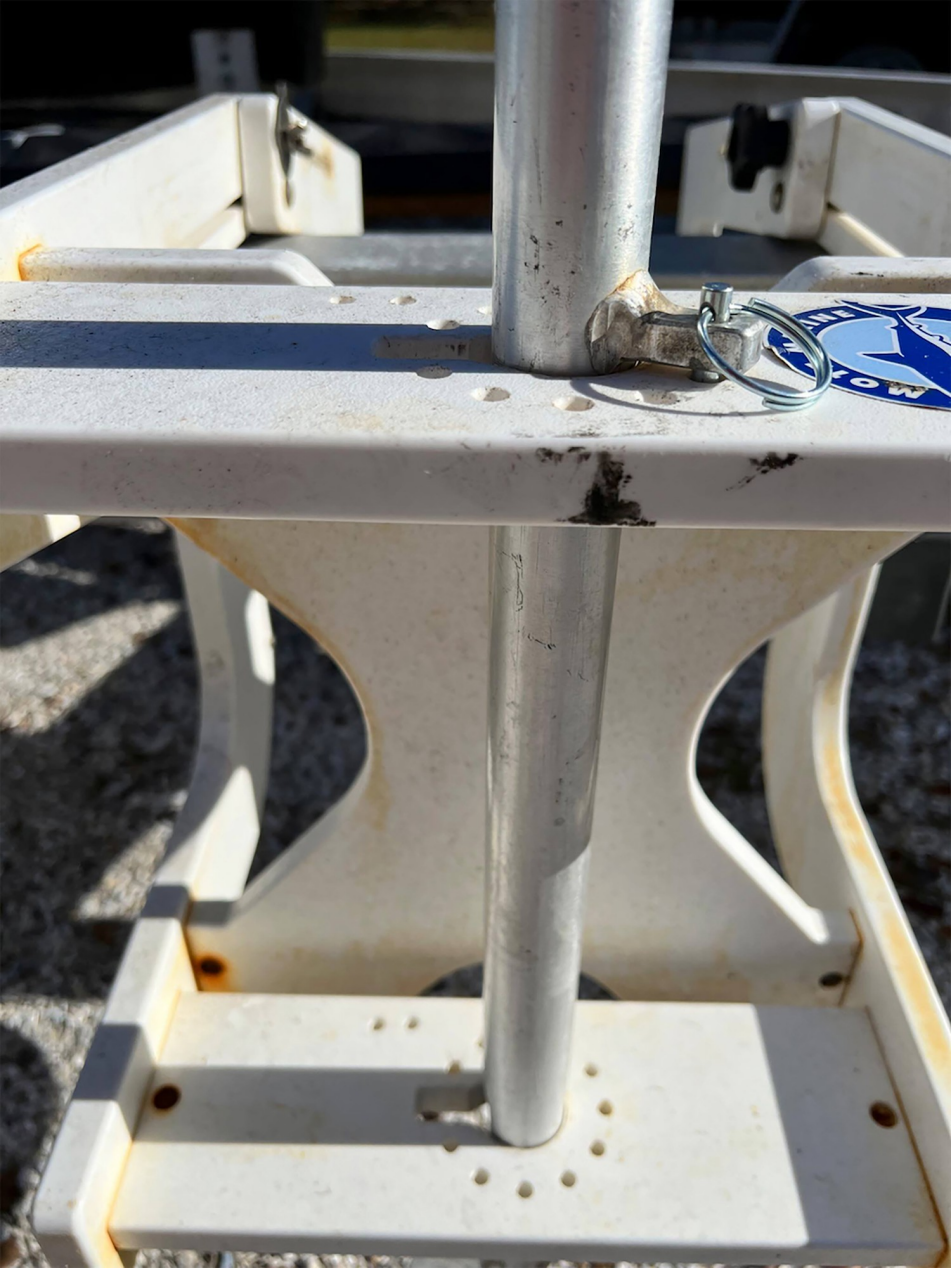
Underwater camera deployment pole positioned through the mount device and secured at a selected depth with a looped stainless steel cotter pin.

During long vessel travel periods to and from fishing areas the crew detached and stored the underwater camera in a protective waterproof Pelican Products Inc. (US/Global) case in the cabin and the deployment device with the power cable was strapped to the cabin roof. When the vessel reached the fishing grounds the crew secured the camera to the pole harness and positioned the deployment device as described on the gunwale, aft of the starboard hauling station. After the crew completed deployment of the vessels’ mainline gear, the camera pole was lowered underwater and rotated to the desired camera angle, where it was fastened using a stainless steel cotter pin through one of the two pole rectangular tabs and through a hole on the top of the device deployment frame (Fig 3). The depth of the camera was adjusted by a series of aluminum pins extending 25.4 mm from the pole in a vertical line 58.42 cm apart. The pins allowed the crew to quickly raise or lower the pole and lock it into position without changing the camera angle, so that the camera remained below the water surface, based on sea conditions. From initial vessel trials, the optimum camera position for documenting a hauling event was to face the center of the camera lens outwards from the hull at a right angle of 18–33 degrees, and at a depth of 1.83 to 2.13 m below the surface.

### Image review and annotation

At CFEMM, the resulting video and sensor data stored on the EMU hard drives were downloaded and preprocessed for review. Using Saltwater Inc. non-proprietary version “02” review software the data from each trip was synced along a timeline, which staff prepared for review by marking each complete event, including the set, soak and haul as indicated by vessel sensor data. The footage that was recorded during the hauls from the four overhead and the one underwater camera were reviewed and annotated for catch and discards, during which a drop down list of species and species groups was available for reviewers to select, as well as menu choices for selecting observed handling, condition on arrival, fate, and specifically for sharks their biological characteristics, including size estimate ([<1m], [1-2m], [>2m]), maturity (juvenile or adult), and sex (presence or absence of claspers). Video imagery could be viewed a single frame at a time, zoomed in or out, reversed, or the speed incrementally increased or slowed. To evaluate if the underwater camera successfully contributed and benefited the EM review process, a blind review of the video was conducted, where footage including the underwater camera was reviewed separately from the same haul footage from only the overhead cameras.

Annotated data were aggregated using R statistical software [20] and contingency tables developed to show differences among reviews with and without the underwater camera (hereafter referred to as UCAM and NOCAM, respectively) regarding the number of individuals classified by species, handling, condition on arrival, fate, size, and sex. Differences were examined for significance with Pearson’s Chi-squared test using the chisq.test function in R, with the exception of differences in species counts for fish and sharks for which Fisher’s exact test was examined using fisher.test in R due to many counts being < 5. P-values were computed by Monte Carlo simulation of 2000 replicates.

## Results and discussion

From February 2020 through November 2021 the UCAM was deployed during 68 hauls over eight trips. Based on the video review of the data collected from the UCAM a total of 243 sharks were caught, which represented 10.27% of the total catch. Sharks <1m in length represented 56.4% of those caught, with 25.9% in the 1m - 2m range, and 17.7% in the >2m range. Although more sharks were identified when reviewing the UCAM footage, the overall distribution of shark species did not differ compared with the NOCAM (p-value = 0.99), due in part to the large number of small bodied sharks, such as Atlantic sharpnose, *Rhizoprionodon terraenovae* and smooth dogfish, *Mustelus canis*, that were readily identified when they were brought on board (Table 1) for the hook to be removed. Review of the NOCAM versus UCAM footage revealed that the onboard cameras (NOCAM) provided better views for documenting and identifying small (>1m) sharks. In contrast, the UCAM only made a small difference in species identification of medium (1m - 2m) sized sharks compared to those identified by review of the overhead camera views (Table 2). This resulted in no significant differences in the distribution of observed shark size classes (X-squared = 1.4845, p-value = 0.47).

**Table 1:** Shark species caught and the number that were documented by reviewers based on video recorded from electronic monitoring overhead cameras (NOCAM) as compared to an underwater camera (UCAM).

Common Name	NOCAM	UCAM
Dogfish, Smooth (Florida)	63	64
Atlantic Sharpnose Shark	56	55
Sandbar Shark	24	36
Blacknose Shark	25	24
Dogfish, Spiny (Cuban)	22	20
Night Shark	10	10
Tiger Shark	8	10
Scalloped Hammerhead	7	7
Nurse Shark	5	5
Silky Shark	4	4
Shark, Unidentified	6	3
Blacktip Shark	1	2
Bull Shark	0	1
Great Hammerhead	1	1
Sixgill Shark (all)	1	1
Carcharhinid, Unidentified	1	0

**Table 2:** Estimated shark lengths in meters based on review of video recorded by overhead cameras (NOCAM) versus an underwater camera (UCAM).

Length of Shark	NOCAM	UCAM
Small (Less than 1m)	140	137
Medium (Between 1m and 2m)	62	63
Large (Greater than 2m)	32	43

None of the sharks >2 m in length were brought onboard the vessel. Of these large sharks almost all were released, with only one that dropped off the hook while at the side of the vessel. Identification of sharks >2m increased by 34.4% compared to using only overhead cameras (Table 2). The primary shark species exceeding >2 m in length in these incidental catches were sandbar sharks, *Carcharhinus plumbeus*, which are prohibited from commercial harvest. There was a notable 50% increase in the number observed, facilitated by the identification capabilities of the UCAM.

**Fig 4:**
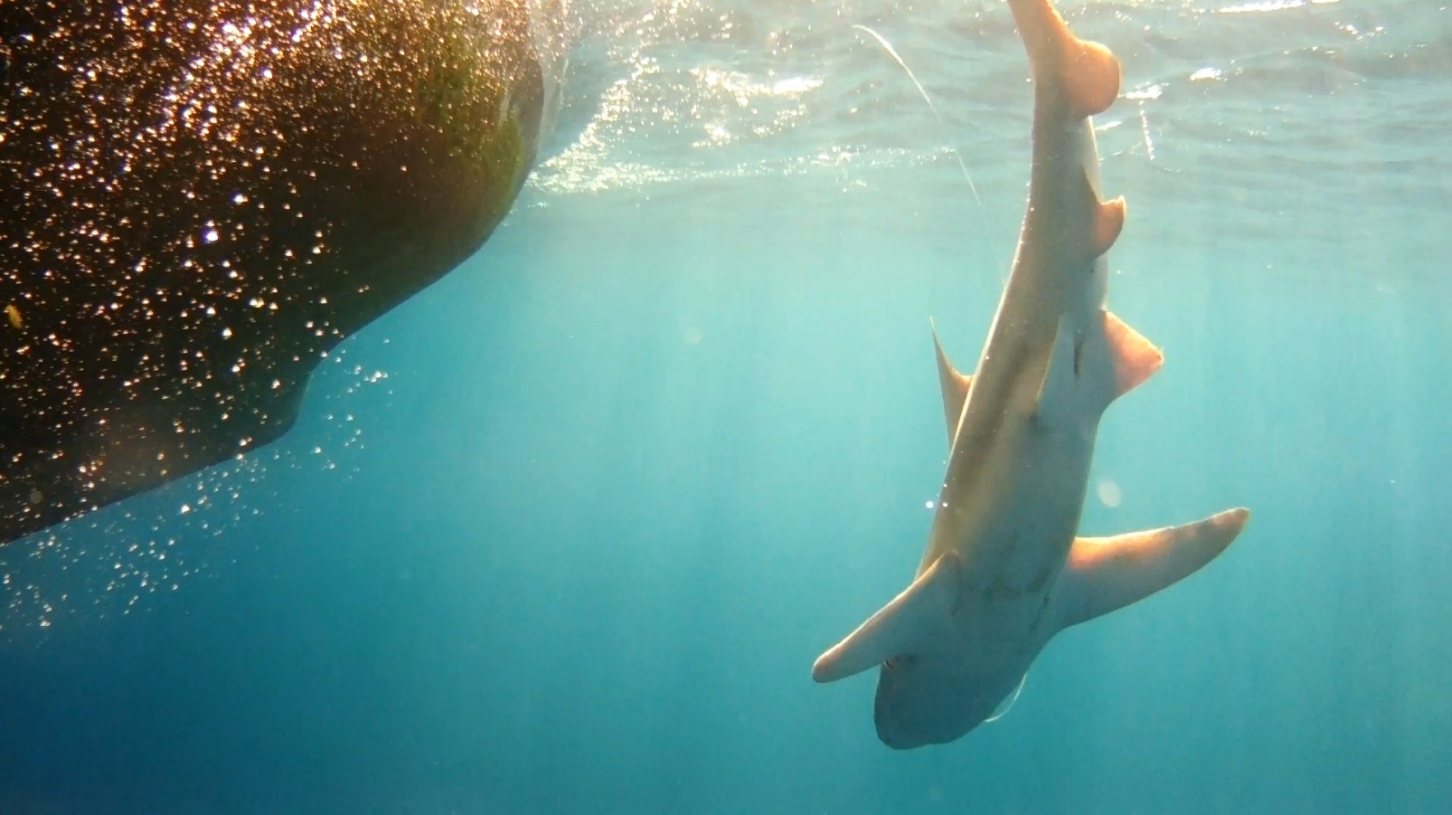
Recorded view of an adult (>2m) female sandbar shark, *C*. *plumbeus* dorsal side up immediately post release from the leader as shown in UCAM footage.

**Fig 5:**
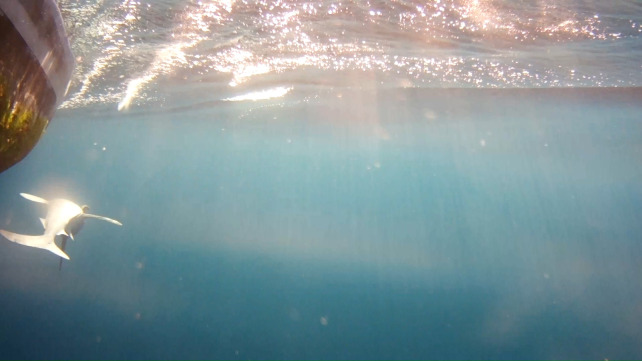
Recorded view of an adult (>2m) female sandbar shark, *C*. *plumbeus* positioned under the vessel hull as viewed from the overhead boom mounted camera.

**Fig 6:**
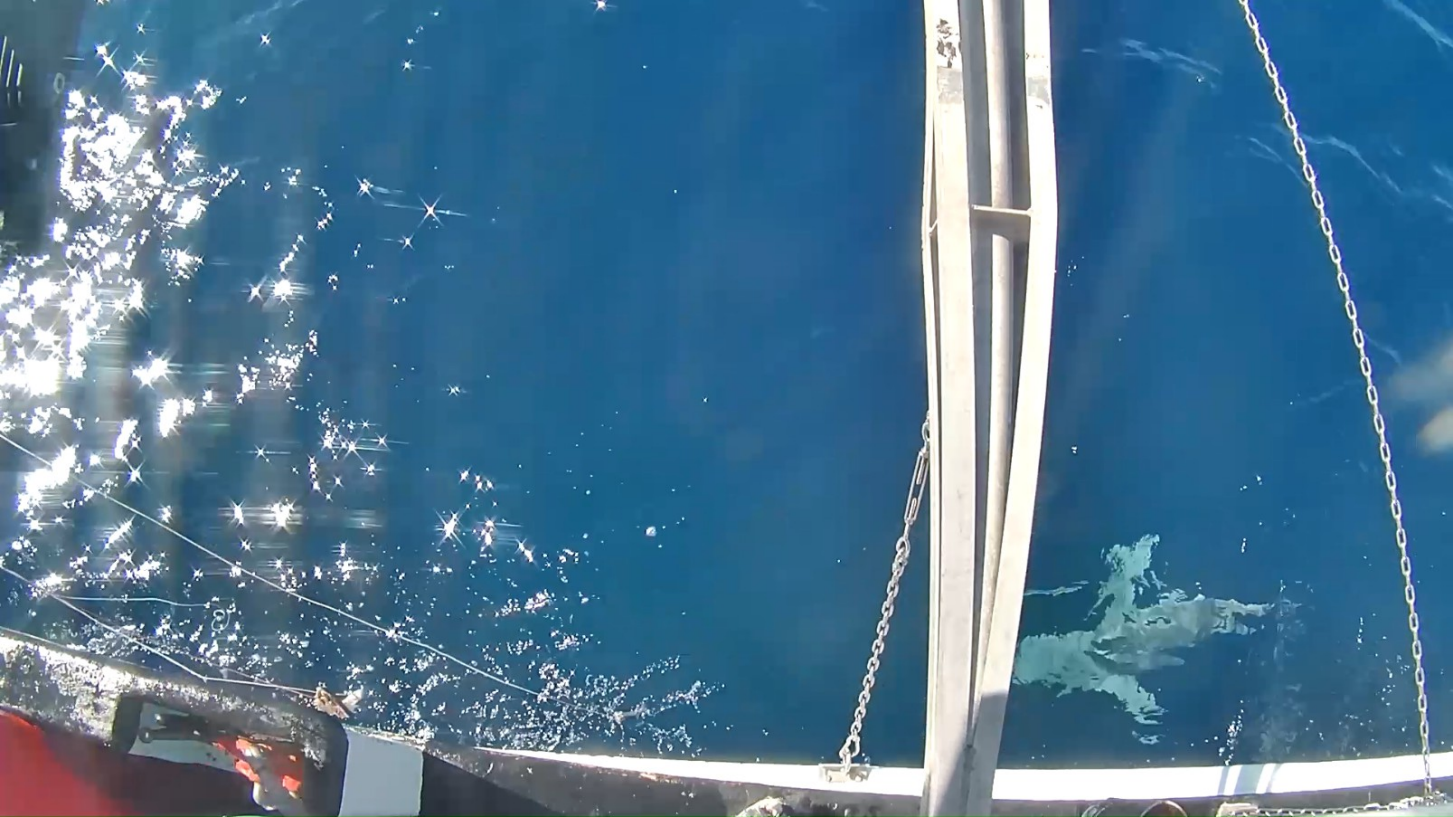
View of an adult (>2m) female sandbar shark, *C*. *plumbeus* (same as the shark seen in Fig 4) as viewed from the overhead boom mounted camera.

**Fig 7:**
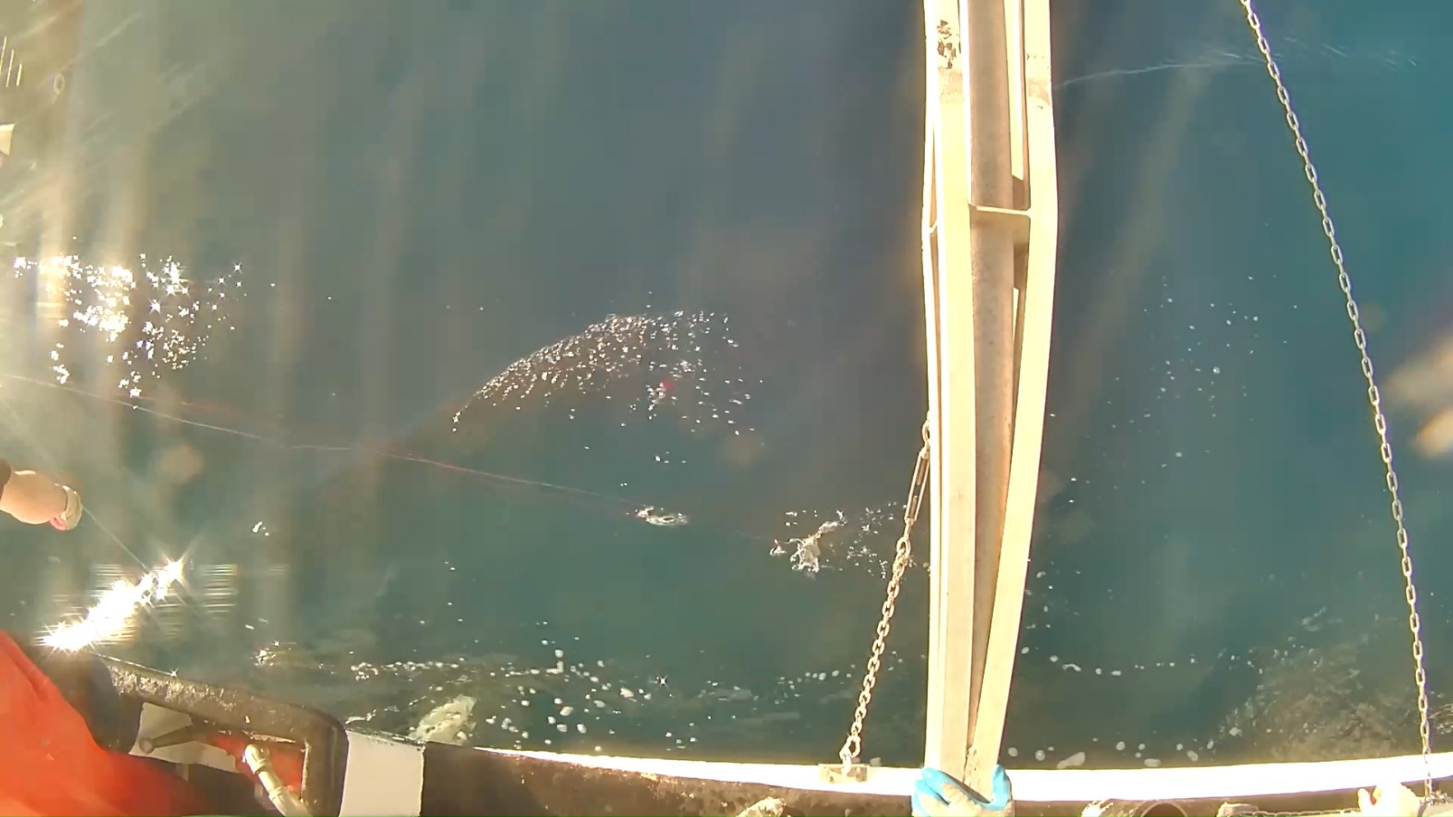
View of an adult (>2m) female sandbar shark, *C*. *plumbeus* (same as the shark documented in Fig 5) as viewed from the overhead boom mounted camera as it moved from under the vessel hull.

The UCAM video provided a clear view for reviewers to discern if a large shark was alive when it was hauled close to the vessel and for determination of the sharks sex and fate at point of release, as presented in Figs 4 and 5 with large (>2 m) sandbar sharks (*C*. *plumbeus*), identified as females, that both swam away. Comparatively, review of respective boom mounted camera footage showed a partial unclear view of potentially large sharks that could not be accurately identified to species, as seen in Figs 6 and 7. Overall, maturity and or sex status was not able to be assessed as frequently with the boom mounted overhead cameras as they were with the UCAM, resulting in significantly different proportions of shark sex being identified (X-squared = 23.17, p-value < 0.01) Table 3.

**Table 3:** Sex of sharks as determined by reviewers based on recorded video views from overhead cameras compared to an underwater camera (UCAM).

Sex of Shark	NOCAM	UCAM
Female	86	116
Male (Claspers)	59	77
Unknown Maturity and/or Sex	62	38
Known Juvenile	7	8
Known Adult—Undetermined Sex	20	4

**Fig 8:**
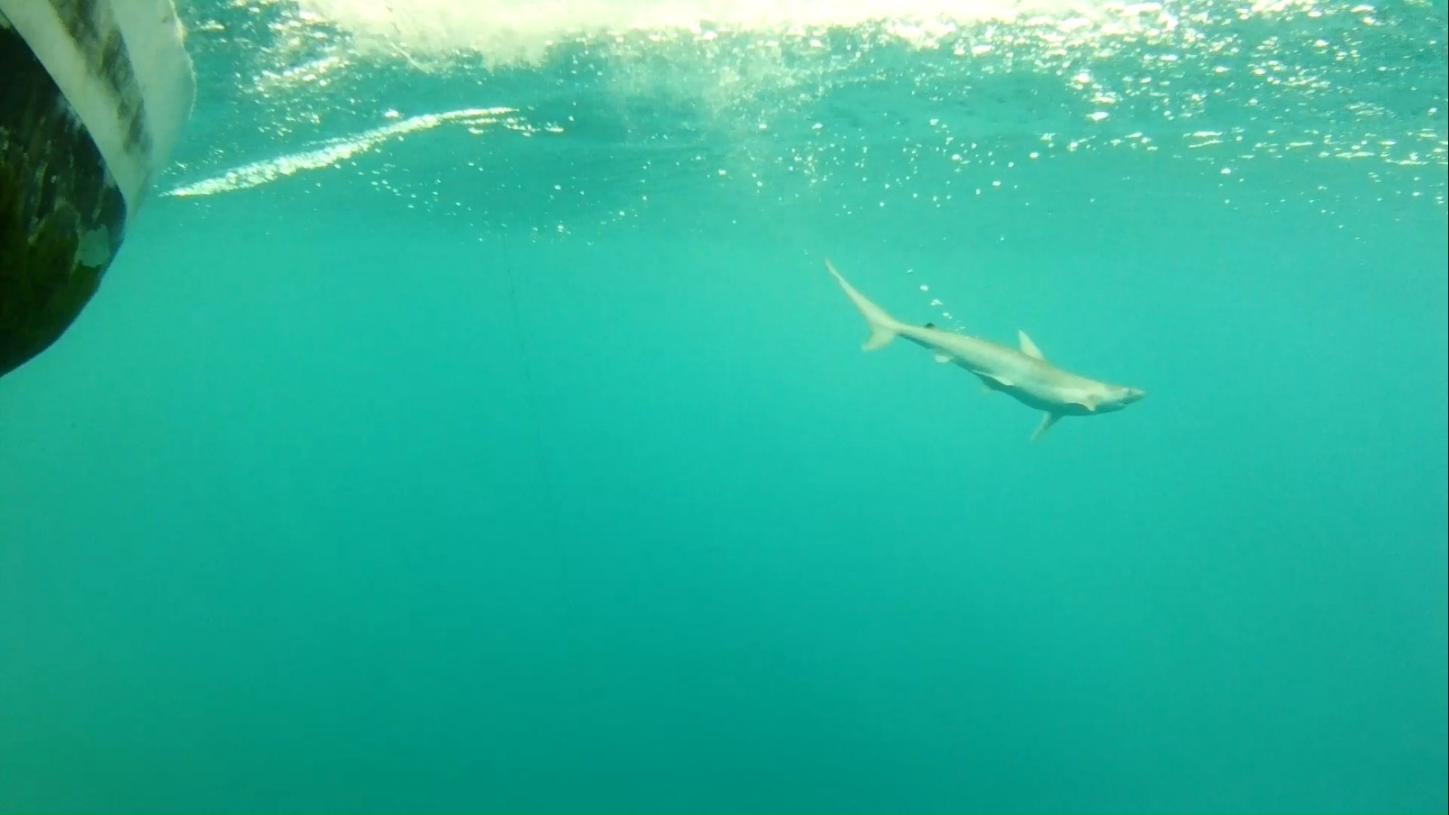
Underwater camera view of a live discarded Atlantic sharpnose, *R*. *terraenovae* shark.

**Fig 9:**
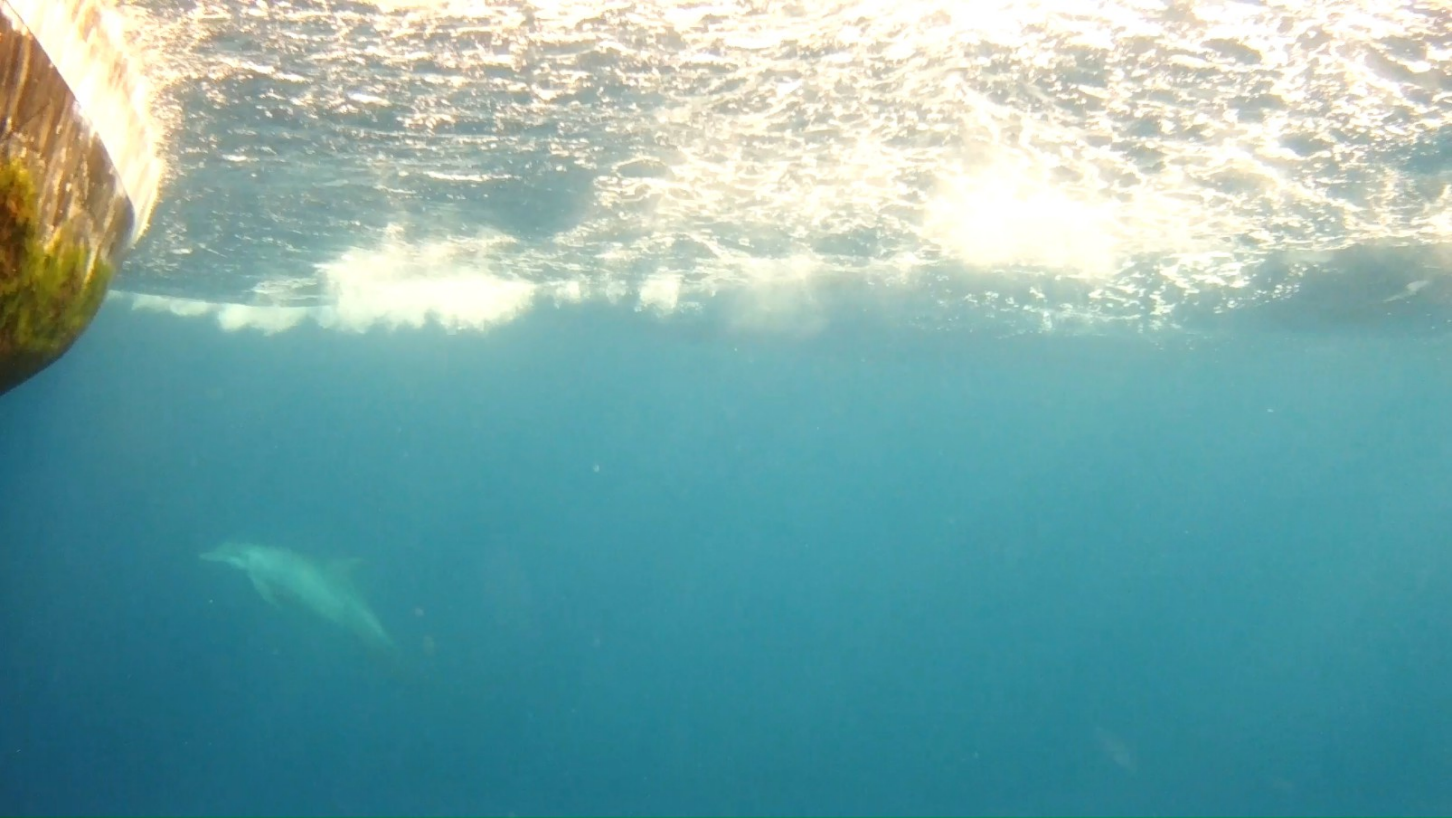
Underwater camera view of a common bottlenose dolphin, *T*. *truncatus* starboard of the vessel bow during gear retrieval.

For smaller <2m sharks, the UCAM provided an additional view to use with the overhead boom camera for accurately documenting fate at discard. This can be seen with an Atlantic sharpnose shark, *R*. *terraenovae* measuring <1m, as depicted in Fig 8, that was observed swimming away after being handled onboard and discarded. In addition, these views were beneficial for documenting the presence of potential predators such as bottlenose dolphins, *Tursiops truncatus* (Fig 9), when they were in close proximity to the vessel hauling area during gear retrieval. Though damaged catch was documented during these occurrences, no predation incidents were observed.

While an underwater camera may not provide benefits in some EM applications, these trials demonstrated the value of integrating an underwater camera with standard EM systems overhead cameras. This integration proved valuable for recording clear underwater video footage of discarded large sharks that were released or broke off in close proximity to the longline vessel. Other studies have successfully used underwater cameras to document fish interactions with commercial gear [15,17,18,21]. However, these studies involved self-contained cameras that were not linked with an EM system. Similarly, a study conducted by Kleitou [19] underscores the value of underwater video evidence of incidental captures of a rare large shark species and their condition in a commercial fishery where large sharks were released and limited observer coverage contributed to an underestimation of the species range. Therefore, the integrated underwater camera with an EM vessel system approach presented in this paper is particularly important for fisheries with limited observer coverage and where review of only overhead EM camera footage could lead to reviewers misidentifications or not seeing the released incidental bycatch at all. This is especially significant in situations where rare or protected species may be encountered.

Implementing a UCAM as described here, or similar underwater camera technology integrated with an EM system during all hauls enhances research capabilities of partnerships with the commercial fishing industry. Enhancements in bycatch data, especially in documenting large shark species identity, biological characteristics, and immediate fate, can reveal sex-specific habitat use patterns and impacts of fishery interactions. This is particularly valuable as the data is collected along a timeline with specific GPS-identified capture locations, allowing for spatiotemporal correlations. To extend this work within and across commercial fisheries, particularly longline fisheries, further research should focus on developing modular or adaptable deployment systems that can be easily customized for different vessel types and sizes, which would enhance the scalability and applicability of the technology. In addition, identifying underwater cameras with advancements of wireless or Bluetooth connectivity to synchronize with EM systems with these technologies, would enhance their usability and allow for the elimination of physical connection cables, making deployment and retrieval more efficient. Advances in these areas can improve integration of this technology for not only recording near vessel releases and short-term post-release survival, but also identification of specific predator species responsible for near surface depredation of targeted catch, discards, and deployed gear.

Similar to the findings presented in this study, it is expected that the incorporation of underwater cameras with EM systems also presents a promising opportunity to augment coverage of captures in pelagic longline fisheries. This enhancement could be particularly valuable for improving the accuracy of identification and accounting for threatened or endangered species and for observing their condition during a break-off near a vessel or other discard event. The underwater camera view can help to document their movement as they swim away, sink, or become preyed upon. This technology opens up new possibilities for researchers to gain more in-depth observations of the dynamics surrounding discard events.

Furthermore, the integration of underwater cameras with EM systems holds the potential to enhance data collection in studies evaluating the efficiency of gear modifications, allowing for a more comprehensive assessment of how alterations to fishing gear impacts catch efficiency and species interactions. Specifically, in the context of longline fisheries, the placement of EM system-synced multiple underwater wireless or Bluetooth cameras along the mainline or attached to leaders at various depths could offer a unique opportunity to gain an increased understanding of depredation dynamics during the soaking period.

Significantly, the current study marked an innovative effort by introducing the integration of an underwater camera with a conventional EM system in a research context. This approach yielded notable improvements in acquiring discarded species information that previously was not captured by the EM system overhead cameras, resulting in more complete data for informing fisheries managers and contributing to more effective fisheries management practices.

## Supporting information

S1 Table List of the unique individual fish that contributed to the tables/counts analyzed. (PDF)

S2 Table List of the unique shark species that contributed to the tables/counts analyzed. (PDF)
