# Supplementary material for: Employing an innovative underwater camera to improve electronic monitoring in the commercial Gulf of Mexico reef fish fishery
Source: PLoS One. 2024 Mar 8;19(3):e0298588. doi: 10.1371/journal.pone.0298588 (PMC10923404; doi:10.1371/journal.pone.0298588)
Supplement: S1 Table — (PDF) [file pone.0298588.s001.pdf]

All BREP data for pub

Haul_Number	Review_Type	Species	Common_Name	Shark	Catch_Handling	Condition_On_Arrival	Catch_Fate	Sex_Of_Shark	Shark_Length_Estimate
1	NOCAM	Balistes capriscus	Gray Triggerfish	N	Brought on Board (Retained/Discarded)	Live - Healthy	Retained	Not Applicable	
1	NOCAM	Carcharhinus acronotus	Blacknose Shark	Y	Brought on Board (Retained/Discarded)	Live - Healthy	Discarded - Live and Healthy (Not Vented)	Unknown Maturity and/or Sex	Small (Less than 1m)
1	NOCAM	Carcharhinus acronotus	Blacknose Shark	Y	Brought on Board (Retained/Discarded)	Live - Healthy	Discarded - Live and Healthy (Not Vented)	Female	Medium (Between 1m and 2m)
1	NOCAM	Carcharhinus acronotus	Blacknose Shark	Y	Brought on Board (Retained/Discarded)	Live - Healthy	Discarded - Live and Healthy (Not Vented)	Female	Small (Less than 1m)
1	NOCAM	Carcharhinus plumbeus	Sandbar Shark	Y	Cut-off at Rail (No Entanglement)	Live - Healthy	Discarded - Live and Healthy (Not Vented)	Known Adult - Undetermined Sex	Large (Greater than 2m)
1	NOCAM	Epinephelus morio	Red Grouper	N	Brought on Board (Retained/Discarded)	Live - Healthy	Discarded - Live and Healthy (Not Vented)	Not Applicable	
1	NOCAM	Epinephelus morio	Red Grouper	N	Brought on Board (Retained/Discarded)	Live - Healthy	Retained	Not Applicable	
1	NOCAM	Epinephelus morio	Red Grouper	N	Brought on Board (Retained/Discarded)	Live - Healthy	Discarded - Live and Healthy (Not Vented)	Not Applicable	
1	NOCAM	Epinephelus morio	Red Grouper	N	Brought on Board (Retained/Discarded)	Live - Stomach and/or Eyes Protruding	Retained	Not Applicable	
1	NOCAM	Epinephelus morio	Red Grouper	N	Brought on Board (Retained/Discarded)	Live - Healthy	Retained	Not Applicable	
1	NOCAM	Epinephelus morio	Red Grouper	N	Brought on Board (Retained/Discarded)	Live - Stomach and/or Eyes Protruding	Discarded - Live and Healthy (Not Vented)	Not Applicable	
1	NOCAM	Epinephelus morio	Red Grouper	N	Brought on Board (Retained/Discarded)	Live - Stomach and/or Eyes Protruding	Retained	Not Applicable	
1	NOCAM	Epinephelus morio	Red Grouper	N	Brought on Board (Retained/Discarded)	Live - Stomach and/or Eyes Protruding	Retained	Not Applicable	
1	NOCAM	Epinephelus morio	Red Grouper	N	Brought on Board (Retained/Discarded)	Live - Stomach and/or Eyes Protruding	Retained	Not Applicable	
1	NOCAM	Epinephelus morio	Red Grouper	N	Brought on Board (Retained/Discarded)	Live - Healthy	Retained	Not Applicable	
1	NOCAM	Epinephelus morio	Red Grouper	N	Brought on Board (Retained/Discarded)	Live - Healthy	Retained	Not Applicable	
1	NOCAM	Epinephelus morio	Red Grouper	N	Brought on Board (Retained/Discarded)	Live - Stomach and/or Eyes Protruding	Retained	Not Applicable	
1	NOCAM	Lutjanus campechanus	Red Snapper	N	Brought on Board (Retained/Discarded)	Live - Stomach and/or Eyes Protruding	Retained	Not Applicable	
1	NOCAM	Lutjanus campechanus	Red Snapper	N	Brought on Board (Retained/Discarded)	Live - Healthy	Retained	Not Applicable	
1	NOCAM	Lutjanus campechanus	Red Snapper	N	Brought on Board (Retained/Discarded)	Live - Healthy	Retained	Not Applicable	
1	NOCAM	Lutjanus campechanus	Red Snapper	N	Brought on Board (Retained/Discarded)	Live - Healthy	Retained	Not Applicable	
1	NOCAM	Lutjanus campechanus	Red Snapper	N	Brought on Board (Retained/Discarded)	Live - Healthy	Retained	Not Applicable	
1	NOCAM	Lutjanus campechanus	Red Snapper	N	Brought on Board (Retained/Discarded)	Live - Healthy	Retained	Not Applicable	
1	NOCAM	Lutjanus campechanus	Red Snapper	N	Brought on Board (Retained/Discarded)	Live - Healthy	Retained	Not Applicable	
1	NOCAM	Lutjanus campechanus	Red Snapper	N	Brought on Board (Retained/Discarded)	Live - Healthy	Retained	Not Applicable	
1	NOCAM	Lutjanus campechanus	Red Snapper	N	Brought on Board (Retained/Discarded)	Live - Healthy	Retained	Not Applicable	
1	NOCAM	Lutjanus campechanus	Red Snapper	N	Brought on Board (Retained/Discarded)	Live - Healthy	Retained	Not Applicable	
1	NOCAM	Lutjanus campechanus	Red Snapper	N	Brought on Board (Retained/Discarded)	Live - Stomach and/or Eyes Protruding	Retained	Not Applicable	
1	NOCAM	Lutjanus campechanus	Red Snapper	N	Brought on Board (Retained/Discarded)	Live - Healthy	Retained	Not Applicable	
1	NOCAM	Lutjanus campechanus	Red Snapper	N	Brought on Board (Retained/Discarded)	Live - Healthy	Retained	Not Applicable	
1	NOCAM	Lutjanus campechanus	Red Snapper	N	Brought on Board (Retained/Discarded)	Live - Stomach and/or Eyes Protruding	Retained	Not Applicable	
1	NOCAM	Lutjanus campechanus	Red Snapper	N	Brought on Board (Retained/Discarded)	Live - Healthy	Retained	Not Applicable	
1	NOCAM	Mycteroperca microlepis	Gag Grouper	N	Brought on Board (Retained/Discarded)	Live - Healthy	Retained	Not Applicable	
1	NOCAM	Mycteroperca phenax	Scamp	N	Brought on Board (Retained/Discarded)	Live - Healthy	Retained	Not Applicable	
1	NOCAM	Rhizoprionodon terraenovae	Atlantic Sharpnose Shark	Y	Brought on Board (Retained/Discarded)	Live - Healthy	Discarded - Live and Healthy (Not Vented)	Male (Claspers)	Small (Less than 1m)
1	NOCAM	Rhizoprionodon terraenovae	Atlantic Sharpnose Shark	Y	Brought on Board (Retained/Discarded)	Live - Healthy	Discarded - Live and Healthy (Not Vented)	Female	Small (Less than 1m)
1	NOCAM	Rhizoprionodon terraenovae	Atlantic Sharpnose Shark	Y	Brought on Board (Retained/Discarded)	Live - Healthy	Discarded - Live and Healthy (Not Vented)	Female	Small (Less than 1m)
1	NOCAM	Rhizoprionodon terraenovae	Atlantic Sharpnose Shark	Y	Brought on Board (Retained/Discarded)	Live - Healthy	Discarded - Live and Healthy (Not Vented)	Female	Small (Less than 1m)
1	NOCAM	Rhizoprionodon terraenovae	Atlantic Sharpnose Shark	Y	Brought on Board (Retained/Discarded)	Live - Healthy	Discarded - Live and Healthy (Not Vented)	Female	Small (Less than 1m)
1	NOCAM	Rhizoprionodon terraenovae	Atlantic Sharpnose Shark	Y	Brought on Board (Retained/Discarded)	Live - Healthy	Discarded - Live and Healthy (Not Vented)	Female	Small (Less than 1m)
1	NOCAM	Rhizoprionodon terraenovae	Atlantic Sharpnose Shark	Y	Brought on Board (Retained/Discarded)	Live - Healthy	Discarded - Live and Healthy (Not Vented)	Male (Claspers)	Small (Less than 1m)
1	NOCAM	Tetraodontidae spp.	Pufferfish (all)	N	Brought on Board (Retained/Discarded)	Live - Healthy	Retained	Not Applicable	
1	UCAM	Balistes capriscus	Gray Triggerfish	N	Brought on Board (Retained/Discarded)	Live - Stomach and/or Eyes Protruding	Retained	Not Applicable	
1	UCAM	Carcharhinus acronotus	Blacknose Shark	Y	Brought on Board (Retained/Discarded)	Live - Healthy	Discarded - Live and Healthy (Not Vented)	Unknown Maturity and/or Sex	Small (Less than 1m)
1	UCAM	Carcharhinus acronotus	Blacknose Shark	Y	Brought on Board (Retained/Discarded)	Live - Healthy	Discarded - Live and Healthy (Not Vented)	Female	Medium (Between 1m and 2m)
1	UCAM	Carcharhinus acronotus	Blacknose Shark	Y	Brought on Board (Retained/Discarded)	Live - Healthy	Discarded - Live and Healthy (Not Vented)	Female	Small (Less than 1m)
1	UCAM	Carcharhinus plumbeus	Sandbar Shark	Y	Cut-off at Rail (No Entanglement)	Live - Healthy	Discarded - Live and Healthy (Not Vented)	Female	Large (Greater than 2m)
1	UCAM	Carcharhinus plumbeus	Sandbar Shark	Y	Cut-off at Rail (No Entanglement)	Live - Healthy	Discarded - Live and Healthy (Not Vented)	Female	Large (Greater than 2m)
1	UCAM	Epinephelus morio	Red Grouper	N	Brought on Board (Retained/Discarded)	Live - Stomach and/or Eyes Protruding	Retained	Not Applicable	
1	UCAM	Epinephelus morio	Red Grouper	N	Brought on Board (Retained/Discarded)	Live - Stomach and/or Eyes Protruding	Retained	Not Applicable	
1	UCAM	Epinephelus morio	Red Grouper	N	Brought on Board (Retained/Discarded)	Live - Stomach and/or Eyes Protruding	Retained	Not Applicable	
1	UCAM	Epinephelus morio	Red Grouper	N	Brought on Board (Retained/Discarded)	Live - Stomach and/or Eyes Protruding	Retained	Not Applicable	
1	UCAM	Epinephelus morio	Red Grouper	N	Brought on Board (Retained/Discarded)	Live - Stomach and/or Eyes Protruding	Retained	Not Applicable	
1	UCAM	Epinephelus morio	Red Grouper	N	Brought on Board (Retained/Discarded)	Live - Healthy	Discarded - Live and Healthy (Not Vented)	Not Applicable	
1	UCAM	Epinephelus morio	Red Grouper	N	Brought on Board (Retained/Discarded)	Live - Healthy	Retained	Not Applicable	
1	UCAM	Epinephelus morio	Red Grouper	N	Brought on Board (Retained/Discarded)	Live - Healthy	Retained	Not Applicable	
1	UCAM	Epinephelus morio	Red Grouper	N	Brought on Board (Retained/Discarded)	Live - Healthy	Retained	Not Applicable	
1	UCAM	Epinephelus morio	Red Grouper	N	Brought on Board (Retained/Discarded)	Live - Healthy	Retained	Not Applicable	
1	UCAM	Epinephelus morio	Red Grouper	N	Brought on Board (Retained/Discarded)	Live - Healthy	Discarded - Live and Healthy (Not Vented)	Not Applicable	
1	UCAM	Epinephelus morio	Red Grouper	N	Brought on Board (Retained/Discarded)	Live - Healthy	Retained	Not Applicable	
1	UCAM	Lutjanus campechanus	Red Snapper	N	Brought on Board (Retained/Discarded)	Live - Healthy	Retained	Not Applicable	
1	UCAM	Lutjanus campechanus	Red Snapper	N	Brought on Board (Retained/Discarded)	Live - Healthy	Retained	Not Applicable	
1	UCAM	Lutjanus campechanus	Red Snapper	N	Brought on Board (Retained/Discarded)	Live - Stomach and/or Eyes Protruding	Retained	Not Applicable	
1	UCAM	Lutjanus campechanus	Red Snapper	N	Brought on Board (Retained/Discarded)	Live - Healthy	Retained	Not Applicable	
1	UCAM	Lutjanus campechanus	Red Snapper	N	Brought on Board (Retained/Discarded)	Live - Stomach and/or Eyes Protruding	Retained	Not Applicable	
1	UCAM	Lutjanus campechanus	Red Snapper	N	Brought on Board (Retained/Discarded)	Live - Stomach and/or Eyes Protruding	Retained	Not Applicable	
1	UCAM	Lutjanus campechanus	Red Snapper	N	Brought on Board (Retained/Discarded)	Live - Stomach and/or Eyes Protruding	Retained	Not Applicable	
1	UCAM	Lutjanus campechanus	Red Snapper	N	Brought on Board (Retained/Discarded)	Live - Healthy	Retained	Not Applicable	
1	UCAM	Lutjanus campechanus	Red Snapper	N	Brought on Board (Retained/Discarded)	Live - Healthy	Retained	Not Applicable	
1	UCAM	Lutjanus campechanus	Red Snapper	N	Brought on Board (Retained/Discarded)	Live - Healthy	Retained	Not Applicable	
1	UCAM	Lutjanus campechanus	Red Snapper	N	Brought on Board (Retained/Discarded)	Live - Healthy	Retained	Not Applicable	
1	UCAM	Lutjanus campechanus	Red Snapper	N	Brought on Board (Retained/Discarded)	Live - Healthy	Retained	Not Applicable	
1	UCAM	Lutjanus campechanus	Red Snapper	N	Brought on Board (Retained/Discarded)	Live - Healthy	Retained	Not Applicable	
1	UCAM	Lutjanus campechanus	Red Snapper	N	Brought on Board (Retained/Discarded)	Live - Healthy	Retained	Not Applicable	
1	UCAM	Mycteroperca microlepis	Gag Grouper	N	Brought on Board (Retained/Discarded)	Live - Healthy	Retained	Not Applicable	
1	UCAM	Mycteroperca phenax	Scamp	N	Brought on Board (Retained/Discarded)	Live - Healthy	Retained	Not Applicable	
1	UCAM	Rhizoprionodon terraenovae	Atlantic Sharpnose Shark	Y	Brought on Board (Retained/Discarded)	Live - Healthy	Discarded - Live and Healthy (Not Vented)	Male (Claspers)	Small (Less than 1m)
1	UCAM	Rhizoprionodon terraenovae	Atlantic Sharpnose Shark	Y	Brought on Board (Retained/Discarded)	Live - Healthy	Discarded - Live and Healthy (Not Vented)	Female	Small (Less than 1m)
1	UCAM	Rhizoprionodon terraenovae	Atlantic Sharpnose Shark	Y	Brought on Board (Retained/Discarded)	Live - Healthy	Discarded - Live and Healthy (Not Vented)	Female	Small (Less than 1m)
1	UCAM	Rhizoprionodon terraenovae	Atlantic Sharpnose Shark	Y	Brought on Board (Retained/Discarded)	Live - Healthy	Discarded - Live and Healthy (Not Vented)	Female	Small (Less than 1m)
1	UCAM	Rhizoprionodon terraenovae	Atlantic Sharpnose Shark	Y	Brought on Board (Retained/Discarded)	Live - Healthy	Discarded - Live and Healthy (Not Vented)	Female	Small (Less than 1m)
1	UCAM	Rhizoprionodon terraenovae	Atlantic Sharpnose Shark	Y	Brought on Board (Retained/Discarded)	Live - Healthy	Discarded - Live and Healthy (Not Vented)	Female	Small (Less than 1m)
1	UCAM	Rhizoprionodon terraenovae	Atlantic Sharpnose Shark	Y	Brought on Board (Retained/Discarded)	Live - Healthy	Discarded - Live and Healthy (Not Vented)	Male (Claspers)	Small (Less than 1m)
1	UCAM	Tetraodontidae spp.	Pufferfish (all)	N	Brought on Board (Retained/Discarded)	Live - Healthy	Retained as Bait	Not Applicable	
1	UCAM	Trachinocephalus myops	Snakefish	N	Brought on Board (Retained/Discarded)	Live - Healthy	Unknown Fate	Not Applicable	
2	NOCAM	Balistes capriscus	Gray Triggerfish	N	Brought on Board (Retained/Discarded)	Live - Healthy	Retained	Not Applicable	
2	NOCAM	Balistes capriscus	Gray Triggerfish	N	Brought on Board (Retained/Discarded)	Live - Healthy	Retained	Not Applicable	
2	NOCAM	Balistes capriscus	Gray Triggerfish	N	Brought on Board (Retained/Discarded)	Live - Healthy	Retained	Not Applicable	
2	NOCAM	Carcharhinus acronotus	Blacknose Shark	Y	Brought on Board (Retained/Discarded)	Live - Healthy	Discarded - Live and Healthy (Not Vented)	Female	Small (Less than 1m)

2	NOCAM	Carcharhinus acronotus	Blacknose Shark	Y	Brought on Board (Retained/Discarded)	Live - Healthy	Discarded - Live and Healthy (Not Vented)	Unknown Maturity and/or Sex	Small (Less than 1m)
2	NOCAM	Carcharhinus falciformis	Silky Shark	Y	Brought on Board (Retained/Discarded)	Live - Healthy	Discarded - Live and Healthy (Not Vented)	Female	Small (Less than 1m)
2	NOCAM	Carcharhinus plumbeus	Sandbar Shark	Y	Cut-off at Rail (No Entanglement)	Live - Healthy	Discarded - Live and Healthy (Not Vented)	Known Adult - Undetermined Sex	Large (Greater than 2m)
2	NOCAM	Epinephelus morio	Red Grouper	N	Brought on Board (Retained/Discarded)	Live - Stomach and/or Eyes Protruding	Retained	Not Applicable	
2	NOCAM	Epinephelus morio	Red Grouper	N	Brought on Board (Retained/Discarded)	Live - Stomach and/or Eyes Protruding	Retained	Not Applicable	
2	NOCAM	Epinephelus morio	Red Grouper	N	Brought on Board (Retained/Discarded)	Live - Stomach and/or Eyes Protruding	Retained	Not Applicable	
2	NOCAM	Epinephelus morio	Red Grouper	N	Brought on Board (Retained/Discarded)	Live - Damaged	Retained	Not Applicable	
2	NOCAM	Epinephelus morio	Red Grouper	N	Brought on Board (Retained/Discarded)	Live - Stomach and/or Eyes Protruding	Retained	Not Applicable	
2	NOCAM	Epinephelus morio	Red Grouper	N	Brought on Board (Retained/Discarded)	Live - Stomach and/or Eyes Protruding	Discarded - Live and Healthy (Not Vented)	Not Applicable	
2	NOCAM	Epinephelus morio	Red Grouper	N	Brought on Board (Retained/Discarded)	Live - Stomach and/or Eyes Protruding	Retained	Not Applicable	
2	NOCAM	Epinephelus morio	Red Grouper	N	Brought on Board (Retained/Discarded)	Live - Stomach and/or Eyes Protruding	Retained	Not Applicable	
2	NOCAM	Epinephelus morio	Red Grouper	N	Brought on Board (Retained/Discarded)	Live - Stomach and/or Eyes Protruding	Retained	Not Applicable	
2	NOCAM	Epinephelus morio	Red Grouper	N	Brought on Board (Retained/Discarded)	Live - Stomach and/or Eyes Protruding	Retained	Not Applicable	
2	NOCAM	Epinephelus morio	Red Grouper	N	Brought on Board (Retained/Discarded)	Live - Stomach and/or Eyes Protruding	Retained	Not Applicable	
2	NOCAM	Epinephelus morio	Red Grouper	N	Brought on Board (Retained/Discarded)	Live - Healthy	Retained	Not Applicable	
2	NOCAM	Epinephelus morio	Red Grouper	N	Brought on Board (Retained/Discarded)	Live - Stomach and/or Eyes Protruding	Retained	Not Applicable	
2	NOCAM	Epinephelus morio	Red Grouper	N	Brought on Board (Retained/Discarded)	Live - Stomach and/or Eyes Protruding	Retained	Not Applicable	
2	NOCAM	Lutjanus campechanus	Red Snapper	N	Brought on Board (Retained/Discarded)	Live - Healthy	Retained	Not Applicable	
2	NOCAM	Lutjanus campechanus	Red Snapper	N	Brought on Board (Retained/Discarded)	Live - Stomach and/or Eyes Protruding	Retained	Not Applicable	
2	NOCAM	Lutjanus campechanus	Red Snapper	N	Brought on Board (Retained/Discarded)	Live - Healthy	Retained	Not Applicable	
2	NOCAM	Lutjanus campechanus	Red Snapper	N	Brought on Board (Retained/Discarded)	Live - Healthy	Retained	Not Applicable	
2	NOCAM	Lutjanus campechanus	Red Snapper	N	Brought on Board (Retained/Discarded)	Live - Stomach and/or Eyes Protruding	Retained	Not Applicable	
2	NOCAM	Lutjanus campechanus	Red Snapper	N	Brought on Board (Retained/Discarded)	Live - Healthy	Retained	Not Applicable	
2	NOCAM	Lutjanus campechanus	Red Snapper	N	Brought on Board (Retained/Discarded)	Live - Healthy	Retained	Not Applicable	
2	NOCAM	Lutjanus campechanus	Red Snapper	N	Brought on Board (Retained/Discarded)	Live - Healthy	Retained	Not Applicable	
2	NOCAM	Lutjanus campechanus	Red Snapper	N	Brought on Board (Retained/Discarded)	Live - Stomach and/or Eyes Protruding	Retained	Not Applicable	
2	NOCAM	Mycteroperca microlepis	Gag Grouper	N	Brought on Board (Retained/Discarded)	Live - Healthy	Discarded - Live and Healthy (Not Vented)	Not Applicable	
2	UCAM	Balistes capriscus	Gray Triggerfish	N	Brought on Board (Retained/Discarded)	Live - Healthy	Retained	Not Applicable	
2	UCAM	Balistes capriscus	Gray Triggerfish	N	Brought on Board (Retained/Discarded)	Live - Healthy	Retained	Not Applicable	
2	UCAM	Balistes capriscus	Gray Triggerfish	N	Brought on Board (Retained/Discarded)	Live - Healthy	Retained	Not Applicable	
2	UCAM	Carcharhinus acronotus	Blacknose Shark	Y	Brought on Board (Retained/Discarded)	Live - Healthy	Discarded - Live and Healthy (Not Vented)	Female	Small (Less than 1m)
2	UCAM	Carcharhinus acronotus	Blacknose Shark	Y	Brought on Board (Retained/Discarded)	Live - Healthy	Discarded - Live and Healthy (Not Vented)	Unknown Maturity and/or Sex	Small (Less than 1m)
2	UCAM	Carcharhinus falciformis	Silky Shark	Y	Brought on Board (Retained/Discarded)	Live - Healthy	Discarded - Live and Healthy (Not Vented)	Female	Small (Less than 1m)
2	UCAM	Carcharhinus plumbeus	Sandbar Shark	Y	Cut-off at Rail (No Entanglement)	Live - Healthy	Discarded - Live and Healthy (Not Vented)	Female	Large (Greater than 2m)
2	UCAM	Epinephelus morio	Red Grouper	N	Brought on Board (Retained/Discarded)	Live - Stomach and/or Eyes Protruding	Retained	Not Applicable	
2	UCAM	Epinephelus morio	Red Grouper	N	Brought on Board (Retained/Discarded)	Live - Stomach and/or Eyes Protruding	Retained	Not Applicable	
2	UCAM	Epinephelus morio	Red Grouper	N	Brought on Board (Retained/Discarded)	Live - Stomach and/or Eyes Protruding	Retained	Not Applicable	
2	UCAM	Epinephelus morio	Red Grouper	N	Brought on Board (Retained/Discarded)	Live - Stomach and/or Eyes Protruding	Retained	Not Applicable	
2	UCAM	Epinephelus morio	Red Grouper	N	Brought on Board (Retained/Discarded)	Live - Stomach and/or Eyes Protruding	Retained	Not Applicable	
2	UCAM	Epinephelus morio	Red Grouper	N	Brought on Board (Retained/Discarded)	Live - Stomach and/or Eyes Protruding	Discarded - Live and Healthy (Not Vented)	Not Applicable	
2	UCAM	Epinephelus morio	Red Grouper	N	Brought on Board (Retained/Discarded)	Live - Healthy	Retained	Not Applicable	
2	UCAM	Epinephelus morio	Red Grouper	N	Brought on Board (Retained/Discarded)	Live - Healthy	Retained	Not Applicable	
2	UCAM	Epinephelus morio	Red Grouper	N	Brought on Board (Retained/Discarded)	Live - Stomach and/or Eyes Protruding	Retained	Not Applicable	
2	UCAM	Epinephelus morio	Red Grouper	N	Brought on Board (Retained/Discarded)	Live - Stomach and/or Eyes Protruding	Retained	Not Applicable	
2	UCAM	Epinephelus morio	Red Grouper	N	Brought on Board (Retained/Discarded)	Live - Healthy	Retained	Not Applicable	
2	UCAM	Epinephelus morio	Red Grouper	N	Brought on Board (Retained/Discarded)	Live - Healthy	Retained	Not Applicable	
2	UCAM	Epinephelus morio	Red Grouper	N	Brought on Board (Retained/Discarded)	Live - Healthy	Retained	Not Applicable	
2	UCAM	Epinephelus morio	Red Grouper	N	Brought on Board (Retained/Discarded)	Live - Stomach and/or Eyes Protruding	Discarded - Live and Healthy (Not Vented)	Not Applicable	
2	UCAM	Epinephelus morio	Red Grouper	N	Brought on Board (Retained/Discarded)	Live - Stomach and/or Eyes Protruding	Retained	Not Applicable	
2	UCAM	Lutjanus campechanus	Red Snapper	N	Brought on Board (Retained/Discarded)	Live - Stomach and/or Eyes Protruding	Retained	Not Applicable	
2	UCAM	Lutjanus campechanus	Red Snapper	N	Brought on Board (Retained/Discarded)	Live - Healthy	Retained	Not Applicable	
2	UCAM	Lutjanus campechanus	Red Snapper	N	Brought on Board (Retained/Discarded)	Live - Healthy	Retained	Not Applicable	
2	UCAM	Lutjanus campechanus	Red Snapper	N	Brought on Board (Retained/Discarded)	Live - Stomach and/or Eyes Protruding	Retained	Not Applicable	
2	UCAM	Lutjanus campechanus	Red Snapper	N	Brought on Board (Retained/Discarded)	Dead on Arrival - Damaged	Discarded - Dead	Not Applicable	
2	UCAM	Lutjanus campechanus	Red Snapper	N	Brought on Board (Retained/Discarded)	Live - Healthy	Retained	Not Applicable	
2	UCAM	Lutjanus campechanus	Red Snapper	N	Brought on Board (Retained/Discarded)	Live - Stomach and/or Eyes Protruding	Retained	Not Applicable	
2	UCAM	Lutjanus campechanus	Red Snapper	N	Brought on Board (Retained/Discarded)	Live - Healthy	Retained	Not Applicable	
2	UCAM	Lutjanus campechanus	Red Snapper	N	Brought on Board (Retained/Discarded)	Live - Stomach and/or Eyes Protruding	Retained	Not Applicable	
2	UCAM	Mycteroperca microlepis	Gag Grouper	N	Brought on Board (Retained/Discarded)	Live - Healthy	Discarded - Live and Healthy (Not Vented)	Not Applicable	
3	NOCAM	Carcharhinus acronotus	Blacknose Shark	Y	Brought on Board (Retained/Discarded)	Live - Healthy	Discarded - Live and Healthy (Not Vented)	Female	Medium (Between 1m and 2m)
3	NOCAM	Carcharhinus falciformis	Silky Shark	Y	Brought on Board (Retained/Discarded)	Live - Healthy	Discarded - Live and Healthy (Not Vented)	Female	Small (Less than 1m)
3	NOCAM	Epinephelus morio	Red Grouper	N	Brought on Board (Retained/Discarded)	Live - Healthy	Retained	Not Applicable	
3	NOCAM	Epinephelus morio	Red Grouper	N	Brought on Board (Retained/Discarded)	Live - Healthy	Retained	Not Applicable	
3	NOCAM	Epinephelus morio	Red Grouper	N	Brought on Board (Retained/Discarded)	Live - Healthy	Retained	Not Applicable	
3	NOCAM	Epinephelus morio	Red Grouper	N	Brought on Board (Retained/Discarded)	Live - Healthy	Retained	Not Applicable	
3	NOCAM	Epinephelus morio	Red Grouper	N	Brought on Board (Retained/Discarded)	Live - Healthy	Discarded - Live and Healthy (Not Vented)	Not Applicable	
3	NOCAM	Rhizoprionodon terraenovae	Atlantic Sharpnose Shark	Y	Brought on Board (Retained/Discarded)	Live - Healthy	Discarded - Live and Healthy (Not Vented)	Male (Claspers)	Small (Less than 1m)
3	UCAM	Carcharhinus acronotus	Blacknose Shark	Y	Brought on Board (Retained/Discarded)	Live - Healthy	Discarded - Live and Healthy (Not Vented)	Female	Medium (Between 1m and 2m)
3	UCAM	Carcharhinus falciformis	Silky Shark	Y	Brought on Board (Retained/Discarded)	Live - Healthy	Discarded - Live and Healthy (Not Vented)	Female	Small (Less than 1m)
3	UCAM	Epinephelus morio	Red Grouper	N	Brought on Board (Retained/Discarded)	Live - Healthy	Retained	Not Applicable	
3	UCAM	Epinephelus morio	Red Grouper	N	Brought on Board (Retained/Discarded)	Live - Stomach and/or Eyes Protruding	Retained	Not Applicable	
3	UCAM	Epinephelus morio	Red Grouper	N	Brought on Board (Retained/Discarded)	Live - Healthy	Retained	Not Applicable	
3	UCAM	Epinephelus morio	Red Grouper	N	Brought on Board (Retained/Discarded)	Live - Healthy	Retained	Not Applicable	
3	UCAM	Epinephelus morio	Red Grouper	N	Brought on Board (Retained/Discarded)	Live - Stomach and/or Eyes Protruding	Retained	Not Applicable	
3	UCAM	Rhizoprionodon terraenovae	Atlantic Sharpnose Shark	Y	Brought on Board (Retained/Discarded)	Live - Healthy	Discarded - Live and Healthy (Not Vented)	Male (Claspers)	Small (Less than 1m)
4	NOCAM	Carcharhinus acronotus	Blacknose Shark	Y	Brought on Board (Retained/Discarded)	Live - Healthy	Discarded - Live and Healthy (Not Vented)	Unknown Maturity and/or Sex	Small (Less than 1m)
4	NOCAM	Epinephelus morio	Red Grouper	N	Brought on Board (Retained/Discarded)	Live - Stomach and/or Eyes Protruding	Retained	Not Applicable	
4	NOCAM	Epinephelus morio	Red Grouper	N	Brought on Board (Retained/Discarded)	Live - Stomach and/or Eyes Protruding	Retained	Not Applicable	
4	NOCAM	Epinephelus morio	Red Grouper	N	Brought on Board (Retained/Discarded)	Live - Healthy	Discarded - Live and Healthy (Not Vented)	Not Applicable	
4	NOCAM	Epinephelus morio	Red Grouper	N	Brought on Board (Retained/Discarded)	Live - Healthy	Discarded - Live and Healthy (Not Vented)	Not Applicable	
4	NOCAM	Epinephelus morio	Red Grouper	N	Brought on Board (Retained/Discarded)	Live - Stomach and/or Eyes Protruding	Retained	Not Applicable	
4	NOCAM	Epinephelus morio	Red Grouper	N	Brought on Board (Retained/Discarded)	Live - Healthy	Retained	Not Applicable	
4	NOCAM	Epinephelus morio	Red Grouper	N	Brought on Board (Retained/Discarded)	Live - Stomach and/or Eyes Protruding	Retained	Not Applicable	
4	NOCAM	Epinephelus morio	Red Grouper	N	Brought on Board (Retained/Discarded)	Live - Healthy	Retained	Not Applicable	
4	NOCAM	Epinephelus morio	Red Grouper	N	Brought on Board (Retained/Discarded)	Live - Healthy	Retained	Not Applicable	
4	NOCAM	Epinephelus morio	Red Grouper	N	Brought on Board (Retained/Discarded)	Live - Stomach and/or Eyes Protruding	Retained	Not Applicable	
4	NOCAM	Epinephelus morio	Red Grouper	N	Brought on Board (Retained/Discarded)	Dead on Arrival - Damaged	Discarded - Dead	Not Applicable	
4	NOCAM	Epinephelus morio	Red Grouper	N	Brought on Board (Retained/Discarded)	Live - Healthy	Retained	Not Applicable	
4	NOCAM	Epinephelus morio	Red Grouper	N	Brought on Board (Retained/Discarded)	Live - Damaged	Discarded - Live and Damaged (Not Ventec	Not Applicable	
4	NOCAM	Epinephelus morio	Red Grouper	N	Brought on Board (Retained/Discarded)	Live - Healthy	Retained	Not Applicable	
4	NOCAM	Epinephelus morio	Red Grouper	N	Brought on Board (Retained/Discarded)	Live - Stomach and/or Eyes Protruding	Retained	Not Applicable	
4	NOCAM	Ginglymostoma cirratum	Nurse Shark	Y	Cut-off at Rail (No Entanglement)	Live - Healthy	Discarded - Live and Healthy (Not Vented)	Male (Claspers)	Large (Greater than 2m)

4	NOCAM	Ginglymostoma cirratum	Nurse Shark	Y	Cut-off at Rail (No Entanglement)	Live - Healthy	Discarded - Live and Healthy (Not Vented)	Known Adult - Undetermined Sex	Large (Greater than 2m)
4	NOCAM	Lutjanus campechanus	Red Snapper	N	Brought on Board (Retained/Discarded)	Live - Healthy	Retained	Not Applicable	
4	NOCAM	Lutjanus campechanus	Red Snapper	N	Brought on Board (Retained/Discarded)	Live - Healthy	Retained	Not Applicable	
4	NOCAM	Lutjanus campechanus	Red Snapper	N	Brought on Board (Retained/Discarded)	Live - Healthy	Retained	Not Applicable	
4	NOCAM	Lutjanus campechanus	Red Snapper	N	Brought on Board (Retained/Discarded)	Dead on Arrival - Damaged	Discarded - Dead	Not Applicable	
4	NOCAM	Lutjanus campechanus	Red Snapper	N	Brought on Board (Retained/Discarded)	Live - Healthy	Retained	Not Applicable	
4	NOCAM	Lutjanus campechanus	Red Snapper	N	Brought on Board (Retained/Discarded)	Live - Healthy	Retained	Not Applicable	
4	NOCAM	Mycteroperca microlepis	Gag Grouper	N	Brought on Board (Retained/Discarded)	Live - Healthy	Retained	Not Applicable	
4	NOCAM	Mycteroperca microlepis	Gag Grouper	N	Brought on Board (Retained/Discarded)	Live - Healthy	Retained	Not Applicable	
4	UCAM	Carcharhinus acronotus	Blacknose Shark	Y	Brought on Board (Retained/Discarded)	Live - Healthy	Discarded - Live and Healthy (Not Vented)	Female	Small (Less than 1m)
4	UCAM	Epinephelus morio	Red Grouper	N	Brought on Board (Retained/Discarded)	Live - Healthy	Discarded - Live and Healthy (Not Vented)	Not Applicable	
4	UCAM	Epinephelus morio	Red Grouper	N	Brought on Board (Retained/Discarded)	Live - Stomach and/or Eyes Protruding	Discarded - Live and Healthy (Not Vented)	Not Applicable	
4	UCAM	Epinephelus morio	Red Grouper	N	Brought on Board (Retained/Discarded)	Live - Healthy	Retained	Not Applicable	
4	UCAM	Epinephelus morio	Red Grouper	N	Brought on Board (Retained/Discarded)	Live - Stomach and/or Eyes Protruding	Retained	Not Applicable	
4	UCAM	Epinephelus morio	Red Grouper	N	Brought on Board (Retained/Discarded)	Live - Stomach and/or Eyes Protruding	Retained	Not Applicable	
4	UCAM	Epinephelus morio	Red Grouper	N	Brought on Board (Retained/Discarded)	Live - Stomach and/or Eyes Protruding	Retained	Not Applicable	
4	UCAM	Epinephelus morio	Red Grouper	N	Brought on Board (Retained/Discarded)	Live - Stomach and/or Eyes Protruding	Retained	Not Applicable	
4	UCAM	Epinephelus morio	Red Grouper	N	Brought on Board (Retained/Discarded)	Dead on Arrival - Damaged	Discarded - Dead	Not Applicable	
4	UCAM	Epinephelus morio	Red Grouper	N	Brought on Board (Retained/Discarded)	Live - Healthy	Retained	Not Applicable	
4	UCAM	Epinephelus morio	Red Grouper	N	Brought on Board (Retained/Discarded)	Live - Stomach and/or Eyes Protruding	Retained	Not Applicable	
4	UCAM	Epinephelus morio	Red Grouper	N	Brought on Board (Retained/Discarded)	Live - Healthy	Discarded - Live and Healthy (Not Vented)	Not Applicable	
4	UCAM	Epinephelus morio	Red Grouper	N	Brought on Board (Retained/Discarded)	Live - Stomach and/or Eyes Protruding	Retained	Not Applicable	
4	UCAM	Epinephelus morio	Red Grouper	N	Brought on Board (Retained/Discarded)	Live - Damaged	Discarded - Live and Damaged (Not Ventec	Not Applicable	
4	UCAM	Epinephelus morio	Red Grouper	N	Brought on Board (Retained/Discarded)	Live - Stomach and/or Eyes Protruding	Retained	Not Applicable	
4	UCAM	Ginglymostoma cirratum	Nurse Shark	Y	Cut-off at Rail (No Entanglement)	Live - Healthy	Discarded - Live and Healthy (Not Vented)	Male (Claspers)	Large (Greater than 2m)
4	UCAM	Ginglymostoma cirratum	Nurse Shark	Y	Cut-off at Rail (No Entanglement)	Live - Healthy	Discarded - Live and Healthy (Not Vented)	Male (Claspers)	Large (Greater than 2m)
4	UCAM	Lutjanus campechanus	Red Snapper	N	Brought on Board (Retained/Discarded)	Dead on Arrival - Damaged	Discarded - Dead	Not Applicable	
4	UCAM	Lutjanus campechanus	Red Snapper	N	Brought on Board (Retained/Discarded)	Live - Healthy	Retained	Not Applicable	
4	UCAM	Lutjanus campechanus	Red Snapper	N	Brought on Board (Retained/Discarded)	Live - Healthy	Retained	Not Applicable	
4	UCAM	Lutjanus campechanus	Red Snapper	N	Brought on Board (Retained/Discarded)	Live - Healthy	Retained	Not Applicable	
4	UCAM	Lutjanus campechanus	Red Snapper	N	Brought on Board (Retained/Discarded)	Live - Healthy	Retained	Not Applicable	
4	UCAM	Lutjanus campechanus	Red Snapper	N	Brought on Board (Retained/Discarded)	Live - Stomach and/or Eyes Protruding	Retained	Not Applicable	
4	UCAM	Mycteroperca microlepis	Gag Grouper	N	Brought on Board (Retained/Discarded)	Live - Healthy	Retained	Not Applicable	
4	UCAM	Mycteroperca microlepis	Gag Grouper	N	Brought on Board (Retained/Discarded)	Live - Healthy	Retained	Not Applicable	
5	NOCAM	Carcharhinus signatus	Night Shark	Y	Brought on Board (Retained/Discarded)	Live - Healthy	Discarded - Live and Healthy (Not Vented)	Known Juvenile	Medium (Between 1m and 2m)
5	NOCAM	Caulolatilus microps	Blueline Tilefish	N	Brought on Board (Retained/Discarded)	Live - Stomach and/or Eyes Protruding	Retained	Not Applicable	
5	NOCAM	Caulolatilus microps	Blueline Tilefish	N	Brought on Board (Retained/Discarded)	Live - Healthy	Retained	Not Applicable	
5	NOCAM	Caulolatilus microps	Blueline Tilefish	N	Brought on Board (Retained/Discarded)	Live - Healthy	Retained	Not Applicable	
5	NOCAM	Caulolatilus microps	Blueline Tilefish	N	Brought on Board (Retained/Discarded)	Live - Healthy	Retained	Not Applicable	
5	NOCAM	Caulolatilus microps	Blueline Tilefish	N	Brought on Board (Retained/Discarded)	Live - Stomach and/or Eyes Protruding	Unknown Fate	Not Applicable	
5	NOCAM	Caulolatilus microps	Blueline Tilefish	N	Brought on Board (Retained/Discarded)	Live - Healthy	Retained	Not Applicable	
5	NOCAM	Caulolatilus microps	Blueline Tilefish	N	Brought on Board (Retained/Discarded)	Live - Healthy	Retained	Not Applicable	
5	NOCAM	Caulolatilus microps	Blueline Tilefish	N	Brought on Board (Retained/Discarded)	Live - Healthy	Retained	Not Applicable	
5	NOCAM	Caulolatilus microps	Blueline Tilefish	N	Brought on Board (Retained/Discarded)	Live - Healthy	Retained	Not Applicable	
5	NOCAM	Caulolatilus microps	Blueline Tilefish	N	Brought on Board (Retained/Discarded)	Live - Healthy	Retained	Not Applicable	
5	NOCAM	Caulolatilus microps	Blueline Tilefish	N	Brought on Board (Retained/Discarded)	Live - Healthy	Retained	Not Applicable	
5	NOCAM	Caulolatilus microps	Blueline Tilefish	N	Brought on Board (Retained/Discarded)	Live - Stomach and/or Eyes Protruding	Retained	Not Applicable	
5	NOCAM	Caulolatilus microps	Blueline Tilefish	N	Brought on Board (Retained/Discarded)	Live - Stomach and/or Eyes Protruding	Retained	Not Applicable	
5	NOCAM	Crab, unidentified	Crab, Unidentified	N	Brought on Board (Retained/Discarded)	Live - Healthy	Discarded - Live and Healthy (Not Vented)	Not Applicable	
5	NOCAM	Crab, unidentified	Crab, Unidentified	N	Brought on Board (Retained/Discarded)	Live - Healthy	Discarded - Live and Healthy (Not Vented)	Not Applicable	
5	NOCAM	Crab, unidentified	Crab, Unidentified	N	Brought on Board (Retained/Discarded)	Live - Healthy	Discarded - Live and Healthy (Not Vented)	Not Applicable	
5	NOCAM	Epinephelus flavolimbatus	Yellowedge Grouper	N	Brought on Board (Retained/Discarded)	Live - Stomach and/or Eyes Protruding	Retained	Not Applicable	
5	NOCAM	Epinephelus flavolimbatus	Yellowedge Grouper	N	Brought on Board (Retained/Discarded)	Live - Stomach and/or Eyes Protruding	Retained	Not Applicable	
5	NOCAM	Epinephelus flavolimbatus	Yellowedge Grouper	N	Brought on Board (Retained/Discarded)	Live - Stomach and/or Eyes Protruding	Retained	Not Applicable	
5	NOCAM	Epinephelus flavolimbatus	Yellowedge Grouper	N	Brought on Board (Retained/Discarded)	Live - Stomach and/or Eyes Protruding	Retained	Not Applicable	
5	NOCAM	Epinephelus flavolimbatus	Yellowedge Grouper	N	Brought on Board (Retained/Discarded)	Live - Stomach and/or Eyes Protruding	Retained	Not Applicable	
5	NOCAM	Epinephelus flavolimbatus	Yellowedge Grouper	N	Brought on Board (Retained/Discarded)	Live - Healthy	Retained	Not Applicable	
5	NOCAM	Epinephelus flavolimbatus	Yellowedge Grouper	N	Brought on Board (Retained/Discarded)	Live - Stomach and/or Eyes Protruding	Retained	Not Applicable	
5	NOCAM	Epinephelus flavolimbatus	Yellowedge Grouper	N	Brought on Board (Retained/Discarded)	Live - Healthy	Retained	Not Applicable	
5	NOCAM	Epinephelus flavolimbatus	Yellowedge Grouper	N	Brought on Board (Retained/Discarded)	Live - Stomach and/or Eyes Protruding	Retained	Not Applicable	
5	NOCAM	Epinephelus flavolimbatus	Yellowedge Grouper	N	Brought on Board (Retained/Discarded)	Live - Stomach and/or Eyes Protruding	Retained	Not Applicable	
5	NOCAM	Epinephelus flavolimbatus	Yellowedge Grouper	N	Brought on Board (Retained/Discarded)	Live - Stomach and/or Eyes Protruding	Retained	Not Applicable	
5	NOCAM	Epinephelus flavolimbatus	Yellowedge Grouper	N	Brought on Board (Retained/Discarded)	Live - Stomach and/or Eyes Protruding	Retained	Not Applicable	
5	NOCAM	Epinephelus flavolimbatus	Yellowedge Grouper	N	Brought on Board (Retained/Discarded)	Live - Stomach and/or Eyes Protruding	Retained	Not Applicable	
5	NOCAM	Epinephelus flavolimbatus	Yellowedge Grouper	N	Brought on Board (Retained/Discarded)	Live - Stomach and/or Eyes Protruding	Retained	Not Applicable	
5	NOCAM	Epinephelus flavolimbatus	Yellowedge Grouper	N	Brought on Board (Retained/Discarded)	Live - Stomach and/or Eyes Protruding	Retained	Not Applicable	
5	NOCAM	Mustelus spp.	Dogfish, Smooth (Florida)	Y	Brought on Board (Retained/Discarded)	Live - Healthy	Discarded - Live and Healthy (Not Vented)	Female	Medium (Between 1m and 2m)
5	NOCAM	Mustelus spp.	Dogfish, Smooth (Florida)	Y	Brought on Board (Retained/Discarded)	Live - Healthy	Discarded - Live and Healthy (Not Vented)	Male (Claspers)	Small (Less than 1m)
5	NOCAM	Mustelus spp.	Dogfish, Smooth (Florida)	Y	Brought on Board (Retained/Discarded)	Live - Healthy	Discarded - Live and Healthy (Not Vented)	Female	Small (Less than 1m)
5	UCAM	Carcharhinus signatus	Night Shark	Y	Brought on Board (Retained/Discarded)	Live - Healthy	Discarded - Live and Healthy (Not Vented)	Known Juvenile	Medium (Between 1m and 2m)
5	UCAM	Caulolatilus microps	Blueline Tilefish	N	Brought on Board (Retained/Discarded)	Live - Healthy	Retained	Not Applicable	
5	UCAM	Caulolatilus microps	Blueline Tilefish	N	Brought on Board (Retained/Discarded)	Live - Healthy	Retained	Not Applicable	
5	UCAM	Caulolatilus microps	Blueline Tilefish	N	Brought on Board (Retained/Discarded)	Live - Stomach and/or Eyes Protruding	Unknown Fate	Not Applicable	
5	UCAM	Caulolatilus microps	Blueline Tilefish	N	Brought on Board (Retained/Discarded)	Live - Healthy	Retained	Not Applicable	
5	UCAM	Caulolatilus microps	Blueline Tilefish	N	Brought on Board (Retained/Discarded)	Live - Stomach and/or Eyes Protruding	Retained	Not Applicable	
5	UCAM	Caulolatilus microps	Blueline Tilefish	N	Brought on Board (Retained/Discarded)	Live - Healthy	Retained	Not Applicable	
5	UCAM	Caulolatilus microps	Blueline Tilefish	N	Brought on Board (Retained/Discarded)	Live - Stomach and/or Eyes Protruding	Retained	Not Applicable	
5	UCAM	Caulolatilus microps	Blueline Tilefish	N	Brought on Board (Retained/Discarded)	Live - Stomach and/or Eyes Protruding	Retained	Not Applicable	
5	UCAM	Caulolatilus microps	Blueline Tilefish	N	Brought on Board (Retained/Discarded)	Live - Healthy	Retained	Not Applicable	
5	UCAM	Caulolatilus microps	Blueline Tilefish	N	Brought on Board (Retained/Discarded)	Live - Healthy	Retained	Not Applicable	
5	UCAM	Caulolatilus microps	Blueline Tilefish	N	Brought on Board (Retained/Discarded)	Live - Healthy	Retained	Not Applicable	
5	UCAM	Caulolatilus microps	Blueline Tilefish	N	Brought on Board (Retained/Discarded)	Live - Healthy	Retained	Not Applicable	
5	UCAM	Caulolatilus microps	Blueline Tilefish	N	Brought on Board (Retained/Discarded)	Live - Healthy	Retained	Not Applicable	
5	UCAM	Crab, unidentified	Crab, Unidentified	N	Brought on Board (Retained/Discarded)	Live - Healthy	Discarded - Live and Healthy (Not Vented)	Not Applicable	
5	UCAM	Crab, unidentified	Crab, Unidentified	N	Brought on Board (Retained/Discarded)	Live - Healthy	Discarded - Live and Healthy (Not Vented)	Not Applicable	
5	UCAM	Crab, unidentified	Crab, Unidentified	N	Brought on Board (Retained/Discarded)	Live - Healthy	Discarded - Live and Healthy (Not Vented)	Not Applicable	
5	UCAM	Epinephelus flavolimbatus	Yellowedge Grouper	N	Brought on Board (Retained/Discarded)	Live - Stomach and/or Eyes Protruding	Retained	Not Applicable	
5	UCAM	Epinephelus flavolimbatus	Yellowedge Grouper	N	Brought on Board (Retained/Discarded)	Live - Stomach and/or Eyes Protruding	Retained	Not Applicable	
5	UCAM	Epinephelus flavolimbatus	Yellowedge Grouper	N	Brought on Board (Retained/Discarded)	Live - Stomach and/or Eyes Protruding	Retained	Not Applicable	
5	UCAM	Epinephelus flavolimbatus	Yellowedge Grouper	N	Brought on Board (Retained/Discarded)	Live - Stomach and/or Eyes Protruding	Retained	Not Applicable	
5	UCAM	Epinephelus flavolimbatus	Yellowedge Grouper	N	Brought on Board (Retained/Discarded)	Live - Stomach and/or Eyes Protruding	Retained	Not Applicable	
5	UCAM	Epinephelus flavolimbatus	Yellowedge Grouper	N	Brought on Board (Retained/Discarded)	Live - Stomach and/or Eyes Protruding	Retained	Not Applicable	

5	UCAM	Epinephelus flavolimbatus	Yellowedge Grouper	N	Brought on Board (Retained/Discarded)	Live - Stomach and/or Eyes Protruding	Retained	Not Applicable	
5	UCAM	Epinephelus flavolimbatus	Yellowedge Grouper	N	Brought on Board (Retained/Discarded)	Live - Stomach and/or Eyes Protruding	Retained	Not Applicable	
5	UCAM	Epinephelus flavolimbatus	Yellowedge Grouper	N	Brought on Board (Retained/Discarded)	Live - Healthy	Retained	Not Applicable	
5	UCAM	Epinephelus flavolimbatus	Yellowedge Grouper	N	Brought on Board (Retained/Discarded)	Live - Stomach and/or Eyes Protruding	Retained	Not Applicable	
5	UCAM	Epinephelus flavolimbatus	Yellowedge Grouper	N	Brought on Board (Retained/Discarded)	Live - Stomach and/or Eyes Protruding	Retained	Not Applicable	
5	UCAM	Epinephelus flavolimbatus	Yellowedge Grouper	N	Brought on Board (Retained/Discarded)	Live - Healthy	Retained	Not Applicable	
5	UCAM	Epinephelus flavolimbatus	Yellowedge Grouper	N	Brought on Board (Retained/Discarded)	Live - Healthy	Retained	Not Applicable	
5	UCAM	Epinephelus flavolimbatus	Yellowedge Grouper	N	Brought on Board (Retained/Discarded)	Live - Stomach and/or Eyes Protruding	Retained	Not Applicable	
5	UCAM	Epinephelus flavolimbatus	Yellowedge Grouper	N	Brought on Board (Retained/Discarded)	Live - Stomach and/or Eyes Protruding	Retained	Not Applicable	
5	UCAM	Mustelus spp.	Dogfish, Smooth (Florida)	Y	Brought on Board (Retained/Discarded)	Live - Healthy	Discarded - Live and Healthy (Not Vented)	Female	Medium (Between 1m and 2m)
5	UCAM	Mustelus spp.	Dogfish, Smooth (Florida)	Y	Brought on Board (Retained/Discarded)	Live - Healthy	Discarded - Live and Healthy (Not Vented)	Male (Claspers)	Small (Less than 1m)
5	UCAM	Mustelus spp.	Dogfish, Smooth (Florida)	Y	Brought on Board (Retained/Discarded)	Live - Healthy	Discarded - Live and Healthy (Not Vented)	Female	Small (Less than 1m)
6	NOCAM	Epinephelus niveatus	Snowy Grouper	N	Brought on Board (Retained/Discarded)	Live - Healthy	Retained	Not Applicable	
6	NOCAM	Epinephelus niveatus	Snowy Grouper	N	Brought on Board (Retained/Discarded)	Live - Stomach and/or Eyes Protruding	Retained	Not Applicable	
6	NOCAM	Epinephelus niveatus	Snowy Grouper	N	Brought on Board (Retained/Discarded)	Live - Stomach and/or Eyes Protruding	Retained	Not Applicable	
6	NOCAM	Epinephelus niveatus	Snowy Grouper	N	Brought on Board (Retained/Discarded)	Live - Stomach and/or Eyes Protruding	Retained	Not Applicable	
6	NOCAM	Epinephelus niveatus	Snowy Grouper	N	Brought on Board (Retained/Discarded)	Live - Stomach and/or Eyes Protruding	Retained	Not Applicable	
6	NOCAM	Epinephelus niveatus	Snowy Grouper	N	Brought on Board (Retained/Discarded)	Live - Stomach and/or Eyes Protruding	Retained	Not Applicable	
6	NOCAM	Epinephelus niveatus	Snowy Grouper	N	Brought on Board (Retained/Discarded)	Live - Stomach and/or Eyes Protruding	Retained	Not Applicable	
6	NOCAM	Epinephelus niveatus	Snowy Grouper	N	Brought on Board (Retained/Discarded)	Live - Stomach and/or Eyes Protruding	Retained	Not Applicable	
6	NOCAM	Epinephelus niveatus	Snowy Grouper	N	Brought on Board (Retained/Discarded)	Live - Stomach and/or Eyes Protruding	Retained	Not Applicable	
6	NOCAM	Epinephelus niveatus	Snowy Grouper	N	Brought on Board (Retained/Discarded)	Live - Stomach and/or Eyes Protruding	Retained	Not Applicable	
6	NOCAM	Epinephelus niveatus	Snowy Grouper	N	Brought on Board (Retained/Discarded)	Live - Stomach and/or Eyes Protruding	Retained	Not Applicable	
6	NOCAM	Istiophorus albicans	Sailfish, Atlantic	N	Brought on Board (Retained/Discarded)	Dead on Arrival - Undamaged	Discarded - Dead	Not Applicable	
6	NOCAM	Lopholatilus chamaelonticeps	Tilefish, Golden	N	Brought on Board (Retained/Discarded)	Live - Healthy	Retained	Not Applicable	
6	NOCAM	Lopholatilus chamaelonticeps	Tilefish, Golden	N	Brought on Board (Retained/Discarded)	Live - Healthy	Retained	Not Applicable	
6	NOCAM	Lopholatilus chamaelonticeps	Tilefish, Golden	N	Brought on Board (Retained/Discarded)	Live - Healthy	Retained	Not Applicable	
6	NOCAM	Lopholatilus chamaelonticeps	Tilefish, Golden	N	Brought on Board (Retained/Discarded)	Live - Healthy	Retained	Not Applicable	
6	NOCAM	Lopholatilus chamaelonticeps	Tilefish, Golden	N	Brought on Board (Retained/Discarded)	Live - Healthy	Retained	Not Applicable	
6	NOCAM	Lopholatilus chamaelonticeps	Tilefish, Golden	N	Brought on Board (Retained/Discarded)	Live - Healthy	Retained	Not Applicable	
6	NOCAM	Lopholatilus chamaelonticeps	Tilefish, Golden	N	Brought on Board (Retained/Discarded)	Live - Healthy	Retained	Not Applicable	
6	NOCAM	Lopholatilus chamaelonticeps	Tilefish, Golden	N	Brought on Board (Retained/Discarded)	Live - Stomach and/or Eyes Protruding	Retained	Not Applicable	
6	NOCAM	Lopholatilus chamaelonticeps	Tilefish, Golden	N	Brought on Board (Retained/Discarded)	Live - Stomach and/or Eyes Protruding	Retained	Not Applicable	
6	NOCAM	Lopholatilus chamaelonticeps	Tilefish, Golden	N	Brought on Board (Retained/Discarded)	Live - Stomach and/or Eyes Protruding	Retained	Not Applicable	
6	UCAM	Epinephelus niveatus	Snowy Grouper	N	Brought on Board (Retained/Discarded)	Live - Stomach and/or Eyes Protruding	Retained	Not Applicable	
6	UCAM	Epinephelus niveatus	Snowy Grouper	N	Not Handled (Dropped Off)	Live - Healthy	Discarded - Live and Healthy (Not Vented)	Not Applicable	
6	UCAM	Epinephelus niveatus	Snowy Grouper	N	Brought on Board (Retained/Discarded)	Live - Stomach and/or Eyes Protruding	Retained	Not Applicable	
6	UCAM	Epinephelus niveatus	Snowy Grouper	N	Brought on Board (Retained/Discarded)	Live - Stomach and/or Eyes Protruding	Retained	Not Applicable	
6	UCAM	Epinephelus niveatus	Snowy Grouper	N	Brought on Board (Retained/Discarded)	Live - Stomach and/or Eyes Protruding	Retained	Not Applicable	
6	UCAM	Epinephelus niveatus	Snowy Grouper	N	Brought on Board (Retained/Discarded)	Live - Stomach and/or Eyes Protruding	Retained	Not Applicable	
6	UCAM	Epinephelus niveatus	Snowy Grouper	N	Brought on Board (Retained/Discarded)	Live - Stomach and/or Eyes Protruding	Retained	Not Applicable	
6	UCAM	Epinephelus niveatus	Snowy Grouper	N	Brought on Board (Retained/Discarded)	Live - Stomach and/or Eyes Protruding	Retained	Not Applicable	
6	UCAM	Epinephelus niveatus	Snowy Grouper	N	Brought on Board (Retained/Discarded)	Live - Stomach and/or Eyes Protruding	Retained	Not Applicable	
6	UCAM	Epinephelus niveatus	Snowy Grouper	N	Brought on Board (Retained/Discarded)	Live - Stomach and/or Eyes Protruding	Retained	Not Applicable	
6	UCAM	Epinephelus niveatus	Snowy Grouper	N	Brought on Board (Retained/Discarded)	Live - Stomach and/or Eyes Protruding	Retained	Not Applicable	
6	UCAM	Epinephelus niveatus	Snowy Grouper	N	Brought on Board (Retained/Discarded)	Live - Stomach and/or Eyes Protruding	Retained	Not Applicable	
6	UCAM	Epinephelus niveatus	Snowy Grouper	N	Brought on Board (Retained/Discarded)	Live - Stomach and/or Eyes Protruding	Retained	Not Applicable	
6	UCAM	Istiophorus albicans	Sailfish, Atlantic	N	Brought on Board (Retained/Discarded)	Dead on Arrival - Undamaged	Discarded - Dead	Not Applicable	
6	UCAM	Lopholatilus chamaelonticeps	Tilefish, Golden	N	Brought on Board (Retained/Discarded)	Live - Healthy	Retained	Not Applicable	
6	UCAM	Lopholatilus chamaelonticeps	Tilefish, Golden	N	Brought on Board (Retained/Discarded)	Live - Healthy	Retained	Not Applicable	
6	UCAM	Lopholatilus chamaelonticeps	Tilefish, Golden	N	Brought on Board (Retained/Discarded)	Live - Healthy	Retained	Not Applicable	
6	UCAM	Lopholatilus chamaelonticeps	Tilefish, Golden	N	Brought on Board (Retained/Discarded)	Live - Healthy	Retained	Not Applicable	
6	UCAM	Lopholatilus chamaelonticeps	Tilefish, Golden	N	Brought on Board (Retained/Discarded)	Live - Healthy	Retained	Not Applicable	
6	UCAM	Lopholatilus chamaelonticeps	Tilefish, Golden	N	Brought on Board (Retained/Discarded)	Live - Stomach and/or Eyes Protruding	Retained	Not Applicable	
6	UCAM	Lopholatilus chamaelonticeps	Tilefish, Golden	N	Brought on Board (Retained/Discarded)	Live - Healthy	Retained	Not Applicable	
6	UCAM	Lopholatilus chamaelonticeps	Tilefish, Golden	N	Brought on Board (Retained/Discarded)	Live - Healthy	Retained	Not Applicable	
6	UCAM	Lopholatilus chamaelonticeps	Tilefish, Golden	N	Brought on Board (Retained/Discarded)	Live - Stomach and/or Eyes Protruding	Retained	Not Applicable	
6	UCAM	Lopholatilus chamaelonticeps	Tilefish, Golden	N	Brought on Board (Retained/Discarded)	Live - Healthy	Retained	Not Applicable	
6	UCAM	Lopholatilus chamaelonticeps	Tilefish, Golden	N	Brought on Board (Retained/Discarded)	Live - Stomach and/or Eyes Protruding	Retained	Not Applicable	
7	NOCAM	Acanthocybius solanderi	Wahoo	N	Brought on Board (Retained/Discarded)	Dead on Arrival - Undamaged	Retained	Not Applicable	
7	NOCAM	Caulolatilus microps	Blueline Tilefish	N	Brought on Board (Retained/Discarded)	Live - Stomach and/or Eyes Protruding	Retained	Not Applicable	
7	NOCAM	Caulolatilus microps	Blueline Tilefish	N	Brought on Board (Retained/Discarded)	Live - Healthy	Retained	Not Applicable	
7	NOCAM	Caulolatilus microps	Blueline Tilefish	N	Brought on Board (Retained/Discarded)	Live - Stomach and/or Eyes Protruding	Retained	Not Applicable	
7	NOCAM	Caulolatilus microps	Blueline Tilefish	N	Brought on Board (Retained/Discarded)	Live - Healthy	Retained	Not Applicable	
7	NOCAM	Caulolatilus microps	Blueline Tilefish	N	Brought on Board (Retained/Discarded)	Live - Stomach and/or Eyes Protruding	Retained	Not Applicable	
7	NOCAM	Caulolatilus microps	Blueline Tilefish	N	Brought on Board (Retained/Discarded)	Live - Healthy	Retained	Not Applicable	
7	NOCAM	Caulolatilus microps	Blueline Tilefish	N	Brought on Board (Retained/Discarded)	Live - Healthy	Retained	Not Applicable	
7	NOCAM	Caulolatilus microps	Blueline Tilefish	N	Brought on Board (Retained/Discarded)	Live - Stomach and/or Eyes Protruding	Retained	Not Applicable	
7	NOCAM	Caulolatilus microps	Blueline Tilefish	N	Brought on Board (Retained/Discarded)	Live - Stomach and/or Eyes Protruding	Retained	Not Applicable	
7	NOCAM	Caulolatilus microps	Blueline Tilefish	N	Brought on Board (Retained/Discarded)	Live - Healthy	Retained	Not Applicable	
7	NOCAM	Caulolatilus microps	Blueline Tilefish	N	Brought on Board (Retained/Discarded)	Live - Healthy	Retained	Not Applicable	
7	NOCAM	Caulolatilus microps	Blueline Tilefish	N	Brought on Board (Retained/Discarded)	Live - Healthy	Retained	Not Applicable	
7	NOCAM	Caulolatilus microps	Blueline Tilefish	N	Brought on Board (Retained/Discarded)	Live - Healthy	Retained	Not Applicable	
7	NOCAM	Caulolatilus microps	Blueline Tilefish	N	Brought on Board (Retained/Discarded)	Live - Stomach and/or Eyes Protruding	Retained	Not Applicable	
7	NOCAM	Crab, unidentified	Crab, Unidentified	N	Brought on Board (Retained/Discarded)	Live - Healthy	Discarded - Live and Healthy (Not Vented)	Not Applicable	
7	NOCAM	Epinephelus flavolimbatus	Yellowedge Grouper	N	Brought on Board (Retained/Discarded)	Live - Stomach and/or Eyes Protruding	Retained	Not Applicable	
7	NOCAM	Epinephelus flavolimbatus	Yellowedge Grouper	N	Brought on Board (Retained/Discarded)	Live - Stomach and/or Eyes Protruding	Retained	Not Applicable	
7	NOCAM	Epinephelus flavolimbatus	Yellowedge Grouper	N	Brought on Board (Retained/Discarded)	Live - Stomach and/or Eyes Protruding	Retained	Not Applicable	
7	NOCAM	Epinephelus flavolimbatus	Yellowedge Grouper	N	Brought on Board (Retained/Discarded)	Live - Healthy	Retained	Not Applicable	
7	NOCAM	Epinephelus flavolimbatus	Yellowedge Grouper	N	Brought on Board (Retained/Discarded)	Live - Stomach and/or Eyes Protruding	Retained	Not Applicable	
7	NOCAM	Epinephelus flavolimbatus	Yellowedge Grouper	N	Brought on Board (Retained/Discarded)	Live - Stomach and/or Eyes Protruding	Retained	Not Applicable	
7	NOCAM	Epinephelus flavolimbatus	Yellowedge Grouper	N	Brought on Board (Retained/Discarded)	Live - Stomach and/or Eyes Protruding	Retained	Not Applicable	
7	NOCAM	Mustelus spp.	Dogfish, Smooth (Florida)	Y	Brought on Board (Retained/Discarded)	Live - Healthy	Discarded - Live and Healthy (Not Vented)	Unknown Maturity and/or Sex	Medium (Between 1m and 2m)
7	NOCAM	Sphyraena barracuda	Barracuda (Great)	N	Brought on Board (Retained/Discarded)	Dead on Arrival - Undamaged	Unknown Fate	Not Applicable	
7	UCAM	Acanthocybius solanderi	Wahoo	N	Brought on Board (Retained/Discarded)	Dead on Arrival - Undamaged	Retained	Not Applicable	
7	UCAM	Caulolatilus microps	Blueline Tilefish	N	Brought on Board (Retained/Discarded)	Live - Stomach and/or Eyes Protruding	Retained	Not Applicable	
7	UCAM	Caulolatilus microps	Blueline Tilefish	N	Brought on Board (Retained/Discarded)	Live - Stomach and/or Eyes Protruding	Retained	Not Applicable	
7	UCAM	Caulolatilus microps	Blueline Tilefish	N	Brought on Board (Retained/Discarded)	Live - Stomach and/or Eyes Protruding	Retained	Not Applicable	
7	UCAM	Caulolatilus microps	Blueline Tilefish	N	Brought on Board (Retained/Discarded)	Live - Healthy	Retained	Not Applicable	
7	UCAM	Caulolatilus microps	Blueline Tilefish	N	Brought on Board (Retained/Discarded)	Live - Healthy	Retained	Not Applicable	
7	UCAM	Caulolatilus microps	Blueline Tilefish	N	Brought on Board (Retained/Discarded)	Live - Healthy	Retained	Not Applicable	
7	UCAM	Caulolatilus microps	Blueline Tilefish	N	Brought on Board (Retained/Discarded)	Live - Healthy	Retained	Not Applicable	

7	UCAM	Caulolatilus microps	Blueline Tilefish	N	Brought on Board (Retained/Discarded)	Live - Healthy	Retained	Not Applicable	
7	UCAM	Caulolatilus microps	Blueline Tilefish	N	Brought on Board (Retained/Discarded)	Live - Healthy	Retained	Not Applicable	
7	UCAM	Caulolatilus microps	Blueline Tilefish	N	Brought on Board (Retained/Discarded)	Live - Healthy	Retained	Not Applicable	
7	UCAM	Caulolatilus microps	Blueline Tilefish	N	Brought on Board (Retained/Discarded)	Live - Healthy	Retained	Not Applicable	
7	UCAM	Caulolatilus microps	Blueline Tilefish	N	Brought on Board (Retained/Discarded)	Live - Healthy	Retained	Not Applicable	
7	UCAM	Caulolatilus microps	Blueline Tilefish	N	Brought on Board (Retained/Discarded)	Live - Stomach and/or Eyes Protruding	Retained	Not Applicable	
7	UCAM	Crab, unidentified	Crab, Unidentified	N	Brought on Board (Retained/Discarded)	Live - Healthy	Discarded - Live and Healthy (Not Vented)	Not Applicable	
7	UCAM	Epinephelus flavolimbatus	Yellowedge Grouper	N	Brought on Board (Retained/Discarded)	Live - Stomach and/or Eyes Protruding	Retained	Not Applicable	
7	UCAM	Epinephelus flavolimbatus	Yellowedge Grouper	N	Brought on Board (Retained/Discarded)	Live - Stomach and/or Eyes Protruding	Retained	Not Applicable	
7	UCAM	Epinephelus flavolimbatus	Yellowedge Grouper	N	Brought on Board (Retained/Discarded)	Live - Stomach and/or Eyes Protruding	Retained	Not Applicable	
7	UCAM	Epinephelus flavolimbatus	Yellowedge Grouper	N	Brought on Board (Retained/Discarded)	Live - Stomach and/or Eyes Protruding	Retained	Not Applicable	
7	UCAM	Epinephelus flavolimbatus	Yellowedge Grouper	N	Brought on Board (Retained/Discarded)	Live - Stomach and/or Eyes Protruding	Retained	Not Applicable	
7	UCAM	Epinephelus flavolimbatus	Yellowedge Grouper	N	Brought on Board (Retained/Discarded)	Live - Stomach and/or Eyes Protruding	Retained	Not Applicable	
7	UCAM	Epinephelus flavolimbatus	Yellowedge Grouper	N	Brought on Board (Retained/Discarded)	Live - Stomach and/or Eyes Protruding	Retained	Not Applicable	
7	UCAM	Epinephelus flavolimbatus	Yellowedge Grouper	N	Not Handled (Dropped Off)	Live - Healthy	Discarded - Live and Healthy (Not Vented)	Not Applicable	
7	UCAM	Mustelus spp.	Dogfish, Smooth (Florida)	Y	Brought on Board (Retained/Discarded)	Live - Healthy	Discarded - Live and Healthy (Not Vented)	Female	Medium (Between 1m and 2m)
7	UCAM	Sphyraena barracuda	Barracuda (Great)	N	Brought on Board (Retained/Discarded)	Dead on Arrival - Undamaged	Unknown Fate	Not Applicable	
8	NOCAM	Caulolatilus microps	Blueline Tilefish	N	Brought on Board (Retained/Discarded)	Live - Healthy	Retained	Not Applicable	
8	NOCAM	Caulolatilus microps	Blueline Tilefish	N	Brought on Board (Retained/Discarded)	Live - Healthy	Retained	Not Applicable	
8	NOCAM	Caulolatilus microps	Blueline Tilefish	N	Brought on Board (Retained/Discarded)	Live - Stomach and/or Eyes Protruding	Retained	Not Applicable	
8	NOCAM	Caulolatilus microps	Blueline Tilefish	N	Brought on Board (Retained/Discarded)	Live - Stomach and/or Eyes Protruding	Retained	Not Applicable	
8	NOCAM	Caulolatilus microps	Blueline Tilefish	N	Brought on Board (Retained/Discarded)	Live - Stomach and/or Eyes Protruding	Retained	Not Applicable	
8	NOCAM	Caulolatilus microps	Blueline Tilefish	N	Brought on Board (Retained/Discarded)	Live - Healthy	Retained	Not Applicable	
8	NOCAM	Caulolatilus microps	Blueline Tilefish	N	Brought on Board (Retained/Discarded)	Live - Healthy	Retained	Not Applicable	
8	NOCAM	Caulolatilus microps	Blueline Tilefish	N	Brought on Board (Retained/Discarded)	Live - Healthy	Retained	Not Applicable	
8	NOCAM	Caulolatilus microps	Blueline Tilefish	N	Brought on Board (Retained/Discarded)	Live - Healthy	Retained	Not Applicable	
8	NOCAM	Caulolatilus microps	Blueline Tilefish	N	Brought on Board (Retained/Discarded)	Live - Healthy	Retained	Not Applicable	
8	NOCAM	Caulolatilus microps	Blueline Tilefish	N	Brought on Board (Retained/Discarded)	Live - Healthy	Retained	Not Applicable	
8	NOCAM	Caulolatilus microps	Blueline Tilefish	N	Brought on Board (Retained/Discarded)	Live - Healthy	Retained	Not Applicable	
8	NOCAM	Caulolatilus microps	Blueline Tilefish	N	Brought on Board (Retained/Discarded)	Live - Stomach and/or Eyes Protruding	Retained	Not Applicable	
8	NOCAM	Caulolatilus microps	Blueline Tilefish	N	Brought on Board (Retained/Discarded)	Live - Healthy	Retained	Not Applicable	
8	NOCAM	Caulolatilus microps	Blueline Tilefish	N	Brought on Board (Retained/Discarded)	Live - Healthy	Retained	Not Applicable	
8	NOCAM	Caulolatilus microps	Blueline Tilefish	N	Brought on Board (Retained/Discarded)	Live - Healthy	Retained	Not Applicable	
8	NOCAM	Caulolatilus microps	Blueline Tilefish	N	Brought on Board (Retained/Discarded)	Live - Healthy	Retained	Not Applicable	
8	NOCAM	Caulolatilus microps	Blueline Tilefish	N	Brought on Board (Retained/Discarded)	Live - Healthy	Retained	Not Applicable	
8	NOCAM	Caulolatilus microps	Blueline Tilefish	N	Brought on Board (Retained/Discarded)	Live - Stomach and/or Eyes Protruding	Retained	Not Applicable	
8	NOCAM	Epinephelus flavolimbatus	Yellowedge Grouper	N	Brought on Board (Retained/Discarded)	Live - Stomach and/or Eyes Protruding	Retained	Not Applicable	
8	NOCAM	Epinephelus flavolimbatus	Yellowedge Grouper	N	Brought on Board (Retained/Discarded)	Live - Healthy	Retained	Not Applicable	
8	NOCAM	Epinephelus flavolimbatus	Yellowedge Grouper	N	Brought on Board (Retained/Discarded)	Live - Stomach and/or Eyes Protruding	Retained	Not Applicable	
8	NOCAM	Epinephelus flavolimbatus	Yellowedge Grouper	N	Brought on Board (Retained/Discarded)	Live - Stomach and/or Eyes Protruding	Retained	Not Applicable	
8	NOCAM	Epinephelus flavolimbatus	Yellowedge Grouper	N	Brought on Board (Retained/Discarded)	Live - Stomach and/or Eyes Protruding	Retained	Not Applicable	
8	NOCAM	Epinephelus flavolimbatus	Yellowedge Grouper	N	Brought on Board (Retained/Discarded)	Live - Stomach and/or Eyes Protruding	Retained	Not Applicable	
8	NOCAM	Epinephelus flavolimbatus	Yellowedge Grouper	N	Brought on Board (Retained/Discarded)	Live - Stomach and/or Eyes Protruding	Retained	Not Applicable	
8	NOCAM	Epinephelus flavolimbatus	Yellowedge Grouper	N	Brought on Board (Retained/Discarded)	Live - Stomach and/or Eyes Protruding	Retained	Not Applicable	
8	NOCAM	Epinephelus flavolimbatus	Yellowedge Grouper	N	Brought on Board (Retained/Discarded)	Live - Stomach and/or Eyes Protruding	Retained	Not Applicable	
8	NOCAM	Epinephelus flavolimbatus	Yellowedge Grouper	N	Brought on Board (Retained/Discarded)	Live - Stomach and/or Eyes Protruding	Retained	Not Applicable	
8	NOCAM	Epinephelus flavolimbatus	Yellowedge Grouper	N	Brought on Board (Retained/Discarded)	Live - Healthy	Retained	Not Applicable	
8	NOCAM	Epinephelus flavolimbatus	Yellowedge Grouper	N	Brought on Board (Retained/Discarded)	Live - Stomach and/or Eyes Protruding	Retained	Not Applicable	
8	NOCAM	Epinephelus flavolimbatus	Yellowedge Grouper	N	Brought on Board (Retained/Discarded)	Live - Healthy	Retained	Not Applicable	
8	NOCAM	Epinephelus flavolimbatus	Yellowedge Grouper	N	Brought on Board (Retained/Discarded)	Live - Stomach and/or Eyes Protruding	Retained	Not Applicable	
8	NOCAM	Epinephelus flavolimbatus	Yellowedge Grouper	N	Brought on Board (Retained/Discarded)	Live - Healthy	Retained	Not Applicable	
8	NOCAM	Epinephelus flavolimbatus	Yellowedge Grouper	N	Brought on Board (Retained/Discarded)	Live - Stomach and/or Eyes Protruding	Retained	Not Applicable	
8	NOCAM	Muraenidae, unidentified	Moray Eel, Unidentified	N	Brought on Board (Retained/Discarded)	Live - Healthy	Retained as Bait	Not Applicable	
8	NOCAM	Mustelus spp.	Dogfish, Smooth (Florida)	Y	Brought on Board (Retained/Discarded)	Live - Healthy	Discarded - Live and Healthy (Not Vented)	Female	Small (Less than 1m)
8	UCAM	Caulolatilus microps	Blueline Tilefish	N	Brought on Board (Retained/Discarded)	Live - Healthy	Retained	Not Applicable	
8	UCAM	Caulolatilus microps	Blueline Tilefish	N	Brought on Board (Retained/Discarded)	Live - Stomach and/or Eyes Protruding	Retained	Not Applicable	
8	UCAM	Caulolatilus microps	Blueline Tilefish	N	Brought on Board (Retained/Discarded)	Live - Healthy	Retained	Not Applicable	
8	UCAM	Caulolatilus microps	Blueline Tilefish	N	Brought on Board (Retained/Discarded)	Live - Stomach and/or Eyes Protruding	Retained	Not Applicable	
8	UCAM	Caulolatilus microps	Blueline Tilefish	N	Brought on Board (Retained/Discarded)	Live - Healthy	Retained	Not Applicable	
8	UCAM	Caulolatilus microps	Blueline Tilefish	N	Brought on Board (Retained/Discarded)	Live - Healthy	Retained	Not Applicable	
8	UCAM	Caulolatilus microps	Blueline Tilefish	N	Brought on Board (Retained/Discarded)	Live - Stomach and/or Eyes Protruding	Retained	Not Applicable	
8	UCAM	Caulolatilus microps	Blueline Tilefish	N	Brought on Board (Retained/Discarded)	Live - Healthy	Retained	Not Applicable	
8	UCAM	Caulolatilus microps	Blueline Tilefish	N	Brought on Board (Retained/Discarded)	Live - Healthy	Retained	Not Applicable	
8	UCAM	Caulolatilus microps	Blueline Tilefish	N	Brought on Board (Retained/Discarded)	Live - Stomach and/or Eyes Protruding	Retained	Not Applicable	
8	UCAM	Caulolatilus microps	Blueline Tilefish	N	Brought on Board (Retained/Discarded)	Live - Healthy	Retained	Not Applicable	
8	UCAM	Caulolatilus microps	Blueline Tilefish	N	Brought on Board (Retained/Discarded)	Live - Healthy	Retained	Not Applicable	
8	UCAM	Caulolatilus microps	Blueline Tilefish	N	Brought on Board (Retained/Discarded)	Live - Stomach and/or Eyes Protruding	Retained	Not Applicable	
8	UCAM	Caulolatilus microps	Blueline Tilefish	N	Brought on Board (Retained/Discarded)	Live - Stomach and/or Eyes Protruding	Retained	Not Applicable	
8	UCAM	Caulolatilus microps	Blueline Tilefish	N	Brought on Board (Retained/Discarded)	Live - Healthy	Retained	Not Applicable	
8	UCAM	Caulolatilus microps	Blueline Tilefish	N	Brought on Board (Retained/Discarded)	Live - Stomach and/or Eyes Protruding	Retained	Not Applicable	
8	UCAM	Caulolatilus microps	Blueline Tilefish	N	Brought on Board (Retained/Discarded)	Live - Healthy	Retained	Not Applicable	
8	UCAM	Caulolatilus microps	Blueline Tilefish	N	Brought on Board (Retained/Discarded)	Live - Healthy	Retained	Not Applicable	
8	UCAM	Caulolatilus microps	Blueline Tilefish	N	Brought on Board (Retained/Discarded)	Live - Healthy	Retained	Not Applicable	
8	UCAM	Caulolatilus microps	Blueline Tilefish	N	Brought on Board (Retained/Discarded)	Live - Stomach and/or Eyes Protruding	Retained	Not Applicable	
8	UCAM	Epinephelus flavolimbatus	Yellowedge Grouper	N	Brought on Board (Retained/Discarded)	Live - Healthy	Retained	Not Applicable	
8	UCAM	Epinephelus flavolimbatus	Yellowedge Grouper	N	Brought on Board (Retained/Discarded)	Live - Stomach and/or Eyes Protruding	Retained	Not Applicable	
8	UCAM	Epinephelus flavolimbatus	Yellowedge Grouper	N	Brought on Board (Retained/Discarded)	Live - Stomach and/or Eyes Protruding	Retained	Not Applicable	
8	UCAM	Epinephelus flavolimbatus	Yellowedge Grouper	N	Brought on Board (Retained/Discarded)	Live - Stomach and/or Eyes Protruding	Retained	Not Applicable	
8	UCAM	Epinephelus flavolimbatus	Yellowedge Grouper	N	Brought on Board (Retained/Discarded)	Live - Stomach and/or Eyes Protruding	Retained	Not Applicable	
8	UCAM	Epinephelus flavolimbatus	Yellowedge Grouper	N	Brought on Board (Retained/Discarded)	Live - Stomach and/or Eyes Protruding	Retained	Not Applicable	
8	UCAM	Epinephelus flavolimbatus	Yellowedge Grouper	N	Brought on Board (Retained/Discarded)	Live - Stomach and/or Eyes Protruding	Retained	Not Applicable	
8	UCAM	Epinephelus flavolimbatus	Yellowedge Grouper	N	Brought on Board (Retained/Discarded)	Live - Stomach and/or Eyes Protruding	Retained	Not Applicable	
8	UCAM	Epinephelus flavolimbatus	Yellowedge Grouper	N	Brought on Board (Retained/Discarded)	Live - Stomach and/or Eyes Protruding	Retained	Not Applicable	
8	UCAM	Epinephelus flavolimbatus	Yellowedge Grouper	N	Brought on Board (Retained/Discarded)	Live - Stomach and/or Eyes Protruding	Retained	Not Applicable	
8	UCAM	Epinephelus flavolimbatus	Yellowedge Grouper	N	Brought on Board (Retained/Discarded)	Live - Stomach and/or Eyes Protruding	Retained	Not Applicable	
8	UCAM	Epinephelus flavolimbatus	Yellowedge Grouper	N	Brought on Board (Retained/Discarded)	Live - Stomach and/or Eyes Protruding	Retained	Not Applicable	
8	UCAM	Epinephelus flavolimbatus	Yellowedge Grouper	N	Brought on Board (Retained/Discarded)	Live - Healthy	Retained	Not Applicable	
8	UCAM	Epinephelus flavolimbatus	Yellowedge Grouper	N	Brought on Board (Retained/Discarded)	Live - Stomach and/or Eyes Protruding	Retained	Not Applicable	
8	UCAM	Epinephelus flavolimbatus	Yellowedge Grouper	N	Brought on Board (Retained/Discarded)	Live - Healthy	Retained	Not Applicable	
8	UCAM	Muraenidae, unidentified	Moray Eel, Unidentified	N	Brought on Board (Retained/Discarded)	Live - Healthy	Retained as Bait	Not Applicable	
8	UCAM	Mustelus spp.	Dogfish, Smooth (Florida)	Y	Brought on Board (Retained/Discarded)	Live - Healthy	Discarded - Live and Healthy (Not Vented)	Female	Small (Less than 1m)
9	NOCAM	Carcharhinus signatus	Night Shark	Y	Brought on Board (Retained/Discarded)	Live - Healthy	Discarded - Live and Healthy (Not Vented)	Female	Small (Less than 1m)

9	NOCAM	Caulolatilus microps	Blueline Tilefish	N	Brought on Board (Retained/Discarded)	Live - Stomach and/or Eyes Protruding	Retained	Not Applicable	
9	NOCAM	Caulolatilus microps	Blueline Tilefish	N	Brought on Board (Retained/Discarded)	Live - Healthy	Retained	Not Applicable	
9	NOCAM	Caulolatilus microps	Blueline Tilefish	N	Brought on Board (Retained/Discarded)	Live - Healthy	Retained	Not Applicable	
9	NOCAM	Caulolatilus microps	Blueline Tilefish	N	Brought on Board (Retained/Discarded)	Live - Healthy	Retained	Not Applicable	
9	NOCAM	Caulolatilus microps	Blueline Tilefish	N	Brought on Board (Retained/Discarded)	Live - Stomach and/or Eyes Protruding	Retained	Not Applicable	
9	NOCAM	Epinephelus flavolimbatus	Yellowedge Grouper	N	Brought on Board (Retained/Discarded)	Live - Stomach and/or Eyes Protruding	Retained	Not Applicable	
9	NOCAM	Epinephelus flavolimbatus	Yellowedge Grouper	N	Brought on Board (Retained/Discarded)	Live - Stomach and/or Eyes Protruding	Retained	Not Applicable	
9	NOCAM	Epinephelus flavolimbatus	Yellowedge Grouper	N	Brought on Board (Retained/Discarded)	Live - Stomach and/or Eyes Protruding	Retained	Not Applicable	
9	NOCAM	Epinephelus flavolimbatus	Yellowedge Grouper	N	Brought on Board (Retained/Discarded)	Live - Stomach and/or Eyes Protruding	Retained	Not Applicable	
9	NOCAM	Epinephelus flavolimbatus	Yellowedge Grouper	N	Brought on Board (Retained/Discarded)	Live - Stomach and/or Eyes Protruding	Retained	Not Applicable	
9	NOCAM	Epinephelus flavolimbatus	Yellowedge Grouper	N	Brought on Board (Retained/Discarded)	Live - Stomach and/or Eyes Protruding	Retained	Not Applicable	
9	NOCAM	Epinephelus flavolimbatus	Yellowedge Grouper	N	Brought on Board (Retained/Discarded)	Live - Stomach and/or Eyes Protruding	Retained	Not Applicable	
9	NOCAM	Epinephelus flavolimbatus	Yellowedge Grouper	N	Brought on Board (Retained/Discarded)	Live - Stomach and/or Eyes Protruding	Retained	Not Applicable	
9	NOCAM	Epinephelus flavolimbatus	Yellowedge Grouper	N	Brought on Board (Retained/Discarded)	Live - Stomach and/or Eyes Protruding	Retained	Not Applicable	
9	NOCAM	Epinephelus flavolimbatus	Yellowedge Grouper	N	Brought on Board (Retained/Discarded)	Live - Stomach and/or Eyes Protruding	Retained	Not Applicable	
9	NOCAM	Epinephelus flavolimbatus	Yellowedge Grouper	N	Brought on Board (Retained/Discarded)	Live - Stomach and/or Eyes Protruding	Retained	Not Applicable	
9	NOCAM	Epinephelus flavolimbatus	Yellowedge Grouper	N	Brought on Board (Retained/Discarded)	Live - Stomach and/or Eyes Protruding	Retained	Not Applicable	
9	NOCAM	Epinephelus flavolimbatus	Yellowedge Grouper	N	Brought on Board (Retained/Discarded)	Live - Stomach and/or Eyes Protruding	Retained	Not Applicable	
9	NOCAM	Epinephelus flavolimbatus	Yellowedge Grouper	N	Brought on Board (Retained/Discarded)	Live - Stomach and/or Eyes Protruding	Retained	Not Applicable	
9	NOCAM	Epinephelus flavolimbatus	Yellowedge Grouper	N	Brought on Board (Retained/Discarded)	Live - Stomach and/or Eyes Protruding	Retained	Not Applicable	
9	NOCAM	Epinephelus flavolimbatus	Yellowedge Grouper	N	Brought on Board (Retained/Discarded)	Live - Stomach and/or Eyes Protruding	Retained	Not Applicable	
9	NOCAM	Epinephelus flavolimbatus	Yellowedge Grouper	N	Brought on Board (Retained/Discarded)	Live - Stomach and/or Eyes Protruding	Retained	Not Applicable	
9	NOCAM	Epinephelus flavolimbatus	Yellowedge Grouper	N	Brought on Board (Retained/Discarded)	Live - Stomach and/or Eyes Protruding	Retained	Not Applicable	
9	NOCAM	Epinephelus flavolimbatus	Yellowedge Grouper	N	Brought on Board (Retained/Discarded)	Live - Stomach and/or Eyes Protruding	Retained	Not Applicable	
9	NOCAM	Epinephelus flavolimbatus	Yellowedge Grouper	N	Brought on Board (Retained/Discarded)	Live - Stomach and/or Eyes Protruding	Retained	Not Applicable	
9	NOCAM	Muraenidae, unidentified	Moray Eel, Unidentified	N	Brought on Board (Retained/Discarded)	Live - Healthy	Retained as Bait	Not Applicable	
9	NOCAM	Mustelus spp.	Dogfish, Smooth (Florida)	Y	Brought on Board (Retained/Discarded)	Live - Healthy	Discarded - Live and Healthy (Not Vented)	Male (Claspers)	Small (Less than 1m)
9	NOCAM	Mustelus spp.	Dogfish, Smooth (Florida)	Y	Brought on Board (Retained/Discarded)	Live - Healthy	Discarded - Live and Healthy (Not Vented)	Male (Claspers)	Medium (Between 1m and 2m)
9	NOCAM	Mustelus spp.	Dogfish, Smooth (Florida)	Y	Brought on Board (Retained/Discarded)	Live - Healthy	Discarded - Live and Healthy (Not Vented)	Unknown Maturity and/or Sex	Small (Less than 1m)
9	NOCAM	Mustelus spp.	Dogfish, Smooth (Florida)	Y	Brought on Board (Retained/Discarded)	Live - Healthy	Discarded - Live and Healthy (Not Vented)	Female	Small (Less than 1m)
9	NOCAM	Mustelus spp.	Dogfish, Smooth (Florida)	Y	Brought on Board (Retained/Discarded)	Live - Healthy	Discarded - Live and Healthy (Not Vented)	Female	Medium (Between 1m and 2m)
9	NOCAM	Seriola rivoliana	Almaco Jack	N	Brought on Board (Retained/Discarded)	Live - Healthy	Discarded - Live and Healthy (Not Vented)	Not Applicable	
9	NOCAM	Sphyraena barracuda	Barracuda (Great)	N	Brought on Board (Retained/Discarded)	Dead on Arrival - Undamaged	Unknown Fate	Not Applicable	
9	UCAM	Carcharhinus signatus	Night Shark	Y	Brought on Board (Retained/Discarded)	Live - Healthy	Discarded - Live and Healthy (Not Vented)	Female	Small (Less than 1m)
9	UCAM	Caulolatilus microps	Blueline Tilefish	N	Brought on Board (Retained/Discarded)	Live - Stomach and/or Eyes Protruding	Retained	Not Applicable	
9	UCAM	Caulolatilus microps	Blueline Tilefish	N	Brought on Board (Retained/Discarded)	Live - Healthy	Retained	Not Applicable	
9	UCAM	Caulolatilus microps	Blueline Tilefish	N	Brought on Board (Retained/Discarded)	Live - Stomach and/or Eyes Protruding	Retained	Not Applicable	
9	UCAM	Caulolatilus microps	Blueline Tilefish	N	Brought on Board (Retained/Discarded)	Live - Stomach and/or Eyes Protruding	Retained	Not Applicable	
9	UCAM	Caulolatilus microps	Blueline Tilefish	N	Brought on Board (Retained/Discarded)	Live - Healthy	Retained	Not Applicable	
9	UCAM	Caulolatilus microps	Blueline Tilefish	N	Brought on Board (Retained/Discarded)	Live - Healthy	Retained	Not Applicable	
9	UCAM	Caulolatilus microps	Blueline Tilefish	N	Brought on Board (Retained/Discarded)	Live - Healthy	Retained	Not Applicable	
9	UCAM	Caulolatilus microps	Blueline Tilefish	N	Brought on Board (Retained/Discarded)	Live - Healthy	Retained	Not Applicable	
9	UCAM	Epinephelus flavolimbatus	Yellowedge Grouper	N	Brought on Board (Retained/Discarded)	Live - Stomach and/or Eyes Protruding	Retained	Not Applicable	
9	UCAM	Epinephelus flavolimbatus	Yellowedge Grouper	N	Brought on Board (Retained/Discarded)	Live - Stomach and/or Eyes Protruding	Retained	Not Applicable	
9	UCAM	Epinephelus flavolimbatus	Yellowedge Grouper	N	Brought on Board (Retained/Discarded)	Live - Stomach and/or Eyes Protruding	Retained	Not Applicable	
9	UCAM	Epinephelus flavolimbatus	Yellowedge Grouper	N	Brought on Board (Retained/Discarded)	Live - Stomach and/or Eyes Protruding	Retained	Not Applicable	
9	UCAM	Epinephelus flavolimbatus	Yellowedge Grouper	N	Brought on Board (Retained/Discarded)	Live - Stomach and/or Eyes Protruding	Retained	Not Applicable	
9	UCAM	Epinephelus flavolimbatus	Yellowedge Grouper	N	Brought on Board (Retained/Discarded)	Live - Stomach and/or Eyes Protruding	Retained	Not Applicable	
9	UCAM	Epinephelus flavolimbatus	Yellowedge Grouper	N	Brought on Board (Retained/Discarded)	Live - Stomach and/or Eyes Protruding	Retained	Not Applicable	
9	UCAM	Epinephelus flavolimbatus	Yellowedge Grouper	N	Brought on Board (Retained/Discarded)	Live - Stomach and/or Eyes Protruding	Retained	Not Applicable	
9	UCAM	Epinephelus flavolimbatus	Yellowedge Grouper	N	Brought on Board (Retained/Discarded)	Live - Stomach and/or Eyes Protruding	Retained	Not Applicable	
9	UCAM	Epinephelus flavolimbatus	Yellowedge Grouper	N	Brought on Board (Retained/Discarded)	Live - Stomach and/or Eyes Protruding	Retained	Not Applicable	
9	UCAM	Epinephelus flavolimbatus	Yellowedge Grouper	N	Not Handled (Dropped Off)	Live - Stomach and/or Eyes Protruding	Discarded - Live and Healthy (Not Vented)	Not Applicable	
9	UCAM	Epinephelus flavolimbatus	Yellowedge Grouper	N	Brought on Board (Retained/Discarded)	Live - Stomach and/or Eyes Protruding	Retained	Not Applicable	
9	UCAM	Epinephelus flavolimbatus	Yellowedge Grouper	N	Brought on Board (Retained/Discarded)	Live - Stomach and/or Eyes Protruding	Retained	Not Applicable	
9	UCAM	Epinephelus flavolimbatus	Yellowedge Grouper	N	Brought on Board (Retained/Discarded)	Live - Stomach and/or Eyes Protruding	Retained	Not Applicable	
9	UCAM	Epinephelus flavolimbatus	Yellowedge Grouper	N	Brought on Board (Retained/Discarded)	Live - Stomach and/or Eyes Protruding	Retained	Not Applicable	
9	UCAM	Epinephelus flavolimbatus	Yellowedge Grouper	N	Brought on Board (Retained/Discarded)	Live - Stomach and/or Eyes Protruding	Retained	Not Applicable	
9	UCAM	Epinephelus flavolimbatus	Yellowedge Grouper	N	Brought on Board (Retained/Discarded)	Live - Stomach and/or Eyes Protruding	Retained	Not Applicable	
9	UCAM	Epinephelus flavolimbatus	Yellowedge Grouper	N	Brought on Board (Retained/Discarded)	Live - Stomach and/or Eyes Protruding	Retained	Not Applicable	
9	UCAM	Epinephelus flavolimbatus	Yellowedge Grouper	N	Brought on Board (Retained/Discarded)	Live - Stomach and/or Eyes Protruding	Retained	Not Applicable	
9	UCAM	Epinephelus flavolimbatus	Yellowedge Grouper	N	Brought on Board (Retained/Discarded)	Live - Stomach and/or Eyes Protruding	Retained	Not Applicable	
9	UCAM	Epinephelus flavolimbatus	Yellowedge Grouper	N	Brought on Board (Retained/Discarded)	Live - Stomach and/or Eyes Protruding	Retained	Not Applicable	
9	UCAM	Epinephelus flavolimbatus	Yellowedge Grouper	N	Brought on Board (Retained/Discarded)	Live - Stomach and/or Eyes Protruding	Retained	Not Applicable	
9	UCAM	Epinephelus flavolimbatus	Yellowedge Grouper	N	Brought on Board (Retained/Discarded)	Live - Stomach and/or Eyes Protruding	Retained	Not Applicable	
9	UCAM	Muraenidae, unidentified	Moray Eel, Unidentified	N	Brought on Board (Retained/Discarded)	Live - Healthy	Retained as Bait	Not Applicable	
9	UCAM	Mustelus spp.	Dogfish, Smooth (Florida)	Y	Brought on Board (Retained/Discarded)	Live - Healthy	Discarded - Live and Healthy (Not Vented)	Male (Claspers)	Medium (Between 1m and 2m)
9	UCAM	Mustelus spp.	Dogfish, Smooth (Florida)	Y	Brought on Board (Retained/Discarded)	Live - Healthy	Discarded - Live and Healthy (Not Vented)	Female	Small (Less than 1m)
9	UCAM	Mustelus spp.	Dogfish, Smooth (Florida)	Y	Brought on Board (Retained/Discarded)	Live - Healthy	Discarded - Live and Healthy (Not Vented)	Unknown Maturity and/or Sex	Small (Less than 1m)
9	UCAM	Mustelus spp.	Dogfish, Smooth (Florida)	Y	Brought on Board (Retained/Discarded)	Live - Healthy	Discarded - Live and Healthy (Not Vented)	Male (Claspers)	Small (Less than 1m)
9	UCAM	Mustelus spp.	Dogfish, Smooth (Florida)	Y	Brought on Board (Retained/Discarded)	Live - Healthy	Discarded - Live and Healthy (Not Vented)	Female	Medium (Between 1m and 2m)
9	UCAM	Seriola rivoliana	Almaco Jack	N	Brought on Board (Retained/Discarded)	Live - Healthy	Discarded - Live and Healthy (Not Vented)	Not Applicable	
9	UCAM	Sphyraena barracuda	Barracuda (Great)	N	Brought on Board (Retained/Discarded)	Dead on Arrival - Undamaged	Unknown Fate	Not Applicable	
10	NOCAM	Epinephelus flavolimbatus	Yellowedge Grouper	N	Brought on Board (Retained/Discarded)	Live - Healthy	Retained	Not Applicable	
10	NOCAM	Epinephelus flavolimbatus	Yellowedge Grouper	N	Brought on Board (Retained/Discarded)	Live - Stomach and/or Eyes Protruding	Retained	Not Applicable	
10	UCAM	Epinephelus flavolimbatus	Yellowedge Grouper	N	Brought on Board (Retained/Discarded)	Live - Stomach and/or Eyes Protruding	Retained	Not Applicable	
10	UCAM	Epinephelus flavolimbatus	Yellowedge Grouper	N	Brought on Board (Retained/Discarded)	Live - Stomach and/or Eyes Protruding	Retained	Not Applicable	
11	NOCAM	Carcharhinus signatus	Night Shark	Y	Brought on Board (Retained/Discarded)	Live - Healthy	Discarded - Live and Healthy (Not Vented)	Female	Small (Less than 1m)
11	NOCAM	Carcharhinus signatus	Night Shark	Y	Brought on Board (Retained/Discarded)	Live - Healthy	Discarded - Live and Healthy (Not Vented)	Female	Medium (Between 1m and 2m)
11	NOCAM	Carcharhinus signatus	Night Shark	Y	Brought on Board (Retained/Discarded)	Live - Healthy	Discarded - Live and Healthy (Not Vented)	Known Juvenile	Small (Less than 1m)
11	NOCAM	Caulolatilus microps	Blueline Tilefish	N	Brought on Board (Retained/Discarded)	Live - Stomach and/or Eyes Protruding	Retained	Not Applicable	
11	NOCAM	Caulolatilus microps	Blueline Tilefish	N	Brought on Board (Retained/Discarded)	Live - Stomach and/or Eyes Protruding	Retained	Not Applicable	
11	NOCAM	Epinephelus niveatus	Snowy Grouper	N	Brought on Board (Retained/Discarded)	Live - Stomach and/or Eyes Protruding	Retained	Not Applicable	
11	NOCAM	Epinephelus niveatus	Snowy Grouper	N	Brought on Board (Retained/Discarded)	Live - Stomach and/or Eyes Protruding	Retained	Not Applicable	
11	NOCAM	Epinephelus niveatus	Snowy Grouper	N	Brought on Board (Retained/Discarded)	Live - Stomach and/or Eyes Protruding	Retained	Not Applicable	
11	NOCAM	Epinephelus niveatus	Snowy Grouper	N	Brought on Board (Retained/Discarded)	Live - Stomach and/or Eyes Protruding	Retained	Not Applicable	
11	NOCAM	Epinephelus niveatus	Snowy Grouper	N	Brought on Board (Retained/Discarded)	Live - Stomach and/or Eyes Protruding	Retained	Not Applicable	
11	NOCAM	Helicolenus dactylopterus	Blackbelly Rosefish	N	Brought on Board (Retained/Discarded)	Live - Healthy	Retained	Not Applicable	
11	NOCAM	Helicolenus dactylopterus	Blackbelly Rosefish	N	Brought on Board (Retained/Discarded)	Live - Healthy	Retained	Not Applicable	
11	NOCAM	Helicolenus dactylopterus	Blackbelly Rosefish	N	Brought on Board (Retained/Discarded)	Live - Healthy	Retained	Not Applicable	
11	NOCAM	Lopholatilus chamaeleonticeps	Tilefish, Golden	N	Brought on Board (Retained/Discarded)	Live - Healthy	Retained	Not Applicable	
11	UCAM	Carcharhinus signatus	Night Shark	Y	Brought on Board (Retained/Discarded)	Live - Healthy	Discarded - Live and Healthy (Not Vented)	Female	Medium (Between 1m and 2m)

11	UCAM	Carcharhinus signatus	Night Shark	Y	Brought on Board (Retained/Discarded)	Live - Healthy	Discarded - Live and Healthy (Not Vented)	Known Juvenile	Small (Less than 1m)
11	UCAM	Carcharhinus signatus	Night Shark	Y	Brought on Board (Retained/Discarded)	Live - Healthy	Discarded - Live and Healthy (Not Vented)	Female	Small (Less than 1m)
11	UCAM	Caulolatilus microps	Blueline Tilefish	N	Brought on Board (Retained/Discarded)	Live - Stomach and/or Eyes Protruding	Retained	Not Applicable	
11	UCAM	Epinephelus niveatus	Snowy Grouper	N	Brought on Board (Retained/Discarded)	Live - Stomach and/or Eyes Protruding	Retained	Not Applicable	
11	UCAM	Epinephelus niveatus	Snowy Grouper	N	Brought on Board (Retained/Discarded)	Live - Stomach and/or Eyes Protruding	Retained	Not Applicable	
11	UCAM	Epinephelus niveatus	Snowy Grouper	N	Brought on Board (Retained/Discarded)	Live - Stomach and/or Eyes Protruding	Retained	Not Applicable	
11	UCAM	Epinephelus niveatus	Snowy Grouper	N	Brought on Board (Retained/Discarded)	Live - Stomach and/or Eyes Protruding	Retained	Not Applicable	
11	UCAM	Epinephelus niveatus	Snowy Grouper	N	Brought on Board (Retained/Discarded)	Live - Stomach and/or Eyes Protruding	Retained	Not Applicable	
11	UCAM	Epinephelus niveatus	Snowy Grouper	N	Brought on Board (Retained/Discarded)	Live - Stomach and/or Eyes Protruding	Retained	Not Applicable	
11	UCAM	Epinephelus niveatus	Snowy Grouper	N	Brought on Board (Retained/Discarded)	Live - Stomach and/or Eyes Protruding	Retained	Not Applicable	
11	UCAM	Helicolenus dactylopterus	Blackbelly Rosefish	N	Brought on Board (Retained/Discarded)	Live - Healthy	Retained	Not Applicable	
11	UCAM	Helicolenus dactylopterus	Blackbelly Rosefish	N	Brought on Board (Retained/Discarded)	Live - Healthy	Retained	Not Applicable	
11	UCAM	Helicolenus dactylopterus	Blackbelly Rosefish	N	Brought on Board (Retained/Discarded)	Live - Healthy	Retained	Not Applicable	
11	UCAM	Lopholatilus chamaelonticeps	Tilefish, Golden	N	Brought on Board (Retained/Discarded)	Live - Healthy	Retained	Not Applicable	
12	NOCAM	Caulolatilus microps	Blueline Tilefish	N	Brought on Board (Retained/Discarded)	Live - Healthy	Retained	Not Applicable	
12	NOCAM	Caulolatilus microps	Blueline Tilefish	N	Brought on Board (Retained/Discarded)	Live - Stomach and/or Eyes Protruding	Retained	Not Applicable	
12	NOCAM	Caulolatilus microps	Blueline Tilefish	N	Brought on Board (Retained/Discarded)	Live - Healthy	Retained	Not Applicable	
12	NOCAM	Caulolatilus microps	Blueline Tilefish	N	Brought on Board (Retained/Discarded)	Live - Healthy	Retained	Not Applicable	
12	NOCAM	Caulolatilus microps	Blueline Tilefish	N	Brought on Board (Retained/Discarded)	Live - Stomach and/or Eyes Protruding	Retained	Not Applicable	
12	NOCAM	Caulolatilus microps	Blueline Tilefish	N	Brought on Board (Retained/Discarded)	Live - Healthy	Retained	Not Applicable	
12	NOCAM	Caulolatilus microps	Blueline Tilefish	N	Brought on Board (Retained/Discarded)	Live - Healthy	Retained	Not Applicable	
12	NOCAM	Caulolatilus microps	Blueline Tilefish	N	Brought on Board (Retained/Discarded)	Live - Stomach and/or Eyes Protruding	Retained	Not Applicable	
12	NOCAM	Caulolatilus microps	Blueline Tilefish	N	Brought on Board (Retained/Discarded)	Live - Healthy	Retained	Not Applicable	
12	NOCAM	Caulolatilus microps	Blueline Tilefish	N	Brought on Board (Retained/Discarded)	Live - Healthy	Retained	Not Applicable	
12	NOCAM	Epinephelus flavolimbatus	Yellowedge Grouper	N	Brought on Board (Retained/Discarded)	Live - Stomach and/or Eyes Protruding	Retained	Not Applicable	
12	NOCAM	Epinephelus flavolimbatus	Yellowedge Grouper	N	Brought on Board (Retained/Discarded)	Live - Stomach and/or Eyes Protruding	Retained	Not Applicable	
12	NOCAM	Epinephelus flavolimbatus	Yellowedge Grouper	N	Brought on Board (Retained/Discarded)	Live - Stomach and/or Eyes Protruding	Retained	Not Applicable	
12	NOCAM	Epinephelus flavolimbatus	Yellowedge Grouper	N	Brought on Board (Retained/Discarded)	Live - Stomach and/or Eyes Protruding	Retained	Not Applicable	
12	NOCAM	Epinephelus flavolimbatus	Yellowedge Grouper	N	Brought on Board (Retained/Discarded)	Live - Healthy	Retained	Not Applicable	
12	NOCAM	Epinephelus flavolimbatus	Yellowedge Grouper	N	Brought on Board (Retained/Discarded)	Live - Stomach and/or Eyes Protruding	Retained	Not Applicable	
12	NOCAM	Epinephelus flavolimbatus	Yellowedge Grouper	N	Brought on Board (Retained/Discarded)	Live - Stomach and/or Eyes Protruding	Retained	Not Applicable	
12	UCAM	Caulolatilus microps	Blueline Tilefish	N	Brought on Board (Retained/Discarded)	Live - Healthy	Retained	Not Applicable	
12	UCAM	Caulolatilus microps	Blueline Tilefish	N	Brought on Board (Retained/Discarded)	Live - Healthy	Retained	Not Applicable	
12	UCAM	Caulolatilus microps	Blueline Tilefish	N	Brought on Board (Retained/Discarded)	Live - Healthy	Retained	Not Applicable	
12	UCAM	Caulolatilus microps	Blueline Tilefish	N	Brought on Board (Retained/Discarded)	Live - Healthy	Retained	Not Applicable	
12	UCAM	Caulolatilus microps	Blueline Tilefish	N	Brought on Board (Retained/Discarded)	Live - Healthy	Retained	Not Applicable	
12	UCAM	Caulolatilus microps	Blueline Tilefish	N	Brought on Board (Retained/Discarded)	Live - Healthy	Retained	Not Applicable	
12	UCAM	Caulolatilus microps	Blueline Tilefish	N	Brought on Board (Retained/Discarded)	Live - Stomach and/or Eyes Protruding	Retained	Not Applicable	
12	UCAM	Caulolatilus microps	Blueline Tilefish	N	Brought on Board (Retained/Discarded)	Live - Healthy	Retained	Not Applicable	
12	UCAM	Caulolatilus microps	Blueline Tilefish	N	Brought on Board (Retained/Discarded)	Live - Stomach and/or Eyes Protruding	Retained	Not Applicable	
12	UCAM	Caulolatilus microps	Blueline Tilefish	N	Brought on Board (Retained/Discarded)	Live - Stomach and/or Eyes Protruding	Retained	Not Applicable	
12	UCAM	Caulolatilus microps	Blueline Tilefish	N	Brought on Board (Retained/Discarded)	Live - Stomach and/or Eyes Protruding	Retained	Not Applicable	
12	UCAM	Caulolatilus microps	Blueline Tilefish	N	Brought on Board (Retained/Discarded)	Live - Healthy	Retained	Not Applicable	
12	UCAM	Epinephelus flavolimbatus	Yellowedge Grouper	N	Brought on Board (Retained/Discarded)	Live - Stomach and/or Eyes Protruding	Retained	Not Applicable	
12	UCAM	Epinephelus flavolimbatus	Yellowedge Grouper	N	Brought on Board (Retained/Discarded)	Live - Stomach and/or Eyes Protruding	Retained	Not Applicable	
12	UCAM	Epinephelus flavolimbatus	Yellowedge Grouper	N	Brought on Board (Retained/Discarded)	Live - Stomach and/or Eyes Protruding	Retained	Not Applicable	
12	UCAM	Epinephelus flavolimbatus	Yellowedge Grouper	N	Brought on Board (Retained/Discarded)	Live - Stomach and/or Eyes Protruding	Retained	Not Applicable	
12	UCAM	Epinephelus flavolimbatus	Yellowedge Grouper	N	Brought on Board (Retained/Discarded)	Live - Stomach and/or Eyes Protruding	Retained	Not Applicable	
12	UCAM	Epinephelus flavolimbatus	Yellowedge Grouper	N	Brought on Board (Retained/Discarded)	Live - Stomach and/or Eyes Protruding	Retained	Not Applicable	
12	UCAM	Epinephelus flavolimbatus	Yellowedge Grouper	N	Brought on Board (Retained/Discarded)	Live - Stomach and/or Eyes Protruding	Retained	Not Applicable	
13	NOCAM	Anguilliformes, unidentified	Eel, Unidentified	N	Brought on Board (Retained/Discarded)	Live - Healthy	Retained as Bait	Not Applicable	
13	NOCAM	Carcharhinus signatus	Night Shark	Y	Cut-off at Rail (No Entanglement)	Live - Healthy	Discarded - Live and Healthy (Not Vented)	Unknown Maturity and/or Sex	Medium (Between 1m and 2m)
13	NOCAM	Caulolatilus microps	Blueline Tilefish	N	Brought on Board (Retained/Discarded)	Live - Healthy	Retained	Not Applicable	
13	NOCAM	Caulolatilus microps	Blueline Tilefish	N	Brought on Board (Retained/Discarded)	Live - Healthy	Retained	Not Applicable	
13	NOCAM	Caulolatilus microps	Blueline Tilefish	N	Brought on Board (Retained/Discarded)	Live - Healthy	Retained	Not Applicable	
13	NOCAM	Caulolatilus microps	Blueline Tilefish	N	Brought on Board (Retained/Discarded)	Live - Healthy	Retained	Not Applicable	
13	NOCAM	Caulolatilus microps	Blueline Tilefish	N	Brought on Board (Retained/Discarded)	Live - Healthy	Retained	Not Applicable	
13	NOCAM	Caulolatilus microps	Blueline Tilefish	N	Brought on Board (Retained/Discarded)	Live - Healthy	Retained	Not Applicable	
13	NOCAM	Caulolatilus microps	Blueline Tilefish	N	Brought on Board (Retained/Discarded)	Live - Healthy	Retained	Not Applicable	
13	NOCAM	Caulolatilus microps	Blueline Tilefish	N	Brought on Board (Retained/Discarded)	Live - Healthy	Retained	Not Applicable	
13	NOCAM	Caulolatilus microps	Blueline Tilefish	N	Brought on Board (Retained/Discarded)	Live - Healthy	Retained	Not Applicable	
13	NOCAM	Caulolatilus microps	Blueline Tilefish	N	Brought on Board (Retained/Discarded)	Live - Healthy	Retained	Not Applicable	
13	NOCAM	Crab, unidentified	Crab, Unidentified	N	Brought on Board (Retained/Discarded)	Live - Healthy	Discarded - Live and Healthy (Not Vented)	Not Applicable	
13	NOCAM	Epinephelus flavolimbatus	Yellowedge Grouper	N	Brought on Board (Retained/Discarded)	Live - Stomach and/or Eyes Protruding	Retained	Not Applicable	
13	NOCAM	Epinephelus flavolimbatus	Yellowedge Grouper	N	Brought on Board (Retained/Discarded)	Live - Stomach and/or Eyes Protruding	Retained	Not Applicable	
13	NOCAM	Epinephelus flavolimbatus	Yellowedge Grouper	N	Brought on Board (Retained/Discarded)	Live - Stomach and/or Eyes Protruding	Retained	Not Applicable	
13	NOCAM	Epinephelus flavolimbatus	Yellowedge Grouper	N	Brought on Board (Retained/Discarded)	Live - Stomach and/or Eyes Protruding	Retained	Not Applicable	
13	NOCAM	Epinephelus flavolimbatus	Yellowedge Grouper	N	Brought on Board (Retained/Discarded)	Live - Stomach and/or Eyes Protruding	Retained	Not Applicable	
13	NOCAM	Epinephelus flavolimbatus	Yellowedge Grouper	N	Brought on Board (Retained/Discarded)	Live - Stomach and/or Eyes Protruding	Retained	Not Applicable	
13	NOCAM	Epinephelus flavolimbatus	Yellowedge Grouper	N	Brought on Board (Retained/Discarded)	Live - Stomach and/or Eyes Protruding	Retained	Not Applicable	
13	NOCAM	Epinephelus flavolimbatus	Yellowedge Grouper	N	Brought on Board (Retained/Discarded)	Live - Stomach and/or Eyes Protruding	Retained	Not Applicable	
13	NOCAM	Epinephelus niveatus	Snowy Grouper	N	Brought on Board (Retained/Discarded)	Live - Stomach and/or Eyes Protruding	Retained	Not Applicable	
13	NOCAM	Epinephelus niveatus	Snowy Grouper	N	Brought on Board (Retained/Discarded)	Live - Stomach and/or Eyes Protruding	Retained	Not Applicable	
13	NOCAM	Epinephelus niveatus	Snowy Grouper	N	Brought on Board (Retained/Discarded)	Live - Healthy	Retained	Not Applicable	
13	NOCAM	Epinephelus niveatus	Snowy Grouper	N	Brought on Board (Retained/Discarded)	Live - Stomach and/or Eyes Protruding	Retained	Not Applicable	
13	UCAM	Anguilliformes, unidentified	Eel, Unidentified	N	Brought on Board (Retained/Discarded)	Live - Healthy	Retained as Bait	Not Applicable	
13	UCAM	Carcharhinus signatus	Night Shark	Y	Cut-off at Rail (No Entanglement)	Live - Healthy	Discarded - Live and Healthy (Not Vented)	Male (Claspers)	Medium (Between 1m and 2m)
13	UCAM	Caulolatilus microps	Blueline Tilefish	N	Brought on Board (Retained/Discarded)	Live - Healthy	Retained	Not Applicable	
13	UCAM	Caulolatilus microps	Blueline Tilefish	N	Brought on Board (Retained/Discarded)	Live - Healthy	Retained	Not Applicable	
13	UCAM	Caulolatilus microps	Blueline Tilefish	N	Brought on Board (Retained/Discarded)	Live - Healthy	Retained	Not Applicable	
13	UCAM	Caulolatilus microps	Blueline Tilefish	N	Brought on Board (Retained/Discarded)	Live - Healthy	Retained	Not Applicable	
13	UCAM	Caulolatilus microps	Blueline Tilefish	N	Brought on Board (Retained/Discarded)	Live - Stomach and/or Eyes Protruding	Retained	Not Applicable	
13	UCAM	Caulolatilus microps	Blueline Tilefish	N	Brought on Board (Retained/Discarded)	Live - Healthy	Retained	Not Applicable	
13	UCAM	Caulolatilus microps	Blueline Tilefish	N	Brought on Board (Retained/Discarded)	Live - Stomach and/or Eyes Protruding	Retained	Not Applicable	
13	UCAM	Caulolatilus microps	Blueline Tilefish	N	Brought on Board (Retained/Discarded)	Live - Healthy	Retained	Not Applicable	
13	UCAM	Caulolatilus microps	Blueline Tilefish	N	Brought on Board (Retained/Discarded)	Live - Healthy	Retained	Not Applicable	
13	UCAM	Caulolatilus microps	Blueline Tilefish	N	Brought on Board (Retained/Discarded)	Live - Healthy	Retained	Not Applicable	
13	UCAM	Crab, unidentified	Crab, Unidentified	N	Brought on Board (Retained/Discarded)	Live - Healthy	Discarded - Live and Healthy (Not Vented)	Not Applicable	
13	UCAM	Epinephelus flavolimbatus	Yellowedge Grouper	N	Brought on Board (Retained/Discarded)	Live - Stomach and/or Eyes Protruding	Retained	Not Applicable	
13	UCAM	Epinephelus flavolimbatus	Yellowedge Grouper	N	Brought on Board (Retained/Discarded)	Live - Stomach and/or Eyes Protruding	Retained	Not Applicable	
13	UCAM	Epinephelus flavolimbatus	Yellowedge Grouper	N	Brought on Board (Retained/Discarded)	Live - Stomach and/or Eyes Protruding	Retained	Not Applicable	
13	UCAM	Epinephelus flavolimbatus	Yellowedge Grouper	N	Brought on Board (Retained/Discarded)	Live - Stomach and/or Eyes Protruding	Retained	Not Applicable	

13	UCAM	Epinephelus flavolimbatus	Yellowedge Grouper	N	Brought on Board (Retained/Discarded)	Live - Stomach and/or Eyes Protruding	Retained	Not Applicable	
13	UCAM	Epinephelus flavolimbatus	Yellowedge Grouper	N	Brought on Board (Retained/Discarded)	Live - Stomach and/or Eyes Protruding	Retained	Not Applicable	
13	UCAM	Epinephelus flavolimbatus	Yellowedge Grouper	N	Brought on Board (Retained/Discarded)	Live - Stomach and/or Eyes Protruding	Retained	Not Applicable	
13	UCAM	Epinephelus flavolimbatus	Yellowedge Grouper	N	Brought on Board (Retained/Discarded)	Live - Stomach and/or Eyes Protruding	Retained	Not Applicable	
13	UCAM	Epinephelus niveatus	Snowy Grouper	N	Brought on Board (Retained/Discarded)	Live - Stomach and/or Eyes Protruding	Retained	Not Applicable	
13	UCAM	Epinephelus niveatus	Snowy Grouper	N	Brought on Board (Retained/Discarded)	Live - Stomach and/or Eyes Protruding	Retained	Not Applicable	
13	UCAM	Epinephelus niveatus	Snowy Grouper	N	Brought on Board (Retained/Discarded)	Live - Stomach and/or Eyes Protruding	Retained	Not Applicable	
13	UCAM	Epinephelus niveatus	Snowy Grouper	N	Brought on Board (Retained/Discarded)	Live - Stomach and/or Eyes Protruding	Retained	Not Applicable	
14	NOCAM	Carcharhinus signatus	Night Shark	Y	Brought on Board (Retained/Discarded)	Live - Healthy	Discarded - Live and Healthy (Not Vented)	Male (Claspers)	Medium (Between 1m and 2m)
14	NOCAM	Caulolatilus microps	Blueline Tilefish	N	Brought on Board (Retained/Discarded)	Live - Healthy	Retained	Not Applicable	
14	NOCAM	Caulolatilus microps	Blueline Tilefish	N	Brought on Board (Retained/Discarded)	Live - Stomach and/or Eyes Protruding	Retained	Not Applicable	
14	NOCAM	Caulolatilus microps	Blueline Tilefish	N	Brought on Board (Retained/Discarded)	Live - Stomach and/or Eyes Protruding	Retained	Not Applicable	
14	NOCAM	Caulolatilus microps	Blueline Tilefish	N	Brought on Board (Retained/Discarded)	Live - Stomach and/or Eyes Protruding	Retained	Not Applicable	
14	NOCAM	Caulolatilus microps	Blueline Tilefish	N	Brought on Board (Retained/Discarded)	Live - Healthy	Retained	Not Applicable	
14	NOCAM	Caulolatilus microps	Blueline Tilefish	N	Brought on Board (Retained/Discarded)	Live - Healthy	Retained	Not Applicable	
14	NOCAM	Caulolatilus microps	Blueline Tilefish	N	Brought on Board (Retained/Discarded)	Live - Stomach and/or Eyes Protruding	Retained	Not Applicable	
14	NOCAM	Caulolatilus microps	Blueline Tilefish	N	Brought on Board (Retained/Discarded)	Live - Healthy	Retained	Not Applicable	
14	NOCAM	Caulolatilus microps	Blueline Tilefish	N	Brought on Board (Retained/Discarded)	Live - Healthy	Retained	Not Applicable	
14	NOCAM	Caulolatilus microps	Blueline Tilefish	N	Brought on Board (Retained/Discarded)	Live - Stomach and/or Eyes Protruding	Retained	Not Applicable	
14	NOCAM	Caulolatilus microps	Blueline Tilefish	N	Brought on Board (Retained/Discarded)	Live - Healthy	Retained	Not Applicable	
14	NOCAM	Caulolatilus microps	Blueline Tilefish	N	Brought on Board (Retained/Discarded)	Live - Healthy	Retained	Not Applicable	
14	NOCAM	Epinephelus flavolimbatus	Yellowedge Grouper	N	Brought on Board (Retained/Discarded)	Live - Stomach and/or Eyes Protruding	Retained	Not Applicable	
14	NOCAM	Epinephelus flavolimbatus	Yellowedge Grouper	N	Brought on Board (Retained/Discarded)	Live - Stomach and/or Eyes Protruding	Retained	Not Applicable	
14	NOCAM	Epinephelus flavolimbatus	Yellowedge Grouper	N	Brought on Board (Retained/Discarded)	Live - Stomach and/or Eyes Protruding	Retained	Not Applicable	
14	NOCAM	Epinephelus flavolimbatus	Yellowedge Grouper	N	Brought on Board (Retained/Discarded)	Live - Stomach and/or Eyes Protruding	Retained	Not Applicable	
14	NOCAM	Epinephelus flavolimbatus	Yellowedge Grouper	N	Brought on Board (Retained/Discarded)	Live - Stomach and/or Eyes Protruding	Retained	Not Applicable	
14	NOCAM	Epinephelus flavolimbatus	Yellowedge Grouper	N	Brought on Board (Retained/Discarded)	Live - Stomach and/or Eyes Protruding	Retained	Not Applicable	
14	NOCAM	Epinephelus flavolimbatus	Yellowedge Grouper	N	Brought on Board (Retained/Discarded)	Live - Stomach and/or Eyes Protruding	Retained	Not Applicable	
14	NOCAM	Epinephelus flavolimbatus	Yellowedge Grouper	N	Brought on Board (Retained/Discarded)	Live - Stomach and/or Eyes Protruding	Retained	Not Applicable	
14	NOCAM	Epinephelus flavolimbatus	Yellowedge Grouper	N	Not Handled (Dropped Off)	Live - Stomach and/or Eyes Protruding	Discarded - Live and Healthy (Not Vented)	Not Applicable	
14	NOCAM	Epinephelus flavolimbatus	Yellowedge Grouper	N	Brought on Board (Retained/Discarded)	Live - Stomach and/or Eyes Protruding	Retained	Not Applicable	
14	NOCAM	Epinephelus flavolimbatus	Yellowedge Grouper	N	Brought on Board (Retained/Discarded)	Live - Stomach and/or Eyes Protruding	Retained	Not Applicable	
14	NOCAM	Epinephelus flavolimbatus	Yellowedge Grouper	N	Brought on Board (Retained/Discarded)	Live - Stomach and/or Eyes Protruding	Retained	Not Applicable	
14	NOCAM	Epinephelus niveatus	Snowy Grouper	N	Brought on Board (Retained/Discarded)	Live - Stomach and/or Eyes Protruding	Retained	Not Applicable	
14	NOCAM	Mustelus spp.	Dogfish, Smooth (Florida)	Y	Brought on Board (Retained/Discarded)	Live - Healthy	Discarded - Live and Healthy (Not Vented)	Female	Medium (Between 1m and 2m)
14	UCAM	Carcharhinus signatus	Night Shark	Y	Brought on Board (Retained/Discarded)	Live - Healthy	Discarded - Live and Healthy (Not Vented)	Male (Claspers)	Medium (Between 1m and 2m)
14	UCAM	Caulolatilus microps	Blueline Tilefish	N	Brought on Board (Retained/Discarded)	Live - Healthy	Retained	Not Applicable	
14	UCAM	Caulolatilus microps	Blueline Tilefish	N	Brought on Board (Retained/Discarded)	Live - Stomach and/or Eyes Protruding	Retained	Not Applicable	
14	UCAM	Caulolatilus microps	Blueline Tilefish	N	Brought on Board (Retained/Discarded)	Live - Healthy	Retained	Not Applicable	
14	UCAM	Caulolatilus microps	Blueline Tilefish	N	Brought on Board (Retained/Discarded)	Live - Stomach and/or Eyes Protruding	Retained	Not Applicable	
14	UCAM	Caulolatilus microps	Blueline Tilefish	N	Brought on Board (Retained/Discarded)	Live - Stomach and/or Eyes Protruding	Retained	Not Applicable	
14	UCAM	Caulolatilus microps	Blueline Tilefish	N	Brought on Board (Retained/Discarded)	Live - Healthy	Retained	Not Applicable	
14	UCAM	Caulolatilus microps	Blueline Tilefish	N	Brought on Board (Retained/Discarded)	Live - Stomach and/or Eyes Protruding	Retained	Not Applicable	
14	UCAM	Caulolatilus microps	Blueline Tilefish	N	Brought on Board (Retained/Discarded)	Live - Healthy	Retained	Not Applicable	
14	UCAM	Caulolatilus microps	Blueline Tilefish	N	Brought on Board (Retained/Discarded)	Live - Healthy	Retained	Not Applicable	
14	UCAM	Caulolatilus microps	Blueline Tilefish	N	Brought on Board (Retained/Discarded)	Live - Healthy	Retained	Not Applicable	
14	UCAM	Caulolatilus microps	Blueline Tilefish	N	Brought on Board (Retained/Discarded)	Live - Healthy	Retained	Not Applicable	
14	UCAM	Caulolatilus microps	Blueline Tilefish	N	Brought on Board (Retained/Discarded)	Live - Healthy	Retained	Not Applicable	
14	UCAM	Epinephelus flavolimbatus	Yellowedge Grouper	N	Brought on Board (Retained/Discarded)	Live - Stomach and/or Eyes Protruding	Retained	Not Applicable	
14	UCAM	Epinephelus flavolimbatus	Yellowedge Grouper	N	Brought on Board (Retained/Discarded)	Live - Stomach and/or Eyes Protruding	Retained	Not Applicable	
14	UCAM	Epinephelus flavolimbatus	Yellowedge Grouper	N	Brought on Board (Retained/Discarded)	Live - Stomach and/or Eyes Protruding	Retained	Not Applicable	
14	UCAM	Epinephelus flavolimbatus	Yellowedge Grouper	N	Brought on Board (Retained/Discarded)	Live - Stomach and/or Eyes Protruding	Retained	Not Applicable	
14	UCAM	Epinephelus flavolimbatus	Yellowedge Grouper	N	Brought on Board (Retained/Discarded)	Live - Stomach and/or Eyes Protruding	Retained	Not Applicable	
14	UCAM	Epinephelus flavolimbatus	Yellowedge Grouper	N	Brought on Board (Retained/Discarded)	Live - Stomach and/or Eyes Protruding	Retained	Not Applicable	
14	UCAM	Epinephelus flavolimbatus	Yellowedge Grouper	N	Brought on Board (Retained/Discarded)	Live - Stomach and/or Eyes Protruding	Retained	Not Applicable	
14	UCAM	Epinephelus flavolimbatus	Yellowedge Grouper	N	Not Handled (Dropped Off)	Live - Stomach and/or Eyes Protruding	Discarded - Unknown	Not Applicable	
14	UCAM	Epinephelus flavolimbatus	Yellowedge Grouper	N	Brought on Board (Retained/Discarded)	Live - Stomach and/or Eyes Protruding	Retained	Not Applicable	
14	UCAM	Epinephelus flavolimbatus	Yellowedge Grouper	N	Brought on Board (Retained/Discarded)	Live - Stomach and/or Eyes Protruding	Retained	Not Applicable	
14	UCAM	Epinephelus flavolimbatus	Yellowedge Grouper	N	Brought on Board (Retained/Discarded)	Live - Stomach and/or Eyes Protruding	Retained	Not Applicable	
14	UCAM	Epinephelus flavolimbatus	Yellowedge Grouper	N	Brought on Board (Retained/Discarded)	Live - Stomach and/or Eyes Protruding	Retained	Not Applicable	
14	UCAM	Epinephelus niveatus	Snowy Grouper	N	Brought on Board (Retained/Discarded)	Live - Stomach and/or Eyes Protruding	Retained	Not Applicable	
14	UCAM	Mustelus spp.	Dogfish, Smooth (Florida)	Y	Brought on Board (Retained/Discarded)	Live - Healthy	Discarded - Live and Healthy (Not Vented)	Female	Medium (Between 1m and 2m)
15	NOCAM	Anguilliformes, unidentified	Eel, Unidentified	N	Brought on Board (Retained/Discarded)	Live - Healthy	Retained as Bait	Not Applicable	
15	NOCAM	Caulolatilus microps	Blueline Tilefish	N	Brought on Board (Retained/Discarded)	Live - Healthy	Retained	Not Applicable	
15	NOCAM	Caulolatilus microps	Blueline Tilefish	N	Brought on Board (Retained/Discarded)	Live - Stomach and/or Eyes Protruding	Retained	Not Applicable	
15	NOCAM	Caulolatilus microps	Blueline Tilefish	N	Brought on Board (Retained/Discarded)	Live - Healthy	Retained	Not Applicable	
15	NOCAM	Caulolatilus microps	Blueline Tilefish	N	Brought on Board (Retained/Discarded)	Live - Healthy	Retained	Not Applicable	
15	NOCAM	Caulolatilus microps	Blueline Tilefish	N	Brought on Board (Retained/Discarded)	Live - Healthy	Retained	Not Applicable	
15	NOCAM	Caulolatilus microps	Blueline Tilefish	N	Brought on Board (Retained/Discarded)	Live - Healthy	Retained	Not Applicable	
15	NOCAM	Caulolatilus microps	Blueline Tilefish	N	Brought on Board (Retained/Discarded)	Live - Healthy	Retained	Not Applicable	
15	NOCAM	Crab, unidentified	Crab, Unidentified	N	Brought on Board (Retained/Discarded)	Live - Healthy	Discarded - Live and Healthy (Not Vented)	Not Applicable	
15	NOCAM	Epinephelus flavolimbatus	Yellowedge Grouper	N	Brought on Board (Retained/Discarded)	Live - Stomach and/or Eyes Protruding	Retained	Not Applicable	
15	NOCAM	Epinephelus flavolimbatus	Yellowedge Grouper	N	Brought on Board (Retained/Discarded)	Live - Stomach and/or Eyes Protruding	Retained	Not Applicable	
15	NOCAM	Epinephelus flavolimbatus	Yellowedge Grouper	N	Brought on Board (Retained/Discarded)	Live - Stomach and/or Eyes Protruding	Retained	Not Applicable	
15	NOCAM	Epinephelus flavolimbatus	Yellowedge Grouper	N	Brought on Board (Retained/Discarded)	Live - Stomach and/or Eyes Protruding	Retained	Not Applicable	
15	NOCAM	Epinephelus niveatus	Snowy Grouper	N	Brought on Board (Retained/Discarded)	Live - Healthy	Retained	Not Applicable	
15	NOCAM	Thunnus atlanticus	Blackfin Tuna	N	Brought on Board (Retained/Discarded)	Dead on Arrival - Undamaged	Retained as Bait	Not Applicable	
15	UCAM	Anguilliformes, unidentified	Eel, Unidentified	N	Brought on Board (Retained/Discarded)	Live - Healthy	Retained as Bait	Not Applicable	
15	UCAM	Caulolatilus microps	Blueline Tilefish	N	Brought on Board (Retained/Discarded)	Live - Healthy	Retained	Not Applicable	
15	UCAM	Caulolatilus microps	Blueline Tilefish	N	Brought on Board (Retained/Discarded)	Live - Healthy	Retained	Not Applicable	
15	UCAM	Caulolatilus microps	Blueline Tilefish	N	Brought on Board (Retained/Discarded)	Live - Healthy	Retained	Not Applicable	
15	UCAM	Caulolatilus microps	Blueline Tilefish	N	Brought on Board (Retained/Discarded)	Live - Healthy	Retained	Not Applicable	
15	UCAM	Caulolatilus microps	Blueline Tilefish	N	Brought on Board (Retained/Discarded)	Live - Healthy	Retained	Not Applicable	
15	UCAM	Caulolatilus microps	Blueline Tilefish	N	Brought on Board (Retained/Discarded)	Live - Healthy	Retained	Not Applicable	
15	UCAM	Caulolatilus microps	Blueline Tilefish	N	Brought on Board (Retained/Discarded)	Live - Healthy	Retained	Not Applicable	
15	UCAM	Crab, unidentified	Crab, Unidentified	N	Brought on Board (Retained/Discarded)	Live - Healthy	Discarded - Live and Healthy (Not Vented)	Not Applicable	
15	UCAM	Crab, unidentified	Crab, Unidentified	N	Brought on Board (Retained/Discarded)	Live - Healthy	Discarded - Live and Healthy (Not Vented)	Not Applicable	
15	UCAM	Epinephelus flavolimbatus	Yellowedge Grouper	N	Brought on Board (Retained/Discarded)	Live - Stomach and/or Eyes Protruding	Retained	Not Applicable	
15	UCAM	Epinephelus flavolimbatus	Yellowedge Grouper	N	Brought on Board (Retained/Discarded)	Live - Stomach and/or Eyes Protruding	Retained	Not Applicable	
15	UCAM	Epinephelus flavolimbatus	Yellowedge Grouper	N	Brought on Board (Retained/Discarded)	Live - Stomach and/or Eyes Protruding	Retained	Not Applicable	
15	UCAM	Epinephelus flavolimbatus	Yellowedge Grouper	N	Brought on Board (Retained/Discarded)	Live - Stomach and/or Eyes Protruding	Retained	Not Applicable	
15	UCAM	Epinephelus niveatus	Snowy Grouper	N	Brought on Board (Retained/Discarded)	Live - Stomach and/or Eyes Protruding	Retained	Not Applicable	

15	UCAM	Thunnus atlanticus	Blackfin Tuna	N	Brought on Board (Retained/Discarded)	Dead on Arrival - Undamaged	Retained as Bait	Not Applicable	
16	NOCAM	Caulolatilus microps	Blueline Tilefish	N	Brought on Board (Retained/Discarded)	Live - Healthy	Retained	Not Applicable	
16	NOCAM	Caulolatilus microps	Blueline Tilefish	N	Brought on Board (Retained/Discarded)	Live - Healthy	Retained	Not Applicable	
16	NOCAM	Caulolatilus microps	Blueline Tilefish	N	Brought on Board (Retained/Discarded)	Live - Healthy	Retained	Not Applicable	
16	NOCAM	Caulolatilus microps	Blueline Tilefish	N	Brought on Board (Retained/Discarded)	Live - Healthy	Retained	Not Applicable	
16	NOCAM	Caulolatilus microps	Blueline Tilefish	N	Brought on Board (Retained/Discarded)	Live - Stomach and/or Eyes Protruding	Retained	Not Applicable	
16	NOCAM	Caulolatilus microps	Blueline Tilefish	N	Brought on Board (Retained/Discarded)	Live - Stomach and/or Eyes Protruding	Retained	Not Applicable	
16	NOCAM	Caulolatilus microps	Blueline Tilefish	N	Brought on Board (Retained/Discarded)	Live - Healthy	Retained	Not Applicable	
16	NOCAM	Caulolatilus microps	Blueline Tilefish	N	Brought on Board (Retained/Discarded)	Live - Healthy	Retained	Not Applicable	
16	NOCAM	Caulolatilus microps	Blueline Tilefish	N	Brought on Board (Retained/Discarded)	Live - Healthy	Retained	Not Applicable	
16	NOCAM	Caulolatilus microps	Blueline Tilefish	N	Brought on Board (Retained/Discarded)	Live - Healthy	Retained	Not Applicable	
16	NOCAM	Caulolatilus microps	Blueline Tilefish	N	Brought on Board (Retained/Discarded)	Live - Healthy	Retained	Not Applicable	
16	NOCAM	Caulolatilus microps	Blueline Tilefish	N	Brought on Board (Retained/Discarded)	Live - Healthy	Retained	Not Applicable	
16	NOCAM	Caulolatilus microps	Blueline Tilefish	N	Brought on Board (Retained/Discarded)	Live - Healthy	Retained as Bait	Not Applicable	
16	NOCAM	Caulolatilus microps	Blueline Tilefish	N	Brought on Board (Retained/Discarded)	Live - Healthy	Retained	Not Applicable	
16	NOCAM	Caulolatilus microps	Blueline Tilefish	N	Brought on Board (Retained/Discarded)	Live - Stomach and/or Eyes Protruding	Retained	Not Applicable	
16	NOCAM	Caulolatilus microps	Blueline Tilefish	N	Brought on Board (Retained/Discarded)	Live - Healthy	Retained	Not Applicable	
16	NOCAM	Caulolatilus microps	Blueline Tilefish	N	Brought on Board (Retained/Discarded)	Live - Stomach and/or Eyes Protruding	Retained	Not Applicable	
16	NOCAM	Caulolatilus microps	Blueline Tilefish	N	Brought on Board (Retained/Discarded)	Live - Healthy	Retained	Not Applicable	
16	NOCAM	Caulolatilus microps	Blueline Tilefish	N	Brought on Board (Retained/Discarded)	Live - Stomach and/or Eyes Protruding	Retained	Not Applicable	
16	NOCAM	Caulolatilus microps	Blueline Tilefish	N	Brought on Board (Retained/Discarded)	Live - Healthy	Retained	Not Applicable	
16	NOCAM	Caulolatilus microps	Blueline Tilefish	N	Brought on Board (Retained/Discarded)	Live - Healthy	Retained	Not Applicable	
16	NOCAM	Caulolatilus microps	Blueline Tilefish	N	Brought on Board (Retained/Discarded)	Live - Healthy	Retained	Not Applicable	
16	NOCAM	Caulolatilus microps	Blueline Tilefish	N	Brought on Board (Retained/Discarded)	Live - Healthy	Retained	Not Applicable	
16	NOCAM	Caulolatilus microps	Blueline Tilefish	N	Brought on Board (Retained/Discarded)	Live - Healthy	Retained	Not Applicable	
16	NOCAM	Caulolatilus microps	Blueline Tilefish	N	Brought on Board (Retained/Discarded)	Live - Stomach and/or Eyes Protruding	Retained	Not Applicable	
16	NOCAM	Caulolatilus microps	Blueline Tilefish	N	Brought on Board (Retained/Discarded)	Dead on Arrival - Damaged	Retained	Not Applicable	
16	NOCAM	Caulolatilus microps	Blueline Tilefish	N	Brought on Board (Retained/Discarded)	Live - Healthy	Retained	Not Applicable	
16	NOCAM	Caulolatilus microps	Blueline Tilefish	N	Brought on Board (Retained/Discarded)	Live - Healthy	Retained	Not Applicable	
16	NOCAM	Caulolatilus microps	Blueline Tilefish	N	Brought on Board (Retained/Discarded)	Live - Stomach and/or Eyes Protruding	Retained	Not Applicable	
16	NOCAM	Caulolatilus microps	Blueline Tilefish	N	Brought on Board (Retained/Discarded)	Live - Stomach and/or Eyes Protruding	Retained	Not Applicable	
16	NOCAM	Caulolatilus microps	Blueline Tilefish	N	Brought on Board (Retained/Discarded)	Live - Healthy	Retained	Not Applicable	
16	NOCAM	Caulolatilus microps	Blueline Tilefish	N	Brought on Board (Retained/Discarded)	Live - Healthy	Retained	Not Applicable	
16	NOCAM	Caulolatilus microps	Blueline Tilefish	N	Brought on Board (Retained/Discarded)	Live - Stomach and/or Eyes Protruding	Retained	Not Applicable	
16	NOCAM	Caulolatilus microps	Blueline Tilefish	N	Brought on Board (Retained/Discarded)	Live - Healthy	Retained	Not Applicable	
16	NOCAM	Caulolatilus microps	Blueline Tilefish	N	Brought on Board (Retained/Discarded)	Live - Healthy	Retained	Not Applicable	
16	NOCAM	Caulolatilus microps	Blueline Tilefish	N	Brought on Board (Retained/Discarded)	Live - Healthy	Retained	Not Applicable	
16	NOCAM	Caulolatilus microps	Blueline Tilefish	N	Brought on Board (Retained/Discarded)	Live - Healthy	Retained	Not Applicable	
16	NOCAM	Caulolatilus microps	Blueline Tilefish	N	Brought on Board (Retained/Discarded)	Live - Healthy	Retained	Not Applicable	
16	NOCAM	Caulolatilus microps	Blueline Tilefish	N	Brought on Board (Retained/Discarded)	Live - Stomach and/or Eyes Protruding	Retained	Not Applicable	
16	NOCAM	Crab, unidentified	Crab, Unidentified	N	Brought on Board (Retained/Discarded)	Live - Healthy	Discarded - Live and Healthy (Not Vented)	Not Applicable	
16	NOCAM	Epinephelus flavolimbatus	Yellowedge Grouper	N	Brought on Board (Retained/Discarded)	Live - Stomach and/or Eyes Protruding	Retained	Not Applicable	
16	NOCAM	Epinephelus flavolimbatus	Yellowedge Grouper	N	Brought on Board (Retained/Discarded)	Live - Healthy	Retained	Not Applicable	
16	NOCAM	Epinephelus flavolimbatus	Yellowedge Grouper	N	Brought on Board (Retained/Discarded)	Live - Healthy	Retained	Not Applicable	
16	NOCAM	Epinephelus flavolimbatus	Yellowedge Grouper	N	Brought on Board (Retained/Discarded)	Live - Stomach and/or Eyes Protruding	Retained	Not Applicable	
16	NOCAM	Epinephelus flavolimbatus	Yellowedge Grouper	N	Brought on Board (Retained/Discarded)	Live - Stomach and/or Eyes Protruding	Retained	Not Applicable	
16	NOCAM	Epinephelus flavolimbatus	Yellowedge Grouper	N	Brought on Board (Retained/Discarded)	Live - Stomach and/or Eyes Protruding	Retained	Not Applicable	
16	NOCAM	Epinephelus flavolimbatus	Yellowedge Grouper	N	Brought on Board (Retained/Discarded)	Live - Stomach and/or Eyes Protruding	Retained	Not Applicable	
16	NOCAM	Epinephelus flavolimbatus	Yellowedge Grouper	N	Brought on Board (Retained/Discarded)	Live - Stomach and/or Eyes Protruding	Retained	Not Applicable	
16	NOCAM	Epinephelus flavolimbatus	Yellowedge Grouper	N	Brought on Board (Retained/Discarded)	Live - Stomach and/or Eyes Protruding	Retained	Not Applicable	
16	NOCAM	Epinephelus flavolimbatus	Yellowedge Grouper	N	Brought on Board (Retained/Discarded)	Live - Stomach and/or Eyes Protruding	Retained	Not Applicable	
16	NOCAM	Epinephelus flavolimbatus	Yellowedge Grouper	N	Brought on Board (Retained/Discarded)	Live - Stomach and/or Eyes Protruding	Retained	Not Applicable	
16	NOCAM	Epinephelus flavolimbatus	Yellowedge Grouper	N	Brought on Board (Retained/Discarded)	Live - Stomach and/or Eyes Protruding	Retained	Not Applicable	
16	NOCAM	Epinephelus flavolimbatus	Yellowedge Grouper	N	Brought on Board (Retained/Discarded)	Live - Stomach and/or Eyes Protruding	Retained	Not Applicable	
16	NOCAM	Epinephelus flavolimbatus	Yellowedge Grouper	N	Brought on Board (Retained/Discarded)	Live - Stomach and/or Eyes Protruding	Retained	Not Applicable	
16	NOCAM	Epinephelus flavolimbatus	Yellowedge Grouper	N	Brought on Board (Retained/Discarded)	Live - Stomach and/or Eyes Protruding	Retained	Not Applicable	
16	NOCAM	Epinephelus flavolimbatus	Yellowedge Grouper	N	Brought on Board (Retained/Discarded)	Live - Stomach and/or Eyes Protruding	Retained	Not Applicable	
16	NOCAM	Seriola rivoliana	Almaco Jack	N	Brought on Board (Retained/Discarded)	Live - Healthy	Unknown Fate	Not Applicable	
16	UCAM	Caulolatilus microps	Blueline Tilefish	N	Brought on Board (Retained/Discarded)	Live - Healthy	Retained	Not Applicable	
16	UCAM	Caulolatilus microps	Blueline Tilefish	N	Brought on Board (Retained/Discarded)	Live - Healthy	Retained	Not Applicable	
16	UCAM	Caulolatilus microps	Blueline Tilefish	N	Brought on Board (Retained/Discarded)	Dead on Arrival - Damaged	Retained	Not Applicable	
16	UCAM	Caulolatilus microps	Blueline Tilefish	N	Brought on Board (Retained/Discarded)	Live - Healthy	Retained	Not Applicable	
16	UCAM	Caulolatilus microps	Blueline Tilefish	N	Brought on Board (Retained/Discarded)	Live - Stomach and/or Eyes Protruding	Retained	Not Applicable	
16	UCAM	Caulolatilus microps	Blueline Tilefish	N	Brought on Board (Retained/Discarded)	Live - Stomach and/or Eyes Protruding	Retained	Not Applicable	
16	UCAM	Caulolatilus microps	Blueline Tilefish	N	Brought on Board (Retained/Discarded)	Live - Healthy	Retained	Not Applicable	
16	UCAM	Caulolatilus microps	Blueline Tilefish	N	Brought on Board (Retained/Discarded)	Live - Healthy	Retained	Not Applicable	
16	UCAM	Caulolatilus microps	Blueline Tilefish	N	Brought on Board (Retained/Discarded)	Live - Healthy	Retained	Not Applicable	
16	UCAM	Caulolatilus microps	Blueline Tilefish	N	Brought on Board (Retained/Discarded)	Live - Stomach and/or Eyes Protruding	Retained	Not Applicable	
16	UCAM	Caulolatilus microps	Blueline Tilefish	N	Brought on Board (Retained/Discarded)	Live - Healthy	Retained	Not Applicable	
16	UCAM	Caulolatilus microps	Blueline Tilefish	N	Brought on Board (Retained/Discarded)	Live - Healthy	Retained	Not Applicable	
16	UCAM	Caulolatilus microps	Blueline Tilefish	N	Brought on Board (Retained/Discarded)	Live - Healthy	Retained	Not Applicable	
16	UCAM	Caulolatilus microps	Blueline Tilefish	N	Brought on Board (Retained/Discarded)	Live - Healthy	Retained	Not Applicable	
16	UCAM	Caulolatilus microps	Blueline Tilefish	N	Brought on Board (Retained/Discarded)	Live - Healthy	Retained	Not Applicable	
16	UCAM	Caulolatilus microps	Blueline Tilefish	N	Brought on Board (Retained/Discarded)	Live - Healthy	Retained	Not Applicable	
16	UCAM	Caulolatilus microps	Blueline Tilefish	N	Brought on Board (Retained/Discarded)	Live - Healthy	Retained	Not Applicable	
16	UCAM	Caulolatilus microps	Blueline Tilefish	N	Brought on Board (Retained/Discarded)	Live - Healthy	Retained	Not Applicable	
16	UCAM	Caulolatilus microps	Blueline Tilefish	N	Brought on Board (Retained/Discarded)	Live - Healthy	Retained	Not Applicable	
16	UCAM	Caulolatilus microps	Blueline Tilefish	N	Brought on Board (Retained/Discarded)	Live - Healthy	Retained	Not Applicable	
16	UCAM	Caulolatilus microps	Blueline Tilefish	N	Brought on Board (Retained/Discarded)	Live - Stomach and/or Eyes Protruding	Retained	Not Applicable	
16	UCAM	Caulolatilus microps	Blueline Tilefish	N	Brought on Board (Retained/Discarded)	Live - Stomach and/or Eyes Protruding	Retained	Not Applicable	
16	UCAM	Caulolatilus microps	Blueline Tilefish	N	Brought on Board (Retained/Discarded)	Live - Healthy	Retained	Not Applicable	
16	UCAM	Caulolatilus microps	Blueline Tilefish	N	Brought on Board (Retained/Discarded)	Live - Stomach and/or Eyes Protruding	Retained	Not Applicable	
16	UCAM	Caulolatilus microps	Blueline Tilefish	N	Brought on Board (Retained/Discarded)	Live - Stomach and/or Eyes Protruding	Retained	Not Applicable	
16	UCAM	Caulolatilus microps	Blueline Tilefish	N	Brought on Board (Retained/Discarded)	Live - Stomach and/or Eyes Protruding	Retained	Not Applicable	
16	UCAM	Caulolatilus microps	Blueline Tilefish	N	Brought on Board (Retained/Discarded)	Live - Stomach and/or Eyes Protruding	Retained	Not Applicable	
16	UCAM	Caulolatilus microps	Blueline Tilefish	N	Brought on Board (Retained/Discarded)	Live - Healthy	Retained	Not Applicable	
16	UCAM	Caulolatilus microps	Blueline Tilefish	N	Brought on Board (Retained/Discarded)	Live - Healthy	Retained	Not Applicable	
16	UCAM	Caulolatilus microps	Blueline Tilefish	N	Brought on Board (Retained/Discarded)	Live - Healthy	Retained	Not Applicable	
16	UCAM	Caulolatilus microps	Blueline Tilefish	N	Brought on Board (Retained/Discarded)	Live - Healthy	Retained	Not Applicable	
16	UCAM	Caulolatilus microps	Blueline Tilefish	N	Brought on Board (Retained/Discarded)	Live - Healthy	Retained	Not Applicable	
16	UCAM	Caulolatilus microps	Blueline Tilefish	N	Brought on Board (Retained/Discarded)	Live - Stomach and/or Eyes Protruding	Retained	Not Applicable	
16	UCAM	Caulolatilus microps	Blueline Tilefish	N	Brought on Board (Retained/Discarded)	Live - Stomach and/or Eyes Protruding	Retained	Not Applicable	

16	UCAM	Caulolatilus microps	Blueline Tilefish	N	Brought on Board (Retained/Discarded)	Live - Stomach and/or Eyes Protruding	Retained	Not Applicable	
16	UCAM	Caulolatilus microps	Blueline Tilefish	N	Brought on Board (Retained/Discarded)	Live - Healthy	Retained	Not Applicable	
16	UCAM	Caulolatilus microps	Blueline Tilefish	N	Brought on Board (Retained/Discarded)	Live - Healthy	Retained	Not Applicable	
16	UCAM	Caulolatilus microps	Blueline Tilefish	N	Brought on Board (Retained/Discarded)	Live - Stomach and/or Eyes Protruding	Retained	Not Applicable	
16	UCAM	Caulolatilus microps	Blueline Tilefish	N	Brought on Board (Retained/Discarded)	Live - Healthy	Retained	Not Applicable	
16	UCAM	Caulolatilus microps	Blueline Tilefish	N	Brought on Board (Retained/Discarded)	Live - Healthy	Retained	Not Applicable	
16	UCAM	Caulolatilus microps	Blueline Tilefish	N	Brought on Board (Retained/Discarded)	Live - Healthy	Retained	Not Applicable	
16	UCAM	Crab, unidentified	Crab, Unidentified	N	Brought on Board (Retained/Discarded)	Live - Healthy	Discarded - Live and Healthy (Not Vented)	Not Applicable	
16	UCAM	Epinephelus flavolimbatus	Yellowedge Grouper	N	Brought on Board (Retained/Discarded)	Live - Stomach and/or Eyes Protruding	Retained	Not Applicable	
16	UCAM	Epinephelus flavolimbatus	Yellowedge Grouper	N	Brought on Board (Retained/Discarded)	Live - Stomach and/or Eyes Protruding	Retained	Not Applicable	
16	UCAM	Epinephelus flavolimbatus	Yellowedge Grouper	N	Brought on Board (Retained/Discarded)	Live - Stomach and/or Eyes Protruding	Retained	Not Applicable	
16	UCAM	Epinephelus flavolimbatus	Yellowedge Grouper	N	Brought on Board (Retained/Discarded)	Live - Stomach and/or Eyes Protruding	Retained	Not Applicable	
16	UCAM	Epinephelus flavolimbatus	Yellowedge Grouper	N	Brought on Board (Retained/Discarded)	Live - Stomach and/or Eyes Protruding	Retained	Not Applicable	
16	UCAM	Epinephelus flavolimbatus	Yellowedge Grouper	N	Brought on Board (Retained/Discarded)	Live - Stomach and/or Eyes Protruding	Retained	Not Applicable	
16	UCAM	Epinephelus flavolimbatus	Yellowedge Grouper	N	Brought on Board (Retained/Discarded)	Live - Stomach and/or Eyes Protruding	Retained	Not Applicable	
16	UCAM	Epinephelus flavolimbatus	Yellowedge Grouper	N	Brought on Board (Retained/Discarded)	Live - Stomach and/or Eyes Protruding	Retained	Not Applicable	
16	UCAM	Epinephelus flavolimbatus	Yellowedge Grouper	N	Brought on Board (Retained/Discarded)	Live - Stomach and/or Eyes Protruding	Retained	Not Applicable	
16	UCAM	Epinephelus flavolimbatus	Yellowedge Grouper	N	Brought on Board (Retained/Discarded)	Live - Stomach and/or Eyes Protruding	Retained	Not Applicable	
16	UCAM	Epinephelus flavolimbatus	Yellowedge Grouper	N	Brought on Board (Retained/Discarded)	Live - Stomach and/or Eyes Protruding	Retained	Not Applicable	
16	UCAM	Epinephelus flavolimbatus	Yellowedge Grouper	N	Brought on Board (Retained/Discarded)	Live - Stomach and/or Eyes Protruding	Retained	Not Applicable	
16	UCAM	Epinephelus flavolimbatus	Yellowedge Grouper	N	Brought on Board (Retained/Discarded)	Live - Stomach and/or Eyes Protruding	Retained	Not Applicable	
16	UCAM	Epinephelus flavolimbatus	Yellowedge Grouper	N	Brought on Board (Retained/Discarded)	Live - Stomach and/or Eyes Protruding	Retained	Not Applicable	
16	UCAM	Epinephelus flavolimbatus	Yellowedge Grouper	N	Brought on Board (Retained/Discarded)	Live - Stomach and/or Eyes Protruding	Retained	Not Applicable	
16	UCAM	Epinephelus flavolimbatus	Yellowedge Grouper	N	Brought on Board (Retained/Discarded)	Live - Stomach and/or Eyes Protruding	Retained	Not Applicable	
16	UCAM	Epinephelus flavolimbatus	Yellowedge Grouper	N	Brought on Board (Retained/Discarded)	Live - Stomach and/or Eyes Protruding	Retained	Not Applicable	
16	UCAM	Seriola rivoliana	Almaco Jack	N	Brought on Board (Retained/Discarded)	Live - Healthy	Unknown Fate	Not Applicable	
17	NOCAM	Caulolatilus microps	Blueline Tilefish	N	Brought on Board (Retained/Discarded)	Live - Healthy	Retained	Not Applicable	
17	NOCAM	Caulolatilus microps	Blueline Tilefish	N	Brought on Board (Retained/Discarded)	Live - Healthy	Retained	Not Applicable	
17	NOCAM	Caulolatilus microps	Blueline Tilefish	N	Brought on Board (Retained/Discarded)	Live - Stomach and/or Eyes Protruding	Retained	Not Applicable	
17	NOCAM	Caulolatilus microps	Blueline Tilefish	N	Brought on Board (Retained/Discarded)	Live - Healthy	Retained	Not Applicable	
17	NOCAM	Caulolatilus microps	Blueline Tilefish	N	Brought on Board (Retained/Discarded)	Live - Stomach and/or Eyes Protruding	Retained	Not Applicable	
17	NOCAM	Caulolatilus microps	Blueline Tilefish	N	Brought on Board (Retained/Discarded)	Live - Stomach and/or Eyes Protruding	Retained	Not Applicable	
17	NOCAM	Caulolatilus microps	Blueline Tilefish	N	Brought on Board (Retained/Discarded)	Live - Stomach and/or Eyes Protruding	Retained	Not Applicable	
17	NOCAM	Caulolatilus microps	Blueline Tilefish	N	Brought on Board (Retained/Discarded)	Live - Healthy	Retained	Not Applicable	
17	NOCAM	Caulolatilus microps	Blueline Tilefish	N	Brought on Board (Retained/Discarded)	Live - Healthy	Retained	Not Applicable	
17	NOCAM	Caulolatilus microps	Blueline Tilefish	N	Brought on Board (Retained/Discarded)	Live - Healthy	Retained	Not Applicable	
17	NOCAM	Caulolatilus microps	Blueline Tilefish	N	Brought on Board (Retained/Discarded)	Live - Healthy	Retained	Not Applicable	
17	NOCAM	Caulolatilus microps	Blueline Tilefish	N	Brought on Board (Retained/Discarded)	Live - Healthy	Retained	Not Applicable	
17	NOCAM	Caulolatilus microps	Blueline Tilefish	N	Brought on Board (Retained/Discarded)	Live - Healthy	Retained	Not Applicable	
17	NOCAM	Caulolatilus microps	Blueline Tilefish	N	Brought on Board (Retained/Discarded)	Live - Healthy	Retained	Not Applicable	
17	NOCAM	Caulolatilus microps	Blueline Tilefish	N	Brought on Board (Retained/Discarded)	Live - Stomach and/or Eyes Protruding	Retained	Not Applicable	
17	NOCAM	Epinephelus flavolimbatus	Yellowedge Grouper	N	Brought on Board (Retained/Discarded)	Live - Stomach and/or Eyes Protruding	Retained	Not Applicable	
17	NOCAM	Epinephelus flavolimbatus	Yellowedge Grouper	N	Brought on Board (Retained/Discarded)	Live - Healthy	Retained	Not Applicable	
17	NOCAM	Epinephelus flavolimbatus	Yellowedge Grouper	N	Brought on Board (Retained/Discarded)	Live - Stomach and/or Eyes Protruding	Retained	Not Applicable	
17	NOCAM	Epinephelus flavolimbatus	Yellowedge Grouper	N	Brought on Board (Retained/Discarded)	Live - Stomach and/or Eyes Protruding	Retained	Not Applicable	
17	NOCAM	Epinephelus flavolimbatus	Yellowedge Grouper	N	Brought on Board (Retained/Discarded)	Live - Stomach and/or Eyes Protruding	Retained	Not Applicable	
17	NOCAM	Epinephelus flavolimbatus	Yellowedge Grouper	N	Brought on Board (Retained/Discarded)	Dead on Arrival - Damaged	Discarded - Dead	Not Applicable	
17	NOCAM	Epinephelus flavolimbatus	Yellowedge Grouper	N	Brought on Board (Retained/Discarded)	Live - Stomach and/or Eyes Protruding	Retained	Not Applicable	
17	NOCAM	Epinephelus flavolimbatus	Yellowedge Grouper	N	Brought on Board (Retained/Discarded)	Live - Healthy	Retained	Not Applicable	
17	NOCAM	Epinephelus flavolimbatus	Yellowedge Grouper	N	Brought on Board (Retained/Discarded)	Live - Stomach and/or Eyes Protruding	Retained	Not Applicable	
17	NOCAM	Epinephelus flavolimbatus	Yellowedge Grouper	N	Brought on Board (Retained/Discarded)	Live - Stomach and/or Eyes Protruding	Retained	Not Applicable	
17	NOCAM	Epinephelus flavolimbatus	Yellowedge Grouper	N	Brought on Board (Retained/Discarded)	Live - Stomach and/or Eyes Protruding	Retained	Not Applicable	
17	NOCAM	Epinephelus flavolimbatus	Yellowedge Grouper	N	Brought on Board (Retained/Discarded)	Live - Stomach and/or Eyes Protruding	Retained	Not Applicable	
17	NOCAM	Epinephelus flavolimbatus	Yellowedge Grouper	N	Brought on Board (Retained/Discarded)	Live - Stomach and/or Eyes Protruding	Retained	Not Applicable	
17	NOCAM	Epinephelus flavolimbatus	Yellowedge Grouper	N	Brought on Board (Retained/Discarded)	Live - Healthy	Retained	Not Applicable	
17	NOCAM	Etelis oculatus	Queen Snapper	N	Brought on Board (Retained/Discarded)	Live - Healthy	Retained	Not Applicable	
17	NOCAM	Mustelus spp.	Dogfish, Smooth (Florida)	Y	Brought on Board (Retained/Discarded)	Live - Healthy	Discarded - Live and Healthy (Not Vented)	Female	Medium (Between 1m and 2m)
17	UCAM	Caulolatilus microps	Blueline Tilefish	N	Brought on Board (Retained/Discarded)	Live - Healthy	Retained	Not Applicable	
17	UCAM	Caulolatilus microps	Blueline Tilefish	N	Brought on Board (Retained/Discarded)	Live - Stomach and/or Eyes Protruding	Retained	Not Applicable	
17	UCAM	Caulolatilus microps	Blueline Tilefish	N	Brought on Board (Retained/Discarded)	Live - Stomach and/or Eyes Protruding	Retained	Not Applicable	
17	UCAM	Caulolatilus microps	Blueline Tilefish	N	Brought on Board (Retained/Discarded)	Live - Healthy	Retained	Not Applicable	
17	UCAM	Caulolatilus microps	Blueline Tilefish	N	Brought on Board (Retained/Discarded)	Live - Stomach and/or Eyes Protruding	Retained	Not Applicable	
17	UCAM	Caulolatilus microps	Blueline Tilefish	N	Brought on Board (Retained/Discarded)	Live - Healthy	Retained	Not Applicable	
17	UCAM	Caulolatilus microps	Blueline Tilefish	N	Brought on Board (Retained/Discarded)	Live - Stomach and/or Eyes Protruding	Retained	Not Applicable	
17	UCAM	Caulolatilus microps	Blueline Tilefish	N	Brought on Board (Retained/Discarded)	Live - Stomach and/or Eyes Protruding	Retained	Not Applicable	
17	UCAM	Caulolatilus microps	Blueline Tilefish	N	Brought on Board (Retained/Discarded)	Live - Stomach and/or Eyes Protruding	Retained	Not Applicable	
17	UCAM	Caulolatilus microps	Blueline Tilefish	N	Brought on Board (Retained/Discarded)	Live - Stomach and/or Eyes Protruding	Retained	Not Applicable	
17	UCAM	Caulolatilus microps	Blueline Tilefish	N	Brought on Board (Retained/Discarded)	Live - Healthy	Retained	Not Applicable	
17	UCAM	Caulolatilus microps	Blueline Tilefish	N	Brought on Board (Retained/Discarded)	Live - Stomach and/or Eyes Protruding	Retained	Not Applicable	
17	UCAM	Caulolatilus microps	Blueline Tilefish	N	Brought on Board (Retained/Discarded)	Live - Stomach and/or Eyes Protruding	Retained	Not Applicable	
17	UCAM	Caulolatilus microps	Blueline Tilefish	N	Brought on Board (Retained/Discarded)	Live - Healthy	Retained	Not Applicable	
17	UCAM	Caulolatilus microps	Blueline Tilefish	N	Brought on Board (Retained/Discarded)	Live - Healthy	Retained	Not Applicable	
17	UCAM	Epinephelus flavolimbatus	Yellowedge Grouper	N	Brought on Board (Retained/Discarded)	Live - Healthy	Retained	Not Applicable	
17	UCAM	Epinephelus flavolimbatus	Yellowedge Grouper	N	Brought on Board (Retained/Discarded)	Live - Stomach and/or Eyes Protruding	Retained	Not Applicable	
17	UCAM	Epinephelus flavolimbatus	Yellowedge Grouper	N	Brought on Board (Retained/Discarded)	Live - Stomach and/or Eyes Protruding	Retained	Not Applicable	
17	UCAM	Epinephelus flavolimbatus	Yellowedge Grouper	N	Brought on Board (Retained/Discarded)	Live - Healthy	Retained	Not Applicable	
17	UCAM	Epinephelus flavolimbatus	Yellowedge Grouper	N	Brought on Board (Retained/Discarded)	Live - Stomach and/or Eyes Protruding	Retained	Not Applicable	
17	UCAM	Epinephelus flavolimbatus	Yellowedge Grouper	N	Brought on Board (Retained/Discarded)	Live - Stomach and/or Eyes Protruding	Retained	Not Applicable	
17	UCAM	Epinephelus flavolimbatus	Yellowedge Grouper	N	Brought on Board (Retained/Discarded)	Live - Stomach and/or Eyes Protruding	Retained	Not Applicable	
17	UCAM	Epinephelus flavolimbatus	Yellowedge Grouper	N	Brought on Board (Retained/Discarded)	Live - Stomach and/or Eyes Protruding	Retained	Not Applicable	
17	UCAM	Epinephelus flavolimbatus	Yellowedge Grouper	N	Brought on Board (Retained/Discarded)	Live - Stomach and/or Eyes Protruding	Retained	Not Applicable	
17	UCAM	Epinephelus flavolimbatus	Yellowedge Grouper	N	Brought on Board (Retained/Discarded)	Live - Stomach and/or Eyes Protruding	Retained	Not Applicable	
17	UCAM	Epinephelus flavolimbatus	Yellowedge Grouper	N	Brought on Board (Retained/Discarded)	Live - Stomach and/or Eyes Protruding	Retained	Not Applicable	
17	UCAM	Epinephelus flavolimbatus	Yellowedge Grouper	N	Brought on Board (Retained/Discarded)	Live - Stomach and/or Eyes Protruding	Retained	Not Applicable	
17	UCAM	Epinephelus flavolimbatus	Yellowedge Grouper	N	Brought on Board (Retained/Discarded)	Dead on Arrival - Damaged	Discarded - Dead	Not Applicable	
17	UCAM	Epinephelus flavolimbatus	Yellowedge Grouper	N	Brought on Board (Retained/Discarded)	Live - Stomach and/or Eyes Protruding	Retained	Not Applicable	
17	UCAM	Etelis oculatus	Queen Snapper	N	Brought on Board (Retained/Discarded)	Live - Healthy	Retained	Not Applicable	
17	UCAM	Mustelus spp.	Dogfish, Smooth (Florida)	Y	Brought on Board (Retained/Discarded)	Live - Healthy	Discarded - Live and Healthy (Not Vented)	Female	Medium (Between 1m and 2m)
18	NOCAM	Caulolatilus microps	Blueline Tilefish	N	Brought on Board (Retained/Discarded)	Live - Stomach and/or Eyes Protruding	Retained	Not Applicable	
18	NOCAM	Caulolatilus microps	Blueline Tilefish	N	Brought on Board (Retained/Discarded)	Live - Healthy	Retained	Not Applicable	
18	NOCAM	Caulolatilus microps	Blueline Tilefish	N	Brought on Board (Retained/Discarded)	Live - Stomach and/or Eyes Protruding	Retained	Not Applicable	
18	NOCAM	Caulolatilus microps	Blueline Tilefish	N	Brought on Board (Retained/Discarded)	Live - Stomach and/or Eyes Protruding	Retained	Not Applicable	

18	NOCAM	Caulolatilus microps	Blueline Tilefish	N	Brought on Board (Retained/Discarded)	Live - Stomach and/or Eyes Protruding	Retained	Not Applicable	
18	NOCAM	Caulolatilus microps	Blueline Tilefish	N	Brought on Board (Retained/Discarded)	Live - Stomach and/or Eyes Protruding	Retained	Not Applicable	
18	NOCAM	Caulolatilus microps	Blueline Tilefish	N	Brought on Board (Retained/Discarded)	Live - Healthy	Retained	Not Applicable	
18	NOCAM	Caulolatilus microps	Blueline Tilefish	N	Brought on Board (Retained/Discarded)	Live - Healthy	Retained	Not Applicable	
18	NOCAM	Caulolatilus microps	Blueline Tilefish	N	Brought on Board (Retained/Discarded)	Live - Healthy	Retained	Not Applicable	
18	NOCAM	Caulolatilus microps	Blueline Tilefish	N	Brought on Board (Retained/Discarded)	Live - Stomach and/or Eyes Protruding	Retained	Not Applicable	
18	NOCAM	Coryphaena hippurus	Dolphin Fish (Mahi mahi)	N	Brought on Board (Retained/Discarded)	Dead on Arrival - Damaged	Discarded - Dead	Not Applicable	
18	NOCAM	Epinephelus flavolimbatus	Yellowedge Grouper	N	Brought on Board (Retained/Discarded)	Live - Stomach and/or Eyes Protruding	Retained	Not Applicable	
18	NOCAM	Epinephelus flavolimbatus	Yellowedge Grouper	N	Brought on Board (Retained/Discarded)	Live - Stomach and/or Eyes Protruding	Retained	Not Applicable	
18	NOCAM	Epinephelus flavolimbatus	Yellowedge Grouper	N	Brought on Board (Retained/Discarded)	Live - Stomach and/or Eyes Protruding	Retained	Not Applicable	
18	NOCAM	Epinephelus flavolimbatus	Yellowedge Grouper	N	Brought on Board (Retained/Discarded)	Live - Stomach and/or Eyes Protruding	Retained	Not Applicable	
18	NOCAM	Epinephelus flavolimbatus	Yellowedge Grouper	N	Brought on Board (Retained/Discarded)	Live - Stomach and/or Eyes Protruding	Retained	Not Applicable	
18	NOCAM	Epinephelus flavolimbatus	Yellowedge Grouper	N	Brought on Board (Retained/Discarded)	Live - Stomach and/or Eyes Protruding	Retained	Not Applicable	
18	NOCAM	Sphyraena barracuda	Barracuda (Great)	N	Brought on Board (Retained/Discarded)	Dead on Arrival - Undamaged	Unknown Fate	Not Applicable	
18	UCAM	Caulolatilus microps	Blueline Tilefish	N	Brought on Board (Retained/Discarded)	Live - Healthy	Retained	Not Applicable	
18	UCAM	Caulolatilus microps	Blueline Tilefish	N	Brought on Board (Retained/Discarded)	Live - Stomach and/or Eyes Protruding	Retained	Not Applicable	
18	UCAM	Caulolatilus microps	Blueline Tilefish	N	Brought on Board (Retained/Discarded)	Live - Healthy	Retained	Not Applicable	
18	UCAM	Caulolatilus microps	Blueline Tilefish	N	Brought on Board (Retained/Discarded)	Live - Healthy	Retained	Not Applicable	
18	UCAM	Caulolatilus microps	Blueline Tilefish	N	Brought on Board (Retained/Discarded)	Live - Stomach and/or Eyes Protruding	Retained	Not Applicable	
18	UCAM	Caulolatilus microps	Blueline Tilefish	N	Brought on Board (Retained/Discarded)	Live - Healthy	Retained	Not Applicable	
18	UCAM	Caulolatilus microps	Blueline Tilefish	N	Brought on Board (Retained/Discarded)	Live - Stomach and/or Eyes Protruding	Retained	Not Applicable	
18	UCAM	Caulolatilus microps	Blueline Tilefish	N	Brought on Board (Retained/Discarded)	Live - Stomach and/or Eyes Protruding	Retained	Not Applicable	
18	UCAM	Caulolatilus microps	Blueline Tilefish	N	Brought on Board (Retained/Discarded)	Live - Stomach and/or Eyes Protruding	Retained	Not Applicable	
18	UCAM	Caulolatilus microps	Blueline Tilefish	N	Brought on Board (Retained/Discarded)	Live - Healthy	Retained	Not Applicable	
18	UCAM	Coryphaena hippurus	Dolphin Fish (Mahi mahi)	N	Brought on Board (Retained/Discarded)	Dead on Arrival - Damaged	Discarded - Dead	Not Applicable	
18	UCAM	Epinephelus flavolimbatus	Yellowedge Grouper	N	Brought on Board (Retained/Discarded)	Live - Stomach and/or Eyes Protruding	Retained	Not Applicable	
18	UCAM	Epinephelus flavolimbatus	Yellowedge Grouper	N	Brought on Board (Retained/Discarded)	Live - Stomach and/or Eyes Protruding	Retained	Not Applicable	
18	UCAM	Epinephelus flavolimbatus	Yellowedge Grouper	N	Brought on Board (Retained/Discarded)	Live - Stomach and/or Eyes Protruding	Retained	Not Applicable	
18	UCAM	Epinephelus flavolimbatus	Yellowedge Grouper	N	Brought on Board (Retained/Discarded)	Live - Stomach and/or Eyes Protruding	Retained	Not Applicable	
18	UCAM	Epinephelus flavolimbatus	Yellowedge Grouper	N	Brought on Board (Retained/Discarded)	Live - Stomach and/or Eyes Protruding	Retained	Not Applicable	
18	UCAM	Epinephelus flavolimbatus	Yellowedge Grouper	N	Brought on Board (Retained/Discarded)	Live - Stomach and/or Eyes Protruding	Retained	Not Applicable	
18	UCAM	Sphyraena barracuda	Barracuda (Great)	N	Brought on Board (Retained/Discarded)	Dead on Arrival - Undamaged	Unknown Fate	Not Applicable	
19	NOCAM	Calamus bajonada	Jolthead Porgy	N	Brought on Board (Retained/Discarded)	Live - Healthy	Retained	Not Applicable	
19	NOCAM	Epinephelus morio	Red Grouper	N	Brought on Board (Retained/Discarded)	Live - Healthy	Discarded - Live and Healthy (Vented)	Not Applicable	
19	NOCAM	Epinephelus morio	Red Grouper	N	Brought on Board (Retained/Discarded)	Live - Healthy	Discarded - Live and Healthy (Vented)	Not Applicable	
19	NOCAM	Epinephelus morio	Red Grouper	N	Brought on Board (Retained/Discarded)	Live - Healthy	Discarded - Live and Healthy (Vented)	Not Applicable	
19	NOCAM	Epinephelus morio	Red Grouper	N	Brought on Board (Retained/Discarded)	Live - Healthy	Retained	Not Applicable	
19	NOCAM	Epinephelus morio	Red Grouper	N	Brought on Board (Retained/Discarded)	Live - Healthy	Discarded - Live and Healthy (Vented)	Not Applicable	
19	NOCAM	Epinephelus morio	Red Grouper	N	Brought on Board (Retained/Discarded)	Live - Healthy	Retained	Not Applicable	
19	NOCAM	Epinephelus morio	Red Grouper	N	Brought on Board (Retained/Discarded)	Live - Healthy	Retained	Not Applicable	
19	NOCAM	Epinephelus morio	Red Grouper	N	Brought on Board (Retained/Discarded)	Live - Healthy	Discarded - Live and Healthy (Vented)	Not Applicable	
19	NOCAM	Epinephelus morio	Red Grouper	N	Brought on Board (Retained/Discarded)	Live - Healthy	Discarded - Live and Healthy (Vented)	Not Applicable	
19	NOCAM	Epinephelus morio	Red Grouper	N	Brought on Board (Retained/Discarded)	Live - Stomach and/or Eyes Protruding	Retained	Not Applicable	
19	NOCAM	Epinephelus morio	Red Grouper	N	Brought on Board (Retained/Discarded)	Live - Healthy	Discarded - Live and Healthy (Vented)	Not Applicable	
19	NOCAM	Epinephelus morio	Red Grouper	N	Brought on Board (Retained/Discarded)	Live - Healthy	Retained	Not Applicable	
19	NOCAM	Epinephelus morio	Red Grouper	N	Brought on Board (Retained/Discarded)	Live - Healthy	Retained	Not Applicable	
19	NOCAM	Epinephelus morio	Red Grouper	N	Brought on Board (Retained/Discarded)	Live - Healthy	Retained	Not Applicable	
19	NOCAM	Epinephelus morio	Red Grouper	N	Brought on Board (Retained/Discarded)	Live - Healthy	Retained	Not Applicable	
19	NOCAM	Epinephelus morio	Red Grouper	N	Brought on Board (Retained/Discarded)	Live - Healthy	Discarded - Live and Healthy (Vented)	Not Applicable	
19	NOCAM	Epinephelus morio	Red Grouper	N	Brought on Board (Retained/Discarded)	Live - Healthy	Discarded - Live and Healthy (Vented)	Not Applicable	
19	NOCAM	Epinephelus morio	Red Grouper	N	Brought on Board (Retained/Discarded)	Live - Healthy	Retained	Not Applicable	
19	NOCAM	Epinephelus morio	Red Grouper	N	Brought on Board (Retained/Discarded)	Live - Healthy	Retained	Not Applicable	
19	NOCAM	Epinephelus morio	Red Grouper	N	Brought on Board (Retained/Discarded)	Dead on Arrival - Damaged	Discarded - Dead	Not Applicable	
19	NOCAM	Epinephelus morio	Red Grouper	N	Brought on Board (Retained/Discarded)	Live - Healthy	Discarded - Live and Healthy (Vented)	Not Applicable	
19	NOCAM	Epinephelus morio	Red Grouper	N	Brought on Board (Retained/Discarded)	Live - Healthy	Retained	Not Applicable	
19	NOCAM	Epinephelus morio	Red Grouper	N	Brought on Board (Retained/Discarded)	Live - Healthy	Discarded - Live and Healthy (Vented)	Not Applicable	
19	NOCAM	Epinephelus morio	Red Grouper	N	Brought on Board (Retained/Discarded)	Live - Healthy	Retained	Not Applicable	
19	NOCAM	Epinephelus morio	Red Grouper	N	Brought on Board (Retained/Discarded)	Live - Healthy	Discarded - Live and Healthy (Vented)	Not Applicable	
19	NOCAM	Epinephelus morio	Red Grouper	N	Brought on Board (Retained/Discarded)	Live - Healthy	Retained	Not Applicable	
19	NOCAM	Epinephelus morio	Red Grouper	N	Brought on Board (Retained/Discarded)	Live - Healthy	Retained	Not Applicable	
19	NOCAM	Epinephelus morio	Red Grouper	N	Brought on Board (Retained/Discarded)	Live - Healthy	Retained	Not Applicable	
19	NOCAM	Epinephelus morio	Red Grouper	N	Brought on Board (Retained/Discarded)	Live - Healthy	Retained	Not Applicable	
19	NOCAM	Epinephelus morio	Red Grouper	N	Brought on Board (Retained/Discarded)	Live - Healthy	Discarded - Live and Healthy (Vented)	Not Applicable	
19	NOCAM	Epinephelus morio	Red Grouper	N	Brought on Board (Retained/Discarded)	Live - Stomach and/or Eyes Protruding	Retained	Not Applicable	
19	NOCAM	Epinephelus morio	Red Grouper	N	Brought on Board (Retained/Discarded)	Live - Healthy	Retained	Not Applicable	
19	NOCAM	Epinephelus morio	Red Grouper	N	Brought on Board (Retained/Discarded)	Live - Stomach and/or Eyes Protruding	Retained	Not Applicable	
19	NOCAM	Lutjanus campechanus	Red Snapper	N	Brought on Board (Retained/Discarded)	Live - Healthy	Discarded - Live and Healthy (Vented)	Not Applicable	
19	NOCAM	Lutjanus synagris	Lane Snapper	N	Brought on Board (Retained/Discarded)	Live - Stomach and/or Eyes Protruding	Retained	Not Applicable	
19	NOCAM	Remora spp.	Remora (all)	N	Brought on Board (Retained/Discarded)	Live - Healthy	Retained as Bait	Not Applicable	
19	UCAM	Calamus bajonada	Jolthead Porgy	N	Brought on Board (Retained/Discarded)	Live - Healthy	Retained	Not Applicable	
19	UCAM	Carcharhinus plumbeus	Sandbar Shark	Y	Cut-off at Rail (No Entanglement)	Live - Healthy	Discarded - Live and Healthy (Not Vented)	Male (Claspers)	Large (Greater than 2m)
19	UCAM	Epinephelus morio	Red Grouper	N	Brought on Board (Retained/Discarded)	Live - Healthy	Retained	Not Applicable	
19	UCAM	Epinephelus morio	Red Grouper	N	Brought on Board (Retained/Discarded)	Live - Healthy	Retained	Not Applicable	
19	UCAM	Epinephelus morio	Red Grouper	N	Brought on Board (Retained/Discarded)	Live - Healthy	Retained	Not Applicable	
19	UCAM	Epinephelus morio	Red Grouper	N	Brought on Board (Retained/Discarded)	Live - Healthy	Discarded - Live and Healthy (Vented)	Not Applicable	
19	UCAM	Epinephelus morio	Red Grouper	N	Brought on Board (Retained/Discarded)	Live - Healthy	Discarded - Live and Healthy (Vented)	Not Applicable	
19	UCAM	Epinephelus morio	Red Grouper	N	Brought on Board (Retained/Discarded)	Live - Healthy	Retained	Not Applicable	
19	UCAM	Epinephelus morio	Red Grouper	N	Brought on Board (Retained/Discarded)	Live - Healthy	Retained	Not Applicable	
19	UCAM	Epinephelus morio	Red Grouper	N	Brought on Board (Retained/Discarded)	Live - Healthy	Retained	Not Applicable	
19	UCAM	Epinephelus morio	Red Grouper	N	Brought on Board (Retained/Discarded)	Live - Healthy	Discarded - Live and Healthy (Vented)	Not Applicable	
19	UCAM	Epinephelus morio	Red Grouper	N	Brought on Board (Retained/Discarded)	Live - Healthy	Retained	Not Applicable	
19	UCAM	Epinephelus morio	Red Grouper	N	Brought on Board (Retained/Discarded)	Live - Healthy	Retained	Not Applicable	
19	UCAM	Epinephelus morio	Red Grouper	N	Brought on Board (Retained/Discarded)	Live - Healthy	Discarded - Live and Healthy (Vented)	Not Applicable	
19	UCAM	Epinephelus morio	Red Grouper	N	Brought on Board (Retained/Discarded)	Live - Healthy	Retained	Not Applicable	
19	UCAM	Epinephelus morio	Red Grouper	N	Brought on Board (Retained/Discarded)	Live - Healthy	Retained	Not Applicable	
19	UCAM	Epinephelus morio	Red Grouper	N	Brought on Board (Retained/Discarded)	Live - Healthy	Discarded - Live and Healthy (Vented)	Not Applicable	
19	UCAM	Epinephelus morio	Red Grouper	N	Brought on Board (Retained/Discarded)	Live - Healthy	Discarded - Live and Healthy (Vented)	Not Applicable	
19	UCAM	Epinephelus morio	Red Grouper	N	Brought on Board (Retained/Discarded)	Live - Healthy	Retained	Not Applicable	
19	UCAM	Epinephelus morio	Red Grouper	N	Brought on Board (Retained/Discarded)	Live - Healthy	Retained	Not Applicable	
19	UCAM	Epinephelus morio	Red Grouper	N	Brought on Board (Retained/Discarded)	Live - Stomach and/or Eyes Protruding	Retained	Not Applicable	
19	UCAM	Epinephelus morio	Red Grouper	N	Brought on Board (Retained/Discarded)	Live - Healthy	Discarded - Live and Healthy (Vented)	Not Applicable	
19	UCAM	Epinephelus morio	Red Grouper	N	Brought on Board (Retained/Discarded)	Live - Healthy	Retained	Not Applicable	

19	UCAM	Epinephelus morio	Red Grouper	N	Brought on Board (Retained/Discarded)	Live - Healthy	Discarded - Live and Healthy (Vented)	Not Applicable	
19	UCAM	Epinephelus morio	Red Grouper	N	Brought on Board (Retained/Discarded)	Live - Healthy	Retained	Not Applicable	
19	UCAM	Epinephelus morio	Red Grouper	N	Brought on Board (Retained/Discarded)	Live - Healthy	Discarded - Live and Healthy (Vented)	Not Applicable	
19	UCAM	Epinephelus morio	Red Grouper	N	Brought on Board (Retained/Discarded)	Live - Healthy	Retained	Not Applicable	
19	UCAM	Epinephelus morio	Red Grouper	N	Brought on Board (Retained/Discarded)	Live - Healthy	Discarded - Live and Healthy (Vented)	Not Applicable	
19	UCAM	Epinephelus morio	Red Grouper	N	Brought on Board (Retained/Discarded)	Live - Healthy	Retained	Not Applicable	
19	UCAM	Epinephelus morio	Red Grouper	N	Brought on Board (Retained/Discarded)	Dead on Arrival - Damaged	Discarded - Dead	Not Applicable	
19	UCAM	Epinephelus morio	Red Grouper	N	Brought on Board (Retained/Discarded)	Live - Healthy	Discarded - Live and Healthy (Vented)	Not Applicable	
19	UCAM	Epinephelus morio	Red Grouper	N	Brought on Board (Retained/Discarded)	Live - Healthy	Retained	Not Applicable	
19	UCAM	Epinephelus morio	Red Grouper	N	Brought on Board (Retained/Discarded)	Live - Healthy	Retained	Not Applicable	
19	UCAM	Epinephelus morio	Red Grouper	N	Brought on Board (Retained/Discarded)	Live - Healthy	Retained	Not Applicable	
19	UCAM	Epinephelus morio	Red Grouper	N	Brought on Board (Retained/Discarded)	Live - Healthy	Retained	Not Applicable	
19	UCAM	Remora spp.	Remora (all)	N	Brought on Board (Retained/Discarded)	Live - Healthy	Retained as Bait	Not Applicable	
20	NOCAM	Carcharhinus plumbeus	Sandbar Shark	Y	Cut-off at Rail (No Entanglement)	Live - Healthy	Discarded - Live and Healthy (Not Vented)	Known Adult - Undetermined Sex	Large (Greater than 2m)
20	NOCAM	Epinephelus morio	Red Grouper	N	Brought on Board (Retained/Discarded)	Live - Healthy	Retained	Not Applicable	
20	NOCAM	Epinephelus morio	Red Grouper	N	Brought on Board (Retained/Discarded)	Live - Stomach and/or Eyes Protruding	Discarded - Live and Healthy (Not Vented)	Not Applicable	
20	NOCAM	Epinephelus morio	Red Grouper	N	Brought on Board (Retained/Discarded)	Live - Healthy	Retained	Not Applicable	
20	NOCAM	Epinephelus morio	Red Grouper	N	Brought on Board (Retained/Discarded)	Live - Stomach and/or Eyes Protruding	Retained	Not Applicable	
20	NOCAM	Epinephelus morio	Red Grouper	N	Brought on Board (Retained/Discarded)	Live - Healthy	Retained	Not Applicable	
20	NOCAM	Epinephelus morio	Red Grouper	N	Brought on Board (Retained/Discarded)	Live - Stomach and/or Eyes Protruding	Discarded - Live and Healthy (Vented)	Not Applicable	
20	NOCAM	Epinephelus morio	Red Grouper	N	Brought on Board (Retained/Discarded)	Live - Healthy	Retained	Not Applicable	
20	NOCAM	Epinephelus morio	Red Grouper	N	Brought on Board (Retained/Discarded)	Live - Stomach and/or Eyes Protruding	Discarded - Live and Healthy (Vented)	Not Applicable	
20	NOCAM	Epinephelus morio	Red Grouper	N	Brought on Board (Retained/Discarded)	Live - Healthy	Retained	Not Applicable	
20	NOCAM	Epinephelus morio	Red Grouper	N	Brought on Board (Retained/Discarded)	Live - Healthy	Discarded - Live and Healthy (Vented)	Not Applicable	
20	NOCAM	Epinephelus morio	Red Grouper	N	Brought on Board (Retained/Discarded)	Live - Healthy	Discarded - Live and Healthy (Vented)	Not Applicable	
20	NOCAM	Epinephelus morio	Red Grouper	N	Brought on Board (Retained/Discarded)	Live - Stomach and/or Eyes Protruding	Discarded - Live and Healthy (Vented)	Not Applicable	
20	NOCAM	Epinephelus morio	Red Grouper	N	Brought on Board (Retained/Discarded)	Live - Stomach and/or Eyes Protruding	Retained	Not Applicable	
20	NOCAM	Epinephelus morio	Red Grouper	N	Brought on Board (Retained/Discarded)	Live - Stomach and/or Eyes Protruding	Discarded - Live and Healthy (Vented)	Not Applicable	
20	NOCAM	Epinephelus morio	Red Grouper	N	Brought on Board (Retained/Discarded)	Live - Healthy	Discarded - Live and Healthy (Vented)	Not Applicable	
20	NOCAM	Epinephelus morio	Red Grouper	N	Brought on Board (Retained/Discarded)	Live - Healthy	Retained	Not Applicable	
20	NOCAM	Epinephelus morio	Red Grouper	N	Brought on Board (Retained/Discarded)	Live - Healthy	Retained	Not Applicable	
20	NOCAM	Epinephelus morio	Red Grouper	N	Brought on Board (Retained/Discarded)	Live - Healthy	Retained	Not Applicable	
20	NOCAM	Epinephelus morio	Red Grouper	N	Brought on Board (Retained/Discarded)	Live - Stomach and/or Eyes Protruding	Retained	Not Applicable	
20	NOCAM	Epinephelus morio	Red Grouper	N	Brought on Board (Retained/Discarded)	Live - Stomach and/or Eyes Protruding	Retained	Not Applicable	
20	NOCAM	Epinephelus morio	Red Grouper	N	Brought on Board (Retained/Discarded)	Live - Healthy	Discarded - Live and Healthy (Vented)	Not Applicable	
20	NOCAM	Epinephelus morio	Red Grouper	N	Brought on Board (Retained/Discarded)	Live - Stomach and/or Eyes Protruding	Retained	Not Applicable	
20	NOCAM	Epinephelus morio	Red Grouper	N	Brought on Board (Retained/Discarded)	Live - Healthy	Discarded - Live and Healthy (Vented)	Not Applicable	
20	NOCAM	Epinephelus morio	Red Grouper	N	Brought on Board (Retained/Discarded)	Live - Stomach and/or Eyes Protruding	Retained	Not Applicable	
20	NOCAM	Epinephelus morio	Red Grouper	N	Brought on Board (Retained/Discarded)	Live - Healthy	Retained	Not Applicable	
20	NOCAM	Epinephelus morio	Red Grouper	N	Brought on Board (Retained/Discarded)	Live - Healthy	Retained	Not Applicable	
20	NOCAM	Epinephelus morio	Red Grouper	N	Brought on Board (Retained/Discarded)	Live - Stomach and/or Eyes Protruding	Retained	Not Applicable	
20	NOCAM	Epinephelus morio	Red Grouper	N	Brought on Board (Retained/Discarded)	Live - Healthy	Discarded - Live and Healthy (Vented)	Not Applicable	
20	NOCAM	Epinephelus morio	Red Grouper	N	Brought on Board (Retained/Discarded)	Live - Stomach and/or Eyes Protruding	Discarded - Live and Healthy (Vented)	Not Applicable	
20	NOCAM	Epinephelus morio	Red Grouper	N	Brought on Board (Retained/Discarded)	Live - Stomach and/or Eyes Protruding	Retained	Not Applicable	
20	NOCAM	Epinephelus morio	Red Grouper	N	Brought on Board (Retained/Discarded)	Live - Healthy	Retained	Not Applicable	
20	NOCAM	Epinephelus morio	Red Grouper	N	Brought on Board (Retained/Discarded)	Live - Stomach and/or Eyes Protruding	Retained	Not Applicable	
20	NOCAM	Epinephelus morio	Red Grouper	N	Brought on Board (Retained/Discarded)	Live - Healthy	Discarded - Live and Healthy (Vented)	Not Applicable	
20	NOCAM	Epinephelus morio	Red Grouper	N	Brought on Board (Retained/Discarded)	Live - Stomach and/or Eyes Protruding	Discarded - Live and Healthy (Vented)	Not Applicable	
20	NOCAM	Epinephelus morio	Red Grouper	N	Not Handled (Dropped Off)	Live - Healthy	Discarded - Live and Healthy (Not Vented)	Not Applicable	
20	NOCAM	Epinephelus morio	Red Grouper	N	Brought on Board (Retained/Discarded)	Live - Healthy	Retained	Not Applicable	
20	NOCAM	Epinephelus morio	Red Grouper	N	Brought on Board (Retained/Discarded)	Live - Stomach and/or Eyes Protruding	Discarded - Live and Healthy (Vented)	Not Applicable	
20	NOCAM	Epinephelus morio	Red Grouper	N	Brought on Board (Retained/Discarded)	Live - Healthy	Discarded - Live and Healthy (Vented)	Not Applicable	
20	NOCAM	Epinephelus morio	Red Grouper	N	Brought on Board (Retained/Discarded)	Live - Stomach and/or Eyes Protruding	Retained	Not Applicable	
20	NOCAM	Epinephelus morio	Red Grouper	N	Brought on Board (Retained/Discarded)	Live - Healthy	Discarded - Live and Healthy (Vented)	Not Applicable	
20	NOCAM	Epinephelus morio	Red Grouper	N	Brought on Board (Retained/Discarded)	Live - Healthy	Retained	Not Applicable	
20	NOCAM	Epinephelus morio	Red Grouper	N	Brought on Board (Retained/Discarded)	Live - Healthy	Retained	Not Applicable	
20	NOCAM	Epinephelus morio	Red Grouper	N	Brought on Board (Retained/Discarded)	Live - Stomach and/or Eyes Protruding	Discarded - Live and Healthy (Vented)	Not Applicable	
20	NOCAM	Epinephelus morio	Red Grouper	N	Brought on Board (Retained/Discarded)	Live - Healthy	Discarded - Live and Healthy (Vented)	Not Applicable	
20	NOCAM	Epinephelus morio	Red Grouper	N	Brought on Board (Retained/Discarded)	Live - Healthy	Retained	Not Applicable	
20	NOCAM	Epinephelus morio	Red Grouper	N	Brought on Board (Retained/Discarded)	Live - Healthy	Retained	Not Applicable	
20	NOCAM	Epinephelus morio	Red Grouper	N	Brought on Board (Retained/Discarded)	Live - Stomach and/or Eyes Protruding	Retained	Not Applicable	
20	NOCAM	Epinephelus morio	Red Grouper	N	Brought on Board (Retained/Discarded)	Live - Healthy	Discarded - Live and Healthy (Vented)	Not Applicable	
20	NOCAM	Epinephelus morio	Red Grouper	N	Brought on Board (Retained/Discarded)	Live - Stomach and/or Eyes Protruding	Discarded - Live and Healthy (Vented)	Not Applicable	
20	NOCAM	Epinephelus morio	Red Grouper	N	Brought on Board (Retained/Discarded)	Live - Healthy	Retained	Not Applicable	
20	NOCAM	Epinephelus morio	Red Grouper	N	Brought on Board (Retained/Discarded)	Live - Healthy	Discarded - Live and Healthy (Vented)	Not Applicable	
20	NOCAM	Epinephelus morio	Red Grouper	N	Brought on Board (Retained/Discarded)	Live - Healthy	Discarded - Live and Healthy (Vented)	Not Applicable	
20	NOCAM	Epinephelus morio	Red Grouper	N	Brought on Board (Retained/Discarded)	Live - Healthy	Retained	Not Applicable	
20	NOCAM	Epinephelus morio	Red Grouper	N	Brought on Board (Retained/Discarded)	Live - Healthy	Discarded - Live and Healthy (Vented)	Not Applicable	
20	NOCAM	Epinephelus morio	Red Grouper	N	Brought on Board (Retained/Discarded)	Live - Stomach and/or Eyes Protruding	Discarded - Live and Healthy (Vented)	Not Applicable	
20	NOCAM	Epinephelus morio	Red Grouper	N	Brought on Board (Retained/Discarded)	Live - Healthy	Retained	Not Applicable	
20	NOCAM	Epinephelus morio	Red Grouper	N	Brought on Board (Retained/Discarded)	Live - Stomach and/or Eyes Protruding	Retained	Not Applicable	
20	NOCAM	Epinephelus morio	Red Grouper	N	Brought on Board (Retained/Discarded)	Live - Healthy	Retained	Not Applicable	
20	NOCAM	Epinephelus morio	Red Grouper	N	Brought on Board (Retained/Discarded)	Live - Stomach and/or Eyes Protruding	Discarded - Live and Healthy (Vented)	Not Applicable	
20	NOCAM	Epinephelus morio	Red Grouper	N	Brought on Board (Retained/Discarded)	Live - Healthy	Discarded - Live and Healthy (Vented)	Not Applicable	
20	NOCAM	Epinephelus morio	Red Grouper	N	Brought on Board (Retained/Discarded)	Live - Stomach and/or Eyes Protruding	Discarded - Live and Healthy (Vented)	Not Applicable	
20	NOCAM	Epinephelus morio	Red Grouper	N	Brought on Board (Retained/Discarded)	Live - Healthy	Discarded - Live and Healthy (Vented)	Not Applicable	
20	NOCAM	Epinephelus morio	Red Grouper	N	Brought on Board (Retained/Discarded)	Live - Healthy	Retained	Not Applicable	
20	NOCAM	Epinephelus morio	Red Grouper	N	Brought on Board (Retained/Discarded)	Live - Stomach and/or Eyes Protruding	Discarded - Live and Healthy (Vented)	Not Applicable	
20	NOCAM	Epinephelus morio	Red Grouper	N	Brought on Board (Retained/Discarded)	Live - Healthy	Retained	Not Applicable	
20	NOCAM	Epinephelus morio	Red Grouper	N	Brought on Board (Retained/Discarded)	Live - Healthy	Retained	Not Applicable	
20	NOCAM	Epinephelus morio	Red Grouper	N	Brought on Board (Retained/Discarded)	Live - Healthy	Discarded - Live and Healthy (Vented)	Not Applicable	
20	NOCAM	Epinephelus morio	Red Grouper	N	Brought on Board (Retained/Discarded)	Live - Stomach and/or Eyes Protruding	Retained	Not Applicable	
20	NOCAM	Epinephelus morio	Red Grouper	N	Brought on Board (Retained/Discarded)	Live - Healthy	Retained	Not Applicable	
20	NOCAM	Galeocerdo cuvier	Tiger Shark	Y	Cut-off at Rail (No Entanglement)	Live - Healthy	Discarded - Live and Healthy (Not Vented)	Unknown Maturity and/or Sex	Medium (Between 1m and 2m)
20	NOCAM	Opsanus spp.	Toadfish (all)	N	Brought on Board (Retained/Discarded)	Live - Healthy	Retained as Bait	Not Applicable	
20	NOCAM	Rhizoprionodon terraenovae	Atlantic Sharpnose Shark	Y	Brought on Board (Retained/Discarded)	Live - Healthy	Discarded - Live and Healthy (Not Vented)	Female	Small (Less than 1m)
20	NOCAM	Sphyrna lewini	Scalloped Hammerhead	Y	Cut-off at Rail (No Entanglement)	Live - Healthy	Discarded - Live and Healthy (Not Vented)	Unknown Maturity and/or Sex	Medium (Between 1m and 2m)
20	UCAM	Carcharhinus plumbeus	Sandbar Shark	Y	Cut-off at Rail (No Entanglement)	Live - Healthy	Discarded - Live and Healthy (Not Vented)	Male (Claspers)	Large (Greater than 2m)
20	UCAM	Epinephelus morio	Red Grouper	N	Brought on Board (Retained/Discarded)	Live - Healthy	Discarded - Live and Healthy (Vented)	Not Applicable	
20	UCAM	Epinephelus morio	Red Grouper	N	Brought on Board (Retained/Discarded)	Live - Stomach and/or Eyes Protruding	Discarded - Live and Healthy (Vented)	Not Applicable	
20	UCAM	Epinephelus morio	Red Grouper	N	Brought on Board (Retained/Discarded)	Live - Stomach and/or Eyes Protruding	Discarded - Live and Healthy (Vented)	Not Applicable	
20	UCAM	Epinephelus morio	Red Grouper	N	Brought on Board (Retained/Discarded)	Live - Healthy	Discarded - Live and Healthy (Vented)	Not Applicable	

20	UCAM	Epinephelus morio	Red Grouper	N	Brought on Board (Retained/Discarded)	Live - Healthy	Retained	Not Applicable	
20	UCAM	Epinephelus morio	Red Grouper	N	Brought on Board (Retained/Discarded)	Live - Healthy	Discarded - Live and Healthy (Vented)	Not Applicable	
20	UCAM	Epinephelus morio	Red Grouper	N	Brought on Board (Retained/Discarded)	Live - Healthy	Retained	Not Applicable	
20	UCAM	Epinephelus morio	Red Grouper	N	Brought on Board (Retained/Discarded)	Live - Healthy	Discarded - Live and Healthy (Vented)	Not Applicable	
20	UCAM	Epinephelus morio	Red Grouper	N	Brought on Board (Retained/Discarded)	Live - Healthy	Discarded - Live and Healthy (Vented)	Not Applicable	
20	UCAM	Epinephelus morio	Red Grouper	N	Brought on Board (Retained/Discarded)	Live - Stomach and/or Eyes Protruding	Discarded - Live and Healthy (Vented)	Not Applicable	
20	UCAM	Epinephelus morio	Red Grouper	N	Brought on Board (Retained/Discarded)	Live - Stomach and/or Eyes Protruding	Discarded - Live and Healthy (Vented)	Not Applicable	
20	UCAM	Epinephelus morio	Red Grouper	N	Brought on Board (Retained/Discarded)	Live - Healthy	Discarded - Live and Healthy (Vented)	Not Applicable	
20	UCAM	Epinephelus morio	Red Grouper	N	Brought on Board (Retained/Discarded)	Live - Healthy	Retained	Not Applicable	
20	UCAM	Epinephelus morio	Red Grouper	N	Brought on Board (Retained/Discarded)	Live - Healthy	Discarded - Live and Healthy (Vented)	Not Applicable	
20	UCAM	Epinephelus morio	Red Grouper	N	Brought on Board (Retained/Discarded)	Live - Stomach and/or Eyes Protruding	Discarded - Live and Healthy (Vented)	Not Applicable	
20	UCAM	Epinephelus morio	Red Grouper	N	Brought on Board (Retained/Discarded)	Live - Healthy	Retained	Not Applicable	
20	UCAM	Epinephelus morio	Red Grouper	N	Brought on Board (Retained/Discarded)	Live - Healthy	Retained	Not Applicable	
20	UCAM	Epinephelus morio	Red Grouper	N	Brought on Board (Retained/Discarded)	Live - Stomach and/or Eyes Protruding	Retained	Not Applicable	
20	UCAM	Epinephelus morio	Red Grouper	N	Brought on Board (Retained/Discarded)	Live - Healthy	Retained	Not Applicable	
20	UCAM	Epinephelus morio	Red Grouper	N	Brought on Board (Retained/Discarded)	Live - Healthy	Retained	Not Applicable	
20	UCAM	Epinephelus morio	Red Grouper	N	Brought on Board (Retained/Discarded)	Live - Stomach and/or Eyes Protruding	Retained	Not Applicable	
20	UCAM	Epinephelus morio	Red Grouper	N	Brought on Board (Retained/Discarded)	Live - Stomach and/or Eyes Protruding	Discarded - Live and Healthy (Vented)	Not Applicable	
20	UCAM	Epinephelus morio	Red Grouper	N	Brought on Board (Retained/Discarded)	Live - Stomach and/or Eyes Protruding	Retained	Not Applicable	
20	UCAM	Epinephelus morio	Red Grouper	N	Brought on Board (Retained/Discarded)	Live - Stomach and/or Eyes Protruding	Discarded - Live and Healthy (Vented)	Not Applicable	
20	UCAM	Epinephelus morio	Red Grouper	N	Brought on Board (Retained/Discarded)	Live - Healthy	Discarded - Live and Healthy (Vented)	Not Applicable	
20	UCAM	Epinephelus morio	Red Grouper	N	Brought on Board (Retained/Discarded)	Live - Stomach and/or Eyes Protruding	Retained	Not Applicable	
20	UCAM	Epinephelus morio	Red Grouper	N	Not Handled (Dropped Off)	Live - Healthy	Discarded - Live and Healthy (Not Vented)	Not Applicable	
20	UCAM	Epinephelus morio	Red Grouper	N	Brought on Board (Retained/Discarded)	Live - Stomach and/or Eyes Protruding	Retained	Not Applicable	
20	UCAM	Epinephelus morio	Red Grouper	N	Brought on Board (Retained/Discarded)	Live - Stomach and/or Eyes Protruding	Retained	Not Applicable	
20	UCAM	Epinephelus morio	Red Grouper	N	Brought on Board (Retained/Discarded)	Live - Stomach and/or Eyes Protruding	Retained	Not Applicable	
20	UCAM	Epinephelus morio	Red Grouper	N	Brought on Board (Retained/Discarded)	Live - Healthy	Retained	Not Applicable	
20	UCAM	Epinephelus morio	Red Grouper	N	Brought on Board (Retained/Discarded)	Live - Healthy	Retained	Not Applicable	
20	UCAM	Epinephelus morio	Red Grouper	N	Brought on Board (Retained/Discarded)	Live - Healthy	Discarded - Live and Healthy (Vented)	Not Applicable	
20	UCAM	Epinephelus morio	Red Grouper	N	Brought on Board (Retained/Discarded)	Live - Healthy	Retained	Not Applicable	
20	UCAM	Epinephelus morio	Red Grouper	N	Brought on Board (Retained/Discarded)	Live - Healthy	Retained	Not Applicable	
20	UCAM	Epinephelus morio	Red Grouper	N	Brought on Board (Retained/Discarded)	Live - Healthy	Discarded - Live and Healthy (Vented)	Not Applicable	
20	UCAM	Epinephelus morio	Red Grouper	N	Brought on Board (Retained/Discarded)	Live - Stomach and/or Eyes Protruding	Discarded - Live and Healthy (Vented)	Not Applicable	
20	UCAM	Epinephelus morio	Red Grouper	N	Brought on Board (Retained/Discarded)	Live - Healthy	Discarded - Live and Healthy (Vented)	Not Applicable	
20	UCAM	Epinephelus morio	Red Grouper	N	Brought on Board (Retained/Discarded)	Live - Healthy	Retained	Not Applicable	
20	UCAM	Epinephelus morio	Red Grouper	N	Brought on Board (Retained/Discarded)	Live - Healthy	Discarded - Live and Healthy (Vented)	Not Applicable	
20	UCAM	Epinephelus morio	Red Grouper	N	Brought on Board (Retained/Discarded)	Live - Healthy	Retained	Not Applicable	
20	UCAM	Epinephelus morio	Red Grouper	N	Brought on Board (Retained/Discarded)	Live - Healthy	Discarded - Live and Healthy (Vented)	Not Applicable	
20	UCAM	Epinephelus morio	Red Grouper	N	Brought on Board (Retained/Discarded)	Live - Stomach and/or Eyes Protruding	Retained	Not Applicable	
20	UCAM	Epinephelus morio	Red Grouper	N	Brought on Board (Retained/Discarded)	Live - Stomach and/or Eyes Protruding	Retained	Not Applicable	
20	UCAM	Epinephelus morio	Red Grouper	N	Brought on Board (Retained/Discarded)	Live - Healthy	Retained	Not Applicable	
20	UCAM	Epinephelus morio	Red Grouper	N	Brought on Board (Retained/Discarded)	Live - Healthy	Discarded - Live and Healthy (Vented)	Not Applicable	
20	UCAM	Epinephelus morio	Red Grouper	N	Brought on Board (Retained/Discarded)	Live - Healthy	Retained	Not Applicable	
20	UCAM	Epinephelus morio	Red Grouper	N	Brought on Board (Retained/Discarded)	Live - Healthy	Discarded - Live and Healthy (Vented)	Not Applicable	
20	UCAM	Epinephelus morio	Red Grouper	N	Brought on Board (Retained/Discarded)	Live - Healthy	Discarded - Live and Healthy (Vented)	Not Applicable	
20	UCAM	Epinephelus morio	Red Grouper	N	Brought on Board (Retained/Discarded)	Live - Healthy	Retained	Not Applicable	
20	UCAM	Epinephelus morio	Red Grouper	N	Brought on Board (Retained/Discarded)	Live - Stomach and/or Eyes Protruding	Retained	Not Applicable	
20	UCAM	Epinephelus morio	Red Grouper	N	Brought on Board (Retained/Discarded)	Live - Healthy	Retained	Not Applicable	
20	UCAM	Epinephelus morio	Red Grouper	N	Brought on Board (Retained/Discarded)	Live - Healthy	Discarded - Live and Healthy (Vented)	Not Applicable	
20	UCAM	Epinephelus morio	Red Grouper	N	Brought on Board (Retained/Discarded)	Live - Healthy	Retained	Not Applicable	
20	UCAM	Epinephelus morio	Red Grouper	N	Brought on Board (Retained/Discarded)	Live - Healthy	Retained	Not Applicable	
20	UCAM	Epinephelus morio	Red Grouper	N	Brought on Board (Retained/Discarded)	Live - Stomach and/or Eyes Protruding	Discarded - Live and Healthy (Vented)	Not Applicable	
20	UCAM	Epinephelus morio	Red Grouper	N	Brought on Board (Retained/Discarded)	Live - Healthy	Discarded - Live and Healthy (Vented)	Not Applicable	
20	UCAM	Epinephelus morio	Red Grouper	N	Brought on Board (Retained/Discarded)	Live - Healthy	Discarded - Live and Healthy (Vented)	Not Applicable	
20	UCAM	Epinephelus morio	Red Grouper	N	Brought on Board (Retained/Discarded)	Live - Stomach and/or Eyes Protruding	Retained	Not Applicable	
20	UCAM	Epinephelus morio	Red Grouper	N	Brought on Board (Retained/Discarded)	Live - Healthy	Discarded - Live and Healthy (Vented)	Not Applicable	
20	UCAM	Epinephelus morio	Red Grouper	N	Brought on Board (Retained/Discarded)	Live - Healthy	Discarded - Live and Healthy (Vented)	Not Applicable	
20	UCAM	Epinephelus morio	Red Grouper	N	Brought on Board (Retained/Discarded)	Live - Healthy	Discarded - Live and Healthy (Vented)	Not Applicable	
20	UCAM	Epinephelus morio	Red Grouper	N	Brought on Board (Retained/Discarded)	Live - Healthy	Discarded - Live and Healthy (Vented)	Not Applicable	
20	UCAM	Epinephelus morio	Red Grouper	N	Brought on Board (Retained/Discarded)	Live - Stomach and/or Eyes Protruding	Retained	Not Applicable	
20	UCAM	Epinephelus morio	Red Grouper	N	Brought on Board (Retained/Discarded)	Live - Stomach and/or Eyes Protruding	Discarded - Live and Healthy (Vented)	Not Applicable	
20	UCAM	Epinephelus morio	Red Grouper	N	Brought on Board (Retained/Discarded)	Live - Stomach and/or Eyes Protruding	Retained	Not Applicable	
20	UCAM	Epinephelus morio	Red Grouper	N	Brought on Board (Retained/Discarded)	Live - Healthy	Discarded - Live and Healthy (Vented)	Not Applicable	
20	UCAM	Epinephelus morio	Red Grouper	N	Brought on Board (Retained/Discarded)	Live - Stomach and/or Eyes Protruding	Discarded - Live and Healthy (Vented)	Not Applicable	
20	UCAM	Epinephelus morio	Red Grouper	N	Brought on Board (Retained/Discarded)	Live - Stomach and/or Eyes Protruding	Retained	Not Applicable	
20	UCAM	Galeocerdo cuvier	Tiger Shark	Y	Cut-off at Rail (No Entanglement)	Live - Healthy	Discarded - Live and Healthy (Not Vented)	Unknown Maturity and/or Sex	Medium (Between 1m and 2m)
20	UCAM	Opsanus spp.	Toadfish (all)	N	Brought on Board (Retained/Discarded)	Live - Healthy	Retained as Bait	Not Applicable	
20	UCAM	Rhizoprionodon terraenovae	Atlantic Sharpnose Shark	Y	Brought on Board (Retained/Discarded)	Live - Healthy	Discarded - Live and Healthy (Not Vented)	Female	Small (Less than 1m)
20	UCAM	Sphyrna lewini	Scalloped Hammerhead	Y	Cut-off at Rail (No Entanglement)	Live - Healthy	Discarded - Live and Healthy (Not Vented)	Male (Claspers)	Medium (Between 1m and 2m)
21	NOCAM	Carcharhinus plumbeus	Sandbar Shark	Y	Cut-off at Rail (No Entanglement)	Live - Healthy	Discarded - Live and Healthy (Not Vented)	Known Adult - Undetermined Sex	Large (Greater than 2m)
21	NOCAM	Carcharhinus plumbeus	Sandbar Shark	Y	Cut-off at Rail (No Entanglement)	Live - Healthy	Discarded - Live and Healthy (Not Vented)	Male (Claspers)	Medium (Between 1m and 2m)
21	NOCAM	Carcharhinus plumbeus	Sandbar Shark	Y	Cut-off at Rail (No Entanglement)	Live - Healthy	Discarded - Live and Healthy (Not Vented)	Known Adult - Undetermined Sex	Large (Greater than 2m)
21	NOCAM	Epinephelus morio	Red Grouper	N	Brought on Board (Retained/Discarded)	Live - Stomach and/or Eyes Protruding	Retained	Not Applicable	
21	NOCAM	Epinephelus morio	Red Grouper	N	Brought on Board (Retained/Discarded)	Live - Stomach and/or Eyes Protruding	Retained	Not Applicable	
21	NOCAM	Epinephelus morio	Red Grouper	N	Brought on Board (Retained/Discarded)	Live - Healthy	Retained	Not Applicable	
21	NOCAM	Epinephelus morio	Red Grouper	N	Brought on Board (Retained/Discarded)	Live - Stomach and/or Eyes Protruding	Discarded - Live and Healthy (Vented)	Not Applicable	
21	NOCAM	Epinephelus morio	Red Grouper	N	Brought on Board (Retained/Discarded)	Live - Healthy	Retained	Not Applicable	
21	NOCAM	Epinephelus morio	Red Grouper	N	Brought on Board (Retained/Discarded)	Live - Stomach and/or Eyes Protruding	Retained	Not Applicable	
21	NOCAM	Epinephelus morio	Red Grouper	N	Brought on Board (Retained/Discarded)	Live - Healthy	Retained	Not Applicable	
21	NOCAM	Epinephelus morio	Red Grouper	N	Brought on Board (Retained/Discarded)	Live - Healthy	Discarded - Live and Healthy (Vented)	Not Applicable	
21	NOCAM	Epinephelus morio	Red Grouper	N	Brought on Board (Retained/Discarded)	Live - Healthy	Retained	Not Applicable	
21	NOCAM	Epinephelus morio	Red Grouper	N	Brought on Board (Retained/Discarded)	Live - Healthy	Discarded - Live and Healthy (Vented)	Not Applicable	
21	NOCAM	Epinephelus morio	Red Grouper	N	Brought on Board (Retained/Discarded)	Live - Stomach and/or Eyes Protruding	Discarded - Live and Healthy (Vented)	Not Applicable	
21	NOCAM	Epinephelus morio	Red Grouper	N	Brought on Board (Retained/Discarded)	Live - Healthy	Discarded - Live and Healthy (Vented)	Not Applicable	
21	NOCAM	Epinephelus morio	Red Grouper	N	Brought on Board (Retained/Discarded)	Live - Healthy	Discarded - Live and Healthy (Vented)	Not Applicable	
21	NOCAM	Epinephelus morio	Red Grouper	N	Brought on Board (Retained/Discarded)	Live - Healthy	Retained	Not Applicable	
21	NOCAM	Epinephelus morio	Red Grouper	N	Brought on Board (Retained/Discarded)	Live - Stomach and/or Eyes Protruding	Retained	Not Applicable	
21	NOCAM	Epinephelus morio	Red Grouper	N	Brought on Board (Retained/Discarded)	Live - Healthy	Retained	Not Applicable	
21	NOCAM	Epinephelus morio	Red Grouper	N	Brought on Board (Retained/Discarded)	Live - Stomach and/or Eyes Protruding	Retained	Not Applicable	
21	NOCAM	Epinephelus morio	Red Grouper	N	Brought on Board (Retained/Discarded)	Live - Healthy	Retained	Not Applicable	
21	NOCAM	Epinephelus morio	Red Grouper	N	Brought on Board (Retained/Discarded)	Live - Stomach and/or Eyes Protruding	Retained	Not Applicable	
21	NOCAM	Epinephelus morio	Red Grouper	N	Brought on Board (Retained/Discarded)	Live - Healthy	Retained	Not Applicable	

21	NOCAM	Epinephelus morio	Red Grouper	N	Brought on Board (Retained/Discarded)	Live - Healthy	Discarded - Live and Healthy (Vented)	Not Applicable	
21	NOCAM	Epinephelus morio	Red Grouper	N	Brought on Board (Retained/Discarded)	Live - Stomach and/or Eyes Protruding	Retained	Not Applicable	
21	NOCAM	Epinephelus morio	Red Grouper	N	Brought on Board (Retained/Discarded)	Live - Healthy	Discarded - Live and Healthy (Vented)	Not Applicable	
21	NOCAM	Epinephelus morio	Red Grouper	N	Brought on Board (Retained/Discarded)	Live - Healthy	Discarded - Live and Healthy (Vented)	Not Applicable	
21	NOCAM	Epinephelus morio	Red Grouper	N	Brought on Board (Retained/Discarded)	Live - Healthy	Discarded - Live and Healthy (Vented)	Not Applicable	
21	NOCAM	Epinephelus morio	Red Grouper	N	Brought on Board (Retained/Discarded)	Live - Healthy	Retained	Not Applicable	
21	NOCAM	Epinephelus morio	Red Grouper	N	Brought on Board (Retained/Discarded)	Live - Stomach and/or Eyes Protruding	Retained	Not Applicable	
21	NOCAM	Epinephelus morio	Red Grouper	N	Brought on Board (Retained/Discarded)	Live - Healthy	Discarded - Live and Healthy (Vented)	Not Applicable	
21	NOCAM	Epinephelus morio	Red Grouper	N	Brought on Board (Retained/Discarded)	Live - Stomach and/or Eyes Protruding	Retained	Not Applicable	
21	NOCAM	Epinephelus morio	Red Grouper	N	Brought on Board (Retained/Discarded)	Live - Healthy	Retained	Not Applicable	
21	NOCAM	Epinephelus morio	Red Grouper	N	Brought on Board (Retained/Discarded)	Live - Stomach and/or Eyes Protruding	Discarded - Live and Healthy (Vented)	Not Applicable	
21	NOCAM	Epinephelus morio	Red Grouper	N	Brought on Board (Retained/Discarded)	Live - Healthy	Retained	Not Applicable	
21	NOCAM	Epinephelus morio	Red Grouper	N	Brought on Board (Retained/Discarded)	Live - Healthy	Discarded - Live and Healthy (Vented)	Not Applicable	
21	NOCAM	Epinephelus morio	Red Grouper	N	Brought on Board (Retained/Discarded)	Live - Stomach and/or Eyes Protruding	Retained	Not Applicable	
21	NOCAM	Epinephelus morio	Red Grouper	N	Brought on Board (Retained/Discarded)	Live - Stomach and/or Eyes Protruding	Discarded - Live and Healthy (Vented)	Not Applicable	
21	NOCAM	Epinephelus morio	Red Grouper	N	Brought on Board (Retained/Discarded)	Live - Stomach and/or Eyes Protruding	Discarded - Live and Healthy (Vented)	Not Applicable	
21	NOCAM	Epinephelus morio	Red Grouper	N	Brought on Board (Retained/Discarded)	Live - Stomach and/or Eyes Protruding	Discarded - Live and Healthy (Vented)	Not Applicable	
21	NOCAM	Epinephelus morio	Red Grouper	N	Brought on Board (Retained/Discarded)	Live - Healthy	Discarded - Live and Healthy (Vented)	Not Applicable	
21	NOCAM	Epinephelus morio	Red Grouper	N	Brought on Board (Retained/Discarded)	Live - Stomach and/or Eyes Protruding	Discarded - Live and Healthy (Vented)	Not Applicable	
21	NOCAM	Epinephelus morio	Red Grouper	N	Brought on Board (Retained/Discarded)	Live - Stomach and/or Eyes Protruding	Discarded - Live and Healthy (Vented)	Not Applicable	
21	NOCAM	Epinephelus morio	Red Grouper	N	Brought on Board (Retained/Discarded)	Live - Stomach and/or Eyes Protruding	Retained	Not Applicable	
21	NOCAM	Epinephelus morio	Red Grouper	N	Brought on Board (Retained/Discarded)	Live - Stomach and/or Eyes Protruding	Discarded - Live and Healthy (Vented)	Not Applicable	
21	NOCAM	Epinephelus morio	Red Grouper	N	Brought on Board (Retained/Discarded)	Live - Healthy	Discarded - Live and Healthy (Vented)	Not Applicable	
21	NOCAM	Epinephelus morio	Red Grouper	N	Brought on Board (Retained/Discarded)	Live - Healthy	Discarded - Live and Healthy (Vented)	Not Applicable	
21	NOCAM	Epinephelus morio	Red Grouper	N	Brought on Board (Retained/Discarded)	Live - Healthy	Retained	Not Applicable	
21	NOCAM	Epinephelus morio	Red Grouper	N	Brought on Board (Retained/Discarded)	Live - Healthy	Retained	Not Applicable	
21	NOCAM	Epinephelus morio	Red Grouper	N	Brought on Board (Retained/Discarded)	Live - Healthy	Retained	Not Applicable	
21	NOCAM	Epinephelus morio	Red Grouper	N	Brought on Board (Retained/Discarded)	Live - Healthy	Discarded - Live and Healthy (Vented)	Not Applicable	
21	NOCAM	Epinephelus morio	Red Grouper	N	Brought on Board (Retained/Discarded)	Live - Healthy	Discarded - Live and Healthy (Vented)	Not Applicable	
21	NOCAM	Epinephelus morio	Red Grouper	N	Brought on Board (Retained/Discarded)	Live - Stomach and/or Eyes Protruding	Discarded - Live and Healthy (Vented)	Not Applicable	
21	NOCAM	Epinephelus morio	Red Grouper	N	Brought on Board (Retained/Discarded)	Live - Healthy	Retained	Not Applicable	
21	NOCAM	Epinephelus morio	Red Grouper	N	Brought on Board (Retained/Discarded)	Live - Stomach and/or Eyes Protruding	Discarded - Live and Healthy (Vented)	Not Applicable	
21	NOCAM	Epinephelus morio	Red Grouper	N	Brought on Board (Retained/Discarded)	Live - Healthy	Discarded - Live and Healthy (Vented)	Not Applicable	
21	NOCAM	Epinephelus morio	Red Grouper	N	Brought on Board (Retained/Discarded)	Live - Stomach and/or Eyes Protruding	Discarded - Live and Healthy (Vented)	Not Applicable	
21	NOCAM	Epinephelus morio	Red Grouper	N	Brought on Board (Retained/Discarded)	Live - Healthy	Discarded - Live and Healthy (Vented)	Not Applicable	
21	NOCAM	Epinephelus morio	Red Grouper	N	Brought on Board (Retained/Discarded)	Live - Healthy	Discarded - Live and Healthy (Vented)	Not Applicable	
21	NOCAM	Epinephelus morio	Red Grouper	N	Brought on Board (Retained/Discarded)	Live - Stomach and/or Eyes Protruding	Retained	Not Applicable	
21	NOCAM	Lutjanus campechanus	Red Snapper	N	Brought on Board (Retained/Discarded)	Live - Healthy	Discarded - Live and Healthy (Vented)	Not Applicable	
21	NOCAM	Lutjanus campechanus	Red Snapper	N	Brought on Board (Retained/Discarded)	Live - Stomach and/or Eyes Protruding	Discarded - Live and Healthy (Vented)	Not Applicable	
21	NOCAM	Lutjanus campechanus	Red Snapper	N	Brought on Board (Retained/Discarded)	Dead on Arrival - Damaged	Discarded - Dead	Not Applicable	
21	NOCAM	Rhizoprionodon terraenovae	Atlantic Sharpnose Shark	Y	Brought on Board (Retained/Discarded)	Live - Healthy	Discarded - Live and Healthy (Not Vented)	Male (Claspers)	Small (Less than 1m)
21	UCAM	Carcharhinus plumbeus	Sandbar Shark	Y	Cut-off at Rail (No Entanglement)	Live - Healthy	Discarded - Live and Healthy (Not Vented)	Male (Claspers)	Large (Greater than 2m)
21	UCAM	Carcharhinus plumbeus	Sandbar Shark	Y	Cut-off at Rail (No Entanglement)	Live - Healthy	Discarded - Live and Healthy (Not Vented)	Male (Claspers)	Large (Greater than 2m)
21	UCAM	Carcharhinus plumbeus	Sandbar Shark	Y	Cut-off at Rail (No Entanglement)	Live - Healthy	Discarded - Live and Healthy (Not Vented)	Known Adult - Undetermined Sex	Large (Greater than 2m)
21	UCAM	Epinephelus morio	Red Grouper	N	Brought on Board (Retained/Discarded)	Live - Stomach and/or Eyes Protruding	Discarded - Live and Healthy (Vented)	Not Applicable	
21	UCAM	Epinephelus morio	Red Grouper	N	Brought on Board (Retained/Discarded)	Live - Healthy	Discarded - Live and Healthy (Vented)	Not Applicable	
21	UCAM	Epinephelus morio	Red Grouper	N	Brought on Board (Retained/Discarded)	Live - Healthy	Retained	Not Applicable	
21	UCAM	Epinephelus morio	Red Grouper	N	Brought on Board (Retained/Discarded)	Live - Stomach and/or Eyes Protruding	Retained	Not Applicable	
21	UCAM	Epinephelus morio	Red Grouper	N	Brought on Board (Retained/Discarded)	Live - Stomach and/or Eyes Protruding	Discarded - Live and Healthy (Vented)	Not Applicable	
21	UCAM	Epinephelus morio	Red Grouper	N	Brought on Board (Retained/Discarded)	Live - Healthy	Discarded - Live and Healthy (Vented)	Not Applicable	
21	UCAM	Epinephelus morio	Red Grouper	N	Brought on Board (Retained/Discarded)	Live - Stomach and/or Eyes Protruding	Retained	Not Applicable	
21	UCAM	Epinephelus morio	Red Grouper	N	Brought on Board (Retained/Discarded)	Live - Healthy	Retained	Not Applicable	
21	UCAM	Epinephelus morio	Red Grouper	N	Brought on Board (Retained/Discarded)	Live - Stomach and/or Eyes Protruding	Retained	Not Applicable	
21	UCAM	Epinephelus morio	Red Grouper	N	Brought on Board (Retained/Discarded)	Live - Healthy	Discarded - Live and Healthy (Vented)	Not Applicable	
21	UCAM	Epinephelus morio	Red Grouper	N	Brought on Board (Retained/Discarded)	Live - Healthy	Discarded - Live and Healthy (Vented)	Not Applicable	
21	UCAM	Epinephelus morio	Red Grouper	N	Brought on Board (Retained/Discarded)	Live - Healthy	Retained	Not Applicable	
21	UCAM	Epinephelus morio	Red Grouper	N	Brought on Board (Retained/Discarded)	Live - Stomach and/or Eyes Protruding	Retained	Not Applicable	
21	UCAM	Epinephelus morio	Red Grouper	N	Brought on Board (Retained/Discarded)	Live - Healthy	Discarded - Live and Healthy (Vented)	Not Applicable	
21	UCAM	Epinephelus morio	Red Grouper	N	Brought on Board (Retained/Discarded)	Live - Healthy	Discarded - Live and Healthy (Vented)	Not Applicable	
21	UCAM	Epinephelus morio	Red Grouper	N	Brought on Board (Retained/Discarded)	Live - Healthy	Retained	Not Applicable	
21	UCAM	Epinephelus morio	Red Grouper	N	Brought on Board (Retained/Discarded)	Live - Stomach and/or Eyes Protruding	Retained	Not Applicable	
21	UCAM	Epinephelus morio	Red Grouper	N	Brought on Board (Retained/Discarded)	Live - Stomach and/or Eyes Protruding	Retained	Not Applicable	
21	UCAM	Epinephelus morio	Red Grouper	N	Brought on Board (Retained/Discarded)	Live - Healthy	Discarded - Live and Healthy (Vented)	Not Applicable	
21	UCAM	Epinephelus morio	Red Grouper	N	Brought on Board (Retained/Discarded)	Live - Healthy	Retained	Not Applicable	
21	UCAM	Epinephelus morio	Red Grouper	N	Brought on Board (Retained/Discarded)	Live - Healthy	Discarded - Live and Healthy (Vented)	Not Applicable	
21	UCAM	Epinephelus morio	Red Grouper	N	Brought on Board (Retained/Discarded)	Live - Healthy	Retained	Not Applicable	
21	UCAM	Epinephelus morio	Red Grouper	N	Brought on Board (Retained/Discarded)	Live - Stomach and/or Eyes Protruding	Retained	Not Applicable	
21	UCAM	Epinephelus morio	Red Grouper	N	Brought on Board (Retained/Discarded)	Live - Stomach and/or Eyes Protruding	Retained	Not Applicable	
21	UCAM	Epinephelus morio	Red Grouper	N	Brought on Board (Retained/Discarded)	Live - Healthy	Discarded - Live and Healthy (Vented)	Not Applicable	
21	UCAM	Epinephelus morio	Red Grouper	N	Brought on Board (Retained/Discarded)	Live - Healthy	Discarded - Live and Healthy (Vented)	Not Applicable	
21	UCAM	Epinephelus morio	Red Grouper	N	Brought on Board (Retained/Discarded)	Live - Healthy	Discarded - Live and Healthy (Vented)	Not Applicable	
21	UCAM	Epinephelus morio	Red Grouper	N	Brought on Board (Retained/Discarded)	Live - Stomach and/or Eyes Protruding	Discarded - Live and Healthy (Vented)	Not Applicable	
21	UCAM	Epinephelus morio	Red Grouper	N	Brought on Board (Retained/Discarded)	Live - Stomach and/or Eyes Protruding	Retained	Not Applicable	
21	UCAM	Epinephelus morio	Red Grouper	N	Brought on Board (Retained/Discarded)	Live - Healthy	Retained	Not Applicable	
21	UCAM	Epinephelus morio	Red Grouper	N	Brought on Board (Retained/Discarded)	Live - Healthy	Discarded - Live and Healthy (Vented)	Not Applicable	
21	UCAM	Epinephelus morio	Red Grouper	N	Brought on Board (Retained/Discarded)	Live - Healthy	Retained	Not Applicable	
21	UCAM	Epinephelus morio	Red Grouper	N	Brought on Board (Retained/Discarded)	Live - Healthy	Discarded - Live and Healthy (Vented)	Not Applicable	
21	UCAM	Epinephelus morio	Red Grouper	N	Brought on Board (Retained/Discarded)	Live - Stomach and/or Eyes Protruding	Discarded - Live and Healthy (Vented)	Not Applicable	
21	UCAM	Epinephelus morio	Red Grouper	N	Brought on Board (Retained/Discarded)	Live - Healthy	Discarded - Live and Healthy (Vented)	Not Applicable	
21	UCAM	Epinephelus morio	Red Grouper	N	Brought on Board (Retained/Discarded)	Live - Stomach and/or Eyes Protruding	Discarded - Live and Healthy (Vented)	Not Applicable	
21	UCAM	Epinephelus morio	Red Grouper	N	Brought on Board (Retained/Discarded)	Live - Healthy	Discarded - Live and Healthy (Vented)	Not Applicable	
21	UCAM	Epinephelus morio	Red Grouper	N	Brought on Board (Retained/Discarded)	Live - Stomach and/or Eyes Protruding	Retained	Not Applicable	
21	UCAM	Epinephelus morio	Red Grouper	N	Brought on Board (Retained/Discarded)	Live - Healthy	Retained	Not Applicable	
21	UCAM	Epinephelus morio	Red Grouper	N	Brought on Board (Retained/Discarded)	Live - Stomach and/or Eyes Protruding	Retained	Not Applicable	
21	UCAM	Epinephelus morio	Red Grouper	N	Brought on Board (Retained/Discarded)	Live - Stomach and/or Eyes Protruding	Retained	Not Applicable	
21	UCAM	Epinephelus morio	Red Grouper	N	Brought on Board (Retained/Discarded)	Live - Stomach and/or Eyes Protruding	Discarded - Live and Healthy (Vented)	Not Applicable	
21	UCAM	Epinephelus morio	Red Grouper	N	Brought on Board (Retained/Discarded)	Live - Stomach and/or Eyes Protruding	Retained	Not Applicable	
21	UCAM	Epinephelus morio	Red Grouper	N	Brought on Board (Retained/Discarded)	Live - Healthy	Discarded - Live and Healthy (Vented)	Not Applicable	
21	UCAM	Epinephelus morio	Red Grouper	N	Brought on Board (Retained/Discarded)	Live - Stomach and/or Eyes Protruding	Retained	Not Applicable	
21	UCAM	Epinephelus morio	Red Grouper	N	Brought on Board (Retained/Discarded)	Live - Healthy	Discarded - Live and Healthy (Vented)	Not Applicable	
21	UCAM	Epinephelus morio	Red Grouper	N	Brought on Board (Retained/Discarded)	Live - Healthy	Retained	Not Applicable	
21	UCAM	Epinephelus morio	Red Grouper	N	Brought on Board (Retained/Discarded)	Live - Stomach and/or Eyes Protruding	Retained	Not Applicable	

21	UCAM	Epinephelus morio	Red Grouper	N	Brought on Board (Retained/Discarded)	Live - Stomach and/or Eyes Protruding	Discarded - Live and Healthy (Vented)	Not Applicable	
21	UCAM	Epinephelus morio	Red Grouper	N	Brought on Board (Retained/Discarded)	Live - Healthy	Discarded - Live and Healthy (Vented)	Not Applicable	
21	UCAM	Epinephelus morio	Red Grouper	N	Brought on Board (Retained/Discarded)	Live - Healthy	Retained	Not Applicable	
21	UCAM	Epinephelus morio	Red Grouper	N	Brought on Board (Retained/Discarded)	Live - Healthy	Discarded - Live and Healthy (Vented)	Not Applicable	
21	UCAM	Epinephelus morio	Red Grouper	N	Brought on Board (Retained/Discarded)	Live - Healthy	Discarded - Live and Healthy (Vented)	Not Applicable	
21	UCAM	Epinephelus morio	Red Grouper	N	Brought on Board (Retained/Discarded)	Live - Healthy	Retained	Not Applicable	
21	UCAM	Epinephelus morio	Red Grouper	N	Brought on Board (Retained/Discarded)	Live - Healthy	Retained	Not Applicable	
21	UCAM	Epinephelus morio	Red Grouper	N	Brought on Board (Retained/Discarded)	Live - Stomach and/or Eyes Protruding	Retained	Not Applicable	
21	UCAM	Epinephelus morio	Red Grouper	N	Brought on Board (Retained/Discarded)	Live - Healthy	Retained	Not Applicable	
21	UCAM	Epinephelus morio	Red Grouper	N	Brought on Board (Retained/Discarded)	Live - Healthy	Discarded - Live and Healthy (Vented)	Not Applicable	
21	UCAM	Lutjanus campechanus	Red Snapper	N	Brought on Board (Retained/Discarded)	Live - Stomach and/or Eyes Protruding	Discarded - Live and Healthy (Vented)	Not Applicable	
21	UCAM	Lutjanus campechanus	Red Snapper	N	Brought on Board (Retained/Discarded)	Dead on Arrival - Damaged	Discarded - Dead	Not Applicable	
21	UCAM	Lutjanus campechanus	Red Snapper	N	Brought on Board (Retained/Discarded)	Live - Healthy	Discarded - Live and Healthy (Vented)	Not Applicable	
21	UCAM	Rhizoprionodon terraenovae	Atlantic Sharpnose Shark	Y	Brought on Board (Retained/Discarded)	Live - Healthy	Discarded - Live and Healthy (Not Vented)	Male (Claspers)	Small (Less than 1m)
21	UCAM	Unidentified Bottom Debris	Unidentified Bottom Debris	N	Brought on Board (Retained/Discarded)	Unknown Condition	Discarded - Unknown	Not Applicable	
22	NOCAM	Epinephelus morio	Red Grouper	N	Brought on Board (Retained/Discarded)	Live - Healthy	Discarded - Live and Healthy (Vented)	Not Applicable	
22	NOCAM	Epinephelus morio	Red Grouper	N	Brought on Board (Retained/Discarded)	Live - Stomach and/or Eyes Protruding	Retained	Not Applicable	
22	NOCAM	Epinephelus morio	Red Grouper	N	Brought on Board (Retained/Discarded)	Live - Healthy	Discarded - Live and Healthy (Vented)	Not Applicable	
22	NOCAM	Epinephelus morio	Red Grouper	N	Brought on Board (Retained/Discarded)	Live - Stomach and/or Eyes Protruding	Discarded - Live and Healthy (Vented)	Not Applicable	
22	NOCAM	Epinephelus morio	Red Grouper	N	Brought on Board (Retained/Discarded)	Live - Healthy	Discarded - Live and Healthy (Vented)	Not Applicable	
22	NOCAM	Epinephelus morio	Red Grouper	N	Brought on Board (Retained/Discarded)	Live - Stomach and/or Eyes Protruding	Discarded - Live and Healthy (Vented)	Not Applicable	
22	NOCAM	Epinephelus morio	Red Grouper	N	Brought on Board (Retained/Discarded)	Live - Healthy	Discarded - Live and Healthy (Vented)	Not Applicable	
22	NOCAM	Epinephelus morio	Red Grouper	N	Brought on Board (Retained/Discarded)	Live - Stomach and/or Eyes Protruding	Retained	Not Applicable	
22	NOCAM	Epinephelus morio	Red Grouper	N	Brought on Board (Retained/Discarded)	Live - Healthy	Discarded - Live and Healthy (Vented)	Not Applicable	
22	NOCAM	Epinephelus morio	Red Grouper	N	Brought on Board (Retained/Discarded)	Live - Healthy	Retained	Not Applicable	
22	NOCAM	Epinephelus morio	Red Grouper	N	Brought on Board (Retained/Discarded)	Live - Stomach and/or Eyes Protruding	Discarded - Live and Healthy (Vented)	Not Applicable	
22	NOCAM	Epinephelus morio	Red Grouper	N	Brought on Board (Retained/Discarded)	Dead on Arrival - Undamaged	Discarded - Dead	Not Applicable	
22	NOCAM	Epinephelus morio	Red Grouper	N	Brought on Board (Retained/Discarded)	Live - Stomach and/or Eyes Protruding	Discarded - Live and Healthy (Vented)	Not Applicable	
22	NOCAM	Epinephelus morio	Red Grouper	N	Brought on Board (Retained/Discarded)	Live - Healthy	Retained	Not Applicable	
22	NOCAM	Epinephelus morio	Red Grouper	N	Brought on Board (Retained/Discarded)	Live - Stomach and/or Eyes Protruding	Retained	Not Applicable	
22	NOCAM	Epinephelus morio	Red Grouper	N	Brought on Board (Retained/Discarded)	Live - Stomach and/or Eyes Protruding	Discarded - Live and Healthy (Vented)	Not Applicable	
22	NOCAM	Epinephelus morio	Red Grouper	N	Brought on Board (Retained/Discarded)	Live - Stomach and/or Eyes Protruding	Retained	Not Applicable	
22	NOCAM	Epinephelus morio	Red Grouper	N	Brought on Board (Retained/Discarded)	Live - Stomach and/or Eyes Protruding	Retained	Not Applicable	
22	NOCAM	Epinephelus morio	Red Grouper	N	Brought on Board (Retained/Discarded)	Live - Stomach and/or Eyes Protruding	Discarded - Live and Healthy (Vented)	Not Applicable	
22	NOCAM	Epinephelus morio	Red Grouper	N	Brought on Board (Retained/Discarded)	Live - Healthy	Discarded - Live and Healthy (Vented)	Not Applicable	
22	NOCAM	Epinephelus morio	Red Grouper	N	Brought on Board (Retained/Discarded)	Live - Stomach and/or Eyes Protruding	Retained	Not Applicable	
22	NOCAM	Epinephelus morio	Red Grouper	N	Brought on Board (Retained/Discarded)	Live - Healthy	Retained	Not Applicable	
22	NOCAM	Epinephelus morio	Red Grouper	N	Brought on Board (Retained/Discarded)	Live - Stomach and/or Eyes Protruding	Retained	Not Applicable	
22	NOCAM	Epinephelus morio	Red Grouper	N	Brought on Board (Retained/Discarded)	Live - Stomach and/or Eyes Protruding	Retained	Not Applicable	
22	NOCAM	Epinephelus morio	Red Grouper	N	Brought on Board (Retained/Discarded)	Live - Healthy	Retained	Not Applicable	
22	NOCAM	Epinephelus morio	Red Grouper	N	Brought on Board (Retained/Discarded)	Live - Stomach and/or Eyes Protruding	Retained	Not Applicable	
22	NOCAM	Epinephelus morio	Red Grouper	N	Brought on Board (Retained/Discarded)	Live - Stomach and/or Eyes Protruding	Discarded - Live and Healthy (Vented)	Not Applicable	
22	NOCAM	Epinephelus morio	Red Grouper	N	Brought on Board (Retained/Discarded)	Live - Stomach and/or Eyes Protruding	Discarded - Live and Healthy (Vented)	Not Applicable	
22	NOCAM	Epinephelus morio	Red Grouper	N	Brought on Board (Retained/Discarded)	Live - Healthy	Retained	Not Applicable	
22	NOCAM	Epinephelus morio	Red Grouper	N	Brought on Board (Retained/Discarded)	Live - Healthy	Discarded - Live and Healthy (Vented)	Not Applicable	
22	NOCAM	Epinephelus morio	Red Grouper	N	Brought on Board (Retained/Discarded)	Live - Stomach and/or Eyes Protruding	Retained	Not Applicable	
22	NOCAM	Epinephelus morio	Red Grouper	N	Brought on Board (Retained/Discarded)	Live - Healthy	Retained	Not Applicable	
22	NOCAM	Epinephelus morio	Red Grouper	N	Brought on Board (Retained/Discarded)	Live - Stomach and/or Eyes Protruding	Retained	Not Applicable	
22	NOCAM	Epinephelus morio	Red Grouper	N	Brought on Board (Retained/Discarded)	Live - Stomach and/or Eyes Protruding	Retained	Not Applicable	
22	NOCAM	Epinephelus morio	Red Grouper	N	Brought on Board (Retained/Discarded)	Live - Healthy	Discarded - Live and Healthy (Vented)	Not Applicable	
22	NOCAM	Rhizoprionodon terraenovae	Atlantic Sharpnose Shark	Y	Brought on Board (Retained/Discarded)	Live - Healthy	Discarded - Live and Healthy (Not Vented)	Female	Small (Less than 1m)
22	NOCAM	Rhizoprionodon terraenovae	Atlantic Sharpnose Shark	Y	Brought on Board (Retained/Discarded)	Live - Healthy	Discarded - Live and Healthy (Not Vented)	Female	Small (Less than 1m)
22	NOCAM	Rhizoprionodon terraenovae	Atlantic Sharpnose Shark	Y	Brought on Board (Retained/Discarded)	Live - Healthy	Discarded - Live and Healthy (Not Vented)	Female	Small (Less than 1m)
22	UCAM	Epinephelus morio	Red Grouper	N	Brought on Board (Retained/Discarded)	Live - Healthy	Retained	Not Applicable	
22	UCAM	Epinephelus morio	Red Grouper	N	Brought on Board (Retained/Discarded)	Live - Healthy	Discarded - Live and Healthy (Vented)	Not Applicable	
22	UCAM	Epinephelus morio	Red Grouper	N	Brought on Board (Retained/Discarded)	Live - Healthy	Discarded - Live and Healthy (Vented)	Not Applicable	
22	UCAM	Epinephelus morio	Red Grouper	N	Brought on Board (Retained/Discarded)	Live - Healthy	Discarded - Live and Healthy (Vented)	Not Applicable	
22	UCAM	Epinephelus morio	Red Grouper	N	Brought on Board (Retained/Discarded)	Live - Healthy	Retained	Not Applicable	
22	UCAM	Epinephelus morio	Red Grouper	N	Brought on Board (Retained/Discarded)	Live - Stomach and/or Eyes Protruding	Retained	Not Applicable	
22	UCAM	Epinephelus morio	Red Grouper	N	Brought on Board (Retained/Discarded)	Live - Healthy	Discarded - Live and Healthy (Vented)	Not Applicable	
22	UCAM	Epinephelus morio	Red Grouper	N	Brought on Board (Retained/Discarded)	Live - Healthy	Retained	Not Applicable	
22	UCAM	Epinephelus morio	Red Grouper	N	Brought on Board (Retained/Discarded)	Live - Healthy	Retained	Not Applicable	
22	UCAM	Epinephelus morio	Red Grouper	N	Brought on Board (Retained/Discarded)	Live - Stomach and/or Eyes Protruding	Retained	Not Applicable	
22	UCAM	Epinephelus morio	Red Grouper	N	Brought on Board (Retained/Discarded)	Live - Healthy	Discarded - Live and Healthy (Vented)	Not Applicable	
22	UCAM	Epinephelus morio	Red Grouper	N	Brought on Board (Retained/Discarded)	Live - Healthy	Discarded - Live and Healthy (Vented)	Not Applicable	
22	UCAM	Epinephelus morio	Red Grouper	N	Brought on Board (Retained/Discarded)	Live - Healthy	Retained	Not Applicable	
22	UCAM	Epinephelus morio	Red Grouper	N	Brought on Board (Retained/Discarded)	Live - Stomach and/or Eyes Protruding	Retained	Not Applicable	
22	UCAM	Epinephelus morio	Red Grouper	N	Brought on Board (Retained/Discarded)	Live - Stomach and/or Eyes Protruding	Discarded - Live and Healthy (Vented)	Not Applicable	
22	UCAM	Epinephelus morio	Red Grouper	N	Brought on Board (Retained/Discarded)	Live - Stomach and/or Eyes Protruding	Retained	Not Applicable	
22	UCAM	Epinephelus morio	Red Grouper	N	Brought on Board (Retained/Discarded)	Live - Healthy	Discarded - Live and Healthy (Vented)	Not Applicable	
22	UCAM	Epinephelus morio	Red Grouper	N	Brought on Board (Retained/Discarded)	Live - Stomach and/or Eyes Protruding	Retained	Not Applicable	
22	UCAM	Epinephelus morio	Red Grouper	N	Brought on Board (Retained/Discarded)	Live - Healthy	Retained	Not Applicable	
22	UCAM	Epinephelus morio	Red Grouper	N	Brought on Board (Retained/Discarded)	Live - Healthy	Discarded - Live and Healthy (Vented)	Not Applicable	
22	UCAM	Epinephelus morio	Red Grouper	N	Brought on Board (Retained/Discarded)	Live - Healthy	Discarded - Live and Healthy (Vented)	Not Applicable	
22	UCAM	Epinephelus morio	Red Grouper	N	Brought on Board (Retained/Discarded)	Live - Healthy	Retained	Not Applicable	
22	UCAM	Epinephelus morio	Red Grouper	N	Brought on Board (Retained/Discarded)	Live - Stomach and/or Eyes Protruding	Retained	Not Applicable	
22	UCAM	Epinephelus morio	Red Grouper	N	Brought on Board (Retained/Discarded)	Live - Healthy	Retained	Not Applicable	
22	UCAM	Epinephelus morio	Red Grouper	N	Brought on Board (Retained/Discarded)	Live - Healthy	Retained	Not Applicable	
22	UCAM	Epinephelus morio	Red Grouper	N	Brought on Board (Retained/Discarded)	Live - Stomach and/or Eyes Protruding	Discarded - Live and Healthy (Vented)	Not Applicable	
22	UCAM	Epinephelus morio	Red Grouper	N	Brought on Board (Retained/Discarded)	Live - Stomach and/or Eyes Protruding	Discarded - Live and Healthy (Vented)	Not Applicable	
22	UCAM	Epinephelus morio	Red Grouper	N	Brought on Board (Retained/Discarded)	Live - Stomach and/or Eyes Protruding	Retained	Not Applicable	
22	UCAM	Epinephelus morio	Red Grouper	N	Brought on Board (Retained/Discarded)	Live - Healthy	Retained	Not Applicable	
22	UCAM	Epinephelus morio	Red Grouper	N	Brought on Board (Retained/Discarded)	Live - Stomach and/or Eyes Protruding	Retained	Not Applicable	
22	UCAM	Epinephelus morio	Red Grouper	N	Brought on Board (Retained/Discarded)	Live - Healthy	Retained	Not Applicable	
22	UCAM	Epinephelus morio	Red Grouper	N	Brought on Board (Retained/Discarded)	Live - Healthy	Discarded - Live and Healthy (Vented)	Not Applicable	
22	UCAM	Epinephelus morio	Red Grouper	N	Brought on Board (Retained/Discarded)	Live - Healthy	Discarded - Live and Healthy (Vented)	Not Applicable	
22	UCAM	Epinephelus morio	Red Grouper	N	Brought on Board (Retained/Discarded)	Live - Healthy	Discarded - Live and Healthy (Vented)	Not Applicable	
22	UCAM	Rhizoprionodon terraenovae	Atlantic Sharpnose Shark	Y	Brought on Board (Retained/Discarded)	Live - Healthy	Discarded - Live and Healthy (Not Vented)	Female	Small (Less than 1m)
22	UCAM	Rhizoprionodon terraenovae	Atlantic Sharpnose Shark	Y	Brought on Board (Retained/Discarded)	Live - Healthy	Discarded - Live and Healthy (Not Vented)	Female	Small (Less than 1m)
22	UCAM	Rhizoprionodon terraenovae	Atlantic Sharpnose Shark	Y	Brought on Board (Retained/Discarded)	Live - Healthy	Discarded - Live and Healthy (Not Vented)	Female	Small (Less than 1m)
23	NOCAM	Carcharhinus plumbeus	Sandbar Shark	Y	Cut-off at Rail (No Entanglement)	Live - Healthy	Discarded - Live and Healthy (Not Vented)	Known Adult - Undetermined Sex	Large (Greater than 2m)
23	NOCAM	Carcharhinus plumbeus	Sandbar Shark	Y	Cut-off at Rail (No Entanglement)	Live - Healthy	Discarded - Live and Healthy (Not Vented)	Known Adult - Undetermined Sex	Large (Greater than 2m)

23	NOCAM	Carcharhinus plumbeus	Sandbar Shark	Y	Cut-off at Rail (No Entanglement)	Live - Healthy	Discarded - Live and Healthy (Not Vented)	Known Adult - Undetermined Sex	Large (Greater than 2m)
23	NOCAM	Epinephelus morio	Red Grouper	N	Brought on Board (Retained/Discarded)	Live - Healthy	Discarded - Live and Healthy (Vented)	Not Applicable	
23	NOCAM	Epinephelus morio	Red Grouper	N	Brought on Board (Retained/Discarded)	Live - Healthy	Retained	Not Applicable	
23	NOCAM	Epinephelus morio	Red Grouper	N	Brought on Board (Retained/Discarded)	Live - Healthy	Retained	Not Applicable	
23	NOCAM	Epinephelus morio	Red Grouper	N	Brought on Board (Retained/Discarded)	Live - Healthy	Retained	Not Applicable	
23	NOCAM	Epinephelus morio	Red Grouper	N	Brought on Board (Retained/Discarded)	Dead on Arrival - Damaged	Discarded - Dead	Not Applicable	
23	NOCAM	Epinephelus morio	Red Grouper	N	Brought on Board (Retained/Discarded)	Live - Stomach and/or Eyes Protruding	Retained	Not Applicable	
23	NOCAM	Epinephelus morio	Red Grouper	N	Brought on Board (Retained/Discarded)	Live - Healthy	Retained	Not Applicable	
23	NOCAM	Epinephelus morio	Red Grouper	N	Brought on Board (Retained/Discarded)	Live - Healthy	Retained	Not Applicable	
23	NOCAM	Epinephelus morio	Red Grouper	N	Brought on Board (Retained/Discarded)	Live - Healthy	Retained	Not Applicable	
23	NOCAM	Epinephelus morio	Red Grouper	N	Brought on Board (Retained/Discarded)	Live - Healthy	Retained	Not Applicable	
23	NOCAM	Epinephelus morio	Red Grouper	N	Brought on Board (Retained/Discarded)	Live - Healthy	Discarded - Live and Healthy (Vented)	Not Applicable	
23	NOCAM	Epinephelus morio	Red Grouper	N	Brought on Board (Retained/Discarded)	Live - Healthy	Retained	Not Applicable	
23	NOCAM	Epinephelus morio	Red Grouper	N	Brought on Board (Retained/Discarded)	Live - Healthy	Discarded - Live and Healthy (Vented)	Not Applicable	
23	NOCAM	Epinephelus morio	Red Grouper	N	Brought on Board (Retained/Discarded)	Live - Healthy	Retained	Not Applicable	
23	NOCAM	Epinephelus morio	Red Grouper	N	Brought on Board (Retained/Discarded)	Live - Healthy	Retained	Not Applicable	
23	NOCAM	Epinephelus morio	Red Grouper	N	Brought on Board (Retained/Discarded)	Live - Healthy	Discarded - Live and Healthy (Vented)	Not Applicable	
23	NOCAM	Galeocerdo cuvier	Tiger Shark	Y	Cut-off at Rail (No Entanglement)	Live - Healthy	Discarded - Live and Healthy (Not Vented)	Unknown Maturity and/or Sex	Large (Greater than 2m)
23	NOCAM	Lutjanus campechanus	Red Snapper	N	Brought on Board (Retained/Discarded)	Live - Healthy	Discarded - Live and Healthy (Not Vented)	Not Applicable	
23	UCAM	Carcharhinus plumbeus	Sandbar Shark	Y	Cut-off at Rail (No Entanglement)	Live - Healthy	Discarded - Live and Healthy (Not Vented)	Male (Claspers)	Large (Greater than 2m)
23	UCAM	Carcharhinus plumbeus	Sandbar Shark	Y	Cut-off at Rail (No Entanglement)	Live - Healthy	Discarded - Live and Healthy (Not Vented)	Female	Large (Greater than 2m)
23	UCAM	Carcharhinus plumbeus	Sandbar Shark	Y	Cut-off at Rail (No Entanglement)	Live - Healthy	Discarded - Live and Healthy (Not Vented)	Male (Claspers)	Large (Greater than 2m)
23	UCAM	Carcharhinus plumbeus	Sandbar Shark	Y	Cut-off at Rail (No Entanglement)	Live - Healthy	Discarded - Live and Healthy (Not Vented)	Known Adult - Undetermined Sex	Large (Greater than 2m)
23	UCAM	Carcharhinus plumbeus	Sandbar Shark	Y	Cut-off at Rail (No Entanglement)	Live - Healthy	Discarded - Live and Healthy (Not Vented)	Male (Claspers)	Large (Greater than 2m)
23	UCAM	Epinephelus morio	Red Grouper	N	Brought on Board (Retained/Discarded)	Live - Stomach and/or Eyes Protruding	Retained	Not Applicable	
23	UCAM	Epinephelus morio	Red Grouper	N	Brought on Board (Retained/Discarded)	Live - Stomach and/or Eyes Protruding	Retained	Not Applicable	
23	UCAM	Epinephelus morio	Red Grouper	N	Brought on Board (Retained/Discarded)	Live - Healthy	Discarded - Live and Healthy (Vented)	Not Applicable	
23	UCAM	Epinephelus morio	Red Grouper	N	Brought on Board (Retained/Discarded)	Live - Stomach and/or Eyes Protruding	Retained	Not Applicable	
23	UCAM	Epinephelus morio	Red Grouper	N	Brought on Board (Retained/Discarded)	Live - Healthy	Discarded - Live and Healthy (Vented)	Not Applicable	
23	UCAM	Epinephelus morio	Red Grouper	N	Brought on Board (Retained/Discarded)	Live - Stomach and/or Eyes Protruding	Retained	Not Applicable	
23	UCAM	Epinephelus morio	Red Grouper	N	Brought on Board (Retained/Discarded)	Live - Stomach and/or Eyes Protruding	Retained	Not Applicable	
23	UCAM	Epinephelus morio	Red Grouper	N	Brought on Board (Retained/Discarded)	Live - Healthy	Discarded - Live and Healthy (Vented)	Not Applicable	
23	UCAM	Epinephelus morio	Red Grouper	N	Brought on Board (Retained/Discarded)	Live - Healthy	Retained	Not Applicable	
23	UCAM	Epinephelus morio	Red Grouper	N	Brought on Board (Retained/Discarded)	Live - Healthy	Retained	Not Applicable	
23	UCAM	Epinephelus morio	Red Grouper	N	Brought on Board (Retained/Discarded)	Live - Healthy	Retained	Not Applicable	
23	UCAM	Epinephelus morio	Red Grouper	N	Brought on Board (Retained/Discarded)	Live - Healthy	Discarded - Live and Healthy (Vented)	Not Applicable	
23	UCAM	Galeocerdo cuvier	Tiger Shark	Y	Cut-off at Rail (No Entanglement)	Live - Healthy	Discarded - Live and Healthy (Not Vented)	Female	Large (Greater than 2m)
23	UCAM	Lutjanus campechanus	Red Snapper	N	Brought on Board (Retained/Discarded)	Live - Healthy	Discarded - Live and Healthy (Vented)	Not Applicable	
24	NOCAM	Carcharhinus plumbeus	Sandbar Shark	Y	Cut-off at Rail (No Entanglement)	Live - Healthy	Discarded - Live and Healthy (Not Vented)	Male (Claspers)	Large (Greater than 2m)
24	NOCAM	Epinephelus morio	Red Grouper	N	Brought on Board (Retained/Discarded)	Live - Stomach and/or Eyes Protruding	Retained	Not Applicable	
24	NOCAM	Epinephelus morio	Red Grouper	N	Brought on Board (Retained/Discarded)	Live - Healthy	Discarded - Live and Healthy (Vented)	Not Applicable	
24	NOCAM	Epinephelus morio	Red Grouper	N	Brought on Board (Retained/Discarded)	Live - Healthy	Retained	Not Applicable	
24	NOCAM	Epinephelus morio	Red Grouper	N	Brought on Board (Retained/Discarded)	Live - Stomach and/or Eyes Protruding	Retained	Not Applicable	
24	NOCAM	Epinephelus morio	Red Grouper	N	Brought on Board (Retained/Discarded)	Live - Healthy	Discarded - Live and Healthy (Vented)	Not Applicable	
24	NOCAM	Epinephelus morio	Red Grouper	N	Brought on Board (Retained/Discarded)	Dead on Arrival - Undamaged	Retained	Not Applicable	
24	NOCAM	Epinephelus morio	Red Grouper	N	Brought on Board (Retained/Discarded)	Live - Healthy	Discarded - Live and Healthy (Vented)	Not Applicable	
24	NOCAM	Epinephelus morio	Red Grouper	N	Brought on Board (Retained/Discarded)	Live - Healthy	Discarded - Live and Healthy (Vented)	Not Applicable	
24	NOCAM	Epinephelus morio	Red Grouper	N	Brought on Board (Retained/Discarded)	Live - Healthy	Retained	Not Applicable	
24	NOCAM	Epinephelus morio	Red Grouper	N	Brought on Board (Retained/Discarded)	Live - Healthy	Discarded - Live and Healthy (Vented)	Not Applicable	
24	NOCAM	Epinephelus morio	Red Grouper	N	Brought on Board (Retained/Discarded)	Live - Healthy	Discarded - Live and Healthy (Vented)	Not Applicable	
24	NOCAM	Epinephelus morio	Red Grouper	N	Brought on Board (Retained/Discarded)	Live - Healthy	Retained	Not Applicable	
24	NOCAM	Epinephelus morio	Red Grouper	N	Brought on Board (Retained/Discarded)	Live - Stomach and/or Eyes Protruding	Retained	Not Applicable	
24	NOCAM	Epinephelus morio	Red Grouper	N	Brought on Board (Retained/Discarded)	Live - Healthy	Retained	Not Applicable	
24	NOCAM	Epinephelus morio	Red Grouper	N	Brought on Board (Retained/Discarded)	Live - Stomach and/or Eyes Protruding	Retained	Not Applicable	
24	NOCAM	Epinephelus morio	Red Grouper	N	Brought on Board (Retained/Discarded)	Live - Healthy	Discarded - Live and Healthy (Vented)	Not Applicable	
24	NOCAM	Epinephelus morio	Red Grouper	N	Brought on Board (Retained/Discarded)	Live - Stomach and/or Eyes Protruding	Retained	Not Applicable	
24	NOCAM	Epinephelus morio	Red Grouper	N	Brought on Board (Retained/Discarded)	Live - Healthy	Retained	Not Applicable	
24	NOCAM	Epinephelus morio	Red Grouper	N	Brought on Board (Retained/Discarded)	Live - Healthy	Discarded - Live and Healthy (Vented)	Not Applicable	
24	NOCAM	Epinephelus morio	Red Grouper	N	Brought on Board (Retained/Discarded)	Live - Healthy	Discarded - Live and Healthy (Vented)	Not Applicable	
24	NOCAM	Epinephelus morio	Red Grouper	N	Brought on Board (Retained/Discarded)	Live - Healthy	Retained	Not Applicable	
24	NOCAM	Epinephelus morio	Red Grouper	N	Brought on Board (Retained/Discarded)	Live - Stomach and/or Eyes Protruding	Discarded - Live and Healthy (Vented)	Not Applicable	
24	NOCAM	Epinephelus morio	Red Grouper	N	Brought on Board (Retained/Discarded)	Live - Healthy	Discarded - Live and Healthy (Vented)	Not Applicable	
24	NOCAM	Epinephelus morio	Red Grouper	N	Brought on Board (Retained/Discarded)	Live - Stomach and/or Eyes Protruding	Retained	Not Applicable	
24	NOCAM	Epinephelus morio	Red Grouper	N	Brought on Board (Retained/Discarded)	Live - Healthy	Retained	Not Applicable	
24	NOCAM	Epinephelus morio	Red Grouper	N	Brought on Board (Retained/Discarded)	Live - Stomach and/or Eyes Protruding	Retained	Not Applicable	
24	NOCAM	Lutjanus campechanus	Red Snapper	N	Brought on Board (Retained/Discarded)	Live - Healthy	Discarded - Live and Healthy (Vented)	Not Applicable	
24	UCAM	Carcharhinus plumbeus	Sandbar Shark	Y	Cut-off at Rail (No Entanglement)	Live - Healthy	Discarded - Live and Healthy (Not Vented)	Male (Claspers)	Large (Greater than 2m)
24	UCAM	Epinephelus morio	Red Grouper	N	Brought on Board (Retained/Discarded)	Live - Stomach and/or Eyes Protruding	Discarded - Live and Healthy (Vented)	Not Applicable	
24	UCAM	Epinephelus morio	Red Grouper	N	Brought on Board (Retained/Discarded)	Live - Stomach and/or Eyes Protruding	Retained	Not Applicable	
24	UCAM	Epinephelus morio	Red Grouper	N	Brought on Board (Retained/Discarded)	Live - Stomach and/or Eyes Protruding	Retained	Not Applicable	
24	UCAM	Epinephelus morio	Red Grouper	N	Brought on Board (Retained/Discarded)	Live - Stomach and/or Eyes Protruding	Retained	Not Applicable	
24	UCAM	Epinephelus morio	Red Grouper	N	Brought on Board (Retained/Discarded)	Live - Stomach and/or Eyes Protruding	Retained	Not Applicable	
24	UCAM	Epinephelus morio	Red Grouper	N	Brought on Board (Retained/Discarded)	Live - Stomach and/or Eyes Protruding	Discarded - Live and Healthy (Vented)	Not Applicable	
24	UCAM	Epinephelus morio	Red Grouper	N	Brought on Board (Retained/Discarded)	Live - Stomach and/or Eyes Protruding	Retained	Not Applicable	
24	UCAM	Epinephelus morio	Red Grouper	N	Brought on Board (Retained/Discarded)	Live - Stomach and/or Eyes Protruding	Retained	Not Applicable	
24	UCAM	Epinephelus morio	Red Grouper	N	Brought on Board (Retained/Discarded)	Live - Healthy	Retained	Not Applicable	
24	UCAM	Epinephelus morio	Red Grouper	N	Brought on Board (Retained/Discarded)	Live - Healthy	Discarded - Live and Healthy (Vented)	Not Applicable	
24	UCAM	Epinephelus morio	Red Grouper	N	Brought on Board (Retained/Discarded)	Live - Healthy	Retained	Not Applicable	
24	UCAM	Epinephelus morio	Red Grouper	N	Brought on Board (Retained/Discarded)	Live - Stomach and/or Eyes Protruding	Discarded - Live and Healthy (Vented)	Not Applicable	
24	UCAM	Epinephelus morio	Red Grouper	N	Brought on Board (Retained/Discarded)	Live - Healthy	Retained	Not Applicable	
24	UCAM	Epinephelus morio	Red Grouper	N	Brought on Board (Retained/Discarded)	Live - Healthy	Discarded - Live and Healthy (Vented)	Not Applicable	
24	UCAM	Epinephelus morio	Red Grouper	N	Brought on Board (Retained/Discarded)	Live - Stomach and/or Eyes Protruding	Retained	Not Applicable	
24	UCAM	Epinephelus morio	Red Grouper	N	Brought on Board (Retained/Discarded)	Live - Healthy	Discarded - Live and Healthy (Vented)	Not Applicable	
24	UCAM	Epinephelus morio	Red Grouper	N	Brought on Board (Retained/Discarded)	Live - Healthy	Discarded - Live and Healthy (Vented)	Not Applicable	
24	UCAM	Epinephelus morio	Red Grouper	N	Brought on Board (Retained/Discarded)	Live - Healthy	Discarded - Live and Healthy (Vented)	Not Applicable	
24	UCAM	Epinephelus morio	Red Grouper	N	Brought on Board (Retained/Discarded)	Live - Stomach and/or Eyes Protruding	Retained	Not Applicable	
24	UCAM	Epinephelus morio	Red Grouper	N	Brought on Board (Retained/Discarded)	Dead on Arrival - Undamaged	Retained	Not Applicable	
24	UCAM	Epinephelus morio	Red Grouper	N	Brought on Board (Retained/Discarded)	Live - Healthy	Discarded - Live and Healthy (Vented)	Not Applicable	
24	UCAM	Epinephelus morio	Red Grouper	N	Brought on Board (Retained/Discarded)	Live - Healthy	Retained	Not Applicable	
24	UCAM	Epinephelus morio	Red Grouper	N	Brought on Board (Retained/Discarded)	Live - Stomach and/or Eyes Protruding	Retained	Not Applicable	
24	UCAM	Epinephelus morio	Red Grouper	N	Brought on Board (Retained/Discarded)	Live - Healthy	Retained	Not Applicable	
24	UCAM	Lutjanus campechanus	Red Snapper	N	Brought on Board (Retained/Discarded)	Live - Healthy	Discarded - Live and Healthy (Vented)	Not Applicable	

25	NOCAM	Carcharhinus acronotus	Blacknose Shark	Y	Cut-off at Rail (No Entanglement)	Live - Healthy	Discarded - Live and Healthy (Not Vented)	Unknown Maturity and/or Sex	Medium (Between 1m and 2m)
25	NOCAM	Carcharhinus plumbeus	Sandbar Shark	Y	Cut-off at Rail (No Entanglement)	Live - Healthy	Discarded - Live and Healthy (Not Vented)	Known Adult - Undetermined Sex	Large (Greater than 2m)
25	NOCAM	Epinephelus morio	Red Grouper	N	Brought on Board (Retained/Discarded)	Live - Healthy	Discarded - Live and Healthy (Vented)	Not Applicable	
25	NOCAM	Epinephelus morio	Red Grouper	N	Brought on Board (Retained/Discarded)	Live - Healthy	Discarded - Live and Healthy (Vented)	Not Applicable	
25	NOCAM	Epinephelus morio	Red Grouper	N	Brought on Board (Retained/Discarded)	Live - Stomach and/or Eyes Protruding	Retained	Not Applicable	
25	NOCAM	Epinephelus morio	Red Grouper	N	Brought on Board (Retained/Discarded)	Live - Healthy	Retained	Not Applicable	
25	NOCAM	Epinephelus morio	Red Grouper	N	Brought on Board (Retained/Discarded)	Live - Healthy	Discarded - Live and Healthy (Vented)	Not Applicable	
25	NOCAM	Epinephelus morio	Red Grouper	N	Brought on Board (Retained/Discarded)	Live - Healthy	Retained	Not Applicable	
25	NOCAM	Epinephelus morio	Red Grouper	N	Brought on Board (Retained/Discarded)	Live - Healthy	Discarded - Live and Healthy (Vented)	Not Applicable	
25	NOCAM	Epinephelus morio	Red Grouper	N	Brought on Board (Retained/Discarded)	Live - Healthy	Discarded - Live and Healthy (Vented)	Not Applicable	
25	NOCAM	Epinephelus morio	Red Grouper	N	Brought on Board (Retained/Discarded)	Live - Healthy	Retained	Not Applicable	
25	NOCAM	Epinephelus morio	Red Grouper	N	Brought on Board (Retained/Discarded)	Live - Healthy	Discarded - Live and Healthy (Vented)	Not Applicable	
25	NOCAM	Epinephelus morio	Red Grouper	N	Brought on Board (Retained/Discarded)	Live - Healthy	Discarded - Live and Healthy (Vented)	Not Applicable	
25	NOCAM	Epinephelus morio	Red Grouper	N	Brought on Board (Retained/Discarded)	Live - Healthy	Retained	Not Applicable	
25	NOCAM	Epinephelus morio	Red Grouper	N	Brought on Board (Retained/Discarded)	Live - Healthy	Discarded - Live and Healthy (Vented)	Not Applicable	
25	NOCAM	Epinephelus morio	Red Grouper	N	Brought on Board (Retained/Discarded)	Live - Healthy	Retained	Not Applicable	
25	NOCAM	Epinephelus morio	Red Grouper	N	Brought on Board (Retained/Discarded)	Live - Healthy	Discarded - Live and Healthy (Vented)	Not Applicable	
25	NOCAM	Epinephelus morio	Red Grouper	N	Brought on Board (Retained/Discarded)	Live - Healthy	Discarded - Live and Healthy (Vented)	Not Applicable	
25	NOCAM	Epinephelus morio	Red Grouper	N	Brought on Board (Retained/Discarded)	Live - Healthy	Retained	Not Applicable	
25	NOCAM	Epinephelus morio	Red Grouper	N	Brought on Board (Retained/Discarded)	Live - Healthy	Discarded - Live and Healthy (Vented)	Not Applicable	
25	NOCAM	Epinephelus morio	Red Grouper	N	Brought on Board (Retained/Discarded)	Live - Healthy	Retained	Not Applicable	
25	NOCAM	Epinephelus morio	Red Grouper	N	Brought on Board (Retained/Discarded)	Live - Healthy	Retained	Not Applicable	
25	NOCAM	Epinephelus morio	Red Grouper	N	Brought on Board (Retained/Discarded)	Live - Healthy	Retained	Not Applicable	
25	NOCAM	Epinephelus morio	Red Grouper	N	Brought on Board (Retained/Discarded)	Live - Healthy	Retained	Not Applicable	
25	NOCAM	Epinephelus morio	Red Grouper	N	Brought on Board (Retained/Discarded)	Live - Healthy	Retained	Not Applicable	
25	NOCAM	Epinephelus morio	Red Grouper	N	Brought on Board (Retained/Discarded)	Live - Stomach and/or Eyes Protruding	Retained	Not Applicable	
25	NOCAM	Epinephelus morio	Red Grouper	N	Brought on Board (Retained/Discarded)	Live - Healthy	Retained	Not Applicable	
25	NOCAM	Epinephelus morio	Red Grouper	N	Brought on Board (Retained/Discarded)	Live - Stomach and/or Eyes Protruding	Retained	Not Applicable	
25	NOCAM	Epinephelus morio	Red Grouper	N	Brought on Board (Retained/Discarded)	Live - Healthy	Discarded - Live and Healthy (Vented)	Not Applicable	
25	NOCAM	Epinephelus morio	Red Grouper	N	Brought on Board (Retained/Discarded)	Live - Stomach and/or Eyes Protruding	Retained	Not Applicable	
25	NOCAM	Epinephelus morio	Red Grouper	N	Brought on Board (Retained/Discarded)	Live - Healthy	Retained	Not Applicable	
25	NOCAM	Epinephelus morio	Red Grouper	N	Brought on Board (Retained/Discarded)	Live - Healthy	Discarded - Live and Healthy (Vented)	Not Applicable	
25	NOCAM	Epinephelus morio	Red Grouper	N	Brought on Board (Retained/Discarded)	Live - Stomach and/or Eyes Protruding	Retained	Not Applicable	
25	NOCAM	Galeocerdo cuvier	Tiger Shark	Y	Cut-off at Rail (No Entanglement)	Live - Healthy	Discarded - Live and Healthy (Not Vented)	Known Juvenile	Medium (Between 1m and 2m)
25	NOCAM	Lutjanus campechanus	Red Snapper	N	Brought on Board (Retained/Discarded)	Live - Healthy	Discarded - Live and Healthy (Vented)	Not Applicable	
25	NOCAM	Seriola dumerili	Greater Amberjack	N	Brought on Board (Retained/Discarded)	Live - Healthy	Discarded - Live and Healthy (Not Vented)	Not Applicable	
25	UCAM	Carcharhinus leucas	Bull Shark	Y	Cut-off at Rail (No Entanglement)	Live - Healthy	Discarded - Live and Healthy (Not Vented)	Female	Large (Greater than 2m)
25	UCAM	Epinephelus morio	Red Grouper	N	Brought on Board (Retained/Discarded)	Live - Stomach and/or Eyes Protruding	Retained	Not Applicable	
25	UCAM	Epinephelus morio	Red Grouper	N	Brought on Board (Retained/Discarded)	Live - Healthy	Discarded - Live and Healthy (Vented)	Not Applicable	
25	UCAM	Epinephelus morio	Red Grouper	N	Brought on Board (Retained/Discarded)	Live - Healthy	Retained	Not Applicable	
25	UCAM	Epinephelus morio	Red Grouper	N	Brought on Board (Retained/Discarded)	Live - Healthy	Retained	Not Applicable	
25	UCAM	Epinephelus morio	Red Grouper	N	Brought on Board (Retained/Discarded)	Live - Healthy	Discarded - Live and Healthy (Vented)	Not Applicable	
25	UCAM	Epinephelus morio	Red Grouper	N	Brought on Board (Retained/Discarded)	Live - Stomach and/or Eyes Protruding	Retained	Not Applicable	
25	UCAM	Epinephelus morio	Red Grouper	N	Brought on Board (Retained/Discarded)	Live - Stomach and/or Eyes Protruding	Retained	Not Applicable	
25	UCAM	Epinephelus morio	Red Grouper	N	Brought on Board (Retained/Discarded)	Live - Healthy	Retained	Not Applicable	
25	UCAM	Epinephelus morio	Red Grouper	N	Brought on Board (Retained/Discarded)	Live - Stomach and/or Eyes Protruding	Discarded - Live and Healthy (Vented)	Not Applicable	
25	UCAM	Epinephelus morio	Red Grouper	N	Brought on Board (Retained/Discarded)	Live - Healthy	Discarded - Live and Healthy (Vented)	Not Applicable	
25	UCAM	Epinephelus morio	Red Grouper	N	Brought on Board (Retained/Discarded)	Live - Healthy	Retained	Not Applicable	
25	UCAM	Epinephelus morio	Red Grouper	N	Brought on Board (Retained/Discarded)	Live - Healthy	Discarded - Live and Healthy (Vented)	Not Applicable	
25	UCAM	Epinephelus morio	Red Grouper	N	Brought on Board (Retained/Discarded)	Live - Healthy	Retained	Not Applicable	
25	UCAM	Epinephelus morio	Red Grouper	N	Brought on Board (Retained/Discarded)	Live - Healthy	Discarded - Live and Healthy (Vented)	Not Applicable	
25	UCAM	Epinephelus morio	Red Grouper	N	Brought on Board (Retained/Discarded)	Live - Stomach and/or Eyes Protruding	Retained	Not Applicable	
25	UCAM	Epinephelus morio	Red Grouper	N	Brought on Board (Retained/Discarded)	Live - Stomach and/or Eyes Protruding	Discarded - Live and Healthy (Vented)	Not Applicable	
25	UCAM	Epinephelus morio	Red Grouper	N	Brought on Board (Retained/Discarded)	Live - Healthy	Retained	Not Applicable	
25	UCAM	Epinephelus morio	Red Grouper	N	Brought on Board (Retained/Discarded)	Live - Stomach and/or Eyes Protruding	Discarded - Live and Healthy (Vented)	Not Applicable	
25	UCAM	Epinephelus morio	Red Grouper	N	Brought on Board (Retained/Discarded)	Live - Stomach and/or Eyes Protruding	Retained	Not Applicable	
25	UCAM	Epinephelus morio	Red Grouper	N	Brought on Board (Retained/Discarded)	Live - Healthy	Retained	Not Applicable	
25	UCAM	Epinephelus morio	Red Grouper	N	Brought on Board (Retained/Discarded)	Live - Stomach and/or Eyes Protruding	Retained	Not Applicable	
25	UCAM	Epinephelus morio	Red Grouper	N	Brought on Board (Retained/Discarded)	Live - Healthy	Retained	Not Applicable	
25	UCAM	Epinephelus morio	Red Grouper	N	Brought on Board (Retained/Discarded)	Live - Stomach and/or Eyes Protruding	Discarded - Live and Healthy (Vented)	Not Applicable	
25	UCAM	Epinephelus morio	Red Grouper	N	Brought on Board (Retained/Discarded)	Live - Healthy	Retained	Not Applicable	
25	UCAM	Epinephelus morio	Red Grouper	N	Brought on Board (Retained/Discarded)	Live - Healthy	Discarded - Live and Healthy (Vented)	Not Applicable	
25	UCAM	Epinephelus morio	Red Grouper	N	Brought on Board (Retained/Discarded)	Live - Stomach and/or Eyes Protruding	Discarded - Live and Healthy (Vented)	Not Applicable	
25	UCAM	Epinephelus morio	Red Grouper	N	Brought on Board (Retained/Discarded)	Live - Healthy	Discarded - Live and Healthy (Vented)	Not Applicable	
25	UCAM	Epinephelus morio	Red Grouper	N	Brought on Board (Retained/Discarded)	Live - Healthy	Retained	Not Applicable	
25	UCAM	Epinephelus morio	Red Grouper	N	Brought on Board (Retained/Discarded)	Live - Healthy	Discarded - Live and Healthy (Vented)	Not Applicable	
25	UCAM	Epinephelus morio	Red Grouper	N	Brought on Board (Retained/Discarded)	Live - Healthy	Retained	Not Applicable	
25	UCAM	Epinephelus morio	Red Grouper	N	Brought on Board (Retained/Discarded)	Live - Stomach and/or Eyes Protruding	Discarded - Live and Healthy (Vented)	Not Applicable	
25	UCAM	Epinephelus morio	Red Grouper	N	Brought on Board (Retained/Discarded)	Live - Healthy	Retained	Not Applicable	
25	UCAM	Epinephelus morio	Red Grouper	N	Brought on Board (Retained/Discarded)	Live - Healthy	Retained	Not Applicable	
25	UCAM	Galeocerdo cuvier	Tiger Shark	Y	Cut-off at Rail (No Entanglement)	Live - Healthy	Discarded - Live and Healthy (Not Vented)	Known Juvenile	Medium (Between 1m and 2m)
25	UCAM	Lutjanus campechanus	Red Snapper	N	Brought on Board (Retained/Discarded)	Live - Healthy	Discarded - Live and Healthy (Vented)	Not Applicable	
25	UCAM	Seriola dumerili	Greater Amberjack	N	Brought on Board (Retained/Discarded)	Live - Healthy	Discarded - Live and Healthy (Not Vented)	Not Applicable	
26	NOCAM	Carcharhinus plumbeus	Sandbar Shark	Y	Cut-off at Rail (No Entanglement)	Live - Healthy	Discarded - Live and Healthy (Not Vented)	Male (Claspers)	Large (Greater than 2m)
26	NOCAM	Carcharhinus plumbeus	Sandbar Shark	Y	Cut-off at Rail (No Entanglement)	Live - Healthy	Discarded - Live and Healthy (Not Vented)	Known Adult - Undetermined Sex	Large (Greater than 2m)
26	NOCAM	Carcharhinus plumbeus	Sandbar Shark	Y	Cut-off at Rail (No Entanglement)	Live - Healthy	Discarded - Live and Healthy (Not Vented)	Male (Claspers)	Large (Greater than 2m)
26	NOCAM	Crab, unidentified	Crab, Unidentified	N	Brought on Board (Retained/Discarded)	Live - Healthy	Discarded - Live and Healthy (Not Vented)	Not Applicable	
26	NOCAM	Epinephelus morio	Red Grouper	N	Brought on Board (Retained/Discarded)	Live - Healthy	Retained	Not Applicable	
26	NOCAM	Epinephelus morio	Red Grouper	N	Brought on Board (Retained/Discarded)	Live - Healthy	Retained	Not Applicable	
26	NOCAM	Epinephelus morio	Red Grouper	N	Brought on Board (Retained/Discarded)	Dead on Arrival - Undamaged	Retained	Not Applicable	
26	NOCAM	Epinephelus morio	Red Grouper	N	Brought on Board (Retained/Discarded)	Live - Healthy	Discarded - Live and Healthy (Vented)	Not Applicable	
26	NOCAM	Epinephelus morio	Red Grouper	N	Brought on Board (Retained/Discarded)	Live - Healthy	Discarded - Live and Healthy (Vented)	Not Applicable	
26	NOCAM	Epinephelus morio	Red Grouper	N	Not Handled (Dropped Off)	Live - Healthy	Discarded - Live and Healthy (Not Vented)	Not Applicable	
26	NOCAM	Epinephelus morio	Red Grouper	N	Brought on Board (Retained/Discarded)	Live - Healthy	Retained	Not Applicable	
26	NOCAM	Epinephelus morio	Red Grouper	N	Brought on Board (Retained/Discarded)	Live - Healthy	Retained	Not Applicable	
26	NOCAM	Epinephelus morio	Red Grouper	N	Brought on Board (Retained/Discarded)	Live - Healthy	Discarded - Live and Healthy (Vented)	Not Applicable	
26	NOCAM	Epinephelus morio	Red Grouper	N	Brought on Board (Retained/Discarded)	Live - Healthy	Discarded - Live and Healthy (Vented)	Not Applicable	
26	NOCAM	Epinephelus morio	Red Grouper	N	Brought on Board (Retained/Discarded)	Live - Healthy	Discarded - Live and Healthy (Vented)	Not Applicable	
26	NOCAM	Epinephelus morio	Red Grouper	N	Brought on Board (Retained/Discarded)	Live - Healthy	Discarded - Live and Healthy (Vented)	Not Applicable	
26	NOCAM	Epinephelus morio	Red Grouper	N	Brought on Board (Retained/Discarded)	Live - Healthy	Discarded - Live and Healthy (Vented)	Not Applicable	
26	NOCAM	Epinephelus morio	Red Grouper	N	Brought on Board (Retained/Discarded)	Dead on Arrival - Undamaged	Discarded - Dead	Not Applicable	
26	NOCAM	Epinephelus morio	Red Grouper	N	Brought on Board (Retained/Discarded)	Live - Stomach and/or Eyes Protruding	Retained	Not Applicable	

26	NOCAM	Epinephelus morio	Red Grouper	N	Brought on Board (Retained/Discarded)	Live - Stomach and/or Eyes Protruding	Retained	Not Applicable	
26	NOCAM	Epinephelus morio	Red Grouper	N	Brought on Board (Retained/Discarded)	Live - Healthy	Discarded - Live and Healthy (Vented)	Not Applicable	
26	NOCAM	Epinephelus morio	Red Grouper	N	Brought on Board (Retained/Discarded)	Live - Healthy	Retained	Not Applicable	
26	NOCAM	Epinephelus morio	Red Grouper	N	Brought on Board (Retained/Discarded)	Live - Healthy	Retained	Not Applicable	
26	NOCAM	Epinephelus morio	Red Grouper	N	Brought on Board (Retained/Discarded)	Live - Healthy	Retained	Not Applicable	
26	NOCAM	Epinephelus morio	Red Grouper	N	Brought on Board (Retained/Discarded)	Live - Healthy	Discarded - Live and Healthy (Vented)	Not Applicable	
26	NOCAM	Epinephelus morio	Red Grouper	N	Brought on Board (Retained/Discarded)	Live - Healthy	Discarded - Live and Healthy (Vented)	Not Applicable	
26	NOCAM	Epinephelus morio	Red Grouper	N	Brought on Board (Retained/Discarded)	Live - Healthy	Discarded - Live and Healthy (Vented)	Not Applicable	
26	NOCAM	Epinephelus morio	Red Grouper	N	Brought on Board (Retained/Discarded)	Live - Stomach and/or Eyes Protruding	Retained	Not Applicable	
26	NOCAM	Epinephelus morio	Red Grouper	N	Brought on Board (Retained/Discarded)	Live - Stomach and/or Eyes Protruding	Retained	Not Applicable	
26	NOCAM	Epinephelus morio	Red Grouper	N	Brought on Board (Retained/Discarded)	Live - Healthy	Retained	Not Applicable	
26	NOCAM	Epinephelus morio	Red Grouper	N	Brought on Board (Retained/Discarded)	Live - Healthy	Retained	Not Applicable	
26	NOCAM	Epinephelus morio	Red Grouper	N	Brought on Board (Retained/Discarded)	Live - Healthy	Retained	Not Applicable	
26	NOCAM	Epinephelus morio	Red Grouper	N	Brought on Board (Retained/Discarded)	Live - Healthy	Discarded - Live and Healthy (Vented)	Not Applicable	
26	NOCAM	Epinephelus morio	Red Grouper	N	Brought on Board (Retained/Discarded)	Live - Healthy	Discarded - Live and Healthy (Vented)	Not Applicable	
26	NOCAM	Epinephelus morio	Red Grouper	N	Brought on Board (Retained/Discarded)	Live - Healthy	Retained	Not Applicable	
26	NOCAM	Epinephelus morio	Red Grouper	N	Brought on Board (Retained/Discarded)	Live - Healthy	Retained	Not Applicable	
26	NOCAM	Epinephelus morio	Red Grouper	N	Brought on Board (Retained/Discarded)	Live - Healthy	Retained	Not Applicable	
26	NOCAM	Epinephelus morio	Red Grouper	N	Brought on Board (Retained/Discarded)	Live - Healthy	Retained	Not Applicable	
26	NOCAM	Lutjanus synagris	Lane Snapper	N	Brought on Board (Retained/Discarded)	Live - Healthy	Retained	Not Applicable	
26	UCAM	Carcharhinus plumbeus	Sandbar Shark	Y	Cut-off at Rail (No Entanglement)	Live - Healthy	Discarded - Live and Healthy (Not Vented)	Male (Claspers)	Large (Greater than 2m)
26	UCAM	Carcharhinus plumbeus	Sandbar Shark	Y	Cut-off at Rail (No Entanglement)	Live - Healthy	Discarded - Live and Healthy (Not Vented)	Male (Claspers)	Large (Greater than 2m)
26	UCAM	Carcharhinus plumbeus	Sandbar Shark	Y	Cut-off at Rail (No Entanglement)	Live - Healthy	Discarded - Live and Healthy (Not Vented)	Female	Large (Greater than 2m)
26	UCAM	Carcharhinus plumbeus	Sandbar Shark	Y	Cut-off at Rail (No Entanglement)	Live - Healthy	Discarded - Live and Healthy (Not Vented)	Male (Claspers)	Large (Greater than 2m)
26	UCAM	Crab, unidentified	Crab, Unidentified	N	Brought on Board (Retained/Discarded)	Live - Healthy	Discarded - Live and Healthy (Not Vented)	Not Applicable	
26	UCAM	Epinephelus morio	Red Grouper	N	Brought on Board (Retained/Discarded)	Live - Healthy	Retained	Not Applicable	
26	UCAM	Epinephelus morio	Red Grouper	N	Not Handled (Dropped Off)	Live - Healthy	Discarded - Live and Healthy (Not Vented)	Not Applicable	
26	UCAM	Epinephelus morio	Red Grouper	N	Brought on Board (Retained/Discarded)	Live - Healthy	Retained	Not Applicable	
26	UCAM	Epinephelus morio	Red Grouper	N	Brought on Board (Retained/Discarded)	Live - Healthy	Retained	Not Applicable	
26	UCAM	Epinephelus morio	Red Grouper	N	Brought on Board (Retained/Discarded)	Live - Healthy	Discarded - Live and Healthy (Vented)	Not Applicable	
26	UCAM	Epinephelus morio	Red Grouper	N	Brought on Board (Retained/Discarded)	Live - Healthy	Discarded - Live and Healthy (Vented)	Not Applicable	
26	UCAM	Epinephelus morio	Red Grouper	N	Brought on Board (Retained/Discarded)	Live - Healthy	Discarded - Live and Healthy (Vented)	Not Applicable	
26	UCAM	Epinephelus morio	Red Grouper	N	Brought on Board (Retained/Discarded)	Live - Healthy	Retained	Not Applicable	
26	UCAM	Epinephelus morio	Red Grouper	N	Brought on Board (Retained/Discarded)	Live - Healthy	Retained	Not Applicable	
26	UCAM	Epinephelus morio	Red Grouper	N	Brought on Board (Retained/Discarded)	Live - Healthy	Retained	Not Applicable	
26	UCAM	Epinephelus morio	Red Grouper	N	Brought on Board (Retained/Discarded)	Live - Healthy	Discarded - Live and Healthy (Vented)	Not Applicable	
26	UCAM	Epinephelus morio	Red Grouper	N	Brought on Board (Retained/Discarded)	Live - Healthy	Discarded - Live and Healthy (Vented)	Not Applicable	
26	UCAM	Epinephelus morio	Red Grouper	N	Brought on Board (Retained/Discarded)	Live - Healthy	Retained	Not Applicable	
26	UCAM	Epinephelus morio	Red Grouper	N	Brought on Board (Retained/Discarded)	Live - Healthy	Discarded - Live and Healthy (Vented)	Not Applicable	
26	UCAM	Epinephelus morio	Red Grouper	N	Brought on Board (Retained/Discarded)	Live - Healthy	Retained	Not Applicable	
26	UCAM	Epinephelus morio	Red Grouper	N	Brought on Board (Retained/Discarded)	Live - Healthy	Discarded - Live and Healthy (Vented)	Not Applicable	
26	UCAM	Epinephelus morio	Red Grouper	N	Brought on Board (Retained/Discarded)	Live - Stomach and/or Eyes Protruding	Retained	Not Applicable	
26	UCAM	Epinephelus morio	Red Grouper	N	Brought on Board (Retained/Discarded)	Live - Healthy	Retained	Not Applicable	
26	UCAM	Epinephelus morio	Red Grouper	N	Brought on Board (Retained/Discarded)	Live - Healthy	Discarded - Live and Healthy (Vented)	Not Applicable	
26	UCAM	Epinephelus morio	Red Grouper	N	Brought on Board (Retained/Discarded)	Live - Healthy	Retained	Not Applicable	
26	UCAM	Epinephelus morio	Red Grouper	N	Brought on Board (Retained/Discarded)	Live - Healthy	Discarded - Live and Healthy (Vented)	Not Applicable	
26	UCAM	Epinephelus morio	Red Grouper	N	Brought on Board (Retained/Discarded)	Live - Healthy	Discarded - Live and Healthy (Vented)	Not Applicable	
26	UCAM	Epinephelus morio	Red Grouper	N	Brought on Board (Retained/Discarded)	Live - Healthy	Retained	Not Applicable	
26	UCAM	Epinephelus morio	Red Grouper	N	Brought on Board (Retained/Discarded)	Live - Stomach and/or Eyes Protruding	Discarded - Live and Healthy (Vented)	Not Applicable	
26	UCAM	Epinephelus morio	Red Grouper	N	Brought on Board (Retained/Discarded)	Live - Healthy	Discarded - Live and Healthy (Vented)	Not Applicable	
26	UCAM	Epinephelus morio	Red Grouper	N	Brought on Board (Retained/Discarded)	Live - Healthy	Retained	Not Applicable	
26	UCAM	Epinephelus morio	Red Grouper	N	Brought on Board (Retained/Discarded)	Live - Healthy	Retained	Not Applicable	
26	UCAM	Epinephelus morio	Red Grouper	N	Brought on Board (Retained/Discarded)	Live - Healthy	Discarded - Live and Healthy (Vented)	Not Applicable	
26	UCAM	Epinephelus morio	Red Grouper	N	Brought on Board (Retained/Discarded)	Live - Healthy	Discarded - Live and Healthy (Vented)	Not Applicable	
26	UCAM	Epinephelus morio	Red Grouper	N	Brought on Board (Retained/Discarded)	Live - Healthy	Retained	Not Applicable	
26	UCAM	Epinephelus morio	Red Grouper	N	Brought on Board (Retained/Discarded)	Live - Healthy	Retained	Not Applicable	
26	UCAM	Epinephelus morio	Red Grouper	N	Brought on Board (Retained/Discarded)	Live - Healthy	Retained	Not Applicable	
26	UCAM	Epinephelus morio	Red Grouper	N	Brought on Board (Retained/Discarded)	Live - Healthy	Discarded - Live and Healthy (Vented)	Not Applicable	
26	UCAM	Epinephelus morio	Red Grouper	N	Brought on Board (Retained/Discarded)	Live - Healthy	Retained	Not Applicable	
26	UCAM	Lutjanus synagris	Lane Snapper	N	Brought on Board (Retained/Discarded)	Live - Healthy	Retained	Not Applicable	
27	NOCAM	Anthozoa spp.	Coral	N	Brought on Board (Retained/Discarded)	Unknown Condition	Discarded - Unknown	Not Applicable	
27	NOCAM	Carcharhinus acronotus	Blacknose Shark	Y	Cut-off at Rail (No Entanglement)	Live - Healthy	Discarded - Live and Healthy (Not Vented)	Unknown Maturity and/or Sex	Medium (Between 1m and 2m)
27	NOCAM	Carcharhinus acronotus	Blacknose Shark	Y	Cut-off at Rail (No Entanglement)	Live - Healthy	Discarded - Live and Healthy (Not Vented)	Unknown Maturity and/or Sex	Medium (Between 1m and 2m)
27	NOCAM	Carcharhinus acronotus	Blacknose Shark	Y	Cut-off at Rail (No Entanglement)	Live - Healthy	Discarded - Live and Healthy (Not Vented)	Female	Medium (Between 1m and 2m)
27	NOCAM	Carcharhinus plumbeus	Sandbar Shark	Y	Cut-off at Rail (No Entanglement)	Live - Healthy	Discarded - Live and Healthy (Not Vented)	Known Adult - Undetermined Sex	Large (Greater than 2m)
27	NOCAM	Carcharhinus plumbeus	Sandbar Shark	Y	Cut-off at Rail (No Entanglement)	Live - Healthy	Discarded - Live and Healthy (Not Vented)	Known Adult - Undetermined Sex	Large (Greater than 2m)
27	NOCAM	Carcharhinus, unidentified	Carcharhinid, Unidentified	Y	Cut-off at Rail (No Entanglement)	Live - Healthy	Discarded - Live and Healthy (Not Vented)	Unknown Maturity and/or Sex	Large (Greater than 2m)
27	NOCAM	Crab, unidentified	Crab, Unidentified	N	Brought on Board (Retained/Discarded)	Live - Healthy	Discarded - Live and Healthy (Not Vented)	Not Applicable	
27	NOCAM	Epinephelus morio	Red Grouper	N	Brought on Board (Retained/Discarded)	Live - Healthy	Discarded - Live and Healthy (Vented)	Not Applicable	
27	NOCAM	Epinephelus morio	Red Grouper	N	Brought on Board (Retained/Discarded)	Live - Healthy	Discarded - Live and Healthy (Vented)	Not Applicable	
27	NOCAM	Epinephelus morio	Red Grouper	N	Brought on Board (Retained/Discarded)	Live - Stomach and/or Eyes Protruding	Retained	Not Applicable	
27	NOCAM	Epinephelus morio	Red Grouper	N	Brought on Board (Retained/Discarded)	Live - Healthy	Retained	Not Applicable	
27	NOCAM	Epinephelus morio	Red Grouper	N	Brought on Board (Retained/Discarded)	Live - Stomach and/or Eyes Protruding	Retained	Not Applicable	
27	NOCAM	Epinephelus morio	Red Grouper	N	Brought on Board (Retained/Discarded)	Live - Healthy	Retained	Not Applicable	
27	NOCAM	Epinephelus morio	Red Grouper	N	Brought on Board (Retained/Discarded)	Live - Healthy	Discarded - Live and Healthy (Vented)	Not Applicable	
27	NOCAM	Epinephelus morio	Red Grouper	N	Brought on Board (Retained/Discarded)	Live - Healthy	Discarded - Live and Healthy (Vented)	Not Applicable	
27	NOCAM	Epinephelus morio	Red Grouper	N	Brought on Board (Retained/Discarded)	Live - Healthy	Retained	Not Applicable	
27	NOCAM	Epinephelus morio	Red Grouper	N	Brought on Board (Retained/Discarded)	Live - Stomach and/or Eyes Protruding	Retained	Not Applicable	
27	NOCAM	Epinephelus morio	Red Grouper	N	Brought on Board (Retained/Discarded)	Live - Healthy	Discarded - Live and Healthy (Vented)	Not Applicable	
27	NOCAM	Epinephelus morio	Red Grouper	N	Brought on Board (Retained/Discarded)	Live - Healthy	Discarded - Live and Healthy (Vented)	Not Applicable	
27	NOCAM	Epinephelus morio	Red Grouper	N	Brought on Board (Retained/Discarded)	Live - Healthy	Discarded - Live and Healthy (Vented)	Not Applicable	
27	NOCAM	Epinephelus morio	Red Grouper	N	Brought on Board (Retained/Discarded)	Live - Healthy	Discarded - Live and Healthy (Vented)	Not Applicable	
27	NOCAM	Epinephelus morio	Red Grouper	N	Brought on Board (Retained/Discarded)	Live - Healthy	Retained	Not Applicable	
27	NOCAM	Epinephelus morio	Red Grouper	N	Brought on Board (Retained/Discarded)	Live - Healthy	Discarded - Live and Healthy (Vented)	Not Applicable	
27	NOCAM	Epinephelus morio	Red Grouper	N	Brought on Board (Retained/Discarded)	Live - Stomach and/or Eyes Protruding	Retained	Not Applicable	
27	NOCAM	Epinephelus morio	Red Grouper	N	Brought on Board (Retained/Discarded)	Live - Healthy	Discarded - Live and Healthy (Vented)	Not Applicable	
27	NOCAM	Epinephelus morio	Red Grouper	N	Brought on Board (Retained/Discarded)	Live - Healthy	Retained	Not Applicable	
27	NOCAM	Epinephelus morio	Red Grouper	N	Brought on Board (Retained/Discarded)	Live - Healthy	Retained	Not Applicable	
27	NOCAM	Epinephelus morio	Red Grouper	N	Brought on Board (Retained/Discarded)	Live - Healthy	Discarded - Live and Healthy (Vented)	Not Applicable	
27	NOCAM	Epinephelus morio	Red Grouper	N	Brought on Board (Retained/Discarded)	Live - Healthy	Discarded - Live and Healthy (Vented)	Not Applicable	
27	NOCAM	Epinephelus morio	Red Grouper	N	Brought on Board (Retained/Discarded)	Live - Healthy	Retained	Not Applicable	
27	NOCAM	Epinephelus morio	Red Grouper	N	Brought on Board (Retained/Discarded)	Live - Healthy	Discarded - Live and Healthy (Vented)	Not Applicable	

27	NOCAM	Epinephelus morio	Red Grouper	N	Brought on Board (Retained/Discarded)	Live - Healthy	Retained	Not Applicable	
27	NOCAM	Epinephelus morio	Red Grouper	N	Brought on Board (Retained/Discarded)	Live - Stomach and/or Eyes Protruding	Retained	Not Applicable	
27	NOCAM	Epinephelus morio	Red Grouper	N	Brought on Board (Retained/Discarded)	Live - Healthy	Retained	Not Applicable	
27	NOCAM	Epinephelus morio	Red Grouper	N	Brought on Board (Retained/Discarded)	Live - Healthy	Retained	Not Applicable	
27	NOCAM	Epinephelus morio	Red Grouper	N	Brought on Board (Retained/Discarded)	Live - Healthy	Retained	Not Applicable	
27	NOCAM	Ginglymostoma cirratum	Nurse Shark	Y	Cut-off at Rail (No Entanglement)	Live - Healthy	Discarded - Live and Healthy (Not Vented)	Known Adult - Undetermined Sex	Large (Greater than 2m)
27	NOCAM	Muraenidae, unidentified	Moray Eel, Unidentified	N	Brought on Board (Retained/Discarded)	Live - Healthy	Retained as Bait	Not Applicable	
27	NOCAM	Sphyrna makarran	Great Hammerhead	Y	Cut-off at Rail (No Entanglement)	Dead on Arrival - Undamaged	Discarded - Dead	Unknown Maturity and/or Sex	Large (Greater than 2m)
27	UCAM	Anthozoa spp.	Coral	N	Brought on Board (Retained/Discarded)	Unknown Condition	Discarded - Unknown	Not Applicable	
27	UCAM	Carcharhinus acronotus	Blacknose Shark	Y	Brought on Board (Retained/Discarded)	Live - Healthy	Discarded - Live and Healthy (Not Vented)	Female	Medium (Between 1m and 2m)
27	UCAM	Carcharhinus acronotus	Blacknose Shark	Y	Cut-off at Rail (No Entanglement)	Live - Healthy	Discarded - Live and Healthy (Not Vented)	Male (Claspers)	Medium (Between 1m and 2m)
27	UCAM	Carcharhinus acronotus	Blacknose Shark	Y	Cut-off at Rail (No Entanglement)	Live - Healthy	Discarded - Live and Healthy (Not Vented)	Female	Medium (Between 1m and 2m)
27	UCAM	Carcharhinus plumbeus	Sandbar Shark	Y	Cut-off at Rail (No Entanglement)	Live - Healthy	Discarded - Live and Healthy (Not Vented)	Female	Large (Greater than 2m)
27	UCAM	Carcharhinus plumbeus	Sandbar Shark	Y	Cut-off at Rail (No Entanglement)	Live - Healthy	Discarded - Live and Healthy (Not Vented)	Male (Claspers)	Large (Greater than 2m)
27	UCAM	Carcharhinus plumbeus	Sandbar Shark	Y	Cut-off at Rail (No Entanglement)	Live - Healthy	Discarded - Live and Healthy (Not Vented)	Female	Medium (Between 1m and 2m)
27	UCAM	Crab, unidentified	Crab, Unidentified	N	Brought on Board (Retained/Discarded)	Live - Healthy	Discarded - Live and Healthy (Not Vented)	Not Applicable	
27	UCAM	Epinephelus morio	Red Grouper	N	Brought on Board (Retained/Discarded)	Live - Healthy	Retained	Not Applicable	
27	UCAM	Epinephelus morio	Red Grouper	N	Brought on Board (Retained/Discarded)	Live - Healthy	Discarded - Live and Healthy (Vented)	Not Applicable	
27	UCAM	Epinephelus morio	Red Grouper	N	Brought on Board (Retained/Discarded)	Live - Healthy	Retained	Not Applicable	
27	UCAM	Epinephelus morio	Red Grouper	N	Brought on Board (Retained/Discarded)	Live - Healthy	Retained	Not Applicable	
27	UCAM	Epinephelus morio	Red Grouper	N	Brought on Board (Retained/Discarded)	Live - Stomach and/or Eyes Protruding	Retained	Not Applicable	
27	UCAM	Epinephelus morio	Red Grouper	N	Brought on Board (Retained/Discarded)	Live - Healthy	Discarded - Live and Healthy (Vented)	Not Applicable	
27	UCAM	Epinephelus morio	Red Grouper	N	Brought on Board (Retained/Discarded)	Live - Healthy	Retained	Not Applicable	
27	UCAM	Epinephelus morio	Red Grouper	N	Brought on Board (Retained/Discarded)	Live - Healthy	Discarded - Live and Healthy (Vented)	Not Applicable	
27	UCAM	Epinephelus morio	Red Grouper	N	Brought on Board (Retained/Discarded)	Live - Stomach and/or Eyes Protruding	Discarded - Live and Healthy (Vented)	Not Applicable	
27	UCAM	Epinephelus morio	Red Grouper	N	Brought on Board (Retained/Discarded)	Live - Healthy	Discarded - Live and Healthy (Vented)	Not Applicable	
27	UCAM	Epinephelus morio	Red Grouper	N	Brought on Board (Retained/Discarded)	Live - Healthy	Discarded - Live and Healthy (Vented)	Not Applicable	
27	UCAM	Epinephelus morio	Red Grouper	N	Brought on Board (Retained/Discarded)	Live - Healthy	Discarded - Live and Healthy (Vented)	Not Applicable	
27	UCAM	Epinephelus morio	Red Grouper	N	Brought on Board (Retained/Discarded)	Live - Stomach and/or Eyes Protruding	Discarded - Live and Healthy (Vented)	Not Applicable	
27	UCAM	Epinephelus morio	Red Grouper	N	Brought on Board (Retained/Discarded)	Live - Stomach and/or Eyes Protruding	Discarded - Live and Healthy (Vented)	Not Applicable	
27	UCAM	Epinephelus morio	Red Grouper	N	Brought on Board (Retained/Discarded)	Live - Healthy	Discarded - Live and Healthy (Vented)	Not Applicable	
27	UCAM	Epinephelus morio	Red Grouper	N	Brought on Board (Retained/Discarded)	Live - Healthy	Retained	Not Applicable	
27	UCAM	Epinephelus morio	Red Grouper	N	Brought on Board (Retained/Discarded)	Live - Healthy	Discarded - Live and Healthy (Vented)	Not Applicable	
27	UCAM	Epinephelus morio	Red Grouper	N	Brought on Board (Retained/Discarded)	Live - Healthy	Retained	Not Applicable	
27	UCAM	Epinephelus morio	Red Grouper	N	Brought on Board (Retained/Discarded)	Live - Healthy	Discarded - Live and Healthy (Vented)	Not Applicable	
27	UCAM	Epinephelus morio	Red Grouper	N	Brought on Board (Retained/Discarded)	Live - Healthy	Retained	Not Applicable	
27	UCAM	Epinephelus morio	Red Grouper	N	Brought on Board (Retained/Discarded)	Live - Healthy	Retained	Not Applicable	
27	UCAM	Epinephelus morio	Red Grouper	N	Brought on Board (Retained/Discarded)	Live - Stomach and/or Eyes Protruding	Retained	Not Applicable	
27	UCAM	Epinephelus morio	Red Grouper	N	Brought on Board (Retained/Discarded)	Live - Healthy	Discarded - Live and Healthy (Vented)	Not Applicable	
27	UCAM	Epinephelus morio	Red Grouper	N	Brought on Board (Retained/Discarded)	Live - Healthy	Discarded - Live and Healthy (Vented)	Not Applicable	
27	UCAM	Epinephelus morio	Red Grouper	N	Brought on Board (Retained/Discarded)	Live - Healthy	Retained	Not Applicable	
27	UCAM	Epinephelus morio	Red Grouper	N	Brought on Board (Retained/Discarded)	Live - Stomach and/or Eyes Protruding	Retained	Not Applicable	
27	UCAM	Epinephelus morio	Red Grouper	N	Brought on Board (Retained/Discarded)	Live - Healthy	Discarded - Live and Healthy (Vented)	Not Applicable	
27	UCAM	Epinephelus morio	Red Grouper	N	Brought on Board (Retained/Discarded)	Live - Healthy	Discarded - Live and Healthy (Vented)	Not Applicable	
27	UCAM	Epinephelus morio	Red Grouper	N	Brought on Board (Retained/Discarded)	Live - Stomach and/or Eyes Protruding	Retained	Not Applicable	
27	UCAM	Ginglymostoma cirratum	Nurse Shark	Y	Cut-off at Rail (No Entanglement)	Live - Healthy	Discarded - Live and Healthy (Not Vented)	Known Adult - Undetermined Sex	Large (Greater than 2m)
27	UCAM	Muraenidae, unidentified	Moray Eel, Unidentified	N	Brought on Board (Retained/Discarded)	Live - Healthy	Retained as Bait	Not Applicable	
27	UCAM	Sphyrna makarran	Great Hammerhead	Y	Cut-off at Rail (No Entanglement)	Dead on Arrival - Undamaged	Discarded - Dead	Unknown Maturity and/or Sex	Large (Greater than 2m)
28	NOCAM	Carcharhinus plumbeus	Sandbar Shark	Y	Cut-off at Rail (No Entanglement)	Live - Healthy	Discarded - Live and Healthy (Not Vented)	Known Adult - Undetermined Sex	Large (Greater than 2m)
28	NOCAM	Carcharhinus plumbeus	Sandbar Shark	Y	Cut-off at Rail (No Entanglement)	Live - Healthy	Discarded - Live and Healthy (Not Vented)	Female	Large (Greater than 2m)
28	NOCAM	Epinephelus morio	Red Grouper	N	Brought on Board (Retained/Discarded)	Live - Healthy	Discarded - Live and Healthy (Vented)	Not Applicable	
28	NOCAM	Epinephelus morio	Red Grouper	N	Brought on Board (Retained/Discarded)	Live - Healthy	Discarded - Live and Healthy (Vented)	Not Applicable	
28	NOCAM	Epinephelus morio	Red Grouper	N	Brought on Board (Retained/Discarded)	Live - Healthy	Retained	Not Applicable	
28	NOCAM	Epinephelus morio	Red Grouper	N	Brought on Board (Retained/Discarded)	Live - Healthy	Discarded - Live and Healthy (Vented)	Not Applicable	
28	NOCAM	Epinephelus morio	Red Grouper	N	Brought on Board (Retained/Discarded)	Live - Healthy	Retained	Not Applicable	
28	NOCAM	Epinephelus morio	Red Grouper	N	Brought on Board (Retained/Discarded)	Live - Healthy	Discarded - Live and Healthy (Vented)	Not Applicable	
28	NOCAM	Epinephelus morio	Red Grouper	N	Brought on Board (Retained/Discarded)	Live - Healthy	Retained	Not Applicable	
28	NOCAM	Epinephelus morio	Red Grouper	N	Brought on Board (Retained/Discarded)	Live - Stomach and/or Eyes Protruding	Discarded - Live and Healthy (Vented)	Not Applicable	
28	NOCAM	Epinephelus morio	Red Grouper	N	Not Handled (Dropped Off)	Live - Healthy	Discarded - Live and Healthy (Not Vented)	Not Applicable	
28	NOCAM	Epinephelus morio	Red Grouper	N	Brought on Board (Retained/Discarded)	Live - Healthy	Discarded - Live and Healthy (Vented)	Not Applicable	
28	NOCAM	Epinephelus morio	Red Grouper	N	Brought on Board (Retained/Discarded)	Live - Healthy	Discarded - Live and Healthy (Vented)	Not Applicable	
28	NOCAM	Epinephelus morio	Red Grouper	N	Brought on Board (Retained/Discarded)	Live - Healthy	Discarded - Live and Healthy (Vented)	Not Applicable	
28	NOCAM	Epinephelus morio	Red Grouper	N	Brought on Board (Retained/Discarded)	Live - Healthy	Retained	Not Applicable	
28	NOCAM	Epinephelus morio	Red Grouper	N	Brought on Board (Retained/Discarded)	Live - Healthy	Discarded - Live and Healthy (Vented)	Not Applicable	
28	NOCAM	Epinephelus morio	Red Grouper	N	Brought on Board (Retained/Discarded)	Live - Healthy	Retained	Not Applicable	
28	NOCAM	Epinephelus morio	Red Grouper	N	Brought on Board (Retained/Discarded)	Live - Healthy	Discarded - Live and Healthy (Vented)	Not Applicable	
28	NOCAM	Epinephelus morio	Red Grouper	N	Brought on Board (Retained/Discarded)	Live - Healthy	Retained	Not Applicable	
28	NOCAM	Epinephelus morio	Red Grouper	N	Brought on Board (Retained/Discarded)	Live - Stomach and/or Eyes Protruding	Retained	Not Applicable	
28	NOCAM	Epinephelus morio	Red Grouper	N	Brought on Board (Retained/Discarded)	Live - Healthy	Retained	Not Applicable	
28	NOCAM	Epinephelus morio	Red Grouper	N	Brought on Board (Retained/Discarded)	Live - Healthy	Discarded - Live and Healthy (Vented)	Not Applicable	
28	NOCAM	Epinephelus morio	Red Grouper	N	Brought on Board (Retained/Discarded)	Live - Healthy	Retained	Not Applicable	
28	NOCAM	Epinephelus morio	Red Grouper	N	Brought on Board (Retained/Discarded)	Live - Healthy	Discarded - Live and Healthy (Vented)	Not Applicable	
28	NOCAM	Epinephelus morio	Red Grouper	N	Brought on Board (Retained/Discarded)	Live - Healthy	Retained	Not Applicable	
28	NOCAM	Epinephelus morio	Red Grouper	N	Brought on Board (Retained/Discarded)	Live - Healthy	Discarded - Live and Healthy (Vented)	Not Applicable	
28	NOCAM	Epinephelus morio	Red Grouper	N	Brought on Board (Retained/Discarded)	Live - Healthy	Discarded - Live and Healthy (Vented)	Not Applicable	
28	NOCAM	Epinephelus morio	Red Grouper	N	Brought on Board (Retained/Discarded)	Live - Stomach and/or Eyes Protruding	Retained	Not Applicable	
28	NOCAM	Epinephelus morio	Red Grouper	N	Brought on Board (Retained/Discarded)	Live - Stomach and/or Eyes Protruding	Retained	Not Applicable	
28	NOCAM	Epinephelus morio	Red Grouper	N	Brought on Board (Retained/Discarded)	Live - Stomach and/or Eyes Protruding	Discarded - Live and Healthy (Vented)	Not Applicable	
28	NOCAM	Epinephelus morio	Red Grouper	N	Brought on Board (Retained/Discarded)	Live - Healthy	Discarded - Live and Healthy (Vented)	Not Applicable	
28	NOCAM	Epinephelus morio	Red Grouper	N	Brought on Board (Retained/Discarded)	Live - Healthy	Retained	Not Applicable	
28	NOCAM	Epinephelus morio	Red Grouper	N	Brought on Board (Retained/Discarded)	Live - Stomach and/or Eyes Protruding	Discarded - Live and Healthy (Vented)	Not Applicable	
28	NOCAM	Rhizoprionodon terraenovae	Atlantic Sharpnose Shark	Y	Brought on Board (Retained/Discarded)	Live - Healthy	Discarded - Live and Healthy (Not Vented)	Female	Small (Less than 1m)
28	UCAM	Carcharhinus plumbeus	Sandbar Shark	Y	Cut-off at Rail (No Entanglement)	Live - Healthy	Discarded - Live and Healthy (Not Vented)	Female	Large (Greater than 2m)
28	UCAM	Carcharhinus plumbeus	Sandbar Shark	Y	Cut-off at Rail (No Entanglement)	Live - Healthy	Discarded - Live and Healthy (Not Vented)	Female	Large (Greater than 2m)
28	UCAM	Epinephelus morio	Red Grouper	N	Brought on Board (Retained/Discarded)	Live - Healthy	Discarded - Live and Healthy (Vented)	Not Applicable	
28	UCAM	Epinephelus morio	Red Grouper	N	Brought on Board (Retained/Discarded)	Live - Healthy	Retained	Not Applicable	
28	UCAM	Epinephelus morio	Red Grouper	N	Brought on Board (Retained/Discarded)	Live - Healthy	Retained	Not Applicable	
28	UCAM	Epinephelus morio	Red Grouper	N	Brought on Board (Retained/Discarded)	Live - Healthy	Discarded - Live and Healthy (Vented)	Not Applicable	
28	UCAM	Epinephelus morio	Red Grouper	N	Brought on Board (Retained/Discarded)	Live - Healthy	Discarded - Live and Healthy (Vented)	Not Applicable	
28	UCAM	Epinephelus morio	Red Grouper	N	Brought on Board (Retained/Discarded)	Live - Healthy	Discarded - Live and Healthy (Vented)	Not Applicable	
28	UCAM	Epinephelus morio	Red Grouper	N	Brought on Board (Retained/Discarded)	Live - Healthy	Retained	Not Applicable	
28	UCAM	Epinephelus morio	Red Grouper	N	Brought on Board (Retained/Discarded)	Live - Healthy	Discarded - Live and Healthy (Vented)	Not Applicable	

28	UCAM	Epinephelus morio	Red Grouper	N	Brought on Board (Retained/Discarded)	Live - Healthy	Retained	Not Applicable	
28	UCAM	Epinephelus morio	Red Grouper	N	Brought on Board (Retained/Discarded)	Live - Healthy	Discarded - Live and Healthy (Vented)	Not Applicable	
28	UCAM	Epinephelus morio	Red Grouper	N	Brought on Board (Retained/Discarded)	Live - Healthy	Retained	Not Applicable	
28	UCAM	Epinephelus morio	Red Grouper	N	Brought on Board (Retained/Discarded)	Live - Healthy	Discarded - Live and Healthy (Vented)	Not Applicable	
28	UCAM	Epinephelus morio	Red Grouper	N	Brought on Board (Retained/Discarded)	Live - Healthy	Discarded - Live and Healthy (Vented)	Not Applicable	
28	UCAM	Epinephelus morio	Red Grouper	N	Brought on Board (Retained/Discarded)	Live - Stomach and/or Eyes Protruding	Retained	Not Applicable	
28	UCAM	Epinephelus morio	Red Grouper	N	Brought on Board (Retained/Discarded)	Live - Stomach and/or Eyes Protruding	Retained	Not Applicable	
28	UCAM	Epinephelus morio	Red Grouper	N	Brought on Board (Retained/Discarded)	Live - Healthy	Retained	Not Applicable	
28	UCAM	Epinephelus morio	Red Grouper	N	Brought on Board (Retained/Discarded)	Live - Stomach and/or Eyes Protruding	Retained	Not Applicable	
28	UCAM	Epinephelus morio	Red Grouper	N	Brought on Board (Retained/Discarded)	Live - Healthy	Retained	Not Applicable	
28	UCAM	Epinephelus morio	Red Grouper	N	Brought on Board (Retained/Discarded)	Live - Healthy	Retained	Not Applicable	
28	UCAM	Epinephelus morio	Red Grouper	N	Brought on Board (Retained/Discarded)	Live - Healthy	Discarded - Live and Healthy (Vented)	Not Applicable	
28	UCAM	Epinephelus morio	Red Grouper	N	Brought on Board (Retained/Discarded)	Live - Healthy	Retained	Not Applicable	
28	UCAM	Epinephelus morio	Red Grouper	N	Brought on Board (Retained/Discarded)	Live - Healthy	Discarded - Live and Healthy (Vented)	Not Applicable	
28	UCAM	Epinephelus morio	Red Grouper	N	Brought on Board (Retained/Discarded)	Live - Healthy	Discarded - Live and Healthy (Vented)	Not Applicable	
28	UCAM	Epinephelus morio	Red Grouper	N	Brought on Board (Retained/Discarded)	Live - Healthy	Discarded - Live and Healthy (Vented)	Not Applicable	
28	UCAM	Epinephelus morio	Red Grouper	N	Brought on Board (Retained/Discarded)	Live - Healthy	Discarded - Live and Healthy (Vented)	Not Applicable	
28	UCAM	Epinephelus morio	Red Grouper	N	Brought on Board (Retained/Discarded)	Live - Stomach and/or Eyes Protruding	Discarded - Live and Healthy (Vented)	Not Applicable	
28	UCAM	Epinephelus morio	Red Grouper	N	Brought on Board (Retained/Discarded)	Live - Stomach and/or Eyes Protruding	Retained	Not Applicable	
28	UCAM	Epinephelus morio	Red Grouper	N	Brought on Board (Retained/Discarded)	Live - Stomach and/or Eyes Protruding	Discarded - Live and Healthy (Vented)	Not Applicable	
28	UCAM	Rhizoprionodon terraenovae	Atlantic Sharpnose Shark	Y	Brought on Board (Retained/Discarded)	Live - Healthy	Discarded - Live and Healthy (Not Vented)	Female	Small (Less than 1m)
29	NOCAM	Carcharhinus acronotus	Blacknose Shark	Y	Brought on Board (Retained/Discarded)	Live - Healthy	Discarded - Live and Healthy (Not Vented)	Female	Small (Less than 1m)
29	NOCAM	Carcharhinus acronotus	Blacknose Shark	Y	Brought on Board (Retained/Discarded)	Live - Healthy	Discarded - Live and Healthy (Not Vented)	Male (Claspers)	Small (Less than 1m)
29	NOCAM	Epinephelus morio	Red Grouper	N	Brought on Board (Retained/Discarded)	Live - Healthy	Discarded - Live and Healthy (Vented)	Not Applicable	
29	NOCAM	Epinephelus morio	Red Grouper	N	Brought on Board (Retained/Discarded)	Live - Healthy	Retained	Not Applicable	
29	NOCAM	Epinephelus morio	Red Grouper	N	Brought on Board (Retained/Discarded)	Live - Healthy	Discarded - Live and Healthy (Vented)	Not Applicable	
29	NOCAM	Epinephelus morio	Red Grouper	N	Brought on Board (Retained/Discarded)	Live - Stomach and/or Eyes Protruding	Retained	Not Applicable	
29	NOCAM	Epinephelus morio	Red Grouper	N	Brought on Board (Retained/Discarded)	Live - Healthy	Retained	Not Applicable	
29	NOCAM	Epinephelus morio	Red Grouper	N	Brought on Board (Retained/Discarded)	Live - Healthy	Discarded - Live and Healthy (Vented)	Not Applicable	
29	NOCAM	Epinephelus morio	Red Grouper	N	Brought on Board (Retained/Discarded)	Live - Healthy	Retained	Not Applicable	
29	NOCAM	Epinephelus morio	Red Grouper	N	Brought on Board (Retained/Discarded)	Live - Stomach and/or Eyes Protruding	Retained	Not Applicable	
29	NOCAM	Epinephelus morio	Red Grouper	N	Brought on Board (Retained/Discarded)	Live - Healthy	Discarded - Live and Healthy (Not Vented)	Not Applicable	
29	NOCAM	Epinephelus morio	Red Grouper	N	Brought on Board (Retained/Discarded)	Live - Healthy	Discarded - Live and Healthy (Vented)	Not Applicable	
29	NOCAM	Epinephelus morio	Red Grouper	N	Brought on Board (Retained/Discarded)	Live - Stomach and/or Eyes Protruding	Discarded - Live and Healthy (Vented)	Not Applicable	
29	NOCAM	Epinephelus morio	Red Grouper	N	Brought on Board (Retained/Discarded)	Live - Healthy	Discarded - Live and Healthy (Vented)	Not Applicable	
29	NOCAM	Epinephelus morio	Red Grouper	N	Brought on Board (Retained/Discarded)	Live - Healthy	Retained	Not Applicable	
29	NOCAM	Epinephelus morio	Red Grouper	N	Brought on Board (Retained/Discarded)	Live - Healthy	Retained	Not Applicable	
29	NOCAM	Epinephelus morio	Red Grouper	N	Brought on Board (Retained/Discarded)	Live - Healthy	Discarded - Live and Healthy (Vented)	Not Applicable	
29	NOCAM	Epinephelus morio	Red Grouper	N	Brought on Board (Retained/Discarded)	Live - Stomach and/or Eyes Protruding	Retained	Not Applicable	
29	NOCAM	Epinephelus morio	Red Grouper	N	Brought on Board (Retained/Discarded)	Live - Healthy	Discarded - Live and Healthy (Vented)	Not Applicable	
29	NOCAM	Epinephelus morio	Red Grouper	N	Brought on Board (Retained/Discarded)	Live - Healthy	Retained	Not Applicable	
29	NOCAM	Epinephelus morio	Red Grouper	N	Brought on Board (Retained/Discarded)	Live - Healthy	Discarded - Live and Healthy (Vented)	Not Applicable	
29	NOCAM	Epinephelus morio	Red Grouper	N	Brought on Board (Retained/Discarded)	Live - Healthy	Retained	Not Applicable	
29	NOCAM	Epinephelus morio	Red Grouper	N	Brought on Board (Retained/Discarded)	Live - Healthy	Retained	Not Applicable	
29	NOCAM	Epinephelus morio	Red Grouper	N	Brought on Board (Retained/Discarded)	Live - Healthy	Retained	Not Applicable	
29	NOCAM	Epinephelus morio	Red Grouper	N	Brought on Board (Retained/Discarded)	Live - Healthy	Discarded - Live and Healthy (Vented)	Not Applicable	
29	NOCAM	Epinephelus morio	Red Grouper	N	Brought on Board (Retained/Discarded)	Live - Healthy	Discarded - Live and Healthy (Vented)	Not Applicable	
29	NOCAM	Epinephelus morio	Red Grouper	N	Brought on Board (Retained/Discarded)	Live - Healthy	Retained	Not Applicable	
29	NOCAM	Epinephelus morio	Red Grouper	N	Brought on Board (Retained/Discarded)	Live - Healthy	Discarded - Live and Healthy (Vented)	Not Applicable	
29	NOCAM	Epinephelus morio	Red Grouper	N	Brought on Board (Retained/Discarded)	Live - Healthy	Retained	Not Applicable	
29	NOCAM	Epinephelus morio	Red Grouper	N	Brought on Board (Retained/Discarded)	Live - Healthy	Discarded - Live and Healthy (Vented)	Not Applicable	
29	NOCAM	Epinephelus morio	Red Grouper	N	Brought on Board (Retained/Discarded)	Live - Healthy	Retained	Not Applicable	
29	NOCAM	Epinephelus morio	Red Grouper	N	Brought on Board (Retained/Discarded)	Live - Healthy	Discarded - Live and Healthy (Vented)	Not Applicable	
29	NOCAM	Epinephelus morio	Red Grouper	N	Brought on Board (Retained/Discarded)	Live - Healthy	Discarded - Live and Healthy (Vented)	Not Applicable	
29	NOCAM	Epinephelus morio	Red Grouper	N	Brought on Board (Retained/Discarded)	Live - Healthy	Discarded - Live and Healthy (Vented)	Not Applicable	
29	NOCAM	Epinephelus morio	Red Grouper	N	Brought on Board (Retained/Discarded)	Live - Healthy	Retained	Not Applicable	
29	NOCAM	Epinephelus morio	Red Grouper	N	Brought on Board (Retained/Discarded)	Live - Stomach and/or Eyes Protruding	Retained	Not Applicable	
29	NOCAM	Epinephelus morio	Red Grouper	N	Brought on Board (Retained/Discarded)	Live - Stomach and/or Eyes Protruding	Retained	Not Applicable	
29	NOCAM	Rhizoprionodon terraenovae	Atlantic Sharpnose Shark	Y	Brought on Board (Retained/Discarded)	Live - Healthy	Discarded - Live and Healthy (Not Vented)	Male (Claspers)	Small (Less than 1m)
29	NOCAM	Rhizoprionodon terraenovae	Atlantic Sharpnose Shark	Y	Brought on Board (Retained/Discarded)	Unknown Condition	Discarded - Unknown	Unknown Maturity and/or Sex	Small (Less than 1m)
29	NOCAM	Rhizoprionodon terraenovae	Atlantic Sharpnose Shark	Y	Brought on Board (Retained/Discarded)	Live - Healthy	Discarded - Live and Healthy (Not Vented)	Female	Small (Less than 1m)
29	NOCAM	Rhizoprionodon terraenovae	Atlantic Sharpnose Shark	Y	Brought on Board (Retained/Discarded)	Live - Healthy	Discarded - Live and Healthy (Not Vented)	Female	Small (Less than 1m)
29	NOCAM	Selachimorpha, unidentified	Shark, Unidentified	Y	Cut-off at Rail (No Entanglement)	Live - Healthy	Discarded - Live and Healthy (Not Vented)	Unknown Maturity and/or Sex	Medium (Between 1m and 2m)
29	NOCAM	Sphyrna lewini	Scalloped Hammerhead	Y	Cut-off at Rail (No Entanglement)	Dead on Arrival - Undamaged	Discarded - Dead	Unknown Maturity and/or Sex	Medium (Between 1m and 2m)
29	UCAM	Carcharhinus acronotus	Blacknose Shark	Y	Brought on Board (Retained/Discarded)	Live - Healthy	Discarded - Live and Healthy (Not Vented)	Male (Claspers)	Small (Less than 1m)
29	UCAM	Carcharhinus acronotus	Blacknose Shark	Y	Brought on Board (Retained/Discarded)	Live - Healthy	Discarded - Live and Healthy (Not Vented)	Female	Small (Less than 1m)
29	UCAM	Carcharhinus limbatus	Blacktip Shark	Y	Cut-off at Rail (No Entanglement)	Live - Healthy	Discarded - Live and Healthy (Not Vented)	Male (Claspers)	Medium (Between 1m and 2m)
29	UCAM	Carcharhinus plumbeus	Sandbar Shark	Y	Cut-off at Rail (No Entanglement)	Live - Healthy	Discarded - Live and Healthy (Not Vented)	Male (Claspers)	Large (Greater than 2m)
29	UCAM	Epinephelus morio	Red Grouper	N	Brought on Board (Retained/Discarded)	Live - Healthy	Discarded - Live and Healthy (Vented)	Not Applicable	
29	UCAM	Epinephelus morio	Red Grouper	N	Brought on Board (Retained/Discarded)	Live - Healthy	Discarded - Live and Healthy (Vented)	Not Applicable	
29	UCAM	Epinephelus morio	Red Grouper	N	Brought on Board (Retained/Discarded)	Live - Healthy	Retained	Not Applicable	
29	UCAM	Epinephelus morio	Red Grouper	N	Brought on Board (Retained/Discarded)	Live - Healthy	Discarded - Live and Healthy (Vented)	Not Applicable	
29	UCAM	Epinephelus morio	Red Grouper	N	Brought on Board (Retained/Discarded)	Live - Healthy	Discarded - Live and Healthy (Not Vented)	Not Applicable	
29	UCAM	Epinephelus morio	Red Grouper	N	Brought on Board (Retained/Discarded)	Live - Healthy	Discarded - Live and Healthy (Vented)	Not Applicable	
29	UCAM	Epinephelus morio	Red Grouper	N	Brought on Board (Retained/Discarded)	Live - Healthy	Retained	Not Applicable	
29	UCAM	Epinephelus morio	Red Grouper	N	Brought on Board (Retained/Discarded)	Live - Healthy	Retained	Not Applicable	
29	UCAM	Epinephelus morio	Red Grouper	N	Brought on Board (Retained/Discarded)	Live - Healthy	Discarded - Live and Healthy (Vented)	Not Applicable	
29	UCAM	Epinephelus morio	Red Grouper	N	Brought on Board (Retained/Discarded)	Live - Stomach and/or Eyes Protruding	Retained	Not Applicable	
29	UCAM	Epinephelus morio	Red Grouper	N	Brought on Board (Retained/Discarded)	Live - Stomach and/or Eyes Protruding	Retained	Not Applicable	
29	UCAM	Epinephelus morio	Red Grouper	N	Brought on Board (Retained/Discarded)	Live - Stomach and/or Eyes Protruding	Retained	Not Applicable	
29	UCAM	Epinephelus morio	Red Grouper	N	Brought on Board (Retained/Discarded)	Live - Healthy	Discarded - Live and Healthy (Vented)	Not Applicable	
29	UCAM	Epinephelus morio	Red Grouper	N	Brought on Board (Retained/Discarded)	Live - Stomach and/or Eyes Protruding	Retained	Not Applicable	
29	UCAM	Epinephelus morio	Red Grouper	N	Brought on Board (Retained/Discarded)	Live - Healthy	Retained	Not Applicable	
29	UCAM	Epinephelus morio	Red Grouper	N	Brought on Board (Retained/Discarded)	Live - Healthy	Discarded - Live and Healthy (Vented)	Not Applicable	
29	UCAM	Epinephelus morio	Red Grouper	N	Brought on Board (Retained/Discarded)	Live - Healthy	Discarded - Live and Healthy (Vented)	Not Applicable	
29	UCAM	Epinephelus morio	Red Grouper	N	Brought on Board (Retained/Discarded)	Live - Healthy	Retained	Not Applicable	
29	UCAM	Epinephelus morio	Red Grouper	N	Brought on Board (Retained/Discarded)	Live - Healthy	Retained	Not Applicable	
29	UCAM	Epinephelus morio	Red Grouper	N	Brought on Board (Retained/Discarded)	Live - Healthy	Discarded - Live and Healthy (Vented)	Not Applicable	
29	UCAM	Epinephelus morio	Red Grouper	N	Brought on Board (Retained/Discarded)	Live - Healthy	Retained	Not Applicable	
29	UCAM	Epinephelus morio	Red Grouper	N	Brought on Board (Retained/Discarded)	Live - Healthy	Discarded - Live and Healthy (Vented)	Not Applicable	
29	UCAM	Epinephelus morio	Red Grouper	N	Brought on Board (Retained/Discarded)	Live - Healthy	Retained	Not Applicable	
29	UCAM	Epinephelus morio	Red Grouper	N	Brought on Board (Retained/Discarded)	Live - Healthy	Retained	Not Applicable	

29	UCAM	Epinephelus morio	Red Grouper	N	Brought on Board (Retained/Discarded)	Live - Healthy	Retained	Not Applicable	
29	UCAM	Epinephelus morio	Red Grouper	N	Brought on Board (Retained/Discarded)	Live - Healthy	Discarded - Live and Healthy (Vented)	Not Applicable	
29	UCAM	Epinephelus morio	Red Grouper	N	Brought on Board (Retained/Discarded)	Live - Healthy	Retained	Not Applicable	
29	UCAM	Epinephelus morio	Red Grouper	N	Brought on Board (Retained/Discarded)	Live - Healthy	Discarded - Live and Healthy (Vented)	Not Applicable	
29	UCAM	Epinephelus morio	Red Grouper	N	Brought on Board (Retained/Discarded)	Live - Healthy	Discarded - Live and Healthy (Vented)	Not Applicable	
29	UCAM	Epinephelus morio	Red Grouper	N	Brought on Board (Retained/Discarded)	Live - Healthy	Discarded - Live and Healthy (Vented)	Not Applicable	
29	UCAM	Epinephelus morio	Red Grouper	N	Brought on Board (Retained/Discarded)	Live - Healthy	Retained	Not Applicable	
29	UCAM	Epinephelus morio	Red Grouper	N	Brought on Board (Retained/Discarded)	Live - Healthy	Discarded - Live and Healthy (Vented)	Not Applicable	
29	UCAM	Epinephelus morio	Red Grouper	N	Brought on Board (Retained/Discarded)	Live - Healthy	Discarded - Live and Healthy (Vented)	Not Applicable	
29	UCAM	Epinephelus morio	Red Grouper	N	Brought on Board (Retained/Discarded)	Live - Healthy	Retained	Not Applicable	
29	UCAM	Epinephelus morio	Red Grouper	N	Brought on Board (Retained/Discarded)	Live - Healthy	Retained	Not Applicable	
29	UCAM	Rhizoprionodon terraenovae	Atlantic Sharpnose Shark	Y	Brought on Board (Retained/Discarded)	Live - Healthy	Discarded - Live and Healthy (Not Vented)	Male (Claspers)	Small (Less than 1m)
29	UCAM	Rhizoprionodon terraenovae	Atlantic Sharpnose Shark	Y	Brought on Board (Retained/Discarded)	Dead on Arrival - Undamaged	Discarded - Dead	Unknown Maturity and/or Sex	Small (Less than 1m)
29	UCAM	Rhizoprionodon terraenovae	Atlantic Sharpnose Shark	Y	Brought on Board (Retained/Discarded)	Live - Healthy	Discarded - Live and Healthy (Not Vented)	Female	Small (Less than 1m)
29	UCAM	Rhizoprionodon terraenovae	Atlantic Sharpnose Shark	Y	Brought on Board (Retained/Discarded)	Live - Healthy	Discarded - Live and Healthy (Not Vented)	Female	Small (Less than 1m)
29	UCAM	Sphyrna lewini	Scalloped Hammerhead	Y	Cut-off at Rail (No Entanglement)	Live - Healthy	Discarded - Live and Healthy (Not Vented)	Unknown Maturity and/or Sex	Medium (Between 1m and 2m)
30	NOCAM	Anthozoa spp.	Coral	N	Brought on Board (Retained/Discarded)	Unknown Condition	Discarded - Unknown	Not Applicable	
30	NOCAM	Carcharhinus acronotus	Blacknose Shark	Y	Brought on Board (Retained/Discarded)	Live - Healthy	Discarded - Live and Healthy (Not Vented)	Female	Small (Less than 1m)
30	NOCAM	Carcharhinus acronotus	Blacknose Shark	Y	Brought on Board (Retained/Discarded)	Live - Healthy	Discarded - Live and Healthy (Not Vented)	Female	Small (Less than 1m)
30	NOCAM	Carcharhinus acronotus	Blacknose Shark	Y	Brought on Board (Retained/Discarded)	Live - Healthy	Discarded - Live and Healthy (Not Vented)	Female	Small (Less than 1m)
30	NOCAM	Carcharhinus acronotus	Blacknose Shark	Y	Brought on Board (Retained/Discarded)	Live - Healthy	Discarded - Live and Healthy (Not Vented)	Female	Small (Less than 1m)
30	NOCAM	Carcharhinus acronotus	Blacknose Shark	Y	Brought on Board (Retained/Discarded)	Live - Healthy	Discarded - Live and Healthy (Not Vented)	Unknown Maturity and/or Sex	Small (Less than 1m)
30	NOCAM	Carcharhinus plumbeus	Sandbar Shark	Y	Cut-off at Rail (No Entanglement)	Live - Healthy	Discarded - Live and Healthy (Not Vented)	Male (Claspers)	Large (Greater than 2m)
30	NOCAM	Epinephelus morio	Red Grouper	N	Brought on Board (Retained/Discarded)	Live - Healthy	Retained	Not Applicable	
30	NOCAM	Epinephelus morio	Red Grouper	N	Brought on Board (Retained/Discarded)	Live - Healthy	Discarded - Live and Healthy (Vented)	Not Applicable	
30	NOCAM	Epinephelus morio	Red Grouper	N	Brought on Board (Retained/Discarded)	Live - Healthy	Discarded - Live and Healthy (Vented)	Not Applicable	
30	NOCAM	Epinephelus morio	Red Grouper	N	Brought on Board (Retained/Discarded)	Live - Healthy	Discarded - Live and Healthy (Vented)	Not Applicable	
30	NOCAM	Epinephelus morio	Red Grouper	N	Brought on Board (Retained/Discarded)	Live - Healthy	Discarded - Live and Damaged (Not Ventec	Not Applicable	
30	NOCAM	Epinephelus morio	Red Grouper	N	Brought on Board (Retained/Discarded)	Live - Healthy	Discarded - Live and Healthy (Vented)	Not Applicable	
30	NOCAM	Epinephelus morio	Red Grouper	N	Brought on Board (Retained/Discarded)	Live - Healthy	Discarded - Live and Healthy (Vented)	Not Applicable	
30	NOCAM	Epinephelus morio	Red Grouper	N	Brought on Board (Retained/Discarded)	Live - Healthy	Retained	Not Applicable	
30	NOCAM	Epinephelus morio	Red Grouper	N	Brought on Board (Retained/Discarded)	Live - Healthy	Discarded - Live and Healthy (Vented)	Not Applicable	
30	NOCAM	Epinephelus morio	Red Grouper	N	Brought on Board (Retained/Discarded)	Live - Healthy	Retained	Not Applicable	
30	NOCAM	Epinephelus morio	Red Grouper	N	Brought on Board (Retained/Discarded)	Live - Healthy	Discarded - Live and Healthy (Vented)	Not Applicable	
30	NOCAM	Epinephelus morio	Red Grouper	N	Brought on Board (Retained/Discarded)	Live - Stomach and/or Eyes Protruding	Discarded - Live and Healthy (Vented)	Not Applicable	
30	NOCAM	Epinephelus morio	Red Grouper	N	Brought on Board (Retained/Discarded)	Live - Healthy	Retained	Not Applicable	
30	NOCAM	Epinephelus morio	Red Grouper	N	Brought on Board (Retained/Discarded)	Live - Healthy	Discarded - Live and Healthy (Vented)	Not Applicable	
30	NOCAM	Epinephelus morio	Red Grouper	N	Brought on Board (Retained/Discarded)	Live - Healthy	Retained	Not Applicable	
30	NOCAM	Epinephelus morio	Red Grouper	N	Brought on Board (Retained/Discarded)	Live - Healthy	Discarded - Live and Healthy (Vented)	Not Applicable	
30	NOCAM	Epinephelus morio	Red Grouper	N	Brought on Board (Retained/Discarded)	Live - Healthy	Discarded - Live and Healthy (Vented)	Not Applicable	
30	NOCAM	Epinephelus morio	Red Grouper	N	Brought on Board (Retained/Discarded)	Live - Healthy	Discarded - Live and Healthy (Vented)	Not Applicable	
30	NOCAM	Epinephelus morio	Red Grouper	N	Brought on Board (Retained/Discarded)	Live - Healthy	Discarded - Live and Healthy (Vented)	Not Applicable	
30	NOCAM	Epinephelus morio	Red Grouper	N	Brought on Board (Retained/Discarded)	Live - Healthy	Retained	Not Applicable	
30	NOCAM	Epinephelus morio	Red Grouper	N	Brought on Board (Retained/Discarded)	Live - Healthy	Discarded - Live and Healthy (Vented)	Not Applicable	
30	NOCAM	Epinephelus morio	Red Grouper	N	Brought on Board (Retained/Discarded)	Live - Healthy	Retained	Not Applicable	
30	NOCAM	Epinephelus morio	Red Grouper	N	Brought on Board (Retained/Discarded)	Live - Healthy	Discarded - Live and Healthy (Vented)	Not Applicable	
30	NOCAM	Epinephelus morio	Red Grouper	N	Brought on Board (Retained/Discarded)	Live - Healthy	Discarded - Live and Healthy (Vented)	Not Applicable	
30	NOCAM	Epinephelus morio	Red Grouper	N	Brought on Board (Retained/Discarded)	Live - Healthy	Discarded - Live and Healthy (Vented)	Not Applicable	
30	NOCAM	Rhizoprionodon terraenovae	Atlantic Sharpnose Shark	Y	Brought on Board (Retained/Discarded)	Live - Healthy	Discarded - Live and Healthy (Not Vented)	Female	Small (Less than 1m)
30	NOCAM	Rhizoprionodon terraenovae	Atlantic Sharpnose Shark	Y	Brought on Board (Retained/Discarded)	Live - Healthy	Discarded - Live and Healthy (Not Vented)	Male (Claspers)	Small (Less than 1m)
30	NOCAM	Unidentified Bottom Debris	Unidentified Bottom Debris	N	Brought on Board (Retained/Discarded)	Unknown Condition	Discarded - Unknown	Not Applicable	
30	UCAM	Anthozoa spp.	Coral	N	Brought on Board (Retained/Discarded)	Unknown Condition	Discarded - Unknown	Not Applicable	
30	UCAM	Carcharhinus acronotus	Blacknose Shark	Y	Brought on Board (Retained/Discarded)	Live - Healthy	Discarded - Live and Healthy (Not Vented)	Female	Small (Less than 1m)
30	UCAM	Carcharhinus acronotus	Blacknose Shark	Y	Brought on Board (Retained/Discarded)	Live - Healthy	Discarded - Live and Healthy (Not Vented)	Unknown Maturity and/or Sex	Small (Less than 1m)
30	UCAM	Carcharhinus acronotus	Blacknose Shark	Y	Brought on Board (Retained/Discarded)	Live - Healthy	Discarded - Live and Healthy (Not Vented)	Female	Small (Less than 1m)
30	UCAM	Carcharhinus acronotus	Blacknose Shark	Y	Brought on Board (Retained/Discarded)	Live - Healthy	Discarded - Live and Healthy (Not Vented)	Female	Small (Less than 1m)
30	UCAM	Carcharhinus acronotus	Blacknose Shark	Y	Brought on Board (Retained/Discarded)	Live - Healthy	Discarded - Live and Healthy (Not Vented)	Female	Small (Less than 1m)
30	UCAM	Carcharhinus plumbeus	Sandbar Shark	Y	Cut-off at Rail (No Entanglement)	Live - Healthy	Discarded - Live and Healthy (Not Vented)	Male (Claspers)	Large (Greater than 2m)
30	UCAM	Epinephelus morio	Red Grouper	N	Brought on Board (Retained/Discarded)	Live - Healthy	Discarded - Live and Healthy (Vented)	Not Applicable	
30	UCAM	Epinephelus morio	Red Grouper	N	Brought on Board (Retained/Discarded)	Live - Healthy	Discarded - Live and Healthy (Vented)	Not Applicable	
30	UCAM	Epinephelus morio	Red Grouper	N	Brought on Board (Retained/Discarded)	Live - Healthy	Discarded - Live and Healthy (Vented)	Not Applicable	
30	UCAM	Epinephelus morio	Red Grouper	N	Brought on Board (Retained/Discarded)	Live - Healthy	Retained	Not Applicable	
30	UCAM	Epinephelus morio	Red Grouper	N	Brought on Board (Retained/Discarded)	Live - Healthy	Discarded - Live and Healthy (Vented)	Not Applicable	
30	UCAM	Epinephelus morio	Red Grouper	N	Brought on Board (Retained/Discarded)	Live - Healthy	Retained	Not Applicable	
30	UCAM	Epinephelus morio	Red Grouper	N	Brought on Board (Retained/Discarded)	Live - Healthy	Discarded - Live and Healthy (Vented)	Not Applicable	
30	UCAM	Epinephelus morio	Red Grouper	N	Brought on Board (Retained/Discarded)	Live - Healthy	Discarded - Live and Healthy (Vented)	Not Applicable	
30	UCAM	Epinephelus morio	Red Grouper	N	Brought on Board (Retained/Discarded)	Live - Healthy	Discarded - Live and Healthy (Vented)	Not Applicable	
30	UCAM	Epinephelus morio	Red Grouper	N	Brought on Board (Retained/Discarded)	Live - Healthy	Discarded - Live and Healthy (Vented)	Not Applicable	
30	UCAM	Epinephelus morio	Red Grouper	N	Brought on Board (Retained/Discarded)	Live - Healthy	Discarded - Live and Healthy (Vented)	Not Applicable	
30	UCAM	Epinephelus morio	Red Grouper	N	Brought on Board (Retained/Discarded)	Live - Stomach and/or Eyes Protruding	Discarded - Live and Healthy (Vented)	Not Applicable	
30	UCAM	Epinephelus morio	Red Grouper	N	Brought on Board (Retained/Discarded)	Live - Healthy	Retained	Not Applicable	
30	UCAM	Epinephelus morio	Red Grouper	N	Brought on Board (Retained/Discarded)	Live - Healthy	Retained	Not Applicable	
30	UCAM	Epinephelus morio	Red Grouper	N	Brought on Board (Retained/Discarded)	Live - Healthy	Discarded - Live and Healthy (Vented)	Not Applicable	
30	UCAM	Epinephelus morio	Red Grouper	N	Brought on Board (Retained/Discarded)	Live - Stomach and/or Eyes Protruding	Discarded - Live and Healthy (Vented)	Not Applicable	
30	UCAM	Epinephelus morio	Red Grouper	N	Brought on Board (Retained/Discarded)	Live - Healthy	Retained	Not Applicable	
30	UCAM	Epinephelus morio	Red Grouper	N	Brought on Board (Retained/Discarded)	Live - Stomach and/or Eyes Protruding	Discarded - Live and Healthy (Vented)	Not Applicable	
30	UCAM	Epinephelus morio	Red Grouper	N	Brought on Board (Retained/Discarded)	Live - Healthy	Discarded - Live and Healthy (Vented)	Not Applicable	
30	UCAM	Epinephelus morio	Red Grouper	N	Brought on Board (Retained/Discarded)	Live - Healthy	Retained	Not Applicable	
30	UCAM	Epinephelus morio	Red Grouper	N	Brought on Board (Retained/Discarded)	Live - Healthy	Discarded - Live and Healthy (Vented)	Not Applicable	
30	UCAM	Epinephelus morio	Red Grouper	N	Brought on Board (Retained/Discarded)	Live - Stomach and/or Eyes Protruding	Discarded - Live and Healthy (Vented)	Not Applicable	
30	UCAM	Epinephelus morio	Red Grouper	N	Brought on Board (Retained/Discarded)	Live - Healthy	Discarded - Live and Healthy (Vented)	Not Applicable	
30	UCAM	Rhizoprionodon terraenovae	Atlantic Sharpnose Shark	Y	Brought on Board (Retained/Discarded)	Live - Healthy	Discarded - Live and Healthy (Not Vented)	Female	Small (Less than 1m)
30	UCAM	Rhizoprionodon terraenovae	Atlantic Sharpnose Shark	Y	Brought on Board (Retained/Discarded)	Live - Healthy	Discarded - Live and Healthy (Not Vented)	Male (Claspers)	Small (Less than 1m)
30	UCAM	Unidentified Bottom Debris	Unidentified Bottom Debris	N	Brought on Board (Retained/Discarded)	Unknown Condition	Discarded - Unknown	Not Applicable	
31	NOCAM	Carcharhinus acronotus	Blacknose Shark	Y	Brought on Board (Retained/Discarded)	Live - Healthy	Discarded - Live and Healthy (Not Vented)	Female	Medium (Between 1m and 2m)
31	NOCAM	Carcharhinus acronotus	Blacknose Shark	Y	Brought on Board (Retained/Discarded)	Live - Healthy	Discarded - Live and Healthy (Not Vented)	Known Adult - Undetermined Sex	Medium (Between 1m and 2m)
31	NOCAM	Carcharhinus acronotus	Blacknose Shark	Y	Cut-off at Rail (No Entanglement)	Live - Healthy	Discarded - Live and Healthy (Not Vented)	Unknown Maturity and/or Sex	Medium (Between 1m and 2m)
31	NOCAM	Carcharhinus acronotus	Blacknose Shark	Y	Brought on Board (Retained/Discarded)	Live - Healthy	Discarded - Live and Healthy (Not Vented)	Unknown Maturity and/or Sex	Small (Less than 1m)
31	NOCAM	Carcharhinus plumbeus	Sandbar Shark	Y	Cut-off at Rail (No Entanglement)	Live - Healthy	Discarded - Live and Healthy (Not Vented)	Known Adult - Undetermined Sex	Large (Greater than 2m)
31	NOCAM	Carcharhinus plumbeus	Sandbar Shark	Y	Cut-off at Rail (No Entanglement)	Live - Healthy	Discarded - Live and Healthy (Not Vented)	Known Adult - Undetermined Sex	Large (Greater than 2m)
31	NOCAM	Epinephelus morio	Red Grouper	N	Brought on Board (Retained/Discarded)	Live - Healthy	Discarded - Live and Healthy (Vented)	Not Applicable	
31	NOCAM	Epinephelus morio	Red Grouper	N	Brought on Board (Retained/Discarded)	Live - Healthy	Retained	Not Applicable	

31	NOCAM	Epinephelus morio	Red Grouper	N	Brought on Board (Retained/Discarded)	Live - Stomach and/or Eyes Protruding	Discarded - Live and Healthy (Not Vented)	Not Applicable	
31	NOCAM	Epinephelus morio	Red Grouper	N	Brought on Board (Retained/Discarded)	Live - Healthy	Retained	Not Applicable	
31	NOCAM	Epinephelus morio	Red Grouper	N	Brought on Board (Retained/Discarded)	Live - Healthy	Discarded - Live and Healthy (Vented)	Not Applicable	
31	NOCAM	Epinephelus morio	Red Grouper	N	Brought on Board (Retained/Discarded)	Live - Healthy	Discarded - Live and Healthy (Vented)	Not Applicable	
31	NOCAM	Epinephelus morio	Red Grouper	N	Brought on Board (Retained/Discarded)	Live - Healthy	Discarded - Live and Healthy (Vented)	Not Applicable	
31	NOCAM	Epinephelus morio	Red Grouper	N	Brought on Board (Retained/Discarded)	Live - Stomach and/or Eyes Protruding	Discarded - Live and Healthy (Vented)	Not Applicable	
31	NOCAM	Epinephelus morio	Red Grouper	N	Brought on Board (Retained/Discarded)	Live - Healthy	Discarded - Live and Healthy (Vented)	Not Applicable	
31	NOCAM	Epinephelus morio	Red Grouper	N	Brought on Board (Retained/Discarded)	Live - Healthy	Discarded - Live and Healthy (Vented)	Not Applicable	
31	NOCAM	Epinephelus morio	Red Grouper	N	Brought on Board (Retained/Discarded)	Live - Healthy	Retained	Not Applicable	
31	NOCAM	Epinephelus morio	Red Grouper	N	Brought on Board (Retained/Discarded)	Live - Healthy	Retained	Not Applicable	
31	NOCAM	Epinephelus morio	Red Grouper	N	Brought on Board (Retained/Discarded)	Live - Healthy	Retained	Not Applicable	
31	NOCAM	Epinephelus morio	Red Grouper	N	Brought on Board (Retained/Discarded)	Live - Healthy	Retained	Not Applicable	
31	NOCAM	Epinephelus morio	Red Grouper	N	Brought on Board (Retained/Discarded)	Live - Healthy	Discarded - Live and Healthy (Vented)	Not Applicable	
31	NOCAM	Epinephelus morio	Red Grouper	N	Brought on Board (Retained/Discarded)	Live - Stomach and/or Eyes Protruding	Discarded - Live and Healthy (Vented)	Not Applicable	
31	NOCAM	Epinephelus morio	Red Grouper	N	Brought on Board (Retained/Discarded)	Live - Healthy	Discarded - Live and Healthy (Vented)	Not Applicable	
31	NOCAM	Epinephelus morio	Red Grouper	N	Brought on Board (Retained/Discarded)	Live - Stomach and/or Eyes Protruding	Retained	Not Applicable	
31	NOCAM	Epinephelus morio	Red Grouper	N	Brought on Board (Retained/Discarded)	Live - Healthy	Discarded - Live and Healthy (Vented)	Not Applicable	
31	NOCAM	Epinephelus morio	Red Grouper	N	Brought on Board (Retained/Discarded)	Live - Healthy	Discarded - Live and Healthy (Vented)	Not Applicable	
31	NOCAM	Epinephelus morio	Red Grouper	N	Brought on Board (Retained/Discarded)	Live - Healthy	Retained	Not Applicable	
31	NOCAM	Epinephelus morio	Red Grouper	N	Brought on Board (Retained/Discarded)	Live - Healthy	Discarded - Live and Healthy (Vented)	Not Applicable	
31	NOCAM	Epinephelus morio	Red Grouper	N	Brought on Board (Retained/Discarded)	Live - Healthy	Retained	Not Applicable	
31	NOCAM	Epinephelus morio	Red Grouper	N	Brought on Board (Retained/Discarded)	Live - Healthy	Retained	Not Applicable	
31	NOCAM	Epinephelus morio	Red Grouper	N	Brought on Board (Retained/Discarded)	Live - Stomach and/or Eyes Protruding	Retained	Not Applicable	
31	NOCAM	Epinephelus morio	Red Grouper	N	Brought on Board (Retained/Discarded)	Live - Stomach and/or Eyes Protruding	Discarded - Live and Healthy (Vented)	Not Applicable	
31	NOCAM	Epinephelus morio	Red Grouper	N	Brought on Board (Retained/Discarded)	Live - Stomach and/or Eyes Protruding	Retained	Not Applicable	
31	NOCAM	Epinephelus morio	Red Grouper	N	Brought on Board (Retained/Discarded)	Live - Stomach and/or Eyes Protruding	Retained	Not Applicable	
31	NOCAM	Epinephelus morio	Red Grouper	N	Brought on Board (Retained/Discarded)	Live - Healthy	Discarded - Live and Healthy (Vented)	Not Applicable	
31	NOCAM	Epinephelus morio	Red Grouper	N	Brought on Board (Retained/Discarded)	Live - Healthy	Retained	Not Applicable	
31	NOCAM	Epinephelus morio	Red Grouper	N	Brought on Board (Retained/Discarded)	Dead on Arrival - Undamaged	Discarded - Dead	Not Applicable	
31	NOCAM	Epinephelus morio	Red Grouper	N	Brought on Board (Retained/Discarded)	Live - Healthy	Discarded - Live and Healthy (Vented)	Not Applicable	
31	NOCAM	Lutjanus campechanus	Red Snapper	N	Brought on Board (Retained/Discarded)	Live - Stomach and/or Eyes Protruding	Discarded - Live and Healthy (Vented)	Not Applicable	
31	NOCAM	Lutjanus campechanus	Red Snapper	N	Brought on Board (Retained/Discarded)	Live - Healthy	Retained	Not Applicable	
31	NOCAM	Lutjanus campechanus	Red Snapper	N	Brought on Board (Retained/Discarded)	Live - Healthy	Discarded - Live and Healthy (Vented)	Not Applicable	
31	NOCAM	Lutjanus griseus	Gray (Mangrove) Snapper	N	Brought on Board (Retained/Discarded)	Live - Stomach and/or Eyes Protruding	Retained	Not Applicable	
31	NOCAM	Rhizoprionodon terraenovae	Atlantic Sharpnose Shark	Y	Brought on Board (Retained/Discarded)	Live - Healthy	Discarded - Live and Healthy (Not Vented)	Unknown Maturity and/or Sex	Small (Less than 1m)
31	NOCAM	Rhizoprionodon terraenovae	Atlantic Sharpnose Shark	Y	Brought on Board (Retained/Discarded)	Live - Healthy	Discarded - Live and Healthy (Not Vented)	Female	Small (Less than 1m)
31	NOCAM	Rhizoprionodon terraenovae	Atlantic Sharpnose Shark	Y	Brought on Board (Retained/Discarded)	Live - Healthy	Discarded - Live and Healthy (Not Vented)	Male (Claspers)	Small (Less than 1m)
31	NOCAM	Rhizoprionodon terraenovae	Atlantic Sharpnose Shark	Y	Brought on Board (Retained/Discarded)	Live - Healthy	Discarded - Live and Healthy (Not Vented)	Female	Small (Less than 1m)
31	NOCAM	Rhizoprionodon terraenovae	Atlantic Sharpnose Shark	Y	Brought on Board (Retained/Discarded)	Live - Healthy	Discarded - Live and Healthy (Not Vented)	Female	Small (Less than 1m)
31	NOCAM	Rhizoprionodon terraenovae	Atlantic Sharpnose Shark	Y	Brought on Board (Retained/Discarded)	Live - Healthy	Discarded - Live and Healthy (Not Vented)	Unknown Maturity and/or Sex	Small (Less than 1m)
31	NOCAM	Rhizoprionodon terraenovae	Atlantic Sharpnose Shark	Y	Brought on Board (Retained/Discarded)	Live - Healthy	Discarded - Live and Healthy (Not Vented)	Female	Small (Less than 1m)
31	NOCAM	Rhizoprionodon terraenovae	Atlantic Sharpnose Shark	Y	Brought on Board (Retained/Discarded)	Live - Healthy	Discarded - Live and Healthy (Not Vented)	Male (Claspers)	Small (Less than 1m)
31	NOCAM	Rhizoprionodon terraenovae	Atlantic Sharpnose Shark	Y	Brought on Board (Retained/Discarded)	Live - Healthy	Discarded - Live and Healthy (Not Vented)	Female	Small (Less than 1m)
31	NOCAM	Rhizoprionodon terraenovae	Atlantic Sharpnose Shark	Y	Cut-off at Rail (No Entanglement)	Live - Healthy	Discarded - Live and Healthy (Not Vented)	Female	Small (Less than 1m)
31	NOCAM	Rhizoprionodon terraenovae	Atlantic Sharpnose Shark	Y	Brought on Board (Retained/Discarded)	Live - Healthy	Discarded - Live and Healthy (Not Vented)	Female	Small (Less than 1m)
31	NOCAM	Rhizoprionodon terraenovae	Atlantic Sharpnose Shark	Y	Brought on Board (Retained/Discarded)	Live - Healthy	Discarded - Live and Healthy (Not Vented)	Male (Claspers)	Small (Less than 1m)
31	NOCAM	Rhizoprionodon terraenovae	Atlantic Sharpnose Shark	Y	Brought on Board (Retained/Discarded)	Live - Healthy	Discarded - Live and Healthy (Not Vented)	Female	Small (Less than 1m)
31	UCAM	Carcharhinus acronotus	Blacknose Shark	Y	Cut-off at Rail (No Entanglement)	Live - Healthy	Discarded - Live and Healthy (Not Vented)	Unknown Maturity and/or Sex	Medium (Between 1m and 2m)
31	UCAM	Carcharhinus acronotus	Blacknose Shark	Y	Brought on Board (Retained/Discarded)	Live - Healthy	Discarded - Live and Healthy (Not Vented)	Female	Medium (Between 1m and 2m)
31	UCAM	Carcharhinus acronotus	Blacknose Shark	Y	Brought on Board (Retained/Discarded)	Live - Healthy	Discarded - Live and Healthy (Not Vented)	Unknown Maturity and/or Sex	Small (Less than 1m)
31	UCAM	Carcharhinus acronotus	Blacknose Shark	Y	Brought on Board (Retained/Discarded)	Live - Healthy	Discarded - Live and Healthy (Not Vented)	Known Adult - Undetermined Sex	Medium (Between 1m and 2m)
31	UCAM	Carcharhinus plumbeus	Sandbar Shark	Y	Cut-off at Rail (No Entanglement)	Live - Healthy	Discarded - Live and Healthy (Not Vented)	Male (Claspers)	Large (Greater than 2m)
31	UCAM	Epinephelus morio	Red Grouper	N	Brought on Board (Retained/Discarded)	Live - Healthy	Discarded - Live and Healthy (Vented)	Not Applicable	
31	UCAM	Epinephelus morio	Red Grouper	N	Brought on Board (Retained/Discarded)	Live - Healthy	Retained	Not Applicable	
31	UCAM	Epinephelus morio	Red Grouper	N	Brought on Board (Retained/Discarded)	Live - Healthy	Retained	Not Applicable	
31	UCAM	Epinephelus morio	Red Grouper	N	Brought on Board (Retained/Discarded)	Live - Stomach and/or Eyes Protruding	Discarded - Live and Healthy (Vented)	Not Applicable	
31	UCAM	Epinephelus morio	Red Grouper	N	Brought on Board (Retained/Discarded)	Live - Healthy	Discarded - Live and Healthy (Vented)	Not Applicable	
31	UCAM	Epinephelus morio	Red Grouper	N	Brought on Board (Retained/Discarded)	Live - Healthy	Retained	Not Applicable	
31	UCAM	Epinephelus morio	Red Grouper	N	Brought on Board (Retained/Discarded)	Live - Healthy	Discarded - Live and Healthy (Vented)	Not Applicable	
31	UCAM	Epinephelus morio	Red Grouper	N	Brought on Board (Retained/Discarded)	Live - Stomach and/or Eyes Protruding	Retained	Not Applicable	
31	UCAM	Epinephelus morio	Red Grouper	N	Brought on Board (Retained/Discarded)	Live - Healthy	Retained	Not Applicable	
31	UCAM	Epinephelus morio	Red Grouper	N	Brought on Board (Retained/Discarded)	Live - Healthy	Discarded - Live and Healthy (Vented)	Not Applicable	
31	UCAM	Epinephelus morio	Red Grouper	N	Brought on Board (Retained/Discarded)	Live - Stomach and/or Eyes Protruding	Discarded - Live and Healthy (Vented)	Not Applicable	
31	UCAM	Epinephelus morio	Red Grouper	N	Brought on Board (Retained/Discarded)	Live - Healthy	Discarded - Live and Healthy (Vented)	Not Applicable	
31	UCAM	Epinephelus morio	Red Grouper	N	Brought on Board (Retained/Discarded)	Live - Stomach and/or Eyes Protruding	Retained	Not Applicable	
31	UCAM	Epinephelus morio	Red Grouper	N	Brought on Board (Retained/Discarded)	Live - Healthy	Retained	Not Applicable	
31	UCAM	Epinephelus morio	Red Grouper	N	Brought on Board (Retained/Discarded)	Live - Healthy	Discarded - Live and Healthy (Vented)	Not Applicable	
31	UCAM	Epinephelus morio	Red Grouper	N	Brought on Board (Retained/Discarded)	Live - Healthy	Discarded - Live and Healthy (Vented)	Not Applicable	
31	UCAM	Epinephelus morio	Red Grouper	N	Brought on Board (Retained/Discarded)	Live - Healthy	Discarded - Live and Healthy (Vented)	Not Applicable	
31	UCAM	Epinephelus morio	Red Grouper	N	Brought on Board (Retained/Discarded)	Live - Healthy	Retained	Not Applicable	
31	UCAM	Epinephelus morio	Red Grouper	N	Brought on Board (Retained/Discarded)	Live - Healthy	Discarded - Live and Healthy (Vented)	Not Applicable	
31	UCAM	Epinephelus morio	Red Grouper	N	Brought on Board (Retained/Discarded)	Dead on Arrival - Undamaged	Discarded - Dead	Not Applicable	
31	UCAM	Epinephelus morio	Red Grouper	N	Brought on Board (Retained/Discarded)	Live - Healthy	Retained	Not Applicable	
31	UCAM	Epinephelus morio	Red Grouper	N	Brought on Board (Retained/Discarded)	Live - Healthy	Discarded - Live and Healthy (Vented)	Not Applicable	
31	UCAM	Epinephelus morio	Red Grouper	N	Brought on Board (Retained/Discarded)	Live - Healthy	Retained	Not Applicable	
31	UCAM	Epinephelus morio	Red Grouper	N	Brought on Board (Retained/Discarded)	Live - Healthy	Retained	Not Applicable	
31	UCAM	Epinephelus morio	Red Grouper	N	Brought on Board (Retained/Discarded)	Live - Stomach and/or Eyes Protruding	Discarded - Live and Healthy (Vented)	Not Applicable	
31	UCAM	Epinephelus morio	Red Grouper	N	Brought on Board (Retained/Discarded)	Live - Stomach and/or Eyes Protruding	Discarded - Live and Healthy (Vented)	Not Applicable	
31	UCAM	Epinephelus morio	Red Grouper	N	Brought on Board (Retained/Discarded)	Live - Stomach and/or Eyes Protruding	Discarded - Live and Healthy (Vented)	Not Applicable	
31	UCAM	Epinephelus morio	Red Grouper	N	Brought on Board (Retained/Discarded)	Live - Stomach and/or Eyes Protruding	Retained	Not Applicable	
31	UCAM	Lutjanus campechanus	Red Snapper	N	Brought on Board (Retained/Discarded)	Live - Healthy	Discarded - Live and Healthy (Vented)	Not Applicable	
31	UCAM	Lutjanus campechanus	Red Snapper	N	Brought on Board (Retained/Discarded)	Live - Stomach and/or Eyes Protruding	Discarded - Live and Healthy (Vented)	Not Applicable	
31	UCAM	Lutjanus campechanus	Red Snapper	N	Brought on Board (Retained/Discarded)	Live - Healthy	Retained	Not Applicable	
31	UCAM	Lutjanus griseus	Gray (Mangrove) Snapper	N	Brought on Board (Retained/Discarded)	Live - Stomach and/or Eyes Protruding	Retained	Not Applicable	
31	UCAM	Rhizoprionodon terraenovae	Atlantic Sharpnose Shark	Y	Brought on Board (Retained/Discarded)	Live - Healthy	Discarded - Live and Healthy (Not Vented)	Male (Claspers)	Small (Less than 1m)
31	UCAM	Rhizoprionodon terraenovae	Atlantic Sharpnose Shark	Y	Brought on Board (Retained/Discarded)	Live - Healthy	Discarded - Live and Healthy (Not Vented)	Female	Small (Less than 1m)
31	UCAM	Rhizoprionodon terraenovae	Atlantic Sharpnose Shark	Y	Brought on Board (Retained/Discarded)	Live - Healthy	Discarded - Live and Healthy (Not Vented)	Female	Small (Less than 1m)
31	UCAM	Rhizoprionodon terraenovae	Atlantic Sharpnose Shark	Y	Brought on Board (Retained/Discarded)	Live - Healthy	Discarded - Live and Healthy (Not Vented)	Unknown Maturity and/or Sex	Small (Less than 1m)
31	UCAM	Rhizoprionodon terraenovae	Atlantic Sharpnose Shark	Y	Brought on Board (Retained/Discarded)	Live - Healthy	Discarded - Live and Healthy (Not Vented)	Unknown Maturity and/or Sex	Small (Less than 1m)
31	UCAM	Rhizoprionodon terraenovae	Atlantic Sharpnose Shark	Y	Brought on Board (Retained/Discarded)	Live - Healthy	Discarded - Live and Healthy (Not Vented)	Female	Small (Less than 1m)
31	UCAM	Rhizoprionodon terraenovae	Atlantic Sharpnose Shark	Y	Brought on Board (Retained/Discarded)	Live - Healthy	Discarded - Live and Healthy (Not Vented)	Female	Small (Less than 1m)
31	UCAM	Rhizoprionodon terraenovae	Atlantic Sharpnose Shark	Y	Brought on Board (Retained/Discarded)	Live - Healthy	Discarded - Live and Healthy (Not Vented)	Female	Small (Less than 1m)

31	UCAM	Rhizoprionodon terraenovae	Atlantic Sharpnose Shark	Y	Brought on Board (Retained/Discarded)	Live - Healthy	Discarded - Live and Healthy (Not Vented)	Male (Claspers)	Small (Less than 1m)
31	UCAM	Rhizoprionodon terraenovae	Atlantic Sharpnose Shark	Y	Brought on Board (Retained/Discarded)	Live - Healthy	Discarded - Live and Healthy (Not Vented)	Female	Small (Less than 1m)
31	UCAM	Rhizoprionodon terraenovae	Atlantic Sharpnose Shark	Y	Brought on Board (Retained/Discarded)	Live - Healthy	Discarded - Live and Healthy (Not Vented)	Female	Small (Less than 1m)
31	UCAM	Rhizoprionodon terraenovae	Atlantic Sharpnose Shark	Y	Brought on Board (Retained/Discarded)	Live - Healthy	Discarded - Live and Healthy (Not Vented)	Unknown Maturity and/or Sex	Small (Less than 1m)
31	UCAM	Rhizoprionodon terraenovae	Atlantic Sharpnose Shark	Y	Brought on Board (Retained/Discarded)	Live - Healthy	Discarded - Live and Healthy (Not Vented)	Male (Claspers)	Small (Less than 1m)
32	NOCAM	Epinephelus morio	Red Grouper	N	Brought on Board (Retained/Discarded)	Live - Stomach and/or Eyes Protruding	Discarded - Live and Healthy (Vented)	Not Applicable	
32	NOCAM	Epinephelus morio	Red Grouper	N	Brought on Board (Retained/Discarded)	Live - Healthy	Retained	Not Applicable	
32	NOCAM	Epinephelus morio	Red Grouper	N	Brought on Board (Retained/Discarded)	Live - Stomach and/or Eyes Protruding	Retained	Not Applicable	
32	NOCAM	Epinephelus morio	Red Grouper	N	Brought on Board (Retained/Discarded)	Live - Healthy	Discarded - Live and Healthy (Vented)	Not Applicable	
32	NOCAM	Epinephelus morio	Red Grouper	N	Brought on Board (Retained/Discarded)	Live - Healthy	Discarded - Live and Healthy (Vented)	Not Applicable	
32	NOCAM	Epinephelus morio	Red Grouper	N	Brought on Board (Retained/Discarded)	Live - Healthy	Retained	Not Applicable	
32	NOCAM	Epinephelus morio	Red Grouper	N	Brought on Board (Retained/Discarded)	Live - Stomach and/or Eyes Protruding	Discarded - Live and Healthy (Vented)	Not Applicable	
32	NOCAM	Epinephelus morio	Red Grouper	N	Brought on Board (Retained/Discarded)	Live - Healthy	Retained	Not Applicable	
32	NOCAM	Epinephelus morio	Red Grouper	N	Brought on Board (Retained/Discarded)	Live - Healthy	Discarded - Live and Healthy (Vented)	Not Applicable	
32	NOCAM	Epinephelus morio	Red Grouper	N	Brought on Board (Retained/Discarded)	Live - Stomach and/or Eyes Protruding	Retained	Not Applicable	
32	NOCAM	Epinephelus morio	Red Grouper	N	Brought on Board (Retained/Discarded)	Live - Healthy	Discarded - Live and Healthy (Vented)	Not Applicable	
32	NOCAM	Epinephelus morio	Red Grouper	N	Brought on Board (Retained/Discarded)	Live - Stomach and/or Eyes Protruding	Retained	Not Applicable	
32	NOCAM	Epinephelus morio	Red Grouper	N	Brought on Board (Retained/Discarded)	Live - Healthy	Retained	Not Applicable	
32	NOCAM	Epinephelus morio	Red Grouper	N	Brought on Board (Retained/Discarded)	Live - Healthy	Discarded - Live and Healthy (Vented)	Not Applicable	
32	NOCAM	Epinephelus morio	Red Grouper	N	Brought on Board (Retained/Discarded)	Live - Stomach and/or Eyes Protruding	Retained	Not Applicable	
32	NOCAM	Epinephelus morio	Red Grouper	N	Brought on Board (Retained/Discarded)	Live - Stomach and/or Eyes Protruding	Discarded - Live and Healthy (Vented)	Not Applicable	
32	NOCAM	Epinephelus morio	Red Grouper	N	Brought on Board (Retained/Discarded)	Live - Healthy	Discarded - Live and Healthy (Vented)	Not Applicable	
32	NOCAM	Epinephelus morio	Red Grouper	N	Brought on Board (Retained/Discarded)	Live - Stomach and/or Eyes Protruding	Retained	Not Applicable	
32	NOCAM	Epinephelus morio	Red Grouper	N	Brought on Board (Retained/Discarded)	Live - Stomach and/or Eyes Protruding	Discarded - Live and Healthy (Vented)	Not Applicable	
32	NOCAM	Epinephelus morio	Red Grouper	N	Brought on Board (Retained/Discarded)	Live - Healthy	Discarded - Live and Healthy (Vented)	Not Applicable	
32	NOCAM	Epinephelus morio	Red Grouper	N	Brought on Board (Retained/Discarded)	Live - Healthy	Discarded - Live and Healthy (Vented)	Not Applicable	
32	NOCAM	Epinephelus morio	Red Grouper	N	Brought on Board (Retained/Discarded)	Live - Stomach and/or Eyes Protruding	Discarded - Live and Healthy (Vented)	Not Applicable	
32	NOCAM	Epinephelus morio	Red Grouper	N	Brought on Board (Retained/Discarded)	Live - Healthy	Retained	Not Applicable	
32	NOCAM	Epinephelus morio	Red Grouper	N	Brought on Board (Retained/Discarded)	Live - Stomach and/or Eyes Protruding	Discarded - Live and Healthy (Vented)	Not Applicable	
32	NOCAM	Epinephelus morio	Red Grouper	N	Brought on Board (Retained/Discarded)	Live - Healthy	Discarded - Live and Healthy (Vented)	Not Applicable	
32	NOCAM	Epinephelus morio	Red Grouper	N	Brought on Board (Retained/Discarded)	Live - Healthy	Discarded - Live and Healthy (Vented)	Not Applicable	
32	NOCAM	Epinephelus morio	Red Grouper	N	Brought on Board (Retained/Discarded)	Live - Healthy	Retained	Not Applicable	
32	NOCAM	Epinephelus morio	Red Grouper	N	Brought on Board (Retained/Discarded)	Live - Healthy	Discarded - Live and Healthy (Vented)	Not Applicable	
32	NOCAM	Epinephelus morio	Red Grouper	N	Brought on Board (Retained/Discarded)	Live - Stomach and/or Eyes Protruding	Discarded - Live and Healthy (Vented)	Not Applicable	
32	NOCAM	Epinephelus morio	Red Grouper	N	Brought on Board (Retained/Discarded)	Live - Stomach and/or Eyes Protruding	Discarded - Live and Healthy (Vented)	Not Applicable	
32	NOCAM	Epinephelus morio	Red Grouper	N	Brought on Board (Retained/Discarded)	Live - Stomach and/or Eyes Protruding	Retained	Not Applicable	
32	NOCAM	Epinephelus morio	Red Grouper	N	Brought on Board (Retained/Discarded)	Live - Stomach and/or Eyes Protruding	Retained	Not Applicable	
32	NOCAM	Epinephelus morio	Red Grouper	N	Brought on Board (Retained/Discarded)	Live - Stomach and/or Eyes Protruding	Discarded - Live and Healthy (Vented)	Not Applicable	
32	NOCAM	Epinephelus morio	Red Grouper	N	Brought on Board (Retained/Discarded)	Live - Stomach and/or Eyes Protruding	Retained	Not Applicable	
32	NOCAM	Epinephelus morio	Red Grouper	N	Brought on Board (Retained/Discarded)	Live - Stomach and/or Eyes Protruding	Retained	Not Applicable	
32	NOCAM	Epinephelus morio	Red Grouper	N	Brought on Board (Retained/Discarded)	Live - Healthy	Retained	Not Applicable	
32	NOCAM	Epinephelus morio	Red Grouper	N	Brought on Board (Retained/Discarded)	Live - Stomach and/or Eyes Protruding	Discarded - Live and Healthy (Vented)	Not Applicable	
32	NOCAM	Epinephelus morio	Red Grouper	N	Brought on Board (Retained/Discarded)	Live - Healthy	Discarded - Live and Healthy (Vented)	Not Applicable	
32	NOCAM	Epinephelus morio	Red Grouper	N	Brought on Board (Retained/Discarded)	Live - Healthy	Discarded - Live and Healthy (Vented)	Not Applicable	
32	NOCAM	Epinephelus morio	Red Grouper	N	Brought on Board (Retained/Discarded)	Live - Healthy	Discarded - Live and Healthy (Not Vented)	Not Applicable	
32	NOCAM	Epinephelus morio	Red Grouper	N	Brought on Board (Retained/Discarded)	Live - Stomach and/or Eyes Protruding	Discarded - Live and Healthy (Vented)	Not Applicable	
32	NOCAM	Epinephelus morio	Red Grouper	N	Brought on Board (Retained/Discarded)	Live - Healthy	Discarded - Live and Healthy (Vented)	Not Applicable	
32	NOCAM	Epinephelus morio	Red Grouper	N	Brought on Board (Retained/Discarded)	Live - Stomach and/or Eyes Protruding	Retained	Not Applicable	
32	NOCAM	Epinephelus morio	Red Grouper	N	Brought on Board (Retained/Discarded)	Live - Healthy	Discarded - Live and Healthy (Vented)	Not Applicable	
32	NOCAM	Epinephelus morio	Red Grouper	N	Brought on Board (Retained/Discarded)	Live - Stomach and/or Eyes Protruding	Discarded - Live and Healthy (Vented)	Not Applicable	
32	NOCAM	Epinephelus morio	Red Grouper	N	Brought on Board (Retained/Discarded)	Live - Stomach and/or Eyes Protruding	Discarded - Live and Healthy (Vented)	Not Applicable	
32	NOCAM	Epinephelus morio	Red Grouper	N	Brought on Board (Retained/Discarded)	Live - Healthy	Discarded - Live and Healthy (Vented)	Not Applicable	
32	NOCAM	Epinephelus morio	Red Grouper	N	Brought on Board (Retained/Discarded)	Live - Stomach and/or Eyes Protruding	Retained	Not Applicable	
32	NOCAM	Epinephelus morio	Red Grouper	N	Brought on Board (Retained/Discarded)	Live - Healthy	Retained	Not Applicable	
32	NOCAM	Epinephelus morio	Red Grouper	N	Brought on Board (Retained/Discarded)	Live - Stomach and/or Eyes Protruding	Discarded - Live and Healthy (Vented)	Not Applicable	
32	NOCAM	Epinephelus morio	Red Grouper	N	Brought on Board (Retained/Discarded)	Live - Stomach and/or Eyes Protruding	Retained	Not Applicable	
32	NOCAM	Epinephelus morio	Red Grouper	N	Brought on Board (Retained/Discarded)	Live - Healthy	Discarded - Live and Healthy (Vented)	Not Applicable	
32	NOCAM	Epinephelus morio	Red Grouper	N	Brought on Board (Retained/Discarded)	Live - Stomach and/or Eyes Protruding	Retained	Not Applicable	
32	NOCAM	Epinephelus morio	Red Grouper	N	Brought on Board (Retained/Discarded)	Live - Stomach and/or Eyes Protruding	Discarded - Live and Healthy (Vented)	Not Applicable	
32	NOCAM	Epinephelus morio	Red Grouper	N	Brought on Board (Retained/Discarded)	Live - Healthy	Discarded - Live and Healthy (Vented)	Not Applicable	
32	NOCAM	Lutjanus campechanus	Red Snapper	N	Brought on Board (Retained/Discarded)	Live - Healthy	Discarded - Live and Healthy (Vented)	Not Applicable	
32	NOCAM	Lutjanus campechanus	Red Snapper	N	Brought on Board (Retained/Discarded)	Live - Stomach and/or Eyes Protruding	Discarded - Live and Healthy (Vented)	Not Applicable	
32	NOCAM	Mycteroperca microlepis	Gag Grouper	N	Brought on Board (Retained/Discarded)	Live - Healthy	Retained	Not Applicable	
32	NOCAM	Opsanus spp.	Toadfish (all)	N	Brought on Board (Retained/Discarded)	Live - Healthy	Retained as Bait	Not Applicable	
32	NOCAM	Rhizoprionodon terraenovae	Atlantic Sharpnose Shark	Y	Brought on Board (Retained/Discarded)	Live - Healthy	Discarded - Live and Healthy (Not Vented)	Female	Small (Less than 1m)
32	NOCAM	Rhizoprionodon terraenovae	Atlantic Sharpnose Shark	Y	Brought on Board (Retained/Discarded)	Live - Healthy	Discarded - Live and Healthy (Not Vented)	Unknown Maturity and/or Sex	Small (Less than 1m)
32	NOCAM	Tetraodontidae spp.	Pufferfish (all)	N	Brought on Board (Retained/Discarded)	Live - Healthy	Retained as Bait	Not Applicable	
32	UCAM	Epinephelus morio	Red Grouper	N	Brought on Board (Retained/Discarded)	Live - Healthy	Discarded - Live and Healthy (Vented)	Not Applicable	
32	UCAM	Epinephelus morio	Red Grouper	N	Brought on Board (Retained/Discarded)	Live - Stomach and/or Eyes Protruding	Retained	Not Applicable	
32	UCAM	Epinephelus morio	Red Grouper	N	Brought on Board (Retained/Discarded)	Live - Stomach and/or Eyes Protruding	Retained	Not Applicable	
32	UCAM	Epinephelus morio	Red Grouper	N	Brought on Board (Retained/Discarded)	Live - Healthy	Discarded - Live and Healthy (Vented)	Not Applicable	
32	UCAM	Epinephelus morio	Red Grouper	N	Brought on Board (Retained/Discarded)	Live - Healthy	Discarded - Live and Healthy (Vented)	Not Applicable	
32	UCAM	Epinephelus morio	Red Grouper	N	Brought on Board (Retained/Discarded)	Live - Healthy	Discarded - Live and Healthy (Vented)	Not Applicable	
32	UCAM	Epinephelus morio	Red Grouper	N	Brought on Board (Retained/Discarded)	Live - Healthy	Discarded - Live and Healthy (Vented)	Not Applicable	
32	UCAM	Epinephelus morio	Red Grouper	N	Brought on Board (Retained/Discarded)	Live - Healthy	Discarded - Live and Healthy (Vented)	Not Applicable	
32	UCAM	Epinephelus morio	Red Grouper	N	Brought on Board (Retained/Discarded)	Live - Stomach and/or Eyes Protruding	Discarded - Live and Healthy (Vented)	Not Applicable	
32	UCAM	Epinephelus morio	Red Grouper	N	Brought on Board (Retained/Discarded)	Live - Stomach and/or Eyes Protruding	Retained	Not Applicable	
32	UCAM	Epinephelus morio	Red Grouper	N	Brought on Board (Retained/Discarded)	Live - Healthy	Discarded - Live and Healthy (Vented)	Not Applicable	
32	UCAM	Epinephelus morio	Red Grouper	N	Brought on Board (Retained/Discarded)	Live - Stomach and/or Eyes Protruding	Retained	Not Applicable	
32	UCAM	Epinephelus morio	Red Grouper	N	Brought on Board (Retained/Discarded)	Live - Healthy	Discarded - Live and Healthy (Vented)	Not Applicable	
32	UCAM	Epinephelus morio	Red Grouper	N	Brought on Board (Retained/Discarded)	Live - Healthy	Retained	Not Applicable	
32	UCAM	Epinephelus morio	Red Grouper	N	Brought on Board (Retained/Discarded)	Live - Healthy	Discarded - Live and Healthy (Vented)	Not Applicable	
32	UCAM	Epinephelus morio	Red Grouper	N	Brought on Board (Retained/Discarded)	Live - Stomach and/or Eyes Protruding	Retained	Not Applicable	
32	UCAM	Epinephelus morio	Red Grouper	N	Brought on Board (Retained/Discarded)	Live - Healthy	Discarded - Live and Healthy (Vented)	Not Applicable	
32	UCAM	Epinephelus morio	Red Grouper	N	Brought on Board (Retained/Discarded)	Live - Stomach and/or Eyes Protruding	Discarded - Live and Healthy (Vented)	Not Applicable	
32	UCAM	Epinephelus morio	Red Grouper	N	Brought on Board (Retained/Discarded)	Live - Healthy	Retained	Not Applicable	
32	UCAM	Epinephelus morio	Red Grouper	N	Brought on Board (Retained/Discarded)	Live - Stomach and/or Eyes Protruding	Retained	Not Applicable	
32	UCAM	Epinephelus morio	Red Grouper	N	Brought on Board (Retained/Discarded)	Live - Stomach and/or Eyes Protruding	Retained	Not Applicable	
32	UCAM	Epinephelus morio	Red Grouper	N	Brought on Board (Retained/Discarded)	Live - Stomach and/or Eyes Protruding	Retained	Not Applicable	
32	UCAM	Epinephelus morio	Red Grouper	N	Brought on Board (Retained/Discarded)	Live - Stomach and/or Eyes Protruding	Retained	Not Applicable	
32	UCAM	Epinephelus morio	Red Grouper	N	Brought on Board (Retained/Discarded)	Live - Healthy	Discarded - Live and Healthy (Vented)	Not Applicable	
32	UCAM	Epinephelus morio	Red Grouper	N	Brought on Board (Retained/Discarded)	Live - Healthy	Discarded - Live and Healthy (Vented)	Not Applicable	

32	UCAM	Epinephelus morio	Red Grouper	N	Brought on Board (Retained/Discarded)	Live - Stomach and/or Eyes Protruding	Retained	Not Applicable	
32	UCAM	Epinephelus morio	Red Grouper	N	Brought on Board (Retained/Discarded)	Live - Healthy	Retained	Not Applicable	
32	UCAM	Epinephelus morio	Red Grouper	N	Brought on Board (Retained/Discarded)	Live - Stomach and/or Eyes Protruding	Retained	Not Applicable	
32	UCAM	Epinephelus morio	Red Grouper	N	Brought on Board (Retained/Discarded)	Live - Stomach and/or Eyes Protruding	Discarded - Live and Healthy (Vented)	Not Applicable	
32	UCAM	Epinephelus morio	Red Grouper	N	Brought on Board (Retained/Discarded)	Live - Healthy	Retained	Not Applicable	
32	UCAM	Epinephelus morio	Red Grouper	N	Brought on Board (Retained/Discarded)	Live - Healthy	Retained	Not Applicable	
32	UCAM	Epinephelus morio	Red Grouper	N	Brought on Board (Retained/Discarded)	Live - Healthy	Discarded - Live and Healthy (Vented)	Not Applicable	
32	UCAM	Epinephelus morio	Red Grouper	N	Brought on Board (Retained/Discarded)	Live - Healthy	Discarded - Live and Healthy (Vented)	Not Applicable	
32	UCAM	Epinephelus morio	Red Grouper	N	Brought on Board (Retained/Discarded)	Live - Stomach and/or Eyes Protruding	Discarded - Live and Healthy (Vented)	Not Applicable	
32	UCAM	Epinephelus morio	Red Grouper	N	Brought on Board (Retained/Discarded)	Live - Healthy	Discarded - Live and Healthy (Vented)	Not Applicable	
32	UCAM	Epinephelus morio	Red Grouper	N	Brought on Board (Retained/Discarded)	Live - Stomach and/or Eyes Protruding	Discarded - Live and Healthy (Vented)	Not Applicable	
32	UCAM	Epinephelus morio	Red Grouper	N	Brought on Board (Retained/Discarded)	Live - Healthy	Discarded - Live and Healthy (Vented)	Not Applicable	
32	UCAM	Epinephelus morio	Red Grouper	N	Brought on Board (Retained/Discarded)	Live - Stomach and/or Eyes Protruding	Discarded - Live and Healthy (Vented)	Not Applicable	
32	UCAM	Epinephelus morio	Red Grouper	N	Brought on Board (Retained/Discarded)	Live - Healthy	Discarded - Live and Healthy (Vented)	Not Applicable	
32	UCAM	Epinephelus morio	Red Grouper	N	Brought on Board (Retained/Discarded)	Live - Healthy	Discarded - Live and Healthy (Vented)	Not Applicable	
32	UCAM	Epinephelus morio	Red Grouper	N	Brought on Board (Retained/Discarded)	Live - Healthy	Discarded - Live and Healthy (Vented)	Not Applicable	
32	UCAM	Epinephelus morio	Red Grouper	N	Brought on Board (Retained/Discarded)	Live - Healthy	Retained	Not Applicable	
32	UCAM	Epinephelus morio	Red Grouper	N	Brought on Board (Retained/Discarded)	Live - Healthy	Discarded - Live and Healthy (Vented)	Not Applicable	
32	UCAM	Epinephelus morio	Red Grouper	N	Brought on Board (Retained/Discarded)	Live - Stomach and/or Eyes Protruding	Retained	Not Applicable	
32	UCAM	Epinephelus morio	Red Grouper	N	Brought on Board (Retained/Discarded)	Live - Healthy	Discarded - Live and Healthy (Vented)	Not Applicable	
32	UCAM	Epinephelus morio	Red Grouper	N	Brought on Board (Retained/Discarded)	Live - Healthy	Discarded - Live and Healthy (Vented)	Not Applicable	
32	UCAM	Epinephelus morio	Red Grouper	N	Brought on Board (Retained/Discarded)	Live - Healthy	Retained	Not Applicable	
32	UCAM	Epinephelus morio	Red Grouper	N	Brought on Board (Retained/Discarded)	Live - Stomach and/or Eyes Protruding	Retained	Not Applicable	
32	UCAM	Epinephelus morio	Red Grouper	N	Brought on Board (Retained/Discarded)	Live - Stomach and/or Eyes Protruding	Retained	Not Applicable	
32	UCAM	Epinephelus morio	Red Grouper	N	Brought on Board (Retained/Discarded)	Live - Healthy	Discarded - Live and Healthy (Vented)	Not Applicable	
32	UCAM	Epinephelus morio	Red Grouper	N	Brought on Board (Retained/Discarded)	Live - Healthy	Discarded - Live and Healthy (Vented)	Not Applicable	
32	UCAM	Epinephelus morio	Red Grouper	N	Brought on Board (Retained/Discarded)	Live - Healthy	Retained	Not Applicable	
32	UCAM	Epinephelus morio	Red Grouper	N	Brought on Board (Retained/Discarded)	Live - Healthy	Retained	Not Applicable	
32	UCAM	Epinephelus morio	Red Grouper	N	Brought on Board (Retained/Discarded)	Live - Healthy	Retained	Not Applicable	
32	UCAM	Epinephelus morio	Red Grouper	N	Brought on Board (Retained/Discarded)	Live - Stomach and/or Eyes Protruding	Retained	Not Applicable	
32	UCAM	Epinephelus morio	Red Grouper	N	Brought on Board (Retained/Discarded)	Live - Stomach and/or Eyes Protruding	Discarded - Live and Healthy (Vented)	Not Applicable	
32	UCAM	Lutjanus campechanus	Red Snapper	N	Brought on Board (Retained/Discarded)	Live - Healthy	Discarded - Live and Healthy (Vented)	Not Applicable	
32	UCAM	Lutjanus campechanus	Red Snapper	N	Brought on Board (Retained/Discarded)	Live - Stomach and/or Eyes Protruding	Discarded - Live and Healthy (Vented)	Not Applicable	
32	UCAM	Mycteroperca microlepis	Gag Grouper	N	Brought on Board (Retained/Discarded)	Live - Healthy	Retained	Not Applicable	
32	UCAM	Rhizoprionodon terraenovae	Atlantic Sharpnose Shark	Y	Brought on Board (Retained/Discarded)	Live - Healthy	Discarded - Live and Healthy (Not Vented)	Female	Small (Less than 1m)
32	UCAM	Tetraodontidae spp.	Pufferfish (all)	N	Brought on Board (Retained/Discarded)	Live - Healthy	Retained as Bait	Not Applicable	
33	NOCAM	Coryphaena hippurus	Dolphin Fish (Mahi mahi)	N	Brought on Board (Retained/Discarded)	Dead on Arrival - Undamaged	Retained	Not Applicable	
33	NOCAM	Epinephelus flavolimbatus	Yellowedge Grouper	N	Brought on Board (Retained/Discarded)	Live - Stomach and/or Eyes Protruding	Retained	Not Applicable	
33	NOCAM	Epinephelus nigritus	Warsaw Grouper	N	Brought on Board (Retained/Discarded)	Live - Healthy	Retained	Not Applicable	
33	NOCAM	Epinephelus niveatus	Snowy Grouper	N	Brought on Board (Retained/Discarded)	Live - Healthy	Discarded - Live and Healthy (Not Vented)	Not Applicable	
33	NOCAM	Epinephelus niveatus	Snowy Grouper	N	Brought on Board (Retained/Discarded)	Live - Healthy	Retained	Not Applicable	
33	NOCAM	Epinephelus niveatus	Snowy Grouper	N	Brought on Board (Retained/Discarded)	Live - Healthy	Discarded - Live and Healthy (Vented)	Not Applicable	
33	NOCAM	Epinephelus niveatus	Snowy Grouper	N	Brought on Board (Retained/Discarded)	Live - Stomach and/or Eyes Protruding	Retained	Not Applicable	
33	NOCAM	Gadiformes, unidentified	Hake, Unidentified	N	Brought on Board (Retained/Discarded)	Live - Stomach and/or Eyes Protruding	Retained as Bait	Not Applicable	
33	NOCAM	Gadiformes, unidentified	Hake, Unidentified	N	Brought on Board (Retained/Discarded)	Live - Stomach and/or Eyes Protruding	Retained as Bait	Not Applicable	
33	NOCAM	Gadiformes, unidentified	Hake, Unidentified	N	Brought on Board (Retained/Discarded)	Live - Healthy	Retained as Bait	Not Applicable	
33	NOCAM	Gadiformes, unidentified	Hake, Unidentified	N	Brought on Board (Retained/Discarded)	Live - Stomach and/or Eyes Protruding	Retained as Bait	Not Applicable	
33	NOCAM	Muraenidae, unidentified	Moray Eel, Unidentified	N	Brought on Board (Retained/Discarded)	Live - Healthy	Retained as Bait	Not Applicable	
33	NOCAM	Muraenidae, unidentified	Moray Eel, Unidentified	N	Brought on Board (Retained/Discarded)	Live - Healthy	Retained as Bait	Not Applicable	
33	NOCAM	Muraenidae, unidentified	Moray Eel, Unidentified	N	Brought on Board (Retained/Discarded)	Live - Healthy	Retained as Bait	Not Applicable	
33	NOCAM	Muraenidae, unidentified	Moray Eel, Unidentified	N	Brought on Board (Retained/Discarded)	Live - Healthy	Retained as Bait	Not Applicable	
33	NOCAM	Muraenidae, unidentified	Moray Eel, Unidentified	N	Brought on Board (Retained/Discarded)	Live - Healthy	Retained as Bait	Not Applicable	
33	NOCAM	Mustelus spp.	Dogfish, Smooth (Florida)	Y	Brought on Board (Retained/Discarded)	Live - Healthy	Discarded - Live and Healthy (Not Vented)	Male (Claspers)	Small (Less than 1m)
33	NOCAM	Mustelus spp.	Dogfish, Smooth (Florida)	Y	Brought on Board (Retained/Discarded)	Live - Healthy	Discarded - Live and Healthy (Not Vented)	Male (Claspers)	Small (Less than 1m)
33	NOCAM	Mustelus spp.	Dogfish, Smooth (Florida)	Y	Brought on Board (Retained/Discarded)	Live - Healthy	Discarded - Live and Healthy (Not Vented)	Male (Claspers)	Medium (Between 1m and 2m)
33	NOCAM	Mustelus spp.	Dogfish, Smooth (Florida)	Y	Brought on Board (Retained/Discarded)	Live - Healthy	Discarded - Live and Healthy (Not Vented)	Male (Claspers)	Medium (Between 1m and 2m)
33	NOCAM	Mustelus spp.	Dogfish, Smooth (Florida)	Y	Brought on Board (Retained/Discarded)	Live - Healthy	Discarded - Live and Healthy (Not Vented)	Female	Small (Less than 1m)
33	NOCAM	Mycteroperca microlepis	Gag Grouper	N	Brought on Board (Retained/Discarded)	Live - Healthy	Retained	Not Applicable	
33	NOCAM	Mycteroperca microlepis	Gag Grouper	N	Brought on Board (Retained/Discarded)	Live - Healthy	Retained	Not Applicable	
33	NOCAM	Neomerinthe hemingwayi	Scorpionfish, Spiny Cheek	N	Brought on Board (Retained/Discarded)	Live - Stomach and/or Eyes Protruding	Retained	Not Applicable	
33	NOCAM	Neomerinthe hemingwayi	Scorpionfish, Spiny Cheek	N	Brought on Board (Retained/Discarded)	Live - Stomach and/or Eyes Protruding	Retained	Not Applicable	
33	NOCAM	Selachimorpha, unidentified	Shark, Unidentified	Y	Brought on Board (Retained/Discarded)	Live - Healthy	Discarded - Live and Healthy (Not Vented)	Unknown Maturity and/or Sex	Medium (Between 1m and 2m)
33	NOCAM	Synodus spp.	Lizardfish (all)	N	Brought on Board (Retained/Discarded)	Live - Healthy	Retained as Bait	Not Applicable	
33	UCAM	Coryphaena hippurus	Dolphin Fish (Mahi mahi)	N	Brought on Board (Retained/Discarded)	Dead on Arrival - Undamaged	Retained	Not Applicable	
33	UCAM	Epinephelus flavolimbatus	Yellowedge Grouper	N	Brought on Board (Retained/Discarded)	Live - Stomach and/or Eyes Protruding	Retained	Not Applicable	
33	UCAM	Epinephelus nigritus	Warsaw Grouper	N	Brought on Board (Retained/Discarded)	Live - Stomach and/or Eyes Protruding	Retained	Not Applicable	
33	UCAM	Epinephelus niveatus	Snowy Grouper	N	Brought on Board (Retained/Discarded)	Live - Healthy	Retained	Not Applicable	
33	UCAM	Epinephelus niveatus	Snowy Grouper	N	Brought on Board (Retained/Discarded)	Live - Healthy	Discarded - Live and Healthy (Vented)	Not Applicable	
33	UCAM	Epinephelus niveatus	Snowy Grouper	N	Brought on Board (Retained/Discarded)	Live - Stomach and/or Eyes Protruding	Retained	Not Applicable	
33	UCAM	Epinephelus niveatus	Snowy Grouper	N	Brought on Board (Retained/Discarded)	Live - Healthy	Discarded - Live and Healthy (Vented)	Not Applicable	
33	UCAM	Gadiformes, unidentified	Hake, Unidentified	N	Brought on Board (Retained/Discarded)	Live - Stomach and/or Eyes Protruding	Retained as Bait	Not Applicable	
33	UCAM	Gadiformes, unidentified	Hake, Unidentified	N	Brought on Board (Retained/Discarded)	Live - Healthy	Retained as Bait	Not Applicable	
33	UCAM	Gadiformes, unidentified	Hake, Unidentified	N	Brought on Board (Retained/Discarded)	Live - Stomach and/or Eyes Protruding	Retained as Bait	Not Applicable	
33	UCAM	Gadiformes, unidentified	Hake, Unidentified	N	Brought on Board (Retained/Discarded)	Live - Stomach and/or Eyes Protruding	Retained as Bait	Not Applicable	
33	UCAM	Muraenidae, unidentified	Moray Eel, Unidentified	N	Brought on Board (Retained/Discarded)	Live - Healthy	Retained as Bait	Not Applicable	
33	UCAM	Muraenidae, unidentified	Moray Eel, Unidentified	N	Brought on Board (Retained/Discarded)	Live - Healthy	Retained as Bait	Not Applicable	
33	UCAM	Muraenidae, unidentified	Moray Eel, Unidentified	N	Brought on Board (Retained/Discarded)	Live - Healthy	Retained as Bait	Not Applicable	
33	UCAM	Muraenidae, unidentified	Moray Eel, Unidentified	N	Brought on Board (Retained/Discarded)	Live - Healthy	Retained as Bait	Not Applicable	
33	UCAM	Muraenidae, unidentified	Moray Eel, Unidentified	N	Brought on Board (Retained/Discarded)	Live - Healthy	Retained as Bait	Not Applicable	
33	UCAM	Mustelus spp.	Dogfish, Smooth (Florida)	Y	Brought on Board (Retained/Discarded)	Live - Healthy	Discarded - Live and Healthy (Not Vented)	Female	Small (Less than 1m)
33	UCAM	Mustelus spp.	Dogfish, Smooth (Florida)	Y	Brought on Board (Retained/Discarded)	Live - Healthy	Discarded - Live and Healthy (Not Vented)	Male (Claspers)	Small (Less than 1m)
33	UCAM	Mustelus spp.	Dogfish, Smooth (Florida)	Y	Brought on Board (Retained/Discarded)	Live - Healthy	Discarded - Live and Healthy (Not Vented)	Male (Claspers)	Medium (Between 1m and 2m)
33	UCAM	Mustelus spp.	Dogfish, Smooth (Florida)	Y	Brought on Board (Retained/Discarded)	Live - Healthy	Discarded - Live and Healthy (Not Vented)	Male (Claspers)	Small (Less than 1m)
33	UCAM	Mustelus spp.	Dogfish, Smooth (Florida)	Y	Brought on Board (Retained/Discarded)	Live - Healthy	Discarded - Live and Healthy (Not Vented)	Male (Claspers)	Medium (Between 1m and 2m)
33	UCAM	Mycteroperca microlepis	Gag Grouper	N	Brought on Board (Retained/Discarded)	Live - Healthy	Retained	Not Applicable	
33	UCAM	Mycteroperca microlepis	Gag Grouper	N	Brought on Board (Retained/Discarded)	Live - Healthy	Retained	Not Applicable	
33	UCAM	Neomerinthe hemingwayi	Scorpionfish, Spiny Cheek	N	Brought on Board (Retained/Discarded)	Live - Stomach and/or Eyes Protruding	Retained	Not Applicable	
33	UCAM	Neomerinthe hemingwayi	Scorpionfish, Spiny Cheek	N	Brought on Board (Retained/Discarded)	Live - Stomach and/or Eyes Protruding	Retained	Not Applicable	
33	UCAM	Selachimorpha, unidentified	Shark, Unidentified	Y	Brought on Board (Retained/Discarded)	Live - Healthy	Discarded - Live and Healthy (Not Vented)	Unknown Maturity and/or Sex	Medium (Between 1m and 2m)
33	UCAM	Synodus spp.	Lizardfish (all)	N	Brought on Board (Retained/Discarded)	Dead on Arrival - Undamaged	Retained as Bait	Not Applicable	
34	NOCAM	Epinephelus flavolimbatus	Yellowedge Grouper	N	Brought on Board (Retained/Discarded)	Live - Stomach and/or Eyes Protruding	Retained	Not Applicable	
34	NOCAM	Epinephelus flavolimbatus	Yellowedge Grouper	N	Brought on Board (Retained/Discarded)	Live - Stomach and/or Eyes Protruding	Retained	Not Applicable	

34	NOCAM	Epinephelus flavolimbatus	Yellowedge Grouper	N	Brought on Board (Retained/Discarded)	Live - Stomach and/or Eyes Protruding	Retained	Not Applicable	
34	NOCAM	Epinephelus flavolimbatus	Yellowedge Grouper	N	Brought on Board (Retained/Discarded)	Live - Stomach and/or Eyes Protruding	Retained	Not Applicable	
34	NOCAM	Epinephelus niveatus	Snowy Grouper	N	Brought on Board (Retained/Discarded)	Live - Stomach and/or Eyes Protruding	Retained	Not Applicable	
34	NOCAM	Epinephelus niveatus	Snowy Grouper	N	Brought on Board (Retained/Discarded)	Live - Stomach and/or Eyes Protruding	Retained	Not Applicable	
34	NOCAM	Epinephelus niveatus	Snowy Grouper	N	Brought on Board (Retained/Discarded)	Live - Healthy	Retained	Not Applicable	
34	NOCAM	Epinephelus niveatus	Snowy Grouper	N	Brought on Board (Retained/Discarded)	Live - Stomach and/or Eyes Protruding	Retained	Not Applicable	
34	NOCAM	Epinephelus niveatus	Snowy Grouper	N	Brought on Board (Retained/Discarded)	Live - Stomach and/or Eyes Protruding	Retained	Not Applicable	
34	NOCAM	Epinephelus niveatus	Snowy Grouper	N	Brought on Board (Retained/Discarded)	Live - Stomach and/or Eyes Protruding	Retained	Not Applicable	
34	NOCAM	Epinephelus niveatus	Snowy Grouper	N	Brought on Board (Retained/Discarded)	Live - Healthy	Retained	Not Applicable	
34	NOCAM	Epinephelus niveatus	Snowy Grouper	N	Brought on Board (Retained/Discarded)	Live - Stomach and/or Eyes Protruding	Retained	Not Applicable	
34	NOCAM	Muraenidae, unidentified	Moray Eel, Unidentified	N	Brought on Board (Retained/Discarded)	Live - Healthy	Retained as Bait	Not Applicable	
34	NOCAM	Muraenidae, unidentified	Moray Eel, Unidentified	N	Brought on Board (Retained/Discarded)	Live - Healthy	Retained as Bait	Not Applicable	
34	NOCAM	Muraenidae, unidentified	Moray Eel, Unidentified	N	Brought on Board (Retained/Discarded)	Live - Healthy	Retained as Bait	Not Applicable	
34	NOCAM	Mustelus spp.	Dogfish, Smooth (Florida)	Y	Brought on Board (Retained/Discarded)	Live - Healthy	Discarded - Live and Healthy (Not Vented)	Female	Medium (Between 1m and 2m)
34	NOCAM	Mustelus spp.	Dogfish, Smooth (Florida)	Y	Brought on Board (Retained/Discarded)	Live - Healthy	Discarded - Live and Healthy (Not Vented)	Male (Claspers)	Small (Less than 1m)
34	NOCAM	Mustelus spp.	Dogfish, Smooth (Florida)	Y	Brought on Board (Retained/Discarded)	Live - Healthy	Discarded - Live and Healthy (Not Vented)	Male (Claspers)	Medium (Between 1m and 2m)
34	NOCAM	Mustelus spp.	Dogfish, Smooth (Florida)	Y	Brought on Board (Retained/Discarded)	Live - Healthy	Discarded - Live and Healthy (Not Vented)	Male (Claspers)	Medium (Between 1m and 2m)
34	NOCAM	Mustelus spp.	Dogfish, Smooth (Florida)	Y	Brought on Board (Retained/Discarded)	Live - Healthy	Discarded - Live and Healthy (Not Vented)	Female	Medium (Between 1m and 2m)
34	NOCAM	Mustelus spp.	Dogfish, Smooth (Florida)	Y	Brought on Board (Retained/Discarded)	Live - Healthy	Discarded - Live and Healthy (Not Vented)	Male (Claspers)	Medium (Between 1m and 2m)
34	NOCAM	Mustelus spp.	Dogfish, Smooth (Florida)	Y	Brought on Board (Retained/Discarded)	Live - Healthy	Discarded - Live and Healthy (Not Vented)	Male (Claspers)	Medium (Between 1m and 2m)
34	NOCAM	Mycteroperca microlepis	Gag Grouper	N	Brought on Board (Retained/Discarded)	Live - Healthy	Retained	Not Applicable	
34	NOCAM	Mycteroperca microlepis	Gag Grouper	N	Brought on Board (Retained/Discarded)	Live - Stomach and/or Eyes Protruding	Retained	Not Applicable	
34	NOCAM	Mycteroperca microlepis	Gag Grouper	N	Brought on Board (Retained/Discarded)	Live - Healthy	Retained	Not Applicable	
34	NOCAM	Mycteroperca microlepis	Gag Grouper	N	Brought on Board (Retained/Discarded)	Live - Healthy	Retained	Not Applicable	
34	NOCAM	Mycteroperca microlepis	Gag Grouper	N	Brought on Board (Retained/Discarded)	Live - Healthy	Retained	Not Applicable	
34	NOCAM	Mycteroperca microlepis	Gag Grouper	N	Brought on Board (Retained/Discarded)	Live - Healthy	Retained	Not Applicable	
34	NOCAM	Mycteroperca microlepis	Gag Grouper	N	Brought on Board (Retained/Discarded)	Live - Stomach and/or Eyes Protruding	Retained	Not Applicable	
34	NOCAM	Mycteroperca microlepis	Gag Grouper	N	Brought on Board (Retained/Discarded)	Live - Healthy	Retained	Not Applicable	
34	NOCAM	Mycteroperca microlepis	Gag Grouper	N	Brought on Board (Retained/Discarded)	Live - Healthy	Retained	Not Applicable	
34	NOCAM	Mycteroperca microlepis	Gag Grouper	N	Brought on Board (Retained/Discarded)	Live - Healthy	Retained	Not Applicable	
34	NOCAM	Mycteroperca microlepis	Gag Grouper	N	Brought on Board (Retained/Discarded)	Live - Healthy	Retained	Not Applicable	
34	NOCAM	Mycteroperca microlepis	Gag Grouper	N	Brought on Board (Retained/Discarded)	Live - Healthy	Retained	Not Applicable	
34	NOCAM	Neomerinthe hemingwayi	Scorpionfish, Spiny Cheek	N	Brought on Board (Retained/Discarded)	Live - Healthy	Retained	Not Applicable	
34	NOCAM	Ophichthus rex	King Snake Eel	N	Brought on Board (Retained/Discarded)	Live - Healthy	Retained as Bait	Not Applicable	
34	NOCAM	Seriola dumerili	Greater Amberjack	N	Brought on Board (Retained/Discarded)	Live - Healthy	Discarded - Live and Healthy (Vented)	Not Applicable	
34	NOCAM	Sphyrna lewini	Scalloped Hammerhead	Y	Cut-off at Rail (No Entanglement)	Unknown Condition	Discarded - Unknown	Unknown Maturity and/or Sex	Medium (Between 1m and 2m)
34	NOCAM	Sphyrna lewini	Scalloped Hammerhead	Y	Cut-off at Rail (No Entanglement)	Dead on Arrival - Undamaged	Discarded - Dead	Unknown Maturity and/or Sex	Medium (Between 1m and 2m)
34	UCAM	Anguilliformes, unidentified	Eel, Unidentified	N	Brought on Board (Retained/Discarded)	Live - Healthy	Retained as Bait	Not Applicable	
34	UCAM	Epinephelus flavolimbatus	Yellowedge Grouper	N	Brought on Board (Retained/Discarded)	Live - Stomach and/or Eyes Protruding	Retained	Not Applicable	
34	UCAM	Epinephelus flavolimbatus	Yellowedge Grouper	N	Brought on Board (Retained/Discarded)	Live - Stomach and/or Eyes Protruding	Retained	Not Applicable	
34	UCAM	Epinephelus flavolimbatus	Yellowedge Grouper	N	Brought on Board (Retained/Discarded)	Live - Stomach and/or Eyes Protruding	Retained	Not Applicable	
34	UCAM	Epinephelus flavolimbatus	Yellowedge Grouper	N	Brought on Board (Retained/Discarded)	Live - Stomach and/or Eyes Protruding	Retained	Not Applicable	
34	UCAM	Epinephelus niveatus	Snowy Grouper	N	Brought on Board (Retained/Discarded)	Live - Healthy	Retained	Not Applicable	
34	UCAM	Epinephelus niveatus	Snowy Grouper	N	Brought on Board (Retained/Discarded)	Live - Stomach and/or Eyes Protruding	Retained	Not Applicable	
34	UCAM	Epinephelus niveatus	Snowy Grouper	N	Brought on Board (Retained/Discarded)	Live - Stomach and/or Eyes Protruding	Retained	Not Applicable	
34	UCAM	Epinephelus niveatus	Snowy Grouper	N	Brought on Board (Retained/Discarded)	Live - Healthy	Retained	Not Applicable	
34	UCAM	Epinephelus niveatus	Snowy Grouper	N	Brought on Board (Retained/Discarded)	Live - Stomach and/or Eyes Protruding	Retained	Not Applicable	
34	UCAM	Epinephelus niveatus	Snowy Grouper	N	Brought on Board (Retained/Discarded)	Live - Stomach and/or Eyes Protruding	Retained	Not Applicable	
34	UCAM	Epinephelus niveatus	Snowy Grouper	N	Brought on Board (Retained/Discarded)	Live - Stomach and/or Eyes Protruding	Retained	Not Applicable	
34	UCAM	Epinephelus niveatus	Snowy Grouper	N	Brought on Board (Retained/Discarded)	Live - Stomach and/or Eyes Protruding	Retained	Not Applicable	
34	UCAM	Muraenidae, unidentified	Moray Eel, Unidentified	N	Brought on Board (Retained/Discarded)	Live - Healthy	Retained as Bait	Not Applicable	
34	UCAM	Mustelus spp.	Dogfish, Smooth (Florida)	Y	Brought on Board (Retained/Discarded)	Live - Healthy	Discarded - Live and Healthy (Not Vented)	Male (Claspers)	Medium (Between 1m and 2m)
34	UCAM	Mustelus spp.	Dogfish, Smooth (Florida)	Y	Brought on Board (Retained/Discarded)	Live - Healthy	Discarded - Live and Healthy (Not Vented)	Male (Claspers)	Medium (Between 1m and 2m)
34	UCAM	Mustelus spp.	Dogfish, Smooth (Florida)	Y	Brought on Board (Retained/Discarded)	Live - Healthy	Discarded - Live and Healthy (Not Vented)	Male (Claspers)	Medium (Between 1m and 2m)
34	UCAM	Mustelus spp.	Dogfish, Smooth (Florida)	Y	Brought on Board (Retained/Discarded)	Live - Healthy	Discarded - Live and Healthy (Not Vented)	Male (Claspers)	Medium (Between 1m and 2m)
34	UCAM	Mustelus spp.	Dogfish, Smooth (Florida)	Y	Brought on Board (Retained/Discarded)	Live - Healthy	Discarded - Live and Healthy (Not Vented)	Female	Medium (Between 1m and 2m)
34	UCAM	Mustelus spp.	Dogfish, Smooth (Florida)	Y	Brought on Board (Retained/Discarded)	Live - Healthy	Discarded - Live and Healthy (Not Vented)	Female	Medium (Between 1m and 2m)
34	UCAM	Mustelus spp.	Dogfish, Smooth (Florida)	Y	Brought on Board (Retained/Discarded)	Live - Healthy	Discarded - Live and Healthy (Not Vented)	Male (Claspers)	Small (Less than 1m)
34	UCAM	Mycteroperca microlepis	Gag Grouper	N	Brought on Board (Retained/Discarded)	Live - Healthy	Retained	Not Applicable	
34	UCAM	Mycteroperca microlepis	Gag Grouper	N	Brought on Board (Retained/Discarded)	Live - Healthy	Retained	Not Applicable	
34	UCAM	Mycteroperca microlepis	Gag Grouper	N	Brought on Board (Retained/Discarded)	Live - Healthy	Retained	Not Applicable	
34	UCAM	Mycteroperca microlepis	Gag Grouper	N	Brought on Board (Retained/Discarded)	Live - Healthy	Retained	Not Applicable	
34	UCAM	Mycteroperca microlepis	Gag Grouper	N	Brought on Board (Retained/Discarded)	Live - Healthy	Retained	Not Applicable	
34	UCAM	Mycteroperca microlepis	Gag Grouper	N	Brought on Board (Retained/Discarded)	Live - Healthy	Retained	Not Applicable	
34	UCAM	Mycteroperca microlepis	Gag Grouper	N	Brought on Board (Retained/Discarded)	Live - Healthy	Retained	Not Applicable	
34	UCAM	Mycteroperca microlepis	Gag Grouper	N	Brought on Board (Retained/Discarded)	Live - Healthy	Retained	Not Applicable	
34	UCAM	Mycteroperca microlepis	Gag Grouper	N	Brought on Board (Retained/Discarded)	Live - Healthy	Retained	Not Applicable	
34	UCAM	Mycteroperca microlepis	Gag Grouper	N	Brought on Board (Retained/Discarded)	Live - Healthy	Retained	Not Applicable	
34	UCAM	Mycteroperca microlepis	Gag Grouper	N	Brought on Board (Retained/Discarded)	Live - Healthy	Retained	Not Applicable	
34	UCAM	Mycteroperca microlepis	Gag Grouper	N	Brought on Board (Retained/Discarded)	Live - Stomach and/or Eyes Protruding	Retained	Not Applicable	
34	UCAM	Neomerinthe hemingwayi	Scorpionfish, Spiny Cheek	N	Brought on Board (Retained/Discarded)	Live - Healthy	Retained	Not Applicable	
34	UCAM	Ophichthus rex	King Snake Eel	N	Brought on Board (Retained/Discarded)	Live - Healthy	Retained as Bait	Not Applicable	
34	UCAM	Seriola dumerili	Greater Amberjack	N	Brought on Board (Retained/Discarded)	Live - Healthy	Discarded - Live and Healthy (Vented)	Not Applicable	
34	UCAM	Sphyrna lewini	Scalloped Hammerhead	Y	Cut-off at Rail (No Entanglement)	Dead on Arrival - Undamaged	Discarded - Dead	Female	Medium (Between 1m and 2m)
34	UCAM	Sphyrna lewini	Scalloped Hammerhead	Y	Cut-off at Rail (No Entanglement)	Dead on Arrival - Undamaged	Discarded - Dead	Female	Medium (Between 1m and 2m)
34	UCAM	Squalidae spp.	Dogfish, Spiny (Cuban)	Y	Brought on Board (Retained/Discarded)	Live - Healthy	Discarded - Live and Healthy (Not Vented)	Male (Claspers)	Medium (Between 1m and 2m)
35	NOCAM	Crab, unidentified	Crab, Unidentified	N	Brought on Board (Retained/Discarded)	Live - Healthy	Discarded - Live and Healthy (Not Vented)	Not Applicable	
35	NOCAM	Crab, unidentified	Crab, Unidentified	N	Brought on Board (Retained/Discarded)	Live - Healthy	Discarded - Live and Healthy (Not Vented)	Not Applicable	
35	NOCAM	Mustelus spp.	Dogfish, Smooth (Florida)	Y	Brought on Board (Retained/Discarded)	Live - Healthy	Discarded - Live and Healthy (Not Vented)	Male (Claspers)	Medium (Between 1m and 2m)
35	NOCAM	Ophichthus rex	King Snake Eel	N	Brought on Board (Retained/Discarded)	Live - Healthy	Retained as Bait	Not Applicable	
35	NOCAM	Ophichthus rex	King Snake Eel	N	Brought on Board (Retained/Discarded)	Live - Healthy	Retained as Bait	Not Applicable	
35	NOCAM	Ophichthus rex	King Snake Eel	N	Brought on Board (Retained/Discarded)	Live - Healthy	Retained as Bait	Not Applicable	
35	UCAM	Crab, unidentified	Crab, Unidentified	N	Brought on Board (Retained/Discarded)	Live - Healthy	Discarded - Live and Healthy (Not Vented)	Not Applicable	
35	UCAM	Crab, unidentified	Crab, Unidentified	N	Brought on Board (Retained/Discarded)	Live - Healthy	Discarded - Live and Healthy (Not Vented)	Not Applicable	
35	UCAM	Mustelus spp.	Dogfish, Smooth (Florida)	Y	Brought on Board (Retained/Discarded)	Live - Healthy	Discarded - Live and Healthy (Not Vented)	Male (Claspers)	Medium (Between 1m and 2m)
35	UCAM	Ophichthus rex	King Snake Eel	N	Brought on Board (Retained/Discarded)	Live - Healthy	Retained as Bait	Not Applicable	
35	UCAM	Ophichthus rex	King Snake Eel	N	Brought on Board (Retained/Discarded)	Live - Healthy	Retained as Bait	Not Applicable	
35	UCAM	Ophichthus rex	King Snake Eel	N	Brought on Board (Retained/Discarded)	Live - Healthy	Retained as Bait	Not Applicable	
36	NOCAM	Carcharhinus falciformis	Silky Shark	Y	Cut-off at Rail (No Entanglement)	Live - Healthy	Discarded - Live and Healthy (Not Vented)	Unknown Maturity and/or Sex	Medium (Between 1m and 2m)
36	NOCAM	Carcharhinus plumbeus	Sandbar Shark	Y	Cut-off at Rail (No Entanglement)	Live - Healthy	Discarded - Live and Healthy (Not Vented)	Male (Claspers)	Large (Greater than 2m)
36	NOCAM	Crab, unidentified	Crab, Unidentified	N	Brought on Board (Retained/Discarded)	Live - Healthy	Discarded - Live and Healthy (Not Vented)	Not Applicable	
36	NOCAM	Epinephelus niveatus	Snowy Grouper	N	Brought on Board (Retained/Discarded)	Live - Healthy	Retained	Not Applicable	

36	NOCAM	Epinephelus niveatus	Snowy Grouper	N	Brought on Board (Retained/Discarded)	Live - Healthy	Retained	Not Applicable	
36	NOCAM	Mycteroperca microlepis	Gag Grouper	N	Brought on Board (Retained/Discarded)	Live - Healthy	Retained	Not Applicable	
36	NOCAM	Mycteroperca microlepis	Gag Grouper	N	Brought on Board (Retained/Discarded)	Live - Stomach and/or Eyes Protruding	Retained	Not Applicable	
36	NOCAM	Mycteroperca microlepis	Gag Grouper	N	Brought on Board (Retained/Discarded)	Live - Healthy	Retained	Not Applicable	
36	NOCAM	Mycteroperca microlepis	Gag Grouper	N	Brought on Board (Retained/Discarded)	Live - Healthy	Retained	Not Applicable	
36	NOCAM	Mycteroperca phenax	Scamp	N	Brought on Board (Retained/Discarded)	Live - Healthy	Retained	Not Applicable	
36	NOCAM	Mycteroperca phenax	Scamp	N	Brought on Board (Retained/Discarded)	Live - Stomach and/or Eyes Protruding	Retained	Not Applicable	
36	UCAM	Carcharhinus falciformis	Silky Shark	Y	Cut-off at Rail (No Entanglement)	Live - Healthy	Discarded - Live and Healthy (Not Vented)	Unknown Maturity and/or Sex	Medium (Between 1m and 2m)
36	UCAM	Carcharhinus plumbeus	Sandbar Shark	Y	Cut-off at Rail (No Entanglement)	Live - Healthy	Discarded - Live and Healthy (Not Vented)	Male (Claspers)	Large (Greater than 2m)
36	UCAM	Crab, unidentified	Crab, Unidentified	N	Brought on Board (Retained/Discarded)	Live - Healthy	Discarded - Live and Healthy (Not Vented)	Not Applicable	
36	UCAM	Epinephelus niveatus	Snowy Grouper	N	Brought on Board (Retained/Discarded)	Live - Stomach and/or Eyes Protruding	Retained	Not Applicable	
36	UCAM	Epinephelus niveatus	Snowy Grouper	N	Brought on Board (Retained/Discarded)	Live - Healthy	Retained	Not Applicable	
36	UCAM	Mycteroperca microlepis	Gag Grouper	N	Brought on Board (Retained/Discarded)	Live - Healthy	Retained	Not Applicable	
36	UCAM	Mycteroperca microlepis	Gag Grouper	N	Brought on Board (Retained/Discarded)	Live - Healthy	Retained	Not Applicable	
36	UCAM	Mycteroperca microlepis	Gag Grouper	N	Brought on Board (Retained/Discarded)	Live - Healthy	Retained	Not Applicable	
36	UCAM	Mycteroperca microlepis	Gag Grouper	N	Brought on Board (Retained/Discarded)	Live - Healthy	Retained	Not Applicable	
36	UCAM	Mycteroperca phenax	Scamp	N	Brought on Board (Retained/Discarded)	Live - Healthy	Retained	Not Applicable	
36	UCAM	Mycteroperca phenax	Scamp	N	Brought on Board (Retained/Discarded)	Live - Stomach and/or Eyes Protruding	Retained	Not Applicable	
37	NOCAM	Carcharhinus plumbeus	Sandbar Shark	Y	Cut-off at Rail (No Entanglement)	Live - Healthy	Discarded - Live and Healthy (Not Vented)	Male (Claspers)	Large (Greater than 2m)
37	NOCAM	Epinephelus flavolimbatus	Yellowedge Grouper	N	Brought on Board (Retained/Discarded)	Live - Stomach and/or Eyes Protruding	Retained	Not Applicable	
37	NOCAM	Gadiformes, unidentified	Hake, Unidentified	N	Brought on Board (Retained/Discarded)	Live - Stomach and/or Eyes Protruding	Retained as Bait	Not Applicable	
37	NOCAM	Gadiformes, unidentified	Hake, Unidentified	N	Brought on Board (Retained/Discarded)	Live - Stomach and/or Eyes Protruding	Retained as Bait	Not Applicable	
37	NOCAM	Gadiformes, unidentified	Hake, Unidentified	N	Brought on Board (Retained/Discarded)	Live - Healthy	Retained as Bait	Not Applicable	
37	NOCAM	Gadiformes, unidentified	Hake, Unidentified	N	Brought on Board (Retained/Discarded)	Live - Healthy	Retained as Bait	Not Applicable	
37	NOCAM	Gadiformes, unidentified	Hake, Unidentified	N	Brought on Board (Retained/Discarded)	Live - Stomach and/or Eyes Protruding	Retained as Bait	Not Applicable	
37	NOCAM	Muraenidae, unidentified	Moray Eel, Unidentified	N	Brought on Board (Retained/Discarded)	Live - Healthy	Retained as Bait	Not Applicable	
37	NOCAM	Mustelus spp.	Dogfish, Smooth (Florida)	Y	Brought on Board (Retained/Discarded)	Live - Healthy	Discarded - Live and Healthy (Not Vented)	Female	Medium (Between 1m and 2m)
37	NOCAM	Ophichthus rex	King Snake Eel	N	Brought on Board (Retained/Discarded)	Live - Healthy	Retained as Bait	Not Applicable	
37	NOCAM	Ophichthus rex	King Snake Eel	N	Brought on Board (Retained/Discarded)	Live - Healthy	Retained as Bait	Not Applicable	
37	NOCAM	Ophichthus rex	King Snake Eel	N	Brought on Board (Retained/Discarded)	Live - Healthy	Retained as Bait	Not Applicable	
37	NOCAM	Ophichthus rex	King Snake Eel	N	Brought on Board (Retained/Discarded)	Live - Healthy	Retained as Bait	Not Applicable	
37	NOCAM	Ophichthus rex	King Snake Eel	N	Brought on Board (Retained/Discarded)	Live - Healthy	Retained as Bait	Not Applicable	
37	NOCAM	Ophichthus rex	King Snake Eel	N	Brought on Board (Retained/Discarded)	Live - Healthy	Retained as Bait	Not Applicable	
37	NOCAM	Ophichthus rex	King Snake Eel	N	Brought on Board (Retained/Discarded)	Live - Healthy	Retained as Bait	Not Applicable	
37	NOCAM	Ophichthus rex	King Snake Eel	N	Brought on Board (Retained/Discarded)	Live - Healthy	Retained as Bait	Not Applicable	
37	UCAM	Carcharhinus plumbeus	Sandbar Shark	Y	Cut-off at Rail (No Entanglement)	Live - Healthy	Discarded - Live and Healthy (Not Vented)	Male (Claspers)	Large (Greater than 2m)
37	UCAM	Epinephelus flavolimbatus	Yellowedge Grouper	N	Brought on Board (Retained/Discarded)	Live - Stomach and/or Eyes Protruding	Retained	Not Applicable	
37	UCAM	Gadiformes, unidentified	Hake, Unidentified	N	Brought on Board (Retained/Discarded)	Live - Healthy	Retained as Bait	Not Applicable	
37	UCAM	Gadiformes, unidentified	Hake, Unidentified	N	Brought on Board (Retained/Discarded)	Live - Healthy	Retained as Bait	Not Applicable	
37	UCAM	Gadiformes, unidentified	Hake, Unidentified	N	Brought on Board (Retained/Discarded)	Live - Stomach and/or Eyes Protruding	Retained as Bait	Not Applicable	
37	UCAM	Gadiformes, unidentified	Hake, Unidentified	N	Brought on Board (Retained/Discarded)	Live - Stomach and/or Eyes Protruding	Retained as Bait	Not Applicable	
37	UCAM	Gadiformes, unidentified	Hake, Unidentified	N	Brought on Board (Retained/Discarded)	Live - Stomach and/or Eyes Protruding	Retained as Bait	Not Applicable	
37	UCAM	Muraenidae, unidentified	Moray Eel, Unidentified	N	Brought on Board (Retained/Discarded)	Live - Healthy	Retained as Bait	Not Applicable	
37	UCAM	Mustelus spp.	Dogfish, Smooth (Florida)	Y	Brought on Board (Retained/Discarded)	Live - Healthy	Discarded - Live and Healthy (Not Vented)	Female	Small (Less than 1m)
37	UCAM	Mustelus spp.	Dogfish, Smooth (Florida)	Y	Brought on Board (Retained/Discarded)	Live - Healthy	Discarded - Live and Healthy (Not Vented)	Female	Medium (Between 1m and 2m)
37	UCAM	Ophichthus rex	King Snake Eel	N	Brought on Board (Retained/Discarded)	Live - Healthy	Retained as Bait	Not Applicable	
37	UCAM	Ophichthus rex	King Snake Eel	N	Brought on Board (Retained/Discarded)	Live - Healthy	Retained as Bait	Not Applicable	
37	UCAM	Ophichthus rex	King Snake Eel	N	Brought on Board (Retained/Discarded)	Live - Healthy	Retained as Bait	Not Applicable	
37	UCAM	Ophichthus rex	King Snake Eel	N	Brought on Board (Retained/Discarded)	Live - Healthy	Retained as Bait	Not Applicable	
37	UCAM	Ophichthus rex	King Snake Eel	N	Brought on Board (Retained/Discarded)	Live - Healthy	Retained as Bait	Not Applicable	
37	UCAM	Ophichthus rex	King Snake Eel	N	Brought on Board (Retained/Discarded)	Live - Healthy	Retained as Bait	Not Applicable	
37	UCAM	Ophichthus rex	King Snake Eel	N	Brought on Board (Retained/Discarded)	Live - Healthy	Retained as Bait	Not Applicable	
37	UCAM	Ophichthus rex	King Snake Eel	N	Brought on Board (Retained/Discarded)	Live - Healthy	Retained as Bait	Not Applicable	
38	NOCAM	Carcharhinus acronotus	Blacknose Shark	Y	Brought on Board (Retained/Discarded)	Live - Healthy	Discarded - Live and Healthy (Not Vented)	Female	Medium (Between 1m and 2m)
38	NOCAM	Carcharhinus acronotus	Blacknose Shark	Y	Brought on Board (Retained/Discarded)	Live - Healthy	Discarded - Live and Healthy (Not Vented)	Female	Medium (Between 1m and 2m)
38	NOCAM	Epinephelus morio	Red Grouper	N	Brought on Board (Retained/Discarded)	Live - Stomach and/or Eyes Protruding	Retained	Not Applicable	
38	NOCAM	Epinephelus morio	Red Grouper	N	Brought on Board (Retained/Discarded)	Live - Healthy	Retained	Not Applicable	
38	NOCAM	Epinephelus morio	Red Grouper	N	Brought on Board (Retained/Discarded)	Live - Stomach and/or Eyes Protruding	Retained	Not Applicable	
38	NOCAM	Epinephelus morio	Red Grouper	N	Brought on Board (Retained/Discarded)	Live - Healthy	Retained	Not Applicable	
38	NOCAM	Epinephelus morio	Red Grouper	N	Brought on Board (Retained/Discarded)	Live - Healthy	Retained	Not Applicable	
38	NOCAM	Epinephelus morio	Red Grouper	N	Brought on Board (Retained/Discarded)	Live - Healthy	Retained	Not Applicable	
38	NOCAM	Epinephelus morio	Red Grouper	N	Brought on Board (Retained/Discarded)	Live - Healthy	Retained	Not Applicable	
38	NOCAM	Epinephelus morio	Red Grouper	N	Brought on Board (Retained/Discarded)	Live - Stomach and/or Eyes Protruding	Discarded - Live and Healthy (Vented)	Not Applicable	
38	NOCAM	Epinephelus morio	Red Grouper	N	Brought on Board (Retained/Discarded)	Live - Healthy	Retained	Not Applicable	
38	NOCAM	Epinephelus morio	Red Grouper	N	Brought on Board (Retained/Discarded)	Live - Stomach and/or Eyes Protruding	Discarded - Live and Healthy (Vented)	Not Applicable	
38	NOCAM	Epinephelus morio	Red Grouper	N	Brought on Board (Retained/Discarded)	Live - Stomach and/or Eyes Protruding	Discarded - Live and Healthy (Vented)	Not Applicable	
38	NOCAM	Epinephelus morio	Red Grouper	N	Brought on Board (Retained/Discarded)	Live - Healthy	Retained	Not Applicable	
38	NOCAM	Epinephelus morio	Red Grouper	N	Brought on Board (Retained/Discarded)	Live - Healthy	Retained	Not Applicable	
38	NOCAM	Epinephelus morio	Red Grouper	N	Brought on Board (Retained/Discarded)	Live - Healthy	Retained	Not Applicable	
38	NOCAM	Epinephelus morio	Red Grouper	N	Brought on Board (Retained/Discarded)	Live - Healthy	Discarded - Live and Healthy (Vented)	Not Applicable	
38	NOCAM	Epinephelus morio	Red Grouper	N	Brought on Board (Retained/Discarded)	Live - Healthy	Retained	Not Applicable	
38	NOCAM	Epinephelus morio	Red Grouper	N	Brought on Board (Retained/Discarded)	Live - Healthy	Retained	Not Applicable	
38	NOCAM	Epinephelus morio	Red Grouper	N	Brought on Board (Retained/Discarded)	Live - Healthy	Retained	Not Applicable	
38	NOCAM	Epinephelus morio	Red Grouper	N	Brought on Board (Retained/Discarded)	Live - Stomach and/or Eyes Protruding	Retained	Not Applicable	
38	NOCAM	Epinephelus morio	Red Grouper	N	Brought on Board (Retained/Discarded)	Live - Healthy	Discarded - Live and Healthy (Not Vented)	Not Applicable	
38	NOCAM	Epinephelus morio	Red Grouper	N	Brought on Board (Retained/Discarded)	Live - Healthy	Retained	Not Applicable	
38	NOCAM	Epinephelus morio	Red Grouper	N	Brought on Board (Retained/Discarded)	Live - Healthy	Discarded - Live and Healthy (Vented)	Not Applicable	
38	NOCAM	Epinephelus morio	Red Grouper	N	Brought on Board (Retained/Discarded)	Live - Stomach and/or Eyes Protruding	Retained	Not Applicable	
38	NOCAM	Epinephelus morio	Red Grouper	N	Brought on Board (Retained/Discarded)	Live - Healthy	Retained	Not Applicable	
38	NOCAM	Epinephelus morio	Red Grouper	N	Brought on Board (Retained/Discarded)	Live - Healthy	Retained	Not Applicable	
38	NOCAM	Epinephelus morio	Red Grouper	N	Brought on Board (Retained/Discarded)	Live - Healthy	Retained	Not Applicable	
38	NOCAM	Epinephelus morio	Red Grouper	N	Brought on Board (Retained/Discarded)	Live - Healthy	Retained	Not Applicable	
38	NOCAM	Epinephelus morio	Red Grouper	N	Brought on Board (Retained/Discarded)	Live - Stomach and/or Eyes Protruding	Discarded - Live and Healthy (Vented)	Not Applicable	
38	NOCAM	Epinephelus morio	Red Grouper	N	Brought on Board (Retained/Discarded)	Live - Stomach and/or Eyes Protruding	Retained	Not Applicable	
38	NOCAM	Epinephelus morio	Red Grouper	N	Brought on Board (Retained/Discarded)	Live - Stomach and/or Eyes Protruding	Retained	Not Applicable	
38	NOCAM	Epinephelus morio	Red Grouper	N	Brought on Board (Retained/Discarded)	Live - Stomach and/or Eyes Protruding	Discarded - Live and Healthy (Vented)	Not Applicable	
38	NOCAM	Epinephelus morio	Red Grouper	N	Brought on Board (Retained/Discarded)	Live - Healthy	Discarded - Live and Healthy (Vented)	Not Applicable	
38	NOCAM	Epinephelus morio	Red Grouper	N	Brought on Board (Retained/Discarded)	Live - Stomach and/or Eyes Protruding	Retained	Not Applicable	
38	NOCAM	Epinephelus morio	Red Grouper	N	Brought on Board (Retained/Discarded)	Live - Healthy	Retained	Not Applicable	
38	NOCAM	Epinephelus morio	Red Grouper	N	Brought on Board (Retained/Discarded)	Live - Stomach and/or Eyes Protruding	Retained	Not Applicable	
38	NOCAM	Epinephelus morio	Red Grouper	N	Brought on Board (Retained/Discarded)	Live - Healthy	Discarded - Live and Healthy (Vented)	Not Applicable	
38	NOCAM	Epinephelus morio	Red Grouper	N	Brought on Board (Retained/Discarded)	Live - Healthy	Retained	Not Applicable	

38	NOCAM	Lutjanus analis	Mutton Snapper	N	Brought on Board (Retained/Discarded)	Live - Stomach and/or Eyes Protruding	Retained	Not Applicable	
38	NOCAM	Lutjanus analis	Mutton Snapper	N	Brought on Board (Retained/Discarded)	Live - Healthy	Retained	Not Applicable	
38	NOCAM	Lutjanus analis	Mutton Snapper	N	Brought on Board (Retained/Discarded)	Live - Healthy	Retained	Not Applicable	
38	NOCAM	Lutjanus campechanus	Red Snapper	N	Brought on Board (Retained/Discarded)	Dead on Arrival - Damaged	Discarded - Dead	Not Applicable	
38	NOCAM	Lutjanus campechanus	Red Snapper	N	Brought on Board (Retained/Discarded)	Live - Healthy	Discarded - Live and Healthy (Vented)	Not Applicable	
38	NOCAM	Lutjanus campechanus	Red Snapper	N	Brought on Board (Retained/Discarded)	Live - Healthy	Discarded - Live and Healthy (Vented)	Not Applicable	
38	NOCAM	Lutjanus campechanus	Red Snapper	N	Brought on Board (Retained/Discarded)	Live - Healthy	Discarded - Live and Healthy (Vented)	Not Applicable	
38	NOCAM	Lutjanus campechanus	Red Snapper	N	Brought on Board (Retained/Discarded)	Live - Healthy	Discarded - Live and Healthy (Vented)	Not Applicable	
38	NOCAM	Lutjanus campechanus	Red Snapper	N	Brought on Board (Retained/Discarded)	Live - Healthy	Discarded - Live and Healthy (Vented)	Not Applicable	
38	NOCAM	Lutjanus campechanus	Red Snapper	N	Brought on Board (Retained/Discarded)	Dead on Arrival - Damaged	Discarded - Dead	Not Applicable	
38	NOCAM	Lutjanus campechanus	Red Snapper	N	Brought on Board (Retained/Discarded)	Live - Healthy	Discarded - Live and Healthy (Vented)	Not Applicable	
38	NOCAM	Lutjanus campechanus	Red Snapper	N	Brought on Board (Retained/Discarded)	Dead on Arrival - Undamaged	Discarded - Dead	Not Applicable	
38	NOCAM	Lutjanus campechanus	Red Snapper	N	Brought on Board (Retained/Discarded)	Live - Healthy	Discarded - Live and Healthy (Vented)	Not Applicable	
38	NOCAM	Lutjanus campechanus	Red Snapper	N	Brought on Board (Retained/Discarded)	Live - Healthy	Discarded - Live and Healthy (Vented)	Not Applicable	
38	NOCAM	Lutjanus campechanus	Red Snapper	N	Brought on Board (Retained/Discarded)	Live - Healthy	Discarded - Live and Healthy (Vented)	Not Applicable	
38	NOCAM	Lutjanus campechanus	Red Snapper	N	Brought on Board (Retained/Discarded)	Live - Healthy	Discarded - Live and Healthy (Vented)	Not Applicable	
38	NOCAM	Lutjanus campechanus	Red Snapper	N	Brought on Board (Retained/Discarded)	Live - Healthy	Discarded - Live and Healthy (Vented)	Not Applicable	
38	NOCAM	Lutjanus campechanus	Red Snapper	N	Brought on Board (Retained/Discarded)	Live - Healthy	Discarded - Live and Healthy (Vented)	Not Applicable	
38	NOCAM	Lutjanus campechanus	Red Snapper	N	Brought on Board (Retained/Discarded)	Live - Healthy	Discarded - Live and Healthy (Vented)	Not Applicable	
38	NOCAM	Lutjanus campechanus	Red Snapper	N	Brought on Board (Retained/Discarded)	Live - Healthy	Discarded - Live and Healthy (Vented)	Not Applicable	
38	NOCAM	Lutjanus campechanus	Red Snapper	N	Brought on Board (Retained/Discarded)	Live - Healthy	Discarded - Live and Healthy (Vented)	Not Applicable	
38	NOCAM	Lutjanus campechanus	Red Snapper	N	Brought on Board (Retained/Discarded)	Live - Healthy	Discarded - Live and Healthy (Vented)	Not Applicable	
38	NOCAM	Lutjanus campechanus	Red Snapper	N	Brought on Board (Retained/Discarded)	Live - Healthy	Discarded - Live and Healthy (Vented)	Not Applicable	
38	NOCAM	Lutjanus campechanus	Red Snapper	N	Brought on Board (Retained/Discarded)	Dead on Arrival - Undamaged	Discarded - Dead	Not Applicable	
38	NOCAM	Lutjanus campechanus	Red Snapper	N	Brought on Board (Retained/Discarded)	Live - Healthy	Discarded - Live and Healthy (Vented)	Not Applicable	
38	NOCAM	Lutjanus campechanus	Red Snapper	N	Brought on Board (Retained/Discarded)	Live - Healthy	Discarded - Live and Healthy (Vented)	Not Applicable	
38	NOCAM	Lutjanus campechanus	Red Snapper	N	Brought on Board (Retained/Discarded)	Live - Healthy	Discarded - Live and Healthy (Vented)	Not Applicable	
38	NOCAM	Lutjanus campechanus	Red Snapper	N	Brought on Board (Retained/Discarded)	Live - Healthy	Discarded - Live and Healthy (Vented)	Not Applicable	
38	NOCAM	Lutjanus campechanus	Red Snapper	N	Brought on Board (Retained/Discarded)	Live - Healthy	Discarded - Live and Healthy (Vented)	Not Applicable	
38	NOCAM	Muraenidae, unidentified	Moray Eel, Unidentified	N	Brought on Board (Retained/Discarded)	Live - Healthy	Retained as Bait	Not Applicable	
38	NOCAM	Muraenidae, unidentified	Moray Eel, Unidentified	N	Brought on Board (Retained/Discarded)	Live - Healthy	Retained as Bait	Not Applicable	
38	NOCAM	Rhizoprionodon terraenovae	Atlantic Sharpnose Shark	Y	Brought on Board (Retained/Discarded)	Live - Healthy	Discarded - Live and Healthy (Not Vented)	Female	Small (Less than 1m)
38	NOCAM	Rhizoprionodon terraenovae	Atlantic Sharpnose Shark	Y	Brought on Board (Retained/Discarded)	Live - Healthy	Discarded - Live and Healthy (Not Vented)	Female	Small (Less than 1m)
38	NOCAM	Rhizoprionodon terraenovae	Atlantic Sharpnose Shark	Y	Brought on Board (Retained/Discarded)	Live - Healthy	Discarded - Live and Healthy (Not Vented)	Female	Small (Less than 1m)
38	NOCAM	Rhizoprionodon terraenovae	Atlantic Sharpnose Shark	Y	Brought on Board (Retained/Discarded)	Live - Healthy	Discarded - Live and Healthy (Not Vented)	Female	Small (Less than 1m)
38	NOCAM	Rhizoprionodon terraenovae	Atlantic Sharpnose Shark	Y	Brought on Board (Retained/Discarded)	Live - Healthy	Discarded - Live and Healthy (Not Vented)	Female	Small (Less than 1m)
38	NOCAM	Rhizoprionodon terraenovae	Atlantic Sharpnose Shark	Y	Brought on Board (Retained/Discarded)	Live - Healthy	Discarded - Live and Healthy (Not Vented)	Female	Small (Less than 1m)
38	NOCAM	Rhizoprionodon terraenovae	Atlantic Sharpnose Shark	Y	Brought on Board (Retained/Discarded)	Live - Healthy	Discarded - Live and Healthy (Not Vented)	Female	Small (Less than 1m)
38	NOCAM	Rhizoprionodon terraenovae	Atlantic Sharpnose Shark	Y	Brought on Board (Retained/Discarded)	Live - Healthy	Discarded - Live and Healthy (Not Vented)	Female	Small (Less than 1m)
38	NOCAM	Rhizoprionodon terraenovae	Atlantic Sharpnose Shark	Y	Brought on Board (Retained/Discarded)	Live - Healthy	Discarded - Live and Healthy (Not Vented)	Female	Small (Less than 1m)
38	NOCAM	Rhizoprionodon terraenovae	Atlantic Sharpnose Shark	Y	Brought on Board (Retained/Discarded)	Live - Healthy	Discarded - Live and Healthy (Not Vented)	Female	Small (Less than 1m)
38	NOCAM	Rhizoprionodon terraenovae	Atlantic Sharpnose Shark	Y	Brought on Board (Retained/Discarded)	Live - Healthy	Discarded - Live and Healthy (Not Vented)	Female	Small (Less than 1m)
38	NOCAM	Rhizoprionodon terraenovae	Atlantic Sharpnose Shark	Y	Brought on Board (Retained/Discarded)	Dead on Arrival - Undamaged	Discarded - Dead	Female	Small (Less than 1m)
38	NOCAM	Rhizoprionodon terraenovae	Atlantic Sharpnose Shark	Y	Brought on Board (Retained/Discarded)	Live - Healthy	Discarded - Live and Healthy (Not Vented)	Female	Small (Less than 1m)
38	NOCAM	Rhizoprionodon terraenovae	Atlantic Sharpnose Shark	Y	Brought on Board (Retained/Discarded)	Live - Healthy	Discarded - Live and Healthy (Not Vented)	Female	Small (Less than 1m)
38	NOCAM	Rhizoprionodon terraenovae	Atlantic Sharpnose Shark	Y	Brought on Board (Retained/Discarded)	Live - Healthy	Discarded - Live and Healthy (Not Vented)	Female	Small (Less than 1m)
38	NOCAM	Rhizoprionodon terraenovae	Atlantic Sharpnose Shark	Y	Brought on Board (Retained/Discarded)	Live - Healthy	Discarded - Live and Healthy (Not Vented)	Female	Small (Less than 1m)
38	NOCAM	Rhizoprionodon terraenovae	Atlantic Sharpnose Shark	Y	Brought on Board (Retained/Discarded)	Live - Healthy	Discarded - Live and Healthy (Not Vented)	Female	Small (Less than 1m)
38	NOCAM	Sphyraena barracuda	Barracuda (Great)	N	Brought on Board (Retained/Discarded)	Live - Healthy	Discarded - Live and Healthy (Not Vented)	Not Applicable	
38	NOCAM	Unidentified Bottom Debris	Unidentified Bottom Debris	N	Brought on Board (Retained/Discarded)	Unknown Condition	Discarded - Unknown	Not Applicable	
38	UCAM	Carcharhinus acronotus	Blacknose Shark	Y	Brought on Board (Retained/Discarded)	Live - Healthy	Discarded - Live and Healthy (Not Vented)	Female	Medium (Between 1m and 2m)
38	UCAM	Carcharhinus acronotus	Blacknose Shark	Y	Brought on Board (Retained/Discarded)	Live - Healthy	Discarded - Live and Healthy (Not Vented)	Female	Medium (Between 1m and 2m)
38	UCAM	Carcharhinus limbatus	Blacktip Shark	Y	Cut-off at Rail (No Entanglement)	Live - Healthy	Discarded - Live and Healthy (Not Vented)	Female	Medium (Between 1m and 2m)
38	UCAM	Epinephelus morio	Red Grouper	N	Brought on Board (Retained/Discarded)	Live - Stomach and/or Eyes Protruding	Retained	Not Applicable	
38	UCAM	Epinephelus morio	Red Grouper	N	Brought on Board (Retained/Discarded)	Live - Healthy	Retained	Not Applicable	
38	UCAM	Epinephelus morio	Red Grouper	N	Brought on Board (Retained/Discarded)	Live - Healthy	Retained	Not Applicable	
38	UCAM	Epinephelus morio	Red Grouper	N	Brought on Board (Retained/Discarded)	Live - Healthy	Discarded - Live and Healthy (Vented)	Not Applicable	
38	UCAM	Epinephelus morio	Red Grouper	N	Brought on Board (Retained/Discarded)	Live - Healthy	Discarded - Live and Healthy (Vented)	Not Applicable	
38	UCAM	Epinephelus morio	Red Grouper	N	Brought on Board (Retained/Discarded)	Live - Healthy	Retained	Not Applicable	
38	UCAM	Epinephelus morio	Red Grouper	N	Brought on Board (Retained/Discarded)	Live - Stomach and/or Eyes Protruding	Retained	Not Applicable	
38	UCAM	Epinephelus morio	Red Grouper	N	Brought on Board (Retained/Discarded)	Live - Stomach and/or Eyes Protruding	Retained	Not Applicable	
38	UCAM	Epinephelus morio	Red Grouper	N	Brought on Board (Retained/Discarded)	Live - Healthy	Discarded - Live and Healthy (Not Vented)	Not Applicable	
38	UCAM	Epinephelus morio	Red Grouper	N	Brought on Board (Retained/Discarded)	Live - Stomach and/or Eyes Protruding	Retained	Not Applicable	
38	UCAM	Epinephelus morio	Red Grouper	N	Brought on Board (Retained/Discarded)	Live - Healthy	Discarded - Live and Healthy (Not Vented)	Not Applicable	
38	UCAM	Epinephelus morio	Red Grouper	N	Brought on Board (Retained/Discarded)	Live - Healthy	Retained	Not Applicable	
38	UCAM	Epinephelus morio	Red Grouper	N	Brought on Board (Retained/Discarded)	Live - Healthy	Discarded - Live and Healthy (Not Vented)	Not Applicable	
38	UCAM	Epinephelus morio	Red Grouper	N	Brought on Board (Retained/Discarded)	Live - Healthy	Retained	Not Applicable	
38	UCAM	Epinephelus morio	Red Grouper	N	Brought on Board (Retained/Discarded)	Live - Healthy	Retained	Not Applicable	
38	UCAM	Epinephelus morio	Red Grouper	N	Brought on Board (Retained/Discarded)	Live - Healthy	Retained	Not Applicable	
38	UCAM	Epinephelus morio	Red Grouper	N	Brought on Board (Retained/Discarded)	Live - Stomach and/or Eyes Protruding	Discarded - Live and Healthy (Not Vented)	Not Applicable	
38	UCAM	Epinephelus morio	Red Grouper	N	Brought on Board (Retained/Discarded)	Live - Healthy	Retained	Not Applicable	
38	UCAM	Epinephelus morio	Red Grouper	N	Brought on Board (Retained/Discarded)	Live - Healthy	Discarded - Live and Healthy (Vented)	Not Applicable	
38	UCAM	Epinephelus morio	Red Grouper	N	Brought on Board (Retained/Discarded)	Live - Healthy	Retained	Not Applicable	
38	UCAM	Epinephelus morio	Red Grouper	N	Brought on Board (Retained/Discarded)	Live - Stomach and/or Eyes Protruding	Discarded - Live and Healthy (Vented)	Not Applicable	
38	UCAM	Epinephelus morio	Red Grouper	N	Brought on Board (Retained/Discarded)	Live - Stomach and/or Eyes Protruding	Retained	Not Applicable	
38	UCAM	Epinephelus morio	Red Grouper	N	Brought on Board (Retained/Discarded)	Live - Healthy	Retained	Not Applicable	
38	UCAM	Epinephelus morio	Red Grouper	N	Brought on Board (Retained/Discarded)	Dead on Arrival - Undamaged	Retained	Not Applicable	
38	UCAM	Epinephelus morio	Red Grouper	N	Brought on Board (Retained/Discarded)	Live - Healthy	Retained	Not Applicable	
38	UCAM	Epinephelus morio	Red Grouper	N	Brought on Board (Retained/Discarded)	Live - Stomach and/or Eyes Protruding	Retained	Not Applicable	
38	UCAM	Epinephelus morio	Red Grouper	N	Brought on Board (Retained/Discarded)	Live - Healthy	Retained	Not Applicable	
38	UCAM	Epinephelus morio	Red Grouper	N	Brought on Board (Retained/Discarded)	Live - Healthy	Retained	Not Applicable	
38	UCAM	Epinephelus morio	Red Grouper	N	Brought on Board (Retained/Discarded)	Live - Stomach and/or Eyes Protruding	Discarded - Live and Healthy (Not Vented)	Not Applicable	
38	UCAM	Epinephelus morio	Red Grouper	N	Brought on Board (Retained/Discarded)	Live - Healthy	Retained	Not Applicable	
38	UCAM	Epinephelus morio	Red Grouper	N	Brought on Board (Retained/Discarded)	Live - Stomach and/or Eyes Protruding	Discarded - Live and Healthy (Vented)	Not Applicable	
38	UCAM	Epinephelus morio	Red Grouper	N	Brought on Board (Retained/Discarded)	Live - Stomach and/or Eyes Protruding	Retained	Not Applicable	
38	UCAM	Epinephelus morio	Red Grouper	N	Brought on Board (Retained/Discarded)	Live - Healthy	Retained	Not Applicable	
38	UCAM	Epinephelus morio	Red Grouper	N	Brought on Board (Retained/Discarded)	Live - Stomach and/or Eyes Protruding	Retained	Not Applicable	
38	UCAM	Epinephelus morio	Red Grouper	N	Brought on Board (Retained/Discarded)	Live - Healthy	Retained	Not Applicable	
38	UCAM	Epinephelus morio	Red Grouper	N	Brought on Board (Retained/Discarded)	Live - Stomach and/or Eyes Protruding	Retained	Not Applicable	
38	UCAM	Lutjanus analis	Mutton Snapper	N	Brought on Board (Retained/Discarded)	Live - Healthy	Retained	Not Applicable	
38	UCAM	Lutjanus analis	Mutton Snapper	N	Brought on Board (Retained/Discarded)	Live - Healthy	Retained	Not Applicable	
38	UCAM	Lutjanus analis	Mutton Snapper	N	Brought on Board (Retained/Discarded)	Live - Stomach and/or Eyes Protruding	Retained	Not Applicable	

38	UCAM	Lutjanus campechanus	Red Snapper	N	Brought on Board (Retained/Discarded)	Live - Healthy	Discarded - Live and Healthy (Vented)	Not Applicable	
38	UCAM	Lutjanus campechanus	Red Snapper	N	Brought on Board (Retained/Discarded)	Live - Healthy	Discarded - Live and Healthy (Vented)	Not Applicable	
38	UCAM	Lutjanus campechanus	Red Snapper	N	Brought on Board (Retained/Discarded)	Live - Healthy	Discarded - Live and Healthy (Vented)	Not Applicable	
38	UCAM	Lutjanus campechanus	Red Snapper	N	Brought on Board (Retained/Discarded)	Dead on Arrival - Undamaged	Discarded - Dead	Not Applicable	
38	UCAM	Lutjanus campechanus	Red Snapper	N	Brought on Board (Retained/Discarded)	Live - Healthy	Discarded - Live and Healthy (Vented)	Not Applicable	
38	UCAM	Lutjanus campechanus	Red Snapper	N	Brought on Board (Retained/Discarded)	Live - Healthy	Discarded - Live and Healthy (Vented)	Not Applicable	
38	UCAM	Lutjanus campechanus	Red Snapper	N	Brought on Board (Retained/Discarded)	Live - Healthy	Discarded - Live and Healthy (Vented)	Not Applicable	
38	UCAM	Lutjanus campechanus	Red Snapper	N	Brought on Board (Retained/Discarded)	Live - Healthy	Discarded - Live and Healthy (Vented)	Not Applicable	
38	UCAM	Lutjanus campechanus	Red Snapper	N	Brought on Board (Retained/Discarded)	Live - Healthy	Discarded - Live and Healthy (Vented)	Not Applicable	
38	UCAM	Lutjanus campechanus	Red Snapper	N	Brought on Board (Retained/Discarded)	Live - Healthy	Discarded - Live and Healthy (Vented)	Not Applicable	
38	UCAM	Lutjanus campechanus	Red Snapper	N	Brought on Board (Retained/Discarded)	Live - Healthy	Discarded - Live and Healthy (Vented)	Not Applicable	
38	UCAM	Lutjanus campechanus	Red Snapper	N	Brought on Board (Retained/Discarded)	Live - Healthy	Discarded - Live and Healthy (Vented)	Not Applicable	
38	UCAM	Lutjanus campechanus	Red Snapper	N	Brought on Board (Retained/Discarded)	Dead on Arrival - Damaged	Discarded - Dead	Not Applicable	
38	UCAM	Lutjanus campechanus	Red Snapper	N	Brought on Board (Retained/Discarded)	Live - Healthy	Discarded - Live and Healthy (Vented)	Not Applicable	
38	UCAM	Lutjanus campechanus	Red Snapper	N	Brought on Board (Retained/Discarded)	Live - Healthy	Discarded - Live and Healthy (Vented)	Not Applicable	
38	UCAM	Lutjanus campechanus	Red Snapper	N	Brought on Board (Retained/Discarded)	Live - Healthy	Discarded - Live and Healthy (Vented)	Not Applicable	
38	UCAM	Lutjanus campechanus	Red Snapper	N	Brought on Board (Retained/Discarded)	Live - Healthy	Discarded - Live and Healthy (Vented)	Not Applicable	
38	UCAM	Lutjanus campechanus	Red Snapper	N	Brought on Board (Retained/Discarded)	Live - Healthy	Discarded - Live and Healthy (Vented)	Not Applicable	
38	UCAM	Lutjanus campechanus	Red Snapper	N	Brought on Board (Retained/Discarded)	Live - Stomach and/or Eyes Protruding	Discarded - Live and Healthy (Vented)	Not Applicable	
38	UCAM	Lutjanus campechanus	Red Snapper	N	Brought on Board (Retained/Discarded)	Live - Healthy	Discarded - Live and Healthy (Vented)	Not Applicable	
38	UCAM	Lutjanus campechanus	Red Snapper	N	Brought on Board (Retained/Discarded)	Live - Healthy	Discarded - Live and Healthy (Vented)	Not Applicable	
38	UCAM	Lutjanus campechanus	Red Snapper	N	Brought on Board (Retained/Discarded)	Live - Healthy	Discarded - Live and Healthy (Vented)	Not Applicable	
38	UCAM	Lutjanus campechanus	Red Snapper	N	Brought on Board (Retained/Discarded)	Live - Healthy	Discarded - Live and Healthy (Vented)	Not Applicable	
38	UCAM	Lutjanus campechanus	Red Snapper	N	Brought on Board (Retained/Discarded)	Live - Healthy	Discarded - Live and Healthy (Vented)	Not Applicable	
38	UCAM	Rhizoprionodon terraenovae	Atlantic Sharpnose Shark	Y	Brought on Board (Retained/Discarded)	Live - Healthy	Discarded - Live and Healthy (Not Vented)	Female	Small (Less than 1m)
38	UCAM	Rhizoprionodon terraenovae	Atlantic Sharpnose Shark	Y	Brought on Board (Retained/Discarded)	Live - Healthy	Discarded - Live and Healthy (Not Vented)	Female	Small (Less than 1m)
38	UCAM	Rhizoprionodon terraenovae	Atlantic Sharpnose Shark	Y	Brought on Board (Retained/Discarded)	Live - Healthy	Discarded - Live and Healthy (Not Vented)	Female	Small (Less than 1m)
38	UCAM	Rhizoprionodon terraenovae	Atlantic Sharpnose Shark	Y	Brought on Board (Retained/Discarded)	Live - Healthy	Discarded - Live and Healthy (Not Vented)	Female	Small (Less than 1m)
38	UCAM	Rhizoprionodon terraenovae	Atlantic Sharpnose Shark	Y	Brought on Board (Retained/Discarded)	Live - Healthy	Discarded - Live and Healthy (Not Vented)	Female	Small (Less than 1m)
38	UCAM	Rhizoprionodon terraenovae	Atlantic Sharpnose Shark	Y	Brought on Board (Retained/Discarded)	Live - Healthy	Discarded - Live and Healthy (Not Vented)	Female	Small (Less than 1m)
38	UCAM	Rhizoprionodon terraenovae	Atlantic Sharpnose Shark	Y	Brought on Board (Retained/Discarded)	Live - Healthy	Discarded - Live and Healthy (Not Vented)	Female	Small (Less than 1m)
38	UCAM	Rhizoprionodon terraenovae	Atlantic Sharpnose Shark	Y	Brought on Board (Retained/Discarded)	Live - Healthy	Discarded - Live and Healthy (Not Vented)	Female	Small (Less than 1m)
38	UCAM	Rhizoprionodon terraenovae	Atlantic Sharpnose Shark	Y	Brought on Board (Retained/Discarded)	Live - Healthy	Discarded - Live and Healthy (Not Vented)	Female	Small (Less than 1m)
38	UCAM	Rhizoprionodon terraenovae	Atlantic Sharpnose Shark	Y	Brought on Board (Retained/Discarded)	Live - Healthy	Discarded - Live and Healthy (Not Vented)	Female	Small (Less than 1m)
38	UCAM	Rhizoprionodon terraenovae	Atlantic Sharpnose Shark	Y	Brought on Board (Retained/Discarded)	Live - Healthy	Discarded - Live and Healthy (Not Vented)	Female	Small (Less than 1m)
38	UCAM	Rhizoprionodon terraenovae	Atlantic Sharpnose Shark	Y	Brought on Board (Retained/Discarded)	Live - Healthy	Discarded - Live and Healthy (Not Vented)	Female	Small (Less than 1m)
38	UCAM	Rhizoprionodon terraenovae	Atlantic Sharpnose Shark	Y	Brought on Board (Retained/Discarded)	Live - Healthy	Discarded - Live and Healthy (Not Vented)	Female	Small (Less than 1m)
38	UCAM	Rhizoprionodon terraenovae	Atlantic Sharpnose Shark	Y	Brought on Board (Retained/Discarded)	Live - Healthy	Discarded - Live and Healthy (Not Vented)	Female	Small (Less than 1m)
38	UCAM	Rhizoprionodon terraenovae	Atlantic Sharpnose Shark	Y	Brought on Board (Retained/Discarded)	Live - Healthy	Discarded - Live and Healthy (Not Vented)	Female	Small (Less than 1m)
38	UCAM	Rhizoprionodon terraenovae	Atlantic Sharpnose Shark	Y	Brought on Board (Retained/Discarded)	Live - Healthy	Discarded - Live and Healthy (Not Vented)	Female	Small (Less than 1m)
38	UCAM	Rhizoprionodon terraenovae	Atlantic Sharpnose Shark	Y	Brought on Board (Retained/Discarded)	Live - Healthy	Discarded - Live and Healthy (Not Vented)	Female	Small (Less than 1m)
38	UCAM	Sphyraena barracuda	Barracuda (Great)	N	Brought on Board (Retained/Discarded)	Live - Healthy	Discarded - Live and Healthy (Not Vented)	Not Applicable	
38	UCAM	Unidentified Bottom Debris	Unidentified Bottom Debris	N	Brought on Board (Retained/Discarded)	Unknown Condition	Discarded - Unknown	Not Applicable	
39	NOCAM	Calamus bajonada	Jolthead Porgy	N	Brought on Board (Retained/Discarded)	Live - Healthy	Retained	Not Applicable	
39	NOCAM	Epinephelus morio	Red Grouper	N	Brought on Board (Retained/Discarded)	Live - Stomach and/or Eyes Protruding	Retained	Not Applicable	
39	NOCAM	Epinephelus morio	Red Grouper	N	Brought on Board (Retained/Discarded)	Live - Healthy	Retained	Not Applicable	
39	NOCAM	Epinephelus morio	Red Grouper	N	Brought on Board (Retained/Discarded)	Live - Healthy	Retained	Not Applicable	
39	NOCAM	Epinephelus morio	Red Grouper	N	Brought on Board (Retained/Discarded)	Live - Healthy	Retained	Not Applicable	
39	NOCAM	Epinephelus morio	Red Grouper	N	Brought on Board (Retained/Discarded)	Live - Stomach and/or Eyes Protruding	Retained	Not Applicable	
39	NOCAM	Epinephelus morio	Red Grouper	N	Brought on Board (Retained/Discarded)	Live - Healthy	Retained	Not Applicable	
39	NOCAM	Epinephelus morio	Red Grouper	N	Brought on Board (Retained/Discarded)	Live - Stomach and/or Eyes Protruding	Retained	Not Applicable	
39	NOCAM	Epinephelus morio	Red Grouper	N	Brought on Board (Retained/Discarded)	Live - Healthy	Discarded - Live and Healthy (Vented)	Not Applicable	
39	NOCAM	Epinephelus morio	Red Grouper	N	Brought on Board (Retained/Discarded)	Live - Stomach and/or Eyes Protruding	Retained	Not Applicable	
39	NOCAM	Epinephelus morio	Red Grouper	N	Brought on Board (Retained/Discarded)	Live - Stomach and/or Eyes Protruding	Retained	Not Applicable	
39	NOCAM	Epinephelus morio	Red Grouper	N	Brought on Board (Retained/Discarded)	Live - Stomach and/or Eyes Protruding	Retained	Not Applicable	
39	NOCAM	Epinephelus morio	Red Grouper	N	Brought on Board (Retained/Discarded)	Live - Healthy	Retained	Not Applicable	
39	NOCAM	Epinephelus morio	Red Grouper	N	Brought on Board (Retained/Discarded)	Live - Stomach and/or Eyes Protruding	Retained	Not Applicable	
39	NOCAM	Epinephelus morio	Red Grouper	N	Brought on Board (Retained/Discarded)	Live - Healthy	Retained	Not Applicable	
39	NOCAM	Epinephelus morio	Red Grouper	N	Brought on Board (Retained/Discarded)	Live - Healthy	Retained	Not Applicable	
39	NOCAM	Epinephelus morio	Red Grouper	N	Brought on Board (Retained/Discarded)	Live - Healthy	Retained	Not Applicable	
39	NOCAM	Epinephelus morio	Red Grouper	N	Brought on Board (Retained/Discarded)	Live - Healthy	Retained	Not Applicable	
39	NOCAM	Epinephelus morio	Red Grouper	N	Brought on Board (Retained/Discarded)	Live - Healthy	Discarded - Live and Healthy (Vented)	Not Applicable	
39	NOCAM	Epinephelus morio	Red Grouper	N	Brought on Board (Retained/Discarded)	Live - Healthy	Discarded - Live and Healthy (Vented)	Not Applicable	
39	NOCAM	Epinephelus morio	Red Grouper	N	Brought on Board (Retained/Discarded)	Live - Stomach and/or Eyes Protruding	Retained	Not Applicable	
39	NOCAM	Epinephelus morio	Red Grouper	N	Brought on Board (Retained/Discarded)	Live - Stomach and/or Eyes Protruding	Retained	Not Applicable	
39	NOCAM	Epinephelus morio	Red Grouper	N	Brought on Board (Retained/Discarded)	Live - Healthy	Discarded - Live and Healthy (Vented)	Not Applicable	
39	NOCAM	Epinephelus morio	Red Grouper	N	Brought on Board (Retained/Discarded)	Live - Stomach and/or Eyes Protruding	Retained	Not Applicable	
39	NOCAM	Epinephelus morio	Red Grouper	N	Brought on Board (Retained/Discarded)	Live - Healthy	Retained	Not Applicable	
39	NOCAM	Epinephelus morio	Red Grouper	N	Brought on Board (Retained/Discarded)	Live - Stomach and/or Eyes Protruding	Retained	Not Applicable	
39	NOCAM	Epinephelus morio	Red Grouper	N	Brought on Board (Retained/Discarded)	Live - Healthy	Retained	Not Applicable	
39	NOCAM	Epinephelus morio	Red Grouper	N	Brought on Board (Retained/Discarded)	Live - Healthy	Retained	Not Applicable	
39	NOCAM	Epinephelus morio	Red Grouper	N	Brought on Board (Retained/Discarded)	Live - Stomach and/or Eyes Protruding	Discarded - Live and Healthy (Vented)	Not Applicable	
39	NOCAM	Epinephelus morio	Red Grouper	N	Brought on Board (Retained/Discarded)	Live - Stomach and/or Eyes Protruding	Retained	Not Applicable	
39	NOCAM	Epinephelus morio	Red Grouper	N	Brought on Board (Retained/Discarded)	Live - Stomach and/or Eyes Protruding	Retained	Not Applicable	
39	NOCAM	Epinephelus morio	Red Grouper	N	Brought on Board (Retained/Discarded)	Live - Healthy	Retained	Not Applicable	
39	NOCAM	Epinephelus morio	Red Grouper	N	Brought on Board (Retained/Discarded)	Live - Stomach and/or Eyes Protruding	Retained	Not Applicable	
39	NOCAM	Epinephelus morio	Red Grouper	N	Brought on Board (Retained/Discarded)	Live - Healthy	Retained	Not Applicable	
39	NOCAM	Epinephelus morio	Red Grouper	N	Brought on Board (Retained/Discarded)	Live - Healthy	Retained	Not Applicable	
39	NOCAM	Epinephelus morio	Red Grouper	N	Brought on Board (Retained/Discarded)	Live - Healthy	Discarded - Live and Healthy (Not Vented)	Not Applicable	
39	NOCAM	Epinephelus morio	Red Grouper	N	Brought on Board (Retained/Discarded)	Live - Healthy	Retained	Not Applicable	
39	NOCAM	Epinephelus morio	Red Grouper	N	Brought on Board (Retained/Discarded)	Live - Healthy	Retained	Not Applicable	
39	NOCAM	Epinephelus morio	Red Grouper	N	Brought on Board (Retained/Discarded)	Live - Healthy	Retained	Not Applicable	
39	NOCAM	Epinephelus morio	Red Grouper	N	Brought on Board (Retained/Discarded)	Live - Healthy	Retained	Not Applicable	
39	NOCAM	Epinephelus morio	Red Grouper	N	Brought on Board (Retained/Discarded)	Live - Stomach and/or Eyes Protruding	Retained	Not Applicable	
39	NOCAM	Epinephelus morio	Red Grouper	N	Brought on Board (Retained/Discarded)	Live - Stomach and/or Eyes Protruding	Retained	Not Applicable	
39	NOCAM	Ginglymostoma cirratum	Nurse Shark	Y	Cut-off at Rail (No Entanglement)	Live - Healthy	Discarded - Live and Healthy (Not Vented)	Known Adult - Undetermined Sex	Medium (Between 1m and 2m)
39	NOCAM	Lutjanus analis	Mutton Snapper	N	Brought on Board (Retained/Discarded)	Dead on Arrival - Undamaged	Retained	Not Applicable	
39	NOCAM	Sphyraena barracuda	Barracuda (Great)	N	Brought on Board (Retained/Discarded)	Live - Healthy	Discarded - Live and Healthy (Not Vented)	Not Applicable	
39	NOCAM	Teleost, unidentified	Finfish, Unidentified	N	Cut-off at Rail (No Entanglement)	Dead on Arrival - Damaged	Discarded - Dead	Not Applicable	
39	UCAM	Calamus bajonada	Jolthead Porgy	N	Brought on Board (Retained/Discarded)	Live - Healthy	Retained	Not Applicable	
39	UCAM	Epinephelus morio	Red Grouper	N	Brought on Board (Retained/Discarded)	Live - Stomach and/or Eyes Protruding	Retained	Not Applicable	
39	UCAM	Epinephelus morio	Red Grouper	N	Brought on Board (Retained/Discarded)	Live - Stomach and/or Eyes Protruding	Retained	Not Applicable	

39	UCAM	Epinephelus morio	Red Grouper	N	Brought on Board (Retained/Discarded)	Live - Healthy	Retained	Not Applicable	
39	UCAM	Epinephelus morio	Red Grouper	N	Brought on Board (Retained/Discarded)	Live - Stomach and/or Eyes Protruding	Retained	Not Applicable	
39	UCAM	Epinephelus morio	Red Grouper	N	Brought on Board (Retained/Discarded)	Live - Healthy	Discarded - Live and Healthy (Vented)	Not Applicable	
39	UCAM	Epinephelus morio	Red Grouper	N	Brought on Board (Retained/Discarded)	Live - Healthy	Retained	Not Applicable	
39	UCAM	Epinephelus morio	Red Grouper	N	Brought on Board (Retained/Discarded)	Live - Healthy	Retained	Not Applicable	
39	UCAM	Epinephelus morio	Red Grouper	N	Brought on Board (Retained/Discarded)	Live - Stomach and/or Eyes Protruding	Retained	Not Applicable	
39	UCAM	Epinephelus morio	Red Grouper	N	Brought on Board (Retained/Discarded)	Live - Stomach and/or Eyes Protruding	Retained	Not Applicable	
39	UCAM	Epinephelus morio	Red Grouper	N	Brought on Board (Retained/Discarded)	Live - Stomach and/or Eyes Protruding	Retained	Not Applicable	
39	UCAM	Epinephelus morio	Red Grouper	N	Brought on Board (Retained/Discarded)	Live - Healthy	Retained	Not Applicable	
39	UCAM	Epinephelus morio	Red Grouper	N	Brought on Board (Retained/Discarded)	Live - Healthy	Retained	Not Applicable	
39	UCAM	Epinephelus morio	Red Grouper	N	Brought on Board (Retained/Discarded)	Live - Stomach and/or Eyes Protruding	Retained	Not Applicable	
39	UCAM	Epinephelus morio	Red Grouper	N	Brought on Board (Retained/Discarded)	Live - Healthy	Discarded - Live and Healthy (Vented)	Not Applicable	
39	UCAM	Epinephelus morio	Red Grouper	N	Brought on Board (Retained/Discarded)	Live - Healthy	Retained	Not Applicable	
39	UCAM	Epinephelus morio	Red Grouper	N	Brought on Board (Retained/Discarded)	Live - Healthy	Retained	Not Applicable	
39	UCAM	Epinephelus morio	Red Grouper	N	Brought on Board (Retained/Discarded)	Live - Stomach and/or Eyes Protruding	Retained	Not Applicable	
39	UCAM	Epinephelus morio	Red Grouper	N	Brought on Board (Retained/Discarded)	Live - Healthy	Retained	Not Applicable	
39	UCAM	Epinephelus morio	Red Grouper	N	Brought on Board (Retained/Discarded)	Live - Stomach and/or Eyes Protruding	Retained	Not Applicable	
39	UCAM	Epinephelus morio	Red Grouper	N	Brought on Board (Retained/Discarded)	Live - Stomach and/or Eyes Protruding	Retained	Not Applicable	
39	UCAM	Epinephelus morio	Red Grouper	N	Brought on Board (Retained/Discarded)	Live - Stomach and/or Eyes Protruding	Retained	Not Applicable	
39	UCAM	Epinephelus morio	Red Grouper	N	Brought on Board (Retained/Discarded)	Live - Healthy	Retained	Not Applicable	
39	UCAM	Epinephelus morio	Red Grouper	N	Brought on Board (Retained/Discarded)	Live - Stomach and/or Eyes Protruding	Retained	Not Applicable	
39	UCAM	Epinephelus morio	Red Grouper	N	Brought on Board (Retained/Discarded)	Live - Healthy	Discarded - Live and Healthy (Vented)	Not Applicable	
39	UCAM	Epinephelus morio	Red Grouper	N	Brought on Board (Retained/Discarded)	Live - Healthy	Retained	Not Applicable	
39	UCAM	Epinephelus morio	Red Grouper	N	Brought on Board (Retained/Discarded)	Live - Healthy	Retained	Not Applicable	
39	UCAM	Epinephelus morio	Red Grouper	N	Brought on Board (Retained/Discarded)	Live - Healthy	Discarded - Live and Healthy (Vented)	Not Applicable	
39	UCAM	Epinephelus morio	Red Grouper	N	Brought on Board (Retained/Discarded)	Live - Healthy	Retained	Not Applicable	
39	UCAM	Epinephelus morio	Red Grouper	N	Brought on Board (Retained/Discarded)	Live - Healthy	Retained	Not Applicable	
39	UCAM	Epinephelus morio	Red Grouper	N	Brought on Board (Retained/Discarded)	Live - Healthy	Retained	Not Applicable	
39	UCAM	Epinephelus morio	Red Grouper	N	Brought on Board (Retained/Discarded)	Live - Healthy	Retained	Not Applicable	
39	UCAM	Epinephelus morio	Red Grouper	N	Brought on Board (Retained/Discarded)	Live - Healthy	Retained	Not Applicable	
39	UCAM	Epinephelus morio	Red Grouper	N	Brought on Board (Retained/Discarded)	Live - Healthy	Discarded - Live and Healthy (Vented)	Not Applicable	
39	UCAM	Epinephelus morio	Red Grouper	N	Brought on Board (Retained/Discarded)	Live - Stomach and/or Eyes Protruding	Retained	Not Applicable	
39	UCAM	Epinephelus morio	Red Grouper	N	Brought on Board (Retained/Discarded)	Live - Healthy	Retained	Not Applicable	
39	UCAM	Epinephelus morio	Red Grouper	N	Brought on Board (Retained/Discarded)	Live - Stomach and/or Eyes Protruding	Retained	Not Applicable	
39	UCAM	Epinephelus morio	Red Grouper	N	Brought on Board (Retained/Discarded)	Live - Stomach and/or Eyes Protruding	Retained	Not Applicable	
39	UCAM	Epinephelus morio	Red Grouper	N	Brought on Board (Retained/Discarded)	Live - Healthy	Discarded - Live and Healthy (Vented)	Not Applicable	
39	UCAM	Epinephelus morio	Red Grouper	N	Brought on Board (Retained/Discarded)	Live - Healthy	Retained	Not Applicable	
39	UCAM	Epinephelus morio	Red Grouper	N	Brought on Board (Retained/Discarded)	Live - Healthy	Retained	Not Applicable	
39	UCAM	Epinephelus morio	Red Grouper	N	Brought on Board (Retained/Discarded)	Live - Healthy	Retained	Not Applicable	
39	UCAM	Ginglymostoma cirratum	Nurse Shark	Y	Cut-off at Rail (No Entanglement)	Live - Healthy	Discarded - Live and Healthy (Not Vented)	Male (Claspers)	Medium (Between 1m and 2m)
39	UCAM	Lutjanus analis	Mutton Snapper	N	Brought on Board (Retained/Discarded)	Dead on Arrival - Undamaged	Retained	Not Applicable	
39	UCAM	Sphyraena barracuda	Barracuda (Great)	N	Brought on Board (Retained/Discarded)	Live - Healthy	Discarded - Live and Healthy (Not Vented)	Not Applicable	
39	UCAM	Teleost, unidentified	Finfish, Unidentified	N	Cut-off at Rail (No Entanglement)	Dead on Arrival - Damaged	Discarded - Dead	Not Applicable	
40	NOCAM	Calamus bajonada	Jolthead Porgy	N	Brought on Board (Retained/Discarded)	Live - Healthy	Retained	Not Applicable	
40	NOCAM	Calamus bajonada	Jolthead Porgy	N	Brought on Board (Retained/Discarded)	Live - Healthy	Retained	Not Applicable	
40	NOCAM	Calamus bajonada	Jolthead Porgy	N	Brought on Board (Retained/Discarded)	Live - Healthy	Retained	Not Applicable	
40	NOCAM	Epinephelus morio	Red Grouper	N	Brought on Board (Retained/Discarded)	Live - Healthy	Retained	Not Applicable	
40	NOCAM	Epinephelus morio	Red Grouper	N	Brought on Board (Retained/Discarded)	Live - Healthy	Retained	Not Applicable	
40	NOCAM	Epinephelus morio	Red Grouper	N	Brought on Board (Retained/Discarded)	Live - Healthy	Retained	Not Applicable	
40	NOCAM	Epinephelus morio	Red Grouper	N	Brought on Board (Retained/Discarded)	Live - Healthy	Discarded - Live and Healthy (Vented)	Not Applicable	
40	NOCAM	Epinephelus morio	Red Grouper	N	Brought on Board (Retained/Discarded)	Live - Healthy	Discarded - Live and Healthy (Vented)	Not Applicable	
40	NOCAM	Epinephelus morio	Red Grouper	N	Brought on Board (Retained/Discarded)	Live - Healthy	Discarded - Live and Healthy (Not Vented)	Not Applicable	
40	NOCAM	Epinephelus morio	Red Grouper	N	Brought on Board (Retained/Discarded)	Live - Stomach and/or Eyes Protruding	Retained	Not Applicable	
40	NOCAM	Epinephelus morio	Red Grouper	N	Brought on Board (Retained/Discarded)	Live - Healthy	Discarded - Live and Healthy (Vented)	Not Applicable	
40	NOCAM	Epinephelus morio	Red Grouper	N	Brought on Board (Retained/Discarded)	Live - Stomach and/or Eyes Protruding	Retained	Not Applicable	
40	NOCAM	Epinephelus morio	Red Grouper	N	Brought on Board (Retained/Discarded)	Live - Stomach and/or Eyes Protruding	Retained	Not Applicable	
40	NOCAM	Epinephelus morio	Red Grouper	N	Brought on Board (Retained/Discarded)	Live - Healthy	Discarded - Live and Healthy (Not Vented)	Not Applicable	
40	NOCAM	Epinephelus morio	Red Grouper	N	Brought on Board (Retained/Discarded)	Live - Stomach and/or Eyes Protruding	Retained	Not Applicable	
40	NOCAM	Epinephelus morio	Red Grouper	N	Brought on Board (Retained/Discarded)	Live - Healthy	Retained	Not Applicable	
40	NOCAM	Epinephelus morio	Red Grouper	N	Brought on Board (Retained/Discarded)	Live - Healthy	Discarded - Live and Healthy (Vented)	Not Applicable	
40	NOCAM	Epinephelus morio	Red Grouper	N	Brought on Board (Retained/Discarded)	Live - Stomach and/or Eyes Protruding	Retained	Not Applicable	
40	NOCAM	Epinephelus morio	Red Grouper	N	Brought on Board (Retained/Discarded)	Live - Healthy	Retained	Not Applicable	
40	NOCAM	Epinephelus morio	Red Grouper	N	Brought on Board (Retained/Discarded)	Live - Stomach and/or Eyes Protruding	Discarded - Live and Healthy (Vented)	Not Applicable	
40	NOCAM	Epinephelus morio	Red Grouper	N	Brought on Board (Retained/Discarded)	Live - Stomach and/or Eyes Protruding	Retained	Not Applicable	
40	NOCAM	Epinephelus morio	Red Grouper	N	Brought on Board (Retained/Discarded)	Live - Stomach and/or Eyes Protruding	Retained	Not Applicable	
40	NOCAM	Epinephelus morio	Red Grouper	N	Brought on Board (Retained/Discarded)	Live - Healthy	Discarded - Live and Healthy (Vented)	Not Applicable	
40	NOCAM	Epinephelus morio	Red Grouper	N	Brought on Board (Retained/Discarded)	Live - Healthy	Retained	Not Applicable	
40	NOCAM	Epinephelus morio	Red Grouper	N	Brought on Board (Retained/Discarded)	Live - Healthy	Discarded - Live and Healthy (Vented)	Not Applicable	
40	NOCAM	Epinephelus morio	Red Grouper	N	Brought on Board (Retained/Discarded)	Live - Healthy	Discarded - Live and Healthy (Vented)	Not Applicable	
40	NOCAM	Epinephelus morio	Red Grouper	N	Brought on Board (Retained/Discarded)	Dead on Arrival - Damaged	Discarded - Dead	Not Applicable	
40	NOCAM	Epinephelus morio	Red Grouper	N	Brought on Board (Retained/Discarded)	Live - Healthy	Discarded - Live and Healthy (Not Vented)	Not Applicable	
40	NOCAM	Epinephelus morio	Red Grouper	N	Brought on Board (Retained/Discarded)	Live - Stomach and/or Eyes Protruding	Retained	Not Applicable	
40	NOCAM	Epinephelus morio	Red Grouper	N	Brought on Board (Retained/Discarded)	Live - Healthy	Discarded - Live and Healthy (Not Vented)	Not Applicable	
40	NOCAM	Epinephelus morio	Red Grouper	N	Brought on Board (Retained/Discarded)	Live - Stomach and/or Eyes Protruding	Retained	Not Applicable	
40	NOCAM	Epinephelus morio	Red Grouper	N	Brought on Board (Retained/Discarded)	Live - Healthy	Discarded - Live and Healthy (Vented)	Not Applicable	
40	NOCAM	Epinephelus morio	Red Grouper	N	Brought on Board (Retained/Discarded)	Live - Stomach and/or Eyes Protruding	Retained	Not Applicable	
40	NOCAM	Epinephelus morio	Red Grouper	N	Brought on Board (Retained/Discarded)	Live - Stomach and/or Eyes Protruding	Retained	Not Applicable	
40	NOCAM	Epinephelus morio	Red Grouper	N	Brought on Board (Retained/Discarded)	Live - Healthy	Retained	Not Applicable	
40	NOCAM	Epinephelus morio	Red Grouper	N	Brought on Board (Retained/Discarded)	Live - Stomach and/or Eyes Protruding	Discarded - Live and Healthy (Vented)	Not Applicable	
40	NOCAM	Epinephelus morio	Red Grouper	N	Brought on Board (Retained/Discarded)	Live - Healthy	Discarded - Live and Healthy (Vented)	Not Applicable	
40	NOCAM	Epinephelus morio	Red Grouper	N	Brought on Board (Retained/Discarded)	Live - Stomach and/or Eyes Protruding	Discarded - Live and Healthy (Vented)	Not Applicable	
40	NOCAM	Lutjanus analis	Mutton Snapper	N	Brought on Board (Retained/Discarded)	Live - Healthy	Retained	Not Applicable	
40	NOCAM	Lutjanus analis	Mutton Snapper	N	Brought on Board (Retained/Discarded)	Live - Healthy	Retained	Not Applicable	
40	NOCAM	Lutjanus analis	Mutton Snapper	N	Brought on Board (Retained/Discarded)	Live - Healthy	Retained	Not Applicable	
40	NOCAM	Lutjanus campechanus	Red Snapper	N	Brought on Board (Retained/Discarded)	Live - Healthy	Retained	Not Applicable	
40	NOCAM	Lutjanus campechanus	Red Snapper	N	Brought on Board (Retained/Discarded)	Live - Healthy	Retained	Not Applicable	
40	NOCAM	Lutjanus campechanus	Red Snapper	N	Brought on Board (Retained/Discarded)	Live - Healthy	Retained	Not Applicable	
40	NOCAM	Lutjanus campechanus	Red Snapper	N	Brought on Board (Retained/Discarded)	Live - Healthy	Retained	Not Applicable	
40	NOCAM	Lutjanus campechanus	Red Snapper	N	Brought on Board (Retained/Discarded)	Live - Healthy	Retained	Not Applicable	
40	NOCAM	Lutjanus campechanus	Red Snapper	N	Brought on Board (Retained/Discarded)	Live - Stomach and/or Eyes Protruding	Retained	Not Applicable	
40	NOCAM	Lutjanus campechanus	Red Snapper	N	Brought on Board (Retained/Discarded)	Live - Healthy	Retained	Not Applicable	
40	NOCAM	Lutjanus campechanus	Red Snapper	N	Brought on Board (Retained/Discarded)	Live - Healthy	Retained	Not Applicable	

40	NOCAM	Lutjanus campechanus	Red Snapper	N	Brought on Board (Retained/Discarded)	Live - Stomach and/or Eyes Protruding	Retained	Not Applicable	
40	UCAM	Calamus bajonada	Jolthead Porgy	N	Brought on Board (Retained/Discarded)	Live - Healthy	Retained	Not Applicable	
40	UCAM	Calamus bajonada	Jolthead Porgy	N	Brought on Board (Retained/Discarded)	Live - Healthy	Retained	Not Applicable	
40	UCAM	Calamus bajonada	Jolthead Porgy	N	Brought on Board (Retained/Discarded)	Live - Healthy	Retained	Not Applicable	
40	UCAM	Epinephelus morio	Red Grouper	N	Brought on Board (Retained/Discarded)	Live - Stomach and/or Eyes Protruding	Discarded - Live and Healthy (Vented)	Not Applicable	
40	UCAM	Epinephelus morio	Red Grouper	N	Brought on Board (Retained/Discarded)	Live - Stomach and/or Eyes Protruding	Retained	Not Applicable	
40	UCAM	Epinephelus morio	Red Grouper	N	Brought on Board (Retained/Discarded)	Live - Stomach and/or Eyes Protruding	Retained	Not Applicable	
40	UCAM	Epinephelus morio	Red Grouper	N	Brought on Board (Retained/Discarded)	Live - Healthy	Discarded - Live and Healthy (Not Vented)	Not Applicable	
40	UCAM	Epinephelus morio	Red Grouper	N	Brought on Board (Retained/Discarded)	Live - Healthy	Discarded - Live and Healthy (Vented)	Not Applicable	
40	UCAM	Epinephelus morio	Red Grouper	N	Brought on Board (Retained/Discarded)	Live - Stomach and/or Eyes Protruding	Discarded - Live and Healthy (Not Vented)	Not Applicable	
40	UCAM	Epinephelus morio	Red Grouper	N	Brought on Board (Retained/Discarded)	Live - Stomach and/or Eyes Protruding	Retained	Not Applicable	
40	UCAM	Epinephelus morio	Red Grouper	N	Brought on Board (Retained/Discarded)	Live - Healthy	Discarded - Live and Healthy (Vented)	Not Applicable	
40	UCAM	Epinephelus morio	Red Grouper	N	Brought on Board (Retained/Discarded)	Live - Stomach and/or Eyes Protruding	Retained	Not Applicable	
40	UCAM	Epinephelus morio	Red Grouper	N	Brought on Board (Retained/Discarded)	Live - Healthy	Retained	Not Applicable	
40	UCAM	Epinephelus morio	Red Grouper	N	Brought on Board (Retained/Discarded)	Live - Healthy	Retained	Not Applicable	
40	UCAM	Epinephelus morio	Red Grouper	N	Brought on Board (Retained/Discarded)	Live - Healthy	Retained	Not Applicable	
40	UCAM	Epinephelus morio	Red Grouper	N	Brought on Board (Retained/Discarded)	Live - Stomach and/or Eyes Protruding	Retained	Not Applicable	
40	UCAM	Epinephelus morio	Red Grouper	N	Brought on Board (Retained/Discarded)	Live - Healthy	Discarded - Live and Healthy (Vented)	Not Applicable	
40	UCAM	Epinephelus morio	Red Grouper	N	Brought on Board (Retained/Discarded)	Live - Stomach and/or Eyes Protruding	Retained	Not Applicable	
40	UCAM	Epinephelus morio	Red Grouper	N	Brought on Board (Retained/Discarded)	Live - Healthy	Discarded - Live and Healthy (Vented)	Not Applicable	
40	UCAM	Epinephelus morio	Red Grouper	N	Brought on Board (Retained/Discarded)	Live - Stomach and/or Eyes Protruding	Discarded - Live and Healthy (Not Vented)	Not Applicable	
40	UCAM	Epinephelus morio	Red Grouper	N	Brought on Board (Retained/Discarded)	Live - Stomach and/or Eyes Protruding	Retained	Not Applicable	
40	UCAM	Epinephelus morio	Red Grouper	N	Brought on Board (Retained/Discarded)	Live - Healthy	Retained	Not Applicable	
40	UCAM	Epinephelus morio	Red Grouper	N	Brought on Board (Retained/Discarded)	Live - Healthy	Retained	Not Applicable	
40	UCAM	Epinephelus morio	Red Grouper	N	Brought on Board (Retained/Discarded)	Live - Healthy	Discarded - Live and Healthy (Vented)	Not Applicable	
40	UCAM	Epinephelus morio	Red Grouper	N	Brought on Board (Retained/Discarded)	Live - Healthy	Discarded - Live and Healthy (Vented)	Not Applicable	
40	UCAM	Epinephelus morio	Red Grouper	N	Brought on Board (Retained/Discarded)	Live - Stomach and/or Eyes Protruding	Retained	Not Applicable	
40	UCAM	Epinephelus morio	Red Grouper	N	Brought on Board (Retained/Discarded)	Live - Healthy	Discarded - Live and Healthy (Vented)	Not Applicable	
40	UCAM	Epinephelus morio	Red Grouper	N	Brought on Board (Retained/Discarded)	Live - Healthy	Retained	Not Applicable	
40	UCAM	Epinephelus morio	Red Grouper	N	Brought on Board (Retained/Discarded)	Live - Healthy	Retained	Not Applicable	
40	UCAM	Epinephelus morio	Red Grouper	N	Brought on Board (Retained/Discarded)	Live - Healthy	Discarded - Live and Healthy (Vented)	Not Applicable	
40	UCAM	Epinephelus morio	Red Grouper	N	Brought on Board (Retained/Discarded)	Live - Healthy	Discarded - Live and Healthy (Not Vented)	Not Applicable	
40	UCAM	Epinephelus morio	Red Grouper	N	Brought on Board (Retained/Discarded)	Live - Healthy	Retained	Not Applicable	
40	UCAM	Epinephelus morio	Red Grouper	N	Brought on Board (Retained/Discarded)	Live - Healthy	Discarded - Live and Healthy (Vented)	Not Applicable	
40	UCAM	Epinephelus morio	Red Grouper	N	Brought on Board (Retained/Discarded)	Live - Stomach and/or Eyes Protruding	Retained	Not Applicable	
40	UCAM	Epinephelus morio	Red Grouper	N	Brought on Board (Retained/Discarded)	Live - Healthy	Retained	Not Applicable	
40	UCAM	Lutjanus analis	Mutton Snapper	N	Brought on Board (Retained/Discarded)	Live - Healthy	Retained	Not Applicable	
40	UCAM	Lutjanus analis	Mutton Snapper	N	Brought on Board (Retained/Discarded)	Live - Stomach and/or Eyes Protruding	Retained	Not Applicable	
40	UCAM	Lutjanus analis	Mutton Snapper	N	Brought on Board (Retained/Discarded)	Live - Healthy	Retained	Not Applicable	
40	UCAM	Lutjanus campechanus	Red Snapper	N	Brought on Board (Retained/Discarded)	Live - Healthy	Retained	Not Applicable	
40	UCAM	Lutjanus campechanus	Red Snapper	N	Brought on Board (Retained/Discarded)	Live - Healthy	Retained	Not Applicable	
40	UCAM	Lutjanus campechanus	Red Snapper	N	Brought on Board (Retained/Discarded)	Live - Healthy	Retained	Not Applicable	
40	UCAM	Lutjanus campechanus	Red Snapper	N	Brought on Board (Retained/Discarded)	Live - Healthy	Retained	Not Applicable	
40	UCAM	Lutjanus campechanus	Red Snapper	N	Brought on Board (Retained/Discarded)	Live - Healthy	Retained	Not Applicable	
40	UCAM	Lutjanus campechanus	Red Snapper	N	Brought on Board (Retained/Discarded)	Live - Healthy	Retained	Not Applicable	
40	UCAM	Lutjanus campechanus	Red Snapper	N	Brought on Board (Retained/Discarded)	Live - Stomach and/or Eyes Protruding	Retained	Not Applicable	
40	UCAM	Lutjanus campechanus	Red Snapper	N	Brought on Board (Retained/Discarded)	Live - Healthy	Retained	Not Applicable	
40	UCAM	Lutjanus campechanus	Red Snapper	N	Brought on Board (Retained/Discarded)	Live - Healthy	Retained	Not Applicable	
40	UCAM	Lutjanus campechanus	Red Snapper	N	Brought on Board (Retained/Discarded)	Live - Healthy	Retained	Not Applicable	
40	UCAM	Rachycentron canadum	Cobia	N	Brought on Board (Retained/Discarded)	Live - Healthy	Discarded - Live and Healthy (Not Vented)	Not Applicable	
41	NOCAM	Epinephelus morio	Red Grouper	N	Brought on Board (Retained/Discarded)	Live - Healthy	Retained	Not Applicable	
41	NOCAM	Epinephelus morio	Red Grouper	N	Brought on Board (Retained/Discarded)	Live - Stomach and/or Eyes Protruding	Retained	Not Applicable	
41	NOCAM	Epinephelus morio	Red Grouper	N	Brought on Board (Retained/Discarded)	Live - Stomach and/or Eyes Protruding	Retained	Not Applicable	
41	NOCAM	Epinephelus morio	Red Grouper	N	Brought on Board (Retained/Discarded)	Live - Healthy	Retained	Not Applicable	
41	NOCAM	Epinephelus morio	Red Grouper	N	Brought on Board (Retained/Discarded)	Live - Healthy	Retained	Not Applicable	
41	NOCAM	Epinephelus morio	Red Grouper	N	Brought on Board (Retained/Discarded)	Live - Healthy	Discarded - Live and Healthy (Vented)	Not Applicable	
41	NOCAM	Epinephelus morio	Red Grouper	N	Brought on Board (Retained/Discarded)	Live - Stomach and/or Eyes Protruding	Retained	Not Applicable	
41	NOCAM	Epinephelus morio	Red Grouper	N	Brought on Board (Retained/Discarded)	Live - Healthy	Retained	Not Applicable	
41	NOCAM	Epinephelus morio	Red Grouper	N	Brought on Board (Retained/Discarded)	Live - Healthy	Retained	Not Applicable	
41	NOCAM	Epinephelus morio	Red Grouper	N	Brought on Board (Retained/Discarded)	Live - Stomach and/or Eyes Protruding	Retained	Not Applicable	
41	NOCAM	Epinephelus morio	Red Grouper	N	Brought on Board (Retained/Discarded)	Live - Healthy	Retained	Not Applicable	
41	NOCAM	Epinephelus morio	Red Grouper	N	Brought on Board (Retained/Discarded)	Live - Healthy	Retained	Not Applicable	
41	NOCAM	Epinephelus morio	Red Grouper	N	Brought on Board (Retained/Discarded)	Live - Stomach and/or Eyes Protruding	Retained	Not Applicable	
41	NOCAM	Epinephelus morio	Red Grouper	N	Brought on Board (Retained/Discarded)	Live - Healthy	Discarded - Live and Healthy (Not Vented)	Not Applicable	
41	NOCAM	Epinephelus morio	Red Grouper	N	Brought on Board (Retained/Discarded)	Live - Healthy	Discarded - Live and Healthy (Vented)	Not Applicable	
41	NOCAM	Epinephelus morio	Red Grouper	N	Brought on Board (Retained/Discarded)	Live - Healthy	Retained	Not Applicable	
41	NOCAM	Epinephelus morio	Red Grouper	N	Brought on Board (Retained/Discarded)	Live - Stomach and/or Eyes Protruding	Retained	Not Applicable	
41	NOCAM	Epinephelus morio	Red Grouper	N	Brought on Board (Retained/Discarded)	Live - Healthy	Retained	Not Applicable	
41	NOCAM	Epinephelus morio	Red Grouper	N	Brought on Board (Retained/Discarded)	Live - Stomach and/or Eyes Protruding	Retained	Not Applicable	
41	NOCAM	Epinephelus morio	Red Grouper	N	Brought on Board (Retained/Discarded)	Live - Healthy	Retained	Not Applicable	
41	NOCAM	Epinephelus morio	Red Grouper	N	Brought on Board (Retained/Discarded)	Live - Healthy	Retained	Not Applicable	
41	NOCAM	Epinephelus morio	Red Grouper	N	Brought on Board (Retained/Discarded)	Live - Healthy	Retained	Not Applicable	
41	NOCAM	Epinephelus morio	Red Grouper	N	Brought on Board (Retained/Discarded)	Live - Healthy	Retained	Not Applicable	
41	NOCAM	Epinephelus morio	Red Grouper	N	Brought on Board (Retained/Discarded)	Live - Stomach and/or Eyes Protruding	Retained	Not Applicable	
41	NOCAM	Epinephelus morio	Red Grouper	N	Brought on Board (Retained/Discarded)	Live - Healthy	Retained	Not Applicable	
41	NOCAM	Epinephelus morio	Red Grouper	N	Brought on Board (Retained/Discarded)	Live - Healthy	Retained	Not Applicable	
41	NOCAM	Epinephelus morio	Red Grouper	N	Brought on Board (Retained/Discarded)	Live - Stomach and/or Eyes Protruding	Retained	Not Applicable	
41	NOCAM	Epinephelus morio	Red Grouper	N	Brought on Board (Retained/Discarded)	Live - Stomach and/or Eyes Protruding	Retained	Not Applicable	
41	NOCAM	Epinephelus morio	Red Grouper	N	Brought on Board (Retained/Discarded)	Live - Healthy	Retained	Not Applicable	
41	NOCAM	Epinephelus morio	Red Grouper	N	Brought on Board (Retained/Discarded)	Live - Healthy	Retained	Not Applicable	
41	NOCAM	Epinephelus morio	Red Grouper	N	Brought on Board (Retained/Discarded)	Live - Healthy	Retained	Not Applicable	
41	NOCAM	Epinephelus morio	Red Grouper	N	Brought on Board (Retained/Discarded)	Live - Healthy	Discarded - Live and Healthy (Vented)	Not Applicable	
41	NOCAM	Epinephelus morio	Red Grouper	N	Brought on Board (Retained/Discarded)	Live - Stomach and/or Eyes Protruding	Retained	Not Applicable	
41	NOCAM	Epinephelus morio	Red Grouper	N	Brought on Board (Retained/Discarded)	Live - Healthy	Discarded - Live and Healthy (Vented)	Not Applicable	
41	NOCAM	Epinephelus morio	Red Grouper	N	Brought on Board (Retained/Discarded)	Live - Stomach and/or Eyes Protruding	Discarded - Live and Healthy (Vented)	Not Applicable	
41	NOCAM	Epinephelus morio	Red Grouper	N	Brought on Board (Retained/Discarded)	Live - Healthy	Retained	Not Applicable	
41	NOCAM	Epinephelus morio	Red Grouper	N	Brought on Board (Retained/Discarded)	Live - Healthy	Retained	Not Applicable	
41	NOCAM	Epinephelus morio	Red Grouper	N	Brought on Board (Retained/Discarded)	Live - Stomach and/or Eyes Protruding	Retained	Not Applicable	
41	NOCAM	Epinephelus morio	Red Grouper	N	Brought on Board (Retained/Discarded)	Live - Healthy	Retained	Not Applicable	
41	NOCAM	Epinephelus morio	Red Grouper	N	Brought on Board (Retained/Discarded)	Live - Healthy	Retained	Not Applicable	
41	NOCAM	Epinephelus morio	Red Grouper	N	Brought on Board (Retained/Discarded)	Live - Stomach and/or Eyes Protruding	Retained	Not Applicable	
41	NOCAM	Epinephelus morio	Red Grouper	N	Brought on Board (Retained/Discarded)	Live - Stomach and/or Eyes Protruding	Retained	Not Applicable	

41	NOCAM	Epinephelus morio	Red Grouper	N	Brought on Board (Retained/Discarded)	Live - Healthy	Retained	Not Applicable	
41	NOCAM	Epinephelus morio	Red Grouper	N	Brought on Board (Retained/Discarded)	Live - Healthy	Discarded - Live and Healthy (Vented)	Not Applicable	
41	NOCAM	Epinephelus morio	Red Grouper	N	Brought on Board (Retained/Discarded)	Live - Healthy	Retained	Not Applicable	
41	NOCAM	Epinephelus morio	Red Grouper	N	Brought on Board (Retained/Discarded)	Dead on Arrival - Damaged	Discarded - Dead	Not Applicable	
41	NOCAM	Epinephelus morio	Red Grouper	N	Brought on Board (Retained/Discarded)	Live - Stomach and/or Eyes Protruding	Retained	Not Applicable	
41	NOCAM	Galeocerdo cuvier	Tiger Shark	Y	Cut-off at Rail (No Entanglement)	Live - Healthy	Discarded - Live and Healthy (Not Vented)	Known Juvenile	Medium (Between 1m and 2m)
41	NOCAM	Galeocerdo cuvier	Tiger Shark	Y	Cut-off at Rail (No Entanglement)	Live - Healthy	Discarded - Live and Healthy (Not Vented)	Known Juvenile	Medium (Between 1m and 2m)
41	NOCAM	Lutjanus campechanus	Red Snapper	N	Brought on Board (Retained/Discarded)	Live - Healthy	Discarded - Live and Healthy (Not Vented)	Not Applicable	
41	NOCAM	Mycteroperca bonaci	Black Grouper	N	Brought on Board (Retained/Discarded)	Live - Healthy	Discarded - Live and Healthy (Vented)	Not Applicable	
41	NOCAM	Mycteroperca phenax	Scamp	N	Brought on Board (Retained/Discarded)	Live - Healthy	Retained	Not Applicable	
41	NOCAM	Rachycentron canadum	Cobia	N	Brought on Board (Retained/Discarded)	Live - Healthy	Discarded - Live and Healthy (Not Vented)	Not Applicable	
41	NOCAM	Sphyraena barracuda	Barracuda (Great)	N	Brought on Board (Retained/Discarded)	Live - Healthy	Discarded - Live and Healthy (Not Vented)	Not Applicable	
41	UCAM	Carcharhinus plumbeus	Sandbar Shark	Y	Cut-off at Rail (No Entanglement)	Live - Healthy	Discarded - Live and Healthy (Not Vented)	Female	Large (Greater than 2m)
41	UCAM	Carcharhinus plumbeus	Sandbar Shark	Y	Not Handled (Dropped Off)	Live - Healthy	Discarded - Live and Healthy (Not Vented)	Female	Large (Greater than 2m)
41	UCAM	Epinephelus morio	Red Grouper	N	Brought on Board (Retained/Discarded)	Live - Healthy	Retained	Not Applicable	
41	UCAM	Epinephelus morio	Red Grouper	N	Brought on Board (Retained/Discarded)	Live - Healthy	Discarded - Live and Healthy (Vented)	Not Applicable	
41	UCAM	Epinephelus morio	Red Grouper	N	Brought on Board (Retained/Discarded)	Live - Healthy	Discarded - Live and Healthy (Vented)	Not Applicable	
41	UCAM	Epinephelus morio	Red Grouper	N	Brought on Board (Retained/Discarded)	Live - Stomach and/or Eyes Protruding	Retained	Not Applicable	
41	UCAM	Epinephelus morio	Red Grouper	N	Brought on Board (Retained/Discarded)	Live - Stomach and/or Eyes Protruding	Retained	Not Applicable	
41	UCAM	Epinephelus morio	Red Grouper	N	Brought on Board (Retained/Discarded)	Live - Healthy	Retained	Not Applicable	
41	UCAM	Epinephelus morio	Red Grouper	N	Brought on Board (Retained/Discarded)	Live - Healthy	Retained	Not Applicable	
41	UCAM	Epinephelus morio	Red Grouper	N	Brought on Board (Retained/Discarded)	Live - Stomach and/or Eyes Protruding	Retained	Not Applicable	
41	UCAM	Epinephelus morio	Red Grouper	N	Brought on Board (Retained/Discarded)	Live - Healthy	Retained	Not Applicable	
41	UCAM	Epinephelus morio	Red Grouper	N	Brought on Board (Retained/Discarded)	Live - Healthy	Retained	Not Applicable	
41	UCAM	Epinephelus morio	Red Grouper	N	Brought on Board (Retained/Discarded)	Live - Healthy	Retained	Not Applicable	
41	UCAM	Epinephelus morio	Red Grouper	N	Brought on Board (Retained/Discarded)	Live - Healthy	Retained	Not Applicable	
41	UCAM	Epinephelus morio	Red Grouper	N	Brought on Board (Retained/Discarded)	Live - Healthy	Retained	Not Applicable	
41	UCAM	Epinephelus morio	Red Grouper	N	Brought on Board (Retained/Discarded)	Live - Healthy	Retained	Not Applicable	
41	UCAM	Epinephelus morio	Red Grouper	N	Brought on Board (Retained/Discarded)	Live - Healthy	Retained	Not Applicable	
41	UCAM	Epinephelus morio	Red Grouper	N	Brought on Board (Retained/Discarded)	Live - Stomach and/or Eyes Protruding	Retained	Not Applicable	
41	UCAM	Epinephelus morio	Red Grouper	N	Brought on Board (Retained/Discarded)	Live - Healthy	Retained	Not Applicable	
41	UCAM	Epinephelus morio	Red Grouper	N	Brought on Board (Retained/Discarded)	Live - Healthy	Retained	Not Applicable	
41	UCAM	Epinephelus morio	Red Grouper	N	Brought on Board (Retained/Discarded)	Live - Healthy	Retained	Not Applicable	
41	UCAM	Epinephelus morio	Red Grouper	N	Brought on Board (Retained/Discarded)	Live - Healthy	Retained	Not Applicable	
41	UCAM	Epinephelus morio	Red Grouper	N	Brought on Board (Retained/Discarded)	Live - Stomach and/or Eyes Protruding	Retained	Not Applicable	
41	UCAM	Epinephelus morio	Red Grouper	N	Brought on Board (Retained/Discarded)	Live - Stomach and/or Eyes Protruding	Retained	Not Applicable	
41	UCAM	Epinephelus morio	Red Grouper	N	Brought on Board (Retained/Discarded)	Live - Healthy	Retained	Not Applicable	
41	UCAM	Epinephelus morio	Red Grouper	N	Brought on Board (Retained/Discarded)	Live - Stomach and/or Eyes Protruding	Retained	Not Applicable	
41	UCAM	Epinephelus morio	Red Grouper	N	Brought on Board (Retained/Discarded)	Live - Healthy	Discarded - Live and Healthy (Vented)	Not Applicable	
41	UCAM	Epinephelus morio	Red Grouper	N	Brought on Board (Retained/Discarded)	Live - Healthy	Retained	Not Applicable	
41	UCAM	Epinephelus morio	Red Grouper	N	Brought on Board (Retained/Discarded)	Live - Stomach and/or Eyes Protruding	Retained	Not Applicable	
41	UCAM	Epinephelus morio	Red Grouper	N	Brought on Board (Retained/Discarded)	Live - Healthy	Discarded - Live and Healthy (Vented)	Not Applicable	
41	UCAM	Epinephelus morio	Red Grouper	N	Brought on Board (Retained/Discarded)	Live - Healthy	Retained	Not Applicable	
41	UCAM	Epinephelus morio	Red Grouper	N	Brought on Board (Retained/Discarded)	Live - Stomach and/or Eyes Protruding	Retained	Not Applicable	
41	UCAM	Epinephelus morio	Red Grouper	N	Brought on Board (Retained/Discarded)	Live - Stomach and/or Eyes Protruding	Retained	Not Applicable	
41	UCAM	Epinephelus morio	Red Grouper	N	Brought on Board (Retained/Discarded)	Live - Healthy	Retained	Not Applicable	
41	UCAM	Epinephelus morio	Red Grouper	N	Brought on Board (Retained/Discarded)	Live - Healthy	Retained	Not Applicable	
41	UCAM	Epinephelus morio	Red Grouper	N	Brought on Board (Retained/Discarded)	Live - Healthy	Discarded - Live and Healthy (Vented)	Not Applicable	
41	UCAM	Epinephelus morio	Red Grouper	N	Brought on Board (Retained/Discarded)	Live - Healthy	Retained	Not Applicable	
41	UCAM	Epinephelus morio	Red Grouper	N	Cut-off at Rail (No Entanglement)	Dead on Arrival - Damaged	Discarded - Dead	Not Applicable	
41	UCAM	Epinephelus morio	Red Grouper	N	Brought on Board (Retained/Discarded)	Live - Healthy	Retained	Not Applicable	
41	UCAM	Epinephelus morio	Red Grouper	N	Brought on Board (Retained/Discarded)	Live - Healthy	Retained	Not Applicable	
41	UCAM	Epinephelus morio	Red Grouper	N	Brought on Board (Retained/Discarded)	Live - Stomach and/or Eyes Protruding	Retained	Not Applicable	
41	UCAM	Epinephelus morio	Red Grouper	N	Brought on Board (Retained/Discarded)	Live - Stomach and/or Eyes Protruding	Retained	Not Applicable	
41	UCAM	Epinephelus morio	Red Grouper	N	Brought on Board (Retained/Discarded)	Dead on Arrival - Damaged	Discarded - Dead	Not Applicable	
41	UCAM	Epinephelus morio	Red Grouper	N	Brought on Board (Retained/Discarded)	Live - Healthy	Discarded - Live and Healthy (Not Vented)	Not Applicable	
41	UCAM	Epinephelus morio	Red Grouper	N	Brought on Board (Retained/Discarded)	Live - Healthy	Discarded - Live and Healthy (Vented)	Not Applicable	
41	UCAM	Epinephelus morio	Red Grouper	N	Brought on Board (Retained/Discarded)	Live - Healthy	Retained	Not Applicable	
41	UCAM	Epinephelus morio	Red Grouper	N	Brought on Board (Retained/Discarded)	Live - Stomach and/or Eyes Protruding	Retained	Not Applicable	
41	UCAM	Epinephelus morio	Red Grouper	N	Brought on Board (Retained/Discarded)	Live - Stomach and/or Eyes Protruding	Retained	Not Applicable	
41	UCAM	Epinephelus morio	Red Grouper	N	Brought on Board (Retained/Discarded)	Live - Stomach and/or Eyes Protruding	Retained	Not Applicable	
41	UCAM	Epinephelus morio	Red Grouper	N	Brought on Board (Retained/Discarded)	Live - Healthy	Retained	Not Applicable	
41	UCAM	Galeocerdo cuvier	Tiger Shark	Y	Cut-off at Rail (No Entanglement)	Live - Healthy	Discarded - Live and Healthy (Not Vented)	Known Juvenile	Medium (Between 1m and 2m)
41	UCAM	Galeocerdo cuvier	Tiger Shark	Y	Cut-off at Rail (No Entanglement)	Live - Healthy	Discarded - Live and Healthy (Not Vented)	Known Juvenile	Medium (Between 1m and 2m)
41	UCAM	Lutjanus campechanus	Red Snapper	N	Brought on Board (Retained/Discarded)	Live - Stomach and/or Eyes Protruding	Discarded - Live and Healthy (Not Vented)	Not Applicable	
41	UCAM	Mycteroperca bonaci	Black Grouper	N	Brought on Board (Retained/Discarded)	Live - Healthy	Discarded - Live and Healthy (Not Vented)	Not Applicable	
41	UCAM	Mycteroperca phenax	Scamp	N	Brought on Board (Retained/Discarded)	Live - Healthy	Retained	Not Applicable	
41	UCAM	Rachycentron canadum	Cobia	N	Brought on Board (Retained/Discarded)	Live - Healthy	Discarded - Live and Healthy (Not Vented)	Not Applicable	
41	UCAM	Sphyraena barracuda	Barracuda (Great)	N	Brought on Board (Retained/Discarded)	Live - Healthy	Discarded - Live and Healthy (Not Vented)	Not Applicable	
42	NOCAM	Epinephelus morio	Red Grouper	N	Brought on Board (Retained/Discarded)	Live - Stomach and/or Eyes Protruding	Retained	Not Applicable	
42	NOCAM	Epinephelus morio	Red Grouper	N	Brought on Board (Retained/Discarded)	Live - Healthy	Retained	Not Applicable	
42	NOCAM	Epinephelus morio	Red Grouper	N	Brought on Board (Retained/Discarded)	Live - Healthy	Retained	Not Applicable	
42	NOCAM	Epinephelus morio	Red Grouper	N	Brought on Board (Retained/Discarded)	Live - Stomach and/or Eyes Protruding	Discarded - Live and Healthy (Vented)	Not Applicable	
42	NOCAM	Epinephelus morio	Red Grouper	N	Brought on Board (Retained/Discarded)	Live - Stomach and/or Eyes Protruding	Retained	Not Applicable	
42	NOCAM	Epinephelus morio	Red Grouper	N	Brought on Board (Retained/Discarded)	Live - Healthy	Discarded - Live and Healthy (Vented)	Not Applicable	
42	NOCAM	Epinephelus morio	Red Grouper	N	Brought on Board (Retained/Discarded)	Live - Stomach and/or Eyes Protruding	Discarded - Live and Healthy (Vented)	Not Applicable	
42	NOCAM	Epinephelus morio	Red Grouper	N	Brought on Board (Retained/Discarded)	Live - Stomach and/or Eyes Protruding	Discarded - Live and Healthy (Vented)	Not Applicable	
42	NOCAM	Epinephelus morio	Red Grouper	N	Brought on Board (Retained/Discarded)	Dead on Arrival - Damaged	Discarded - Dead	Not Applicable	
42	NOCAM	Epinephelus morio	Red Grouper	N	Brought on Board (Retained/Discarded)	Live - Healthy	Retained	Not Applicable	
42	NOCAM	Epinephelus morio	Red Grouper	N	Brought on Board (Retained/Discarded)	Live - Healthy	Retained	Not Applicable	
42	NOCAM	Epinephelus morio	Red Grouper	N	Brought on Board (Retained/Discarded)	Live - Stomach and/or Eyes Protruding	Discarded - Live and Healthy (Vented)	Not Applicable	
42	NOCAM	Epinephelus morio	Red Grouper	N	Brought on Board (Retained/Discarded)	Live - Healthy	Retained	Not Applicable	
42	NOCAM	Epinephelus morio	Red Grouper	N	Brought on Board (Retained/Discarded)	Live - Healthy	Discarded - Live and Healthy (Vented)	Not Applicable	
42	NOCAM	Epinephelus morio	Red Grouper	N	Brought on Board (Retained/Discarded)	Live - Healthy	Retained	Not Applicable	
42	NOCAM	Epinephelus morio	Red Grouper	N	Brought on Board (Retained/Discarded)	Live - Stomach and/or Eyes Protruding	Retained	Not Applicable	
42	NOCAM	Epinephelus morio	Red Grouper	N	Brought on Board (Retained/Discarded)	Live - Stomach and/or Eyes Protruding	Retained	Not Applicable	
42	NOCAM	Epinephelus morio	Red Grouper	N	Brought on Board (Retained/Discarded)	Live - Healthy	Retained	Not Applicable	
42	NOCAM	Epinephelus morio	Red Grouper	N	Brought on Board (Retained/Discarded)	Live - Healthy	Retained	Not Applicable	
42	NOCAM	Epinephelus morio	Red Grouper	N	Brought on Board (Retained/Discarded)	Live - Healthy	Retained	Not Applicable	
42	NOCAM	Epinephelus morio	Red Grouper	N	Brought on Board (Retained/Discarded)	Live - Healthy	Discarded - Live and Healthy (Vented)	Not Applicable	
42	NOCAM	Epinephelus morio	Red Grouper	N	Brought on Board (Retained/Discarded)	Live - Healthy	Discarded - Live and Healthy (Vented)	Not Applicable	
42	NOCAM	Epinephelus morio	Red Grouper	N	Brought on Board (Retained/Discarded)	Live - Stomach and/or Eyes Protruding	Retained	Not Applicable	

42	NOCAM	Epinephelus morio	Red Grouper	N	Brought on Board (Retained/Discarded)	Live - Stomach and/or Eyes Protruding	Retained	Not Applicable	
42	NOCAM	Epinephelus morio	Red Grouper	N	Brought on Board (Retained/Discarded)	Live - Healthy	Discarded - Live and Healthy (Vented)	Not Applicable	
42	NOCAM	Epinephelus morio	Red Grouper	N	Brought on Board (Retained/Discarded)	Live - Stomach and/or Eyes Protruding	Retained	Not Applicable	
42	NOCAM	Epinephelus morio	Red Grouper	N	Brought on Board (Retained/Discarded)	Live - Stomach and/or Eyes Protruding	Retained	Not Applicable	
42	NOCAM	Epinephelus morio	Red Grouper	N	Brought on Board (Retained/Discarded)	Live - Healthy	Discarded - Live and Healthy (Vented)	Not Applicable	
42	NOCAM	Epinephelus morio	Red Grouper	N	Brought on Board (Retained/Discarded)	Live - Healthy	Discarded - Live and Healthy (Vented)	Not Applicable	
42	NOCAM	Epinephelus morio	Red Grouper	N	Brought on Board (Retained/Discarded)	Live - Healthy	Discarded - Live and Healthy (Vented)	Not Applicable	
42	NOCAM	Epinephelus morio	Red Grouper	N	Brought on Board (Retained/Discarded)	Live - Stomach and/or Eyes Protruding	Retained	Not Applicable	
42	NOCAM	Epinephelus morio	Red Grouper	N	Brought on Board (Retained/Discarded)	Live - Stomach and/or Eyes Protruding	Discarded - Live and Healthy (Vented)	Not Applicable	
42	NOCAM	Epinephelus morio	Red Grouper	N	Brought on Board (Retained/Discarded)	Live - Healthy	Discarded - Live and Healthy (Vented)	Not Applicable	
42	NOCAM	Epinephelus morio	Red Grouper	N	Brought on Board (Retained/Discarded)	Live - Stomach and/or Eyes Protruding	Discarded - Live and Healthy (Vented)	Not Applicable	
42	NOCAM	Epinephelus morio	Red Grouper	N	Brought on Board (Retained/Discarded)	Live - Healthy	Discarded - Live and Healthy (Vented)	Not Applicable	
42	NOCAM	Epinephelus morio	Red Grouper	N	Brought on Board (Retained/Discarded)	Live - Stomach and/or Eyes Protruding	Retained	Not Applicable	
42	NOCAM	Epinephelus morio	Red Grouper	N	Brought on Board (Retained/Discarded)	Live - Healthy	Discarded - Live and Healthy (Vented)	Not Applicable	
42	NOCAM	Lutjanus analis	Mutton Snapper	N	Brought on Board (Retained/Discarded)	Live - Healthy	Retained	Not Applicable	
42	UCAM	Epinephelus morio	Red Grouper	N	Brought on Board (Retained/Discarded)	Live - Stomach and/or Eyes Protruding	Discarded - Live and Healthy (Vented)	Not Applicable	
42	UCAM	Epinephelus morio	Red Grouper	N	Brought on Board (Retained/Discarded)	Live - Healthy	Retained	Not Applicable	
42	UCAM	Epinephelus morio	Red Grouper	N	Brought on Board (Retained/Discarded)	Live - Stomach and/or Eyes Protruding	Retained	Not Applicable	
42	UCAM	Epinephelus morio	Red Grouper	N	Brought on Board (Retained/Discarded)	Live - Stomach and/or Eyes Protruding	Retained	Not Applicable	
42	UCAM	Epinephelus morio	Red Grouper	N	Brought on Board (Retained/Discarded)	Live - Healthy	Discarded - Live and Healthy (Vented)	Not Applicable	
42	UCAM	Epinephelus morio	Red Grouper	N	Brought on Board (Retained/Discarded)	Live - Stomach and/or Eyes Protruding	Retained	Not Applicable	
42	UCAM	Epinephelus morio	Red Grouper	N	Brought on Board (Retained/Discarded)	Live - Healthy	Discarded - Live and Healthy (Vented)	Not Applicable	
42	UCAM	Epinephelus morio	Red Grouper	N	Brought on Board (Retained/Discarded)	Live - Healthy	Discarded - Live and Healthy (Vented)	Not Applicable	
42	UCAM	Epinephelus morio	Red Grouper	N	Brought on Board (Retained/Discarded)	Live - Healthy	Retained	Not Applicable	
42	UCAM	Epinephelus morio	Red Grouper	N	Brought on Board (Retained/Discarded)	Live - Healthy	Discarded - Live and Healthy (Vented)	Not Applicable	
42	UCAM	Epinephelus morio	Red Grouper	N	Brought on Board (Retained/Discarded)	Live - Healthy	Retained	Not Applicable	
42	UCAM	Epinephelus morio	Red Grouper	N	Brought on Board (Retained/Discarded)	Live - Stomach and/or Eyes Protruding	Retained	Not Applicable	
42	UCAM	Epinephelus morio	Red Grouper	N	Brought on Board (Retained/Discarded)	Live - Healthy	Discarded - Live and Healthy (Vented)	Not Applicable	
42	UCAM	Epinephelus morio	Red Grouper	N	Brought on Board (Retained/Discarded)	Live - Stomach and/or Eyes Protruding	Discarded - Live and Healthy (Vented)	Not Applicable	
42	UCAM	Epinephelus morio	Red Grouper	N	Brought on Board (Retained/Discarded)	Live - Healthy	Retained	Not Applicable	
42	UCAM	Epinephelus morio	Red Grouper	N	Brought on Board (Retained/Discarded)	Live - Healthy	Retained	Not Applicable	
42	UCAM	Epinephelus morio	Red Grouper	N	Brought on Board (Retained/Discarded)	Live - Stomach and/or Eyes Protruding	Retained	Not Applicable	
42	UCAM	Epinephelus morio	Red Grouper	N	Brought on Board (Retained/Discarded)	Live - Stomach and/or Eyes Protruding	Retained	Not Applicable	
42	UCAM	Epinephelus morio	Red Grouper	N	Brought on Board (Retained/Discarded)	Live - Healthy	Discarded - Live and Healthy (Vented)	Not Applicable	
42	UCAM	Epinephelus morio	Red Grouper	N	Brought on Board (Retained/Discarded)	Live - Stomach and/or Eyes Protruding	Discarded - Live and Healthy (Vented)	Not Applicable	
42	UCAM	Epinephelus morio	Red Grouper	N	Brought on Board (Retained/Discarded)	Live - Stomach and/or Eyes Protruding	Retained	Not Applicable	
42	UCAM	Epinephelus morio	Red Grouper	N	Brought on Board (Retained/Discarded)	Live - Healthy	Retained	Not Applicable	
42	UCAM	Epinephelus morio	Red Grouper	N	Brought on Board (Retained/Discarded)	Dead on Arrival - Damaged	Discarded - Dead	Not Applicable	
42	UCAM	Epinephelus morio	Red Grouper	N	Brought on Board (Retained/Discarded)	Live - Stomach and/or Eyes Protruding	Discarded - Live and Healthy (Vented)	Not Applicable	
42	UCAM	Epinephelus morio	Red Grouper	N	Brought on Board (Retained/Discarded)	Live - Stomach and/or Eyes Protruding	Retained	Not Applicable	
42	UCAM	Epinephelus morio	Red Grouper	N	Brought on Board (Retained/Discarded)	Live - Healthy	Discarded - Live and Healthy (Vented)	Not Applicable	
42	UCAM	Epinephelus morio	Red Grouper	N	Brought on Board (Retained/Discarded)	Live - Stomach and/or Eyes Protruding	Discarded - Live and Healthy (Vented)	Not Applicable	
42	UCAM	Epinephelus morio	Red Grouper	N	Brought on Board (Retained/Discarded)	Live - Stomach and/or Eyes Protruding	Retained	Not Applicable	
42	UCAM	Epinephelus morio	Red Grouper	N	Not Handled (Dropped Off)	Live - Healthy	Discarded - Unknown	Not Applicable	
42	UCAM	Epinephelus morio	Red Grouper	N	Brought on Board (Retained/Discarded)	Live - Stomach and/or Eyes Protruding	Retained	Not Applicable	
42	UCAM	Epinephelus morio	Red Grouper	N	Brought on Board (Retained/Discarded)	Live - Healthy	Retained	Not Applicable	
42	UCAM	Epinephelus morio	Red Grouper	N	Brought on Board (Retained/Discarded)	Live - Healthy	Discarded - Live and Healthy (Vented)	Not Applicable	
42	UCAM	Epinephelus morio	Red Grouper	N	Brought on Board (Retained/Discarded)	Live - Stomach and/or Eyes Protruding	Discarded - Live and Healthy (Vented)	Not Applicable	
42	UCAM	Epinephelus morio	Red Grouper	N	Brought on Board (Retained/Discarded)	Live - Healthy	Discarded - Live and Healthy (Vented)	Not Applicable	
42	UCAM	Epinephelus morio	Red Grouper	N	Brought on Board (Retained/Discarded)	Live - Healthy	Retained	Not Applicable	
42	UCAM	Epinephelus morio	Red Grouper	N	Brought on Board (Retained/Discarded)	Live - Stomach and/or Eyes Protruding	Discarded - Live and Healthy (Vented)	Not Applicable	
42	UCAM	Epinephelus morio	Red Grouper	N	Brought on Board (Retained/Discarded)	Live - Healthy	Retained	Not Applicable	
42	UCAM	Lutjanus analis	Mutton Snapper	N	Brought on Board (Retained/Discarded)	Live - Healthy	Retained	Not Applicable	
43	NOCAM	Calamus bajonada	Jolthead Porgy	N	Brought on Board (Retained/Discarded)	Live - Healthy	Retained	Not Applicable	
43	NOCAM	Calamus bajonada	Jolthead Porgy	N	Brought on Board (Retained/Discarded)	Live - Healthy	Retained	Not Applicable	
43	NOCAM	Epinephelus morio	Red Grouper	N	Brought on Board (Retained/Discarded)	Live - Healthy	Retained	Not Applicable	
43	NOCAM	Epinephelus morio	Red Grouper	N	Brought on Board (Retained/Discarded)	Live - Stomach and/or Eyes Protruding	Retained	Not Applicable	
43	NOCAM	Epinephelus morio	Red Grouper	N	Brought on Board (Retained/Discarded)	Live - Healthy	Retained	Not Applicable	
43	NOCAM	Epinephelus morio	Red Grouper	N	Brought on Board (Retained/Discarded)	Live - Healthy	Retained	Not Applicable	
43	NOCAM	Epinephelus morio	Red Grouper	N	Brought on Board (Retained/Discarded)	Live - Stomach and/or Eyes Protruding	Retained	Not Applicable	
43	NOCAM	Epinephelus morio	Red Grouper	N	Brought on Board (Retained/Discarded)	Live - Healthy	Discarded - Live and Healthy (Vented)	Not Applicable	
43	NOCAM	Epinephelus morio	Red Grouper	N	Brought on Board (Retained/Discarded)	Live - Healthy	Retained	Not Applicable	
43	NOCAM	Lutjanus analis	Mutton Snapper	N	Brought on Board (Retained/Discarded)	Live - Healthy	Retained	Not Applicable	
43	NOCAM	Lutjanus analis	Mutton Snapper	N	Brought on Board (Retained/Discarded)	Live - Healthy	Retained	Not Applicable	
43	NOCAM	Lutjanus campechanus	Red Snapper	N	Brought on Board (Retained/Discarded)	Live - Stomach and/or Eyes Protruding	Retained	Not Applicable	
43	NOCAM	Lutjanus campechanus	Red Snapper	N	Brought on Board (Retained/Discarded)	Live - Healthy	Retained	Not Applicable	
43	NOCAM	Lutjanus campechanus	Red Snapper	N	Brought on Board (Retained/Discarded)	Live - Healthy	Retained	Not Applicable	
43	NOCAM	Lutjanus campechanus	Red Snapper	N	Brought on Board (Retained/Discarded)	Live - Healthy	Retained	Not Applicable	
43	NOCAM	Lutjanus campechanus	Red Snapper	N	Brought on Board (Retained/Discarded)	Live - Healthy	Retained	Not Applicable	
43	NOCAM	Lutjanus campechanus	Red Snapper	N	Brought on Board (Retained/Discarded)	Live - Healthy	Retained	Not Applicable	
43	NOCAM	Lutjanus campechanus	Red Snapper	N	Brought on Board (Retained/Discarded)	Live - Healthy	Retained	Not Applicable	
43	NOCAM	Lutjanus campechanus	Red Snapper	N	Brought on Board (Retained/Discarded)	Live - Stomach and/or Eyes Protruding	Retained	Not Applicable	
43	NOCAM	Lutjanus campechanus	Red Snapper	N	Brought on Board (Retained/Discarded)	Live - Healthy	Retained	Not Applicable	
43	NOCAM	Lutjanus campechanus	Red Snapper	N	Brought on Board (Retained/Discarded)	Live - Healthy	Retained	Not Applicable	
43	NOCAM	Lutjanus campechanus	Red Snapper	N	Brought on Board (Retained/Discarded)	Live - Healthy	Retained	Not Applicable	
43	NOCAM	Lutjanus campechanus	Red Snapper	N	Brought on Board (Retained/Discarded)	Live - Healthy	Retained	Not Applicable	
43	NOCAM	Lutjanus campechanus	Red Snapper	N	Brought on Board (Retained/Discarded)	Live - Healthy	Retained	Not Applicable	
43	NOCAM	Lutjanus campechanus	Red Snapper	N	Brought on Board (Retained/Discarded)	Live - Healthy	Retained	Not Applicable	
43	NOCAM	Lutjanus campechanus	Red Snapper	N	Brought on Board (Retained/Discarded)	Live - Healthy	Retained	Not Applicable	
43	NOCAM	Lutjanus campechanus	Red Snapper	N	Brought on Board (Retained/Discarded)	Live - Healthy	Retained	Not Applicable	
43	NOCAM	Lutjanus campechanus	Red Snapper	N	Brought on Board (Retained/Discarded)	Live - Stomach and/or Eyes Protruding	Retained	Not Applicable	
43	NOCAM	Lutjanus campechanus	Red Snapper	N	Brought on Board (Retained/Discarded)	Live - Healthy	Retained	Not Applicable	
43	NOCAM	Lutjanus campechanus	Red Snapper	N	Brought on Board (Retained/Discarded)	Live - Healthy	Retained	Not Applicable	
43	NOCAM	Lutjanus campechanus	Red Snapper	N	Brought on Board (Retained/Discarded)	Live - Healthy	Retained	Not Applicable	
43	NOCAM	Lutjanus campechanus	Red Snapper	N	Brought on Board (Retained/Discarded)	Live - Healthy	Retained	Not Applicable	
43	NOCAM	Lutjanus campechanus	Red Snapper	N	Brought on Board (Retained/Discarded)	Live - Healthy	Retained	Not Applicable	
43	NOCAM	Lutjanus campechanus	Red Snapper	N	Brought on Board (Retained/Discarded)	Live - Healthy	Retained	Not Applicable	
43	NOCAM	Lutjanus campechanus	Red Snapper	N	Brought on Board (Retained/Discarded)	Live - Healthy	Retained	Not Applicable	
43	NOCAM	Lutjanus campechanus	Red Snapper	N	Brought on Board (Retained/Discarded)	Live - Stomach and/or Eyes Protruding	Retained	Not Applicable	
43	NOCAM	Lutjanus campechanus	Red Snapper	N	Brought on Board (Retained/Discarded)	Live - Healthy	Retained	Not Applicable	
43	NOCAM	Lutjanus campechanus	Red Snapper	N	Brought on Board (Retained/Discarded)	Live - Healthy	Retained	Not Applicable	
43	NOCAM	Lutjanus campechanus	Red Snapper	N	Brought on Board (Retained/Discarded)	Live - Healthy	Retained	Not Applicable	

43	NOCAM	Lutjanus campechanus	Red Snapper	N	Brought on Board (Retained/Discarded)	Live - Healthy	Retained	Not Applicable	
43	NOCAM	Rhizoprionodon terraenovae	Atlantic Sharpnose Shark	Y	Brought on Board (Retained/Discarded)	Dead on Arrival - Damaged	Discarded - Dead	Unknown Maturity and/or Sex	Small (Less than 1m)
43	UCAM	Calamus bajonada	Jolthead Porgy	N	Brought on Board (Retained/Discarded)	Live - Healthy	Retained	Not Applicable	
43	UCAM	Calamus bajonada	Jolthead Porgy	N	Brought on Board (Retained/Discarded)	Live - Healthy	Retained	Not Applicable	
43	UCAM	Epinephelus morio	Red Grouper	N	Brought on Board (Retained/Discarded)	Live - Healthy	Retained	Not Applicable	
43	UCAM	Epinephelus morio	Red Grouper	N	Brought on Board (Retained/Discarded)	Live - Stomach and/or Eyes Protruding	Retained	Not Applicable	
43	UCAM	Epinephelus morio	Red Grouper	N	Brought on Board (Retained/Discarded)	Live - Stomach and/or Eyes Protruding	Retained	Not Applicable	
43	UCAM	Epinephelus morio	Red Grouper	N	Brought on Board (Retained/Discarded)	Live - Healthy	Discarded - Live and Healthy (Vented)	Not Applicable	
43	UCAM	Epinephelus morio	Red Grouper	N	Brought on Board (Retained/Discarded)	Live - Healthy	Retained	Not Applicable	
43	UCAM	Epinephelus morio	Red Grouper	N	Brought on Board (Retained/Discarded)	Live - Stomach and/or Eyes Protruding	Retained	Not Applicable	
43	UCAM	Epinephelus morio	Red Grouper	N	Brought on Board (Retained/Discarded)	Live - Stomach and/or Eyes Protruding	Retained	Not Applicable	
43	UCAM	Lutjanus analis	Mutton Snapper	N	Brought on Board (Retained/Discarded)	Live - Healthy	Retained	Not Applicable	
43	UCAM	Lutjanus analis	Mutton Snapper	N	Brought on Board (Retained/Discarded)	Live - Healthy	Retained	Not Applicable	
43	UCAM	Lutjanus campechanus	Red Snapper	N	Brought on Board (Retained/Discarded)	Live - Healthy	Retained	Not Applicable	
43	UCAM	Lutjanus campechanus	Red Snapper	N	Brought on Board (Retained/Discarded)	Live - Healthy	Retained	Not Applicable	
43	UCAM	Lutjanus campechanus	Red Snapper	N	Brought on Board (Retained/Discarded)	Live - Healthy	Retained	Not Applicable	
43	UCAM	Lutjanus campechanus	Red Snapper	N	Brought on Board (Retained/Discarded)	Live - Healthy	Retained	Not Applicable	
43	UCAM	Lutjanus campechanus	Red Snapper	N	Brought on Board (Retained/Discarded)	Live - Healthy	Retained	Not Applicable	
43	UCAM	Lutjanus campechanus	Red Snapper	N	Brought on Board (Retained/Discarded)	Live - Healthy	Retained	Not Applicable	
43	UCAM	Lutjanus campechanus	Red Snapper	N	Brought on Board (Retained/Discarded)	Live - Healthy	Retained	Not Applicable	
43	UCAM	Lutjanus campechanus	Red Snapper	N	Brought on Board (Retained/Discarded)	Live - Healthy	Retained	Not Applicable	
43	UCAM	Lutjanus campechanus	Red Snapper	N	Brought on Board (Retained/Discarded)	Live - Healthy	Retained	Not Applicable	
43	UCAM	Lutjanus campechanus	Red Snapper	N	Brought on Board (Retained/Discarded)	Live - Stomach and/or Eyes Protruding	Retained	Not Applicable	
43	UCAM	Lutjanus campechanus	Red Snapper	N	Brought on Board (Retained/Discarded)	Live - Healthy	Retained	Not Applicable	
43	UCAM	Lutjanus campechanus	Red Snapper	N	Brought on Board (Retained/Discarded)	Live - Healthy	Retained	Not Applicable	
43	UCAM	Lutjanus campechanus	Red Snapper	N	Brought on Board (Retained/Discarded)	Live - Healthy	Retained	Not Applicable	
43	UCAM	Lutjanus campechanus	Red Snapper	N	Brought on Board (Retained/Discarded)	Live - Healthy	Retained	Not Applicable	
43	UCAM	Lutjanus campechanus	Red Snapper	N	Brought on Board (Retained/Discarded)	Live - Healthy	Retained	Not Applicable	
43	UCAM	Lutjanus campechanus	Red Snapper	N	Brought on Board (Retained/Discarded)	Live - Healthy	Retained	Not Applicable	
43	UCAM	Lutjanus campechanus	Red Snapper	N	Brought on Board (Retained/Discarded)	Live - Healthy	Retained	Not Applicable	
43	UCAM	Lutjanus campechanus	Red Snapper	N	Brought on Board (Retained/Discarded)	Live - Healthy	Retained	Not Applicable	
43	UCAM	Lutjanus campechanus	Red Snapper	N	Brought on Board (Retained/Discarded)	Live - Healthy	Retained	Not Applicable	
43	UCAM	Lutjanus campechanus	Red Snapper	N	Brought on Board (Retained/Discarded)	Live - Healthy	Retained	Not Applicable	
43	UCAM	Lutjanus campechanus	Red Snapper	N	Brought on Board (Retained/Discarded)	Live - Stomach and/or Eyes Protruding	Retained	Not Applicable	
43	UCAM	Lutjanus campechanus	Red Snapper	N	Brought on Board (Retained/Discarded)	Live - Healthy	Retained	Not Applicable	
43	UCAM	Lutjanus campechanus	Red Snapper	N	Brought on Board (Retained/Discarded)	Live - Healthy	Retained	Not Applicable	
43	UCAM	Lutjanus campechanus	Red Snapper	N	Brought on Board (Retained/Discarded)	Live - Healthy	Retained	Not Applicable	
43	UCAM	Lutjanus campechanus	Red Snapper	N	Brought on Board (Retained/Discarded)	Live - Stomach and/or Eyes Protruding	Retained	Not Applicable	
43	UCAM	Lutjanus campechanus	Red Snapper	N	Brought on Board (Retained/Discarded)	Live - Healthy	Retained	Not Applicable	
43	UCAM	Lutjanus campechanus	Red Snapper	N	Brought on Board (Retained/Discarded)	Live - Healthy	Retained	Not Applicable	
43	UCAM	Lutjanus campechanus	Red Snapper	N	Brought on Board (Retained/Discarded)	Live - Healthy	Retained	Not Applicable	
43	UCAM	Lutjanus campechanus	Red Snapper	N	Brought on Board (Retained/Discarded)	Live - Healthy	Retained	Not Applicable	
43	UCAM	Rhizoprionodon terraenovae	Atlantic Sharpnose Shark	Y	Brought on Board (Retained/Discarded)	Dead on Arrival - Damaged	Discarded - Dead	Unknown Maturity and/or Sex	Small (Less than 1m)
44	NOCAM	Anguilliformes, unidentified	Eel, Unidentified	N	Brought on Board (Retained/Discarded)	Live - Healthy	Retained as Bait	Not Applicable	
44	NOCAM	Caulolatilus microps	Blueline Tilefish	N	Brought on Board (Retained/Discarded)	Live - Healthy	Retained	Not Applicable	
44	NOCAM	Caulolatilus microps	Blueline Tilefish	N	Brought on Board (Retained/Discarded)	Live - Healthy	Retained	Not Applicable	
44	NOCAM	Caulolatilus microps	Blueline Tilefish	N	Brought on Board (Retained/Discarded)	Live - Healthy	Retained	Not Applicable	
44	NOCAM	Caulolatilus microps	Blueline Tilefish	N	Brought on Board (Retained/Discarded)	Live - Healthy	Retained	Not Applicable	
44	NOCAM	Caulolatilus microps	Blueline Tilefish	N	Brought on Board (Retained/Discarded)	Live - Stomach and/or Eyes Protruding	Retained	Not Applicable	
44	NOCAM	Caulolatilus microps	Blueline Tilefish	N	Brought on Board (Retained/Discarded)	Live - Healthy	Retained	Not Applicable	
44	NOCAM	Epinephelus flavolimbatus	Yellowedge Grouper	N	Brought on Board (Retained/Discarded)	Live - Stomach and/or Eyes Protruding	Retained	Not Applicable	
44	NOCAM	Epinephelus flavolimbatus	Yellowedge Grouper	N	Brought on Board (Retained/Discarded)	Live - Stomach and/or Eyes Protruding	Retained	Not Applicable	
44	NOCAM	Epinephelus flavolimbatus	Yellowedge Grouper	N	Brought on Board (Retained/Discarded)	Live - Stomach and/or Eyes Protruding	Retained	Not Applicable	
44	NOCAM	Epinephelus flavolimbatus	Yellowedge Grouper	N	Brought on Board (Retained/Discarded)	Live - Stomach and/or Eyes Protruding	Retained	Not Applicable	
44	NOCAM	Epinephelus flavolimbatus	Yellowedge Grouper	N	Brought on Board (Retained/Discarded)	Live - Stomach and/or Eyes Protruding	Retained	Not Applicable	
44	NOCAM	Epinephelus flavolimbatus	Yellowedge Grouper	N	Brought on Board (Retained/Discarded)	Live - Stomach and/or Eyes Protruding	Retained	Not Applicable	
44	NOCAM	Epinephelus flavolimbatus	Yellowedge Grouper	N	Brought on Board (Retained/Discarded)	Live - Stomach and/or Eyes Protruding	Retained	Not Applicable	
44	NOCAM	Muraenidae, unidentified	Moray Eel, Unidentified	N	Brought on Board (Retained/Discarded)	Live - Healthy	Retained as Bait	Not Applicable	
44	NOCAM	Muraenidae, unidentified	Moray Eel, Unidentified	N	Brought on Board (Retained/Discarded)	Live - Healthy	Retained as Bait	Not Applicable	
44	UCAM	Anguilliformes, unidentified	Eel, Unidentified	N	Brought on Board (Retained/Discarded)	Live - Healthy	Retained as Bait	Not Applicable	
44	UCAM	Caulolatilus microps	Blueline Tilefish	N	Brought on Board (Retained/Discarded)	Live - Healthy	Retained	Not Applicable	
44	UCAM	Caulolatilus microps	Blueline Tilefish	N	Brought on Board (Retained/Discarded)	Live - Stomach and/or Eyes Protruding	Retained	Not Applicable	
44	UCAM	Caulolatilus microps	Blueline Tilefish	N	Brought on Board (Retained/Discarded)	Live - Healthy	Retained	Not Applicable	
44	UCAM	Caulolatilus microps	Blueline Tilefish	N	Brought on Board (Retained/Discarded)	Live - Stomach and/or Eyes Protruding	Retained	Not Applicable	
44	UCAM	Caulolatilus microps	Blueline Tilefish	N	Brought on Board (Retained/Discarded)	Live - Healthy	Retained	Not Applicable	
44	UCAM	Caulolatilus microps	Blueline Tilefish	N	Brought on Board (Retained/Discarded)	Live - Healthy	Retained	Not Applicable	
44	UCAM	Epinephelus flavolimbatus	Yellowedge Grouper	N	Brought on Board (Retained/Discarded)	Live - Stomach and/or Eyes Protruding	Retained	Not Applicable	
44	UCAM	Epinephelus flavolimbatus	Yellowedge Grouper	N	Brought on Board (Retained/Discarded)	Live - Stomach and/or Eyes Protruding	Retained	Not Applicable	
44	UCAM	Epinephelus flavolimbatus	Yellowedge Grouper	N	Brought on Board (Retained/Discarded)	Live - Stomach and/or Eyes Protruding	Retained	Not Applicable	
44	UCAM	Epinephelus flavolimbatus	Yellowedge Grouper	N	Brought on Board (Retained/Discarded)	Live - Stomach and/or Eyes Protruding	Retained	Not Applicable	
44	UCAM	Epinephelus flavolimbatus	Yellowedge Grouper	N	Brought on Board (Retained/Discarded)	Live - Stomach and/or Eyes Protruding	Retained	Not Applicable	
44	UCAM	Epinephelus flavolimbatus	Yellowedge Grouper	N	Brought on Board (Retained/Discarded)	Live - Stomach and/or Eyes Protruding	Retained	Not Applicable	
44	UCAM	Epinephelus flavolimbatus	Yellowedge Grouper	N	Brought on Board (Retained/Discarded)	Live - Stomach and/or Eyes Protruding	Retained	Not Applicable	
44	UCAM	Muraenidae, unidentified	Moray Eel, Unidentified	N	Brought on Board (Retained/Discarded)	Live - Healthy	Retained as Bait	Not Applicable	
44	UCAM	Muraenidae, unidentified	Moray Eel, Unidentified	N	Brought on Board (Retained/Discarded)	Live - Healthy	Retained as Bait	Not Applicable	
45	NOCAM	Anguilliformes, unidentified	Eel, Unidentified	N	Brought on Board (Retained/Discarded)	Live - Healthy	Retained as Bait	Not Applicable	
45	NOCAM	Anguilliformes, unidentified	Eel, Unidentified	N	Brought on Board (Retained/Discarded)	Live - Healthy	Retained as Bait	Not Applicable	
45	NOCAM	Caulolatilus microps	Blueline Tilefish	N	Brought on Board (Retained/Discarded)	Live - Healthy	Retained	Not Applicable	
45	NOCAM	Epinephelus flavolimbatus	Yellowedge Grouper	N	Brought on Board (Retained/Discarded)	Live - Stomach and/or Eyes Protruding	Retained	Not Applicable	
45	NOCAM	Epinephelus flavolimbatus	Yellowedge Grouper	N	Brought on Board (Retained/Discarded)	Live - Stomach and/or Eyes Protruding	Retained	Not Applicable	
45	NOCAM	Epinephelus flavolimbatus	Yellowedge Grouper	N	Brought on Board (Retained/Discarded)	Live - Stomach and/or Eyes Protruding	Retained	Not Applicable	
45	NOCAM	Epinephelus flavolimbatus	Yellowedge Grouper	N	Brought on Board (Retained/Discarded)	Live - Stomach and/or Eyes Protruding	Retained	Not Applicable	
45	NOCAM	Muraenidae, unidentified	Moray Eel, Unidentified	N	Brought on Board (Retained/Discarded)	Live - Healthy	Retained as Bait	Not Applicable	
45	NOCAM	Muraenidae, unidentified	Moray Eel, Unidentified	N	Brought on Board (Retained/Discarded)	Live - Healthy	Retained as Bait	Not Applicable	
45	UCAM	Anguilliformes, unidentified	Eel, Unidentified	N	Brought on Board (Retained/Discarded)	Live - Healthy	Retained as Bait	Not Applicable	
45	UCAM	Anguilliformes, unidentified	Eel, Unidentified	N	Brought on Board (Retained/Discarded)	Live - Healthy	Retained as Bait	Not Applicable	
45	UCAM	Caulolatilus microps	Blueline Tilefish	N	Brought on Board (Retained/Discarded)	Live - Healthy	Retained	Not Applicable	
45	UCAM	Epinephelus flavolimbatus	Yellowedge Grouper	N	Brought on Board (Retained/Discarded)	Live - Stomach and/or Eyes Protruding	Retained	Not Applicable	
45	UCAM	Epinephelus flavolimbatus	Yellowedge Grouper	N	Brought on Board (Retained/Discarded)	Live - Stomach and/or Eyes Protruding	Retained	Not Applicable	
45	UCAM	Epinephelus flavolimbatus	Yellowedge Grouper	N	Brought on Board (Retained/Discarded)	Live - Stomach and/or Eyes Protruding	Retained	Not Applicable	
45	UCAM	Epinephelus flavolimbatus	Yellowedge Grouper	N	Brought on Board (Retained/Discarded)	Live - Stomach and/or Eyes Protruding	Retained	Not Applicable	
45	UCAM	Muraenidae, unidentified	Moray Eel, Unidentified	N	Brought on Board (Retained/Discarded)	Live - Healthy	Retained as Bait	Not Applicable	

45	UCAM	Muraenidae, unidentified	Moray Eel, Unidentified	N	Brought on Board (Retained/Discarded)	Live - Healthy	Retained as Bait	Not Applicable	
46	NOCAM	Caulolatilus microps	Blueline Tilefish	N	Brought on Board (Retained/Discarded)	Live - Healthy	Retained	Not Applicable	
46	NOCAM	Epinephelus flavolimbatus	Yellowedge Grouper	N	Brought on Board (Retained/Discarded)	Live - Stomach and/or Eyes Protruding	Retained	Not Applicable	
46	NOCAM	Epinephelus flavolimbatus	Yellowedge Grouper	N	Brought on Board (Retained/Discarded)	Live - Stomach and/or Eyes Protruding	Retained	Not Applicable	
46	NOCAM	Epinephelus flavolimbatus	Yellowedge Grouper	N	Brought on Board (Retained/Discarded)	Live - Stomach and/or Eyes Protruding	Retained	Not Applicable	
46	NOCAM	Epinephelus flavolimbatus	Yellowedge Grouper	N	Brought on Board (Retained/Discarded)	Live - Stomach and/or Eyes Protruding	Retained	Not Applicable	
46	NOCAM	Epinephelus flavolimbatus	Yellowedge Grouper	N	Brought on Board (Retained/Discarded)	Live - Healthy	Retained	Not Applicable	
46	NOCAM	Epinephelus flavolimbatus	Yellowedge Grouper	N	Brought on Board (Retained/Discarded)	Live - Stomach and/or Eyes Protruding	Retained	Not Applicable	
46	NOCAM	Epinephelus flavolimbatus	Yellowedge Grouper	N	Brought on Board (Retained/Discarded)	Live - Stomach and/or Eyes Protruding	Retained	Not Applicable	
46	NOCAM	Epinephelus flavolimbatus	Yellowedge Grouper	N	Brought on Board (Retained/Discarded)	Live - Stomach and/or Eyes Protruding	Retained	Not Applicable	
46	NOCAM	Epinephelus flavolimbatus	Yellowedge Grouper	N	Brought on Board (Retained/Discarded)	Live - Healthy	Retained	Not Applicable	
46	NOCAM	Epinephelus flavolimbatus	Yellowedge Grouper	N	Brought on Board (Retained/Discarded)	Live - Stomach and/or Eyes Protruding	Retained	Not Applicable	
46	NOCAM	Epinephelus flavolimbatus	Yellowedge Grouper	N	Brought on Board (Retained/Discarded)	Dead on Arrival - Undamaged	Retained	Not Applicable	
46	NOCAM	Epinephelus flavolimbatus	Yellowedge Grouper	N	Brought on Board (Retained/Discarded)	Live - Stomach and/or Eyes Protruding	Retained	Not Applicable	
46	NOCAM	Epinephelus flavolimbatus	Yellowedge Grouper	N	Brought on Board (Retained/Discarded)	Live - Stomach and/or Eyes Protruding	Retained	Not Applicable	
46	NOCAM	Epinephelus flavolimbatus	Yellowedge Grouper	N	Brought on Board (Retained/Discarded)	Live - Healthy	Retained	Not Applicable	
46	NOCAM	Epinephelus flavolimbatus	Yellowedge Grouper	N	Brought on Board (Retained/Discarded)	Live - Stomach and/or Eyes Protruding	Retained	Not Applicable	
46	NOCAM	Pagrus pagrus	Red Porgy	N	Brought on Board (Retained/Discarded)	Live - Healthy	Retained	Not Applicable	
46	UCAM	Caulolatilus microps	Blueline Tilefish	N	Brought on Board (Retained/Discarded)	Live - Healthy	Retained	Not Applicable	
46	UCAM	Crab, unidentified	Crab, Unidentified	N	Brought on Board (Retained/Discarded)	Live - Healthy	Discarded - Live and Healthy (Not Vented)	Not Applicable	
46	UCAM	Epinephelus flavolimbatus	Yellowedge Grouper	N	Brought on Board (Retained/Discarded)	Live - Stomach and/or Eyes Protruding	Retained	Not Applicable	
46	UCAM	Epinephelus flavolimbatus	Yellowedge Grouper	N	Brought on Board (Retained/Discarded)	Dead on Arrival - Undamaged	Retained	Not Applicable	
46	UCAM	Epinephelus flavolimbatus	Yellowedge Grouper	N	Brought on Board (Retained/Discarded)	Live - Healthy	Retained	Not Applicable	
46	UCAM	Epinephelus flavolimbatus	Yellowedge Grouper	N	Brought on Board (Retained/Discarded)	Live - Stomach and/or Eyes Protruding	Retained	Not Applicable	
46	UCAM	Epinephelus flavolimbatus	Yellowedge Grouper	N	Brought on Board (Retained/Discarded)	Live - Stomach and/or Eyes Protruding	Retained	Not Applicable	
46	UCAM	Epinephelus flavolimbatus	Yellowedge Grouper	N	Brought on Board (Retained/Discarded)	Live - Stomach and/or Eyes Protruding	Retained	Not Applicable	
46	UCAM	Epinephelus flavolimbatus	Yellowedge Grouper	N	Brought on Board (Retained/Discarded)	Live - Healthy	Retained	Not Applicable	
46	UCAM	Epinephelus flavolimbatus	Yellowedge Grouper	N	Brought on Board (Retained/Discarded)	Live - Healthy	Retained	Not Applicable	
46	UCAM	Epinephelus flavolimbatus	Yellowedge Grouper	N	Brought on Board (Retained/Discarded)	Live - Stomach and/or Eyes Protruding	Retained	Not Applicable	
46	UCAM	Epinephelus flavolimbatus	Yellowedge Grouper	N	Brought on Board (Retained/Discarded)	Live - Stomach and/or Eyes Protruding	Retained	Not Applicable	
46	UCAM	Epinephelus flavolimbatus	Yellowedge Grouper	N	Brought on Board (Retained/Discarded)	Live - Stomach and/or Eyes Protruding	Retained	Not Applicable	
46	UCAM	Epinephelus flavolimbatus	Yellowedge Grouper	N	Brought on Board (Retained/Discarded)	Live - Stomach and/or Eyes Protruding	Retained	Not Applicable	
46	UCAM	Epinephelus flavolimbatus	Yellowedge Grouper	N	Brought on Board (Retained/Discarded)	Live - Stomach and/or Eyes Protruding	Retained	Not Applicable	
46	UCAM	Epinephelus flavolimbatus	Yellowedge Grouper	N	Brought on Board (Retained/Discarded)	Live - Stomach and/or Eyes Protruding	Retained	Not Applicable	
46	UCAM	Epinephelus flavolimbatus	Yellowedge Grouper	N	Brought on Board (Retained/Discarded)	Live - Stomach and/or Eyes Protruding	Retained	Not Applicable	
46	UCAM	Pagrus pagrus	Red Porgy	N	Brought on Board (Retained/Discarded)	Live - Healthy	Retained	Not Applicable	
47	NOCAM	Caulolatilus microps	Blueline Tilefish	N	Brought on Board (Retained/Discarded)	Live - Healthy	Retained	Not Applicable	
47	NOCAM	Caulolatilus microps	Blueline Tilefish	N	Brought on Board (Retained/Discarded)	Live - Healthy	Retained	Not Applicable	
47	NOCAM	Caulolatilus microps	Blueline Tilefish	N	Brought on Board (Retained/Discarded)	Live - Stomach and/or Eyes Protruding	Retained	Not Applicable	
47	NOCAM	Caulolatilus microps	Blueline Tilefish	N	Brought on Board (Retained/Discarded)	Live - Healthy	Retained	Not Applicable	
47	NOCAM	Caulolatilus microps	Blueline Tilefish	N	Brought on Board (Retained/Discarded)	Live - Healthy	Retained	Not Applicable	
47	NOCAM	Caulolatilus microps	Blueline Tilefish	N	Brought on Board (Retained/Discarded)	Live - Healthy	Retained	Not Applicable	
47	NOCAM	Epinephelus flavolimbatus	Yellowedge Grouper	N	Brought on Board (Retained/Discarded)	Live - Stomach and/or Eyes Protruding	Retained	Not Applicable	
47	NOCAM	Epinephelus flavolimbatus	Yellowedge Grouper	N	Brought on Board (Retained/Discarded)	Live - Stomach and/or Eyes Protruding	Retained	Not Applicable	
47	NOCAM	Epinephelus flavolimbatus	Yellowedge Grouper	N	Brought on Board (Retained/Discarded)	Live - Stomach and/or Eyes Protruding	Retained	Not Applicable	
47	NOCAM	Epinephelus flavolimbatus	Yellowedge Grouper	N	Brought on Board (Retained/Discarded)	Live - Stomach and/or Eyes Protruding	Retained	Not Applicable	
47	NOCAM	Epinephelus flavolimbatus	Yellowedge Grouper	N	Brought on Board (Retained/Discarded)	Live - Stomach and/or Eyes Protruding	Retained	Not Applicable	
47	NOCAM	Epinephelus flavolimbatus	Yellowedge Grouper	N	Brought on Board (Retained/Discarded)	Live - Stomach and/or Eyes Protruding	Retained	Not Applicable	
47	NOCAM	Epinephelus flavolimbatus	Yellowedge Grouper	N	Brought on Board (Retained/Discarded)	Live - Stomach and/or Eyes Protruding	Retained	Not Applicable	
47	NOCAM	Epinephelus flavolimbatus	Yellowedge Grouper	N	Brought on Board (Retained/Discarded)	Live - Stomach and/or Eyes Protruding	Retained	Not Applicable	
47	NOCAM	Epinephelus flavolimbatus	Yellowedge Grouper	N	Brought on Board (Retained/Discarded)	Live - Healthy	Retained	Not Applicable	
47	NOCAM	Epinephelus flavolimbatus	Yellowedge Grouper	N	Brought on Board (Retained/Discarded)	Live - Stomach and/or Eyes Protruding	Retained	Not Applicable	
47	NOCAM	Epinephelus flavolimbatus	Yellowedge Grouper	N	Brought on Board (Retained/Discarded)	Live - Stomach and/or Eyes Protruding	Retained	Not Applicable	
47	NOCAM	Epinephelus flavolimbatus	Yellowedge Grouper	N	Brought on Board (Retained/Discarded)	Live - Stomach and/or Eyes Protruding	Retained	Not Applicable	
47	NOCAM	Epinephelus flavolimbatus	Yellowedge Grouper	N	Brought on Board (Retained/Discarded)	Live - Healthy	Retained	Not Applicable	
47	NOCAM	Epinephelus flavolimbatus	Yellowedge Grouper	N	Brought on Board (Retained/Discarded)	Live - Stomach and/or Eyes Protruding	Retained	Not Applicable	
47	NOCAM	Epinephelus flavolimbatus	Yellowedge Grouper	N	Brought on Board (Retained/Discarded)	Live - Stomach and/or Eyes Protruding	Retained	Not Applicable	
47	NOCAM	Gadiformes, unidentified	Hake, Unidentified	N	Brought on Board (Retained/Discarded)	Live - Stomach and/or Eyes Protruding	Retained as Bait	Not Applicable	
47	NOCAM	Muraenidae, unidentified	Moray Eel, Unidentified	N	Brought on Board (Retained/Discarded)	Live - Healthy	Retained as Bait	Not Applicable	
47	NOCAM	Muraenidae, unidentified	Moray Eel, Unidentified	N	Brought on Board (Retained/Discarded)	Live - Healthy	Retained as Bait	Not Applicable	
47	NOCAM	Muraenidae, unidentified	Moray Eel, Unidentified	N	Brought on Board (Retained/Discarded)	Live - Healthy	Retained as Bait	Not Applicable	
47	NOCAM	Muraenidae, unidentified	Moray Eel, Unidentified	N	Brought on Board (Retained/Discarded)	Live - Healthy	Retained as Bait	Not Applicable	
47	NOCAM	Muraenidae, unidentified	Moray Eel, Unidentified	N	Brought on Board (Retained/Discarded)	Live - Healthy	Retained as Bait	Not Applicable	
47	NOCAM	Muraenidae, unidentified	Moray Eel, Unidentified	N	Brought on Board (Retained/Discarded)	Live - Healthy	Retained as Bait	Not Applicable	
47	NOCAM	Muraenidae, unidentified	Moray Eel, Unidentified	N	Brought on Board (Retained/Discarded)	Live - Healthy	Retained as Bait	Not Applicable	
47	NOCAM	Muraenidae, unidentified	Moray Eel, Unidentified	N	Brought on Board (Retained/Discarded)	Live - Healthy	Retained as Bait	Not Applicable	
47	NOCAM	Pagrus pagrus	Red Porgy	N	Brought on Board (Retained/Discarded)	Live - Healthy	Retained	Not Applicable	
47	UCAM	Caulolatilus microps	Blueline Tilefish	N	Brought on Board (Retained/Discarded)	Live - Stomach and/or Eyes Protruding	Retained	Not Applicable	
47	UCAM	Caulolatilus microps	Blueline Tilefish	N	Brought on Board (Retained/Discarded)	Live - Healthy	Retained	Not Applicable	
47	UCAM	Caulolatilus microps	Blueline Tilefish	N	Brought on Board (Retained/Discarded)	Live - Healthy	Retained	Not Applicable	
47	UCAM	Caulolatilus microps	Blueline Tilefish	N	Brought on Board (Retained/Discarded)	Live - Healthy	Retained	Not Applicable	
47	UCAM	Caulolatilus microps	Blueline Tilefish	N	Brought on Board (Retained/Discarded)	Live - Healthy	Retained	Not Applicable	
47	UCAM	Caulolatilus microps	Blueline Tilefish	N	Brought on Board (Retained/Discarded)	Live - Stomach and/or Eyes Protruding	Retained	Not Applicable	
47	UCAM	Crab, unidentified	Crab, Unidentified	N	Brought on Board (Retained/Discarded)	Live - Healthy	Discarded - Live and Healthy (Not Vented)	Not Applicable	
47	UCAM	Epinephelus flavolimbatus	Yellowedge Grouper	N	Brought on Board (Retained/Discarded)	Live - Healthy	Retained	Not Applicable	
47	UCAM	Epinephelus flavolimbatus	Yellowedge Grouper	N	Brought on Board (Retained/Discarded)	Live - Stomach and/or Eyes Protruding	Retained	Not Applicable	
47	UCAM	Epinephelus flavolimbatus	Yellowedge Grouper	N	Brought on Board (Retained/Discarded)	Live - Stomach and/or Eyes Protruding	Retained	Not Applicable	
47	UCAM	Epinephelus flavolimbatus	Yellowedge Grouper	N	Brought on Board (Retained/Discarded)	Live - Healthy	Retained	Not Applicable	
47	UCAM	Epinephelus flavolimbatus	Yellowedge Grouper	N	Brought on Board (Retained/Discarded)	Live - Stomach and/or Eyes Protruding	Retained	Not Applicable	
47	UCAM	Epinephelus flavolimbatus	Yellowedge Grouper	N	Brought on Board (Retained/Discarded)	Live - Stomach and/or Eyes Protruding	Retained	Not Applicable	
47	UCAM	Epinephelus flavolimbatus	Yellowedge Grouper	N	Brought on Board (Retained/Discarded)	Live - Stomach and/or Eyes Protruding	Retained	Not Applicable	
47	UCAM	Epinephelus flavolimbatus	Yellowedge Grouper	N	Brought on Board (Retained/Discarded)	Live - Stomach and/or Eyes Protruding	Retained	Not Applicable	
47	UCAM	Epinephelus flavolimbatus	Yellowedge Grouper	N	Brought on Board (Retained/Discarded)	Live - Stomach and/or Eyes Protruding	Retained	Not Applicable	
47	UCAM	Epinephelus flavolimbatus	Yellowedge Grouper	N	Brought on Board (Retained/Discarded)	Live - Stomach and/or Eyes Protruding	Retained	Not Applicable	
47	UCAM	Epinephelus flavolimbatus	Yellowedge Grouper	N	Brought on Board (Retained/Discarded)	Live - Stomach and/or Eyes Protruding	Retained	Not Applicable	
47	UCAM	Epinephelus flavolimbatus	Yellowedge Grouper	N	Brought on Board (Retained/Discarded)	Live - Stomach and/or Eyes Protruding	Retained	Not Applicable	
47	UCAM	Epinephelus flavolimbatus	Yellowedge Grouper	N	Brought on Board (Retained/Discarded)	Live - Stomach and/or Eyes Protruding	Retained	Not Applicable	
47	UCAM	Epinephelus flavolimbatus	Yellowedge Grouper	N	Brought on Board (Retained/Discarded)	Live - Stomach and/or Eyes Protruding	Retained	Not Applicable	
47	UCAM	Epinephelus flavolimbatus	Yellowedge Grouper	N	Brought on Board (Retained/Discarded)	Live - Stomach and/or Eyes Protruding	Retained	Not Applicable	
47	UCAM	Gadiformes, unidentified	Hake, Unidentified	N	Brought on Board (Retained/Discarded)	Live - Healthy	Retained as Bait	Not Applicable	
47	UCAM	Muraenidae, unidentified	Moray Eel, Unidentified	N	Brought on Board (Retained/Discarded)	Live - Healthy	Retained as Bait	Not Applicable	
47	UCAM	Muraenidae, unidentified	Moray Eel, Unidentified	N	Brought on Board (Retained/Discarded)	Live - Healthy	Retained as Bait	Not Applicable	

47	UCAM	Muraenidae, unidentified	Moray Eel, Unidentified	N	Brought on Board (Retained/Discarded)	Live - Healthy	Retained as Bait	Not Applicable	
47	UCAM	Muraenidae, unidentified	Moray Eel, Unidentified	N	Brought on Board (Retained/Discarded)	Live - Healthy	Retained as Bait	Not Applicable	
47	UCAM	Muraenidae, unidentified	Moray Eel, Unidentified	N	Brought on Board (Retained/Discarded)	Live - Healthy	Retained as Bait	Not Applicable	
47	UCAM	Muraenidae, unidentified	Moray Eel, Unidentified	N	Brought on Board (Retained/Discarded)	Live - Healthy	Retained as Bait	Not Applicable	
47	UCAM	Pagrus pagrus	Red Porgy	N	Brought on Board (Retained/Discarded)	Live - Damaged	Retained	Not Applicable	
48	NOCAM	Anguilliformes, unidentified	Eel, Unidentified	N	Brought on Board (Retained/Discarded)	Live - Healthy	Retained as Bait	Not Applicable	
48	NOCAM	Caulolatilus microps	Blueline Tilefish	N	Brought on Board (Retained/Discarded)	Live - Healthy	Discarded - Live and Healthy (Not Vented)	Not Applicable	
48	NOCAM	Caulolatilus microps	Blueline Tilefish	N	Brought on Board (Retained/Discarded)	Live - Healthy	Retained	Not Applicable	
48	NOCAM	Caulolatilus microps	Blueline Tilefish	N	Brought on Board (Retained/Discarded)	Live - Healthy	Retained	Not Applicable	
48	NOCAM	Caulolatilus microps	Blueline Tilefish	N	Brought on Board (Retained/Discarded)	Live - Healthy	Retained	Not Applicable	
48	NOCAM	Epinephelus flavolimbatus	Yellowedge Grouper	N	Brought on Board (Retained/Discarded)	Live - Stomach and/or Eyes Protruding	Retained	Not Applicable	
48	NOCAM	Epinephelus flavolimbatus	Yellowedge Grouper	N	Brought on Board (Retained/Discarded)	Live - Stomach and/or Eyes Protruding	Retained	Not Applicable	
48	NOCAM	Epinephelus flavolimbatus	Yellowedge Grouper	N	Brought on Board (Retained/Discarded)	Live - Stomach and/or Eyes Protruding	Retained	Not Applicable	
48	NOCAM	Epinephelus flavolimbatus	Yellowedge Grouper	N	Brought on Board (Retained/Discarded)	Live - Stomach and/or Eyes Protruding	Retained	Not Applicable	
48	NOCAM	Epinephelus flavolimbatus	Yellowedge Grouper	N	Brought on Board (Retained/Discarded)	Live - Stomach and/or Eyes Protruding	Retained	Not Applicable	
48	NOCAM	Epinephelus niveatus	Snowy Grouper	N	Brought on Board (Retained/Discarded)	Live - Healthy	Retained	Not Applicable	
48	NOCAM	Muraenidae, unidentified	Moray Eel, Unidentified	N	Brought on Board (Retained/Discarded)	Live - Healthy	Retained as Bait	Not Applicable	
48	UCAM	Anguilliformes, unidentified	Eel, Unidentified	N	Brought on Board (Retained/Discarded)	Live - Healthy	Retained as Bait	Not Applicable	
48	UCAM	Caulolatilus microps	Blueline Tilefish	N	Brought on Board (Retained/Discarded)	Live - Healthy	Discarded - Live and Healthy (Not Vented)	Not Applicable	
48	UCAM	Caulolatilus microps	Blueline Tilefish	N	Brought on Board (Retained/Discarded)	Live - Healthy	Retained	Not Applicable	
48	UCAM	Caulolatilus microps	Blueline Tilefish	N	Brought on Board (Retained/Discarded)	Live - Healthy	Retained	Not Applicable	
48	UCAM	Caulolatilus microps	Blueline Tilefish	N	Brought on Board (Retained/Discarded)	Live - Healthy	Retained	Not Applicable	
48	UCAM	Crab, unidentified	Crab, Unidentified	N	Brought on Board (Retained/Discarded)	Live - Healthy	Discarded - Live and Healthy (Not Vented)	Not Applicable	
48	UCAM	Epinephelus flavolimbatus	Yellowedge Grouper	N	Brought on Board (Retained/Discarded)	Live - Stomach and/or Eyes Protruding	Retained	Not Applicable	
48	UCAM	Epinephelus flavolimbatus	Yellowedge Grouper	N	Brought on Board (Retained/Discarded)	Live - Stomach and/or Eyes Protruding	Retained	Not Applicable	
48	UCAM	Epinephelus flavolimbatus	Yellowedge Grouper	N	Brought on Board (Retained/Discarded)	Live - Stomach and/or Eyes Protruding	Retained	Not Applicable	
48	UCAM	Epinephelus flavolimbatus	Yellowedge Grouper	N	Brought on Board (Retained/Discarded)	Live - Stomach and/or Eyes Protruding	Retained	Not Applicable	
48	UCAM	Epinephelus flavolimbatus	Yellowedge Grouper	N	Brought on Board (Retained/Discarded)	Live - Stomach and/or Eyes Protruding	Retained	Not Applicable	
48	UCAM	Epinephelus niveatus	Snowy Grouper	N	Brought on Board (Retained/Discarded)	Live - Healthy	Retained	Not Applicable	
48	UCAM	Muraenidae, unidentified	Moray Eel, Unidentified	N	Brought on Board (Retained/Discarded)	Live - Healthy	Retained as Bait	Not Applicable	
49	NOCAM	Carcharhinus acronotus	Blacknose Shark	Y	Brought on Board (Retained/Discarded)	Live - Healthy	Discarded - Live and Healthy (Not Vented)	Female	Medium (Between 1m and 2m)
49	NOCAM	Epinephelus morio	Red Grouper	N	Brought on Board (Retained/Discarded)	Live - Healthy	Discarded - Live and Healthy (Vented)	Not Applicable	
49	NOCAM	Epinephelus morio	Red Grouper	N	Brought on Board (Retained/Discarded)	Live - Healthy	Discarded - Live and Healthy (Vented)	Not Applicable	
49	NOCAM	Epinephelus morio	Red Grouper	N	Brought on Board (Retained/Discarded)	Live - Healthy	Retained	Not Applicable	
49	NOCAM	Epinephelus morio	Red Grouper	N	Brought on Board (Retained/Discarded)	Live - Healthy	Retained	Not Applicable	
49	NOCAM	Epinephelus morio	Red Grouper	N	Brought on Board (Retained/Discarded)	Live - Healthy	Retained	Not Applicable	
49	NOCAM	Epinephelus morio	Red Grouper	N	Brought on Board (Retained/Discarded)	Live - Stomach and/or Eyes Protruding	Retained	Not Applicable	
49	NOCAM	Epinephelus morio	Red Grouper	N	Brought on Board (Retained/Discarded)	Live - Healthy	Retained	Not Applicable	
49	NOCAM	Epinephelus morio	Red Grouper	N	Brought on Board (Retained/Discarded)	Live - Healthy	Retained	Not Applicable	
49	NOCAM	Epinephelus morio	Red Grouper	N	Brought on Board (Retained/Discarded)	Live - Healthy	Discarded - Live and Healthy (Vented)	Not Applicable	
49	NOCAM	Epinephelus morio	Red Grouper	N	Brought on Board (Retained/Discarded)	Live - Stomach and/or Eyes Protruding	Discarded - Live and Healthy (Vented)	Not Applicable	
49	NOCAM	Epinephelus morio	Red Grouper	N	Brought on Board (Retained/Discarded)	Live - Healthy	Retained	Not Applicable	
49	NOCAM	Epinephelus morio	Red Grouper	N	Brought on Board (Retained/Discarded)	Live - Healthy	Retained	Not Applicable	
49	NOCAM	Epinephelus morio	Red Grouper	N	Brought on Board (Retained/Discarded)	Live - Healthy	Discarded - Live and Healthy (Vented)	Not Applicable	
49	NOCAM	Epinephelus morio	Red Grouper	N	Brought on Board (Retained/Discarded)	Live - Healthy	Retained	Not Applicable	
49	NOCAM	Epinephelus morio	Red Grouper	N	Brought on Board (Retained/Discarded)	Live - Stomach and/or Eyes Protruding	Discarded - Live and Healthy (Vented)	Not Applicable	
49	NOCAM	Epinephelus morio	Red Grouper	N	Brought on Board (Retained/Discarded)	Live - Stomach and/or Eyes Protruding	Discarded - Live and Healthy (Vented)	Not Applicable	
49	NOCAM	Epinephelus morio	Red Grouper	N	Brought on Board (Retained/Discarded)	Live - Healthy	Discarded - Live and Healthy (Vented)	Not Applicable	
49	NOCAM	Epinephelus morio	Red Grouper	N	Brought on Board (Retained/Discarded)	Live - Healthy	Discarded - Live and Healthy (Vented)	Not Applicable	
49	NOCAM	Epinephelus morio	Red Grouper	N	Brought on Board (Retained/Discarded)	Live - Healthy	Retained	Not Applicable	
49	NOCAM	Epinephelus morio	Red Grouper	N	Brought on Board (Retained/Discarded)	Live - Healthy	Retained	Not Applicable	
49	NOCAM	Epinephelus morio	Red Grouper	N	Brought on Board (Retained/Discarded)	Live - Stomach and/or Eyes Protruding	Discarded - Live and Healthy (Vented)	Not Applicable	
49	NOCAM	Epinephelus morio	Red Grouper	N	Brought on Board (Retained/Discarded)	Live - Healthy	Retained	Not Applicable	
49	NOCAM	Epinephelus morio	Red Grouper	N	Brought on Board (Retained/Discarded)	Live - Stomach and/or Eyes Protruding	Discarded - Live and Healthy (Vented)	Not Applicable	
49	NOCAM	Epinephelus morio	Red Grouper	N	Brought on Board (Retained/Discarded)	Live - Healthy	Discarded - Live and Healthy (Vented)	Not Applicable	
49	NOCAM	Epinephelus morio	Red Grouper	N	Brought on Board (Retained/Discarded)	Live - Healthy	Retained	Not Applicable	
49	NOCAM	Epinephelus morio	Red Grouper	N	Brought on Board (Retained/Discarded)	Live - Healthy	Retained	Not Applicable	
49	NOCAM	Epinephelus morio	Red Grouper	N	Brought on Board (Retained/Discarded)	Live - Healthy	Retained	Not Applicable	
49	NOCAM	Epinephelus morio	Red Grouper	N	Brought on Board (Retained/Discarded)	Live - Stomach and/or Eyes Protruding	Retained	Not Applicable	
49	NOCAM	Epinephelus morio	Red Grouper	N	Brought on Board (Retained/Discarded)	Live - Healthy	Discarded - Live and Healthy (Vented)	Not Applicable	
49	NOCAM	Epinephelus morio	Red Grouper	N	Brought on Board (Retained/Discarded)	Live - Healthy	Discarded - Live and Healthy (Not Vented)	Not Applicable	
49	NOCAM	Epinephelus morio	Red Grouper	N	Brought on Board (Retained/Discarded)	Live - Stomach and/or Eyes Protruding	Retained	Not Applicable	
49	NOCAM	Epinephelus morio	Red Grouper	N	Brought on Board (Retained/Discarded)	Live - Healthy	Retained	Not Applicable	
49	NOCAM	Epinephelus morio	Red Grouper	N	Brought on Board (Retained/Discarded)	Live - Stomach and/or Eyes Protruding	Retained	Not Applicable	
49	NOCAM	Epinephelus morio	Red Grouper	N	Brought on Board (Retained/Discarded)	Live - Healthy	Retained	Not Applicable	
49	NOCAM	Epinephelus morio	Red Grouper	N	Brought on Board (Retained/Discarded)	Dead on Arrival - Damaged	Discarded - Dead	Not Applicable	
49	NOCAM	Epinephelus morio	Red Grouper	N	Brought on Board (Retained/Discarded)	Live - Healthy	Retained	Not Applicable	
49	NOCAM	Epinephelus morio	Red Grouper	N	Brought on Board (Retained/Discarded)	Live - Stomach and/or Eyes Protruding	Retained	Not Applicable	
49	NOCAM	Epinephelus morio	Red Grouper	N	Brought on Board (Retained/Discarded)	Live - Healthy	Retained	Not Applicable	
49	NOCAM	Epinephelus morio	Red Grouper	N	Brought on Board (Retained/Discarded)	Live - Stomach and/or Eyes Protruding	Retained	Not Applicable	
49	NOCAM	Epinephelus morio	Red Grouper	N	Brought on Board (Retained/Discarded)	Live - Healthy	Retained	Not Applicable	
49	NOCAM	Epinephelus morio	Red Grouper	N	Brought on Board (Retained/Discarded)	Live - Healthy	Retained	Not Applicable	
49	NOCAM	Epinephelus morio	Red Grouper	N	Brought on Board (Retained/Discarded)	Live - Stomach and/or Eyes Protruding	Retained	Not Applicable	
49	NOCAM	Epinephelus morio	Red Grouper	N	Brought on Board (Retained/Discarded)	Live - Stomach and/or Eyes Protruding	Discarded - Live and Healthy (Vented)	Not Applicable	
49	NOCAM	Epinephelus morio	Red Grouper	N	Brought on Board (Retained/Discarded)	Live - Healthy	Retained	Not Applicable	
49	NOCAM	Ginglymostoma cirratum	Nurse Shark	Y	Cut-off at Rail (No Entanglement)	Live - Healthy	Discarded - Live and Healthy (Not Vented)	Unknown Maturity and/or Sex	Medium (Between 1m and 2m)
49	NOCAM	Lutjanus campechanus	Red Snapper	N	Brought on Board (Retained/Discarded)	Live - Healthy	Discarded - Live and Healthy (Vented)	Not Applicable	
49	NOCAM	Lutjanus campechanus	Red Snapper	N	Brought on Board (Retained/Discarded)	Live - Healthy	Discarded - Live and Healthy (Vented)	Not Applicable	
49	NOCAM	Lutjanus campechanus	Red Snapper	N	Brought on Board (Retained/Discarded)	Live - Healthy	Discarded - Live and Healthy (Vented)	Not Applicable	
49	NOCAM	Lutjanus campechanus	Red Snapper	N	Brought on Board (Retained/Discarded)	Live - Healthy	Discarded - Live and Healthy (Vented)	Not Applicable	
49	NOCAM	Lutjanus campechanus	Red Snapper	N	Brought on Board (Retained/Discarded)	Live - Healthy	Discarded - Live and Healthy (Vented)	Not Applicable	
49	NOCAM	Lutjanus campechanus	Red Snapper	N	Brought on Board (Retained/Discarded)	Live - Healthy	Discarded - Live and Healthy (Vented)	Not Applicable	
49	NOCAM	Lutjanus campechanus	Red Snapper	N	Brought on Board (Retained/Discarded)	Live - Healthy	Discarded - Live and Healthy (Vented)	Not Applicable	
49	NOCAM	Lutjanus campechanus	Red Snapper	N	Brought on Board (Retained/Discarded)	Live - Healthy	Discarded - Live and Healthy (Vented)	Not Applicable	
49	NOCAM	Lutjanus campechanus	Red Snapper	N	Brought on Board (Retained/Discarded)	Live - Healthy	Discarded - Live and Healthy (Vented)	Not Applicable	
49	NOCAM	Lutjanus campechanus	Red Snapper	N	Brought on Board (Retained/Discarded)	Live - Stomach and/or Eyes Protruding	Discarded - Live and Healthy (Vented)	Not Applicable	
49	NOCAM	Lutjanus campechanus	Red Snapper	N	Brought on Board (Retained/Discarded)	Live - Healthy	Discarded - Live and Healthy (Vented)	Not Applicable	
49	NOCAM	Lutjanus griseus	Gray (Mangrove) Snapper	N	Brought on Board (Retained/Discarded)	Live - Stomach and/or Eyes Protruding	Retained	Not Applicable	
49	NOCAM	Sphyraena barracuda	Barracuda (Great)	N	Brought on Board (Retained/Discarded)	Live - Healthy	Discarded - Live and Healthy (Not Vented)	Not Applicable	
49	UCAM	Carcharhinus acronotus	Blacknose Shark	Y	Brought on Board (Retained/Discarded)	Live - Healthy	Discarded - Live and Healthy (Not Vented)	Female	Medium (Between 1m and 2m)
49	UCAM	Carcharhinus plumbeus	Sandbar Shark	Y	Cut-off at Rail (No Entanglement)	Live - Healthy	Discarded - Live and Healthy (Not Vented)	Female	Large (Greater than 2m)
49	UCAM	Epinephelus morio	Red Grouper	N	Brought on Board (Retained/Discarded)	Live - Healthy	Retained	Not Applicable	

49	UCAM	Epinephelus morio	Red Grouper	N	Brought on Board (Retained/Discarded)	Live - Healthy	Retained	Not Applicable	
49	UCAM	Epinephelus morio	Red Grouper	N	Brought on Board (Retained/Discarded)	Dead on Arrival - Damaged	Discarded - Dead	Not Applicable	
49	UCAM	Epinephelus morio	Red Grouper	N	Brought on Board (Retained/Discarded)	Live - Healthy	Discarded - Live and Healthy (Vented)	Not Applicable	
49	UCAM	Epinephelus morio	Red Grouper	N	Brought on Board (Retained/Discarded)	Live - Healthy	Discarded - Live and Healthy (Vented)	Not Applicable	
49	UCAM	Epinephelus morio	Red Grouper	N	Brought on Board (Retained/Discarded)	Live - Healthy	Discarded - Live and Healthy (Vented)	Not Applicable	
49	UCAM	Epinephelus morio	Red Grouper	N	Brought on Board (Retained/Discarded)	Live - Healthy	Retained	Not Applicable	
49	UCAM	Epinephelus morio	Red Grouper	N	Brought on Board (Retained/Discarded)	Live - Healthy	Retained	Not Applicable	
49	UCAM	Epinephelus morio	Red Grouper	N	Brought on Board (Retained/Discarded)	Live - Healthy	Retained	Not Applicable	
49	UCAM	Epinephelus morio	Red Grouper	N	Brought on Board (Retained/Discarded)	Live - Stomach and/or Eyes Protruding	Retained	Not Applicable	
49	UCAM	Epinephelus morio	Red Grouper	N	Brought on Board (Retained/Discarded)	Live - Healthy	Discarded - Live and Healthy (Vented)	Not Applicable	
49	UCAM	Epinephelus morio	Red Grouper	N	Brought on Board (Retained/Discarded)	Live - Healthy	Discarded - Live and Healthy (Vented)	Not Applicable	
49	UCAM	Epinephelus morio	Red Grouper	N	Brought on Board (Retained/Discarded)	Live - Stomach and/or Eyes Protruding	Retained	Not Applicable	
49	UCAM	Epinephelus morio	Red Grouper	N	Brought on Board (Retained/Discarded)	Live - Healthy	Retained	Not Applicable	
49	UCAM	Epinephelus morio	Red Grouper	N	Brought on Board (Retained/Discarded)	Live - Healthy	Retained	Not Applicable	
49	UCAM	Epinephelus morio	Red Grouper	N	Brought on Board (Retained/Discarded)	Live - Healthy	Retained	Not Applicable	
49	UCAM	Epinephelus morio	Red Grouper	N	Brought on Board (Retained/Discarded)	Live - Stomach and/or Eyes Protruding	Discarded - Live and Healthy (Vented)	Not Applicable	
49	UCAM	Epinephelus morio	Red Grouper	N	Brought on Board (Retained/Discarded)	Live - Stomach and/or Eyes Protruding	Retained	Not Applicable	
49	UCAM	Epinephelus morio	Red Grouper	N	Brought on Board (Retained/Discarded)	Live - Healthy	Retained	Not Applicable	
49	UCAM	Epinephelus morio	Red Grouper	N	Brought on Board (Retained/Discarded)	Live - Healthy	Discarded - Live and Healthy (Vented)	Not Applicable	
49	UCAM	Epinephelus morio	Red Grouper	N	Brought on Board (Retained/Discarded)	Live - Healthy	Retained	Not Applicable	
49	UCAM	Epinephelus morio	Red Grouper	N	Brought on Board (Retained/Discarded)	Live - Stomach and/or Eyes Protruding	Retained	Not Applicable	
49	UCAM	Epinephelus morio	Red Grouper	N	Brought on Board (Retained/Discarded)	Live - Healthy	Retained	Not Applicable	
49	UCAM	Epinephelus morio	Red Grouper	N	Brought on Board (Retained/Discarded)	Live - Healthy	Retained	Not Applicable	
49	UCAM	Epinephelus morio	Red Grouper	N	Brought on Board (Retained/Discarded)	Live - Stomach and/or Eyes Protruding	Discarded - Live and Healthy (Vented)	Not Applicable	
49	UCAM	Epinephelus morio	Red Grouper	N	Brought on Board (Retained/Discarded)	Live - Stomach and/or Eyes Protruding	Retained	Not Applicable	
49	UCAM	Epinephelus morio	Red Grouper	N	Brought on Board (Retained/Discarded)	Live - Healthy	Discarded - Live and Healthy (Vented)	Not Applicable	
49	UCAM	Epinephelus morio	Red Grouper	N	Brought on Board (Retained/Discarded)	Live - Healthy	Retained	Not Applicable	
49	UCAM	Epinephelus morio	Red Grouper	N	Brought on Board (Retained/Discarded)	Live - Stomach and/or Eyes Protruding	Retained	Not Applicable	
49	UCAM	Epinephelus morio	Red Grouper	N	Brought on Board (Retained/Discarded)	Live - Healthy	Retained	Not Applicable	
49	UCAM	Epinephelus morio	Red Grouper	N	Brought on Board (Retained/Discarded)	Live - Healthy	Retained	Not Applicable	
49	UCAM	Epinephelus morio	Red Grouper	N	Brought on Board (Retained/Discarded)	Live - Healthy	Retained	Not Applicable	
49	UCAM	Epinephelus morio	Red Grouper	N	Brought on Board (Retained/Discarded)	Live - Stomach and/or Eyes Protruding	Retained	Not Applicable	
49	UCAM	Epinephelus morio	Red Grouper	N	Brought on Board (Retained/Discarded)	Live - Healthy	Retained	Not Applicable	
49	UCAM	Epinephelus morio	Red Grouper	N	Brought on Board (Retained/Discarded)	Live - Healthy	Retained	Not Applicable	
49	UCAM	Epinephelus morio	Red Grouper	N	Brought on Board (Retained/Discarded)	Live - Healthy	Discarded - Live and Healthy (Not Vented)	Not Applicable	
49	UCAM	Epinephelus morio	Red Grouper	N	Brought on Board (Retained/Discarded)	Live - Healthy	Retained	Not Applicable	
49	UCAM	Epinephelus morio	Red Grouper	N	Brought on Board (Retained/Discarded)	Live - Healthy	Retained	Not Applicable	
49	UCAM	Epinephelus morio	Red Grouper	N	Brought on Board (Retained/Discarded)	Live - Stomach and/or Eyes Protruding	Retained	Not Applicable	
49	UCAM	Epinephelus morio	Red Grouper	N	Brought on Board (Retained/Discarded)	Live - Stomach and/or Eyes Protruding	Discarded - Live and Healthy (Vented)	Not Applicable	
49	UCAM	Epinephelus morio	Red Grouper	N	Brought on Board (Retained/Discarded)	Live - Stomach and/or Eyes Protruding	Retained	Not Applicable	
49	UCAM	Epinephelus morio	Red Grouper	N	Brought on Board (Retained/Discarded)	Live - Healthy	Retained	Not Applicable	
49	UCAM	Epinephelus morio	Red Grouper	N	Brought on Board (Retained/Discarded)	Live - Stomach and/or Eyes Protruding	Discarded - Live and Healthy (Vented)	Not Applicable	
49	UCAM	Epinephelus morio	Red Grouper	N	Brought on Board (Retained/Discarded)	Live - Healthy	Discarded - Live and Healthy (Vented)	Not Applicable	
49	UCAM	Epinephelus morio	Red Grouper	N	Brought on Board (Retained/Discarded)	Live - Healthy	Discarded - Live and Healthy (Vented)	Not Applicable	
49	UCAM	Ginglymostoma cirratum	Nurse Shark	Y	Cut-off at Rail (No Entanglement)	Live - Healthy	Discarded - Live and Healthy (Not Vented)	Unknown Maturity and/or Sex	Medium (Between 1m and 2m)
49	UCAM	Lutjanus campechanus	Red Snapper	N	Brought on Board (Retained/Discarded)	Live - Healthy	Discarded - Live and Healthy (Vented)	Not Applicable	
49	UCAM	Lutjanus campechanus	Red Snapper	N	Brought on Board (Retained/Discarded)	Live - Healthy	Discarded - Live and Healthy (Vented)	Not Applicable	
49	UCAM	Lutjanus campechanus	Red Snapper	N	Brought on Board (Retained/Discarded)	Live - Healthy	Discarded - Live and Healthy (Vented)	Not Applicable	
49	UCAM	Lutjanus campechanus	Red Snapper	N	Brought on Board (Retained/Discarded)	Live - Stomach and/or Eyes Protruding	Discarded - Live and Healthy (Vented)	Not Applicable	
49	UCAM	Lutjanus campechanus	Red Snapper	N	Brought on Board (Retained/Discarded)	Live - Healthy	Discarded - Live and Healthy (Vented)	Not Applicable	
49	UCAM	Lutjanus campechanus	Red Snapper	N	Brought on Board (Retained/Discarded)	Live - Healthy	Discarded - Live and Healthy (Vented)	Not Applicable	
49	UCAM	Lutjanus campechanus	Red Snapper	N	Brought on Board (Retained/Discarded)	Live - Healthy	Discarded - Live and Healthy (Vented)	Not Applicable	
49	UCAM	Lutjanus campechanus	Red Snapper	N	Brought on Board (Retained/Discarded)	Live - Healthy	Discarded - Live and Healthy (Vented)	Not Applicable	
49	UCAM	Lutjanus campechanus	Red Snapper	N	Brought on Board (Retained/Discarded)	Live - Healthy	Discarded - Live and Healthy (Vented)	Not Applicable	
49	UCAM	Lutjanus campechanus	Red Snapper	N	Brought on Board (Retained/Discarded)	Live - Stomach and/or Eyes Protruding	Discarded - Live and Healthy (Vented)	Not Applicable	
49	UCAM	Lutjanus campechanus	Red Snapper	N	Brought on Board (Retained/Discarded)	Live - Healthy	Discarded - Live and Healthy (Vented)	Not Applicable	
49	UCAM	Lutjanus campechanus	Red Snapper	N	Brought on Board (Retained/Discarded)	Live - Healthy	Discarded - Live and Healthy (Vented)	Not Applicable	
49	UCAM	Lutjanus griseus	Gray (Mangrove) Snapper	N	Brought on Board (Retained/Discarded)	Live - Stomach and/or Eyes Protruding	Retained	Not Applicable	
49	UCAM	Sphyraena barracuda	Barracuda (Great)	N	Brought on Board (Retained/Discarded)	Live - Healthy	Discarded - Live and Healthy (Not Vented)	Not Applicable	
50	NOCAM	Caulolatilus microps	Blueline Tilefish	N	Brought on Board (Retained/Discarded)	Live - Stomach and/or Eyes Protruding	Retained	Not Applicable	
50	NOCAM	Caulolatilus microps	Blueline Tilefish	N	Brought on Board (Retained/Discarded)	Live - Stomach and/or Eyes Protruding	Retained	Not Applicable	
50	NOCAM	Caulolatilus microps	Blueline Tilefish	N	Brought on Board (Retained/Discarded)	Dead on Arrival - Damaged	Discarded - Dead	Not Applicable	
50	NOCAM	Epinephelus flavolimbatus	Yellowedge Grouper	N	Brought on Board (Retained/Discarded)	Live - Stomach and/or Eyes Protruding	Retained	Not Applicable	
50	NOCAM	Epinephelus flavolimbatus	Yellowedge Grouper	N	Brought on Board (Retained/Discarded)	Live - Stomach and/or Eyes Protruding	Retained	Not Applicable	
50	NOCAM	Epinephelus flavolimbatus	Yellowedge Grouper	N	Brought on Board (Retained/Discarded)	Live - Stomach and/or Eyes Protruding	Retained	Not Applicable	
50	NOCAM	Epinephelus flavolimbatus	Yellowedge Grouper	N	Brought on Board (Retained/Discarded)	Live - Stomach and/or Eyes Protruding	Retained	Not Applicable	
50	NOCAM	Epinephelus flavolimbatus	Yellowedge Grouper	N	Brought on Board (Retained/Discarded)	Live - Stomach and/or Eyes Protruding	Retained	Not Applicable	
50	NOCAM	Epinephelus flavolimbatus	Yellowedge Grouper	N	Brought on Board (Retained/Discarded)	Live - Stomach and/or Eyes Protruding	Retained	Not Applicable	
50	NOCAM	Epinephelus flavolimbatus	Yellowedge Grouper	N	Brought on Board (Retained/Discarded)	Live - Stomach and/or Eyes Protruding	Retained	Not Applicable	
50	NOCAM	Epinephelus flavolimbatus	Yellowedge Grouper	N	Brought on Board (Retained/Discarded)	Live - Stomach and/or Eyes Protruding	Retained	Not Applicable	
50	NOCAM	Epinephelus flavolimbatus	Yellowedge Grouper	N	Brought on Board (Retained/Discarded)	Live - Stomach and/or Eyes Protruding	Retained	Not Applicable	
50	NOCAM	Epinephelus flavolimbatus	Yellowedge Grouper	N	Brought on Board (Retained/Discarded)	Live - Stomach and/or Eyes Protruding	Retained	Not Applicable	
50	NOCAM	Epinephelus flavolimbatus	Yellowedge Grouper	N	Brought on Board (Retained/Discarded)	Live - Stomach and/or Eyes Protruding	Retained	Not Applicable	
50	NOCAM	Lopholatilus chamaelonticeps	Tilefish, Golden	N	Brought on Board (Retained/Discarded)	Dead on Arrival - Damaged	Discarded - Dead	Not Applicable	
50	NOCAM	Squalidae spp.	Dogfish, Spiny (Cuban)	Y	Brought on Board (Retained/Discarded)	Live - Healthy	Discarded - Live and Healthy (Not Vented)	Unknown Maturity and/or Sex	Small (Less than 1m)
50	UCAM	Caulolatilus microps	Blueline Tilefish	N	Brought on Board (Retained/Discarded)	Live - Healthy	Retained	Not Applicable	
50	UCAM	Caulolatilus microps	Blueline Tilefish	N	Brought on Board (Retained/Discarded)	Live - Stomach and/or Eyes Protruding	Retained	Not Applicable	
50	UCAM	Caulolatilus microps	Blueline Tilefish	N	Brought on Board (Retained/Discarded)	Dead on Arrival - Damaged	Discarded - Dead	Not Applicable	
50	UCAM	Epinephelus flavolimbatus	Yellowedge Grouper	N	Brought on Board (Retained/Discarded)	Live - Stomach and/or Eyes Protruding	Retained	Not Applicable	
50	UCAM	Epinephelus flavolimbatus	Yellowedge Grouper	N	Brought on Board (Retained/Discarded)	Live - Stomach and/or Eyes Protruding	Retained	Not Applicable	
50	UCAM	Epinephelus flavolimbatus	Yellowedge Grouper	N	Brought on Board (Retained/Discarded)	Live - Stomach and/or Eyes Protruding	Retained	Not Applicable	
50	UCAM	Epinephelus flavolimbatus	Yellowedge Grouper	N	Brought on Board (Retained/Discarded)	Live - Stomach and/or Eyes Protruding	Retained	Not Applicable	
50	UCAM	Epinephelus flavolimbatus	Yellowedge Grouper	N	Brought on Board (Retained/Discarded)	Live - Stomach and/or Eyes Protruding	Retained	Not Applicable	
50	UCAM	Epinephelus flavolimbatus	Yellowedge Grouper	N	Brought on Board (Retained/Discarded)	Live - Stomach and/or Eyes Protruding	Retained	Not Applicable	
50	UCAM	Epinephelus flavolimbatus	Yellowedge Grouper	N	Brought on Board (Retained/Discarded)	Dead on Arrival - Damaged	Discarded - Dead	Not Applicable	
50	UCAM	Epinephelus flavolimbatus	Yellowedge Grouper	N	Brought on Board (Retained/Discarded)	Live - Stomach and/or Eyes Protruding	Retained	Not Applicable	
50	UCAM	Epinephelus flavolimbatus	Yellowedge Grouper	N	Brought on Board (Retained/Discarded)	Live - Stomach and/or Eyes Protruding	Retained	Not Applicable	
50	UCAM	Epinephelus flavolimbatus	Yellowedge Grouper	N	Brought on Board (Retained/Discarded)	Live - Stomach and/or Eyes Protruding	Retained	Not Applicable	
50	UCAM	Epinephelus flavolimbatus	Yellowedge Grouper	N	Brought on Board (Retained/Discarded)	Live - Stomach and/or Eyes Protruding	Retained	Not Applicable	
50	UCAM	Epinephelus flavolimbatus	Yellowedge Grouper	N	Brought on Board (Retained/Discarded)	Live - Stomach and/or Eyes Protruding	Retained	Not Applicable	
50	UCAM	Lopholatilus chamaelonticeps	Tilefish, Golden	N	Brought on Board (Retained/Discarded)	Dead on Arrival - Damaged	Discarded - Dead	Not Applicable	
50	UCAM	Squalidae spp.	Dogfish, Spiny (Cuban)	Y	Brought on Board (Retained/Discarded)	Live - Healthy	Discarded - Live and Healthy (Not Vented)	Unknown Maturity and/or Sex	Small (Less than 1m)

51	NOCAM	Caulolatilus microps	Blueline Tilefish	N	Brought on Board (Retained/Discarded)	Live - Healthy	Retained	Not Applicable	
51	NOCAM	Caulolatilus microps	Blueline Tilefish	N	Brought on Board (Retained/Discarded)	Live - Healthy	Retained	Not Applicable	
51	NOCAM	Caulolatilus microps	Blueline Tilefish	N	Brought on Board (Retained/Discarded)	Live - Healthy	Retained	Not Applicable	
51	NOCAM	Epinephelus flavolimbatus	Yellowedge Grouper	N	Brought on Board (Retained/Discarded)	Live - Stomach and/or Eyes Protruding	Retained	Not Applicable	
51	NOCAM	Epinephelus flavolimbatus	Yellowedge Grouper	N	Brought on Board (Retained/Discarded)	Live - Healthy	Retained	Not Applicable	
51	NOCAM	Epinephelus flavolimbatus	Yellowedge Grouper	N	Brought on Board (Retained/Discarded)	Live - Healthy	Retained	Not Applicable	
51	NOCAM	Epinephelus flavolimbatus	Yellowedge Grouper	N	Brought on Board (Retained/Discarded)	Live - Stomach and/or Eyes Protruding	Retained	Not Applicable	
51	NOCAM	Epinephelus flavolimbatus	Yellowedge Grouper	N	Brought on Board (Retained/Discarded)	Live - Stomach and/or Eyes Protruding	Retained	Not Applicable	
51	NOCAM	Epinephelus flavolimbatus	Yellowedge Grouper	N	Brought on Board (Retained/Discarded)	Live - Stomach and/or Eyes Protruding	Retained	Not Applicable	
51	NOCAM	Epinephelus flavolimbatus	Yellowedge Grouper	N	Brought on Board (Retained/Discarded)	Live - Healthy	Retained	Not Applicable	
51	NOCAM	Epinephelus flavolimbatus	Yellowedge Grouper	N	Brought on Board (Retained/Discarded)	Live - Healthy	Retained	Not Applicable	
51	NOCAM	Epinephelus flavolimbatus	Yellowedge Grouper	N	Brought on Board (Retained/Discarded)	Live - Stomach and/or Eyes Protruding	Retained	Not Applicable	
51	NOCAM	Epinephelus flavolimbatus	Yellowedge Grouper	N	Brought on Board (Retained/Discarded)	Live - Stomach and/or Eyes Protruding	Retained	Not Applicable	
51	NOCAM	Epinephelus flavolimbatus	Yellowedge Grouper	N	Brought on Board (Retained/Discarded)	Live - Healthy	Retained	Not Applicable	
51	NOCAM	Epinephelus flavolimbatus	Yellowedge Grouper	N	Brought on Board (Retained/Discarded)	Live - Stomach and/or Eyes Protruding	Retained	Not Applicable	
51	NOCAM	Epinephelus flavolimbatus	Yellowedge Grouper	N	Brought on Board (Retained/Discarded)	Live - Stomach and/or Eyes Protruding	Retained	Not Applicable	
51	NOCAM	Epinephelus flavolimbatus	Yellowedge Grouper	N	Brought on Board (Retained/Discarded)	Live - Stomach and/or Eyes Protruding	Retained	Not Applicable	
51	NOCAM	Squalidae spp.	Dogfish, Spiny (Cuban)	Y	Brought on Board (Retained/Discarded)	Live - Healthy	Discarded - Live and Healthy (Not Vented)	Female	Small (Less than 1m)
51	NOCAM	Squalidae spp.	Dogfish, Spiny (Cuban)	Y	Brought on Board (Retained/Discarded)	Live - Healthy	Discarded - Live and Healthy (Not Vented)	Female	Small (Less than 1m)
51	NOCAM	Unidentified Bottom Debris	Unidentified Bottom Debris	N	Cut-off at Rail (No Entanglement)	Unknown Condition	Discarded - Unknown	Not Applicable	
51	UCAM	Caulolatilus microps	Blueline Tilefish	N	Brought on Board (Retained/Discarded)	Live - Healthy	Retained	Not Applicable	
51	UCAM	Caulolatilus microps	Blueline Tilefish	N	Brought on Board (Retained/Discarded)	Live - Healthy	Retained	Not Applicable	
51	UCAM	Caulolatilus microps	Blueline Tilefish	N	Brought on Board (Retained/Discarded)	Live - Healthy	Retained	Not Applicable	
51	UCAM	Epinephelus flavolimbatus	Yellowedge Grouper	N	Brought on Board (Retained/Discarded)	Live - Healthy	Retained	Not Applicable	
51	UCAM	Epinephelus flavolimbatus	Yellowedge Grouper	N	Brought on Board (Retained/Discarded)	Live - Stomach and/or Eyes Protruding	Retained	Not Applicable	
51	UCAM	Epinephelus flavolimbatus	Yellowedge Grouper	N	Brought on Board (Retained/Discarded)	Live - Stomach and/or Eyes Protruding	Retained	Not Applicable	
51	UCAM	Epinephelus flavolimbatus	Yellowedge Grouper	N	Brought on Board (Retained/Discarded)	Live - Stomach and/or Eyes Protruding	Retained	Not Applicable	
51	UCAM	Epinephelus flavolimbatus	Yellowedge Grouper	N	Brought on Board (Retained/Discarded)	Live - Stomach and/or Eyes Protruding	Retained	Not Applicable	
51	UCAM	Epinephelus flavolimbatus	Yellowedge Grouper	N	Brought on Board (Retained/Discarded)	Live - Stomach and/or Eyes Protruding	Retained	Not Applicable	
51	UCAM	Epinephelus flavolimbatus	Yellowedge Grouper	N	Brought on Board (Retained/Discarded)	Live - Healthy	Retained	Not Applicable	
51	UCAM	Epinephelus flavolimbatus	Yellowedge Grouper	N	Brought on Board (Retained/Discarded)	Live - Healthy	Retained	Not Applicable	
51	UCAM	Epinephelus flavolimbatus	Yellowedge Grouper	N	Brought on Board (Retained/Discarded)	Live - Stomach and/or Eyes Protruding	Retained	Not Applicable	
51	UCAM	Epinephelus flavolimbatus	Yellowedge Grouper	N	Brought on Board (Retained/Discarded)	Live - Stomach and/or Eyes Protruding	Retained	Not Applicable	
51	UCAM	Epinephelus flavolimbatus	Yellowedge Grouper	N	Brought on Board (Retained/Discarded)	Live - Stomach and/or Eyes Protruding	Retained	Not Applicable	
51	UCAM	Epinephelus flavolimbatus	Yellowedge Grouper	N	Brought on Board (Retained/Discarded)	Live - Healthy	Retained	Not Applicable	
51	UCAM	Epinephelus flavolimbatus	Yellowedge Grouper	N	Brought on Board (Retained/Discarded)	Live - Stomach and/or Eyes Protruding	Retained	Not Applicable	
51	UCAM	Epinephelus flavolimbatus	Yellowedge Grouper	N	Brought on Board (Retained/Discarded)	Live - Stomach and/or Eyes Protruding	Retained	Not Applicable	
51	UCAM	Squalidae spp.	Dogfish, Spiny (Cuban)	Y	Brought on Board (Retained/Discarded)	Live - Healthy	Discarded - Live and Healthy (Not Vented)	Female	Small (Less than 1m)
51	UCAM	Squalidae spp.	Dogfish, Spiny (Cuban)	Y	Brought on Board (Retained/Discarded)	Live - Healthy	Discarded - Live and Healthy (Not Vented)	Female	Small (Less than 1m)
52	NOCAM	Carcharhinus plumbeus	Sandbar Shark	Y	Cut-off at Rail (No Entanglement)	Live - Healthy	Discarded - Live and Healthy (Not Vented)	Known Adult - Undetermined Sex	Large (Greater than 2m)
52	NOCAM	Epinephelus morio	Red Grouper	N	Brought on Board (Retained/Discarded)	Live - Stomach and/or Eyes Protruding	Retained	Not Applicable	
52	NOCAM	Epinephelus morio	Red Grouper	N	Brought on Board (Retained/Discarded)	Live - Stomach and/or Eyes Protruding	Retained	Not Applicable	
52	NOCAM	Epinephelus morio	Red Grouper	N	Brought on Board (Retained/Discarded)	Live - Healthy	Retained	Not Applicable	
52	NOCAM	Epinephelus morio	Red Grouper	N	Brought on Board (Retained/Discarded)	Live - Healthy	Retained	Not Applicable	
52	NOCAM	Epinephelus morio	Red Grouper	N	Brought on Board (Retained/Discarded)	Live - Stomach and/or Eyes Protruding	Retained	Not Applicable	
52	NOCAM	Epinephelus morio	Red Grouper	N	Brought on Board (Retained/Discarded)	Live - Stomach and/or Eyes Protruding	Retained	Not Applicable	
52	NOCAM	Epinephelus morio	Red Grouper	N	Brought on Board (Retained/Discarded)	Live - Stomach and/or Eyes Protruding	Retained	Not Applicable	
52	NOCAM	Epinephelus morio	Red Grouper	N	Brought on Board (Retained/Discarded)	Live - Stomach and/or Eyes Protruding	Retained	Not Applicable	
52	NOCAM	Epinephelus morio	Red Grouper	N	Brought on Board (Retained/Discarded)	Live - Stomach and/or Eyes Protruding	Retained	Not Applicable	
52	NOCAM	Epinephelus morio	Red Grouper	N	Brought on Board (Retained/Discarded)	Live - Healthy	Discarded - Live and Healthy (Vented)	Not Applicable	
52	NOCAM	Epinephelus morio	Red Grouper	N	Brought on Board (Retained/Discarded)	Live - Healthy	Retained	Not Applicable	
52	NOCAM	Epinephelus morio	Red Grouper	N	Brought on Board (Retained/Discarded)	Live - Stomach and/or Eyes Protruding	Retained	Not Applicable	
52	NOCAM	Epinephelus morio	Red Grouper	N	Brought on Board (Retained/Discarded)	Live - Healthy	Discarded - Live and Healthy (Vented)	Not Applicable	
52	NOCAM	Epinephelus morio	Red Grouper	N	Brought on Board (Retained/Discarded)	Live - Healthy	Discarded - Live and Healthy (Vented)	Not Applicable	
52	NOCAM	Epinephelus morio	Red Grouper	N	Brought on Board (Retained/Discarded)	Dead on Arrival - Undamaged	Retained	Not Applicable	
52	NOCAM	Epinephelus morio	Red Grouper	N	Brought on Board (Retained/Discarded)	Live - Healthy	Discarded - Live and Healthy (Vented)	Not Applicable	
52	NOCAM	Epinephelus morio	Red Grouper	N	Brought on Board (Retained/Discarded)	Live - Stomach and/or Eyes Protruding	Retained	Not Applicable	
52	NOCAM	Epinephelus morio	Red Grouper	N	Brought on Board (Retained/Discarded)	Live - Stomach and/or Eyes Protruding	Retained	Not Applicable	
52	NOCAM	Epinephelus morio	Red Grouper	N	Brought on Board (Retained/Discarded)	Live - Healthy	Discarded - Live and Healthy (Vented)	Not Applicable	
52	NOCAM	Epinephelus morio	Red Grouper	N	Brought on Board (Retained/Discarded)	Live - Stomach and/or Eyes Protruding	Retained	Not Applicable	
52	NOCAM	Epinephelus morio	Red Grouper	N	Brought on Board (Retained/Discarded)	Dead on Arrival - Damaged	Discarded - Dead	Not Applicable	
52	NOCAM	Epinephelus morio	Red Grouper	N	Brought on Board (Retained/Discarded)	Live - Healthy	Discarded - Live and Healthy (Vented)	Not Applicable	
52	NOCAM	Epinephelus morio	Red Grouper	N	Brought on Board (Retained/Discarded)	Live - Healthy	Discarded - Live and Healthy (Vented)	Not Applicable	
52	NOCAM	Epinephelus morio	Red Grouper	N	Brought on Board (Retained/Discarded)	Live - Healthy	Discarded - Live and Healthy (Vented)	Not Applicable	
52	NOCAM	Epinephelus morio	Red Grouper	N	Brought on Board (Retained/Discarded)	Live - Healthy	Retained	Not Applicable	
52	NOCAM	Epinephelus morio	Red Grouper	N	Brought on Board (Retained/Discarded)	Live - Healthy	Retained	Not Applicable	
52	NOCAM	Epinephelus morio	Red Grouper	N	Brought on Board (Retained/Discarded)	Live - Stomach and/or Eyes Protruding	Retained	Not Applicable	
52	NOCAM	Epinephelus morio	Red Grouper	N	Brought on Board (Retained/Discarded)	Live - Healthy	Discarded - Live and Healthy (Vented)	Not Applicable	
52	NOCAM	Epinephelus morio	Red Grouper	N	Brought on Board (Retained/Discarded)	Live - Healthy	Retained	Not Applicable	
52	NOCAM	Epinephelus morio	Red Grouper	N	Brought on Board (Retained/Discarded)	Live - Healthy	Discarded - Live and Healthy (Vented)	Not Applicable	
52	NOCAM	Epinephelus morio	Red Grouper	N	Brought on Board (Retained/Discarded)	Live - Healthy	Discarded - Live and Healthy (Vented)	Not Applicable	
52	NOCAM	Epinephelus morio	Red Grouper	N	Brought on Board (Retained/Discarded)	Live - Healthy	Retained	Not Applicable	
52	NOCAM	Epinephelus morio	Red Grouper	N	Brought on Board (Retained/Discarded)	Live - Healthy	Retained	Not Applicable	
52	NOCAM	Epinephelus morio	Red Grouper	N	Brought on Board (Retained/Discarded)	Live - Healthy	Retained	Not Applicable	
52	NOCAM	Epinephelus morio	Red Grouper	N	Brought on Board (Retained/Discarded)	Live - Stomach and/or Eyes Protruding	Retained	Not Applicable	
52	NOCAM	Epinephelus morio	Red Grouper	N	Brought on Board (Retained/Discarded)	Live - Stomach and/or Eyes Protruding	Discarded - Live and Healthy (Vented)	Not Applicable	
52	NOCAM	Epinephelus morio	Red Grouper	N	Brought on Board (Retained/Discarded)	Live - Healthy	Discarded - Live and Healthy (Vented)	Not Applicable	
52	NOCAM	Epinephelus morio	Red Grouper	N	Brought on Board (Retained/Discarded)	Live - Healthy	Retained	Not Applicable	
52	NOCAM	Epinephelus morio	Red Grouper	N	Brought on Board (Retained/Discarded)	Live - Healthy	Retained	Not Applicable	
52	NOCAM	Epinephelus morio	Red Grouper	N	Brought on Board (Retained/Discarded)	Live - Stomach and/or Eyes Protruding	Discarded - Live and Healthy (Vented)	Not Applicable	
52	NOCAM	Epinephelus morio	Red Grouper	N	Brought on Board (Retained/Discarded)	Live - Stomach and/or Eyes Protruding	Retained	Not Applicable	
52	NOCAM	Epinephelus morio	Red Grouper	N	Brought on Board (Retained/Discarded)	Live - Stomach and/or Eyes Protruding	Discarded - Live and Healthy (Vented)	Not Applicable	
52	NOCAM	Epinephelus morio	Red Grouper	N	Brought on Board (Retained/Discarded)	Live - Healthy	Retained	Not Applicable	
52	NOCAM	Epinephelus morio	Red Grouper	N	Brought on Board (Retained/Discarded)	Live - Healthy	Retained	Not Applicable	
52	NOCAM	Epinephelus morio	Red Grouper	N	Brought on Board (Retained/Discarded)	Live - Stomach and/or Eyes Protruding	Retained	Not Applicable	
52	NOCAM	Epinephelus morio	Red Grouper	N	Brought on Board (Retained/Discarded)	Live - Healthy	Discarded - Live and Healthy (Vented)	Not Applicable	
52	NOCAM	Epinephelus morio	Red Grouper	N	Brought on Board (Retained/Discarded)	Live - Healthy	Discarded - Live and Healthy (Vented)	Not Applicable	
52	NOCAM	Epinephelus morio	Red Grouper	N	Brought on Board (Retained/Discarded)	Live - Stomach and/or Eyes Protruding	Discarded - Live and Healthy (Vented)	Not Applicable	
52	NOCAM	Epinephelus morio	Red Grouper	N	Brought on Board (Retained/Discarded)	Live - Healthy	Discarded - Live and Healthy (Vented)	Not Applicable	
52	NOCAM	Epinephelus morio	Red Grouper	N	Brought on Board (Retained/Discarded)	Live - Healthy	Retained	Not Applicable	
52	NOCAM	Epinephelus morio	Red Grouper	N	Brought on Board (Retained/Discarded)	Live - Stomach and/or Eyes Protruding	Retained	Not Applicable	
52	NOCAM	Epinephelus morio	Red Grouper	N	Brought on Board (Retained/Discarded)	Live - Healthy	Retained	Not Applicable	

52	NOCAM	Epinephelus morio	Red Grouper	N	Brought on Board (Retained/Discarded)	Live - Stomach and/or Eyes Protruding	Retained	Not Applicable	
52	NOCAM	Galeocerdo cuvier	Tiger Shark	Y	Cut-off at Rail (No Entanglement)	Live - Healthy	Discarded - Live and Healthy (Not Vented)	Unknown Maturity and/or Sex	Large (Greater than 2m)
52	NOCAM	Lutjanus analis	Mutton Snapper	N	Brought on Board (Retained/Discarded)	Live - Healthy	Retained	Not Applicable	
52	NOCAM	Lutjanus analis	Mutton Snapper	N	Brought on Board (Retained/Discarded)	Live - Healthy	Retained	Not Applicable	
52	NOCAM	Lutjanus campechanus	Red Snapper	N	Brought on Board (Retained/Discarded)	Dead on Arrival - Damaged	Discarded - Dead	Not Applicable	
52	NOCAM	Lutjanus campechanus	Red Snapper	N	Brought on Board (Retained/Discarded)	Live - Healthy	Discarded - Live and Healthy (Vented)	Not Applicable	
52	NOCAM	Lutjanus campechanus	Red Snapper	N	Brought on Board (Retained/Discarded)	Live - Healthy	Discarded - Live and Healthy (Vented)	Not Applicable	
52	NOCAM	Lutjanus campechanus	Red Snapper	N	Brought on Board (Retained/Discarded)	Live - Healthy	Discarded - Live and Healthy (Vented)	Not Applicable	
52	NOCAM	Lutjanus campechanus	Red Snapper	N	Brought on Board (Retained/Discarded)	Live - Stomach and/or Eyes Protruding	Discarded - Live and Healthy (Vented)	Not Applicable	
52	NOCAM	Lutjanus campechanus	Red Snapper	N	Brought on Board (Retained/Discarded)	Live - Healthy	Discarded - Live and Healthy (Vented)	Not Applicable	
52	NOCAM	Lutjanus campechanus	Red Snapper	N	Brought on Board (Retained/Discarded)	Live - Healthy	Discarded - Live and Healthy (Vented)	Not Applicable	
52	NOCAM	Rhizoprionodon terraenovae	Atlantic Sharpnose Shark	Y	Brought on Board (Retained/Discarded)	Live - Healthy	Discarded - Live and Healthy (Not Vented)	Male (Claspers)	Small (Less than 1m)
52	UCAM	Carcharhinus plumbeus	Sandbar Shark	Y	Cut-off at Rail (No Entanglement)	Live - Healthy	Discarded - Live and Healthy (Not Vented)	Female	Large (Greater than 2m)
52	UCAM	Carcharhinus plumbeus	Sandbar Shark	Y	Cut-off at Rail (No Entanglement)	Live - Healthy	Discarded - Live and Healthy (Not Vented)	Female	Large (Greater than 2m)
52	UCAM	Epinephelus morio	Red Grouper	N	Brought on Board (Retained/Discarded)	Live - Healthy	Retained	Not Applicable	
52	UCAM	Epinephelus morio	Red Grouper	N	Brought on Board (Retained/Discarded)	Live - Healthy	Discarded - Live and Healthy (Vented)	Not Applicable	
52	UCAM	Epinephelus morio	Red Grouper	N	Brought on Board (Retained/Discarded)	Live - Healthy	Retained	Not Applicable	
52	UCAM	Epinephelus morio	Red Grouper	N	Brought on Board (Retained/Discarded)	Live - Healthy	Retained	Not Applicable	
52	UCAM	Epinephelus morio	Red Grouper	N	Brought on Board (Retained/Discarded)	Live - Healthy	Retained	Not Applicable	
52	UCAM	Epinephelus morio	Red Grouper	N	Brought on Board (Retained/Discarded)	Live - Healthy	Retained	Not Applicable	
52	UCAM	Epinephelus morio	Red Grouper	N	Brought on Board (Retained/Discarded)	Live - Stomach and/or Eyes Protruding	Discarded - Live and Healthy (Vented)	Not Applicable	
52	UCAM	Epinephelus morio	Red Grouper	N	Brought on Board (Retained/Discarded)	Live - Stomach and/or Eyes Protruding	Retained	Not Applicable	
52	UCAM	Epinephelus morio	Red Grouper	N	Brought on Board (Retained/Discarded)	Live - Stomach and/or Eyes Protruding	Retained	Not Applicable	
52	UCAM	Epinephelus morio	Red Grouper	N	Brought on Board (Retained/Discarded)	Live - Stomach and/or Eyes Protruding	Retained	Not Applicable	
52	UCAM	Epinephelus morio	Red Grouper	N	Brought on Board (Retained/Discarded)	Live - Healthy	Retained	Not Applicable	
52	UCAM	Epinephelus morio	Red Grouper	N	Brought on Board (Retained/Discarded)	Live - Stomach and/or Eyes Protruding	Discarded - Live and Healthy (Vented)	Not Applicable	
52	UCAM	Epinephelus morio	Red Grouper	N	Brought on Board (Retained/Discarded)	Live - Healthy	Discarded - Live and Healthy (Vented)	Not Applicable	
52	UCAM	Epinephelus morio	Red Grouper	N	Brought on Board (Retained/Discarded)	Live - Healthy	Retained	Not Applicable	
52	UCAM	Epinephelus morio	Red Grouper	N	Brought on Board (Retained/Discarded)	Live - Healthy	Discarded - Live and Healthy (Vented)	Not Applicable	
52	UCAM	Epinephelus morio	Red Grouper	N	Brought on Board (Retained/Discarded)	Live - Healthy	Discarded - Live and Healthy (Not Vented)	Not Applicable	
52	UCAM	Epinephelus morio	Red Grouper	N	Brought on Board (Retained/Discarded)	Live - Healthy	Discarded - Live and Healthy (Vented)	Not Applicable	
52	UCAM	Epinephelus morio	Red Grouper	N	Brought on Board (Retained/Discarded)	Live - Healthy	Retained	Not Applicable	
52	UCAM	Epinephelus morio	Red Grouper	N	Brought on Board (Retained/Discarded)	Live - Healthy	Discarded - Live and Healthy (Vented)	Not Applicable	
52	UCAM	Epinephelus morio	Red Grouper	N	Brought on Board (Retained/Discarded)	Live - Healthy	Retained	Not Applicable	
52	UCAM	Epinephelus morio	Red Grouper	N	Brought on Board (Retained/Discarded)	Live - Healthy	Discarded - Live and Healthy (Vented)	Not Applicable	
52	UCAM	Epinephelus morio	Red Grouper	N	Brought on Board (Retained/Discarded)	Live - Healthy	Retained	Not Applicable	
52	UCAM	Epinephelus morio	Red Grouper	N	Brought on Board (Retained/Discarded)	Live - Healthy	Retained	Not Applicable	
52	UCAM	Epinephelus morio	Red Grouper	N	Brought on Board (Retained/Discarded)	Live - Healthy	Retained	Not Applicable	
52	UCAM	Epinephelus morio	Red Grouper	N	Brought on Board (Retained/Discarded)	Live - Stomach and/or Eyes Protruding	Retained	Not Applicable	
52	UCAM	Epinephelus morio	Red Grouper	N	Brought on Board (Retained/Discarded)	Live - Stomach and/or Eyes Protruding	Retained	Not Applicable	
52	UCAM	Epinephelus morio	Red Grouper	N	Brought on Board (Retained/Discarded)	Live - Healthy	Retained	Not Applicable	
52	UCAM	Epinephelus morio	Red Grouper	N	Brought on Board (Retained/Discarded)	Live - Stomach and/or Eyes Protruding	Retained	Not Applicable	
52	UCAM	Epinephelus morio	Red Grouper	N	Brought on Board (Retained/Discarded)	Live - Healthy	Discarded - Live and Healthy (Vented)	Not Applicable	
52	UCAM	Epinephelus morio	Red Grouper	N	Brought on Board (Retained/Discarded)	Live - Healthy	Discarded - Live and Healthy (Vented)	Not Applicable	
52	UCAM	Epinephelus morio	Red Grouper	N	Brought on Board (Retained/Discarded)	Live - Healthy	Retained	Not Applicable	
52	UCAM	Epinephelus morio	Red Grouper	N	Brought on Board (Retained/Discarded)	Live - Stomach and/or Eyes Protruding	Retained	Not Applicable	
52	UCAM	Epinephelus morio	Red Grouper	N	Brought on Board (Retained/Discarded)	Live - Healthy	Discarded - Live and Healthy (Vented)	Not Applicable	
52	UCAM	Epinephelus morio	Red Grouper	N	Brought on Board (Retained/Discarded)	Live - Stomach and/or Eyes Protruding	Retained	Not Applicable	
52	UCAM	Epinephelus morio	Red Grouper	N	Brought on Board (Retained/Discarded)	Live - Healthy	Discarded - Live and Healthy (Vented)	Not Applicable	
52	UCAM	Epinephelus morio	Red Grouper	N	Brought on Board (Retained/Discarded)	Live - Healthy	Retained	Not Applicable	
52	UCAM	Epinephelus morio	Red Grouper	N	Brought on Board (Retained/Discarded)	Live - Healthy	Discarded - Live and Healthy (Vented)	Not Applicable	
52	UCAM	Epinephelus morio	Red Grouper	N	Brought on Board (Retained/Discarded)	Live - Healthy	Retained	Not Applicable	
52	UCAM	Epinephelus morio	Red Grouper	N	Brought on Board (Retained/Discarded)	Live - Healthy	Retained	Not Applicable	
52	UCAM	Epinephelus morio	Red Grouper	N	Brought on Board (Retained/Discarded)	Live - Stomach and/or Eyes Protruding	Retained	Not Applicable	
52	UCAM	Epinephelus morio	Red Grouper	N	Brought on Board (Retained/Discarded)	Live - Healthy	Retained	Not Applicable	
52	UCAM	Epinephelus morio	Red Grouper	N	Brought on Board (Retained/Discarded)	Live - Stomach and/or Eyes Protruding	Retained	Not Applicable	
52	UCAM	Epinephelus morio	Red Grouper	N	Brought on Board (Retained/Discarded)	Live - Stomach and/or Eyes Protruding	Retained	Not Applicable	
52	UCAM	Epinephelus morio	Red Grouper	N	Brought on Board (Retained/Discarded)	Live - Healthy	Discarded - Live and Healthy (Vented)	Not Applicable	
52	UCAM	Epinephelus morio	Red Grouper	N	Brought on Board (Retained/Discarded)	Live - Stomach and/or Eyes Protruding	Discarded - Live and Healthy (Vented)	Not Applicable	
52	UCAM	Epinephelus morio	Red Grouper	N	Brought on Board (Retained/Discarded)	Live - Stomach and/or Eyes Protruding	Retained	Not Applicable	
52	UCAM	Epinephelus morio	Red Grouper	N	Brought on Board (Retained/Discarded)	Live - Healthy	Retained	Not Applicable	
52	UCAM	Epinephelus morio	Red Grouper	N	Brought on Board (Retained/Discarded)	Live - Stomach and/or Eyes Protruding	Retained	Not Applicable	
52	UCAM	Epinephelus morio	Red Grouper	N	Brought on Board (Retained/Discarded)	Live - Stomach and/or Eyes Protruding	Retained	Not Applicable	
52	UCAM	Epinephelus morio	Red Grouper	N	Brought on Board (Retained/Discarded)	Live - Stomach and/or Eyes Protruding	Discarded - Live and Healthy (Vented)	Not Applicable	
52	UCAM	Epinephelus morio	Red Grouper	N	Brought on Board (Retained/Discarded)	Live - Healthy	Retained	Not Applicable	
52	UCAM	Epinephelus morio	Red Grouper	N	Brought on Board (Retained/Discarded)	Live - Healthy	Discarded - Live and Healthy (Vented)	Not Applicable	
52	UCAM	Galeocerdo cuvier	Tiger Shark	Y	Cut-off at Rail (No Entanglement)	Live - Healthy	Discarded - Live and Healthy (Not Vented)	Female	Large (Greater than 2m)
52	UCAM	Galeocerdo cuvier	Tiger Shark	Y	Cut-off at Rail (No Entanglement)	Live - Healthy	Discarded - Live and Healthy (Not Vented)	Female	Large (Greater than 2m)
52	UCAM	Lutjanus analis	Mutton Snapper	N	Brought on Board (Retained/Discarded)	Live - Healthy	Retained	Not Applicable	
52	UCAM	Lutjanus analis	Mutton Snapper	N	Brought on Board (Retained/Discarded)	Live - Healthy	Retained	Not Applicable	
52	UCAM	Lutjanus campechanus	Red Snapper	N	Brought on Board (Retained/Discarded)	Dead on Arrival - Undamaged	Discarded - Dead	Not Applicable	
52	UCAM	Lutjanus campechanus	Red Snapper	N	Brought on Board (Retained/Discarded)	Live - Healthy	Discarded - Live and Healthy (Vented)	Not Applicable	
52	UCAM	Lutjanus campechanus	Red Snapper	N	Brought on Board (Retained/Discarded)	Live - Stomach and/or Eyes Protruding	Discarded - Live and Healthy (Vented)	Not Applicable	
52	UCAM	Lutjanus campechanus	Red Snapper	N	Brought on Board (Retained/Discarded)	Live - Healthy	Discarded - Live and Healthy (Vented)	Not Applicable	
52	UCAM	Lutjanus campechanus	Red Snapper	N	Brought on Board (Retained/Discarded)	Dead on Arrival - Damaged	Discarded - Dead	Not Applicable	
52	UCAM	Lutjanus campechanus	Red Snapper	N	Brought on Board (Retained/Discarded)	Live - Stomach and/or Eyes Protruding	Discarded - Live and Healthy (Vented)	Not Applicable	
52	UCAM	Lutjanus campechanus	Red Snapper	N	Brought on Board (Retained/Discarded)	Dead on Arrival - Damaged	Discarded - Dead	Not Applicable	
52	UCAM	Lutjanus campechanus	Red Snapper	N	Brought on Board (Retained/Discarded)	Live - Healthy	Discarded - Live and Healthy (Vented)	Not Applicable	
52	UCAM	Rhizoprionodon terraenovae	Atlantic Sharpnose Shark	Y	Brought on Board (Retained/Discarded)	Live - Healthy	Discarded - Live and Healthy (Not Vented)	Male (Claspers)	Small (Less than 1m)
53	NOCAM	Calamus bajonada	Jolthead Porgy	N	Brought on Board (Retained/Discarded)	Live - Healthy	Retained	Not Applicable	
53	NOCAM	Epinephelus morio	Red Grouper	N	Brought on Board (Retained/Discarded)	Live - Stomach and/or Eyes Protruding	Retained	Not Applicable	
53	NOCAM	Epinephelus morio	Red Grouper	N	Brought on Board (Retained/Discarded)	Live - Healthy	Retained	Not Applicable	
53	NOCAM	Epinephelus morio	Red Grouper	N	Brought on Board (Retained/Discarded)	Live - Healthy	Retained	Not Applicable	
53	NOCAM	Epinephelus morio	Red Grouper	N	Brought on Board (Retained/Discarded)	Live - Stomach and/or Eyes Protruding	Retained	Not Applicable	
53	NOCAM	Epinephelus morio	Red Grouper	N	Brought on Board (Retained/Discarded)	Live - Stomach and/or Eyes Protruding	Retained	Not Applicable	
53	NOCAM	Epinephelus morio	Red Grouper	N	Brought on Board (Retained/Discarded)	Live - Healthy	Discarded - Live and Healthy (Vented)	Not Applicable	
53	NOCAM	Epinephelus morio	Red Grouper	N	Brought on Board (Retained/Discarded)	Live - Healthy	Discarded - Live and Healthy (Vented)	Not Applicable	
53	NOCAM	Epinephelus morio	Red Grouper	N	Brought on Board (Retained/Discarded)	Live - Healthy	Retained	Not Applicable	
53	NOCAM	Epinephelus morio	Red Grouper	N	Brought on Board (Retained/Discarded)	Live - Stomach and/or Eyes Protruding	Retained	Not Applicable	
53	NOCAM	Epinephelus morio	Red Grouper	N	Brought on Board (Retained/Discarded)	Live - Stomach and/or Eyes Protruding	Retained	Not Applicable	
53	NOCAM	Epinephelus morio	Red Grouper	N	Brought on Board (Retained/Discarded)	Live - Healthy	Discarded - Live and Healthy (Vented)	Not Applicable	
53	NOCAM	Epinephelus morio	Red Grouper	N	Brought on Board (Retained/Discarded)	Live - Healthy	Retained	Not Applicable	

53	NOCAM	Epinephelus morio	Red Grouper	N	Brought on Board (Retained/Discarded)	Live - Stomach and/or Eyes Protruding	Retained	Not Applicable	
53	NOCAM	Epinephelus morio	Red Grouper	N	Brought on Board (Retained/Discarded)	Live - Stomach and/or Eyes Protruding	Retained	Not Applicable	
53	NOCAM	Epinephelus morio	Red Grouper	N	Brought on Board (Retained/Discarded)	Live - Stomach and/or Eyes Protruding	Retained	Not Applicable	
53	NOCAM	Epinephelus morio	Red Grouper	N	Brought on Board (Retained/Discarded)	Live - Stomach and/or Eyes Protruding	Discarded - Live and Healthy (Vented)	Not Applicable	
53	NOCAM	Epinephelus morio	Red Grouper	N	Brought on Board (Retained/Discarded)	Live - Healthy	Retained	Not Applicable	
53	NOCAM	Epinephelus morio	Red Grouper	N	Brought on Board (Retained/Discarded)	Live - Healthy	Retained	Not Applicable	
53	NOCAM	Epinephelus morio	Red Grouper	N	Brought on Board (Retained/Discarded)	Live - Healthy	Discarded - Live and Damaged (Not Vente	Not Applicable	
53	NOCAM	Epinephelus morio	Red Grouper	N	Brought on Board (Retained/Discarded)	Live - Stomach and/or Eyes Protruding	Retained	Not Applicable	
53	NOCAM	Epinephelus morio	Red Grouper	N	Brought on Board (Retained/Discarded)	Live - Stomach and/or Eyes Protruding	Retained	Not Applicable	
53	NOCAM	Epinephelus morio	Red Grouper	N	Brought on Board (Retained/Discarded)	Live - Stomach and/or Eyes Protruding	Retained	Not Applicable	
53	NOCAM	Epinephelus morio	Red Grouper	N	Brought on Board (Retained/Discarded)	Live - Stomach and/or Eyes Protruding	Discarded - Live and Healthy (Vented)	Not Applicable	
53	NOCAM	Epinephelus morio	Red Grouper	N	Brought on Board (Retained/Discarded)	Live - Stomach and/or Eyes Protruding	Retained	Not Applicable	
53	NOCAM	Lutjanus analis	Mutton Snapper	N	Brought on Board (Retained/Discarded)	Dead on Arrival - Damaged	Discarded - Dead	Not Applicable	
53	NOCAM	Lutjanus campechanus	Red Snapper	N	Brought on Board (Retained/Discarded)	Dead on Arrival - Damaged	Discarded - Dead	Not Applicable	
53	NOCAM	Lutjanus campechanus	Red Snapper	N	Brought on Board (Retained/Discarded)	Live - Healthy	Discarded - Live and Healthy (Vented)	Not Applicable	
53	NOCAM	Lutjanus campechanus	Red Snapper	N	Brought on Board (Retained/Discarded)	Dead on Arrival - Undamaged	Discarded - Dead	Not Applicable	
53	NOCAM	Lutjanus campechanus	Red Snapper	N	Brought on Board (Retained/Discarded)	Live - Healthy	Discarded - Live and Healthy (Vented)	Not Applicable	
53	NOCAM	Lutjanus campechanus	Red Snapper	N	Brought on Board (Retained/Discarded)	Dead on Arrival - Undamaged	Discarded - Dead	Not Applicable	
53	NOCAM	Lutjanus campechanus	Red Snapper	N	Brought on Board (Retained/Discarded)	Live - Healthy	Discarded - Live and Healthy (Vented)	Not Applicable	
53	NOCAM	Rhizoprionodon terraenovae	Atlantic Sharpnose Shark	Y	Brought on Board (Retained/Discarded)	Live - Healthy	Discarded - Live and Healthy (Not Vented)	Male (Claspers)	Small (Less than 1m)
53	NOCAM	Selachimorpha, unidentified	Shark, Unidentified	Y	Cut-off at Rail (No Entanglement)	Live - Healthy	Discarded - Live and Healthy (Not Vented)	Unknown Maturity and/or Sex	Large (Greater than 2m)
53	UCAM	Calamus bajonada	Jolthead Porgy	N	Brought on Board (Retained/Discarded)	Live - Healthy	Retained	Not Applicable	
53	UCAM	Carcharhinus plumbeus	Sandbar Shark	Y	Cut-off at Rail (No Entanglement)	Live - Healthy	Discarded - Live and Healthy (Not Vented)	Female	Large (Greater than 2m)
53	UCAM	Carcharhinus plumbeus	Sandbar Shark	Y	Cut-off at Rail (No Entanglement)	Live - Healthy	Discarded - Live and Healthy (Not Vented)	Female	Large (Greater than 2m)
53	UCAM	Epinephelus morio	Red Grouper	N	Brought on Board (Retained/Discarded)	Live - Healthy	Retained	Not Applicable	
53	UCAM	Epinephelus morio	Red Grouper	N	Brought on Board (Retained/Discarded)	Live - Stomach and/or Eyes Protruding	Retained	Not Applicable	
53	UCAM	Epinephelus morio	Red Grouper	N	Brought on Board (Retained/Discarded)	Live - Stomach and/or Eyes Protruding	Retained	Not Applicable	
53	UCAM	Epinephelus morio	Red Grouper	N	Brought on Board (Retained/Discarded)	Live - Stomach and/or Eyes Protruding	Retained	Not Applicable	
53	UCAM	Epinephelus morio	Red Grouper	N	Brought on Board (Retained/Discarded)	Live - Stomach and/or Eyes Protruding	Retained	Not Applicable	
53	UCAM	Epinephelus morio	Red Grouper	N	Brought on Board (Retained/Discarded)	Live - Healthy	Discarded - Live and Healthy (Vented)	Not Applicable	
53	UCAM	Epinephelus morio	Red Grouper	N	Brought on Board (Retained/Discarded)	Live - Healthy	Retained	Not Applicable	
53	UCAM	Epinephelus morio	Red Grouper	N	Brought on Board (Retained/Discarded)	Live - Stomach and/or Eyes Protruding	Retained	Not Applicable	
53	UCAM	Epinephelus morio	Red Grouper	N	Brought on Board (Retained/Discarded)	Live - Healthy	Retained	Not Applicable	
53	UCAM	Epinephelus morio	Red Grouper	N	Brought on Board (Retained/Discarded)	Live - Stomach and/or Eyes Protruding	Retained	Not Applicable	
53	UCAM	Epinephelus morio	Red Grouper	N	Brought on Board (Retained/Discarded)	Live - Stomach and/or Eyes Protruding	Retained	Not Applicable	
53	UCAM	Epinephelus morio	Red Grouper	N	Brought on Board (Retained/Discarded)	Live - Healthy	Discarded - Live and Healthy (Vented)	Not Applicable	
53	UCAM	Epinephelus morio	Red Grouper	N	Brought on Board (Retained/Discarded)	Live - Healthy	Retained	Not Applicable	
53	UCAM	Epinephelus morio	Red Grouper	N	Brought on Board (Retained/Discarded)	Live - Stomach and/or Eyes Protruding	Discarded - Live and Healthy (Vented)	Not Applicable	
53	UCAM	Epinephelus morio	Red Grouper	N	Brought on Board (Retained/Discarded)	Live - Stomach and/or Eyes Protruding	Retained	Not Applicable	
53	UCAM	Epinephelus morio	Red Grouper	N	Brought on Board (Retained/Discarded)	Live - Stomach and/or Eyes Protruding	Discarded - Live and Healthy (Vented)	Not Applicable	
53	UCAM	Epinephelus morio	Red Grouper	N	Brought on Board (Retained/Discarded)	Live - Stomach and/or Eyes Protruding	Retained	Not Applicable	
53	UCAM	Epinephelus morio	Red Grouper	N	Brought on Board (Retained/Discarded)	Live - Healthy	Retained	Not Applicable	
53	UCAM	Epinephelus morio	Red Grouper	N	Brought on Board (Retained/Discarded)	Live - Healthy	Retained	Not Applicable	
53	UCAM	Epinephelus morio	Red Grouper	N	Brought on Board (Retained/Discarded)	Live - Healthy	Retained	Not Applicable	
53	UCAM	Epinephelus morio	Red Grouper	N	Brought on Board (Retained/Discarded)	Live - Healthy	Discarded - Live and Healthy (Not Vented)	Not Applicable	
53	UCAM	Epinephelus morio	Red Grouper	N	Brought on Board (Retained/Discarded)	Live - Stomach and/or Eyes Protruding	Retained	Not Applicable	
53	UCAM	Epinephelus morio	Red Grouper	N	Brought on Board (Retained/Discarded)	Live - Healthy	Discarded - Live and Healthy (Not Vented)	Not Applicable	
53	UCAM	Epinephelus morio	Red Grouper	N	Brought on Board (Retained/Discarded)	Live - Healthy	Retained	Not Applicable	
53	UCAM	Lutjanus analis	Mutton Snapper	N	Brought on Board (Retained/Discarded)	Dead on Arrival - Damaged	Discarded - Dead	Not Applicable	
53	UCAM	Lutjanus campechanus	Red Snapper	N	Brought on Board (Retained/Discarded)	Dead on Arrival - Undamaged	Discarded - Dead	Not Applicable	
53	UCAM	Lutjanus campechanus	Red Snapper	N	Brought on Board (Retained/Discarded)	Live - Healthy	Discarded - Live and Healthy (Vented)	Not Applicable	
53	UCAM	Lutjanus campechanus	Red Snapper	N	Brought on Board (Retained/Discarded)	Live - Healthy	Discarded - Live and Healthy (Vented)	Not Applicable	
53	UCAM	Lutjanus campechanus	Red Snapper	N	Brought on Board (Retained/Discarded)	Live - Healthy	Discarded - Live and Healthy (Vented)	Not Applicable	
53	UCAM	Lutjanus campechanus	Red Snapper	N	Brought on Board (Retained/Discarded)	Live - Healthy	Discarded - Live and Healthy (Vented)	Not Applicable	
53	UCAM	Lutjanus campechanus	Red Snapper	N	Brought on Board (Retained/Discarded)	Dead on Arrival - Damaged	Discarded - Dead	Not Applicable	
53	UCAM	Rhizoprionodon terraenovae	Atlantic Sharpnose Shark	Y	Brought on Board (Retained/Discarded)	Live - Healthy	Discarded - Live and Healthy (Not Vented)	Male (Claspers)	Small (Less than 1m)
54	NOCAM	Epinephelus morio	Red Grouper	N	Brought on Board (Retained/Discarded)	Live - Healthy	Discarded - Live and Healthy (Vented)	Not Applicable	
54	NOCAM	Epinephelus morio	Red Grouper	N	Brought on Board (Retained/Discarded)	Live - Healthy	Discarded - Live and Healthy (Vented)	Not Applicable	
54	NOCAM	Epinephelus morio	Red Grouper	N	Brought on Board (Retained/Discarded)	Live - Stomach and/or Eyes Protruding	Retained	Not Applicable	
54	NOCAM	Epinephelus morio	Red Grouper	N	Brought on Board (Retained/Discarded)	Live - Healthy	Retained	Not Applicable	
54	NOCAM	Epinephelus morio	Red Grouper	N	Brought on Board (Retained/Discarded)	Live - Healthy	Discarded - Live and Healthy (Vented)	Not Applicable	
54	NOCAM	Epinephelus morio	Red Grouper	N	Brought on Board (Retained/Discarded)	Live - Stomach and/or Eyes Protruding	Retained	Not Applicable	
54	NOCAM	Epinephelus morio	Red Grouper	N	Brought on Board (Retained/Discarded)	Live - Stomach and/or Eyes Protruding	Retained	Not Applicable	
54	NOCAM	Epinephelus morio	Red Grouper	N	Brought on Board (Retained/Discarded)	Live - Stomach and/or Eyes Protruding	Retained	Not Applicable	
54	NOCAM	Epinephelus morio	Red Grouper	N	Brought on Board (Retained/Discarded)	Live - Healthy	Retained	Not Applicable	
54	NOCAM	Epinephelus morio	Red Grouper	N	Brought on Board (Retained/Discarded)	Live - Stomach and/or Eyes Protruding	Retained	Not Applicable	
54	NOCAM	Epinephelus morio	Red Grouper	N	Brought on Board (Retained/Discarded)	Live - Stomach and/or Eyes Protruding	Retained	Not Applicable	
54	NOCAM	Epinephelus morio	Red Grouper	N	Brought on Board (Retained/Discarded)	Live - Healthy	Discarded - Live and Healthy (Vented)	Not Applicable	
54	NOCAM	Epinephelus morio	Red Grouper	N	Brought on Board (Retained/Discarded)	Live - Stomach and/or Eyes Protruding	Retained	Not Applicable	
54	NOCAM	Epinephelus morio	Red Grouper	N	Brought on Board (Retained/Discarded)	Live - Healthy	Discarded - Live and Healthy (Vented)	Not Applicable	
54	NOCAM	Epinephelus morio	Red Grouper	N	Brought on Board (Retained/Discarded)	Live - Healthy	Discarded - Live and Healthy (Vented)	Not Applicable	
54	NOCAM	Epinephelus morio	Red Grouper	N	Brought on Board (Retained/Discarded)	Live - Healthy	Discarded - Live and Healthy (Vented)	Not Applicable	
54	NOCAM	Epinephelus morio	Red Grouper	N	Brought on Board (Retained/Discarded)	Live - Stomach and/or Eyes Protruding	Retained	Not Applicable	
54	NOCAM	Epinephelus morio	Red Grouper	N	Brought on Board (Retained/Discarded)	Live - Stomach and/or Eyes Protruding	Retained	Not Applicable	
54	NOCAM	Epinephelus morio	Red Grouper	N	Brought on Board (Retained/Discarded)	Dead on Arrival - Undamaged	Retained	Not Applicable	
54	NOCAM	Epinephelus morio	Red Grouper	N	Brought on Board (Retained/Discarded)	Live - Healthy	Discarded - Live and Healthy (Vented)	Not Applicable	
54	NOCAM	Epinephelus morio	Red Grouper	N	Brought on Board (Retained/Discarded)	Live - Stomach and/or Eyes Protruding	Retained	Not Applicable	
54	NOCAM	Epinephelus morio	Red Grouper	N	Brought on Board (Retained/Discarded)	Live - Stomach and/or Eyes Protruding	Discarded - Live and Healthy (Vented)	Not Applicable	
54	NOCAM	Epinephelus morio	Red Grouper	N	Brought on Board (Retained/Discarded)	Live - Stomach and/or Eyes Protruding	Retained	Not Applicable	
54	NOCAM	Epinephelus morio	Red Grouper	N	Brought on Board (Retained/Discarded)	Live - Healthy	Discarded - Live and Healthy (Vented)	Not Applicable	
54	NOCAM	Epinephelus morio	Red Grouper	N	Brought on Board (Retained/Discarded)	Live - Healthy	Discarded - Live and Healthy (Vented)	Not Applicable	
54	NOCAM	Epinephelus morio	Red Grouper	N	Brought on Board (Retained/Discarded)	Live - Healthy	Discarded - Live and Healthy (Vented)	Not Applicable	
54	NOCAM	Epinephelus morio	Red Grouper	N	Brought on Board (Retained/Discarded)	Live - Stomach and/or Eyes Protruding	Retained	Not Applicable	
54	NOCAM	Epinephelus morio	Red Grouper	N	Brought on Board (Retained/Discarded)	Live - Healthy	Discarded - Live and Healthy (Vented)	Not Applicable	
54	NOCAM	Epinephelus morio	Red Grouper	N	Brought on Board (Retained/Discarded)	Live - Stomach and/or Eyes Protruding	Retained	Not Applicable	
54	NOCAM	Epinephelus morio	Red Grouper	N	Brought on Board (Retained/Discarded)	Live - Healthy	Discarded - Live and Healthy (Vented)	Not Applicable	
54	NOCAM	Epinephelus morio	Red Grouper	N	Brought on Board (Retained/Discarded)	Live - Healthy	Discarded - Live and Healthy (Vented)	Not Applicable	
54	NOCAM	Epinephelus morio	Red Grouper	N	Brought on Board (Retained/Discarded)	Live - Stomach and/or Eyes Protruding	Retained	Not Applicable	
54	NOCAM	Epinephelus morio	Red Grouper	N	Brought on Board (Retained/Discarded)	Live - Stomach and/or Eyes Protruding	Retained	Not Applicable	
54	NOCAM	Galeocerdo cuvier	Tiger Shark	Y	Cut-off at Rail (No Entanglement)	Live - Healthy	Discarded - Live and Healthy (Not Vented)	Known Juvenile	Medium (Between 1m and 2m)
54	NOCAM	Lutjanus analis	Mutton Snapper	N	Brought on Board (Retained/Discarded)	Live - Healthy	Retained	Not Applicable	
54	NOCAM	Lutjanus analis	Mutton Snapper	N	Brought on Board (Retained/Discarded)	Live - Healthy	Retained	Not Applicable	

54	NOCAM	Lutjanus campechanus	Red Snapper	N	Brought on Board (Retained/Discarded)	Live - Healthy	Discarded - Live and Healthy (Vented)	Not Applicable	
54	NOCAM	Lutjanus campechanus	Red Snapper	N	Brought on Board (Retained/Discarded)	Live - Healthy	Discarded - Live and Healthy (Vented)	Not Applicable	
54	NOCAM	Lutjanus griseus	Gray (Mangrove) Snapper	N	Brought on Board (Retained/Discarded)	Live - Healthy	Retained	Not Applicable	
54	UCAM	Epinephelus morio	Red Grouper	N	Brought on Board (Retained/Discarded)	Live - Stomach and/or Eyes Protruding	Retained	Not Applicable	
54	UCAM	Epinephelus morio	Red Grouper	N	Brought on Board (Retained/Discarded)	Live - Healthy	Discarded - Live and Healthy (Vented)	Not Applicable	
54	UCAM	Epinephelus morio	Red Grouper	N	Brought on Board (Retained/Discarded)	Live - Healthy	Discarded - Live and Healthy (Vented)	Not Applicable	
54	UCAM	Epinephelus morio	Red Grouper	N	Brought on Board (Retained/Discarded)	Live - Healthy	Retained	Not Applicable	
54	UCAM	Epinephelus morio	Red Grouper	N	Brought on Board (Retained/Discarded)	Live - Healthy	Discarded - Live and Healthy (Vented)	Not Applicable	
54	UCAM	Epinephelus morio	Red Grouper	N	Brought on Board (Retained/Discarded)	Dead on Arrival - Undamaged	Retained	Not Applicable	
54	UCAM	Epinephelus morio	Red Grouper	N	Brought on Board (Retained/Discarded)	Live - Healthy	Discarded - Live and Healthy (Vented)	Not Applicable	
54	UCAM	Epinephelus morio	Red Grouper	N	Brought on Board (Retained/Discarded)	Live - Healthy	Discarded - Live and Healthy (Vented)	Not Applicable	
54	UCAM	Epinephelus morio	Red Grouper	N	Brought on Board (Retained/Discarded)	Live - Stomach and/or Eyes Protruding	Retained	Not Applicable	
54	UCAM	Epinephelus morio	Red Grouper	N	Brought on Board (Retained/Discarded)	Live - Healthy	Discarded - Live and Healthy (Vented)	Not Applicable	
54	UCAM	Epinephelus morio	Red Grouper	N	Brought on Board (Retained/Discarded)	Live - Healthy	Discarded - Live and Healthy (Vented)	Not Applicable	
54	UCAM	Epinephelus morio	Red Grouper	N	Brought on Board (Retained/Discarded)	Live - Stomach and/or Eyes Protruding	Retained	Not Applicable	
54	UCAM	Epinephelus morio	Red Grouper	N	Brought on Board (Retained/Discarded)	Live - Healthy	Retained	Not Applicable	
54	UCAM	Epinephelus morio	Red Grouper	N	Brought on Board (Retained/Discarded)	Live - Stomach and/or Eyes Protruding	Retained	Not Applicable	
54	UCAM	Epinephelus morio	Red Grouper	N	Brought on Board (Retained/Discarded)	Live - Stomach and/or Eyes Protruding	Retained	Not Applicable	
54	UCAM	Epinephelus morio	Red Grouper	N	Brought on Board (Retained/Discarded)	Live - Stomach and/or Eyes Protruding	Retained	Not Applicable	
54	UCAM	Epinephelus morio	Red Grouper	N	Brought on Board (Retained/Discarded)	Live - Healthy	Discarded - Live and Healthy (Vented)	Not Applicable	
54	UCAM	Epinephelus morio	Red Grouper	N	Brought on Board (Retained/Discarded)	Live - Stomach and/or Eyes Protruding	Retained	Not Applicable	
54	UCAM	Epinephelus morio	Red Grouper	N	Brought on Board (Retained/Discarded)	Live - Stomach and/or Eyes Protruding	Retained	Not Applicable	
54	UCAM	Epinephelus morio	Red Grouper	N	Brought on Board (Retained/Discarded)	Live - Healthy	Retained	Not Applicable	
54	UCAM	Epinephelus morio	Red Grouper	N	Brought on Board (Retained/Discarded)	Live - Healthy	Discarded - Live and Healthy (Vented)	Not Applicable	
54	UCAM	Epinephelus morio	Red Grouper	N	Brought on Board (Retained/Discarded)	Live - Stomach and/or Eyes Protruding	Retained	Not Applicable	
54	UCAM	Epinephelus morio	Red Grouper	N	Brought on Board (Retained/Discarded)	Live - Healthy	Retained	Not Applicable	
54	UCAM	Epinephelus morio	Red Grouper	N	Brought on Board (Retained/Discarded)	Live - Stomach and/or Eyes Protruding	Retained	Not Applicable	
54	UCAM	Epinephelus morio	Red Grouper	N	Brought on Board (Retained/Discarded)	Live - Stomach and/or Eyes Protruding	Retained	Not Applicable	
54	UCAM	Epinephelus morio	Red Grouper	N	Brought on Board (Retained/Discarded)	Live - Healthy	Discarded - Live and Healthy (Vented)	Not Applicable	
54	UCAM	Epinephelus morio	Red Grouper	N	Brought on Board (Retained/Discarded)	Live - Stomach and/or Eyes Protruding	Retained	Not Applicable	
54	UCAM	Epinephelus morio	Red Grouper	N	Brought on Board (Retained/Discarded)	Live - Healthy	Discarded - Live and Healthy (Vented)	Not Applicable	
54	UCAM	Epinephelus morio	Red Grouper	N	Brought on Board (Retained/Discarded)	Live - Healthy	Retained	Not Applicable	
54	UCAM	Epinephelus morio	Red Grouper	N	Brought on Board (Retained/Discarded)	Live - Stomach and/or Eyes Protruding	Retained	Not Applicable	
54	UCAM	Epinephelus morio	Red Grouper	N	Brought on Board (Retained/Discarded)	Live - Stomach and/or Eyes Protruding	Discarded - Live and Healthy (Vented)	Not Applicable	
54	UCAM	Epinephelus morio	Red Grouper	N	Brought on Board (Retained/Discarded)	Live - Healthy	Discarded - Live and Healthy (Vented)	Not Applicable	
54	UCAM	Epinephelus morio	Red Grouper	N	Brought on Board (Retained/Discarded)	Live - Healthy	Discarded - Live and Healthy (Vented)	Not Applicable	
54	UCAM	Galeocerdo cuvier	Tiger Shark	Y	Cut-off at Rail (No Entanglement)	Live - Healthy	Discarded - Live and Healthy (Not Vented)	Known Juvenile	Medium (Between 1m and 2m)
54	UCAM	Holocentrus adscensionis	Squirrelfish	N	Brought on Board (Retained/Discarded)	Live - Healthy	Discarded - Live and Healthy (Vented)	Not Applicable	
54	UCAM	Lutjanus analis	Mutton Snapper	N	Brought on Board (Retained/Discarded)	Live - Healthy	Retained	Not Applicable	
54	UCAM	Lutjanus analis	Mutton Snapper	N	Brought on Board (Retained/Discarded)	Live - Healthy	Retained	Not Applicable	
54	UCAM	Lutjanus campechanus	Red Snapper	N	Brought on Board (Retained/Discarded)	Live - Healthy	Discarded - Live and Healthy (Vented)	Not Applicable	
54	UCAM	Lutjanus campechanus	Red Snapper	N	Brought on Board (Retained/Discarded)	Live - Stomach and/or Eyes Protruding	Discarded - Live and Healthy (Vented)	Not Applicable	
54	UCAM	Lutjanus griseus	Gray (Mangrove) Snapper	N	Brought on Board (Retained/Discarded)	Live - Healthy	Retained	Not Applicable	
55	NOCAM	Epinephelus morio	Red Grouper	N	Brought on Board (Retained/Discarded)	Live - Healthy	Retained	Not Applicable	
55	NOCAM	Epinephelus morio	Red Grouper	N	Brought on Board (Retained/Discarded)	Live - Healthy	Retained	Not Applicable	
55	NOCAM	Epinephelus morio	Red Grouper	N	Brought on Board (Retained/Discarded)	Dead on Arrival - Damaged	Retained	Not Applicable	
55	NOCAM	Epinephelus morio	Red Grouper	N	Brought on Board (Retained/Discarded)	Live - Healthy	Retained	Not Applicable	
55	NOCAM	Epinephelus morio	Red Grouper	N	Brought on Board (Retained/Discarded)	Live - Healthy	Retained	Not Applicable	
55	NOCAM	Epinephelus morio	Red Grouper	N	Brought on Board (Retained/Discarded)	Live - Stomach and/or Eyes Protruding	Retained	Not Applicable	
55	NOCAM	Epinephelus morio	Red Grouper	N	Brought on Board (Retained/Discarded)	Live - Healthy	Retained	Not Applicable	
55	NOCAM	Epinephelus morio	Red Grouper	N	Brought on Board (Retained/Discarded)	Live - Healthy	Retained	Not Applicable	
55	NOCAM	Epinephelus morio	Red Grouper	N	Brought on Board (Retained/Discarded)	Live - Stomach and/or Eyes Protruding	Retained	Not Applicable	
55	NOCAM	Epinephelus morio	Red Grouper	N	Brought on Board (Retained/Discarded)	Live - Stomach and/or Eyes Protruding	Discarded - Live and Healthy (Vented)	Not Applicable	
55	NOCAM	Epinephelus morio	Red Grouper	N	Brought on Board (Retained/Discarded)	Live - Healthy	Retained	Not Applicable	
55	NOCAM	Epinephelus morio	Red Grouper	N	Brought on Board (Retained/Discarded)	Live - Stomach and/or Eyes Protruding	Retained	Not Applicable	
55	NOCAM	Epinephelus morio	Red Grouper	N	Brought on Board (Retained/Discarded)	Live - Healthy	Retained	Not Applicable	
55	NOCAM	Epinephelus morio	Red Grouper	N	Brought on Board (Retained/Discarded)	Live - Healthy	Retained	Not Applicable	
55	NOCAM	Epinephelus morio	Red Grouper	N	Brought on Board (Retained/Discarded)	Live - Healthy	Retained	Not Applicable	
55	NOCAM	Epinephelus morio	Red Grouper	N	Brought on Board (Retained/Discarded)	Live - Healthy	Retained	Not Applicable	
55	NOCAM	Epinephelus morio	Red Grouper	N	Brought on Board (Retained/Discarded)	Live - Stomach and/or Eyes Protruding	Retained	Not Applicable	
55	NOCAM	Epinephelus morio	Red Grouper	N	Brought on Board (Retained/Discarded)	Live - Healthy	Retained	Not Applicable	
55	NOCAM	Epinephelus morio	Red Grouper	N	Brought on Board (Retained/Discarded)	Live - Stomach and/or Eyes Protruding	Retained	Not Applicable	
55	NOCAM	Epinephelus morio	Red Grouper	N	Brought on Board (Retained/Discarded)	Live - Stomach and/or Eyes Protruding	Retained	Not Applicable	
55	NOCAM	Epinephelus morio	Red Grouper	N	Brought on Board (Retained/Discarded)	Live - Healthy	Retained	Not Applicable	
55	NOCAM	Epinephelus morio	Red Grouper	N	Brought on Board (Retained/Discarded)	Live - Healthy	Retained	Not Applicable	
55	NOCAM	Epinephelus morio	Red Grouper	N	Brought on Board (Retained/Discarded)	Live - Healthy	Retained	Not Applicable	
55	NOCAM	Epinephelus morio	Red Grouper	N	Brought on Board (Retained/Discarded)	Live - Healthy	Retained	Not Applicable	
55	NOCAM	Epinephelus morio	Red Grouper	N	Brought on Board (Retained/Discarded)	Live - Stomach and/or Eyes Protruding	Retained	Not Applicable	
55	NOCAM	Epinephelus morio	Red Grouper	N	Brought on Board (Retained/Discarded)	Live - Stomach and/or Eyes Protruding	Retained	Not Applicable	
55	NOCAM	Epinephelus morio	Red Grouper	N	Brought on Board (Retained/Discarded)	Live - Healthy	Retained	Not Applicable	
55	NOCAM	Epinephelus morio	Red Grouper	N	Brought on Board (Retained/Discarded)	Live - Healthy	Retained	Not Applicable	
55	NOCAM	Epinephelus morio	Red Grouper	N	Brought on Board (Retained/Discarded)	Live - Stomach and/or Eyes Protruding	Retained	Not Applicable	
55	NOCAM	Epinephelus morio	Red Grouper	N	Brought on Board (Retained/Discarded)	Live - Stomach and/or Eyes Protruding	Retained	Not Applicable	
55	NOCAM	Epinephelus morio	Red Grouper	N	Brought on Board (Retained/Discarded)	Live - Stomach and/or Eyes Protruding	Retained	Not Applicable	
55	NOCAM	Epinephelus morio	Red Grouper	N	Brought on Board (Retained/Discarded)	Live - Stomach and/or Eyes Protruding	Retained	Not Applicable	
55	NOCAM	Epinephelus morio	Red Grouper	N	Brought on Board (Retained/Discarded)	Live - Stomach and/or Eyes Protruding	Retained	Not Applicable	
55	NOCAM	Epinephelus morio	Red Grouper	N	Brought on Board (Retained/Discarded)	Live - Stomach and/or Eyes Protruding	Retained	Not Applicable	
55	NOCAM	Epinephelus morio	Red Grouper	N	Brought on Board (Retained/Discarded)	Live - Healthy	Retained	Not Applicable	
55	NOCAM	Epinephelus morio	Red Grouper	N	Brought on Board (Retained/Discarded)	Live - Stomach and/or Eyes Protruding	Retained	Not Applicable	
55	NOCAM	Galeocerdo cuvier	Tiger Shark	Y	Cut-off at Rail (No Entanglement)	Live - Healthy	Discarded - Live and Healthy (Not Vented)	Known Juvenile	Medium (Between 1m and 2m)
55	UCAM	Epinephelus morio	Red Grouper	N	Brought on Board (Retained/Discarded)	Live - Stomach and/or Eyes Protruding	Retained	Not Applicable	
55	UCAM	Epinephelus morio	Red Grouper	N	Brought on Board (Retained/Discarded)	Dead on Arrival - Damaged	Retained	Not Applicable	
55	UCAM	Epinephelus morio	Red Grouper	N	Brought on Board (Retained/Discarded)	Live - Stomach and/or Eyes Protruding	Retained	Not Applicable	
55	UCAM	Epinephelus morio	Red Grouper	N	Brought on Board (Retained/Discarded)	Live - Stomach and/or Eyes Protruding	Retained	Not Applicable	
55	UCAM	Epinephelus morio	Red Grouper	N	Brought on Board (Retained/Discarded)	Live - Healthy	Retained	Not Applicable	
55	UCAM	Epinephelus morio	Red Grouper	N	Brought on Board (Retained/Discarded)	Live - Healthy	Retained	Not Applicable	
55	UCAM	Epinephelus morio	Red Grouper	N	Brought on Board (Retained/Discarded)	Live - Healthy	Retained	Not Applicable	
55	UCAM	Epinephelus morio	Red Grouper	N	Brought on Board (Retained/Discarded)	Live - Stomach and/or Eyes Protruding	Retained	Not Applicable	
55	UCAM	Epinephelus morio	Red Grouper	N	Brought on Board (Retained/Discarded)	Live - Stomach and/or Eyes Protruding	Retained	Not Applicable	
55	UCAM	Epinephelus morio	Red Grouper	N	Brought on Board (Retained/Discarded)	Live - Stomach and/or Eyes Protruding	Retained	Not Applicable	
55	UCAM	Epinephelus morio	Red Grouper	N	Brought on Board (Retained/Discarded)	Live - Healthy	Retained	Not Applicable	
55	UCAM	Epinephelus morio	Red Grouper	N	Brought on Board (Retained/Discarded)	Live - Healthy	Retained	Not Applicable	

55	UCAM	Epinephelus morio	Red Grouper	N	Brought on Board (Retained/Discarded)	Live - Healthy	Retained	Not Applicable	
55	UCAM	Epinephelus morio	Red Grouper	N	Brought on Board (Retained/Discarded)	Live - Healthy	Retained	Not Applicable	
55	UCAM	Epinephelus morio	Red Grouper	N	Brought on Board (Retained/Discarded)	Live - Healthy	Retained	Not Applicable	
55	UCAM	Epinephelus morio	Red Grouper	N	Brought on Board (Retained/Discarded)	Live - Healthy	Retained	Not Applicable	
55	UCAM	Epinephelus morio	Red Grouper	N	Brought on Board (Retained/Discarded)	Live - Stomach and/or Eyes Protruding	Retained	Not Applicable	
55	UCAM	Epinephelus morio	Red Grouper	N	Brought on Board (Retained/Discarded)	Live - Stomach and/or Eyes Protruding	Retained	Not Applicable	
55	UCAM	Epinephelus morio	Red Grouper	N	Brought on Board (Retained/Discarded)	Live - Healthy	Retained	Not Applicable	
55	UCAM	Epinephelus morio	Red Grouper	N	Brought on Board (Retained/Discarded)	Live - Stomach and/or Eyes Protruding	Retained	Not Applicable	
55	UCAM	Epinephelus morio	Red Grouper	N	Brought on Board (Retained/Discarded)	Live - Healthy	Retained	Not Applicable	
55	UCAM	Epinephelus morio	Red Grouper	N	Brought on Board (Retained/Discarded)	Live - Healthy	Retained	Not Applicable	
55	UCAM	Epinephelus morio	Red Grouper	N	Brought on Board (Retained/Discarded)	Live - Healthy	Retained	Not Applicable	
55	UCAM	Epinephelus morio	Red Grouper	N	Brought on Board (Retained/Discarded)	Live - Healthy	Retained	Not Applicable	
55	UCAM	Epinephelus morio	Red Grouper	N	Brought on Board (Retained/Discarded)	Live - Stomach and/or Eyes Protruding	Retained	Not Applicable	
55	UCAM	Epinephelus morio	Red Grouper	N	Brought on Board (Retained/Discarded)	Live - Stomach and/or Eyes Protruding	Retained	Not Applicable	
55	UCAM	Epinephelus morio	Red Grouper	N	Brought on Board (Retained/Discarded)	Live - Healthy	Retained	Not Applicable	
55	UCAM	Epinephelus morio	Red Grouper	N	Brought on Board (Retained/Discarded)	Live - Stomach and/or Eyes Protruding	Discarded - Live and Healthy (Vented)	Not Applicable	
55	UCAM	Epinephelus morio	Red Grouper	N	Brought on Board (Retained/Discarded)	Live - Stomach and/or Eyes Protruding	Retained	Not Applicable	
55	UCAM	Epinephelus morio	Red Grouper	N	Brought on Board (Retained/Discarded)	Live - Stomach and/or Eyes Protruding	Retained	Not Applicable	
55	UCAM	Epinephelus morio	Red Grouper	N	Brought on Board (Retained/Discarded)	Live - Stomach and/or Eyes Protruding	Retained	Not Applicable	
55	UCAM	Epinephelus morio	Red Grouper	N	Brought on Board (Retained/Discarded)	Live - Healthy	Retained	Not Applicable	
55	UCAM	Epinephelus morio	Red Grouper	N	Brought on Board (Retained/Discarded)	Live - Stomach and/or Eyes Protruding	Retained	Not Applicable	
55	UCAM	Epinephelus morio	Red Grouper	N	Brought on Board (Retained/Discarded)	Live - Healthy	Retained	Not Applicable	
55	UCAM	Epinephelus morio	Red Grouper	N	Brought on Board (Retained/Discarded)	Live - Healthy	Retained	Not Applicable	
55	UCAM	Epinephelus morio	Red Grouper	N	Brought on Board (Retained/Discarded)	Live - Healthy	Retained	Not Applicable	
55	UCAM	Epinephelus morio	Red Grouper	N	Brought on Board (Retained/Discarded)	Live - Healthy	Retained	Not Applicable	
55	UCAM	Galeocerdo cuvier	Tiger Shark	Y	Cut-off at Rail (No Entanglement)	Live - Healthy	Discarded - Live and Healthy (Not Vented)	Known Juvenile	Medium (Between 1m and 2m)
56	NOCAM	Epinephelus morio	Red Grouper	N	Brought on Board (Retained/Discarded)	Live - Healthy	Retained	Not Applicable	
56	NOCAM	Epinephelus morio	Red Grouper	N	Brought on Board (Retained/Discarded)	Live - Healthy	Discarded - Live and Healthy (Vented)	Not Applicable	
56	NOCAM	Epinephelus morio	Red Grouper	N	Brought on Board (Retained/Discarded)	Live - Stomach and/or Eyes Protruding	Retained	Not Applicable	
56	NOCAM	Epinephelus morio	Red Grouper	N	Brought on Board (Retained/Discarded)	Live - Stomach and/or Eyes Protruding	Retained	Not Applicable	
56	NOCAM	Epinephelus morio	Red Grouper	N	Brought on Board (Retained/Discarded)	Live - Stomach and/or Eyes Protruding	Retained	Not Applicable	
56	NOCAM	Epinephelus morio	Red Grouper	N	Brought on Board (Retained/Discarded)	Live - Healthy	Retained	Not Applicable	
56	NOCAM	Epinephelus morio	Red Grouper	N	Brought on Board (Retained/Discarded)	Live - Stomach and/or Eyes Protruding	Retained	Not Applicable	
56	NOCAM	Epinephelus morio	Red Grouper	N	Brought on Board (Retained/Discarded)	Live - Stomach and/or Eyes Protruding	Retained	Not Applicable	
56	NOCAM	Epinephelus morio	Red Grouper	N	Brought on Board (Retained/Discarded)	Live - Healthy	Retained	Not Applicable	
56	NOCAM	Epinephelus morio	Red Grouper	N	Brought on Board (Retained/Discarded)	Live - Stomach and/or Eyes Protruding	Retained	Not Applicable	
56	NOCAM	Epinephelus morio	Red Grouper	N	Brought on Board (Retained/Discarded)	Live - Stomach and/or Eyes Protruding	Retained	Not Applicable	
56	NOCAM	Epinephelus morio	Red Grouper	N	Brought on Board (Retained/Discarded)	Live - Healthy	Discarded - Live and Healthy (Vented)	Not Applicable	
56	NOCAM	Epinephelus morio	Red Grouper	N	Brought on Board (Retained/Discarded)	Live - Stomach and/or Eyes Protruding	Retained	Not Applicable	
56	NOCAM	Epinephelus morio	Red Grouper	N	Brought on Board (Retained/Discarded)	Live - Healthy	Retained	Not Applicable	
56	NOCAM	Epinephelus morio	Red Grouper	N	Brought on Board (Retained/Discarded)	Live - Healthy	Retained	Not Applicable	
56	NOCAM	Epinephelus morio	Red Grouper	N	Brought on Board (Retained/Discarded)	Live - Healthy	Discarded - Live and Healthy (Vented)	Not Applicable	
56	NOCAM	Epinephelus morio	Red Grouper	N	Brought on Board (Retained/Discarded)	Live - Healthy	Retained	Not Applicable	
56	NOCAM	Epinephelus morio	Red Grouper	N	Brought on Board (Retained/Discarded)	Live - Stomach and/or Eyes Protruding	Retained	Not Applicable	
56	NOCAM	Epinephelus morio	Red Grouper	N	Brought on Board (Retained/Discarded)	Live - Stomach and/or Eyes Protruding	Retained	Not Applicable	
56	NOCAM	Epinephelus morio	Red Grouper	N	Brought on Board (Retained/Discarded)	Live - Healthy	Retained	Not Applicable	
56	NOCAM	Epinephelus morio	Red Grouper	N	Brought on Board (Retained/Discarded)	Live - Stomach and/or Eyes Protruding	Retained	Not Applicable	
56	NOCAM	Epinephelus morio	Red Grouper	N	Brought on Board (Retained/Discarded)	Live - Stomach and/or Eyes Protruding	Retained	Not Applicable	
56	NOCAM	Epinephelus morio	Red Grouper	N	Brought on Board (Retained/Discarded)	Live - Healthy	Retained	Not Applicable	
56	NOCAM	Epinephelus morio	Red Grouper	N	Brought on Board (Retained/Discarded)	Live - Stomach and/or Eyes Protruding	Retained	Not Applicable	
56	NOCAM	Epinephelus morio	Red Grouper	N	Brought on Board (Retained/Discarded)	Live - Stomach and/or Eyes Protruding	Retained	Not Applicable	
56	NOCAM	Epinephelus morio	Red Grouper	N	Brought on Board (Retained/Discarded)	Live - Stomach and/or Eyes Protruding	Retained	Not Applicable	
56	NOCAM	Epinephelus morio	Red Grouper	N	Brought on Board (Retained/Discarded)	Live - Healthy	Retained	Not Applicable	
56	NOCAM	Epinephelus morio	Red Grouper	N	Brought on Board (Retained/Discarded)	Live - Healthy	Retained	Not Applicable	
56	NOCAM	Epinephelus morio	Red Grouper	N	Brought on Board (Retained/Discarded)	Live - Healthy	Retained	Not Applicable	
56	NOCAM	Epinephelus morio	Red Grouper	N	Brought on Board (Retained/Discarded)	Live - Stomach and/or Eyes Protruding	Discarded - Live and Healthy (Vented)	Not Applicable	
56	NOCAM	Epinephelus morio	Red Grouper	N	Brought on Board (Retained/Discarded)	Live - Stomach and/or Eyes Protruding	Discarded - Live and Healthy (Vented)	Not Applicable	
56	NOCAM	Epinephelus morio	Red Grouper	N	Brought on Board (Retained/Discarded)	Live - Healthy	Retained	Not Applicable	
56	NOCAM	Epinephelus morio	Red Grouper	N	Brought on Board (Retained/Discarded)	Live - Stomach and/or Eyes Protruding	Discarded - Live and Healthy (Vented)	Not Applicable	
56	NOCAM	Epinephelus morio	Red Grouper	N	Brought on Board (Retained/Discarded)	Live - Stomach and/or Eyes Protruding	Retained	Not Applicable	
56	NOCAM	Epinephelus morio	Red Grouper	N	Brought on Board (Retained/Discarded)	Live - Healthy	Discarded - Live and Healthy (Vented)	Not Applicable	
56	NOCAM	Epinephelus morio	Red Grouper	N	Brought on Board (Retained/Discarded)	Live - Healthy	Retained	Not Applicable	
56	NOCAM	Epinephelus morio	Red Grouper	N	Brought on Board (Retained/Discarded)	Live - Healthy	Discarded - Live and Healthy (Vented)	Not Applicable	
56	NOCAM	Epinephelus morio	Red Grouper	N	Brought on Board (Retained/Discarded)	Live - Healthy	Retained	Not Applicable	
56	NOCAM	Epinephelus morio	Red Grouper	N	Brought on Board (Retained/Discarded)	Live - Healthy	Retained	Not Applicable	
56	NOCAM	Epinephelus morio	Red Grouper	N	Brought on Board (Retained/Discarded)	Live - Stomach and/or Eyes Protruding	Retained	Not Applicable	
56	NOCAM	Epinephelus morio	Red Grouper	N	Brought on Board (Retained/Discarded)	Live - Healthy	Retained	Not Applicable	
56	NOCAM	Epinephelus morio	Red Grouper	N	Brought on Board (Retained/Discarded)	Live - Stomach and/or Eyes Protruding	Retained	Not Applicable	
56	NOCAM	Epinephelus morio	Red Grouper	N	Brought on Board (Retained/Discarded)	Live - Stomach and/or Eyes Protruding	Retained	Not Applicable	
56	NOCAM	Epinephelus morio	Red Grouper	N	Brought on Board (Retained/Discarded)	Live - Stomach and/or Eyes Protruding	Retained	Not Applicable	
56	NOCAM	Epinephelus morio	Red Grouper	N	Brought on Board (Retained/Discarded)	Live - Healthy	Retained	Not Applicable	
56	NOCAM	Epinephelus morio	Red Grouper	N	Brought on Board (Retained/Discarded)	Live - Stomach and/or Eyes Protruding	Retained	Not Applicable	
56	NOCAM	Epinephelus morio	Red Grouper	N	Brought on Board (Retained/Discarded)	Live - Stomach and/or Eyes Protruding	Retained	Not Applicable	
56	NOCAM	Epinephelus morio	Red Grouper	N	Brought on Board (Retained/Discarded)	Live - Healthy	Retained	Not Applicable	
56	NOCAM	Epinephelus morio	Red Grouper	N	Brought on Board (Retained/Discarded)	Live - Healthy	Retained	Not Applicable	
56	NOCAM	Epinephelus morio	Red Grouper	N	Brought on Board (Retained/Discarded)	Live - Stomach and/or Eyes Protruding	Retained	Not Applicable	
56	NOCAM	Epinephelus morio	Red Grouper	N	Brought on Board (Retained/Discarded)	Live - Stomach and/or Eyes Protruding	Retained	Not Applicable	
56	NOCAM	Epinephelus morio	Red Grouper	N	Brought on Board (Retained/Discarded)	Live - Stomach and/or Eyes Protruding	Retained	Not Applicable	
56	NOCAM	Epinephelus morio	Red Grouper	N	Brought on Board (Retained/Discarded)	Live - Stomach and/or Eyes Protruding	Retained	Not Applicable	
56	NOCAM	Epinephelus morio	Red Grouper	N	Brought on Board (Retained/Discarded)	Live - Healthy	Retained	Not Applicable	
56	NOCAM	Epinephelus morio	Red Grouper	N	Brought on Board (Retained/Discarded)	Live - Healthy	Retained	Not Applicable	
56	NOCAM	Lutjanus analis	Mutton Snapper	N	Brought on Board (Retained/Discarded)	Live - Healthy	Retained	Not Applicable	
56	NOCAM	Lutjanus analis	Mutton Snapper	N	Brought on Board (Retained/Discarded)	Live - Healthy	Retained	Not Applicable	
56	NOCAM	Lutjanus analis	Mutton Snapper	N	Brought on Board (Retained/Discarded)	Live - Healthy	Retained	Not Applicable	
56	NOCAM	Lutjanus campechanus	Red Snapper	N	Brought on Board (Retained/Discarded)	Live - Healthy	Discarded - Live and Healthy (Vented)	Not Applicable	
56	NOCAM	Lutjanus campechanus	Red Snapper	N	Brought on Board (Retained/Discarded)	Live - Healthy	Discarded - Live and Healthy (Vented)	Not Applicable	
56	NOCAM	Lutjanus campechanus	Red Snapper	N	Brought on Board (Retained/Discarded)	Live - Stomach and/or Eyes Protruding	Discarded - Live and Healthy (Vented)	Not Applicable	
56	NOCAM	Lutjanus campechanus	Red Snapper	N	Brought on Board (Retained/Discarded)	Live - Healthy	Discarded - Live and Healthy (Vented)	Not Applicable	
56	NOCAM	Lutjanus campechanus	Red Snapper	N	Brought on Board (Retained/Discarded)	Live - Healthy	Discarded - Live and Healthy (Vented)	Not Applicable	
56	NOCAM	Lutjanus campechanus	Red Snapper	N	Brought on Board (Retained/Discarded)	Live - Healthy	Discarded - Live and Healthy (Vented)	Not Applicable	
56	NOCAM	Lutjanus campechanus	Red Snapper	N	Brought on Board (Retained/Discarded)	Live - Healthy	Discarded - Live and Healthy (Vented)	Not Applicable	
56	NOCAM	Lutjanus campechanus	Red Snapper	N	Brought on Board (Retained/Discarded)	Dead on Arrival - Undamaged	Discarded - Dead	Not Applicable	

56	NOCAM	Lutjanus campechanus	Red Snapper	N	Brought on Board (Retained/Discarded)	Live - Healthy	Discarded - Live and Healthy (Vented)	Not Applicable	
56	NOCAM	Mycteroperca phenax	Scamp	N	Brought on Board (Retained/Discarded)	Live - Stomach and/or Eyes Protruding	Retained	Not Applicable	
56	NOCAM	Rhizoprionodon terraenovae	Atlantic Sharpnose Shark	Y	Brought on Board (Retained/Discarded)	Live - Healthy	Discarded - Live and Healthy (Not Vented)	Female	Small (Less than 1m)
56	UCAM	Epinephelinae, unidentified	Grouper, Unidentified	N	Not Handled (Dropped Off)	Live - Healthy	Discarded - Live and Healthy (Not Vented)	Not Applicable	
56	UCAM	Epinephelus morio	Red Grouper	N	Brought on Board (Retained/Discarded)	Live - Stomach and/or Eyes Protruding	Retained	Not Applicable	
56	UCAM	Epinephelus morio	Red Grouper	N	Brought on Board (Retained/Discarded)	Live - Stomach and/or Eyes Protruding	Retained	Not Applicable	
56	UCAM	Epinephelus morio	Red Grouper	N	Brought on Board (Retained/Discarded)	Live - Stomach and/or Eyes Protruding	Retained	Not Applicable	
56	UCAM	Epinephelus morio	Red Grouper	N	Brought on Board (Retained/Discarded)	Live - Healthy	Retained	Not Applicable	
56	UCAM	Epinephelus morio	Red Grouper	N	Brought on Board (Retained/Discarded)	Live - Healthy	Retained	Not Applicable	
56	UCAM	Epinephelus morio	Red Grouper	N	Brought on Board (Retained/Discarded)	Live - Stomach and/or Eyes Protruding	Retained	Not Applicable	
56	UCAM	Epinephelus morio	Red Grouper	N	Brought on Board (Retained/Discarded)	Live - Stomach and/or Eyes Protruding	Retained	Not Applicable	
56	UCAM	Epinephelus morio	Red Grouper	N	Brought on Board (Retained/Discarded)	Live - Stomach and/or Eyes Protruding	Retained	Not Applicable	
56	UCAM	Epinephelus morio	Red Grouper	N	Brought on Board (Retained/Discarded)	Live - Healthy	Retained	Not Applicable	
56	UCAM	Epinephelus morio	Red Grouper	N	Brought on Board (Retained/Discarded)	Live - Healthy	Retained	Not Applicable	
56	UCAM	Epinephelus morio	Red Grouper	N	Brought on Board (Retained/Discarded)	Live - Healthy	Discarded - Live and Healthy (Vented)	Not Applicable	
56	UCAM	Epinephelus morio	Red Grouper	N	Brought on Board (Retained/Discarded)	Live - Stomach and/or Eyes Protruding	Retained	Not Applicable	
56	UCAM	Epinephelus morio	Red Grouper	N	Brought on Board (Retained/Discarded)	Live - Healthy	Retained	Not Applicable	
56	UCAM	Epinephelus morio	Red Grouper	N	Brought on Board (Retained/Discarded)	Live - Healthy	Retained	Not Applicable	
56	UCAM	Epinephelus morio	Red Grouper	N	Brought on Board (Retained/Discarded)	Live - Healthy	Retained	Not Applicable	
56	UCAM	Epinephelus morio	Red Grouper	N	Brought on Board (Retained/Discarded)	Live - Healthy	Discarded - Live and Healthy (Not Vented)	Not Applicable	
56	UCAM	Epinephelus morio	Red Grouper	N	Brought on Board (Retained/Discarded)	Live - Healthy	Retained	Not Applicable	
56	UCAM	Epinephelus morio	Red Grouper	N	Brought on Board (Retained/Discarded)	Live - Healthy	Retained	Not Applicable	
56	UCAM	Epinephelus morio	Red Grouper	N	Brought on Board (Retained/Discarded)	Live - Stomach and/or Eyes Protruding	Retained	Not Applicable	
56	UCAM	Epinephelus morio	Red Grouper	N	Brought on Board (Retained/Discarded)	Live - Stomach and/or Eyes Protruding	Retained	Not Applicable	
56	UCAM	Epinephelus morio	Red Grouper	N	Brought on Board (Retained/Discarded)	Live - Healthy	Discarded - Live and Healthy (Vented)	Not Applicable	
56	UCAM	Epinephelus morio	Red Grouper	N	Brought on Board (Retained/Discarded)	Live - Healthy	Retained	Not Applicable	
56	UCAM	Epinephelus morio	Red Grouper	N	Brought on Board (Retained/Discarded)	Live - Healthy	Retained	Not Applicable	
56	UCAM	Epinephelus morio	Red Grouper	N	Brought on Board (Retained/Discarded)	Live - Healthy	Discarded - Live and Healthy (Vented)	Not Applicable	
56	UCAM	Epinephelus morio	Red Grouper	N	Brought on Board (Retained/Discarded)	Live - Healthy	Retained	Not Applicable	
56	UCAM	Epinephelus morio	Red Grouper	N	Brought on Board (Retained/Discarded)	Live - Stomach and/or Eyes Protruding	Retained	Not Applicable	
56	UCAM	Epinephelus morio	Red Grouper	N	Brought on Board (Retained/Discarded)	Live - Stomach and/or Eyes Protruding	Retained	Not Applicable	
56	UCAM	Epinephelus morio	Red Grouper	N	Brought on Board (Retained/Discarded)	Live - Healthy	Retained	Not Applicable	
56	UCAM	Epinephelus morio	Red Grouper	N	Brought on Board (Retained/Discarded)	Live - Healthy	Discarded - Live and Healthy (Vented)	Not Applicable	
56	UCAM	Epinephelus morio	Red Grouper	N	Brought on Board (Retained/Discarded)	Live - Stomach and/or Eyes Protruding	Retained	Not Applicable	
56	UCAM	Epinephelus morio	Red Grouper	N	Brought on Board (Retained/Discarded)	Live - Stomach and/or Eyes Protruding	Retained	Not Applicable	
56	UCAM	Epinephelus morio	Red Grouper	N	Brought on Board (Retained/Discarded)	Live - Healthy	Discarded - Live and Healthy (Not Vented)	Not Applicable	
56	UCAM	Epinephelus morio	Red Grouper	N	Brought on Board (Retained/Discarded)	Live - Healthy	Retained	Not Applicable	
56	UCAM	Epinephelus morio	Red Grouper	N	Brought on Board (Retained/Discarded)	Live - Stomach and/or Eyes Protruding	Retained	Not Applicable	
56	UCAM	Epinephelus morio	Red Grouper	N	Brought on Board (Retained/Discarded)	Live - Healthy	Retained	Not Applicable	
56	UCAM	Epinephelus morio	Red Grouper	N	Brought on Board (Retained/Discarded)	Live - Stomach and/or Eyes Protruding	Discarded - Live and Healthy (Vented)	Not Applicable	
56	UCAM	Epinephelus morio	Red Grouper	N	Brought on Board (Retained/Discarded)	Live - Healthy	Retained	Not Applicable	
56	UCAM	Epinephelus morio	Red Grouper	N	Brought on Board (Retained/Discarded)	Dead on Arrival - Undamaged	Retained	Not Applicable	
56	UCAM	Epinephelus morio	Red Grouper	N	Brought on Board (Retained/Discarded)	Live - Healthy	Retained	Not Applicable	
56	UCAM	Epinephelus morio	Red Grouper	N	Brought on Board (Retained/Discarded)	Live - Stomach and/or Eyes Protruding	Retained	Not Applicable	
56	UCAM	Epinephelus morio	Red Grouper	N	Brought on Board (Retained/Discarded)	Live - Healthy	Discarded - Live and Healthy (Vented)	Not Applicable	
56	UCAM	Epinephelus morio	Red Grouper	N	Brought on Board (Retained/Discarded)	Live - Stomach and/or Eyes Protruding	Retained	Not Applicable	
56	UCAM	Epinephelus morio	Red Grouper	N	Brought on Board (Retained/Discarded)	Live - Healthy	Discarded - Live and Healthy (Vented)	Not Applicable	
56	UCAM	Epinephelus morio	Red Grouper	N	Brought on Board (Retained/Discarded)	Live - Healthy	Retained	Not Applicable	
56	UCAM	Epinephelus morio	Red Grouper	N	Brought on Board (Retained/Discarded)	Live - Healthy	Retained	Not Applicable	
56	UCAM	Epinephelus morio	Red Grouper	N	Brought on Board (Retained/Discarded)	Live - Stomach and/or Eyes Protruding	Retained	Not Applicable	
56	UCAM	Epinephelus morio	Red Grouper	N	Brought on Board (Retained/Discarded)	Live - Stomach and/or Eyes Protruding	Retained	Not Applicable	
56	UCAM	Epinephelus morio	Red Grouper	N	Brought on Board (Retained/Discarded)	Live - Stomach and/or Eyes Protruding	Retained	Not Applicable	
56	UCAM	Epinephelus morio	Red Grouper	N	Brought on Board (Retained/Discarded)	Live - Stomach and/or Eyes Protruding	Retained	Not Applicable	
56	UCAM	Epinephelus morio	Red Grouper	N	Brought on Board (Retained/Discarded)	Live - Stomach and/or Eyes Protruding	Retained	Not Applicable	
56	UCAM	Epinephelus morio	Red Grouper	N	Brought on Board (Retained/Discarded)	Live - Healthy	Retained	Not Applicable	
56	UCAM	Epinephelus morio	Red Grouper	N	Brought on Board (Retained/Discarded)	Live - Stomach and/or Eyes Protruding	Retained	Not Applicable	
56	UCAM	Epinephelus morio	Red Grouper	N	Brought on Board (Retained/Discarded)	Live - Healthy	Retained	Not Applicable	
56	UCAM	Epinephelus morio	Red Grouper	N	Brought on Board (Retained/Discarded)	Live - Healthy	Retained	Not Applicable	
56	UCAM	Epinephelus morio	Red Grouper	N	Brought on Board (Retained/Discarded)	Live - Healthy	Retained	Not Applicable	
56	UCAM	Epinephelus morio	Red Grouper	N	Brought on Board (Retained/Discarded)	Live - Stomach and/or Eyes Protruding	Retained	Not Applicable	
56	UCAM	Lutjanus analis	Mutton Snapper	N	Brought on Board (Retained/Discarded)	Live - Healthy	Retained	Not Applicable	
56	UCAM	Lutjanus analis	Mutton Snapper	N	Brought on Board (Retained/Discarded)	Live - Healthy	Retained	Not Applicable	
56	UCAM	Lutjanus analis	Mutton Snapper	N	Brought on Board (Retained/Discarded)	Live - Healthy	Retained	Not Applicable	
56	UCAM	Lutjanus campechanus	Red Snapper	N	Brought on Board (Retained/Discarded)	Live - Healthy	Discarded - Live and Healthy (Vented)	Not Applicable	
56	UCAM	Lutjanus campechanus	Red Snapper	N	Brought on Board (Retained/Discarded)	Live - Healthy	Discarded - Live and Healthy (Vented)	Not Applicable	
56	UCAM	Lutjanus campechanus	Red Snapper	N	Brought on Board (Retained/Discarded)	Live - Healthy	Discarded - Live and Healthy (Vented)	Not Applicable	
56	UCAM	Lutjanus campechanus	Red Snapper	N	Brought on Board (Retained/Discarded)	Dead on Arrival - Undamaged	Discarded - Dead	Not Applicable	
56	UCAM	Lutjanus campechanus	Red Snapper	N	Brought on Board (Retained/Discarded)	Live - Healthy	Discarded - Live and Healthy (Vented)	Not Applicable	
56	UCAM	Lutjanus campechanus	Red Snapper	N	Brought on Board (Retained/Discarded)	Dead on Arrival - Undamaged	Discarded - Dead	Not Applicable	
56	UCAM	Lutjanus campechanus	Red Snapper	N	Brought on Board (Retained/Discarded)	Live - Healthy	Discarded - Live and Healthy (Vented)	Not Applicable	
56	UCAM	Lutjanus campechanus	Red Snapper	N	Brought on Board (Retained/Discarded)	Live - Healthy	Discarded - Live and Healthy (Vented)	Not Applicable	
56	UCAM	Lutjanus campechanus	Red Snapper	N	Brought on Board (Retained/Discarded)	Live - Healthy	Discarded - Live and Healthy (Vented)	Not Applicable	
56	UCAM	Mycteroperca phenax	Scamp	N	Brought on Board (Retained/Discarded)	Live - Healthy	Retained	Not Applicable	
56	UCAM	Rhizoprionodon terraenovae	Atlantic Sharpnose Shark	Y	Brought on Board (Retained/Discarded)	Live - Healthy	Discarded - Live and Healthy (Not Vented)	Female	Small (Less than 1m)
57	NOCAM	Carcharhinus limbatus	Blacktip Shark	Y	Cut-off at Rail (No Entanglement)	Live - Healthy	Discarded - Live and Healthy (Not Vented)	Unknown Maturity and/or Sex	Medium (Between 1m and 2m)
57	NOCAM	Epinephelus itajara	Goliath Grouper	N	Cut-off at Rail (No Entanglement)	Live - Stomach and/or Eyes Protruding	Discarded - Live and Healthy (Not Vented)	Not Applicable	
57	NOCAM	Epinephelus morio	Red Grouper	N	Brought on Board (Retained/Discarded)	Live - Healthy	Retained	Not Applicable	
57	NOCAM	Epinephelus morio	Red Grouper	N	Brought on Board (Retained/Discarded)	Live - Stomach and/or Eyes Protruding	Retained	Not Applicable	
57	NOCAM	Epinephelus morio	Red Grouper	N	Brought on Board (Retained/Discarded)	Live - Stomach and/or Eyes Protruding	Retained	Not Applicable	
57	NOCAM	Epinephelus morio	Red Grouper	N	Brought on Board (Retained/Discarded)	Live - Stomach and/or Eyes Protruding	Discarded - Live and Healthy (Vented)	Not Applicable	
57	NOCAM	Epinephelus morio	Red Grouper	N	Brought on Board (Retained/Discarded)	Live - Stomach and/or Eyes Protruding	Retained	Not Applicable	
57	NOCAM	Epinephelus morio	Red Grouper	N	Brought on Board (Retained/Discarded)	Live - Healthy	Retained	Not Applicable	
57	NOCAM	Epinephelus morio	Red Grouper	N	Brought on Board (Retained/Discarded)	Live - Healthy	Retained	Not Applicable	
57	NOCAM	Epinephelus morio	Red Grouper	N	Brought on Board (Retained/Discarded)	Live - Stomach and/or Eyes Protruding	Retained	Not Applicable	
57	NOCAM	Epinephelus morio	Red Grouper	N	Brought on Board (Retained/Discarded)	Live - Stomach and/or Eyes Protruding	Retained	Not Applicable	
57	NOCAM	Epinephelus morio	Red Grouper	N	Brought on Board (Retained/Discarded)	Live - Healthy	Discarded - Live and Healthy (Vented)	Not Applicable	
57	NOCAM	Epinephelus morio	Red Grouper	N	Brought on Board (Retained/Discarded)	Live - Healthy	Retained	Not Applicable	
57	NOCAM	Epinephelus morio	Red Grouper	N	Brought on Board (Retained/Discarded)	Live - Stomach and/or Eyes Protruding	Retained	Not Applicable	
57	NOCAM	Epinephelus morio	Red Grouper	N	Brought on Board (Retained/Discarded)	Live - Healthy	Discarded - Live and Healthy (Vented)	Not Applicable	
57	NOCAM	Epinephelus morio	Red Grouper	N	Brought on Board (Retained/Discarded)	Live - Healthy	Retained	Not Applicable	
57	NOCAM	Epinephelus morio	Red Grouper	N	Brought on Board (Retained/Discarded)	Live - Stomach and/or Eyes Protruding	Discarded - Live and Healthy (Vented)	Not Applicable	
57	NOCAM	Epinephelus morio	Red Grouper	N	Brought on Board (Retained/Discarded)	Live - Healthy	Discarded - Live and Healthy (Vented)	Not Applicable	

57	NOCAM	Epinephelus morio	Red Grouper	N	Brought on Board (Retained/Discarded)	Live - Stomach and/or Eyes Protruding	Retained	Not Applicable	
57	NOCAM	Epinephelus morio	Red Grouper	N	Brought on Board (Retained/Discarded)	Live - Stomach and/or Eyes Protruding	Retained	Not Applicable	
57	NOCAM	Epinephelus morio	Red Grouper	N	Brought on Board (Retained/Discarded)	Live - Stomach and/or Eyes Protruding	Retained	Not Applicable	
57	NOCAM	Epinephelus morio	Red Grouper	N	Brought on Board (Retained/Discarded)	Live - Stomach and/or Eyes Protruding	Retained	Not Applicable	
57	NOCAM	Epinephelus morio	Red Grouper	N	Brought on Board (Retained/Discarded)	Live - Stomach and/or Eyes Protruding	Retained	Not Applicable	
57	NOCAM	Epinephelus morio	Red Grouper	N	Brought on Board (Retained/Discarded)	Live - Healthy	Retained	Not Applicable	
57	NOCAM	Epinephelus morio	Red Grouper	N	Brought on Board (Retained/Discarded)	Live - Healthy	Discarded - Live and Healthy (Vented)	Not Applicable	
57	NOCAM	Epinephelus morio	Red Grouper	N	Brought on Board (Retained/Discarded)	Live - Healthy	Discarded - Live and Healthy (Vented)	Not Applicable	
57	NOCAM	Epinephelus morio	Red Grouper	N	Brought on Board (Retained/Discarded)	Live - Stomach and/or Eyes Protruding	Retained	Not Applicable	
57	NOCAM	Epinephelus morio	Red Grouper	N	Brought on Board (Retained/Discarded)	Dead on Arrival - Undamaged	Retained	Not Applicable	
57	NOCAM	Epinephelus morio	Red Grouper	N	Brought on Board (Retained/Discarded)	Live - Healthy	Discarded - Live and Healthy (Vented)	Not Applicable	
57	NOCAM	Epinephelus morio	Red Grouper	N	Brought on Board (Retained/Discarded)	Live - Healthy	Discarded - Live and Healthy (Vented)	Not Applicable	
57	NOCAM	Epinephelus morio	Red Grouper	N	Brought on Board (Retained/Discarded)	Live - Healthy	Retained	Not Applicable	
57	NOCAM	Epinephelus morio	Red Grouper	N	Brought on Board (Retained/Discarded)	Live - Healthy	Retained	Not Applicable	
57	NOCAM	Epinephelus morio	Red Grouper	N	Brought on Board (Retained/Discarded)	Live - Stomach and/or Eyes Protruding	Retained	Not Applicable	
57	NOCAM	Epinephelus morio	Red Grouper	N	Brought on Board (Retained/Discarded)	Live - Healthy	Retained	Not Applicable	
57	NOCAM	Epinephelus morio	Red Grouper	N	Brought on Board (Retained/Discarded)	Live - Stomach and/or Eyes Protruding	Discarded - Live and Healthy (Vented)	Not Applicable	
57	NOCAM	Epinephelus morio	Red Grouper	N	Brought on Board (Retained/Discarded)	Live - Healthy	Retained	Not Applicable	
57	NOCAM	Epinephelus morio	Red Grouper	N	Brought on Board (Retained/Discarded)	Live - Healthy	Discarded - Live and Healthy (Vented)	Not Applicable	
57	NOCAM	Epinephelus morio	Red Grouper	N	Brought on Board (Retained/Discarded)	Live - Stomach and/or Eyes Protruding	Retained	Not Applicable	
57	NOCAM	Epinephelus morio	Red Grouper	N	Brought on Board (Retained/Discarded)	Live - Healthy	Discarded - Live and Healthy (Vented)	Not Applicable	
57	NOCAM	Epinephelus morio	Red Grouper	N	Brought on Board (Retained/Discarded)	Live - Stomach and/or Eyes Protruding	Retained	Not Applicable	
57	NOCAM	Epinephelus morio	Red Grouper	N	Brought on Board (Retained/Discarded)	Live - Stomach and/or Eyes Protruding	Retained	Not Applicable	
57	NOCAM	Lutjanus campechanus	Red Snapper	N	Brought on Board (Retained/Discarded)	Live - Stomach and/or Eyes Protruding	Discarded - Live and Healthy (Vented)	Not Applicable	
57	NOCAM	Lutjanus campechanus	Red Snapper	N	Brought on Board (Retained/Discarded)	Live - Stomach and/or Eyes Protruding	Discarded - Live and Healthy (Vented)	Not Applicable	
57	NOCAM	Lutjanus synagris	Lane Snapper	N	Brought on Board (Retained/Discarded)	Live - Healthy	Discarded - Live and Healthy (Not Vented)	Not Applicable	
57	NOCAM	Muraenidae, unidentified	Moray Eel, Unidentified	N	Brought on Board (Retained/Discarded)	Live - Healthy	Retained as Bait	Not Applicable	
57	UCAM	Epinephelus itajara	Goliath Grouper	N	Cut-off at Rail (No Entanglement)	Live - Stomach and/or Eyes Protruding	Discarded - Live and Healthy (Not Vented)	Not Applicable	
57	UCAM	Epinephelus morio	Red Grouper	N	Brought on Board (Retained/Discarded)	Live - Healthy	Retained	Not Applicable	
57	UCAM	Epinephelus morio	Red Grouper	N	Brought on Board (Retained/Discarded)	Live - Healthy	Discarded - Live and Healthy (Vented)	Not Applicable	
57	UCAM	Epinephelus morio	Red Grouper	N	Brought on Board (Retained/Discarded)	Live - Healthy	Retained	Not Applicable	
57	UCAM	Epinephelus morio	Red Grouper	N	Brought on Board (Retained/Discarded)	Live - Stomach and/or Eyes Protruding	Retained	Not Applicable	
57	UCAM	Epinephelus morio	Red Grouper	N	Brought on Board (Retained/Discarded)	Live - Healthy	Retained	Not Applicable	
57	UCAM	Epinephelus morio	Red Grouper	N	Brought on Board (Retained/Discarded)	Live - Healthy	Retained	Not Applicable	
57	UCAM	Epinephelus morio	Red Grouper	N	Brought on Board (Retained/Discarded)	Live - Stomach and/or Eyes Protruding	Discarded - Live and Healthy (Vented)	Not Applicable	
57	UCAM	Epinephelus morio	Red Grouper	N	Brought on Board (Retained/Discarded)	Live - Healthy	Retained	Not Applicable	
57	UCAM	Epinephelus morio	Red Grouper	N	Brought on Board (Retained/Discarded)	Live - Stomach and/or Eyes Protruding	Retained	Not Applicable	
57	UCAM	Epinephelus morio	Red Grouper	N	Brought on Board (Retained/Discarded)	Live - Healthy	Retained	Not Applicable	
57	UCAM	Epinephelus morio	Red Grouper	N	Brought on Board (Retained/Discarded)	Live - Healthy	Retained	Not Applicable	
57	UCAM	Epinephelus morio	Red Grouper	N	Brought on Board (Retained/Discarded)	Live - Healthy	Retained	Not Applicable	
57	UCAM	Epinephelus morio	Red Grouper	N	Brought on Board (Retained/Discarded)	Live - Healthy	Discarded - Live and Healthy (Vented)	Not Applicable	
57	UCAM	Epinephelus morio	Red Grouper	N	Brought on Board (Retained/Discarded)	Live - Stomach and/or Eyes Protruding	Retained	Not Applicable	
57	UCAM	Epinephelus morio	Red Grouper	N	Brought on Board (Retained/Discarded)	Live - Stomach and/or Eyes Protruding	Retained	Not Applicable	
57	UCAM	Epinephelus morio	Red Grouper	N	Brought on Board (Retained/Discarded)	Live - Stomach and/or Eyes Protruding	Retained	Not Applicable	
57	UCAM	Epinephelus morio	Red Grouper	N	Brought on Board (Retained/Discarded)	Live - Healthy	Retained	Not Applicable	
57	UCAM	Epinephelus morio	Red Grouper	N	Brought on Board (Retained/Discarded)	Live - Healthy	Retained	Not Applicable	
57	UCAM	Epinephelus morio	Red Grouper	N	Brought on Board (Retained/Discarded)	Live - Stomach and/or Eyes Protruding	Retained	Not Applicable	
57	UCAM	Epinephelus morio	Red Grouper	N	Brought on Board (Retained/Discarded)	Live - Healthy	Retained	Not Applicable	
57	UCAM	Epinephelus morio	Red Grouper	N	Brought on Board (Retained/Discarded)	Live - Healthy	Discarded - Live and Healthy (Vented)	Not Applicable	
57	UCAM	Epinephelus morio	Red Grouper	N	Brought on Board (Retained/Discarded)	Live - Healthy	Discarded - Live and Healthy (Vented)	Not Applicable	
57	UCAM	Epinephelus morio	Red Grouper	N	Brought on Board (Retained/Discarded)	Live - Healthy	Retained	Not Applicable	
57	UCAM	Epinephelus morio	Red Grouper	N	Brought on Board (Retained/Discarded)	Live - Healthy	Discarded - Live and Healthy (Vented)	Not Applicable	
57	UCAM	Epinephelus morio	Red Grouper	N	Brought on Board (Retained/Discarded)	Live - Healthy	Retained	Not Applicable	
57	UCAM	Epinephelus morio	Red Grouper	N	Brought on Board (Retained/Discarded)	Live - Healthy	Retained	Not Applicable	
57	UCAM	Epinephelus morio	Red Grouper	N	Brought on Board (Retained/Discarded)	Live - Healthy	Discarded - Live and Healthy (Vented)	Not Applicable	
57	UCAM	Epinephelus morio	Red Grouper	N	Brought on Board (Retained/Discarded)	Live - Stomach and/or Eyes Protruding	Discarded - Live and Healthy (Vented)	Not Applicable	
57	UCAM	Epinephelus morio	Red Grouper	N	Brought on Board (Retained/Discarded)	Live - Healthy	Retained	Not Applicable	
57	UCAM	Epinephelus morio	Red Grouper	N	Brought on Board (Retained/Discarded)	Live - Healthy	Discarded - Live and Healthy (Vented)	Not Applicable	
57	UCAM	Epinephelus morio	Red Grouper	N	Brought on Board (Retained/Discarded)	Live - Stomach and/or Eyes Protruding	Retained	Not Applicable	
57	UCAM	Epinephelus morio	Red Grouper	N	Brought on Board (Retained/Discarded)	Live - Healthy	Discarded - Live and Healthy (Vented)	Not Applicable	
57	UCAM	Epinephelus morio	Red Grouper	N	Brought on Board (Retained/Discarded)	Live - Healthy	Retained	Not Applicable	
57	UCAM	Epinephelus morio	Red Grouper	N	Brought on Board (Retained/Discarded)	Live - Stomach and/or Eyes Protruding	Retained	Not Applicable	
57	UCAM	Epinephelus morio	Red Grouper	N	Brought on Board (Retained/Discarded)	Live - Healthy	Retained	Not Applicable	
57	UCAM	Epinephelus morio	Red Grouper	N	Brought on Board (Retained/Discarded)	Live - Stomach and/or Eyes Protruding	Retained	Not Applicable	
57	UCAM	Epinephelus morio	Red Grouper	N	Brought on Board (Retained/Discarded)	Live - Stomach and/or Eyes Protruding	Retained	Not Applicable	
57	UCAM	Epinephelus morio	Red Grouper	N	Brought on Board (Retained/Discarded)	Live - Healthy	Discarded - Live and Healthy (Vented)	Not Applicable	
57	UCAM	Epinephelus morio	Red Grouper	N	Brought on Board (Retained/Discarded)	Live - Healthy	Discarded - Live and Healthy (Vented)	Not Applicable	
57	UCAM	Epinephelus morio	Red Grouper	N	Brought on Board (Retained/Discarded)	Live - Healthy	Discarded - Live and Healthy (Vented)	Not Applicable	
57	UCAM	Lutjanus campechanus	Red Snapper	N	Brought on Board (Retained/Discarded)	Live - Stomach and/or Eyes Protruding	Discarded - Live and Healthy (Vented)	Not Applicable	
57	UCAM	Lutjanus campechanus	Red Snapper	N	Brought on Board (Retained/Discarded)	Live - Stomach and/or Eyes Protruding	Discarded - Live and Healthy (Vented)	Not Applicable	
57	UCAM	Muraenidae, unidentified	Moray Eel, Unidentified	N	Brought on Board (Retained/Discarded)	Live - Healthy	Retained as Bait	Not Applicable	
58	NOCAM	Epinephelus morio	Red Grouper	N	Brought on Board (Retained/Discarded)	Live - Healthy	Retained	Not Applicable	
58	NOCAM	Epinephelus morio	Red Grouper	N	Brought on Board (Retained/Discarded)	Live - Healthy	Retained	Not Applicable	
58	NOCAM	Epinephelus morio	Red Grouper	N	Brought on Board (Retained/Discarded)	Live - Healthy	Retained	Not Applicable	
58	NOCAM	Epinephelus morio	Red Grouper	N	Brought on Board (Retained/Discarded)	Live - Stomach and/or Eyes Protruding	Retained	Not Applicable	
58	NOCAM	Epinephelus morio	Red Grouper	N	Brought on Board (Retained/Discarded)	Live - Stomach and/or Eyes Protruding	Retained	Not Applicable	
58	NOCAM	Epinephelus morio	Red Grouper	N	Brought on Board (Retained/Discarded)	Live - Stomach and/or Eyes Protruding	Retained	Not Applicable	
58	NOCAM	Epinephelus morio	Red Grouper	N	Brought on Board (Retained/Discarded)	Live - Stomach and/or Eyes Protruding	Retained	Not Applicable	
58	NOCAM	Epinephelus morio	Red Grouper	N	Brought on Board (Retained/Discarded)	Live - Healthy	Retained	Not Applicable	
58	NOCAM	Epinephelus morio	Red Grouper	N	Brought on Board (Retained/Discarded)	Live - Healthy	Retained	Not Applicable	
58	NOCAM	Epinephelus morio	Red Grouper	N	Brought on Board (Retained/Discarded)	Live - Stomach and/or Eyes Protruding	Retained	Not Applicable	
58	NOCAM	Epinephelus morio	Red Grouper	N	Brought on Board (Retained/Discarded)	Live - Stomach and/or Eyes Protruding	Retained	Not Applicable	
58	NOCAM	Epinephelus morio	Red Grouper	N	Brought on Board (Retained/Discarded)	Live - Healthy	Retained	Not Applicable	
58	NOCAM	Epinephelus morio	Red Grouper	N	Brought on Board (Retained/Discarded)	Live - Healthy	Retained	Not Applicable	
58	NOCAM	Epinephelus morio	Red Grouper	N	Brought on Board (Retained/Discarded)	Dead on Arrival - Damaged	Discarded - Dead	Not Applicable	
58	NOCAM	Epinephelus morio	Red Grouper	N	Brought on Board (Retained/Discarded)	Live - Stomach and/or Eyes Protruding	Retained	Not Applicable	
58	NOCAM	Epinephelus morio	Red Grouper	N	Brought on Board (Retained/Discarded)	Live - Healthy	Retained	Not Applicable	
58	NOCAM	Epinephelus morio	Red Grouper	N	Brought on Board (Retained/Discarded)	Live - Healthy	Retained	Not Applicable	
58	NOCAM	Epinephelus morio	Red Grouper	N	Brought on Board (Retained/Discarded)	Live - Stomach and/or Eyes Protruding	Retained	Not Applicable	
58	NOCAM	Epinephelus morio	Red Grouper	N	Brought on Board (Retained/Discarded)	Live - Healthy	Retained	Not Applicable	
58	NOCAM	Epinephelus morio	Red Grouper	N	Brought on Board (Retained/Discarded)	Live - Healthy	Retained	Not Applicable	
58	NOCAM	Epinephelus morio	Red Grouper	N	Brought on Board (Retained/Discarded)	Live - Healthy	Discarded - Live and Healthy (Vented)	Not Applicable	

58	NOCAM	Epinephelus morio	Red Grouper	N	Brought on Board (Retained/Discarded)	Live - Healthy	Retained	Not Applicable	
58	NOCAM	Epinephelus morio	Red Grouper	N	Brought on Board (Retained/Discarded)	Live - Healthy	Discarded - Live and Damaged (Not Ventec	Not Applicable	
58	NOCAM	Lutjanus analis	Mutton Snapper	N	Brought on Board (Retained/Discarded)	Live - Healthy	Retained	Not Applicable	
58	NOCAM	Lutjanus campechanus	Red Snapper	N	Brought on Board (Retained/Discarded)	Dead on Arrival - Damaged	Discarded - Dead	Not Applicable	
58	UCAM	Epinephelus morio	Red Grouper	N	Brought on Board (Retained/Discarded)	Live - Healthy	Retained	Not Applicable	
58	UCAM	Epinephelus morio	Red Grouper	N	Brought on Board (Retained/Discarded)	Live - Stomach and/or Eyes Protruding	Retained	Not Applicable	
58	UCAM	Epinephelus morio	Red Grouper	N	Brought on Board (Retained/Discarded)	Live - Stomach and/or Eyes Protruding	Retained	Not Applicable	
58	UCAM	Epinephelus morio	Red Grouper	N	Brought on Board (Retained/Discarded)	Live - Stomach and/or Eyes Protruding	Retained	Not Applicable	
58	UCAM	Epinephelus morio	Red Grouper	N	Brought on Board (Retained/Discarded)	Live - Healthy	Retained	Not Applicable	
58	UCAM	Epinephelus morio	Red Grouper	N	Brought on Board (Retained/Discarded)	Live - Healthy	Discarded - Live and Healthy (Not Vented)	Not Applicable	
58	UCAM	Epinephelus morio	Red Grouper	N	Brought on Board (Retained/Discarded)	Live - Healthy	Retained	Not Applicable	
58	UCAM	Epinephelus morio	Red Grouper	N	Brought on Board (Retained/Discarded)	Live - Healthy	Retained	Not Applicable	
58	UCAM	Epinephelus morio	Red Grouper	N	Brought on Board (Retained/Discarded)	Live - Stomach and/or Eyes Protruding	Retained	Not Applicable	
58	UCAM	Epinephelus morio	Red Grouper	N	Brought on Board (Retained/Discarded)	Live - Healthy	Retained	Not Applicable	
58	UCAM	Epinephelus morio	Red Grouper	N	Brought on Board (Retained/Discarded)	Live - Stomach and/or Eyes Protruding	Retained	Not Applicable	
58	UCAM	Epinephelus morio	Red Grouper	N	Brought on Board (Retained/Discarded)	Live - Healthy	Retained	Not Applicable	
58	UCAM	Epinephelus morio	Red Grouper	N	Brought on Board (Retained/Discarded)	Live - Healthy	Retained	Not Applicable	
58	UCAM	Epinephelus morio	Red Grouper	N	Not Handled (Dropped Off)	Live - Healthy	Discarded - Live and Healthy (Not Vented)	Not Applicable	
58	UCAM	Epinephelus morio	Red Grouper	N	Brought on Board (Retained/Discarded)	Live - Stomach and/or Eyes Protruding	Retained	Not Applicable	
58	UCAM	Epinephelus morio	Red Grouper	N	Brought on Board (Retained/Discarded)	Live - Healthy	Retained	Not Applicable	
58	UCAM	Epinephelus morio	Red Grouper	N	Brought on Board (Retained/Discarded)	Live - Healthy	Retained	Not Applicable	
58	UCAM	Epinephelus morio	Red Grouper	N	Brought on Board (Retained/Discarded)	Dead on Arrival - Damaged	Discarded - Dead	Not Applicable	
58	UCAM	Epinephelus morio	Red Grouper	N	Brought on Board (Retained/Discarded)	Live - Healthy	Retained	Not Applicable	
58	UCAM	Epinephelus morio	Red Grouper	N	Brought on Board (Retained/Discarded)	Live - Healthy	Discarded - Live and Healthy (Vented)	Not Applicable	
58	UCAM	Epinephelus morio	Red Grouper	N	Brought on Board (Retained/Discarded)	Live - Stomach and/or Eyes Protruding	Retained	Not Applicable	
58	UCAM	Epinephelus morio	Red Grouper	N	Brought on Board (Retained/Discarded)	Live - Healthy	Retained	Not Applicable	
58	UCAM	Epinephelus morio	Red Grouper	N	Brought on Board (Retained/Discarded)	Live - Healthy	Retained	Not Applicable	
58	UCAM	Epinephelus morio	Red Grouper	N	Brought on Board (Retained/Discarded)	Live - Healthy	Retained	Not Applicable	
58	UCAM	Galeocerdo cuvier	Tiger Shark	Y	Cut-off at Rail (No Entanglement)	Live - Healthy	Discarded - Live and Healthy (Not Vented)	Known Juvenile	Medium (Between 1m and 2m)
58	UCAM	Lutjanus analis	Mutton Snapper	N	Brought on Board (Retained/Discarded)	Live - Healthy	Retained	Not Applicable	
58	UCAM	Lutjanus campechanus	Red Snapper	N	Brought on Board (Retained/Discarded)	Dead on Arrival - Damaged	Discarded - Dead	Not Applicable	
59	NOCAM	Crab, unidentified	Crab, Unidentified	N	Brought on Board (Retained/Discarded)	Live - Healthy	Discarded - Live and Healthy (Not Vented)	Not Applicable	
59	NOCAM	Epinephelus flavolimbatus	Yellowedge Grouper	N	Brought on Board (Retained/Discarded)	Live - Stomach and/or Eyes Protruding	Retained	Not Applicable	
59	NOCAM	Epinephelus niveatus	Snowy Grouper	N	Brought on Board (Retained/Discarded)	Live - Stomach and/or Eyes Protruding	Retained	Not Applicable	
59	NOCAM	Epinephelus niveatus	Snowy Grouper	N	Brought on Board (Retained/Discarded)	Live - Healthy	Retained	Not Applicable	
59	NOCAM	Muraenidae, unidentified	Moray Eel, Unidentified	N	Brought on Board (Retained/Discarded)	Live - Healthy	Retained as Bait	Not Applicable	
59	NOCAM	Mustelus spp.	Dogfish, Smooth (Florida)	Y	Brought on Board (Retained/Discarded)	Live - Healthy	Discarded - Live and Healthy (Not Vented)	Male (Claspers)	Small (Less than 1m)
59	NOCAM	Mustelus spp.	Dogfish, Smooth (Florida)	Y	Brought on Board (Retained/Discarded)	Live - Healthy	Discarded - Live and Healthy (Not Vented)	Male (Claspers)	Small (Less than 1m)
59	NOCAM	Mustelus spp.	Dogfish, Smooth (Florida)	Y	Brought on Board (Retained/Discarded)	Live - Healthy	Discarded - Live and Healthy (Not Vented)	Male (Claspers)	Small (Less than 1m)
59	NOCAM	Mustelus spp.	Dogfish, Smooth (Florida)	Y	Brought on Board (Retained/Discarded)	Live - Healthy	Discarded - Live and Healthy (Not Vented)	Female	Small (Less than 1m)
59	NOCAM	Mustelus spp.	Dogfish, Smooth (Florida)	Y	Brought on Board (Retained/Discarded)	Live - Healthy	Discarded - Live and Healthy (Not Vented)	Male (Claspers)	Small (Less than 1m)
59	NOCAM	Mustelus spp.	Dogfish, Smooth (Florida)	Y	Brought on Board (Retained/Discarded)	Live - Healthy	Discarded - Live and Healthy (Not Vented)	Male (Claspers)	Small (Less than 1m)
59	NOCAM	Mustelus spp.	Dogfish, Smooth (Florida)	Y	Brought on Board (Retained/Discarded)	Live - Healthy	Discarded - Live and Healthy (Not Vented)	Male (Claspers)	Small (Less than 1m)
59	NOCAM	Mustelus spp.	Dogfish, Smooth (Florida)	Y	Brought on Board (Retained/Discarded)	Live - Healthy	Discarded - Live and Healthy (Not Vented)	Male (Claspers)	Small (Less than 1m)
59	NOCAM	Mustelus spp.	Dogfish, Smooth (Florida)	Y	Brought on Board (Retained/Discarded)	Live - Healthy	Discarded - Live and Healthy (Not Vented)	Female	Small (Less than 1m)
59	NOCAM	Mustelus spp.	Dogfish, Smooth (Florida)	Y	Brought on Board (Retained/Discarded)	Live - Healthy	Discarded - Live and Healthy (Not Vented)	Male (Claspers)	Small (Less than 1m)
59	NOCAM	Mustelus spp.	Dogfish, Smooth (Florida)	Y	Brought on Board (Retained/Discarded)	Live - Healthy	Discarded - Live and Healthy (Not Vented)	Male (Claspers)	Small (Less than 1m)
59	NOCAM	Mustelus spp.	Dogfish, Smooth (Florida)	Y	Brought on Board (Retained/Discarded)	Live - Healthy	Discarded - Live and Healthy (Not Vented)	Female	Small (Less than 1m)
59	NOCAM	Mustelus spp.	Dogfish, Smooth (Florida)	Y	Brought on Board (Retained/Discarded)	Live - Healthy	Discarded - Live and Healthy (Not Vented)	Male (Claspers)	Small (Less than 1m)
59	NOCAM	Mustelus spp.	Dogfish, Smooth (Florida)	Y	Brought on Board (Retained/Discarded)	Live - Healthy	Discarded - Live and Healthy (Not Vented)	Male (Claspers)	Small (Less than 1m)
59	NOCAM	Mustelus spp.	Dogfish, Smooth (Florida)	Y	Brought on Board (Retained/Discarded)	Live - Healthy	Discarded - Live and Healthy (Not Vented)	Female	Small (Less than 1m)
59	NOCAM	Mustelus spp.	Dogfish, Smooth (Florida)	Y	Brought on Board (Retained/Discarded)	Live - Healthy	Discarded - Live and Healthy (Not Vented)	Male (Claspers)	Small (Less than 1m)
59	NOCAM	Mustelus spp.	Dogfish, Smooth (Florida)	Y	Brought on Board (Retained/Discarded)	Live - Healthy	Discarded - Live and Healthy (Not Vented)	Female	Small (Less than 1m)
59	NOCAM	Mustelus spp.	Dogfish, Smooth (Florida)	Y	Brought on Board (Retained/Discarded)	Live - Healthy	Discarded - Live and Healthy (Not Vented)	Male (Claspers)	Small (Less than 1m)
59	NOCAM	Mustelus spp.	Dogfish, Smooth (Florida)	Y	Brought on Board (Retained/Discarded)	Live - Healthy	Discarded - Live and Healthy (Not Vented)	Male (Claspers)	Small (Less than 1m)
59	NOCAM	Mustelus spp.	Dogfish, Smooth (Florida)	Y	Brought on Board (Retained/Discarded)	Live - Healthy	Discarded - Live and Healthy (Not Vented)	Male (Claspers)	Small (Less than 1m)
59	NOCAM	Mustelus spp.	Dogfish, Smooth (Florida)	Y	Brought on Board (Retained/Discarded)	Live - Healthy	Discarded - Live and Healthy (Not Vented)	Male (Claspers)	Small (Less than 1m)
59	NOCAM	Mustelus spp.	Dogfish, Smooth (Florida)	Y	Brought on Board (Retained/Discarded)	Live - Healthy	Discarded - Live and Healthy (Not Vented)	Male (Claspers)	Medium (Between 1m and 2m)
59	NOCAM	Mustelus spp.	Dogfish, Smooth (Florida)	Y	Brought on Board (Retained/Discarded)	Live - Healthy	Discarded - Live and Healthy (Not Vented)	Female	Small (Less than 1m)
59	NOCAM	Mustelus spp.	Dogfish, Smooth (Florida)	Y	Brought on Board (Retained/Discarded)	Live - Healthy	Discarded - Live and Healthy (Not Vented)	Male (Claspers)	Small (Less than 1m)
59	NOCAM	Mustelus spp.	Dogfish, Smooth (Florida)	Y	Brought on Board (Retained/Discarded)	Live - Healthy	Discarded - Live and Healthy (Not Vented)	Female	Small (Less than 1m)
59	NOCAM	Mustelus spp.	Dogfish, Smooth (Florida)	Y	Brought on Board (Retained/Discarded)	Live - Healthy	Discarded - Live and Healthy (Not Vented)	Male (Claspers)	Small (Less than 1m)
59	NOCAM	Mycteroperca microlepis	Gag Grouper	N	Brought on Board (Retained/Discarded)	Live - Stomach and/or Eyes Protruding	Retained	Not Applicable	
59	NOCAM	Mycteroperca microlepis	Gag Grouper	N	Brought on Board (Retained/Discarded)	Dead on Arrival - Damaged	Discarded - Dead	Not Applicable	
59	NOCAM	Ophichthus rex	King Snake Eel	N	Brought on Board (Retained/Discarded)	Live - Healthy	Retained as Bait	Not Applicable	
59	NOCAM	Selachimorpha, unidentified	Shark, Unidentified	Y	Cut-off at Rail (No Entanglement)	Live - Healthy	Discarded - Live and Healthy (Not Vented)	Unknown Maturity and/or Sex	Large (Greater than 2m)
59	NOCAM	Seriola rivoliana	Almaco Jack	N	Brought on Board (Retained/Discarded)	Live - Healthy	Retained	Not Applicable	
59	NOCAM	Seriola rivoliana	Almaco Jack	N	Brought on Board (Retained/Discarded)	Live - Healthy	Discarded - Live and Healthy (Vented)	Not Applicable	
59	NOCAM	Sphyrna lewini	Scalloped Hammerhead	Y	Cut-off at Rail (No Entanglement)	Dead on Arrival - Undamaged	Discarded - Dead	Unknown Maturity and/or Sex	Medium (Between 1m and 2m)
59	NOCAM	Synodus spp.	Lizardfish (all)	N	Brought on Board (Retained/Discarded)	Live - Healthy	Retained as Bait	Not Applicable	
59	UCAM	Carcharhinus plumbeus	Sandbar Shark	Y	Cut-off at Rail (No Entanglement)	Live - Healthy	Discarded - Live and Healthy (Not Vented)	Male (Claspers)	Large (Greater than 2m)
59	UCAM	Crab, unidentified	Crab, Unidentified	N	Brought on Board (Retained/Discarded)	Live - Healthy	Discarded - Live and Healthy (Not Vented)	Not Applicable	
59	UCAM	Crab, unidentified	Crab, Unidentified	N	Brought on Board (Retained/Discarded)	Live - Healthy	Discarded - Live and Healthy (Not Vented)	Not Applicable	
59	UCAM	Epinephelus flavolimbatus	Yellowedge Grouper	N	Brought on Board (Retained/Discarded)	Live - Stomach and/or Eyes Protruding	Retained	Not Applicable	
59	UCAM	Epinephelus niveatus	Snowy Grouper	N	Brought on Board (Retained/Discarded)	Live - Healthy	Retained	Not Applicable	
59	UCAM	Muraenidae, unidentified	Moray Eel, Unidentified	N	Brought on Board (Retained/Discarded)	Live - Healthy	Retained as Bait	Not Applicable	
59	UCAM	Mustelus spp.	Dogfish, Smooth (Florida)	Y	Brought on Board (Retained/Discarded)	Live - Healthy	Discarded - Live and Healthy (Not Vented)	Male (Claspers)	Small (Less than 1m)
59	UCAM	Mustelus spp.	Dogfish, Smooth (Florida)	Y	Brought on Board (Retained/Discarded)	Live - Healthy	Discarded - Live and Healthy (Not Vented)	Female	Small (Less than 1m)
59	UCAM	Mustelus spp.	Dogfish, Smooth (Florida)	Y	Brought on Board (Retained/Discarded)	Live - Healthy	Discarded - Live and Healthy (Not Vented)	Male (Claspers)	Small (Less than 1m)
59	UCAM	Mustelus spp.	Dogfish, Smooth (Florida)	Y	Brought on Board (Retained/Discarded)	Live - Healthy	Discarded - Live and Healthy (Not Vented)	Male (Claspers)	Small (Less than 1m)
59	UCAM	Mustelus spp.	Dogfish, Smooth (Florida)	Y	Brought on Board (Retained/Discarded)	Live - Healthy	Discarded - Live and Healthy (Not Vented)	Male (Claspers)	Small (Less than 1m)
59	UCAM	Mustelus spp.	Dogfish, Smooth (Florida)	Y	Brought on Board (Retained/Discarded)	Live - Healthy	Discarded - Live and Healthy (Not Vented)	Female	Small (Less than 1m)
59	UCAM	Mustelus spp.	Dogfish, Smooth (Florida)	Y	Brought on Board (Retained/Discarded)	Live - Healthy	Discarded - Live and Healthy (Not Vented)	Male (Claspers)	Small (Less than 1m)
59	UCAM	Mustelus spp.	Dogfish, Smooth (Florida)	Y	Brought on Board (Retained/Discarded)	Live - Healthy	Discarded - Live and Healthy (Not Vented)	Male (Claspers)	Small (Less than 1m)
59	UCAM	Mustelus spp.	Dogfish, Smooth (Florida)	Y	Brought on Board (Retained/Discarded)	Live - Healthy	Discarded - Live and Healthy (Not Vented)	Male (Claspers)	Small (Less than 1m)
59	UCAM	Mustelus spp.	Dogfish, Smooth (Florida)	Y	Brought on Board (Retained/Discarded)	Live - Healthy	Discarded - Live and Healthy (Not Vented)	Male (Claspers)	Small (Less than 1m)
59	UCAM	Mustelus spp.	Dogfish, Smooth (Florida)	Y	Brought on Board (Retained/Discarded)	Live - Healthy	Discarded - Live and Healthy (Not Vented)	Male (Claspers)	Small (Less than 1m)
59	UCAM	Mustelus spp.	Dogfish, Smooth (Florida)	Y	Brought on Board (Retained/Discarded)	Live - Healthy	Discarded - Live and Healthy (Not Vented)	Female	Small (Less than 1m)
59	UCAM	Mustelus spp.	Dogfish, Smooth (Florida)	Y	Brought on Board (Retained/Discarded)	Live - Healthy	Discarded - Live and Healthy (Not Vented)	Male (Claspers)	Small (Less than 1m)
59	UCAM	Mustelus spp.	Dogfish, Smooth (Florida)	Y	Brought on Board (Retained/Discarded)	Live - Healthy	Discarded - Live and Healthy (Not Vented)	Male (Claspers)	Medium (Between 1m and 2m)
59	UCAM	Mustelus spp.	Dogfish, Smooth (Florida)	Y	Brought on Board (Retained/Discarded)	Live - Healthy	Discarded - Live and Healthy (Not Vented)	Male (Claspers)	Small (Less than 1m)
59	UCAM	Mustelus spp.	Dogfish, Smooth (Florida)	Y	Brought on Board (Retained/Discarded)	Live - Healthy	Discarded - Live and Healthy (Not Vented)	Female	Small (Less than 1m)

59	UCAM	Mustelus spp.	Dogfish, Smooth (Florida)	Y	Brought on Board (Retained/Discarded)	Live - Healthy	Discarded - Live and Healthy (Not Vented)	Female	Small (Less than 1m)
59	UCAM	Mustelus spp.	Dogfish, Smooth (Florida)	Y	Brought on Board (Retained/Discarded)	Live - Healthy	Discarded - Live and Healthy (Not Vented)	Male (Claspers)	Small (Less than 1m)
59	UCAM	Mustelus spp.	Dogfish, Smooth (Florida)	Y	Brought on Board (Retained/Discarded)	Live - Healthy	Discarded - Live and Healthy (Not Vented)	Male (Claspers)	Small (Less than 1m)
59	UCAM	Mustelus spp.	Dogfish, Smooth (Florida)	Y	Brought on Board (Retained/Discarded)	Live - Healthy	Discarded - Live and Healthy (Not Vented)	Male (Claspers)	Small (Less than 1m)
59	UCAM	Mustelus spp.	Dogfish, Smooth (Florida)	Y	Brought on Board (Retained/Discarded)	Live - Healthy	Discarded - Live and Healthy (Not Vented)	Female	Small (Less than 1m)
59	UCAM	Mustelus spp.	Dogfish, Smooth (Florida)	Y	Brought on Board (Retained/Discarded)	Live - Healthy	Discarded - Live and Healthy (Not Vented)	Male (Claspers)	Small (Less than 1m)
59	UCAM	Mustelus spp.	Dogfish, Smooth (Florida)	Y	Brought on Board (Retained/Discarded)	Live - Healthy	Discarded - Live and Healthy (Not Vented)	Male (Claspers)	Small (Less than 1m)
59	UCAM	Mustelus spp.	Dogfish, Smooth (Florida)	Y	Brought on Board (Retained/Discarded)	Live - Healthy	Discarded - Live and Healthy (Not Vented)	Male (Claspers)	Small (Less than 1m)
59	UCAM	Mustelus spp.	Dogfish, Smooth (Florida)	Y	Brought on Board (Retained/Discarded)	Live - Healthy	Discarded - Live and Healthy (Not Vented)	Female	Small (Less than 1m)
59	UCAM	Mustelus spp.	Dogfish, Smooth (Florida)	Y	Brought on Board (Retained/Discarded)	Live - Healthy	Discarded - Live and Healthy (Not Vented)	Male (Claspers)	Small (Less than 1m)
59	UCAM	Mycteroperca microlepis	Gag Grouper	N	Brought on Board (Retained/Discarded)	Live - Healthy	Retained	Not Applicable	
59	UCAM	Ophichthus rex	King Snake Eel	N	Brought on Board (Retained/Discarded)	Live - Healthy	Retained as Bait	Not Applicable	
59	UCAM	Seriola rivoliana	Almaco Jack	N	Brought on Board (Retained/Discarded)	Live - Healthy	Retained	Not Applicable	
59	UCAM	Seriola rivoliana	Almaco Jack	N	Brought on Board (Retained/Discarded)	Live - Healthy	Discarded - Live and Healthy (Vented)	Not Applicable	
59	UCAM	Sphyrna lewini	Scalloped Hammerhead	Y	Cut-off at Rail (No Entanglement)	Dead on Arrival - Undamaged	Discarded - Dead	Female	Medium (Between 1m and 2m)
60	NOCAM	Caulolatilus microps	Blueline Tilefish	N	Brought on Board (Retained/Discarded)	Live - Healthy	Retained	Not Applicable	
60	NOCAM	Caulolatilus microps	Blueline Tilefish	N	Brought on Board (Retained/Discarded)	Live - Healthy	Retained	Not Applicable	
60	NOCAM	Epinephelus flavolimbatus	Yellowedge Grouper	N	Brought on Board (Retained/Discarded)	Live - Stomach and/or Eyes Protruding	Retained	Not Applicable	
60	NOCAM	Epinephelus flavolimbatus	Yellowedge Grouper	N	Brought on Board (Retained/Discarded)	Live - Stomach and/or Eyes Protruding	Retained	Not Applicable	
60	NOCAM	Epinephelus flavolimbatus	Yellowedge Grouper	N	Brought on Board (Retained/Discarded)	Live - Stomach and/or Eyes Protruding	Retained	Not Applicable	
60	NOCAM	Epinephelus flavolimbatus	Yellowedge Grouper	N	Brought on Board (Retained/Discarded)	Live - Stomach and/or Eyes Protruding	Retained	Not Applicable	
60	NOCAM	Epinephelus flavolimbatus	Yellowedge Grouper	N	Brought on Board (Retained/Discarded)	Live - Stomach and/or Eyes Protruding	Retained	Not Applicable	
60	NOCAM	Epinephelus flavolimbatus	Yellowedge Grouper	N	Brought on Board (Retained/Discarded)	Live - Stomach and/or Eyes Protruding	Retained	Not Applicable	
60	NOCAM	Epinephelus flavolimbatus	Yellowedge Grouper	N	Brought on Board (Retained/Discarded)	Live - Stomach and/or Eyes Protruding	Retained	Not Applicable	
60	NOCAM	Epinephelus flavolimbatus	Yellowedge Grouper	N	Brought on Board (Retained/Discarded)	Live - Stomach and/or Eyes Protruding	Retained	Not Applicable	
60	NOCAM	Epinephelus flavolimbatus	Yellowedge Grouper	N	Brought on Board (Retained/Discarded)	Live - Stomach and/or Eyes Protruding	Retained	Not Applicable	
60	NOCAM	Gadiformes, unidentified	Hake, Unidentified	N	Brought on Board (Retained/Discarded)	Live - Healthy	Retained as Bait	Not Applicable	
60	NOCAM	Hexanchus spp.	Sixgill Shark (all)	Y	Brought on Board (Retained/Discarded)	Live - Healthy	Discarded - Live and Healthy (Not Vented)	Female	Small (Less than 1m)
60	NOCAM	Lopholatilus chamaelonticeps	Tilefish, Golden	N	Brought on Board (Retained/Discarded)	Live - Stomach and/or Eyes Protruding	Retained	Not Applicable	
60	NOCAM	Lopholatilus chamaelonticeps	Tilefish, Golden	N	Brought on Board (Retained/Discarded)	Live - Healthy	Retained	Not Applicable	
60	NOCAM	Lopholatilus chamaelonticeps	Tilefish, Golden	N	Brought on Board (Retained/Discarded)	Live - Stomach and/or Eyes Protruding	Retained	Not Applicable	
60	NOCAM	Lopholatilus chamaelonticeps	Tilefish, Golden	N	Brought on Board (Retained/Discarded)	Live - Healthy	Retained	Not Applicable	
60	NOCAM	Lopholatilus chamaelonticeps	Tilefish, Golden	N	Brought on Board (Retained/Discarded)	Live - Healthy	Retained	Not Applicable	
60	NOCAM	Lopholatilus chamaelonticeps	Tilefish, Golden	N	Brought on Board (Retained/Discarded)	Live - Healthy	Retained	Not Applicable	
60	NOCAM	Lopholatilus chamaelonticeps	Tilefish, Golden	N	Brought on Board (Retained/Discarded)	Live - Stomach and/or Eyes Protruding	Retained	Not Applicable	
60	NOCAM	Lopholatilus chamaelonticeps	Tilefish, Golden	N	Brought on Board (Retained/Discarded)	Live - Healthy	Retained	Not Applicable	
60	NOCAM	Muraenidae, unidentified	Moray Eel, Unidentified	N	Brought on Board (Retained/Discarded)	Live - Healthy	Retained as Bait	Not Applicable	
60	NOCAM	Neomerinthe hemingwayi	Scorpionfish, Spiny Cheek	N	Brought on Board (Retained/Discarded)	Live - Stomach and/or Eyes Protruding	Retained	Not Applicable	
60	NOCAM	Ophichthus rex	King Snake Eel	N	Brought on Board (Retained/Discarded)	Live - Healthy	Retained as Bait	Not Applicable	
60	NOCAM	Ophichthus rex	King Snake Eel	N	Brought on Board (Retained/Discarded)	Live - Healthy	Retained as Bait	Not Applicable	
60	NOCAM	Squalidae spp.	Dogfish, Spiny (Cuban)	Y	Brought on Board (Retained/Discarded)	Live - Healthy	Discarded - Live and Healthy (Not Vented)	Unknown Maturity and/or Sex	Small (Less than 1m)
60	NOCAM	Squalidae spp.	Dogfish, Spiny (Cuban)	Y	Brought on Board (Retained/Discarded)	Live - Healthy	Discarded - Live and Healthy (Not Vented)	Unknown Maturity and/or Sex	Small (Less than 1m)
60	NOCAM	Squalidae spp.	Dogfish, Spiny (Cuban)	Y	Brought on Board (Retained/Discarded)	Live - Healthy	Discarded - Live and Healthy (Not Vented)	Unknown Maturity and/or Sex	Small (Less than 1m)
60	NOCAM	Squalidae spp.	Dogfish, Spiny (Cuban)	Y	Brought on Board (Retained/Discarded)	Live - Healthy	Discarded - Live and Healthy (Not Vented)	Female	Small (Less than 1m)
60	NOCAM	Squalidae spp.	Dogfish, Spiny (Cuban)	Y	Brought on Board (Retained/Discarded)	Live - Healthy	Discarded - Live and Healthy (Not Vented)	Unknown Maturity and/or Sex	Small (Less than 1m)
60	NOCAM	Synodus spp.	Lizardfish (all)	N	Brought on Board (Retained/Discarded)	Live - Healthy	Retained as Bait	Not Applicable	
60	NOCAM	Synodus spp.	Lizardfish (all)	N	Brought on Board (Retained/Discarded)	Live - Healthy	Retained as Bait	Not Applicable	
60	UCAM	Caulolatilus microps	Blueline Tilefish	N	Brought on Board (Retained/Discarded)	Live - Healthy	Retained	Not Applicable	
60	UCAM	Caulolatilus microps	Blueline Tilefish	N	Brought on Board (Retained/Discarded)	Live - Healthy	Retained	Not Applicable	
60	UCAM	Epinephelus flavolimbatus	Yellowedge Grouper	N	Brought on Board (Retained/Discarded)	Live - Stomach and/or Eyes Protruding	Retained	Not Applicable	
60	UCAM	Epinephelus flavolimbatus	Yellowedge Grouper	N	Brought on Board (Retained/Discarded)	Live - Stomach and/or Eyes Protruding	Retained	Not Applicable	
60	UCAM	Epinephelus flavolimbatus	Yellowedge Grouper	N	Brought on Board (Retained/Discarded)	Live - Stomach and/or Eyes Protruding	Retained	Not Applicable	
60	UCAM	Epinephelus flavolimbatus	Yellowedge Grouper	N	Brought on Board (Retained/Discarded)	Live - Stomach and/or Eyes Protruding	Retained	Not Applicable	
60	UCAM	Epinephelus flavolimbatus	Yellowedge Grouper	N	Brought on Board (Retained/Discarded)	Live - Stomach and/or Eyes Protruding	Retained	Not Applicable	
60	UCAM	Epinephelus flavolimbatus	Yellowedge Grouper	N	Brought on Board (Retained/Discarded)	Live - Stomach and/or Eyes Protruding	Retained	Not Applicable	
60	UCAM	Epinephelus flavolimbatus	Yellowedge Grouper	N	Brought on Board (Retained/Discarded)	Live - Stomach and/or Eyes Protruding	Retained	Not Applicable	
60	UCAM	Epinephelus flavolimbatus	Yellowedge Grouper	N	Brought on Board (Retained/Discarded)	Live - Stomach and/or Eyes Protruding	Retained	Not Applicable	
60	UCAM	Epinephelus flavolimbatus	Yellowedge Grouper	N	Brought on Board (Retained/Discarded)	Live - Stomach and/or Eyes Protruding	Retained	Not Applicable	
60	UCAM	Gadiformes, unidentified	Hake, Unidentified	N	Brought on Board (Retained/Discarded)	Live - Healthy	Retained as Bait	Not Applicable	
60	UCAM	Hexanchus spp.	Sixgill Shark (all)	Y	Brought on Board (Retained/Discarded)	Live - Healthy	Discarded - Live and Healthy (Not Vented)	Female	Small (Less than 1m)
60	UCAM	Lopholatilus chamaelonticeps	Tilefish, Golden	N	Brought on Board (Retained/Discarded)	Live - Stomach and/or Eyes Protruding	Retained	Not Applicable	
60	UCAM	Lopholatilus chamaelonticeps	Tilefish, Golden	N	Brought on Board (Retained/Discarded)	Live - Stomach and/or Eyes Protruding	Retained	Not Applicable	
60	UCAM	Lopholatilus chamaelonticeps	Tilefish, Golden	N	Brought on Board (Retained/Discarded)	Live - Stomach and/or Eyes Protruding	Retained	Not Applicable	
60	UCAM	Lopholatilus chamaelonticeps	Tilefish, Golden	N	Brought on Board (Retained/Discarded)	Live - Healthy	Retained	Not Applicable	
60	UCAM	Lopholatilus chamaelonticeps	Tilefish, Golden	N	Brought on Board (Retained/Discarded)	Live - Stomach and/or Eyes Protruding	Retained	Not Applicable	
60	UCAM	Lopholatilus chamaelonticeps	Tilefish, Golden	N	Brought on Board (Retained/Discarded)	Live - Healthy	Retained	Not Applicable	
60	UCAM	Lopholatilus chamaelonticeps	Tilefish, Golden	N	Brought on Board (Retained/Discarded)	Live - Stomach and/or Eyes Protruding	Retained	Not Applicable	
60	UCAM	Lopholatilus chamaelonticeps	Tilefish, Golden	N	Brought on Board (Retained/Discarded)	Live - Healthy	Retained	Not Applicable	
60	UCAM	Muraenidae, unidentified	Moray Eel, Unidentified	N	Brought on Board (Retained/Discarded)	Live - Healthy	Retained as Bait	Not Applicable	
60	UCAM	Neomerinthe hemingwayi	Scorpionfish, Spiny Cheek	N	Brought on Board (Retained/Discarded)	Live - Stomach and/or Eyes Protruding	Retained	Not Applicable	
60	UCAM	Ophichthus rex	King Snake Eel	N	Brought on Board (Retained/Discarded)	Live - Healthy	Retained as Bait	Not Applicable	
60	UCAM	Ophichthus rex	King Snake Eel	N	Brought on Board (Retained/Discarded)	Live - Healthy	Retained as Bait	Not Applicable	
60	UCAM	Squalidae spp.	Dogfish, Spiny (Cuban)	Y	Brought on Board (Retained/Discarded)	Live - Healthy	Discarded - Live and Healthy (Not Vented)	Unknown Maturity and/or Sex	Small (Less than 1m)
60	UCAM	Squalidae spp.	Dogfish, Spiny (Cuban)	Y	Brought on Board (Retained/Discarded)	Live - Healthy	Discarded - Live and Healthy (Not Vented)	Female	Small (Less than 1m)
60	UCAM	Squalidae spp.	Dogfish, Spiny (Cuban)	Y	Brought on Board (Retained/Discarded)	Live - Healthy	Discarded - Live and Healthy (Not Vented)	Unknown Maturity and/or Sex	Small (Less than 1m)
60	UCAM	Squalidae spp.	Dogfish, Spiny (Cuban)	Y	Brought on Board (Retained/Discarded)	Live - Healthy	Discarded - Live and Healthy (Not Vented)	Unknown Maturity and/or Sex	Small (Less than 1m)
60	UCAM	Synodus spp.	Lizardfish (all)	N	Brought on Board (Retained/Discarded)	Live - Healthy	Retained as Bait	Not Applicable	
60	UCAM	Synodus spp.	Lizardfish (all)	N	Brought on Board (Retained/Discarded)	Live - Healthy	Retained as Bait	Not Applicable	
61	NOCAM	Epinephelus flavolimbatus	Yellowedge Grouper	N	Brought on Board (Retained/Discarded)	Live - Stomach and/or Eyes Protruding	Retained	Not Applicable	
61	NOCAM	Epinephelus flavolimbatus	Yellowedge Grouper	N	Brought on Board (Retained/Discarded)	Live - Stomach and/or Eyes Protruding	Retained	Not Applicable	
61	NOCAM	Gadiformes, unidentified	Hake, Unidentified	N	Brought on Board (Retained/Discarded)	Live - Stomach and/or Eyes Protruding	Retained as Bait	Not Applicable	
61	NOCAM	Gadiformes, unidentified	Hake, Unidentified	N	Brought on Board (Retained/Discarded)	Live - Stomach and/or Eyes Protruding	Retained as Bait	Not Applicable	
61	NOCAM	Gadiformes, unidentified	Hake, Unidentified	N	Brought on Board (Retained/Discarded)	Live - Stomach and/or Eyes Protruding	Retained as Bait	Not Applicable	
61	NOCAM	Lopholatilus chamaelonticeps	Tilefish, Golden	N	Brought on Board (Retained/Discarded)	Dead on Arrival - Damaged	Discarded - Dead	Not Applicable	
61	NOCAM	Lopholatilus chamaelonticeps	Tilefish, Golden	N	Brought on Board (Retained/Discarded)	Live - Healthy	Retained	Not Applicable	
61	NOCAM	Ophichthus rex	King Snake Eel	N	Brought on Board (Retained/Discarded)	Live - Healthy	Retained as Bait	Not Applicable	
61	NOCAM	Squalidae spp.	Dogfish, Spiny (Cuban)	Y	Brought on Board (Retained/Discarded)	Live - Healthy	Discarded - Live and Healthy (Not Vented)	Unknown Maturity and/or Sex	Small (Less than 1m)
61	NOCAM	Squalidae spp.	Dogfish, Spiny (Cuban)	Y	Brought on Board (Retained/Discarded)	Live - Healthy	Discarded - Live and Healthy (Not Vented)	Unknown Maturity and/or Sex	Small (Less than 1m)
61	NOCAM	Squalidae spp.	Dogfish, Spiny (Cuban)	Y	Brought on Board (Retained/Discarded)	Live - Healthy	Discarded - Live and Healthy (Not Vented)	Unknown Maturity and/or Sex	Small (Less than 1m)
61	NOCAM	Squalidae spp.	Dogfish, Spiny (Cuban)	Y	Brought on Board (Retained/Discarded)	Live - Healthy	Discarded - Live and Healthy (Not Vented)	Unknown Maturity and/or Sex	Small (Less than 1m)
61	NOCAM	Squalidae spp.	Dogfish, Spiny (Cuban)	Y	Brought on Board (Retained/Discarded)	Live - Healthy	Discarded - Live and Healthy (Not Vented)	Unknown Maturity and/or Sex	Small (Less than 1m)
61	NOCAM	Squalidae spp.	Dogfish, Spiny (Cuban)	Y	Brought on Board (Retained/Discarded)	Live - Healthy	Discarded - Live and Healthy (Not Vented)	Unknown Maturity and/or Sex	Small (Less than 1m)

61	NOCAM	Squalidae spp.	Dogfish, Spiny (Cuban)	Y	Cut-off at Rail (No Entanglement)	Live - Healthy	Discarded - Live and Healthy (Not Vented)	Unknown Maturity and/or Sex	Small (Less than 1m)
61	UCAM	Epinephelus flavolimbatus	Yellowedge Grouper	N	Brought on Board (Retained/Discarded)	Live - Stomach and/or Eyes Protruding	Retained	Not Applicable	
61	UCAM	Epinephelus flavolimbatus	Yellowedge Grouper	N	Brought on Board (Retained/Discarded)	Live - Stomach and/or Eyes Protruding	Retained	Not Applicable	
61	UCAM	Gadiformes, unidentified	Hake, Unidentified	N	Brought on Board (Retained/Discarded)	Live - Stomach and/or Eyes Protruding	Retained as Bait	Not Applicable	
61	UCAM	Gadiformes, unidentified	Hake, Unidentified	N	Brought on Board (Retained/Discarded)	Live - Stomach and/or Eyes Protruding	Retained	Not Applicable	
61	UCAM	Gadiformes, unidentified	Hake, Unidentified	N	Brought on Board (Retained/Discarded)	Live - Stomach and/or Eyes Protruding	Retained as Bait	Not Applicable	
61	UCAM	Lopholatilus chamaelonticeps	Tilefish, Golden	N	Brought on Board (Retained/Discarded)	Dead on Arrival - Damaged	Discarded - Dead	Not Applicable	
61	UCAM	Lopholatilus chamaelonticeps	Tilefish, Golden	N	Brought on Board (Retained/Discarded)	Live - Healthy	Retained	Not Applicable	
61	UCAM	Ophichthus rex	King Snake Eel	N	Brought on Board (Retained/Discarded)	Live - Healthy	Retained as Bait	Not Applicable	
61	UCAM	Squalidae spp.	Dogfish, Spiny (Cuban)	Y	Cut-off at Rail (No Entanglement)	Live - Healthy	Discarded - Live and Healthy (Not Vented)	Unknown Maturity and/or Sex	Small (Less than 1m)
61	UCAM	Squalidae spp.	Dogfish, Spiny (Cuban)	Y	Brought on Board (Retained/Discarded)	Live - Healthy	Discarded - Live and Healthy (Not Vented)	Unknown Maturity and/or Sex	Small (Less than 1m)
61	UCAM	Squalidae spp.	Dogfish, Spiny (Cuban)	Y	Brought on Board (Retained/Discarded)	Live - Healthy	Discarded - Live and Healthy (Not Vented)	Unknown Maturity and/or Sex	Small (Less than 1m)
61	UCAM	Squalidae spp.	Dogfish, Spiny (Cuban)	Y	Brought on Board (Retained/Discarded)	Live - Healthy	Discarded - Live and Healthy (Not Vented)	Unknown Maturity and/or Sex	Small (Less than 1m)
61	UCAM	Squalidae spp.	Dogfish, Spiny (Cuban)	Y	Brought on Board (Retained/Discarded)	Live - Healthy	Discarded - Live and Healthy (Not Vented)	Unknown Maturity and/or Sex	Small (Less than 1m)
62	NOCAM	Acanthocybius solanderi	Wahoo	N	Brought on Board (Retained/Discarded)	Dead on Arrival - Damaged	Discarded - Dead	Not Applicable	
62	NOCAM	Caulolatilus microps	Blueline Tilefish	N	Brought on Board (Retained/Discarded)	Live - Healthy	Retained	Not Applicable	
62	NOCAM	Caulolatilus microps	Blueline Tilefish	N	Brought on Board (Retained/Discarded)	Live - Healthy	Retained	Not Applicable	
62	NOCAM	Caulolatilus microps	Blueline Tilefish	N	Brought on Board (Retained/Discarded)	Live - Healthy	Retained	Not Applicable	
62	NOCAM	Caulolatilus microps	Blueline Tilefish	N	Brought on Board (Retained/Discarded)	Live - Healthy	Retained	Not Applicable	
62	NOCAM	Caulolatilus microps	Blueline Tilefish	N	Brought on Board (Retained/Discarded)	Live - Stomach and/or Eyes Protruding	Retained	Not Applicable	
62	NOCAM	Caulolatilus microps	Blueline Tilefish	N	Brought on Board (Retained/Discarded)	Live - Healthy	Retained	Not Applicable	
62	NOCAM	Caulolatilus microps	Blueline Tilefish	N	Brought on Board (Retained/Discarded)	Live - Stomach and/or Eyes Protruding	Retained	Not Applicable	
62	NOCAM	Caulolatilus microps	Blueline Tilefish	N	Brought on Board (Retained/Discarded)	Live - Healthy	Retained	Not Applicable	
62	NOCAM	Caulolatilus microps	Blueline Tilefish	N	Brought on Board (Retained/Discarded)	Live - Healthy	Retained	Not Applicable	
62	NOCAM	Caulolatilus microps	Blueline Tilefish	N	Brought on Board (Retained/Discarded)	Live - Healthy	Retained	Not Applicable	
62	NOCAM	Caulolatilus microps	Blueline Tilefish	N	Brought on Board (Retained/Discarded)	Live - Stomach and/or Eyes Protruding	Retained	Not Applicable	
62	NOCAM	Caulolatilus microps	Blueline Tilefish	N	Brought on Board (Retained/Discarded)	Dead on Arrival - Damaged	Discarded - Dead	Not Applicable	
62	NOCAM	Caulolatilus microps	Blueline Tilefish	N	Brought on Board (Retained/Discarded)	Live - Stomach and/or Eyes Protruding	Retained	Not Applicable	
62	NOCAM	Caulolatilus microps	Blueline Tilefish	N	Brought on Board (Retained/Discarded)	Live - Healthy	Retained	Not Applicable	
62	NOCAM	Caulolatilus microps	Blueline Tilefish	N	Brought on Board (Retained/Discarded)	Live - Healthy	Retained	Not Applicable	
62	NOCAM	Caulolatilus microps	Blueline Tilefish	N	Brought on Board (Retained/Discarded)	Live - Healthy	Retained	Not Applicable	
62	NOCAM	Caulolatilus microps	Blueline Tilefish	N	Brought on Board (Retained/Discarded)	Live - Stomach and/or Eyes Protruding	Retained	Not Applicable	
62	NOCAM	Epinephelus flavolimbatus	Yellowedge Grouper	N	Brought on Board (Retained/Discarded)	Live - Stomach and/or Eyes Protruding	Retained	Not Applicable	
62	NOCAM	Epinephelus flavolimbatus	Yellowedge Grouper	N	Brought on Board (Retained/Discarded)	Live - Stomach and/or Eyes Protruding	Retained	Not Applicable	
62	NOCAM	Epinephelus flavolimbatus	Yellowedge Grouper	N	Brought on Board (Retained/Discarded)	Live - Stomach and/or Eyes Protruding	Retained	Not Applicable	
62	NOCAM	Epinephelus flavolimbatus	Yellowedge Grouper	N	Brought on Board (Retained/Discarded)	Live - Stomach and/or Eyes Protruding	Retained	Not Applicable	
62	NOCAM	Epinephelus flavolimbatus	Yellowedge Grouper	N	Brought on Board (Retained/Discarded)	Live - Stomach and/or Eyes Protruding	Retained	Not Applicable	
62	NOCAM	Epinephelus flavolimbatus	Yellowedge Grouper	N	Brought on Board (Retained/Discarded)	Live - Stomach and/or Eyes Protruding	Retained	Not Applicable	
62	NOCAM	Epinephelus flavolimbatus	Yellowedge Grouper	N	Brought on Board (Retained/Discarded)	Live - Stomach and/or Eyes Protruding	Retained	Not Applicable	
62	NOCAM	Epinephelus flavolimbatus	Yellowedge Grouper	N	Brought on Board (Retained/Discarded)	Live - Stomach and/or Eyes Protruding	Retained	Not Applicable	
62	NOCAM	Epinephelus flavolimbatus	Yellowedge Grouper	N	Brought on Board (Retained/Discarded)	Live - Stomach and/or Eyes Protruding	Retained	Not Applicable	
62	NOCAM	Epinephelus flavolimbatus	Yellowedge Grouper	N	Brought on Board (Retained/Discarded)	Live - Damaged	Retained	Not Applicable	
62	NOCAM	Epinephelus flavolimbatus	Yellowedge Grouper	N	Brought on Board (Retained/Discarded)	Live - Stomach and/or Eyes Protruding	Retained	Not Applicable	
62	NOCAM	Epinephelus flavolimbatus	Yellowedge Grouper	N	Brought on Board (Retained/Discarded)	Live - Stomach and/or Eyes Protruding	Retained	Not Applicable	
62	NOCAM	Epinephelus flavolimbatus	Yellowedge Grouper	N	Brought on Board (Retained/Discarded)	Live - Stomach and/or Eyes Protruding	Retained	Not Applicable	
62	NOCAM	Epinephelus flavolimbatus	Yellowedge Grouper	N	Brought on Board (Retained/Discarded)	Live - Healthy	Retained	Not Applicable	
62	NOCAM	Epinephelus flavolimbatus	Yellowedge Grouper	N	Brought on Board (Retained/Discarded)	Live - Stomach and/or Eyes Protruding	Retained	Not Applicable	
62	NOCAM	Epinephelus flavolimbatus	Yellowedge Grouper	N	Brought on Board (Retained/Discarded)	Live - Stomach and/or Eyes Protruding	Retained	Not Applicable	
62	NOCAM	Epinephelus flavolimbatus	Yellowedge Grouper	N	Brought on Board (Retained/Discarded)	Live - Stomach and/or Eyes Protruding	Retained	Not Applicable	
62	NOCAM	Epinephelus flavolimbatus	Yellowedge Grouper	N	Brought on Board (Retained/Discarded)	Live - Stomach and/or Eyes Protruding	Retained	Not Applicable	
62	NOCAM	Epinephelus flavolimbatus	Yellowedge Grouper	N	Brought on Board (Retained/Discarded)	Live - Stomach and/or Eyes Protruding	Retained	Not Applicable	
62	NOCAM	Mustelus spp.	Dogfish, Smooth (Florida)	Y	Cut-off at Rail (No Entanglement)	Live - Healthy	Discarded - Live and Healthy (Not Vented)	Unknown Maturity and/or Sex	Medium (Between 1m and 2m)
62	UCAM	Acanthocybius solanderi	Wahoo	N	Brought on Board (Retained/Discarded)	Dead on Arrival - Damaged	Discarded - Dead	Not Applicable	
62	UCAM	Caulolatilus microps	Blueline Tilefish	N	Brought on Board (Retained/Discarded)	Live - Healthy	Retained	Not Applicable	
62	UCAM	Caulolatilus microps	Blueline Tilefish	N	Brought on Board (Retained/Discarded)	Live - Healthy	Retained	Not Applicable	
62	UCAM	Caulolatilus microps	Blueline Tilefish	N	Brought on Board (Retained/Discarded)	Live - Healthy	Retained	Not Applicable	
62	UCAM	Caulolatilus microps	Blueline Tilefish	N	Brought on Board (Retained/Discarded)	Live - Stomach and/or Eyes Protruding	Retained	Not Applicable	
62	UCAM	Caulolatilus microps	Blueline Tilefish	N	Brought on Board (Retained/Discarded)	Live - Healthy	Retained	Not Applicable	
62	UCAM	Caulolatilus microps	Blueline Tilefish	N	Brought on Board (Retained/Discarded)	Live - Healthy	Retained	Not Applicable	
62	UCAM	Caulolatilus microps	Blueline Tilefish	N	Brought on Board (Retained/Discarded)	Live - Stomach and/or Eyes Protruding	Retained	Not Applicable	
62	UCAM	Caulolatilus microps	Blueline Tilefish	N	Brought on Board (Retained/Discarded)	Live - Healthy	Retained	Not Applicable	
62	UCAM	Caulolatilus microps	Blueline Tilefish	N	Brought on Board (Retained/Discarded)	Dead on Arrival - Damaged	Discarded - Dead	Not Applicable	
62	UCAM	Caulolatilus microps	Blueline Tilefish	N	Brought on Board (Retained/Discarded)	Live - Healthy	Retained	Not Applicable	
62	UCAM	Caulolatilus microps	Blueline Tilefish	N	Brought on Board (Retained/Discarded)	Live - Stomach and/or Eyes Protruding	Retained	Not Applicable	
62	UCAM	Caulolatilus microps	Blueline Tilefish	N	Brought on Board (Retained/Discarded)	Live - Healthy	Retained	Not Applicable	
62	UCAM	Caulolatilus microps	Blueline Tilefish	N	Brought on Board (Retained/Discarded)	Live - Healthy	Retained	Not Applicable	
62	UCAM	Caulolatilus microps	Blueline Tilefish	N	Brought on Board (Retained/Discarded)	Live - Healthy	Retained	Not Applicable	
62	UCAM	Caulolatilus microps	Blueline Tilefish	N	Brought on Board (Retained/Discarded)	Live - Healthy	Retained	Not Applicable	
62	UCAM	Caulolatilus microps	Blueline Tilefish	N	Brought on Board (Retained/Discarded)	Live - Healthy	Retained	Not Applicable	
62	UCAM	Caulolatilus microps	Blueline Tilefish	N	Brought on Board (Retained/Discarded)	Live - Healthy	Retained	Not Applicable	
62	UCAM	Caulolatilus microps	Blueline Tilefish	N	Brought on Board (Retained/Discarded)	Live - Stomach and/or Eyes Protruding	Retained	Not Applicable	
62	UCAM	Caulolatilus microps	Blueline Tilefish	N	Brought on Board (Retained/Discarded)	Live - Stomach and/or Eyes Protruding	Retained	Not Applicable	
62	UCAM	Epinephelus flavolimbatus	Yellowedge Grouper	N	Brought on Board (Retained/Discarded)	Live - Stomach and/or Eyes Protruding	Retained	Not Applicable	
62	UCAM	Epinephelus flavolimbatus	Yellowedge Grouper	N	Brought on Board (Retained/Discarded)	Live - Healthy	Retained	Not Applicable	
62	UCAM	Epinephelus flavolimbatus	Yellowedge Grouper	N	Brought on Board (Retained/Discarded)	Live - Stomach and/or Eyes Protruding	Retained	Not Applicable	
62	UCAM	Epinephelus flavolimbatus	Yellowedge Grouper	N	Brought on Board (Retained/Discarded)	Live - Stomach and/or Eyes Protruding	Retained	Not Applicable	
62	UCAM	Epinephelus flavolimbatus	Yellowedge Grouper	N	Brought on Board (Retained/Discarded)	Live - Stomach and/or Eyes Protruding	Retained	Not Applicable	
62	UCAM	Epinephelus flavolimbatus	Yellowedge Grouper	N	Brought on Board (Retained/Discarded)	Live - Stomach and/or Eyes Protruding	Retained	Not Applicable	
62	UCAM	Epinephelus flavolimbatus	Yellowedge Grouper	N	Brought on Board (Retained/Discarded)	Live - Stomach and/or Eyes Protruding	Retained	Not Applicable	
62	UCAM	Epinephelus flavolimbatus	Yellowedge Grouper	N	Brought on Board (Retained/Discarded)	Live - Stomach and/or Eyes Protruding	Retained	Not Applicable	
62	UCAM	Epinephelus flavolimbatus	Yellowedge Grouper	N	Brought on Board (Retained/Discarded)	Live - Stomach and/or Eyes Protruding	Retained	Not Applicable	
62	UCAM	Epinephelus flavolimbatus	Yellowedge Grouper	N	Brought on Board (Retained/Discarded)	Live - Stomach and/or Eyes Protruding	Retained	Not Applicable	
62	UCAM	Epinephelus flavolimbatus	Yellowedge Grouper	N	Brought on Board (Retained/Discarded)	Live - Stomach and/or Eyes Protruding	Retained	Not Applicable	
62	UCAM	Epinephelus flavolimbatus	Yellowedge Grouper	N	Brought on Board (Retained/Discarded)	Live - Stomach and/or Eyes Protruding	Retained	Not Applicable	
62	UCAM	Epinephelus flavolimbatus	Yellowedge Grouper	N	Brought on Board (Retained/Discarded)	Live - Stomach and/or Eyes Protruding	Retained	Not Applicable	
62	UCAM	Epinephelus flavolimbatus	Yellowedge Grouper	N	Brought on Board (Retained/Discarded)	Live - Stomach and/or Eyes Protruding	Retained	Not Applicable	
62	UCAM	Epinephelus flavolimbatus	Yellowedge Grouper	N	Brought on Board (Retained/Discarded)	Live - Healthy	Retained	Not Applicable	
62	UCAM	Epinephelus flavolimbatus	Yellowedge Grouper	N	Brought on Board (Retained/Discarded)	Live - Stomach and/or Eyes Protruding	Retained	Not Applicable	
62	UCAM	Epinephelus flavolimbatus	Yellowedge Grouper	N	Brought on Board (Retained/Discarded)	Live - Stomach and/or Eyes Protruding	Retained	Not Applicable	
62	UCAM	Epinephelus flavolimbatus	Yellowedge Grouper	N	Brought on Board (Retained/Discarded)	Live - Stomach and/or Eyes Protruding	Retained	Not Applicable	
62	UCAM	Epinephelus flavolimbatus	Yellowedge Grouper	N	Brought on Board (Retained/Discarded)	Live - Stomach and/or Eyes Protruding	Retained	Not Applicable	
62	UCAM	Epinephelus flavolimbatus	Yellowedge Grouper	N	Brought on Board (Retained/Discarded)	Live - Stomach and/or Eyes Protruding	Retained	Not Applicable	

62	UCAM	Mustelus spp.	Dogfish, Smooth (Florida)	Y	Cut-off at Rail (No Entanglement)	Live - Healthy	Discarded - Live and Healthy (Not Vented)	Unknown Maturity and/or Sex	Medium (Between 1m and 2m)
63	NOCAM	Acanthocybius solanderi	Wahoo	N	Brought on Board (Retained/Discarded)	Dead on Arrival - Undamaged	Retained	Not Applicable	
63	NOCAM	Brotula barbata	Bearded Brotula	N	Brought on Board (Retained/Discarded)	Live - Healthy	Retained	Not Applicable	
63	NOCAM	Brotula barbata	Bearded Brotula	N	Brought on Board (Retained/Discarded)	Live - Stomach and/or Eyes Protruding	Retained	Not Applicable	
63	NOCAM	Carcharhinus falciformis	Silky Shark	Y	Cut-off at Rail (No Entanglement)	Live - Healthy	Discarded - Live and Healthy (Not Vented)	Unknown Maturity and/or Sex	Medium (Between 1m and 2m)
63	NOCAM	Epinephelus flavolimbatus	Yellowedge Grouper	N	Brought on Board (Retained/Discarded)	Live - Stomach and/or Eyes Protruding	Retained	Not Applicable	
63	NOCAM	Epinephelus flavolimbatus	Yellowedge Grouper	N	Brought on Board (Retained/Discarded)	Live - Stomach and/or Eyes Protruding	Retained	Not Applicable	
63	NOCAM	Epinephelus flavolimbatus	Yellowedge Grouper	N	Brought on Board (Retained/Discarded)	Live - Stomach and/or Eyes Protruding	Retained	Not Applicable	
63	NOCAM	Epinephelus flavolimbatus	Yellowedge Grouper	N	Brought on Board (Retained/Discarded)	Dead on Arrival - Undamaged	Retained	Not Applicable	
63	NOCAM	Epinephelus flavolimbatus	Yellowedge Grouper	N	Brought on Board (Retained/Discarded)	Dead on Arrival - Undamaged	Retained	Not Applicable	
63	NOCAM	Epinephelus flavolimbatus	Yellowedge Grouper	N	Brought on Board (Retained/Discarded)	Live - Stomach and/or Eyes Protruding	Retained	Not Applicable	
63	NOCAM	Epinephelus flavolimbatus	Yellowedge Grouper	N	Brought on Board (Retained/Discarded)	Live - Healthy	Retained	Not Applicable	
63	NOCAM	Epinephelus flavolimbatus	Yellowedge Grouper	N	Brought on Board (Retained/Discarded)	Live - Stomach and/or Eyes Protruding	Retained	Not Applicable	
63	NOCAM	Epinephelus flavolimbatus	Yellowedge Grouper	N	Brought on Board (Retained/Discarded)	Live - Stomach and/or Eyes Protruding	Retained	Not Applicable	
63	NOCAM	Epinephelus flavolimbatus	Yellowedge Grouper	N	Brought on Board (Retained/Discarded)	Live - Stomach and/or Eyes Protruding	Retained	Not Applicable	
63	NOCAM	Epinephelus flavolimbatus	Yellowedge Grouper	N	Brought on Board (Retained/Discarded)	Live - Stomach and/or Eyes Protruding	Retained	Not Applicable	
63	NOCAM	Epinephelus flavolimbatus	Yellowedge Grouper	N	Brought on Board (Retained/Discarded)	Live - Stomach and/or Eyes Protruding	Retained	Not Applicable	
63	NOCAM	Epinephelus flavolimbatus	Yellowedge Grouper	N	Brought on Board (Retained/Discarded)	Live - Stomach and/or Eyes Protruding	Retained	Not Applicable	
63	NOCAM	Epinephelus flavolimbatus	Yellowedge Grouper	N	Brought on Board (Retained/Discarded)	Live - Stomach and/or Eyes Protruding	Retained	Not Applicable	
63	NOCAM	Epinephelus flavolimbatus	Yellowedge Grouper	N	Brought on Board (Retained/Discarded)	Live - Stomach and/or Eyes Protruding	Retained	Not Applicable	
63	NOCAM	Epinephelus niveatus	Snowy Grouper	N	Brought on Board (Retained/Discarded)	Live - Healthy	Retained	Not Applicable	
63	NOCAM	Epinephelus niveatus	Snowy Grouper	N	Brought on Board (Retained/Discarded)	Live - Stomach and/or Eyes Protruding	Retained	Not Applicable	
63	NOCAM	Epinephelus niveatus	Snowy Grouper	N	Brought on Board (Retained/Discarded)	Live - Healthy	Retained	Not Applicable	
63	NOCAM	Epinephelus niveatus	Snowy Grouper	N	Brought on Board (Retained/Discarded)	Live - Healthy	Retained	Not Applicable	
63	NOCAM	Epinephelus niveatus	Snowy Grouper	N	Brought on Board (Retained/Discarded)	Live - Stomach and/or Eyes Protruding	Retained	Not Applicable	
63	NOCAM	Epinephelus niveatus	Snowy Grouper	N	Brought on Board (Retained/Discarded)	Live - Stomach and/or Eyes Protruding	Retained	Not Applicable	
63	NOCAM	Epinephelus niveatus	Snowy Grouper	N	Brought on Board (Retained/Discarded)	Live - Stomach and/or Eyes Protruding	Retained	Not Applicable	
63	NOCAM	Epinephelus niveatus	Snowy Grouper	N	Brought on Board (Retained/Discarded)	Live - Healthy	Retained	Not Applicable	
63	NOCAM	Gadiformes, unidentified	Hake, Unidentified	N	Brought on Board (Retained/Discarded)	Live - Healthy	Retained as Bait	Not Applicable	
63	NOCAM	Mustelus spp.	Dogfish, Smooth (Florida)	Y	Brought on Board (Retained/Discarded)	Live - Healthy	Discarded - Live and Healthy (Not Vented)	Female	Small (Less than 1m)
63	NOCAM	Mustelus spp.	Dogfish, Smooth (Florida)	Y	Brought on Board (Retained/Discarded)	Live - Healthy	Discarded - Live and Healthy (Not Vented)	Male (Claspers)	Small (Less than 1m)
63	NOCAM	Mustelus spp.	Dogfish, Smooth (Florida)	Y	Brought on Board (Retained/Discarded)	Live - Healthy	Discarded - Live and Healthy (Not Vented)	Male (Claspers)	Small (Less than 1m)
63	NOCAM	Mustelus spp.	Dogfish, Smooth (Florida)	Y	Brought on Board (Retained/Discarded)	Live - Healthy	Discarded - Live and Healthy (Not Vented)	Male (Claspers)	Small (Less than 1m)
63	NOCAM	Mustelus spp.	Dogfish, Smooth (Florida)	Y	Brought on Board (Retained/Discarded)	Live - Healthy	Discarded - Live and Healthy (Not Vented)	Female	Small (Less than 1m)
63	NOCAM	Mustelus spp.	Dogfish, Smooth (Florida)	Y	Brought on Board (Retained/Discarded)	Live - Healthy	Discarded - Live and Healthy (Not Vented)	Male (Claspers)	Small (Less than 1m)
63	NOCAM	Mustelus spp.	Dogfish, Smooth (Florida)	Y	Not Handled (Dropped Off)	Live - Healthy	Discarded - Live and Healthy (Not Vented)	Unknown Maturity and/or Sex	Small (Less than 1m)
63	NOCAM	Mustelus spp.	Dogfish, Smooth (Florida)	Y	Brought on Board (Retained/Discarded)	Live - Healthy	Discarded - Live and Healthy (Not Vented)	Male (Claspers)	Medium (Between 1m and 2m)
63	NOCAM	Mustelus spp.	Dogfish, Smooth (Florida)	Y	Brought on Board (Retained/Discarded)	Live - Healthy	Discarded - Live and Healthy (Not Vented)	Unknown Maturity and/or Sex	Small (Less than 1m)
63	NOCAM	Mustelus spp.	Dogfish, Smooth (Florida)	Y	Brought on Board (Retained/Discarded)	Live - Healthy	Discarded - Live and Healthy (Not Vented)	Male (Claspers)	Medium (Between 1m and 2m)
63	NOCAM	Mycteroperca microlepis	Gag Grouper	N	Brought on Board (Retained/Discarded)	Live - Healthy	Retained	Not Applicable	
63	NOCAM	Mycteroperca microlepis	Gag Grouper	N	Brought on Board (Retained/Discarded)	Live - Healthy	Retained	Not Applicable	
63	NOCAM	Mycteroperca microlepis	Gag Grouper	N	Brought on Board (Retained/Discarded)	Live - Healthy	Retained	Not Applicable	
63	NOCAM	Mycteroperca microlepis	Gag Grouper	N	Brought on Board (Retained/Discarded)	Live - Healthy	Retained	Not Applicable	
63	NOCAM	Mycteroperca microlepis	Gag Grouper	N	Brought on Board (Retained/Discarded)	Live - Healthy	Retained	Not Applicable	
63	NOCAM	Mycteroperca microlepis	Gag Grouper	N	Brought on Board (Retained/Discarded)	Live - Healthy	Retained	Not Applicable	
63	NOCAM	Mycteroperca microlepis	Gag Grouper	N	Brought on Board (Retained/Discarded)	Live - Healthy	Retained	Not Applicable	
63	NOCAM	Mycteroperca microlepis	Gag Grouper	N	Brought on Board (Retained/Discarded)	Live - Stomach and/or Eyes Protruding	Retained	Not Applicable	
63	NOCAM	Neomerinthe hemingwayi	Scorpionfish, Spiny Cheek	N	Brought on Board (Retained/Discarded)	Live - Stomach and/or Eyes Protruding	Retained	Not Applicable	
63	NOCAM	Neomerinthe hemingwayi	Scorpionfish, Spiny Cheek	N	Brought on Board (Retained/Discarded)	Live - Stomach and/or Eyes Protruding	Retained	Not Applicable	
63	NOCAM	Neomerinthe hemingwayi	Scorpionfish, Spiny Cheek	N	Brought on Board (Retained/Discarded)	Live - Stomach and/or Eyes Protruding	Retained	Not Applicable	
63	NOCAM	Neomerinthe hemingwayi	Scorpionfish, Spiny Cheek	N	Brought on Board (Retained/Discarded)	Live - Stomach and/or Eyes Protruding	Retained	Not Applicable	
63	NOCAM	Neomerinthe hemingwayi	Scorpionfish, Spiny Cheek	N	Brought on Board (Retained/Discarded)	Live - Stomach and/or Eyes Protruding	Retained	Not Applicable	
63	NOCAM	Neomerinthe hemingwayi	Scorpionfish, Spiny Cheek	N	Brought on Board (Retained/Discarded)	Live - Stomach and/or Eyes Protruding	Retained	Not Applicable	
63	NOCAM	Neomerinthe hemingwayi	Scorpionfish, Spiny Cheek	N	Brought on Board (Retained/Discarded)	Live - Stomach and/or Eyes Protruding	Retained	Not Applicable	
63	NOCAM	Neomerinthe hemingwayi	Scorpionfish, Spiny Cheek	N	Brought on Board (Retained/Discarded)	Live - Healthy	Retained	Not Applicable	
63	NOCAM	Pagrus pagrus	Red Porgy	N	Brought on Board (Retained/Discarded)	Live - Healthy	Retained	Not Applicable	
63	NOCAM	Pagrus pagrus	Red Porgy	N	Brought on Board (Retained/Discarded)	Live - Healthy	Retained	Not Applicable	
63	NOCAM	Pagrus pagrus	Red Porgy	N	Brought on Board (Retained/Discarded)	Live - Healthy	Retained	Not Applicable	
63	NOCAM	Selachimorpha, unidentified	Shark, Unidentified	Y	Cut-off at Rail (No Entanglement)	Dead on Arrival - Undamaged	Discarded - Dead	Unknown Maturity and/or Sex	Medium (Between 1m and 2m)
63	NOCAM	Seriola dumerili	Greater Amberjack	N	Brought on Board (Retained/Discarded)	Live - Healthy	Discarded - Live and Healthy (Vented)	Not Applicable	
63	NOCAM	Seriola dumerili	Greater Amberjack	N	Brought on Board (Retained/Discarded)	Live - Healthy	Discarded - Live and Healthy (Not Vented)	Not Applicable	
63	NOCAM	Seriola dumerili	Greater Amberjack	N	Brought on Board (Retained/Discarded)	Live - Healthy	Discarded - Live and Healthy (Vented)	Not Applicable	
63	NOCAM	Seriola dumerili	Greater Amberjack	N	Brought on Board (Retained/Discarded)	Live - Healthy	Discarded - Live and Healthy (Vented)	Not Applicable	
63	NOCAM	Seriola dumerili	Greater Amberjack	N	Brought on Board (Retained/Discarded)	Live - Healthy	Discarded - Live and Healthy (Vented)	Not Applicable	
63	NOCAM	Seriola dumerili	Greater Amberjack	N	Brought on Board (Retained/Discarded)	Live - Healthy	Discarded - Live and Healthy (Vented)	Not Applicable	
63	NOCAM	Seriola dumerili	Greater Amberjack	N	Brought on Board (Retained/Discarded)	Live - Healthy	Discarded - Live and Healthy (Vented)	Not Applicable	
63	NOCAM	Seriola dumerili	Greater Amberjack	N	Brought on Board (Retained/Discarded)	Live - Healthy	Discarded - Live and Healthy (Vented)	Not Applicable	
63	NOCAM	Sphyrna lewini	Scalloped Hammerhead	Y	Cut-off at Rail (No Entanglement)	Dead on Arrival - Undamaged	Discarded - Dead	Unknown Maturity and/or Sex	Medium (Between 1m and 2m)
63	NOCAM	Sphyrna lewini	Scalloped Hammerhead	Y	Cut-off at Rail (No Entanglement)	Live - Healthy	Discarded - Live and Healthy (Not Vented)	Unknown Maturity and/or Sex	Medium (Between 1m and 2m)
63	NOCAM	Thunnus atlanticus	Blackfin Tuna	N	Brought on Board (Retained/Discarded)	Dead on Arrival - Damaged	Discarded - Dead	Not Applicable	
63	NOCAM	Thunnus atlanticus	Blackfin Tuna	N	Brought on Board (Retained/Discarded)	Dead on Arrival - Undamaged	Discarded - Dead	Not Applicable	
63	NOCAM	Thunnus atlanticus	Blackfin Tuna	N	Brought on Board (Retained/Discarded)	Dead on Arrival - Undamaged	Discarded - Dead	Not Applicable	
63	UCAM	Acanthocybius solanderi	Wahoo	N	Brought on Board (Retained/Discarded)	Dead on Arrival - Undamaged	Retained	Not Applicable	
63	UCAM	Brotula barbata	Bearded Brotula	N	Brought on Board (Retained/Discarded)	Live - Healthy	Retained	Not Applicable	
63	UCAM	Brotula barbata	Bearded Brotula	N	Brought on Board (Retained/Discarded)	Live - Healthy	Retained	Not Applicable	
63	UCAM	Carcharhinus falciformis	Silky Shark	Y	Cut-off at Rail (No Entanglement)	Live - Healthy	Discarded - Live and Healthy (Not Vented)	Female	Medium (Between 1m and 2m)
63	UCAM	Epinephelus flavolimbatus	Yellowedge Grouper	N	Brought on Board (Retained/Discarded)	Live - Healthy	Retained	Not Applicable	
63	UCAM	Epinephelus flavolimbatus	Yellowedge Grouper	N	Brought on Board (Retained/Discarded)	Live - Stomach and/or Eyes Protruding	Retained	Not Applicable	
63	UCAM	Epinephelus flavolimbatus	Yellowedge Grouper	N	Brought on Board (Retained/Discarded)	Live - Stomach and/or Eyes Protruding	Retained	Not Applicable	
63	UCAM	Epinephelus flavolimbatus	Yellowedge Grouper	N	Brought on Board (Retained/Discarded)	Live - Stomach and/or Eyes Protruding	Retained	Not Applicable	
63	UCAM	Epinephelus flavolimbatus	Yellowedge Grouper	N	Brought on Board (Retained/Discarded)	Live - Stomach and/or Eyes Protruding	Retained	Not Applicable	
63	UCAM	Epinephelus flavolimbatus	Yellowedge Grouper	N	Brought on Board (Retained/Discarded)	Live - Stomach and/or Eyes Protruding	Retained	Not Applicable	
63	UCAM	Epinephelus flavolimbatus	Yellowedge Grouper	N	Brought on Board (Retained/Discarded)	Live - Stomach and/or Eyes Protruding	Retained	Not Applicable	
63	UCAM	Epinephelus flavolimbatus	Yellowedge Grouper	N	Brought on Board (Retained/Discarded)	Live - Stomach and/or Eyes Protruding	Retained	Not Applicable	
63	UCAM	Epinephelus flavolimbatus	Yellowedge Grouper	N	Brought on Board (Retained/Discarded)	Live - Stomach and/or Eyes Protruding	Retained	Not Applicable	
63	UCAM	Epinephelus flavolimbatus	Yellowedge Grouper	N	Brought on Board (Retained/Discarded)	Live - Healthy	Retained	Not Applicable	
63	UCAM	Epinephelus flavolimbatus	Yellowedge Grouper	N	Brought on Board (Retained/Discarded)	Live - Stomach and/or Eyes Protruding	Retained	Not Applicable	
63	UCAM	Epinephelus flavolimbatus	Yellowedge Grouper	N	Brought on Board (Retained/Discarded)	Live - Healthy	Retained	Not Applicable	
63	UCAM	Epinephelus flavolimbatus	Yellowedge Grouper	N	Brought on Board (Retained/Discarded)	Dead on Arrival - Undamaged	Retained	Not Applicable	
63	UCAM	Epinephelus flavolimbatus	Yellowedge Grouper	N	Brought on Board (Retained/Discarded)	Live - Stomach and/or Eyes Protruding	Retained	Not Applicable	
63	UCAM	Epinephelus flavolimbatus	Yellowedge Grouper	N	Brought on Board (Retained/Discarded)	Live - Stomach and/or Eyes Protruding	Retained	Not Applicable	
63	UCAM	Epinephelus flavolimbatus	Yellowedge Grouper	N	Brought on Board (Retained/Discarded)	Live - Stomach and/or Eyes Protruding	Retained	Not Applicable	

63	UCAM	Epinephelus niveatus	Snowy Grouper	N	Brought on Board (Retained/Discarded)	Live - Stomach and/or Eyes Protruding	Retained	Not Applicable	
63	UCAM	Epinephelus niveatus	Snowy Grouper	N	Brought on Board (Retained/Discarded)	Live - Stomach and/or Eyes Protruding	Retained	Not Applicable	
63	UCAM	Epinephelus niveatus	Snowy Grouper	N	Brought on Board (Retained/Discarded)	Live - Stomach and/or Eyes Protruding	Retained	Not Applicable	
63	UCAM	Epinephelus niveatus	Snowy Grouper	N	Brought on Board (Retained/Discarded)	Live - Stomach and/or Eyes Protruding	Retained	Not Applicable	
63	UCAM	Epinephelus niveatus	Snowy Grouper	N	Brought on Board (Retained/Discarded)	Live - Stomach and/or Eyes Protruding	Retained	Not Applicable	
63	UCAM	Epinephelus niveatus	Snowy Grouper	N	Brought on Board (Retained/Discarded)	Live - Healthy	Retained	Not Applicable	
63	UCAM	Epinephelus niveatus	Snowy Grouper	N	Brought on Board (Retained/Discarded)	Live - Stomach and/or Eyes Protruding	Retained	Not Applicable	
63	UCAM	Epinephelus niveatus	Snowy Grouper	N	Brought on Board (Retained/Discarded)	Live - Healthy	Retained	Not Applicable	
63	UCAM	Gadiformes, unidentified	Hake, Unidentified	N	Brought on Board (Retained/Discarded)	Live - Healthy	Retained as Bait	Not Applicable	
63	UCAM	Muraenidae, unidentified	Moray Eel, Unidentified	N	Brought on Board (Retained/Discarded)	Live - Healthy	Retained as Bait	Not Applicable	
63	UCAM	Mustelus spp.	Dogfish, Smooth (Florida)	Y	Brought on Board (Retained/Discarded)	Live - Healthy	Discarded - Live and Healthy (Not Vented)	Male (Claspers)	Medium (Between 1m and 2m)
63	UCAM	Mustelus spp.	Dogfish, Smooth (Florida)	Y	Not Handled (Dropped Off)	Live - Healthy	Discarded - Live and Healthy (Not Vented)	Female	Small (Less than 1m)
63	UCAM	Mustelus spp.	Dogfish, Smooth (Florida)	Y	Brought on Board (Retained/Discarded)	Live - Healthy	Discarded - Live and Healthy (Not Vented)	Male (Claspers)	Small (Less than 1m)
63	UCAM	Mustelus spp.	Dogfish, Smooth (Florida)	Y	Brought on Board (Retained/Discarded)	Live - Healthy	Discarded - Live and Healthy (Not Vented)	Female	Small (Less than 1m)
63	UCAM	Mustelus spp.	Dogfish, Smooth (Florida)	Y	Brought on Board (Retained/Discarded)	Live - Healthy	Discarded - Live and Healthy (Not Vented)	Male (Claspers)	Small (Less than 1m)
63	UCAM	Mustelus spp.	Dogfish, Smooth (Florida)	Y	Brought on Board (Retained/Discarded)	Live - Healthy	Discarded - Live and Healthy (Not Vented)	Male (Claspers)	Medium (Between 1m and 2m)
63	UCAM	Mustelus spp.	Dogfish, Smooth (Florida)	Y	Brought on Board (Retained/Discarded)	Live - Healthy	Discarded - Live and Healthy (Not Vented)	Unknown Maturity and/or Sex	Small (Less than 1m)
63	UCAM	Mustelus spp.	Dogfish, Smooth (Florida)	Y	Brought on Board (Retained/Discarded)	Live - Healthy	Discarded - Live and Healthy (Not Vented)	Male (Claspers)	Small (Less than 1m)
63	UCAM	Mustelus spp.	Dogfish, Smooth (Florida)	Y	Brought on Board (Retained/Discarded)	Live - Healthy	Discarded - Live and Healthy (Not Vented)	Male (Claspers)	Small (Less than 1m)
63	UCAM	Mustelus spp.	Dogfish, Smooth (Florida)	Y	Brought on Board (Retained/Discarded)	Live - Healthy	Discarded - Live and Healthy (Not Vented)	Female	Small (Less than 1m)
63	UCAM	Mycteroperca microlepis	Gag Grouper	N	Brought on Board (Retained/Discarded)	Live - Stomach and/or Eyes Protruding	Retained	Not Applicable	
63	UCAM	Mycteroperca microlepis	Gag Grouper	N	Brought on Board (Retained/Discarded)	Live - Healthy	Retained	Not Applicable	
63	UCAM	Mycteroperca microlepis	Gag Grouper	N	Brought on Board (Retained/Discarded)	Live - Stomach and/or Eyes Protruding	Retained	Not Applicable	
63	UCAM	Mycteroperca microlepis	Gag Grouper	N	Brought on Board (Retained/Discarded)	Live - Healthy	Retained	Not Applicable	
63	UCAM	Mycteroperca microlepis	Gag Grouper	N	Brought on Board (Retained/Discarded)	Live - Healthy	Retained	Not Applicable	
63	UCAM	Mycteroperca microlepis	Gag Grouper	N	Brought on Board (Retained/Discarded)	Live - Healthy	Retained	Not Applicable	
63	UCAM	Mycteroperca microlepis	Gag Grouper	N	Brought on Board (Retained/Discarded)	Live - Healthy	Retained	Not Applicable	
63	UCAM	Mycteroperca microlepis	Gag Grouper	N	Brought on Board (Retained/Discarded)	Live - Healthy	Retained	Not Applicable	
63	UCAM	Neomerinthe hemingwayi	Scorpionfish, Spiny Cheek	N	Brought on Board (Retained/Discarded)	Live - Stomach and/or Eyes Protruding	Retained	Not Applicable	
63	UCAM	Neomerinthe hemingwayi	Scorpionfish, Spiny Cheek	N	Brought on Board (Retained/Discarded)	Live - Stomach and/or Eyes Protruding	Retained	Not Applicable	
63	UCAM	Neomerinthe hemingwayi	Scorpionfish, Spiny Cheek	N	Brought on Board (Retained/Discarded)	Live - Healthy	Retained	Not Applicable	
63	UCAM	Neomerinthe hemingwayi	Scorpionfish, Spiny Cheek	N	Brought on Board (Retained/Discarded)	Live - Stomach and/or Eyes Protruding	Retained	Not Applicable	
63	UCAM	Neomerinthe hemingwayi	Scorpionfish, Spiny Cheek	N	Brought on Board (Retained/Discarded)	Live - Stomach and/or Eyes Protruding	Retained	Not Applicable	
63	UCAM	Neomerinthe hemingwayi	Scorpionfish, Spiny Cheek	N	Brought on Board (Retained/Discarded)	Live - Stomach and/or Eyes Protruding	Retained	Not Applicable	
63	UCAM	Neomerinthe hemingwayi	Scorpionfish, Spiny Cheek	N	Brought on Board (Retained/Discarded)	Live - Stomach and/or Eyes Protruding	Retained	Not Applicable	
63	UCAM	Neomerinthe hemingwayi	Scorpionfish, Spiny Cheek	N	Brought on Board (Retained/Discarded)	Live - Stomach and/or Eyes Protruding	Retained	Not Applicable	
63	UCAM	Pagrus pagrus	Red Porgy	N	Brought on Board (Retained/Discarded)	Live - Healthy	Retained	Not Applicable	
63	UCAM	Pagrus pagrus	Red Porgy	N	Brought on Board (Retained/Discarded)	Live - Healthy	Retained	Not Applicable	
63	UCAM	Pagrus pagrus	Red Porgy	N	Brought on Board (Retained/Discarded)	Live - Healthy	Retained	Not Applicable	
63	UCAM	Selachimorpha, unidentified	Shark, Unidentified	Y	Brought on Board (Retained/Discarded)	Dead on Arrival - Undamaged	Discarded - Dead	Unknown Maturity and/or Sex	Medium (Between 1m and 2m)
63	UCAM	Seriola dumerili	Greater Amberjack	N	Brought on Board (Retained/Discarded)	Live - Healthy	Discarded - Live and Healthy (Vented)	Not Applicable	
63	UCAM	Seriola dumerili	Greater Amberjack	N	Brought on Board (Retained/Discarded)	Live - Healthy	Discarded - Live and Healthy (Vented)	Not Applicable	
63	UCAM	Seriola dumerili	Greater Amberjack	N	Brought on Board (Retained/Discarded)	Live - Healthy	Discarded - Live and Healthy (Vented)	Not Applicable	
63	UCAM	Seriola dumerili	Greater Amberjack	N	Brought on Board (Retained/Discarded)	Live - Healthy	Discarded - Live and Healthy (Not Vented)	Not Applicable	
63	UCAM	Seriola dumerili	Greater Amberjack	N	Brought on Board (Retained/Discarded)	Live - Healthy	Discarded - Live and Healthy (Vented)	Not Applicable	
63	UCAM	Seriola dumerili	Greater Amberjack	N	Brought on Board (Retained/Discarded)	Live - Healthy	Discarded - Live and Healthy (Vented)	Not Applicable	
63	UCAM	Seriola dumerili	Greater Amberjack	N	Brought on Board (Retained/Discarded)	Live - Healthy	Discarded - Live and Healthy (Vented)	Not Applicable	
63	UCAM	Seriola dumerili	Greater Amberjack	N	Brought on Board (Retained/Discarded)	Live - Healthy	Discarded - Live and Healthy (Vented)	Not Applicable	
63	UCAM	Sphyrna lewini	Scalloped Hammerhead	Y	Cut-off at Rail (No Entanglement)	Live - Healthy	Discarded - Live and Healthy (Not Vented)	Female	Medium (Between 1m and 2m)
63	UCAM	Sphyrna lewini	Scalloped Hammerhead	Y	Cut-off at Rail (No Entanglement)	Live - Healthy	Discarded - Live and Healthy (Not Vented)	Female	Medium (Between 1m and 2m)
63	UCAM	Thunnus atlanticus	Blackfin Tuna	N	Brought on Board (Retained/Discarded)	Dead on Arrival - Undamaged	Discarded - Dead	Not Applicable	
63	UCAM	Thunnus atlanticus	Blackfin Tuna	N	Brought on Board (Retained/Discarded)	Dead on Arrival - Damaged	Discarded - Dead	Not Applicable	
63	UCAM	Thunnus atlanticus	Blackfin Tuna	N	Brought on Board (Retained/Discarded)	Dead on Arrival - Undamaged	Discarded - Dead	Not Applicable	
64	NOCAM	Caulolatilus microps	Blueline Tilefish	N	Brought on Board (Retained/Discarded)	Live - Stomach and/or Eyes Protruding	Retained	Not Applicable	
64	NOCAM	Caulolatilus microps	Blueline Tilefish	N	Brought on Board (Retained/Discarded)	Live - Stomach and/or Eyes Protruding	Retained	Not Applicable	
64	NOCAM	Caulolatilus microps	Blueline Tilefish	N	Brought on Board (Retained/Discarded)	Live - Healthy	Retained	Not Applicable	
64	NOCAM	Caulolatilus microps	Blueline Tilefish	N	Brought on Board (Retained/Discarded)	Live - Stomach and/or Eyes Protruding	Retained	Not Applicable	
64	NOCAM	Caulolatilus microps	Blueline Tilefish	N	Brought on Board (Retained/Discarded)	Live - Stomach and/or Eyes Protruding	Retained	Not Applicable	
64	NOCAM	Caulolatilus microps	Blueline Tilefish	N	Brought on Board (Retained/Discarded)	Live - Healthy	Retained	Not Applicable	
64	NOCAM	Caulolatilus microps	Blueline Tilefish	N	Brought on Board (Retained/Discarded)	Live - Healthy	Retained	Not Applicable	
64	NOCAM	Caulolatilus microps	Blueline Tilefish	N	Brought on Board (Retained/Discarded)	Live - Healthy	Retained	Not Applicable	
64	NOCAM	Caulolatilus microps	Blueline Tilefish	N	Brought on Board (Retained/Discarded)	Live - Healthy	Retained	Not Applicable	
64	NOCAM	Caulolatilus microps	Blueline Tilefish	N	Brought on Board (Retained/Discarded)	Live - Healthy	Retained	Not Applicable	
64	NOCAM	Caulolatilus microps	Blueline Tilefish	N	Brought on Board (Retained/Discarded)	Live - Stomach and/or Eyes Protruding	Retained	Not Applicable	
64	NOCAM	Caulolatilus microps	Blueline Tilefish	N	Brought on Board (Retained/Discarded)	Live - Healthy	Retained	Not Applicable	
64	NOCAM	Caulolatilus microps	Blueline Tilefish	N	Brought on Board (Retained/Discarded)	Live - Healthy	Retained	Not Applicable	
64	NOCAM	Caulolatilus microps	Blueline Tilefish	N	Brought on Board (Retained/Discarded)	Live - Healthy	Retained	Not Applicable	
64	NOCAM	Caulolatilus microps	Blueline Tilefish	N	Brought on Board (Retained/Discarded)	Live - Healthy	Retained	Not Applicable	
64	NOCAM	Caulolatilus microps	Blueline Tilefish	N	Brought on Board (Retained/Discarded)	Live - Healthy	Retained	Not Applicable	
64	NOCAM	Caulolatilus microps	Blueline Tilefish	N	Brought on Board (Retained/Discarded)	Live - Healthy	Retained	Not Applicable	
64	NOCAM	Caulolatilus microps	Blueline Tilefish	N	Brought on Board (Retained/Discarded)	Live - Healthy	Retained	Not Applicable	
64	NOCAM	Caulolatilus microps	Blueline Tilefish	N	Brought on Board (Retained/Discarded)	Live - Healthy	Retained	Not Applicable	
64	NOCAM	Caulolatilus microps	Blueline Tilefish	N	Brought on Board (Retained/Discarded)	Live - Healthy	Retained	Not Applicable	
64	NOCAM	Epinephelus flavolimbatus	Yellowedge Grouper	N	Brought on Board (Retained/Discarded)	Live - Stomach and/or Eyes Protruding	Retained	Not Applicable	
64	NOCAM	Epinephelus flavolimbatus	Yellowedge Grouper	N	Brought on Board (Retained/Discarded)	Live - Stomach and/or Eyes Protruding	Retained	Not Applicable	
64	NOCAM	Epinephelus niveatus	Snowy Grouper	N	Brought on Board (Retained/Discarded)	Live - Healthy	Retained	Not Applicable	
64	UCAM	Caulolatilus microps	Blueline Tilefish	N	Brought on Board (Retained/Discarded)	Live - Healthy	Retained	Not Applicable	
64	UCAM	Caulolatilus microps	Blueline Tilefish	N	Brought on Board (Retained/Discarded)	Live - Healthy	Retained	Not Applicable	
64	UCAM	Caulolatilus microps	Blueline Tilefish	N	Brought on Board (Retained/Discarded)	Live - Healthy	Retained	Not Applicable	
64	UCAM	Caulolatilus microps	Blueline Tilefish	N	Brought on Board (Retained/Discarded)	Live - Healthy	Retained	Not Applicable	
64	UCAM	Caulolatilus microps	Blueline Tilefish	N	Brought on Board (Retained/Discarded)	Live - Healthy	Retained	Not Applicable	
64	UCAM	Caulolatilus microps	Blueline Tilefish	N	Brought on Board (Retained/Discarded)	Live - Healthy	Retained	Not Applicable	
64	UCAM	Caulolatilus microps	Blueline Tilefish	N	Brought on Board (Retained/Discarded)	Live - Healthy	Retained	Not Applicable	
64	UCAM	Caulolatilus microps	Blueline Tilefish	N	Brought on Board (Retained/Discarded)	Live - Healthy	Retained	Not Applicable	
64	UCAM	Caulolatilus microps	Blueline Tilefish	N	Brought on Board (Retained/Discarded)	Live - Stomach and/or Eyes Protruding	Retained	Not Applicable	
64	UCAM	Caulolatilus microps	Blueline Tilefish	N	Brought on Board (Retained/Discarded)	Live - Healthy	Retained	Not Applicable	
64	UCAM	Caulolatilus microps	Blueline Tilefish	N	Brought on Board (Retained/Discarded)	Live - Healthy	Retained	Not Applicable	
64	UCAM	Caulolatilus microps	Blueline Tilefish	N	Brought on Board (Retained/Discarded)	Live - Stomach and/or Eyes Protruding	Retained	Not Applicable	
64	UCAM	Caulolatilus microps	Blueline Tilefish	N	Brought on Board (Retained/Discarded)	Live - Healthy	Retained	Not Applicable	
64	UCAM	Caulolatilus microps	Blueline Tilefish	N	Brought on Board (Retained/Discarded)	Live - Stomach and/or Eyes Protruding	Retained	Not Applicable	
64	UCAM	Caulolatilus microps	Blueline Tilefish	N	Brought on Board (Retained/Discarded)	Live - Healthy	Retained	Not Applicable	
64	UCAM	Caulolatilus microps	Blueline Tilefish	N	Brought on Board (Retained/Discarded)	Live - Healthy	Retained	Not Applicable	

64	UCAM	Caulolatilus microps	Blueline Tilefish	N	Brought on Board (Retained/Discarded)	Live - Healthy	Retained	Not Applicable	
64	UCAM	Caulolatilus microps	Blueline Tilefish	N	Brought on Board (Retained/Discarded)	Live - Healthy	Retained	Not Applicable	
64	UCAM	Caulolatilus microps	Blueline Tilefish	N	Brought on Board (Retained/Discarded)	Live - Healthy	Retained	Not Applicable	
64	UCAM	Caulolatilus microps	Blueline Tilefish	N	Brought on Board (Retained/Discarded)	Live - Healthy	Retained	Not Applicable	
64	UCAM	Epinephelus flavolimbatus	Yellowedge Grouper	N	Brought on Board (Retained/Discarded)	Live - Stomach and/or Eyes Protruding	Retained	Not Applicable	
64	UCAM	Epinephelus flavolimbatus	Yellowedge Grouper	N	Brought on Board (Retained/Discarded)	Live - Stomach and/or Eyes Protruding	Retained	Not Applicable	
64	UCAM	Epinephelus niveatus	Snowy Grouper	N	Brought on Board (Retained/Discarded)	Live - Healthy	Retained	Not Applicable	
65	NOCAM	Caulolatilus microps	Blueline Tilefish	N	Brought on Board (Retained/Discarded)	Live - Stomach and/or Eyes Protruding	Retained	Not Applicable	
65	NOCAM	Caulolatilus microps	Blueline Tilefish	N	Brought on Board (Retained/Discarded)	Live - Healthy	Retained	Not Applicable	
65	NOCAM	Caulolatilus microps	Blueline Tilefish	N	Brought on Board (Retained/Discarded)	Live - Healthy	Retained	Not Applicable	
65	NOCAM	Caulolatilus microps	Blueline Tilefish	N	Brought on Board (Retained/Discarded)	Live - Stomach and/or Eyes Protruding	Retained	Not Applicable	
65	NOCAM	Caulolatilus microps	Blueline Tilefish	N	Brought on Board (Retained/Discarded)	Live - Healthy	Retained	Not Applicable	
65	NOCAM	Caulolatilus microps	Blueline Tilefish	N	Brought on Board (Retained/Discarded)	Live - Stomach and/or Eyes Protruding	Retained	Not Applicable	
65	NOCAM	Caulolatilus microps	Blueline Tilefish	N	Brought on Board (Retained/Discarded)	Live - Healthy	Retained	Not Applicable	
65	NOCAM	Caulolatilus microps	Blueline Tilefish	N	Brought on Board (Retained/Discarded)	Live - Healthy	Retained	Not Applicable	
65	NOCAM	Caulolatilus microps	Blueline Tilefish	N	Brought on Board (Retained/Discarded)	Live - Stomach and/or Eyes Protruding	Retained	Not Applicable	
65	NOCAM	Caulolatilus microps	Blueline Tilefish	N	Brought on Board (Retained/Discarded)	Live - Stomach and/or Eyes Protruding	Retained	Not Applicable	
65	NOCAM	Epinephelus flavolimbatus	Yellowedge Grouper	N	Brought on Board (Retained/Discarded)	Live - Stomach and/or Eyes Protruding	Retained	Not Applicable	
65	NOCAM	Epinephelus flavolimbatus	Yellowedge Grouper	N	Brought on Board (Retained/Discarded)	Live - Stomach and/or Eyes Protruding	Retained	Not Applicable	
65	NOCAM	Epinephelus flavolimbatus	Yellowedge Grouper	N	Brought on Board (Retained/Discarded)	Live - Healthy	Retained	Not Applicable	
65	NOCAM	Epinephelus flavolimbatus	Yellowedge Grouper	N	Brought on Board (Retained/Discarded)	Live - Stomach and/or Eyes Protruding	Retained	Not Applicable	
65	NOCAM	Epinephelus flavolimbatus	Yellowedge Grouper	N	Brought on Board (Retained/Discarded)	Live - Healthy	Retained	Not Applicable	
65	NOCAM	Epinephelus flavolimbatus	Yellowedge Grouper	N	Brought on Board (Retained/Discarded)	Live - Stomach and/or Eyes Protruding	Retained	Not Applicable	
65	NOCAM	Epinephelus flavolimbatus	Yellowedge Grouper	N	Brought on Board (Retained/Discarded)	Live - Stomach and/or Eyes Protruding	Retained	Not Applicable	
65	NOCAM	Epinephelus niveatus	Snowy Grouper	N	Brought on Board (Retained/Discarded)	Live - Healthy	Retained	Not Applicable	
65	UCAM	Caulolatilus microps	Blueline Tilefish	N	Brought on Board (Retained/Discarded)	Live - Healthy	Retained	Not Applicable	
65	UCAM	Caulolatilus microps	Blueline Tilefish	N	Brought on Board (Retained/Discarded)	Live - Healthy	Retained	Not Applicable	
65	UCAM	Caulolatilus microps	Blueline Tilefish	N	Brought on Board (Retained/Discarded)	Live - Stomach and/or Eyes Protruding	Retained	Not Applicable	
65	UCAM	Caulolatilus microps	Blueline Tilefish	N	Brought on Board (Retained/Discarded)	Live - Healthy	Retained	Not Applicable	
65	UCAM	Caulolatilus microps	Blueline Tilefish	N	Brought on Board (Retained/Discarded)	Live - Healthy	Retained	Not Applicable	
65	UCAM	Caulolatilus microps	Blueline Tilefish	N	Brought on Board (Retained/Discarded)	Live - Stomach and/or Eyes Protruding	Retained	Not Applicable	
65	UCAM	Caulolatilus microps	Blueline Tilefish	N	Brought on Board (Retained/Discarded)	Live - Stomach and/or Eyes Protruding	Retained	Not Applicable	
65	UCAM	Caulolatilus microps	Blueline Tilefish	N	Brought on Board (Retained/Discarded)	Live - Healthy	Retained	Not Applicable	
65	UCAM	Caulolatilus microps	Blueline Tilefish	N	Brought on Board (Retained/Discarded)	Live - Healthy	Retained	Not Applicable	
65	UCAM	Caulolatilus microps	Blueline Tilefish	N	Brought on Board (Retained/Discarded)	Live - Healthy	Retained	Not Applicable	
65	UCAM	Epinephelus flavolimbatus	Yellowedge Grouper	N	Brought on Board (Retained/Discarded)	Live - Stomach and/or Eyes Protruding	Retained	Not Applicable	
65	UCAM	Epinephelus flavolimbatus	Yellowedge Grouper	N	Brought on Board (Retained/Discarded)	Live - Stomach and/or Eyes Protruding	Retained	Not Applicable	
65	UCAM	Epinephelus flavolimbatus	Yellowedge Grouper	N	Brought on Board (Retained/Discarded)	Live - Healthy	Retained	Not Applicable	
65	UCAM	Epinephelus flavolimbatus	Yellowedge Grouper	N	Brought on Board (Retained/Discarded)	Live - Healthy	Retained	Not Applicable	
65	UCAM	Epinephelus flavolimbatus	Yellowedge Grouper	N	Brought on Board (Retained/Discarded)	Live - Stomach and/or Eyes Protruding	Retained	Not Applicable	
65	UCAM	Epinephelus flavolimbatus	Yellowedge Grouper	N	Brought on Board (Retained/Discarded)	Live - Stomach and/or Eyes Protruding	Retained	Not Applicable	
65	UCAM	Epinephelus flavolimbatus	Yellowedge Grouper	N	Brought on Board (Retained/Discarded)	Live - Stomach and/or Eyes Protruding	Retained	Not Applicable	
65	UCAM	Epinephelus niveatus	Snowy Grouper	N	Brought on Board (Retained/Discarded)	Live - Healthy	Retained	Not Applicable	
66	NOCAM	Caulolatilus microps	Blueline Tilefish	N	Brought on Board (Retained/Discarded)	Live - Stomach and/or Eyes Protruding	Retained	Not Applicable	
66	NOCAM	Caulolatilus microps	Blueline Tilefish	N	Brought on Board (Retained/Discarded)	Live - Healthy	Retained	Not Applicable	
66	NOCAM	Caulolatilus microps	Blueline Tilefish	N	Brought on Board (Retained/Discarded)	Live - Healthy	Retained	Not Applicable	
66	NOCAM	Caulolatilus microps	Blueline Tilefish	N	Brought on Board (Retained/Discarded)	Live - Stomach and/or Eyes Protruding	Retained	Not Applicable	
66	NOCAM	Caulolatilus microps	Blueline Tilefish	N	Brought on Board (Retained/Discarded)	Live - Healthy	Retained	Not Applicable	
66	NOCAM	Caulolatilus microps	Blueline Tilefish	N	Brought on Board (Retained/Discarded)	Live - Healthy	Retained	Not Applicable	
66	NOCAM	Caulolatilus microps	Blueline Tilefish	N	Brought on Board (Retained/Discarded)	Live - Healthy	Retained	Not Applicable	
66	NOCAM	Caulolatilus microps	Blueline Tilefish	N	Brought on Board (Retained/Discarded)	Live - Healthy	Retained	Not Applicable	
66	NOCAM	Caulolatilus microps	Blueline Tilefish	N	Brought on Board (Retained/Discarded)	Live - Healthy	Retained	Not Applicable	
66	NOCAM	Caulolatilus microps	Blueline Tilefish	N	Brought on Board (Retained/Discarded)	Live - Healthy	Retained	Not Applicable	
66	NOCAM	Caulolatilus microps	Blueline Tilefish	N	Brought on Board (Retained/Discarded)	Live - Healthy	Retained	Not Applicable	
66	NOCAM	Caulolatilus microps	Blueline Tilefish	N	Brought on Board (Retained/Discarded)	Live - Healthy	Retained	Not Applicable	
66	NOCAM	Caulolatilus microps	Blueline Tilefish	N	Brought on Board (Retained/Discarded)	Live - Stomach and/or Eyes Protruding	Retained	Not Applicable	
66	NOCAM	Caulolatilus microps	Blueline Tilefish	N	Brought on Board (Retained/Discarded)	Live - Healthy	Retained	Not Applicable	
66	NOCAM	Caulolatilus microps	Blueline Tilefish	N	Brought on Board (Retained/Discarded)	Live - Healthy	Retained	Not Applicable	
66	NOCAM	Caulolatilus microps	Blueline Tilefish	N	Brought on Board (Retained/Discarded)	Live - Healthy	Retained	Not Applicable	
66	NOCAM	Caulolatilus microps	Blueline Tilefish	N	Brought on Board (Retained/Discarded)	Live - Stomach and/or Eyes Protruding	Retained	Not Applicable	
66	NOCAM	Caulolatilus microps	Blueline Tilefish	N	Brought on Board (Retained/Discarded)	Live - Stomach and/or Eyes Protruding	Retained	Not Applicable	
66	NOCAM	Caulolatilus microps	Blueline Tilefish	N	Brought on Board (Retained/Discarded)	Live - Healthy	Retained	Not Applicable	
66	NOCAM	Caulolatilus microps	Blueline Tilefish	N	Brought on Board (Retained/Discarded)	Live - Healthy	Retained	Not Applicable	
66	NOCAM	Caulolatilus microps	Blueline Tilefish	N	Brought on Board (Retained/Discarded)	Live - Stomach and/or Eyes Protruding	Retained	Not Applicable	
66	NOCAM	Caulolatilus microps	Blueline Tilefish	N	Brought on Board (Retained/Discarded)	Live - Healthy	Retained	Not Applicable	
66	NOCAM	Caulolatilus microps	Blueline Tilefish	N	Brought on Board (Retained/Discarded)	Live - Healthy	Retained	Not Applicable	
66	NOCAM	Caulolatilus microps	Blueline Tilefish	N	Brought on Board (Retained/Discarded)	Live - Healthy	Retained	Not Applicable	
66	NOCAM	Caulolatilus microps	Blueline Tilefish	N	Brought on Board (Retained/Discarded)	Live - Healthy	Retained	Not Applicable	
66	NOCAM	Caulolatilus microps	Blueline Tilefish	N	Brought on Board (Retained/Discarded)	Live - Stomach and/or Eyes Protruding	Retained	Not Applicable	
66	NOCAM	Caulolatilus microps	Blueline Tilefish	N	Brought on Board (Retained/Discarded)	Live - Stomach and/or Eyes Protruding	Retained	Not Applicable	
66	NOCAM	Caulolatilus microps	Blueline Tilefish	N	Brought on Board (Retained/Discarded)	Live - Healthy	Retained	Not Applicable	
66	NOCAM	Caulolatilus microps	Blueline Tilefish	N	Brought on Board (Retained/Discarded)	Live - Healthy	Retained	Not Applicable	
66	NOCAM	Caulolatilus microps	Blueline Tilefish	N	Brought on Board (Retained/Discarded)	Live - Stomach and/or Eyes Protruding	Retained	Not Applicable	
66	NOCAM	Caulolatilus microps	Blueline Tilefish	N	Brought on Board (Retained/Discarded)	Live - Healthy	Retained	Not Applicable	
66	NOCAM	Caulolatilus microps	Blueline Tilefish	N	Brought on Board (Retained/Discarded)	Live - Healthy	Retained	Not Applicable	
66	NOCAM	Caulolatilus microps	Blueline Tilefish	N	Brought on Board (Retained/Discarded)	Live - Stomach and/or Eyes Protruding	Retained	Not Applicable	
66	NOCAM	Caulolatilus microps	Blueline Tilefish	N	Brought on Board (Retained/Discarded)	Live - Healthy	Retained	Not Applicable	
66	NOCAM	Caulolatilus microps	Blueline Tilefish	N	Brought on Board (Retained/Discarded)	Live - Healthy	Retained	Not Applicable	
66	NOCAM	Caulolatilus microps	Blueline Tilefish	N	Brought on Board (Retained/Discarded)	Live - Stomach and/or Eyes Protruding	Retained	Not Applicable	
66	NOCAM	Caulolatilus microps	Blueline Tilefish	N	Brought on Board (Retained/Discarded)	Live - Healthy	Retained	Not Applicable	
66	NOCAM	Caulolatilus microps	Blueline Tilefish	N	Brought on Board (Retained/Discarded)	Live - Stomach and/or Eyes Protruding	Retained	Not Applicable	
66	NOCAM	Caulolatilus microps	Blueline Tilefish	N	Brought on Board (Retained/Discarded)	Live - Healthy	Retained	Not Applicable	
66	NOCAM	Caulolatilus microps	Blueline Tilefish	N	Brought on Board (Retained/Discarded)	Live - Healthy	Retained	Not Applicable	
66	NOCAM	Epinephelus flavolimbatus	Yellowedge Grouper	N	Brought on Board (Retained/Discarded)	Live - Stomach and/or Eyes Protruding	Retained	Not Applicable	
66	NOCAM	Epinephelus flavolimbatus	Yellowedge Grouper	N	Brought on Board (Retained/Discarded)	Live - Healthy	Retained	Not Applicable	
66	NOCAM	Epinephelus flavolimbatus	Yellowedge Grouper	N	Brought on Board (Retained/Discarded)	Live - Stomach and/or Eyes Protruding	Retained	Not Applicable	
66	NOCAM	Epinephelus flavolimbatus	Yellowedge Grouper	N	Brought on Board (Retained/Discarded)	Live - Stomach and/or Eyes Protruding	Retained	Not Applicable	
66	NOCAM	Epinephelus flavolimbatus	Yellowedge Grouper	N	Brought on Board (Retained/Discarded)	Live - Stomach and/or Eyes Protruding	Retained	Not Applicable	
66	NOCAM	Epinephelus flavolimbatus	Yellowedge Grouper	N	Brought on Board (Retained/Discarded)	Live - Stomach and/or Eyes Protruding	Retained	Not Applicable	
66	NOCAM	Epinephelus flavolimbatus	Yellowedge Grouper	N	Brought on Board (Retained/Discarded)	Live - Stomach and/or Eyes Protruding	Retained	Not Applicable	
66	NOCAM	Epinephelus flavolimbatus	Yellowedge Grouper	N	Brought on Board (Retained/Discarded)	Live - Stomach and/or Eyes Protruding	Retained	Not Applicable	
66	NOCAM	Epinephelus flavolimbatus	Yellowedge Grouper	N	Brought on Board (Retained/Discarded)	Live - Stomach and/or Eyes Protruding	Retained	Not Applicable	

66	NOCAM	Epinephelus flavolimbatus	Yellowedge Grouper	N	Brought on Board (Retained/Discarded)	Live - Stomach and/or Eyes Protruding	Retained	Not Applicable	
66	NOCAM	Lopholatilus chamaelonticeps	Tilefish, Golden	N	Brought on Board (Retained/Discarded)	Live - Healthy	Retained	Not Applicable	
66	NOCAM	Lopholatilus chamaelonticeps	Tilefish, Golden	N	Brought on Board (Retained/Discarded)	Live - Stomach and/or Eyes Protruding	Retained	Not Applicable	
66	NOCAM	Lopholatilus chamaelonticeps	Tilefish, Golden	N	Brought on Board (Retained/Discarded)	Live - Stomach and/or Eyes Protruding	Retained	Not Applicable	
66	NOCAM	Lopholatilus chamaelonticeps	Tilefish, Golden	N	Brought on Board (Retained/Discarded)	Live - Stomach and/or Eyes Protruding	Retained	Not Applicable	
66	NOCAM	Lopholatilus chamaelonticeps	Tilefish, Golden	N	Brought on Board (Retained/Discarded)	Live - Stomach and/or Eyes Protruding	Retained	Not Applicable	
66	NOCAM	Lopholatilus chamaelonticeps	Tilefish, Golden	N	Brought on Board (Retained/Discarded)	Live - Stomach and/or Eyes Protruding	Retained	Not Applicable	
66	UCAM	Caulolatilus microps	Blueline Tilefish	N	Brought on Board (Retained/Discarded)	Live - Healthy	Retained	Not Applicable	
66	UCAM	Caulolatilus microps	Blueline Tilefish	N	Brought on Board (Retained/Discarded)	Live - Stomach and/or Eyes Protruding	Retained	Not Applicable	
66	UCAM	Caulolatilus microps	Blueline Tilefish	N	Brought on Board (Retained/Discarded)	Live - Stomach and/or Eyes Protruding	Retained	Not Applicable	
66	UCAM	Caulolatilus microps	Blueline Tilefish	N	Brought on Board (Retained/Discarded)	Live - Healthy	Retained	Not Applicable	
66	UCAM	Caulolatilus microps	Blueline Tilefish	N	Brought on Board (Retained/Discarded)	Live - Healthy	Retained	Not Applicable	
66	UCAM	Caulolatilus microps	Blueline Tilefish	N	Brought on Board (Retained/Discarded)	Live - Healthy	Retained	Not Applicable	
66	UCAM	Caulolatilus microps	Blueline Tilefish	N	Brought on Board (Retained/Discarded)	Live - Healthy	Retained	Not Applicable	
66	UCAM	Caulolatilus microps	Blueline Tilefish	N	Brought on Board (Retained/Discarded)	Live - Stomach and/or Eyes Protruding	Retained	Not Applicable	
66	UCAM	Caulolatilus microps	Blueline Tilefish	N	Brought on Board (Retained/Discarded)	Live - Healthy	Retained	Not Applicable	
66	UCAM	Caulolatilus microps	Blueline Tilefish	N	Brought on Board (Retained/Discarded)	Live - Healthy	Retained	Not Applicable	
66	UCAM	Caulolatilus microps	Blueline Tilefish	N	Brought on Board (Retained/Discarded)	Live - Healthy	Retained	Not Applicable	
66	UCAM	Caulolatilus microps	Blueline Tilefish	N	Brought on Board (Retained/Discarded)	Live - Healthy	Retained	Not Applicable	
66	UCAM	Caulolatilus microps	Blueline Tilefish	N	Brought on Board (Retained/Discarded)	Live - Stomach and/or Eyes Protruding	Retained	Not Applicable	
66	UCAM	Caulolatilus microps	Blueline Tilefish	N	Brought on Board (Retained/Discarded)	Live - Healthy	Retained	Not Applicable	
66	UCAM	Caulolatilus microps	Blueline Tilefish	N	Brought on Board (Retained/Discarded)	Live - Healthy	Retained	Not Applicable	
66	UCAM	Caulolatilus microps	Blueline Tilefish	N	Brought on Board (Retained/Discarded)	Live - Healthy	Retained	Not Applicable	
66	UCAM	Caulolatilus microps	Blueline Tilefish	N	Brought on Board (Retained/Discarded)	Live - Healthy	Retained	Not Applicable	
66	UCAM	Caulolatilus microps	Blueline Tilefish	N	Brought on Board (Retained/Discarded)	Live - Stomach and/or Eyes Protruding	Retained	Not Applicable	
66	UCAM	Caulolatilus microps	Blueline Tilefish	N	Brought on Board (Retained/Discarded)	Live - Healthy	Retained	Not Applicable	
66	UCAM	Caulolatilus microps	Blueline Tilefish	N	Brought on Board (Retained/Discarded)	Live - Stomach and/or Eyes Protruding	Retained	Not Applicable	
66	UCAM	Caulolatilus microps	Blueline Tilefish	N	Brought on Board (Retained/Discarded)	Live - Healthy	Retained	Not Applicable	
66	UCAM	Caulolatilus microps	Blueline Tilefish	N	Brought on Board (Retained/Discarded)	Live - Healthy	Retained	Not Applicable	
66	UCAM	Caulolatilus microps	Blueline Tilefish	N	Brought on Board (Retained/Discarded)	Live - Stomach and/or Eyes Protruding	Retained	Not Applicable	
66	UCAM	Caulolatilus microps	Blueline Tilefish	N	Brought on Board (Retained/Discarded)	Live - Stomach and/or Eyes Protruding	Retained	Not Applicable	
66	UCAM	Caulolatilus microps	Blueline Tilefish	N	Brought on Board (Retained/Discarded)	Live - Healthy	Retained	Not Applicable	
66	UCAM	Caulolatilus microps	Blueline Tilefish	N	Brought on Board (Retained/Discarded)	Live - Healthy	Retained	Not Applicable	
66	UCAM	Caulolatilus microps	Blueline Tilefish	N	Brought on Board (Retained/Discarded)	Live - Stomach and/or Eyes Protruding	Retained	Not Applicable	
66	UCAM	Caulolatilus microps	Blueline Tilefish	N	Brought on Board (Retained/Discarded)	Live - Healthy	Retained	Not Applicable	
66	UCAM	Caulolatilus microps	Blueline Tilefish	N	Brought on Board (Retained/Discarded)	Live - Healthy	Retained	Not Applicable	
66	UCAM	Caulolatilus microps	Blueline Tilefish	N	Brought on Board (Retained/Discarded)	Live - Healthy	Retained	Not Applicable	
66	UCAM	Caulolatilus microps	Blueline Tilefish	N	Brought on Board (Retained/Discarded)	Live - Healthy	Retained	Not Applicable	
66	UCAM	Caulolatilus microps	Blueline Tilefish	N	Brought on Board (Retained/Discarded)	Live - Stomach and/or Eyes Protruding	Retained	Not Applicable	
66	UCAM	Caulolatilus microps	Blueline Tilefish	N	Brought on Board (Retained/Discarded)	Live - Healthy	Retained	Not Applicable	
66	UCAM	Caulolatilus microps	Blueline Tilefish	N	Brought on Board (Retained/Discarded)	Live - Stomach and/or Eyes Protruding	Retained	Not Applicable	
66	UCAM	Caulolatilus microps	Blueline Tilefish	N	Brought on Board (Retained/Discarded)	Live - Stomach and/or Eyes Protruding	Retained	Not Applicable	
66	UCAM	Caulolatilus microps	Blueline Tilefish	N	Brought on Board (Retained/Discarded)	Live - Stomach and/or Eyes Protruding	Retained	Not Applicable	
66	UCAM	Caulolatilus microps	Blueline Tilefish	N	Brought on Board (Retained/Discarded)	Live - Stomach and/or Eyes Protruding	Retained	Not Applicable	
66	UCAM	Caulolatilus microps	Blueline Tilefish	N	Brought on Board (Retained/Discarded)	Live - Healthy	Retained	Not Applicable	
66	UCAM	Caulolatilus microps	Blueline Tilefish	N	Brought on Board (Retained/Discarded)	Live - Healthy	Retained	Not Applicable	
66	UCAM	Caulolatilus microps	Blueline Tilefish	N	Brought on Board (Retained/Discarded)	Live - Healthy	Retained	Not Applicable	
66	UCAM	Epinephelus flavolimbatus	Yellowedge Grouper	N	Brought on Board (Retained/Discarded)	Live - Stomach and/or Eyes Protruding	Retained	Not Applicable	
66	UCAM	Epinephelus flavolimbatus	Yellowedge Grouper	N	Brought on Board (Retained/Discarded)	Live - Healthy	Retained	Not Applicable	
66	UCAM	Epinephelus flavolimbatus	Yellowedge Grouper	N	Brought on Board (Retained/Discarded)	Live - Stomach and/or Eyes Protruding	Retained	Not Applicable	
66	UCAM	Epinephelus flavolimbatus	Yellowedge Grouper	N	Brought on Board (Retained/Discarded)	Live - Stomach and/or Eyes Protruding	Retained	Not Applicable	
66	UCAM	Epinephelus flavolimbatus	Yellowedge Grouper	N	Brought on Board (Retained/Discarded)	Live - Stomach and/or Eyes Protruding	Retained	Not Applicable	
66	UCAM	Epinephelus flavolimbatus	Yellowedge Grouper	N	Brought on Board (Retained/Discarded)	Live - Stomach and/or Eyes Protruding	Retained	Not Applicable	
66	UCAM	Epinephelus flavolimbatus	Yellowedge Grouper	N	Brought on Board (Retained/Discarded)	Live - Stomach and/or Eyes Protruding	Retained	Not Applicable	
66	UCAM	Epinephelus flavolimbatus	Yellowedge Grouper	N	Brought on Board (Retained/Discarded)	Live - Stomach and/or Eyes Protruding	Retained	Not Applicable	
66	UCAM	Epinephelus flavolimbatus	Yellowedge Grouper	N	Brought on Board (Retained/Discarded)	Live - Stomach and/or Eyes Protruding	Retained	Not Applicable	
66	UCAM	Epinephelus flavolimbatus	Yellowedge Grouper	N	Brought on Board (Retained/Discarded)	Live - Stomach and/or Eyes Protruding	Retained	Not Applicable	
66	UCAM	Lopholatilus chamaelonticeps	Tilefish, Golden	N	Brought on Board (Retained/Discarded)	Live - Stomach and/or Eyes Protruding	Retained	Not Applicable	
66	UCAM	Lopholatilus chamaelonticeps	Tilefish, Golden	N	Brought on Board (Retained/Discarded)	Live - Stomach and/or Eyes Protruding	Retained	Not Applicable	
66	UCAM	Lopholatilus chamaelonticeps	Tilefish, Golden	N	Brought on Board (Retained/Discarded)	Live - Stomach and/or Eyes Protruding	Retained	Not Applicable	
66	UCAM	Lopholatilus chamaelonticeps	Tilefish, Golden	N	Brought on Board (Retained/Discarded)	Live - Healthy	Retained	Not Applicable	
66	UCAM	Lopholatilus chamaelonticeps	Tilefish, Golden	N	Brought on Board (Retained/Discarded)	Live - Stomach and/or Eyes Protruding	Retained	Not Applicable	
66	UCAM	Lopholatilus chamaelonticeps	Tilefish, Golden	N	Brought on Board (Retained/Discarded)	Live - Stomach and/or Eyes Protruding	Retained	Not Applicable	
67	NOCAM	Caulolatilus microps	Blueline Tilefish	N	Brought on Board (Retained/Discarded)	Live - Stomach and/or Eyes Protruding	Retained	Not Applicable	
67	NOCAM	Caulolatilus microps	Blueline Tilefish	N	Brought on Board (Retained/Discarded)	Live - Healthy	Retained	Not Applicable	
67	NOCAM	Caulolatilus microps	Blueline Tilefish	N	Brought on Board (Retained/Discarded)	Live - Stomach and/or Eyes Protruding	Retained	Not Applicable	
67	NOCAM	Caulolatilus microps	Blueline Tilefish	N	Brought on Board (Retained/Discarded)	Live - Healthy	Retained	Not Applicable	
67	NOCAM	Caulolatilus microps	Blueline Tilefish	N	Brought on Board (Retained/Discarded)	Live - Stomach and/or Eyes Protruding	Retained	Not Applicable	
67	NOCAM	Caulolatilus microps	Blueline Tilefish	N	Brought on Board (Retained/Discarded)	Live - Stomach and/or Eyes Protruding	Retained	Not Applicable	
67	NOCAM	Caulolatilus microps	Blueline Tilefish	N	Brought on Board (Retained/Discarded)	Live - Healthy	Retained	Not Applicable	
67	NOCAM	Caulolatilus microps	Blueline Tilefish	N	Brought on Board (Retained/Discarded)	Live - Stomach and/or Eyes Protruding	Retained	Not Applicable	
67	NOCAM	Caulolatilus microps	Blueline Tilefish	N	Brought on Board (Retained/Discarded)	Live - Stomach and/or Eyes Protruding	Retained	Not Applicable	
67	NOCAM	Caulolatilus microps	Blueline Tilefish	N	Brought on Board (Retained/Discarded)	Live - Stomach and/or Eyes Protruding	Retained	Not Applicable	
67	NOCAM	Caulolatilus microps	Blueline Tilefish	N	Brought on Board (Retained/Discarded)	Live - Stomach and/or Eyes Protruding	Retained	Not Applicable	
67	NOCAM	Caulolatilus microps	Blueline Tilefish	N	Brought on Board (Retained/Discarded)	Live - Healthy	Retained	Not Applicable	
67	NOCAM	Caulolatilus microps	Blueline Tilefish	N	Brought on Board (Retained/Discarded)	Live - Healthy	Retained	Not Applicable	
67	NOCAM	Caulolatilus microps	Blueline Tilefish	N	Brought on Board (Retained/Discarded)	Live - Healthy	Retained	Not Applicable	
67	NOCAM	Caulolatilus microps	Blueline Tilefish	N	Brought on Board (Retained/Discarded)	Live - Stomach and/or Eyes Protruding	Retained	Not Applicable	
67	NOCAM	Caulolatilus microps	Blueline Tilefish	N	Brought on Board (Retained/Discarded)	Live - Stomach and/or Eyes Protruding	Retained	Not Applicable	
67	NOCAM	Caulolatilus microps	Blueline Tilefish	N	Brought on Board (Retained/Discarded)	Live - Stomach and/or Eyes Protruding	Retained	Not Applicable	
67	NOCAM	Caulolatilus microps	Blueline Tilefish	N	Brought on Board (Retained/Discarded)	Live - Healthy	Retained	Not Applicable	
67	NOCAM	Caulolatilus microps	Blueline Tilefish	N	Brought on Board (Retained/Discarded)	Live - Healthy	Retained	Not Applicable	
67	NOCAM	Caulolatilus microps	Blueline Tilefish	N	Brought on Board (Retained/Discarded)	Live - Healthy	Retained	Not Applicable	
67	NOCAM	Caulolatilus microps	Blueline Tilefish	N	Brought on Board (Retained/Discarded)	Live - Stomach and/or Eyes Protruding	Retained	Not Applicable	
67	NOCAM	Caulolatilus microps	Blueline Tilefish	N	Brought on Board (Retained/Discarded)	Live - Stomach and/or Eyes Protruding	Retained	Not Applicable	
67	NOCAM	Caulolatilus microps	Blueline Tilefish	N	Brought on Board (Retained/Discarded)	Live - Healthy	Retained	Not Applicable	
67	NOCAM	Caulolatilus microps	Blueline Tilefish	N	Brought on Board (Retained/Discarded)	Live - Healthy	Retained	Not Applicable	
67	NOCAM	Caulolatilus microps	Blueline Tilefish	N	Brought on Board (Retained/Discarded)	Live - Stomach and/or Eyes Protruding	Retained	Not Applicable	
67	NOCAM	Caulolatilus microps	Blueline Tilefish	N	Brought on Board (Retained/Discarded)	Live - Healthy	Retained	Not Applicable	
67	NOCAM	Caulolatilus microps	Blueline Tilefish	N	Brought on Board (Retained/Discarded)	Live - Stomach and/or Eyes Protruding	Retained	Not Applicable	
67	NOCAM	Caulolatilus microps	Blueline Tilefish	N	Brought on Board (Retained/Discarded)	Live - Healthy	Retained	Not Applicable	
67	NOCAM	Epinephelus flavolimbatus	Yellowedge Grouper	N	Brought on Board (Retained/Discarded)	Live - Stomach and/or Eyes Protruding	Retained	Not Applicable	

67	NOCAM	Epinephelus flavolimbatus	Yellowedge Grouper	N	Brought on Board (Retained/Discarded)	Live - Stomach and/or Eyes Protruding	Retained	Not Applicable	
67	NOCAM	Epinephelus flavolimbatus	Yellowedge Grouper	N	Brought on Board (Retained/Discarded)	Live - Stomach and/or Eyes Protruding	Retained	Not Applicable	
67	NOCAM	Epinephelus flavolimbatus	Yellowedge Grouper	N	Brought on Board (Retained/Discarded)	Live - Stomach and/or Eyes Protruding	Retained	Not Applicable	
67	NOCAM	Epinephelus flavolimbatus	Yellowedge Grouper	N	Brought on Board (Retained/Discarded)	Live - Stomach and/or Eyes Protruding	Retained	Not Applicable	
67	NOCAM	Epinephelus flavolimbatus	Yellowedge Grouper	N	Brought on Board (Retained/Discarded)	Live - Stomach and/or Eyes Protruding	Retained	Not Applicable	
67	NOCAM	Epinephelus flavolimbatus	Yellowedge Grouper	N	Brought on Board (Retained/Discarded)	Live - Stomach and/or Eyes Protruding	Retained	Not Applicable	
67	NOCAM	Epinephelus flavolimbatus	Yellowedge Grouper	N	Brought on Board (Retained/Discarded)	Live - Stomach and/or Eyes Protruding	Retained	Not Applicable	
67	NOCAM	Epinephelus flavolimbatus	Yellowedge Grouper	N	Brought on Board (Retained/Discarded)	Live - Stomach and/or Eyes Protruding	Retained	Not Applicable	
67	NOCAM	Epinephelus flavolimbatus	Yellowedge Grouper	N	Brought on Board (Retained/Discarded)	Live - Healthy	Retained	Not Applicable	
67	NOCAM	Epinephelus flavolimbatus	Yellowedge Grouper	N	Brought on Board (Retained/Discarded)	Live - Stomach and/or Eyes Protruding	Retained	Not Applicable	
67	NOCAM	Epinephelus flavolimbatus	Yellowedge Grouper	N	Brought on Board (Retained/Discarded)	Live - Stomach and/or Eyes Protruding	Retained	Not Applicable	
67	NOCAM	Epinephelus flavolimbatus	Yellowedge Grouper	N	Brought on Board (Retained/Discarded)	Live - Stomach and/or Eyes Protruding	Retained	Not Applicable	
67	NOCAM	Epinephelus flavolimbatus	Yellowedge Grouper	N	Brought on Board (Retained/Discarded)	Live - Stomach and/or Eyes Protruding	Retained	Not Applicable	
67	NOCAM	Epinephelus flavolimbatus	Yellowedge Grouper	N	Brought on Board (Retained/Discarded)	Live - Stomach and/or Eyes Protruding	Retained	Not Applicable	
67	NOCAM	Gadiformes, unidentified	Hake, Unidentified	N	Brought on Board (Retained/Discarded)	Live - Stomach and/or Eyes Protruding	Retained as Bait	Not Applicable	
67	NOCAM	Lopholatilus chamaelonticeps	Tilefish, Golden	N	Brought on Board (Retained/Discarded)	Live - Healthy	Retained	Not Applicable	
67	UCAM	Caulolatilus microps	Blueline Tilefish	N	Brought on Board (Retained/Discarded)	Live - Healthy	Retained	Not Applicable	
67	UCAM	Caulolatilus microps	Blueline Tilefish	N	Brought on Board (Retained/Discarded)	Live - Stomach and/or Eyes Protruding	Retained	Not Applicable	
67	UCAM	Caulolatilus microps	Blueline Tilefish	N	Brought on Board (Retained/Discarded)	Live - Stomach and/or Eyes Protruding	Retained	Not Applicable	
67	UCAM	Caulolatilus microps	Blueline Tilefish	N	Brought on Board (Retained/Discarded)	Live - Stomach and/or Eyes Protruding	Retained	Not Applicable	
67	UCAM	Caulolatilus microps	Blueline Tilefish	N	Brought on Board (Retained/Discarded)	Live - Healthy	Retained	Not Applicable	
67	UCAM	Caulolatilus microps	Blueline Tilefish	N	Brought on Board (Retained/Discarded)	Live - Stomach and/or Eyes Protruding	Retained	Not Applicable	
67	UCAM	Caulolatilus microps	Blueline Tilefish	N	Brought on Board (Retained/Discarded)	Live - Stomach and/or Eyes Protruding	Retained	Not Applicable	
67	UCAM	Caulolatilus microps	Blueline Tilefish	N	Brought on Board (Retained/Discarded)	Live - Stomach and/or Eyes Protruding	Retained	Not Applicable	
67	UCAM	Caulolatilus microps	Blueline Tilefish	N	Brought on Board (Retained/Discarded)	Live - Healthy	Retained	Not Applicable	
67	UCAM	Caulolatilus microps	Blueline Tilefish	N	Brought on Board (Retained/Discarded)	Live - Healthy	Retained	Not Applicable	
67	UCAM	Caulolatilus microps	Blueline Tilefish	N	Brought on Board (Retained/Discarded)	Live - Stomach and/or Eyes Protruding	Retained	Not Applicable	
67	UCAM	Caulolatilus microps	Blueline Tilefish	N	Brought on Board (Retained/Discarded)	Live - Healthy	Retained	Not Applicable	
67	UCAM	Caulolatilus microps	Blueline Tilefish	N	Brought on Board (Retained/Discarded)	Live - Healthy	Retained	Not Applicable	
67	UCAM	Caulolatilus microps	Blueline Tilefish	N	Brought on Board (Retained/Discarded)	Live - Stomach and/or Eyes Protruding	Retained	Not Applicable	
67	UCAM	Caulolatilus microps	Blueline Tilefish	N	Brought on Board (Retained/Discarded)	Live - Stomach and/or Eyes Protruding	Retained	Not Applicable	
67	UCAM	Caulolatilus microps	Blueline Tilefish	N	Brought on Board (Retained/Discarded)	Live - Healthy	Retained	Not Applicable	
67	UCAM	Caulolatilus microps	Blueline Tilefish	N	Brought on Board (Retained/Discarded)	Live - Healthy	Retained	Not Applicable	
67	UCAM	Caulolatilus microps	Blueline Tilefish	N	Brought on Board (Retained/Discarded)	Live - Stomach and/or Eyes Protruding	Retained	Not Applicable	
67	UCAM	Caulolatilus microps	Blueline Tilefish	N	Brought on Board (Retained/Discarded)	Live - Healthy	Retained	Not Applicable	
67	UCAM	Caulolatilus microps	Blueline Tilefish	N	Brought on Board (Retained/Discarded)	Live - Stomach and/or Eyes Protruding	Retained	Not Applicable	
67	UCAM	Caulolatilus microps	Blueline Tilefish	N	Brought on Board (Retained/Discarded)	Live - Healthy	Retained	Not Applicable	
67	UCAM	Caulolatilus microps	Blueline Tilefish	N	Brought on Board (Retained/Discarded)	Live - Healthy	Retained	Not Applicable	
67	UCAM	Caulolatilus microps	Blueline Tilefish	N	Brought on Board (Retained/Discarded)	Live - Healthy	Retained	Not Applicable	
67	UCAM	Caulolatilus microps	Blueline Tilefish	N	Brought on Board (Retained/Discarded)	Live - Healthy	Retained	Not Applicable	
67	UCAM	Caulolatilus microps	Blueline Tilefish	N	Brought on Board (Retained/Discarded)	Live - Stomach and/or Eyes Protruding	Retained	Not Applicable	
67	UCAM	Caulolatilus microps	Blueline Tilefish	N	Brought on Board (Retained/Discarded)	Live - Stomach and/or Eyes Protruding	Retained	Not Applicable	
67	UCAM	Caulolatilus microps	Blueline Tilefish	N	Brought on Board (Retained/Discarded)	Live - Stomach and/or Eyes Protruding	Retained	Not Applicable	
67	UCAM	Caulolatilus microps	Blueline Tilefish	N	Brought on Board (Retained/Discarded)	Live - Stomach and/or Eyes Protruding	Retained	Not Applicable	
67	UCAM	Epinephelus flavolimbatus	Yellowedge Grouper	N	Brought on Board (Retained/Discarded)	Live - Stomach and/or Eyes Protruding	Retained	Not Applicable	
67	UCAM	Epinephelus flavolimbatus	Yellowedge Grouper	N	Brought on Board (Retained/Discarded)	Live - Stomach and/or Eyes Protruding	Retained	Not Applicable	
67	UCAM	Epinephelus flavolimbatus	Yellowedge Grouper	N	Brought on Board (Retained/Discarded)	Live - Stomach and/or Eyes Protruding	Retained	Not Applicable	
67	UCAM	Epinephelus flavolimbatus	Yellowedge Grouper	N	Brought on Board (Retained/Discarded)	Live - Stomach and/or Eyes Protruding	Retained	Not Applicable	
67	UCAM	Epinephelus flavolimbatus	Yellowedge Grouper	N	Brought on Board (Retained/Discarded)	Live - Stomach and/or Eyes Protruding	Retained	Not Applicable	
67	UCAM	Epinephelus flavolimbatus	Yellowedge Grouper	N	Brought on Board (Retained/Discarded)	Live - Stomach and/or Eyes Protruding	Retained	Not Applicable	
67	UCAM	Epinephelus flavolimbatus	Yellowedge Grouper	N	Brought on Board (Retained/Discarded)	Live - Stomach and/or Eyes Protruding	Retained	Not Applicable	
67	UCAM	Epinephelus flavolimbatus	Yellowedge Grouper	N	Brought on Board (Retained/Discarded)	Live - Stomach and/or Eyes Protruding	Retained	Not Applicable	
67	UCAM	Epinephelus flavolimbatus	Yellowedge Grouper	N	Brought on Board (Retained/Discarded)	Live - Stomach and/or Eyes Protruding	Retained	Not Applicable	
67	UCAM	Epinephelus flavolimbatus	Yellowedge Grouper	N	Brought on Board (Retained/Discarded)	Live - Healthy	Retained	Not Applicable	
67	UCAM	Epinephelus flavolimbatus	Yellowedge Grouper	N	Brought on Board (Retained/Discarded)	Live - Stomach and/or Eyes Protruding	Retained	Not Applicable	
67	UCAM	Epinephelus flavolimbatus	Yellowedge Grouper	N	Brought on Board (Retained/Discarded)	Live - Stomach and/or Eyes Protruding	Retained	Not Applicable	
67	UCAM	Epinephelus flavolimbatus	Yellowedge Grouper	N	Brought on Board (Retained/Discarded)	Live - Stomach and/or Eyes Protruding	Retained	Not Applicable	
67	UCAM	Epinephelus flavolimbatus	Yellowedge Grouper	N	Brought on Board (Retained/Discarded)	Live - Stomach and/or Eyes Protruding	Retained	Not Applicable	
67	UCAM	Epinephelus flavolimbatus	Yellowedge Grouper	N	Brought on Board (Retained/Discarded)	Live - Stomach and/or Eyes Protruding	Retained	Not Applicable	
67	UCAM	Gadiformes, unidentified	Hake, Unidentified	N	Brought on Board (Retained/Discarded)	Live - Stomach and/or Eyes Protruding	Retained as Bait	Not Applicable	
67	UCAM	Lopholatilus chamaelonticeps	Tilefish, Golden	N	Brought on Board (Retained/Discarded)	Live - Healthy	Retained	Not Applicable	
68	NOCAM	Anguilliformes, unidentified	Eel, Unidentified	N	Brought on Board (Retained/Discarded)	Live - Healthy	Retained as Bait	Not Applicable	
68	NOCAM	Carcharhinus signatus	Night Shark	Y	Brought on Board (Retained/Discarded)	Live - Healthy	Discarded - Live and Healthy (Not Vented)	Unknown Maturity and/or Sex	Medium (Between 1m and 2m)
68	NOCAM	Carcharhinus signatus	Night Shark	Y	Brought on Board (Retained/Discarded)	Live - Healthy	Discarded - Live and Healthy (Not Vented)	Unknown Maturity and/or Sex	Medium (Between 1m and 2m)
68	NOCAM	Carcharhinus signatus	Night Shark	Y	Brought on Board (Retained/Discarded)	Dead on Arrival - Undamaged	Discarded - Dead	Female	Medium (Between 1m and 2m)
68	NOCAM	Caulolatilus microps	Blueline Tilefish	N	Brought on Board (Retained/Discarded)	Live - Healthy	Retained	Not Applicable	
68	NOCAM	Caulolatilus microps	Blueline Tilefish	N	Brought on Board (Retained/Discarded)	Live - Healthy	Retained	Not Applicable	
68	NOCAM	Caulolatilus microps	Blueline Tilefish	N	Brought on Board (Retained/Discarded)	Live - Healthy	Retained	Not Applicable	
68	NOCAM	Caulolatilus microps	Blueline Tilefish	N	Brought on Board (Retained/Discarded)	Live - Healthy	Retained	Not Applicable	
68	NOCAM	Caulolatilus microps	Blueline Tilefish	N	Brought on Board (Retained/Discarded)	Live - Stomach and/or Eyes Protruding	Retained	Not Applicable	
68	NOCAM	Caulolatilus microps	Blueline Tilefish	N	Brought on Board (Retained/Discarded)	Live - Stomach and/or Eyes Protruding	Retained	Not Applicable	
68	NOCAM	Caulolatilus microps	Blueline Tilefish	N	Brought on Board (Retained/Discarded)	Live - Healthy	Retained	Not Applicable	
68	NOCAM	Caulolatilus microps	Blueline Tilefish	N	Brought on Board (Retained/Discarded)	Live - Healthy	Retained	Not Applicable	
68	NOCAM	Caulolatilus microps	Blueline Tilefish	N	Brought on Board (Retained/Discarded)	Live - Healthy	Retained	Not Applicable	
68	NOCAM	Caulolatilus microps	Blueline Tilefish	N	Brought on Board (Retained/Discarded)	Live - Healthy	Retained	Not Applicable	
68	NOCAM	Caulolatilus microps	Blueline Tilefish	N	Brought on Board (Retained/Discarded)	Live - Healthy	Retained	Not Applicable	
68	NOCAM	Epinephelus flavolimbatus	Yellowedge Grouper	N	Brought on Board (Retained/Discarded)	Live - Stomach and/or Eyes Protruding	Retained	Not Applicable	
68	NOCAM	Epinephelus flavolimbatus	Yellowedge Grouper	N	Brought on Board (Retained/Discarded)	Live - Stomach and/or Eyes Protruding	Retained	Not Applicable	
68	NOCAM	Epinephelus flavolimbatus	Yellowedge Grouper	N	Brought on Board (Retained/Discarded)	Live - Stomach and/or Eyes Protruding	Retained	Not Applicable	
68	NOCAM	Epinephelus flavolimbatus	Yellowedge Grouper	N	Brought on Board (Retained/Discarded)	Dead on Arrival - Damaged	Discarded - Dead	Not Applicable	
68	NOCAM	Gadiformes, unidentified	Hake, Unidentified	N	Brought on Board (Retained/Discarded)	Live - Stomach and/or Eyes Protruding	Retained as Bait	Not Applicable	
68	NOCAM	Gadiformes, unidentified	Hake, Unidentified	N	Brought on Board (Retained/Discarded)	Live - Stomach and/or Eyes Protruding	Retained as Bait	Not Applicable	
68	NOCAM	Gadiformes, unidentified	Hake, Unidentified	N	Brought on Board (Retained/Discarded)	Live - Stomach and/or Eyes Protruding	Retained as Bait	Not Applicable	
68	NOCAM	Gadiformes, unidentified	Hake, Unidentified	N	Brought on Board (Retained/Discarded)	Live - Stomach and/or Eyes Protruding	Retained as Bait	Not Applicable	
68	NOCAM	Lopholatilus chamaelonticeps	Tilefish, Golden	N	Brought on Board (Retained/Discarded)	Live - Healthy	Retained	Not Applicable	
68	NOCAM	Selachimorpha, unidentified	Shark, Unidentified	Y	Cut-off at Rail (No Entanglement)	Live - Healthy	Discarded - Live and Healthy (Not Vented)	Unknown Maturity and/or Sex	Medium (Between 1m and 2m)
68	NOCAM	Squalidae spp.	Dogfish, Spiny (Cuban)	Y	Brought on Board (Retained/Discarded)	Live - Healthy	Discarded - Live and Healthy (Not Vented)	Unknown Maturity and/or Sex	Small (Less than 1m)
68	NOCAM	Squalidae spp.	Dogfish, Spiny (Cuban)	Y	Brought on Board (Retained/Discarded)	Live - Healthy	Discarded - Live and Healthy (Not Vented)	Unknown Maturity and/or Sex	Small (Less than 1m)
68	NOCAM	Squalidae spp.	Dogfish, Spiny (Cuban)	Y	Brought on Board (Retained/Discarded)	Live - Healthy	Discarded - Live and Healthy (Not Vented)	Unknown Maturity and/or Sex	Small (Less than 1m)
68	NOCAM	Squalidae spp.	Dogfish, Spiny (Cuban)	Y	Brought on Board (Retained/Discarded)	Live - Healthy	Discarded - Live and Healthy (Not Vented)	Female	Small (Less than 1m)
68	NOCAM	Squalidae spp.	Dogfish, Spiny (Cuban)	Y	Brought on Board (Retained/Discarded)	Live - Healthy	Discarded - Live and Healthy (Not Vented)	Unknown Maturity and/or Sex	Small (Less than 1m)
68	NOCAM	Squalidae spp.	Dogfish, Spiny (Cuban)	Y	Brought on Board (Retained/Discarded)	Live - Healthy	Discarded - Live and Healthy (Not Vented)	Unknown Maturity and/or Sex	Small (Less than 1m)

68	NOCAM	Squalidae spp.	Dogfish, Spiny (Cuban)	Y	Brought on Board (Retained/Discarded)	Live - Healthy	Discarded - Live and Healthy (Not Vented)	Unknown Maturity and/or Sex	Small (Less than 1m)
68	UCAM	Anguilliformes, unidentified	Eel, Unidentified	N	Brought on Board (Retained/Discarded)	Live - Healthy	Retained as Bait	Not Applicable	
68	UCAM	Carcharhinus signatus	Night Shark	Y	Brought on Board (Retained/Discarded)	Live - Healthy	Discarded - Live and Healthy (Not Vented)	Unknown Maturity and/or Sex	Medium (Between 1m and 2m)
68	UCAM	Carcharhinus signatus	Night Shark	Y	Brought on Board (Retained/Discarded)	Live - Healthy	Discarded - Live and Healthy (Not Vented)	Unknown Maturity and/or Sex	Medium (Between 1m and 2m)
68	UCAM	Carcharhinus signatus	Night Shark	Y	Brought on Board (Retained/Discarded)	Dead on Arrival - Undamaged	Discarded - Dead	Female	Medium (Between 1m and 2m)
68	UCAM	Caulolatilus microps	Blueline Tilefish	N	Brought on Board (Retained/Discarded)	Live - Healthy	Retained	Not Applicable	
68	UCAM	Caulolatilus microps	Blueline Tilefish	N	Brought on Board (Retained/Discarded)	Live - Healthy	Retained	Not Applicable	
68	UCAM	Caulolatilus microps	Blueline Tilefish	N	Brought on Board (Retained/Discarded)	Live - Healthy	Retained	Not Applicable	
68	UCAM	Caulolatilus microps	Blueline Tilefish	N	Brought on Board (Retained/Discarded)	Live - Healthy	Retained	Not Applicable	
68	UCAM	Caulolatilus microps	Blueline Tilefish	N	Brought on Board (Retained/Discarded)	Live - Healthy	Retained	Not Applicable	
68	UCAM	Caulolatilus microps	Blueline Tilefish	N	Brought on Board (Retained/Discarded)	Live - Healthy	Retained	Not Applicable	
68	UCAM	Caulolatilus microps	Blueline Tilefish	N	Brought on Board (Retained/Discarded)	Live - Healthy	Retained	Not Applicable	
68	UCAM	Caulolatilus microps	Blueline Tilefish	N	Brought on Board (Retained/Discarded)	Live - Healthy	Retained	Not Applicable	
68	UCAM	Caulolatilus microps	Blueline Tilefish	N	Brought on Board (Retained/Discarded)	Live - Stomach and/or Eyes Protruding	Retained	Not Applicable	
68	UCAM	Caulolatilus microps	Blueline Tilefish	N	Brought on Board (Retained/Discarded)	Live - Stomach and/or Eyes Protruding	Retained	Not Applicable	
68	UCAM	Caulolatilus microps	Blueline Tilefish	N	Brought on Board (Retained/Discarded)	Live - Healthy	Retained	Not Applicable	
68	UCAM	Epinephelus flavolimbatus	Yellowedge Grouper	N	Brought on Board (Retained/Discarded)	Live - Stomach and/or Eyes Protruding	Retained	Not Applicable	
68	UCAM	Epinephelus flavolimbatus	Yellowedge Grouper	N	Brought on Board (Retained/Discarded)	Dead on Arrival - Damaged	Discarded - Dead	Not Applicable	
68	UCAM	Epinephelus flavolimbatus	Yellowedge Grouper	N	Brought on Board (Retained/Discarded)	Live - Stomach and/or Eyes Protruding	Retained	Not Applicable	
68	UCAM	Epinephelus flavolimbatus	Yellowedge Grouper	N	Brought on Board (Retained/Discarded)	Live - Stomach and/or Eyes Protruding	Retained	Not Applicable	
68	UCAM	Gadiformes, unidentified	Hake, Unidentified	N	Brought on Board (Retained/Discarded)	Live - Healthy	Retained as Bait	Not Applicable	
68	UCAM	Gadiformes, unidentified	Hake, Unidentified	N	Brought on Board (Retained/Discarded)	Live - Healthy	Retained as Bait	Not Applicable	
68	UCAM	Gadiformes, unidentified	Hake, Unidentified	N	Brought on Board (Retained/Discarded)	Live - Stomach and/or Eyes Protruding	Retained as Bait	Not Applicable	
68	UCAM	Gadiformes, unidentified	Hake, Unidentified	N	Brought on Board (Retained/Discarded)	Live - Stomach and/or Eyes Protruding	Retained as Bait	Not Applicable	
68	UCAM	Lopholatilus chamaelonticeps	Tilefish, Golden	N	Brought on Board (Retained/Discarded)	Live - Healthy	Retained	Not Applicable	
68	UCAM	Selachimorpha, unidentified	Shark, Unidentified	Y	Cut-off at Rail (No Entanglement)	Live - Healthy	Discarded - Live and Healthy (Not Vented)	Unknown Maturity and/or Sex	Medium (Between 1m and 2m)
68	UCAM	Squalidae spp.	Dogfish, Spiny (Cuban)	Y	Brought on Board (Retained/Discarded)	Live - Healthy	Discarded - Live and Healthy (Not Vented)	Female	Small (Less than 1m)
68	UCAM	Squalidae spp.	Dogfish, Spiny (Cuban)	Y	Brought on Board (Retained/Discarded)	Live - Healthy	Discarded - Live and Healthy (Not Vented)	Unknown Maturity and/or Sex	Small (Less than 1m)
68	UCAM	Squalidae spp.	Dogfish, Spiny (Cuban)	Y	Brought on Board (Retained/Discarded)	Live - Healthy	Discarded - Live and Healthy (Not Vented)	Unknown Maturity and/or Sex	Small (Less than 1m)
68	UCAM	Squalidae spp.	Dogfish, Spiny (Cuban)	Y	Brought on Board (Retained/Discarded)	Live - Healthy	Discarded - Live and Healthy (Not Vented)	Unknown Maturity and/or Sex	Small (Less than 1m)
68	UCAM	Squalidae spp.	Dogfish, Spiny (Cuban)	Y	Brought on Board (Retained/Discarded)	Live - Healthy	Discarded - Live and Healthy (Not Vented)	Unknown Maturity and/or Sex	Small (Less than 1m)
68	UCAM	Squalidae spp.	Dogfish, Spiny (Cuban)	Y	Brought on Board (Retained/Discarded)	Live - Healthy	Discarded - Live and Healthy (Not Vented)	Unknown Maturity and/or Sex	Small (Less than 1m)
68	UCAM	Squalidae spp.	Dogfish, Spiny (Cuban)	Y	Brought on Board (Retained/Discarded)	Live - Healthy	Discarded - Live and Healthy (Not Vented)	Unknown Maturity and/or Sex	Small (Less than 1m)

Table 1

