# Supplementary material for: Employing an innovative underwater camera to improve electronic monitoring in the commercial Gulf of Mexico reef fish fishery
Source: PLoS One. 2024 Mar 8;19(3):e0298588. doi: 10.1371/journal.pone.0298588 (PMC10923404; doi:10.1371/journal.pone.0298588)
Supplement: S2 Table — (PDF) [file pone.0298588.s002.pdf]

Shark BREP data for pub

Haul_Number	Review_Type	Species	Common_Name	Catch_Handling	Condition_On_Arrival	Catch_Fate	Sex_Of_Shark	Shark_Length_Estimate
1	NOCAM	Carcharhinus acronotus	Blacknose Shark	Brought on Board (Retained/Discarded)	Live - Healthy	Discarded - Live and Healthy (Not Vented)	Unknown Maturity and/or Sex	Small (Less than 1m)
1	NOCAM	Carcharhinus acronotus	Blacknose Shark	Brought on Board (Retained/Discarded)	Live - Healthy	Discarded - Live and Healthy (Not Vented)	Female	Medium (Between 1m and 2m)
1	NOCAM	Carcharhinus acronotus	Blacknose Shark	Brought on Board (Retained/Discarded)	Live - Healthy	Discarded - Live and Healthy (Not Vented)	Female	Small (Less than 1m)
1	NOCAM	Carcharhinus plumbeus	Sandbar Shark	Cut-off at Rail (No Entanglement)	Live - Healthy	Discarded - Live and Healthy (Not Vented)	Known Adult - Undetermined Sex	Large (Greater than 2m)
1	NOCAM	Rhizoprionodon terraenovae	Atlantic Sharpnose Shark	Brought on Board (Retained/Discarded)	Live - Healthy	Discarded - Live and Healthy (Not Vented)	Male (Claspers)	Small (Less than 1m)
1	NOCAM	Rhizoprionodon terraenovae	Atlantic Sharpnose Shark	Brought on Board (Retained/Discarded)	Live - Healthy	Discarded - Live and Healthy (Not Vented)	Female	Small (Less than 1m)
1	NOCAM	Rhizoprionodon terraenovae	Atlantic Sharpnose Shark	Brought on Board (Retained/Discarded)	Live - Healthy	Discarded - Live and Healthy (Not Vented)	Female	Small (Less than 1m)
1	NOCAM	Rhizoprionodon terraenovae	Atlantic Sharpnose Shark	Brought on Board (Retained/Discarded)	Live - Healthy	Discarded - Live and Healthy (Not Vented)	Female	Small (Less than 1m)
1	NOCAM	Rhizoprionodon terraenovae	Atlantic Sharpnose Shark	Brought on Board (Retained/Discarded)	Live - Healthy	Discarded - Live and Healthy (Not Vented)	Female	Small (Less than 1m)
1	NOCAM	Rhizoprionodon terraenovae	Atlantic Sharpnose Shark	Brought on Board (Retained/Discarded)	Live - Healthy	Discarded - Live and Healthy (Not Vented)	Female	Small (Less than 1m)
1	NOCAM	Rhizoprionodon terraenovae	Atlantic Sharpnose Shark	Brought on Board (Retained/Discarded)	Live - Healthy	Discarded - Live and Healthy (Not Vented)	Male (Claspers)	Small (Less than 1m)
1	UCAM	Carcharhinus acronotus	Blacknose Shark	Brought on Board (Retained/Discarded)	Live - Healthy	Discarded - Live and Healthy (Not Vented)	Unknown Maturity and/or Sex	Small (Less than 1m)
1	UCAM	Carcharhinus acronotus	Blacknose Shark	Brought on Board (Retained/Discarded)	Live - Healthy	Discarded - Live and Healthy (Not Vented)	Female	Medium (Between 1m and 2m)
1	UCAM	Carcharhinus acronotus	Blacknose Shark	Brought on Board (Retained/Discarded)	Live - Healthy	Discarded - Live and Healthy (Not Vented)	Female	Small (Less than 1m)
1	UCAM	Carcharhinus plumbeus	Sandbar Shark	Cut-off at Rail (No Entanglement)	Live - Healthy	Discarded - Live and Healthy (Not Vented)	Female	Large (Greater than 2m)
1	UCAM	Carcharhinus plumbeus	Sandbar Shark	Cut-off at Rail (No Entanglement)	Live - Healthy	Discarded - Live and Healthy (Not Vented)	Female	Large (Greater than 2m)
1	UCAM	Rhizoprionodon terraenovae	Atlantic Sharpnose Shark	Brought on Board (Retained/Discarded)	Live - Healthy	Discarded - Live and Healthy (Not Vented)	Male (Claspers)	Small (Less than 1m)
1	UCAM	Rhizoprionodon terraenovae	Atlantic Sharpnose Shark	Brought on Board (Retained/Discarded)	Live - Healthy	Discarded - Live and Healthy (Not Vented)	Female	Small (Less than 1m)
1	UCAM	Rhizoprionodon terraenovae	Atlantic Sharpnose Shark	Brought on Board (Retained/Discarded)	Live - Healthy	Discarded - Live and Healthy (Not Vented)	Female	Small (Less than 1m)
1	UCAM	Rhizoprionodon terraenovae	Atlantic Sharpnose Shark	Brought on Board (Retained/Discarded)	Live - Healthy	Discarded - Live and Healthy (Not Vented)	Female	Small (Less than 1m)
1	UCAM	Rhizoprionodon terraenovae	Atlantic Sharpnose Shark	Brought on Board (Retained/Discarded)	Live - Healthy	Discarded - Live and Healthy (Not Vented)	Female	Small (Less than 1m)
1	UCAM	Rhizoprionodon terraenovae	Atlantic Sharpnose Shark	Brought on Board (Retained/Discarded)	Live - Healthy	Discarded - Live and Healthy (Not Vented)	Female	Small (Less than 1m)
1	UCAM	Rhizoprionodon terraenovae	Atlantic Sharpnose Shark	Brought on Board (Retained/Discarded)	Live - Healthy	Discarded - Live and Healthy (Not Vented)	Male (Claspers)	Small (Less than 1m)
2	NOCAM	Carcharhinus acronotus	Blacknose Shark	Brought on Board (Retained/Discarded)	Live - Healthy	Discarded - Live and Healthy (Not Vented)	Female	Small (Less than 1m)
2	NOCAM	Carcharhinus acronotus	Blacknose Shark	Brought on Board (Retained/Discarded)	Live - Healthy	Discarded - Live and Healthy (Not Vented)	Unknown Maturity and/or Sex	Small (Less than 1m)
2	NOCAM	Carcharhinus falciformis	Silky Shark	Brought on Board (Retained/Discarded)	Live - Healthy	Discarded - Live and Healthy (Not Vented)	Female	Small (Less than 1m)
2	NOCAM	Carcharhinus plumbeus	Sandbar Shark	Cut-off at Rail (No Entanglement)	Live - Healthy	Discarded - Live and Healthy (Not Vented)	Known Adult - Undetermined Sex	Large (Greater than 2m)
2	UCAM	Carcharhinus acronotus	Blacknose Shark	Brought on Board (Retained/Discarded)	Live - Healthy	Discarded - Live and Healthy (Not Vented)	Female	Small (Less than 1m)
2	UCAM	Carcharhinus acronotus	Blacknose Shark	Brought on Board (Retained/Discarded)	Live - Healthy	Discarded - Live and Healthy (Not Vented)	Unknown Maturity and/or Sex	Small (Less than 1m)
2	UCAM	Carcharhinus falciformis	Silky Shark	Brought on Board (Retained/Discarded)	Live - Healthy	Discarded - Live and Healthy (Not Vented)	Female	Small (Less than 1m)
2	UCAM	Carcharhinus plumbeus	Sandbar Shark	Cut-off at Rail (No Entanglement)	Live - Healthy	Discarded - Live and Healthy (Not Vented)	Female	Large (Greater than 2m)
3	NOCAM	Carcharhinus acronotus	Blacknose Shark	Brought on Board (Retained/Discarded)	Live - Healthy	Discarded - Live and Healthy (Not Vented)	Female	Medium (Between 1m and 2m)
3	NOCAM	Carcharhinus falciformis	Silky Shark	Brought on Board (Retained/Discarded)	Live - Healthy	Discarded - Live and Healthy (Not Vented)	Female	Small (Less than 1m)
3	NOCAM	Rhizoprionodon terraenovae	Atlantic Sharpnose Shark	Brought on Board (Retained/Discarded)	Live - Healthy	Discarded - Live and Healthy (Not Vented)	Male (Claspers)	Small (Less than 1m)
3	UCAM	Carcharhinus acronotus	Blacknose Shark	Brought on Board (Retained/Discarded)	Live - Healthy	Discarded - Live and Healthy (Not Vented)	Female	Medium (Between 1m and 2m)
3	UCAM	Carcharhinus falciformis	Silky Shark	Brought on Board (Retained/Discarded)	Live - Healthy	Discarded - Live and Healthy (Not Vented)	Female	Small (Less than 1m)
3	UCAM	Rhizoprionodon terraenovae	Atlantic Sharpnose Shark	Brought on Board (Retained/Discarded)	Live - Healthy	Discarded - Live and Healthy (Not Vented)	Male (Claspers)	Small (Less than 1m)
4	NOCAM	Carcharhinus acronotus	Blacknose Shark	Brought on Board (Retained/Discarded)	Live - Healthy	Discarded - Live and Healthy (Not Vented)	Unknown Maturity and/or Sex	Small (Less than 1m)
4	NOCAM	Ginglymostoma cirratum	Nurse Shark	Cut-off at Rail (No Entanglement)	Live - Healthy	Discarded - Live and Healthy (Not Vented)	Male (Claspers)	Large (Greater than 2m)
4	NOCAM	Ginglymostoma cirratum	Nurse Shark	Cut-off at Rail (No Entanglement)	Live - Healthy	Discarded - Live and Healthy (Not Vented)	Known Adult - Undetermined Sex	Large (Greater than 2m)
4	UCAM	Carcharhinus acronotus	Blacknose Shark	Brought on Board (Retained/Discarded)	Live - Healthy	Discarded - Live and Healthy (Not Vented)	Female	Small (Less than 1m)
4	UCAM	Ginglymostoma cirratum	Nurse Shark	Cut-off at Rail (No Entanglement)	Live - Healthy	Discarded - Live and Healthy (Not Vented)	Male (Claspers)	Large (Greater than 2m)
4	UCAM	Ginglymostoma cirratum	Nurse Shark	Cut-off at Rail (No Entanglement)	Live - Healthy	Discarded - Live and Healthy (Not Vented)	Male (Claspers)	Large (Greater than 2m)
5	NOCAM	Carcharhinus signatus	Night Shark	Brought on Board (Retained/Discarded)	Live - Healthy	Discarded - Live and Healthy (Not Vented)	Known Juvenile	Medium (Between 1m and 2m)
5	NOCAM	Mustelus spp.	Dogfish, Smooth (Florida)	Brought on Board (Retained/Discarded)	Live - Healthy	Discarded - Live and Healthy (Not Vented)	Female	Medium (Between 1m and 2m)
5	NOCAM	Mustelus spp.	Dogfish, Smooth (Florida)	Brought on Board (Retained/Discarded)	Live - Healthy	Discarded - Live and Healthy (Not Vented)	Male (Claspers)	Small (Less than 1m)
5	NOCAM	Mustelus spp.	Dogfish, Smooth (Florida)	Brought on Board (Retained/Discarded)	Live - Healthy	Discarded - Live and Healthy (Not Vented)	Female	Small (Less than 1m)
5	UCAM	Carcharhinus signatus	Night Shark	Brought on Board (Retained/Discarded)	Live - Healthy	Discarded - Live and Healthy (Not Vented)	Known Juvenile	Medium (Between 1m and 2m)
5	UCAM	Mustelus spp.	Dogfish, Smooth (Florida)	Brought on Board (Retained/Discarded)	Live - Healthy	Discarded - Live and Healthy (Not Vented)	Female	Medium (Between 1m and 2m)
5	UCAM	Mustelus spp.	Dogfish, Smooth (Florida)	Brought on Board (Retained/Discarded)	Live - Healthy	Discarded - Live and Healthy (Not Vented)	Male (Claspers)	Small (Less than 1m)
5	UCAM	Mustelus spp.	Dogfish, Smooth (Florida)	Brought on Board (Retained/Discarded)	Live - Healthy	Discarded - Live and Healthy (Not Vented)	Female	Small (Less than 1m)
7	NOCAM	Mustelus spp.	Dogfish, Smooth (Florida)	Brought on Board (Retained/Discarded)	Live - Healthy	Discarded - Live and Healthy (Not Vented)	Unknown Maturity and/or Sex	Medium (Between 1m and 2m)
7	UCAM	Mustelus spp.	Dogfish, Smooth (Florida)	Brought on Board (Retained/Discarded)	Live - Healthy	Discarded - Live and Healthy (Not Vented)	Female	Medium (Between 1m and 2m)
8	NOCAM	Mustelus spp.	Dogfish, Smooth (Florida)	Brought on Board (Retained/Discarded)	Live - Healthy	Discarded - Live and Healthy (Not Vented)	Female	Small (Less than 1m)
8	UCAM	Mustelus spp.	Dogfish, Smooth (Florida)	Brought on Board (Retained/Discarded)	Live - Healthy	Discarded - Live and Healthy (Not Vented)	Female	Small (Less than 1m)
9	NOCAM	Carcharhinus signatus	Night Shark	Brought on Board (Retained/Discarded)	Live - Healthy	Discarded - Live and Healthy (Not Vented)	Female	Small (Less than 1m)
9	NOCAM	Mustelus spp.	Dogfish, Smooth (Florida)	Brought on Board (Retained/Discarded)	Live - Healthy	Discarded - Live and Healthy (Not Vented)	Male (Claspers)	Small (Less than 1m)
9	NOCAM	Mustelus spp.	Dogfish, Smooth (Florida)	Brought on Board (Retained/Discarded)	Live - Healthy	Discarded - Live and Healthy (Not Vented)	Male (Claspers)	Medium (Between 1m and 2m)
9	NOCAM	Mustelus spp.	Dogfish, Smooth (Florida)	Brought on Board (Retained/Discarded)	Live - Healthy	Discarded - Live and Healthy (Not Vented)	Unknown Maturity and/or Sex	Small (Less than 1m)
9	NOCAM	Mustelus spp.	Dogfish, Smooth (Florida)	Brought on Board (Retained/Discarded)	Live - Healthy	Discarded - Live and Healthy (Not Vented)	Female	Small (Less than 1m)
9	NOCAM	Mustelus spp.	Dogfish, Smooth (Florida)	Brought on Board (Retained/Discarded)	Live - Healthy	Discarded - Live and Healthy (Not Vented)	Female	Medium (Between 1m and 2m)
9	UCAM	Carcharhinus signatus	Night Shark	Brought on Board (Retained/Discarded)	Live - Healthy	Discarded - Live and Healthy (Not Vented)	Female	Small (Less than 1m)
9	UCAM	Mustelus spp.	Dogfish, Smooth (Florida)	Brought on Board (Retained/Discarded)	Live - Healthy	Discarded - Live and Healthy (Not Vented)	Male (Claspers)	Medium (Between 1m and 2m)
9	UCAM	Mustelus spp.	Dogfish, Smooth (Florida)	Brought on Board (Retained/Discarded)	Live - Healthy	Discarded - Live and Healthy (Not Vented)	Female	Small (Less than 1m)
9	UCAM	Mustelus spp.	Dogfish, Smooth (Florida)	Brought on Board (Retained/Discarded)	Live - Healthy	Discarded - Live and Healthy (Not Vented)	Unknown Maturity and/or Sex	Small (Less than 1m)
9	UCAM	Mustelus spp.	Dogfish, Smooth (Florida)	Brought on Board (Retained/Discarded)	Live - Healthy	Discarded - Live and Healthy (Not Vented)	Male (Claspers)	Small (Less than 1m)
9	UCAM	Mustelus spp.	Dogfish, Smooth (Florida)	Brought on Board (Retained/Discarded)	Live - Healthy	Discarded - Live and Healthy (Not Vented)	Female	Medium (Between 1m and 2m)
11	NOCAM	Carcharhinus signatus	Night Shark	Brought on Board (Retained/Discarded)	Live - Healthy	Discarded - Live and Healthy (Not Vented)	Female	Small (Less than 1m)
11	NOCAM	Carcharhinus signatus	Night Shark	Brought on Board (Retained/Discarded)	Live - Healthy	Discarded - Live and Healthy (Not Vented)	Female	Medium (Between 1m and 2m)
11	NOCAM	Carcharhinus signatus	Night Shark	Brought on Board (Retained/Discarded)	Live - Healthy	Discarded - Live and Healthy (Not Vented)	Known Juvenile	Small (Less than 1m)
11	UCAM	Carcharhinus signatus	Night Shark	Brought on Board (Retained/Discarded)	Live - Healthy	Discarded - Live and Healthy (Not Vented)	Female	Medium (Between 1m and 2m)
11	UCAM	Carcharhinus signatus	Night Shark	Brought on Board (Retained/Discarded)	Live - Healthy	Discarded - Live and Healthy (Not Vented)	Known Juvenile	Small (Less than 1m)
11	UCAM	Carcharhinus signatus	Night Shark	Brought on Board (Retained/Discarded)	Live - Healthy	Discarded - Live and Healthy (Not Vented)	Female	Small (Less than 1m)
13	NOCAM	Carcharhinus signatus	Night Shark	Cut-off at Rail (No Entanglement)	Live - Healthy	Discarded - Live and Healthy (Not Vented)	Unknown Maturity and/or Sex	Medium (Between 1m and 2m)
13	UCAM	Carcharhinus signatus	Night Shark	Cut-off at Rail (No Entanglement)	Live - Healthy	Discarded - Live and Healthy (Not Vented)	Male (Claspers)	Medium (Between 1m and 2m)
14	NOCAM	Carcharhinus signatus	Night Shark	Brought on Board (Retained/Discarded)	Live - Healthy	Discarded - Live and Healthy (Not Vented)	Male (Claspers)	Medium (Between 1m and 2m)
14	NOCAM	Mustelus spp.	Dogfish, Smooth (Florida)	Brought on Board (Retained/Discarded)	Live - Healthy	Discarded - Live and Healthy (Not Vented)	Female	Medium (Between 1m and 2m)
14	UCAM	Carcharhinus signatus	Night Shark	Brought on Board (Retained/Discarded)	Live - Healthy	Discarded - Live and Healthy (Not Vented)	Male (Claspers)	Medium (Between 1m and 2m)
14	UCAM	Mustelus spp.	Dogfish, Smooth (Florida)	Brought on Board (Retained/Discarded)	Live - Healthy	Discarded - Live and Healthy (Not Vented)	Female	Medium (Between 1m and 2m)
17	NOCAM	Mustelus spp.	Dogfish, Smooth (Florida)	Brought on Board (Retained/Discarded)	Live - Healthy	Discarded - Live and Healthy (Not Vented)	Female	Medium (Between 1m and 2m)
17	UCAM	Mustelus spp.	Dogfish, Smooth (Florida)	Brought on Board (Retained/Discarded)	Live - Healthy	Discarded - Live and Healthy (Not Vented)	Female	Medium (Between 1m and 2m)
19	UCAM	Carcharhinus plumbeus	Sandbar Shark	Cut-off at Rail (No Entanglement)	Live - Healthy	Discarded - Live and Healthy (Not Vented)	Male (Claspers)	Large (Greater than 2m)

20	NOCAM	Carcharhinus plumbeus	Sandbar Shark	Cut-off at Rail (No Entanglement)	Live - Healthy	Discarded - Live and Healthy (Not Vented)	Known Adult - Undetermined Sex	Large (Greater than 2m)
20	NOCAM	Galeocerdo cuvier	Tiger Shark	Cut-off at Rail (No Entanglement)	Live - Healthy	Discarded - Live and Healthy (Not Vented)	Unknown Maturity and/or Sex	Medium (Between 1m and 2m)
20	NOCAM	Rhizoprionodon terraenovae	Atlantic Sharpnose Shark	Brought on Board (Retained/Discarded)	Live - Healthy	Discarded - Live and Healthy (Not Vented)	Female	Small (Less than 1m)
20	NOCAM	Sphyrna lewini	Scalloped Hammerhead	Cut-off at Rail (No Entanglement)	Live - Healthy	Discarded - Live and Healthy (Not Vented)	Unknown Maturity and/or Sex	Medium (Between 1m and 2m)
20	UCAM	Carcharhinus plumbeus	Sandbar Shark	Cut-off at Rail (No Entanglement)	Live - Healthy	Discarded - Live and Healthy (Not Vented)	Male (Claspers)	Large (Greater than 2m)
20	UCAM	Galeocerdo cuvier	Tiger Shark	Cut-off at Rail (No Entanglement)	Live - Healthy	Discarded - Live and Healthy (Not Vented)	Unknown Maturity and/or Sex	Medium (Between 1m and 2m)
20	UCAM	Rhizoprionodon terraenovae	Atlantic Sharpnose Shark	Brought on Board (Retained/Discarded)	Live - Healthy	Discarded - Live and Healthy (Not Vented)	Female	Small (Less than 1m)
20	UCAM	Sphyrna lewini	Scalloped Hammerhead	Cut-off at Rail (No Entanglement)	Live - Healthy	Discarded - Live and Healthy (Not Vented)	Male (Claspers)	Medium (Between 1m and 2m)
21	NOCAM	Carcharhinus plumbeus	Sandbar Shark	Cut-off at Rail (No Entanglement)	Live - Healthy	Discarded - Live and Healthy (Not Vented)	Known Adult - Undetermined Sex	Large (Greater than 2m)
21	NOCAM	Carcharhinus plumbeus	Sandbar Shark	Cut-off at Rail (No Entanglement)	Live - Healthy	Discarded - Live and Healthy (Not Vented)	Male (Claspers)	Medium (Between 1m and 2m)
21	NOCAM	Carcharhinus plumbeus	Sandbar Shark	Cut-off at Rail (No Entanglement)	Live - Healthy	Discarded - Live and Healthy (Not Vented)	Known Adult - Undetermined Sex	Large (Greater than 2m)
21	NOCAM	Rhizoprionodon terraenovae	Atlantic Sharpnose Shark	Brought on Board (Retained/Discarded)	Live - Healthy	Discarded - Live and Healthy (Not Vented)	Male (Claspers)	Small (Less than 1m)
21	UCAM	Carcharhinus plumbeus	Sandbar Shark	Cut-off at Rail (No Entanglement)	Live - Healthy	Discarded - Live and Healthy (Not Vented)	Male (Claspers)	Large (Greater than 2m)
21	UCAM	Carcharhinus plumbeus	Sandbar Shark	Cut-off at Rail (No Entanglement)	Live - Healthy	Discarded - Live and Healthy (Not Vented)	Male (Claspers)	Large (Greater than 2m)
21	UCAM	Carcharhinus plumbeus	Sandbar Shark	Cut-off at Rail (No Entanglement)	Live - Healthy	Discarded - Live and Healthy (Not Vented)	Known Adult - Undetermined Sex	Large (Greater than 2m)
21	UCAM	Rhizoprionodon terraenovae	Atlantic Sharpnose Shark	Brought on Board (Retained/Discarded)	Live - Healthy	Discarded - Live and Healthy (Not Vented)	Male (Claspers)	Small (Less than 1m)
22	NOCAM	Rhizoprionodon terraenovae	Atlantic Sharpnose Shark	Brought on Board (Retained/Discarded)	Live - Healthy	Discarded - Live and Healthy (Not Vented)	Female	Small (Less than 1m)
22	NOCAM	Rhizoprionodon terraenovae	Atlantic Sharpnose Shark	Brought on Board (Retained/Discarded)	Live - Healthy	Discarded - Live and Healthy (Not Vented)	Female	Small (Less than 1m)
22	NOCAM	Rhizoprionodon terraenovae	Atlantic Sharpnose Shark	Brought on Board (Retained/Discarded)	Live - Healthy	Discarded - Live and Healthy (Not Vented)	Female	Small (Less than 1m)
22	UCAM	Rhizoprionodon terraenovae	Atlantic Sharpnose Shark	Brought on Board (Retained/Discarded)	Live - Healthy	Discarded - Live and Healthy (Not Vented)	Female	Small (Less than 1m)
22	UCAM	Rhizoprionodon terraenovae	Atlantic Sharpnose Shark	Brought on Board (Retained/Discarded)	Live - Healthy	Discarded - Live and Healthy (Not Vented)	Female	Small (Less than 1m)
22	UCAM	Rhizoprionodon terraenovae	Atlantic Sharpnose Shark	Brought on Board (Retained/Discarded)	Live - Healthy	Discarded - Live and Healthy (Not Vented)	Female	Small (Less than 1m)
23	NOCAM	Carcharhinus plumbeus	Sandbar Shark	Cut-off at Rail (No Entanglement)	Live - Healthy	Discarded - Live and Healthy (Not Vented)	Known Adult - Undetermined Sex	Large (Greater than 2m)
23	NOCAM	Carcharhinus plumbeus	Sandbar Shark	Cut-off at Rail (No Entanglement)	Live - Healthy	Discarded - Live and Healthy (Not Vented)	Known Adult - Undetermined Sex	Large (Greater than 2m)
23	NOCAM	Carcharhinus plumbeus	Sandbar Shark	Cut-off at Rail (No Entanglement)	Live - Healthy	Discarded - Live and Healthy (Not Vented)	Known Adult - Undetermined Sex	Large (Greater than 2m)
23	NOCAM	Galeocerdo cuvier	Tiger Shark	Cut-off at Rail (No Entanglement)	Live - Healthy	Discarded - Live and Healthy (Not Vented)	Unknown Maturity and/or Sex	Large (Greater than 2m)
23	UCAM	Carcharhinus plumbeus	Sandbar Shark	Cut-off at Rail (No Entanglement)	Live - Healthy	Discarded - Live and Healthy (Not Vented)	Male (Claspers)	Large (Greater than 2m)
23	UCAM	Carcharhinus plumbeus	Sandbar Shark	Cut-off at Rail (No Entanglement)	Live - Healthy	Discarded - Live and Healthy (Not Vented)	Female	Large (Greater than 2m)
23	UCAM	Carcharhinus plumbeus	Sandbar Shark	Cut-off at Rail (No Entanglement)	Live - Healthy	Discarded - Live and Healthy (Not Vented)	Male (Claspers)	Large (Greater than 2m)
23	UCAM	Carcharhinus plumbeus	Sandbar Shark	Cut-off at Rail (No Entanglement)	Live - Healthy	Discarded - Live and Healthy (Not Vented)	Known Adult - Undetermined Sex	Large (Greater than 2m)
23	UCAM	Carcharhinus plumbeus	Sandbar Shark	Cut-off at Rail (No Entanglement)	Live - Healthy	Discarded - Live and Healthy (Not Vented)	Male (Claspers)	Large (Greater than 2m)
23	UCAM	Galeocerdo cuvier	Tiger Shark	Cut-off at Rail (No Entanglement)	Live - Healthy	Discarded - Live and Healthy (Not Vented)	Female	Large (Greater than 2m)
24	NOCAM	Carcharhinus plumbeus	Sandbar Shark	Cut-off at Rail (No Entanglement)	Live - Healthy	Discarded - Live and Healthy (Not Vented)	Male (Claspers)	Large (Greater than 2m)
24	UCAM	Carcharhinus plumbeus	Sandbar Shark	Cut-off at Rail (No Entanglement)	Live - Healthy	Discarded - Live and Healthy (Not Vented)	Male (Claspers)	Large (Greater than 2m)
25	NOCAM	Carcharhinus acronotus	Blacknose Shark	Cut-off at Rail (No Entanglement)	Live - Healthy	Discarded - Live and Healthy (Not Vented)	Unknown Maturity and/or Sex	Medium (Between 1m and 2m)
25	NOCAM	Carcharhinus plumbeus	Sandbar Shark	Cut-off at Rail (No Entanglement)	Live - Healthy	Discarded - Live and Healthy (Not Vented)	Known Adult - Undetermined Sex	Large (Greater than 2m)
25	NOCAM	Galeocerdo cuvier	Tiger Shark	Cut-off at Rail (No Entanglement)	Live - Healthy	Discarded - Live and Healthy (Not Vented)	Known Juvenile	Medium (Between 1m and 2m)
25	UCAM	Carcharhinus leucas	Bull Shark	Cut-off at Rail (No Entanglement)	Live - Healthy	Discarded - Live and Healthy (Not Vented)	Female	Large (Greater than 2m)
25	UCAM	Galeocerdo cuvier	Tiger Shark	Cut-off at Rail (No Entanglement)	Live - Healthy	Discarded - Live and Healthy (Not Vented)	Known Juvenile	Medium (Between 1m and 2m)
26	NOCAM	Carcharhinus plumbeus	Sandbar Shark	Cut-off at Rail (No Entanglement)	Live - Healthy	Discarded - Live and Healthy (Not Vented)	Male (Claspers)	Large (Greater than 2m)
26	NOCAM	Carcharhinus plumbeus	Sandbar Shark	Cut-off at Rail (No Entanglement)	Live - Healthy	Discarded - Live and Healthy (Not Vented)	Known Adult - Undetermined Sex	Large (Greater than 2m)
26	NOCAM	Carcharhinus plumbeus	Sandbar Shark	Cut-off at Rail (No Entanglement)	Live - Healthy	Discarded - Live and Healthy (Not Vented)	Male (Claspers)	Large (Greater than 2m)
26	UCAM	Carcharhinus plumbeus	Sandbar Shark	Cut-off at Rail (No Entanglement)	Live - Healthy	Discarded - Live and Healthy (Not Vented)	Male (Claspers)	Large (Greater than 2m)
26	UCAM	Carcharhinus plumbeus	Sandbar Shark	Cut-off at Rail (No Entanglement)	Live - Healthy	Discarded - Live and Healthy (Not Vented)	Male (Claspers)	Large (Greater than 2m)
26	UCAM	Carcharhinus plumbeus	Sandbar Shark	Cut-off at Rail (No Entanglement)	Live - Healthy	Discarded - Live and Healthy (Not Vented)	Female	Large (Greater than 2m)
26	UCAM	Carcharhinus plumbeus	Sandbar Shark	Cut-off at Rail (No Entanglement)	Live - Healthy	Discarded - Live and Healthy (Not Vented)	Male (Claspers)	Large (Greater than 2m)
27	NOCAM	Carcharhinus acronotus	Blacknose Shark	Cut-off at Rail (No Entanglement)	Live - Healthy	Discarded - Live and Healthy (Not Vented)	Unknown Maturity and/or Sex	Medium (Between 1m and 2m)
27	NOCAM	Carcharhinus acronotus	Blacknose Shark	Cut-off at Rail (No Entanglement)	Live - Healthy	Discarded - Live and Healthy (Not Vented)	Unknown Maturity and/or Sex	Medium (Between 1m and 2m)
27	NOCAM	Carcharhinus acronotus	Blacknose Shark	Cut-off at Rail (No Entanglement)	Live - Healthy	Discarded - Live and Healthy (Not Vented)	Female	Medium (Between 1m and 2m)
27	NOCAM	Carcharhinus plumbeus	Sandbar Shark	Cut-off at Rail (No Entanglement)	Live - Healthy	Discarded - Live and Healthy (Not Vented)	Known Adult - Undetermined Sex	Large (Greater than 2m)
27	NOCAM	Carcharhinus plumbeus	Sandbar Shark	Cut-off at Rail (No Entanglement)	Live - Healthy	Discarded - Live and Healthy (Not Vented)	Known Adult - Undetermined Sex	Large (Greater than 2m)
27	NOCAM	Carcharhinus, unidentified	Carcharhinid, Unidentified	Cut-off at Rail (No Entanglement)	Live - Healthy	Discarded - Live and Healthy (Not Vented)	Unknown Maturity and/or Sex	Large (Greater than 2m)
27	NOCAM	Ginglymostoma cirratum	Nurse Shark	Cut-off at Rail (No Entanglement)	Live - Healthy	Discarded - Live and Healthy (Not Vented)	Known Adult - Undetermined Sex	Large (Greater than 2m)
27	NOCAM	Sphyrna makarran	Great Hammerhead	Cut-off at Rail (No Entanglement)	Dead on Arrival - Undar	Discarded - Dead	Unknown Maturity and/or Sex	Large (Greater than 2m)
27	UCAM	Carcharhinus acronotus	Blacknose Shark	Brought on Board (Retained/Discarded)	Live - Healthy	Discarded - Live and Healthy (Not Vented)	Female	Medium (Between 1m and 2m)
27	UCAM	Carcharhinus acronotus	Blacknose Shark	Cut-off at Rail (No Entanglement)	Live - Healthy	Discarded - Live and Healthy (Not Vented)	Male (Claspers)	Medium (Between 1m and 2m)
27	UCAM	Carcharhinus acronotus	Blacknose Shark	Cut-off at Rail (No Entanglement)	Live - Healthy	Discarded - Live and Healthy (Not Vented)	Female	Medium (Between 1m and 2m)
27	UCAM	Carcharhinus plumbeus	Sandbar Shark	Cut-off at Rail (No Entanglement)	Live - Healthy	Discarded - Live and Healthy (Not Vented)	Female	Large (Greater than 2m)
27	UCAM	Carcharhinus plumbeus	Sandbar Shark	Cut-off at Rail (No Entanglement)	Live - Healthy	Discarded - Live and Healthy (Not Vented)	Male (Claspers)	Large (Greater than 2m)
27	UCAM	Carcharhinus plumbeus	Sandbar Shark	Cut-off at Rail (No Entanglement)	Live - Healthy	Discarded - Live and Healthy (Not Vented)	Female	Medium (Between 1m and 2m)
27	UCAM	Ginglymostoma cirratum	Nurse Shark	Cut-off at Rail (No Entanglement)	Live - Healthy	Discarded - Live and Healthy (Not Vented)	Known Adult - Undetermined Sex	Large (Greater than 2m)
27	UCAM	Sphyrna makarran	Great Hammerhead	Cut-off at Rail (No Entanglement)	Dead on Arrival - Undar	Discarded - Dead	Unknown Maturity and/or Sex	Large (Greater than 2m)
28	NOCAM	Carcharhinus plumbeus	Sandbar Shark	Cut-off at Rail (No Entanglement)	Live - Healthy	Discarded - Live and Healthy (Not Vented)	Known Adult - Undetermined Sex	Large (Greater than 2m)
28	NOCAM	Carcharhinus plumbeus	Sandbar Shark	Cut-off at Rail (No Entanglement)	Live - Healthy	Discarded - Live and Healthy (Not Vented)	Female	Large (Greater than 2m)
28	NOCAM	Rhizoprionodon terraenovae	Atlantic Sharpnose Shark	Brought on Board (Retained/Discarded)	Live - Healthy	Discarded - Live and Healthy (Not Vented)	Female	Small (Less than 1m)
28	UCAM	Carcharhinus plumbeus	Sandbar Shark	Cut-off at Rail (No Entanglement)	Live - Healthy	Discarded - Live and Healthy (Not Vented)	Female	Large (Greater than 2m)
28	UCAM	Carcharhinus plumbeus	Sandbar Shark	Cut-off at Rail (No Entanglement)	Live - Healthy	Discarded - Live and Healthy (Not Vented)	Female	Large (Greater than 2m)
28	UCAM	Rhizoprionodon terraenovae	Atlantic Sharpnose Shark	Brought on Board (Retained/Discarded)	Live - Healthy	Discarded - Live and Healthy (Not Vented)	Female	Small (Less than 1m)
29	NOCAM	Carcharhinus acronotus	Blacknose Shark	Brought on Board (Retained/Discarded)	Live - Healthy	Discarded - Live and Healthy (Not Vented)	Female	Small (Less than 1m)
29	NOCAM	Carcharhinus acronotus	Blacknose Shark	Brought on Board (Retained/Discarded)	Live - Healthy	Discarded - Live and Healthy (Not Vented)	Male (Claspers)	Small (Less than 1m)
29	NOCAM	Rhizoprionodon terraenovae	Atlantic Sharpnose Shark	Brought on Board (Retained/Discarded)	Live - Healthy	Discarded - Live and Healthy (Not Vented)	Male (Claspers)	Small (Less than 1m)
29	NOCAM	Rhizoprionodon terraenovae	Atlantic Sharpnose Shark	Brought on Board (Retained/Discarded)	Unknown Condition	Discarded - Unknown	Unknown Maturity and/or Sex	Small (Less than 1m)
29	NOCAM	Rhizoprionodon terraenovae	Atlantic Sharpnose Shark	Brought on Board (Retained/Discarded)	Live - Healthy	Discarded - Live and Healthy (Not Vented)	Female	Small (Less than 1m)
29	NOCAM	Rhizoprionodon terraenovae	Atlantic Sharpnose Shark	Brought on Board (Retained/Discarded)	Live - Healthy	Discarded - Live and Healthy (Not Vented)	Female	Small (Less than 1m)
29	NOCAM	Selachimorpha, unidentified	Shark, Unidentified	Cut-off at Rail (No Entanglement)	Live - Healthy	Discarded - Live and Healthy (Not Vented)	Unknown Maturity and/or Sex	Medium (Between 1m and 2m)
29	NOCAM	Sphyrna lewini	Scalloped Hammerhead	Cut-off at Rail (No Entanglement)	Dead on Arrival - Undar	Discarded - Dead	Unknown Maturity and/or Sex	Medium (Between 1m and 2m)
29	UCAM	Carcharhinus acronotus	Blacknose Shark	Brought on Board (Retained/Discarded)	Live - Healthy	Discarded - Live and Healthy (Not Vented)	Male (Claspers)	Small (Less than 1m)
29	UCAM	Carcharhinus acronotus	Blacknose Shark	Brought on Board (Retained/Discarded)	Live - Healthy	Discarded - Live and Healthy (Not Vented)	Female	Small (Less than 1m)
29	UCAM	Carcharhinus limbatus	Blacktip Shark	Cut-off at Rail (No Entanglement)	Live - Healthy	Discarded - Live and Healthy (Not Vented)	Male (Claspers)	Medium (Between 1m and 2m)
29	UCAM	Carcharhinus plumbeus	Sandbar Shark	Cut-off at Rail (No Entanglement)	Live - Healthy	Discarded - Live and Healthy (Not Vented)	Male (Claspers)	Large (Greater than 2m)
29	UCAM	Rhizoprionodon terraenovae	Atlantic Sharpnose Shark	Brought on Board (Retained/Discarded)	Live - Healthy	Discarded - Live and Healthy (Not Vented)	Male (Claspers)	Small (Less than 1m)
29	UCAM	Rhizoprionodon terraenovae	Atlantic Sharpnose Shark	Brought on Board (Retained/Discarded)	Dead on Arrival - Undar	Discarded - Dead	Unknown Maturity and/or Sex	Small (Less than 1m)
29	UCAM	Rhizoprionodon terraenovae	Atlantic Sharpnose Shark	Brought on Board (Retained/Discarded)	Live - Healthy	Discarded - Live and Healthy (Not Vented)	Female	Small (Less than 1m)
29	UCAM	Rhizoprionodon terraenovae	Atlantic Sharpnose Shark	Brought on Board (Retained/Discarded)	Live - Healthy	Discarded - Live and Healthy (Not Vented)	Female	Small (Less than 1m)

29	UCAM	Sphyrna lewini	Scalloped Hammerhead	Cut-off at Rail (No Entanglement)	Live - Healthy	Discarded - Live and Healthy (Not Vented)	Unknown Maturity and/or Sex	Medium (Between 1m and 2m)
30	NOCAM	Carcharhinus acronotus	Blacknose Shark	Brought on Board (Retained/Discarded)	Live - Healthy	Discarded - Live and Healthy (Not Vented)	Female	Small (Less than 1m)
30	NOCAM	Carcharhinus acronotus	Blacknose Shark	Brought on Board (Retained/Discarded)	Live - Healthy	Discarded - Live and Healthy (Not Vented)	Female	Small (Less than 1m)
30	NOCAM	Carcharhinus acronotus	Blacknose Shark	Brought on Board (Retained/Discarded)	Live - Healthy	Discarded - Live and Healthy (Not Vented)	Female	Small (Less than 1m)
30	NOCAM	Carcharhinus acronotus	Blacknose Shark	Brought on Board (Retained/Discarded)	Live - Healthy	Discarded - Live and Healthy (Not Vented)	Female	Small (Less than 1m)
30	NOCAM	Carcharhinus acronotus	Blacknose Shark	Brought on Board (Retained/Discarded)	Live - Healthy	Discarded - Live and Healthy (Not Vented)	Unknown Maturity and/or Sex	Small (Less than 1m)
30	NOCAM	Carcharhinus plumbeus	Sandbar Shark	Cut-off at Rail (No Entanglement)	Live - Healthy	Discarded - Live and Healthy (Not Vented)	Male (Claspers)	Large (Greater than 2m)
30	NOCAM	Rhizoprionodon terraenovae	Atlantic Sharpnose Shark	Brought on Board (Retained/Discarded)	Live - Healthy	Discarded - Live and Healthy (Not Vented)	Female	Small (Less than 1m)
30	NOCAM	Rhizoprionodon terraenovae	Atlantic Sharpnose Shark	Brought on Board (Retained/Discarded)	Live - Healthy	Discarded - Live and Healthy (Not Vented)	Male (Claspers)	Small (Less than 1m)
30	UCAM	Carcharhinus acronotus	Blacknose Shark	Brought on Board (Retained/Discarded)	Live - Healthy	Discarded - Live and Healthy (Not Vented)	Female	Small (Less than 1m)
30	UCAM	Carcharhinus acronotus	Blacknose Shark	Brought on Board (Retained/Discarded)	Live - Healthy	Discarded - Live and Healthy (Not Vented)	Unknown Maturity and/or Sex	Small (Less than 1m)
30	UCAM	Carcharhinus acronotus	Blacknose Shark	Brought on Board (Retained/Discarded)	Live - Healthy	Discarded - Live and Healthy (Not Vented)	Female	Small (Less than 1m)
30	UCAM	Carcharhinus acronotus	Blacknose Shark	Brought on Board (Retained/Discarded)	Live - Healthy	Discarded - Live and Healthy (Not Vented)	Female	Small (Less than 1m)
30	UCAM	Carcharhinus acronotus	Blacknose Shark	Brought on Board (Retained/Discarded)	Live - Healthy	Discarded - Live and Healthy (Not Vented)	Female	Small (Less than 1m)
30	UCAM	Carcharhinus plumbeus	Sandbar Shark	Cut-off at Rail (No Entanglement)	Live - Healthy	Discarded - Live and Healthy (Not Vented)	Male (Claspers)	Large (Greater than 2m)
30	UCAM	Rhizoprionodon terraenovae	Atlantic Sharpnose Shark	Brought on Board (Retained/Discarded)	Live - Healthy	Discarded - Live and Healthy (Not Vented)	Female	Small (Less than 1m)
30	UCAM	Rhizoprionodon terraenovae	Atlantic Sharpnose Shark	Brought on Board (Retained/Discarded)	Live - Healthy	Discarded - Live and Healthy (Not Vented)	Male (Claspers)	Small (Less than 1m)
31	NOCAM	Carcharhinus acronotus	Blacknose Shark	Brought on Board (Retained/Discarded)	Live - Healthy	Discarded - Live and Healthy (Not Vented)	Female	Medium (Between 1m and 2m)
31	NOCAM	Carcharhinus acronotus	Blacknose Shark	Brought on Board (Retained/Discarded)	Live - Healthy	Discarded - Live and Healthy (Not Vented)	Known Adult - Undetermined Sex	Medium (Between 1m and 2m)
31	NOCAM	Carcharhinus acronotus	Blacknose Shark	Cut-off at Rail (No Entanglement)	Live - Healthy	Discarded - Live and Healthy (Not Vented)	Unknown Maturity and/or Sex	Medium (Between 1m and 2m)
31	NOCAM	Carcharhinus acronotus	Blacknose Shark	Brought on Board (Retained/Discarded)	Live - Healthy	Discarded - Live and Healthy (Not Vented)	Unknown Maturity and/or Sex	Small (Less than 1m)
31	NOCAM	Carcharhinus plumbeus	Sandbar Shark	Cut-off at Rail (No Entanglement)	Live - Healthy	Discarded - Live and Healthy (Not Vented)	Known Adult - Undetermined Sex	Large (Greater than 2m)
31	NOCAM	Carcharhinus plumbeus	Sandbar Shark	Cut-off at Rail (No Entanglement)	Live - Healthy	Discarded - Live and Healthy (Not Vented)	Known Adult - Undetermined Sex	Large (Greater than 2m)
31	NOCAM	Rhizoprionodon terraenovae	Atlantic Sharpnose Shark	Brought on Board (Retained/Discarded)	Live - Healthy	Discarded - Live and Healthy (Not Vented)	Unknown Maturity and/or Sex	Small (Less than 1m)
31	NOCAM	Rhizoprionodon terraenovae	Atlantic Sharpnose Shark	Brought on Board (Retained/Discarded)	Live - Healthy	Discarded - Live and Healthy (Not Vented)	Female	Small (Less than 1m)
31	NOCAM	Rhizoprionodon terraenovae	Atlantic Sharpnose Shark	Brought on Board (Retained/Discarded)	Live - Healthy	Discarded - Live and Healthy (Not Vented)	Male (Claspers)	Small (Less than 1m)
31	NOCAM	Rhizoprionodon terraenovae	Atlantic Sharpnose Shark	Brought on Board (Retained/Discarded)	Live - Healthy	Discarded - Live and Healthy (Not Vented)	Female	Small (Less than 1m)
31	NOCAM	Rhizoprionodon terraenovae	Atlantic Sharpnose Shark	Brought on Board (Retained/Discarded)	Live - Healthy	Discarded - Live and Healthy (Not Vented)	Female	Small (Less than 1m)
31	NOCAM	Rhizoprionodon terraenovae	Atlantic Sharpnose Shark	Brought on Board (Retained/Discarded)	Live - Healthy	Discarded - Live and Healthy (Not Vented)	Unknown Maturity and/or Sex	Small (Less than 1m)
31	NOCAM	Rhizoprionodon terraenovae	Atlantic Sharpnose Shark	Brought on Board (Retained/Discarded)	Live - Healthy	Discarded - Live and Healthy (Not Vented)	Female	Small (Less than 1m)
31	NOCAM	Rhizoprionodon terraenovae	Atlantic Sharpnose Shark	Brought on Board (Retained/Discarded)	Live - Healthy	Discarded - Live and Healthy (Not Vented)	Male (Claspers)	Small (Less than 1m)
31	NOCAM	Rhizoprionodon terraenovae	Atlantic Sharpnose Shark	Brought on Board (Retained/Discarded)	Live - Healthy	Discarded - Live and Healthy (Not Vented)	Female	Small (Less than 1m)
31	NOCAM	Rhizoprionodon terraenovae	Atlantic Sharpnose Shark	Cut-off at Rail (No Entanglement)	Live - Healthy	Discarded - Live and Healthy (Not Vented)	Female	Small (Less than 1m)
31	NOCAM	Rhizoprionodon terraenovae	Atlantic Sharpnose Shark	Brought on Board (Retained/Discarded)	Live - Healthy	Discarded - Live and Healthy (Not Vented)	Female	Small (Less than 1m)
31	NOCAM	Rhizoprionodon terraenovae	Atlantic Sharpnose Shark	Brought on Board (Retained/Discarded)	Live - Healthy	Discarded - Live and Healthy (Not Vented)	Male (Claspers)	Small (Less than 1m)
31	NOCAM	Rhizoprionodon terraenovae	Atlantic Sharpnose Shark	Brought on Board (Retained/Discarded)	Live - Healthy	Discarded - Live and Healthy (Not Vented)	Female	Small (Less than 1m)
31	UCAM	Carcharhinus acronotus	Blacknose Shark	Cut-off at Rail (No Entanglement)	Live - Healthy	Discarded - Live and Healthy (Not Vented)	Unknown Maturity and/or Sex	Medium (Between 1m and 2m)
31	UCAM	Carcharhinus acronotus	Blacknose Shark	Brought on Board (Retained/Discarded)	Live - Healthy	Discarded - Live and Healthy (Not Vented)	Female	Medium (Between 1m and 2m)
31	UCAM	Carcharhinus acronotus	Blacknose Shark	Brought on Board (Retained/Discarded)	Live - Healthy	Discarded - Live and Healthy (Not Vented)	Unknown Maturity and/or Sex	Small (Less than 1m)
31	UCAM	Carcharhinus acronotus	Blacknose Shark	Brought on Board (Retained/Discarded)	Live - Healthy	Discarded - Live and Healthy (Not Vented)	Known Adult - Undetermined Sex	Medium (Between 1m and 2m)
31	UCAM	Carcharhinus plumbeus	Sandbar Shark	Cut-off at Rail (No Entanglement)	Live - Healthy	Discarded - Live and Healthy (Not Vented)	Male (Claspers)	Large (Greater than 2m)
31	UCAM	Rhizoprionodon terraenovae	Atlantic Sharpnose Shark	Brought on Board (Retained/Discarded)	Live - Healthy	Discarded - Live and Healthy (Not Vented)	Male (Claspers)	Small (Less than 1m)
31	UCAM	Rhizoprionodon terraenovae	Atlantic Sharpnose Shark	Brought on Board (Retained/Discarded)	Live - Healthy	Discarded - Live and Healthy (Not Vented)	Female	Small (Less than 1m)
31	UCAM	Rhizoprionodon terraenovae	Atlantic Sharpnose Shark	Brought on Board (Retained/Discarded)	Live - Healthy	Discarded - Live and Healthy (Not Vented)	Female	Small (Less than 1m)
31	UCAM	Rhizoprionodon terraenovae	Atlantic Sharpnose Shark	Brought on Board (Retained/Discarded)	Live - Healthy	Discarded - Live and Healthy (Not Vented)	Unknown Maturity and/or Sex	Small (Less than 1m)
31	UCAM	Rhizoprionodon terraenovae	Atlantic Sharpnose Shark	Brought on Board (Retained/Discarded)	Live - Healthy	Discarded - Live and Healthy (Not Vented)	Unknown Maturity and/or Sex	Small (Less than 1m)
31	UCAM	Rhizoprionodon terraenovae	Atlantic Sharpnose Shark	Brought on Board (Retained/Discarded)	Live - Healthy	Discarded - Live and Healthy (Not Vented)	Female	Small (Less than 1m)
31	UCAM	Rhizoprionodon terraenovae	Atlantic Sharpnose Shark	Brought on Board (Retained/Discarded)	Live - Healthy	Discarded - Live and Healthy (Not Vented)	Female	Small (Less than 1m)
31	UCAM	Rhizoprionodon terraenovae	Atlantic Sharpnose Shark	Brought on Board (Retained/Discarded)	Live - Healthy	Discarded - Live and Healthy (Not Vented)	Female	Small (Less than 1m)
31	UCAM	Rhizoprionodon terraenovae	Atlantic Sharpnose Shark	Brought on Board (Retained/Discarded)	Live - Healthy	Discarded - Live and Healthy (Not Vented)	Male (Claspers)	Small (Less than 1m)
31	UCAM	Rhizoprionodon terraenovae	Atlantic Sharpnose Shark	Brought on Board (Retained/Discarded)	Live - Healthy	Discarded - Live and Healthy (Not Vented)	Female	Small (Less than 1m)
31	UCAM	Rhizoprionodon terraenovae	Atlantic Sharpnose Shark	Brought on Board (Retained/Discarded)	Live - Healthy	Discarded - Live and Healthy (Not Vented)	Female	Small (Less than 1m)
31	UCAM	Rhizoprionodon terraenovae	Atlantic Sharpnose Shark	Brought on Board (Retained/Discarded)	Live - Healthy	Discarded - Live and Healthy (Not Vented)	Unknown Maturity and/or Sex	Small (Less than 1m)
31	UCAM	Rhizoprionodon terraenovae	Atlantic Sharpnose Shark	Brought on Board (Retained/Discarded)	Live - Healthy	Discarded - Live and Healthy (Not Vented)	Male (Claspers)	Small (Less than 1m)
32	NOCAM	Rhizoprionodon terraenovae	Atlantic Sharpnose Shark	Brought on Board (Retained/Discarded)	Live - Healthy	Discarded - Live and Healthy (Not Vented)	Female	Small (Less than 1m)
32	NOCAM	Rhizoprionodon terraenovae	Atlantic Sharpnose Shark	Brought on Board (Retained/Discarded)	Live - Healthy	Discarded - Live and Healthy (Not Vented)	Unknown Maturity and/or Sex	Small (Less than 1m)
32	UCAM	Rhizoprionodon terraenovae	Atlantic Sharpnose Shark	Brought on Board (Retained/Discarded)	Live - Healthy	Discarded - Live and Healthy (Not Vented)	Female	Small (Less than 1m)
33	NOCAM	Mustelus spp.	Dogfish, Smooth (Florida)	Brought on Board (Retained/Discarded)	Live - Healthy	Discarded - Live and Healthy (Not Vented)	Male (Claspers)	Small (Less than 1m)
33	NOCAM	Mustelus spp.	Dogfish, Smooth (Florida)	Brought on Board (Retained/Discarded)	Live - Healthy	Discarded - Live and Healthy (Not Vented)	Male (Claspers)	Small (Less than 1m)
33	NOCAM	Mustelus spp.	Dogfish, Smooth (Florida)	Brought on Board (Retained/Discarded)	Live - Healthy	Discarded - Live and Healthy (Not Vented)	Male (Claspers)	Medium (Between 1m and 2m)
33	NOCAM	Mustelus spp.	Dogfish, Smooth (Florida)	Brought on Board (Retained/Discarded)	Live - Healthy	Discarded - Live and Healthy (Not Vented)	Male (Claspers)	Medium (Between 1m and 2m)
33	NOCAM	Mustelus spp.	Dogfish, Smooth (Florida)	Brought on Board (Retained/Discarded)	Live - Healthy	Discarded - Live and Healthy (Not Vented)	Female	Small (Less than 1m)
33	NOCAM	Selachimorpha, unidentified	Shark, Unidentified	Brought on Board (Retained/Discarded)	Live - Healthy	Discarded - Live and Healthy (Not Vented)	Unknown Maturity and/or Sex	Medium (Between 1m and 2m)
33	UCAM	Mustelus spp.	Dogfish, Smooth (Florida)	Brought on Board (Retained/Discarded)	Live - Healthy	Discarded - Live and Healthy (Not Vented)	Female	Small (Less than 1m)
33	UCAM	Mustelus spp.	Dogfish, Smooth (Florida)	Brought on Board (Retained/Discarded)	Live - Healthy	Discarded - Live and Healthy (Not Vented)	Male (Claspers)	Small (Less than 1m)
33	UCAM	Mustelus spp.	Dogfish, Smooth (Florida)	Brought on Board (Retained/Discarded)	Live - Healthy	Discarded - Live and Healthy (Not Vented)	Male (Claspers)	Medium (Between 1m and 2m)
33	UCAM	Mustelus spp.	Dogfish, Smooth (Florida)	Brought on Board (Retained/Discarded)	Live - Healthy	Discarded - Live and Healthy (Not Vented)	Male (Claspers)	Small (Less than 1m)
33	UCAM	Mustelus spp.	Dogfish, Smooth (Florida)	Brought on Board (Retained/Discarded)	Live - Healthy	Discarded - Live and Healthy (Not Vented)	Male (Claspers)	Medium (Between 1m and 2m)
33	UCAM	Selachimorpha, unidentified	Shark, Unidentified	Brought on Board (Retained/Discarded)	Live - Healthy	Discarded - Live and Healthy (Not Vented)	Unknown Maturity and/or Sex	Medium (Between 1m and 2m)
34	NOCAM	Mustelus spp.	Dogfish, Smooth (Florida)	Brought on Board (Retained/Discarded)	Live - Healthy	Discarded - Live and Healthy (Not Vented)	Female	Medium (Between 1m and 2m)
34	NOCAM	Mustelus spp.	Dogfish, Smooth (Florida)	Brought on Board (Retained/Discarded)	Live - Healthy	Discarded - Live and Healthy (Not Vented)	Male (Claspers)	Small (Less than 1m)
34	NOCAM	Mustelus spp.	Dogfish, Smooth (Florida)	Brought on Board (Retained/Discarded)	Live - Healthy	Discarded - Live and Healthy (Not Vented)	Male (Claspers)	Medium (Between 1m and 2m)
34	NOCAM	Mustelus spp.	Dogfish, Smooth (Florida)	Brought on Board (Retained/Discarded)	Live - Healthy	Discarded - Live and Healthy (Not Vented)	Male (Claspers)	Medium (Between 1m and 2m)
34	NOCAM	Mustelus spp.	Dogfish, Smooth (Florida)	Brought on Board (Retained/Discarded)	Live - Healthy	Discarded - Live and Healthy (Not Vented)	Female	Medium (Between 1m and 2m)
34	NOCAM	Mustelus spp.	Dogfish, Smooth (Florida)	Brought on Board (Retained/Discarded)	Live - Healthy	Discarded - Live and Healthy (Not Vented)	Male (Claspers)	Medium (Between 1m and 2m)
34	NOCAM	Mustelus spp.	Dogfish, Smooth (Florida)	Brought on Board (Retained/Discarded)	Live - Healthy	Discarded - Live and Healthy (Not Vented)	Male (Claspers)	Medium (Between 1m and 2m)
34	NOCAM	Sphyrna lewini	Scalloped Hammerhead	Cut-off at Rail (No Entanglement)	Unknown Condition	Discarded - Unknown	Unknown Maturity and/or Sex	Medium (Between 1m and 2m)
34	NOCAM	Sphyrna lewini	Scalloped Hammerhead	Cut-off at Rail (No Entanglement)	Dead on Arrival - Undar	Discarded - Dead	Unknown Maturity and/or Sex	Medium (Between 1m and 2m)
34	UCAM	Mustelus spp.	Dogfish, Smooth (Florida)	Brought on Board (Retained/Discarded)	Live - Healthy	Discarded - Live and Healthy (Not Vented)	Male (Claspers)	Medium (Between 1m and 2m)
34	UCAM	Mustelus spp.	Dogfish, Smooth (Florida)	Brought on Board (Retained/Discarded)	Live - Healthy	Discarded - Live and Healthy (Not Vented)	Male (Claspers)	Medium (Between 1m and 2m)
34	UCAM	Mustelus spp.	Dogfish, Smooth (Florida)	Brought on Board (Retained/Discarded)	Live - Healthy	Discarded - Live and Healthy (Not Vented)	Male (Claspers)	Medium (Between 1m and 2m)
34	UCAM	Mustelus spp.	Dogfish, Smooth (Florida)	Brought on Board (Retained/Discarded)	Live - Healthy	Discarded - Live and Healthy (Not Vented)	Male (Claspers)	Medium (Between 1m and 2m)
34	UCAM	Mustelus spp.	Dogfish, Smooth (Florida)	Brought on Board (Retained/Discarded)	Live - Healthy	Discarded - Live and Healthy (Not Vented)	Female	Medium (Between 1m and 2m)
34	UCAM	Mustelus spp.	Dogfish, Smooth (Florida)	Brought on Board (Retained/Discarded)	Live - Healthy	Discarded - Live and Healthy (Not Vented)	Female	Medium (Between 1m and 2m)

34	UCAM	Mustelus spp.	Dogfish, Smooth (Florida)	Brought on Board (Retained/Discarded)	Live - Healthy	Discarded - Live and Healthy (Not Vented)	Male (Claspers)	Small (Less than 1m)
34	UCAM	Sphyrna lewini	Scalloped Hammerhead	Cut-off at Rail (No Entanglement)	Dead on Arrival - Undar	Discarded - Dead	Female	Medium (Between 1m and 2m)
34	UCAM	Sphyrna lewini	Scalloped Hammerhead	Cut-off at Rail (No Entanglement)	Dead on Arrival - Undar	Discarded - Dead	Female	Medium (Between 1m and 2m)
34	UCAM	Squalidae spp.	Dogfish, Spiny (Cuban)	Brought on Board (Retained/Discarded)	Live - Healthy	Discarded - Live and Healthy (Not Vented)	Male (Claspers)	Medium (Between 1m and 2m)
35	NOCAM	Mustelus spp.	Dogfish, Smooth (Florida)	Brought on Board (Retained/Discarded)	Live - Healthy	Discarded - Live and Healthy (Not Vented)	Male (Claspers)	Medium (Between 1m and 2m)
35	UCAM	Mustelus spp.	Dogfish, Smooth (Florida)	Brought on Board (Retained/Discarded)	Live - Healthy	Discarded - Live and Healthy (Not Vented)	Male (Claspers)	Medium (Between 1m and 2m)
36	NOCAM	Carcharhinus falciformis	Silky Shark	Cut-off at Rail (No Entanglement)	Live - Healthy	Discarded - Live and Healthy (Not Vented)	Unknown Maturity and/or Sex	Medium (Between 1m and 2m)
36	NOCAM	Carcharhinus plumbeus	Sandbar Shark	Cut-off at Rail (No Entanglement)	Live - Healthy	Discarded - Live and Healthy (Not Vented)	Male (Claspers)	Large (Greater than 2m)
36	UCAM	Carcharhinus falciformis	Silky Shark	Cut-off at Rail (No Entanglement)	Live - Healthy	Discarded - Live and Healthy (Not Vented)	Unknown Maturity and/or Sex	Medium (Between 1m and 2m)
36	UCAM	Carcharhinus plumbeus	Sandbar Shark	Cut-off at Rail (No Entanglement)	Live - Healthy	Discarded - Live and Healthy (Not Vented)	Male (Claspers)	Large (Greater than 2m)
37	NOCAM	Carcharhinus plumbeus	Sandbar Shark	Cut-off at Rail (No Entanglement)	Live - Healthy	Discarded - Live and Healthy (Not Vented)	Male (Claspers)	Large (Greater than 2m)
37	NOCAM	Mustelus spp.	Dogfish, Smooth (Florida)	Brought on Board (Retained/Discarded)	Live - Healthy	Discarded - Live and Healthy (Not Vented)	Female	Medium (Between 1m and 2m)
37	UCAM	Carcharhinus plumbeus	Sandbar Shark	Cut-off at Rail (No Entanglement)	Live - Healthy	Discarded - Live and Healthy (Not Vented)	Male (Claspers)	Large (Greater than 2m)
37	UCAM	Mustelus spp.	Dogfish, Smooth (Florida)	Brought on Board (Retained/Discarded)	Live - Healthy	Discarded - Live and Healthy (Not Vented)	Female	Small (Less than 1m)
37	UCAM	Mustelus spp.	Dogfish, Smooth (Florida)	Brought on Board (Retained/Discarded)	Live - Healthy	Discarded - Live and Healthy (Not Vented)	Female	Medium (Between 1m and 2m)
38	NOCAM	Carcharhinus acronotus	Blacknose Shark	Brought on Board (Retained/Discarded)	Live - Healthy	Discarded - Live and Healthy (Not Vented)	Female	Medium (Between 1m and 2m)
38	NOCAM	Carcharhinus acronotus	Blacknose Shark	Brought on Board (Retained/Discarded)	Live - Healthy	Discarded - Live and Healthy (Not Vented)	Female	Medium (Between 1m and 2m)
38	NOCAM	Rhizoprionodon terraenovae	Atlantic Sharpnose Shark	Brought on Board (Retained/Discarded)	Live - Healthy	Discarded - Live and Healthy (Not Vented)	Female	Small (Less than 1m)
38	NOCAM	Rhizoprionodon terraenovae	Atlantic Sharpnose Shark	Brought on Board (Retained/Discarded)	Live - Healthy	Discarded - Live and Healthy (Not Vented)	Female	Small (Less than 1m)
38	NOCAM	Rhizoprionodon terraenovae	Atlantic Sharpnose Shark	Brought on Board (Retained/Discarded)	Live - Healthy	Discarded - Live and Healthy (Not Vented)	Female	Small (Less than 1m)
38	NOCAM	Rhizoprionodon terraenovae	Atlantic Sharpnose Shark	Brought on Board (Retained/Discarded)	Live - Healthy	Discarded - Live and Healthy (Not Vented)	Female	Small (Less than 1m)
38	NOCAM	Rhizoprionodon terraenovae	Atlantic Sharpnose Shark	Brought on Board (Retained/Discarded)	Live - Healthy	Discarded - Live and Healthy (Not Vented)	Female	Small (Less than 1m)
38	NOCAM	Rhizoprionodon terraenovae	Atlantic Sharpnose Shark	Brought on Board (Retained/Discarded)	Live - Healthy	Discarded - Live and Healthy (Not Vented)	Female	Small (Less than 1m)
38	NOCAM	Rhizoprionodon terraenovae	Atlantic Sharpnose Shark	Brought on Board (Retained/Discarded)	Live - Healthy	Discarded - Live and Healthy (Not Vented)	Female	Small (Less than 1m)
38	NOCAM	Rhizoprionodon terraenovae	Atlantic Sharpnose Shark	Brought on Board (Retained/Discarded)	Live - Healthy	Discarded - Live and Healthy (Not Vented)	Female	Small (Less than 1m)
38	NOCAM	Rhizoprionodon terraenovae	Atlantic Sharpnose Shark	Brought on Board (Retained/Discarded)	Live - Healthy	Discarded - Live and Healthy (Not Vented)	Female	Small (Less than 1m)
38	NOCAM	Rhizoprionodon terraenovae	Atlantic Sharpnose Shark	Brought on Board (Retained/Discarded)	Live - Healthy	Discarded - Live and Healthy (Not Vented)	Female	Small (Less than 1m)
38	NOCAM	Rhizoprionodon terraenovae	Atlantic Sharpnose Shark	Brought on Board (Retained/Discarded)	Live - Healthy	Discarded - Live and Healthy (Not Vented)	Female	Small (Less than 1m)
38	NOCAM	Rhizoprionodon terraenovae	Atlantic Sharpnose Shark	Brought on Board (Retained/Discarded)	Dead on Arrival - Undar	Discarded - Dead	Female	Small (Less than 1m)
38	NOCAM	Rhizoprionodon terraenovae	Atlantic Sharpnose Shark	Brought on Board (Retained/Discarded)	Live - Healthy	Discarded - Live and Healthy (Not Vented)	Female	Small (Less than 1m)
38	NOCAM	Rhizoprionodon terraenovae	Atlantic Sharpnose Shark	Brought on Board (Retained/Discarded)	Live - Healthy	Discarded - Live and Healthy (Not Vented)	Female	Small (Less than 1m)
38	NOCAM	Rhizoprionodon terraenovae	Atlantic Sharpnose Shark	Brought on Board (Retained/Discarded)	Live - Healthy	Discarded - Live and Healthy (Not Vented)	Female	Small (Less than 1m)
38	NOCAM	Rhizoprionodon terraenovae	Atlantic Sharpnose Shark	Brought on Board (Retained/Discarded)	Live - Healthy	Discarded - Live and Healthy (Not Vented)	Female	Small (Less than 1m)
38	NOCAM	Rhizoprionodon terraenovae	Atlantic Sharpnose Shark	Brought on Board (Retained/Discarded)	Live - Healthy	Discarded - Live and Healthy (Not Vented)	Female	Small (Less than 1m)
38	UCAM	Carcharhinus acronotus	Blacknose Shark	Brought on Board (Retained/Discarded)	Live - Healthy	Discarded - Live and Healthy (Not Vented)	Female	Medium (Between 1m and 2m)
38	UCAM	Carcharhinus acronotus	Blacknose Shark	Brought on Board (Retained/Discarded)	Live - Healthy	Discarded - Live and Healthy (Not Vented)	Female	Medium (Between 1m and 2m)
38	UCAM	Carcharhinus limbatus	Blacktip Shark	Cut-off at Rail (No Entanglement)	Live - Healthy	Discarded - Live and Healthy (Not Vented)	Female	Medium (Between 1m and 2m)
38	UCAM	Rhizoprionodon terraenovae	Atlantic Sharpnose Shark	Brought on Board (Retained/Discarded)	Live - Healthy	Discarded - Live and Healthy (Not Vented)	Female	Small (Less than 1m)
38	UCAM	Rhizoprionodon terraenovae	Atlantic Sharpnose Shark	Brought on Board (Retained/Discarded)	Live - Healthy	Discarded - Live and Healthy (Not Vented)	Female	Small (Less than 1m)
38	UCAM	Rhizoprionodon terraenovae	Atlantic Sharpnose Shark	Brought on Board (Retained/Discarded)	Live - Healthy	Discarded - Live and Healthy (Not Vented)	Female	Small (Less than 1m)
38	UCAM	Rhizoprionodon terraenovae	Atlantic Sharpnose Shark	Brought on Board (Retained/Discarded)	Live - Healthy	Discarded - Live and Healthy (Not Vented)	Female	Small (Less than 1m)
38	UCAM	Rhizoprionodon terraenovae	Atlantic Sharpnose Shark	Brought on Board (Retained/Discarded)	Live - Healthy	Discarded - Live and Healthy (Not Vented)	Female	Small (Less than 1m)
38	UCAM	Rhizoprionodon terraenovae	Atlantic Sharpnose Shark	Brought on Board (Retained/Discarded)	Live - Healthy	Discarded - Live and Healthy (Not Vented)	Female	Small (Less than 1m)
38	UCAM	Rhizoprionodon terraenovae	Atlantic Sharpnose Shark	Brought on Board (Retained/Discarded)	Live - Healthy	Discarded - Live and Healthy (Not Vented)	Female	Small (Less than 1m)
38	UCAM	Rhizoprionodon terraenovae	Atlantic Sharpnose Shark	Brought on Board (Retained/Discarded)	Live - Healthy	Discarded - Live and Healthy (Not Vented)	Female	Small (Less than 1m)
38	UCAM	Rhizoprionodon terraenovae	Atlantic Sharpnose Shark	Brought on Board (Retained/Discarded)	Live - Healthy	Discarded - Live and Healthy (Not Vented)	Female	Small (Less than 1m)
38	UCAM	Rhizoprionodon terraenovae	Atlantic Sharpnose Shark	Brought on Board (Retained/Discarded)	Live - Healthy	Discarded - Live and Healthy (Not Vented)	Female	Small (Less than 1m)
38	UCAM	Rhizoprionodon terraenovae	Atlantic Sharpnose Shark	Brought on Board (Retained/Discarded)	Live - Healthy	Discarded - Live and Healthy (Not Vented)	Female	Small (Less than 1m)
38	UCAM	Rhizoprionodon terraenovae	Atlantic Sharpnose Shark	Brought on Board (Retained/Discarded)	Live - Healthy	Discarded - Live and Healthy (Not Vented)	Female	Small (Less than 1m)
38	UCAM	Rhizoprionodon terraenovae	Atlantic Sharpnose Shark	Brought on Board (Retained/Discarded)	Live - Healthy	Discarded - Live and Healthy (Not Vented)	Female	Small (Less than 1m)
38	UCAM	Rhizoprionodon terraenovae	Atlantic Sharpnose Shark	Brought on Board (Retained/Discarded)	Live - Healthy	Discarded - Live and Healthy (Not Vented)	Female	Small (Less than 1m)
38	UCAM	Rhizoprionodon terraenovae	Atlantic Sharpnose Shark	Brought on Board (Retained/Discarded)	Live - Healthy	Discarded - Live and Healthy (Not Vented)	Female	Small (Less than 1m)
38	UCAM	Rhizoprionodon terraenovae	Atlantic Sharpnose Shark	Brought on Board (Retained/Discarded)	Live - Healthy	Discarded - Live and Healthy (Not Vented)	Female	Small (Less than 1m)
38	UCAM	Rhizoprionodon terraenovae	Atlantic Sharpnose Shark	Brought on Board (Retained/Discarded)	Live - Healthy	Discarded - Live and Healthy (Not Vented)	Female	Small (Less than 1m)
39	NOCAM	Ginglymostoma cirratum	Nurse Shark	Cut-off at Rail (No Entanglement)	Live - Healthy	Discarded - Live and Healthy (Not Vented)	Known Adult - Undetermined Sex	Medium (Between 1m and 2m)
39	UCAM	Ginglymostoma cirratum	Nurse Shark	Cut-off at Rail (No Entanglement)	Live - Healthy	Discarded - Live and Healthy (Not Vented)	Male (Claspers)	Medium (Between 1m and 2m)
41	NOCAM	Galeocerdo cuvier	Tiger Shark	Cut-off at Rail (No Entanglement)	Live - Healthy	Discarded - Live and Healthy (Not Vented)	Known Juvenile	Medium (Between 1m and 2m)
41	NOCAM	Galeocerdo cuvier	Tiger Shark	Cut-off at Rail (No Entanglement)	Live - Healthy	Discarded - Live and Healthy (Not Vented)	Known Juvenile	Medium (Between 1m and 2m)
41	UCAM	Carcharhinus plumbeus	Sandbar Shark	Cut-off at Rail (No Entanglement)	Live - Healthy	Discarded - Live and Healthy (Not Vented)	Female	Large (Greater than 2m)
41	UCAM	Carcharhinus plumbeus	Sandbar Shark	Not Handled (Dropped Off)	Live - Healthy	Discarded - Live and Healthy (Not Vented)	Female	Large (Greater than 2m)
41	UCAM	Galeocerdo cuvier	Tiger Shark	Cut-off at Rail (No Entanglement)	Live - Healthy	Discarded - Live and Healthy (Not Vented)	Known Juvenile	Medium (Between 1m and 2m)
41	UCAM	Galeocerdo cuvier	Tiger Shark	Cut-off at Rail (No Entanglement)	Live - Healthy	Discarded - Live and Healthy (Not Vented)	Known Juvenile	Medium (Between 1m and 2m)
43	NOCAM	Rhizoprionodon terraenovae	Atlantic Sharpnose Shark	Brought on Board (Retained/Discarded)	Dead on Arrival - Damag	Discarded - Dead	Unknown Maturity and/or Sex	Small (Less than 1m)
43	UCAM	Rhizoprionodon terraenovae	Atlantic Sharpnose Shark	Brought on Board (Retained/Discarded)	Dead on Arrival - Damag	Discarded - Dead	Unknown Maturity and/or Sex	Small (Less than 1m)
49	NOCAM	Carcharhinus acronotus	Blacknose Shark	Brought on Board (Retained/Discarded)	Live - Healthy	Discarded - Live and Healthy (Not Vented)	Female	Medium (Between 1m and 2m)
49	NOCAM	Ginglymostoma cirratum	Nurse Shark	Cut-off at Rail (No Entanglement)	Live - Healthy	Discarded - Live and Healthy (Not Vented)	Unknown Maturity and/or Sex	Medium (Between 1m and 2m)
49	UCAM	Carcharhinus acronotus	Blacknose Shark	Brought on Board (Retained/Discarded)	Live - Healthy	Discarded - Live and Healthy (Not Vented)	Female	Medium (Between 1m and 2m)
49	UCAM	Carcharhinus plumbeus	Sandbar Shark	Cut-off at Rail (No Entanglement)	Live - Healthy	Discarded - Live and Healthy (Not Vented)	Female	Large (Greater than 2m)
49	UCAM	Ginglymostoma cirratum	Nurse Shark	Cut-off at Rail (No Entanglement)	Live - Healthy	Discarded - Live and Healthy (Not Vented)	Unknown Maturity and/or Sex	Medium (Between 1m and 2m)
50	NOCAM	Squalidae spp.	Dogfish, Spiny (Cuban)	Brought on Board (Retained/Discarded)	Live - Healthy	Discarded - Live and Healthy (Not Vented)	Unknown Maturity and/or Sex	Small (Less than 1m)
50	UCAM	Squalidae spp.	Dogfish, Spiny (Cuban)	Brought on Board (Retained/Discarded)	Live - Healthy	Discarded - Live and Healthy (Not Vented)	Unknown Maturity and/or Sex	Small (Less than 1m)
51	NOCAM	Squalidae spp.	Dogfish, Spiny (Cuban)	Brought on Board (Retained/Discarded)	Live - Healthy	Discarded - Live and Healthy (Not Vented)	Female	Small (Less than 1m)
51	NOCAM	Squalidae spp.	Dogfish, Spiny (Cuban)	Brought on Board (Retained/Discarded)	Live - Healthy	Discarded - Live and Healthy (Not Vented)	Female	Small (Less than 1m)
51	UCAM	Squalidae spp.	Dogfish, Spiny (Cuban)	Brought on Board (Retained/Discarded)	Live - Healthy	Discarded - Live and Healthy (Not Vented)	Female	Small (Less than 1m)
51	UCAM	Squalidae spp.	Dogfish, Spiny (Cuban)	Brought on Board (Retained/Discarded)	Live - Healthy	Discarded - Live and Healthy (Not Vented)	Female	Small (Less than 1m)
52	NOCAM	Carcharhinus plumbeus	Sandbar Shark	Cut-off at Rail (No Entanglement)	Live - Healthy	Discarded - Live and Healthy (Not Vented)	Known Adult - Undetermined Sex	Large (Greater than 2m)
52	NOCAM	Galeocerdo cuvier	Tiger Shark	Cut-off at Rail (No Entanglement)	Live - Healthy	Discarded - Live and Healthy (Not Vented)	Unknown Maturity and/or Sex	Large (Greater than 2m)
52	NOCAM	Rhizoprionodon terraenovae	Atlantic Sharpnose Shark	Brought on Board (Retained/Discarded)	Live - Healthy	Discarded - Live and Healthy (Not Vented)	Male (Claspers)	Small (Less than 1m)
52	UCAM	Carcharhinus plumbeus	Sandbar Shark	Cut-off at Rail (No Entanglement)	Live - Healthy	Discarded - Live and Healthy (Not Vented)	Female	Large (Greater than 2m)
52	UCAM	Carcharhinus plumbeus	Sandbar Shark	Cut-off at Rail (No Entanglement)	Live - Healthy	Discarded - Live and Healthy (Not Vented)	Female	Large (Greater than 2m)
52	UCAM	Galeocerdo cuvier	Tiger Shark	Cut-off at Rail (No Entanglement)	Live - Healthy	Discarded - Live and Healthy (Not Vented)	Female	Large (Greater than 2m)
52	UCAM	Galeocerdo cuvier	Tiger Shark	Cut-off at Rail (No Entanglement)	Live - Healthy	Discarded - Live and Healthy (Not Vented)	Female	Large (Greater than 2m)
52	UCAM	Rhizoprionodon terraenovae	Atlantic Sharpnose Shark	Brought on Board (Retained/Discarded)	Live - Healthy	Discarded - Live and Healthy (Not Vented)	Male (Claspers)	Small (Less than 1m)
53	NOCAM	Rhizoprionodon terraenovae	Atlantic Sharpnose Shark	Brought on Board (Retained/Discarded)	Live - Healthy	Discarded - Live and Healthy (Not Vented)	Male (Claspers)	Small (Less than 1m)

53	NOCAM	Selachimorpha, unidentified	Shark, Unidentified	Cut-off at Rail (No Entanglement)	Live - Healthy	Discarded - Live and Healthy (Not Vented)	Unknown Maturity and/or Sex	Large (Greater than 2m)
53	UCAM	Carcharhinus plumbeus	Sandbar Shark	Cut-off at Rail (No Entanglement)	Live - Healthy	Discarded - Live and Healthy (Not Vented)	Female	Large (Greater than 2m)
53	UCAM	Carcharhinus plumbeus	Sandbar Shark	Cut-off at Rail (No Entanglement)	Live - Healthy	Discarded - Live and Healthy (Not Vented)	Female	Large (Greater than 2m)
53	UCAM	Rhizoprionodon terraenovae	Atlantic Sharpnose Shark	Brought on Board (Retained/Discarded)	Live - Healthy	Discarded - Live and Healthy (Not Vented)	Male (Claspers)	Small (Less than 1m)
54	NOCAM	Galeocerdo cuvier	Tiger Shark	Cut-off at Rail (No Entanglement)	Live - Healthy	Discarded - Live and Healthy (Not Vented)	Known Juvenile	Medium (Between 1m and 2m)
54	UCAM	Galeocerdo cuvier	Tiger Shark	Cut-off at Rail (No Entanglement)	Live - Healthy	Discarded - Live and Healthy (Not Vented)	Known Juvenile	Medium (Between 1m and 2m)
55	NOCAM	Galeocerdo cuvier	Tiger Shark	Cut-off at Rail (No Entanglement)	Live - Healthy	Discarded - Live and Healthy (Not Vented)	Known Juvenile	Medium (Between 1m and 2m)
55	UCAM	Galeocerdo cuvier	Tiger Shark	Cut-off at Rail (No Entanglement)	Live - Healthy	Discarded - Live and Healthy (Not Vented)	Known Juvenile	Medium (Between 1m and 2m)
56	NOCAM	Rhizoprionodon terraenovae	Atlantic Sharpnose Shark	Brought on Board (Retained/Discarded)	Live - Healthy	Discarded - Live and Healthy (Not Vented)	Female	Small (Less than 1m)
56	UCAM	Rhizoprionodon terraenovae	Atlantic Sharpnose Shark	Brought on Board (Retained/Discarded)	Live - Healthy	Discarded - Live and Healthy (Not Vented)	Female	Small (Less than 1m)
57	NOCAM	Carcharhinus limbatus	Blacktip Shark	Cut-off at Rail (No Entanglement)	Live - Healthy	Discarded - Live and Healthy (Not Vented)	Unknown Maturity and/or Sex	Medium (Between 1m and 2m)
58	UCAM	Galeocerdo cuvier	Tiger Shark	Cut-off at Rail (No Entanglement)	Live - Healthy	Discarded - Live and Healthy (Not Vented)	Known Juvenile	Medium (Between 1m and 2m)
59	NOCAM	Mustelus spp.	Dogfish, Smooth (Florida)	Brought on Board (Retained/Discarded)	Live - Healthy	Discarded - Live and Healthy (Not Vented)	Male (Claspers)	Small (Less than 1m)
59	NOCAM	Mustelus spp.	Dogfish, Smooth (Florida)	Brought on Board (Retained/Discarded)	Live - Healthy	Discarded - Live and Healthy (Not Vented)	Male (Claspers)	Small (Less than 1m)
59	NOCAM	Mustelus spp.	Dogfish, Smooth (Florida)	Brought on Board (Retained/Discarded)	Live - Healthy	Discarded - Live and Healthy (Not Vented)	Male (Claspers)	Small (Less than 1m)
59	NOCAM	Mustelus spp.	Dogfish, Smooth (Florida)	Brought on Board (Retained/Discarded)	Live - Healthy	Discarded - Live and Healthy (Not Vented)	Female	Small (Less than 1m)
59	NOCAM	Mustelus spp.	Dogfish, Smooth (Florida)	Brought on Board (Retained/Discarded)	Live - Healthy	Discarded - Live and Healthy (Not Vented)	Male (Claspers)	Small (Less than 1m)
59	NOCAM	Mustelus spp.	Dogfish, Smooth (Florida)	Brought on Board (Retained/Discarded)	Live - Healthy	Discarded - Live and Healthy (Not Vented)	Male (Claspers)	Small (Less than 1m)
59	NOCAM	Mustelus spp.	Dogfish, Smooth (Florida)	Brought on Board (Retained/Discarded)	Live - Healthy	Discarded - Live and Healthy (Not Vented)	Male (Claspers)	Small (Less than 1m)
59	NOCAM	Mustelus spp.	Dogfish, Smooth (Florida)	Brought on Board (Retained/Discarded)	Live - Healthy	Discarded - Live and Healthy (Not Vented)	Male (Claspers)	Small (Less than 1m)
59	NOCAM	Mustelus spp.	Dogfish, Smooth (Florida)	Brought on Board (Retained/Discarded)	Live - Healthy	Discarded - Live and Healthy (Not Vented)	Female	Small (Less than 1m)
59	NOCAM	Mustelus spp.	Dogfish, Smooth (Florida)	Brought on Board (Retained/Discarded)	Live - Healthy	Discarded - Live and Healthy (Not Vented)	Male (Claspers)	Small (Less than 1m)
59	NOCAM	Mustelus spp.	Dogfish, Smooth (Florida)	Brought on Board (Retained/Discarded)	Live - Healthy	Discarded - Live and Healthy (Not Vented)	Male (Claspers)	Small (Less than 1m)
59	NOCAM	Mustelus spp.	Dogfish, Smooth (Florida)	Brought on Board (Retained/Discarded)	Live - Healthy	Discarded - Live and Healthy (Not Vented)	Female	Small (Less than 1m)
59	NOCAM	Mustelus spp.	Dogfish, Smooth (Florida)	Brought on Board (Retained/Discarded)	Live - Healthy	Discarded - Live and Healthy (Not Vented)	Male (Claspers)	Small (Less than 1m)
59	NOCAM	Mustelus spp.	Dogfish, Smooth (Florida)	Brought on Board (Retained/Discarded)	Live - Healthy	Discarded - Live and Healthy (Not Vented)	Male (Claspers)	Small (Less than 1m)
59	NOCAM	Mustelus spp.	Dogfish, Smooth (Florida)	Brought on Board (Retained/Discarded)	Live - Healthy	Discarded - Live and Healthy (Not Vented)	Female	Small (Less than 1m)
59	NOCAM	Mustelus spp.	Dogfish, Smooth (Florida)	Brought on Board (Retained/Discarded)	Live - Healthy	Discarded - Live and Healthy (Not Vented)	Male (Claspers)	Small (Less than 1m)
59	NOCAM	Mustelus spp.	Dogfish, Smooth (Florida)	Brought on Board (Retained/Discarded)	Live - Healthy	Discarded - Live and Healthy (Not Vented)	Female	Small (Less than 1m)
59	NOCAM	Mustelus spp.	Dogfish, Smooth (Florida)	Brought on Board (Retained/Discarded)	Live - Healthy	Discarded - Live and Healthy (Not Vented)	Male (Claspers)	Small (Less than 1m)
59	NOCAM	Mustelus spp.	Dogfish, Smooth (Florida)	Brought on Board (Retained/Discarded)	Live - Healthy	Discarded - Live and Healthy (Not Vented)	Male (Claspers)	Small (Less than 1m)
59	NOCAM	Mustelus spp.	Dogfish, Smooth (Florida)	Brought on Board (Retained/Discarded)	Live - Healthy	Discarded - Live and Healthy (Not Vented)	Male (Claspers)	Small (Less than 1m)
59	NOCAM	Mustelus spp.	Dogfish, Smooth (Florida)	Brought on Board (Retained/Discarded)	Live - Healthy	Discarded - Live and Healthy (Not Vented)	Male (Claspers)	Small (Less than 1m)
59	NOCAM	Mustelus spp.	Dogfish, Smooth (Florida)	Brought on Board (Retained/Discarded)	Live - Healthy	Discarded - Live and Healthy (Not Vented)	Male (Claspers)	Medium (Between 1m and 2m)
59	NOCAM	Mustelus spp.	Dogfish, Smooth (Florida)	Brought on Board (Retained/Discarded)	Live - Healthy	Discarded - Live and Healthy (Not Vented)	Female	Small (Less than 1m)
59	NOCAM	Mustelus spp.	Dogfish, Smooth (Florida)	Brought on Board (Retained/Discarded)	Live - Healthy	Discarded - Live and Healthy (Not Vented)	Male (Claspers)	Small (Less than 1m)
59	NOCAM	Mustelus spp.	Dogfish, Smooth (Florida)	Brought on Board (Retained/Discarded)	Live - Healthy	Discarded - Live and Healthy (Not Vented)	Female	Small (Less than 1m)
59	NOCAM	Mustelus spp.	Dogfish, Smooth (Florida)	Brought on Board (Retained/Discarded)	Live - Healthy	Discarded - Live and Healthy (Not Vented)	Male (Claspers)	Small (Less than 1m)
59	NOCAM	Selachimorpha, unidentified	Shark, Unidentified	Cut-off at Rail (No Entanglement)	Live - Healthy	Discarded - Live and Healthy (Not Vented)	Unknown Maturity and/or Sex	Large (Greater than 2m)
59	NOCAM	Sphyrna lewini	Scalloped Hammerhead	Cut-off at Rail (No Entanglement)	Dead on Arrival - Undar	Discarded - Dead	Unknown Maturity and/or Sex	Medium (Between 1m and 2m)
59	UCAM	Carcharhinus plumbeus	Sandbar Shark	Cut-off at Rail (No Entanglement)	Live - Healthy	Discarded - Live and Healthy (Not Vented)	Male (Claspers)	Large (Greater than 2m)
59	UCAM	Mustelus spp.	Dogfish, Smooth (Florida)	Brought on Board (Retained/Discarded)	Live - Healthy	Discarded - Live and Healthy (Not Vented)	Male (Claspers)	Small (Less than 1m)
59	UCAM	Mustelus spp.	Dogfish, Smooth (Florida)	Brought on Board (Retained/Discarded)	Live - Healthy	Discarded - Live and Healthy (Not Vented)	Female	Small (Less than 1m)
59	UCAM	Mustelus spp.	Dogfish, Smooth (Florida)	Brought on Board (Retained/Discarded)	Live - Healthy	Discarded - Live and Healthy (Not Vented)	Male (Claspers)	Small (Less than 1m)
59	UCAM	Mustelus spp.	Dogfish, Smooth (Florida)	Brought on Board (Retained/Discarded)	Live - Healthy	Discarded - Live and Healthy (Not Vented)	Male (Claspers)	Small (Less than 1m)
59	UCAM	Mustelus spp.	Dogfish, Smooth (Florida)	Brought on Board (Retained/Discarded)	Live - Healthy	Discarded - Live and Healthy (Not Vented)	Male (Claspers)	Small (Less than 1m)
59	UCAM	Mustelus spp.	Dogfish, Smooth (Florida)	Brought on Board (Retained/Discarded)	Live - Healthy	Discarded - Live and Healthy (Not Vented)	Female	Small (Less than 1m)
59	UCAM	Mustelus spp.	Dogfish, Smooth (Florida)	Brought on Board (Retained/Discarded)	Live - Healthy	Discarded - Live and Healthy (Not Vented)	Male (Claspers)	Small (Less than 1m)
59	UCAM	Mustelus spp.	Dogfish, Smooth (Florida)	Brought on Board (Retained/Discarded)	Live - Healthy	Discarded - Live and Healthy (Not Vented)	Male (Claspers)	Small (Less than 1m)
59	UCAM	Mustelus spp.	Dogfish, Smooth (Florida)	Brought on Board (Retained/Discarded)	Live - Healthy	Discarded - Live and Healthy (Not Vented)	Male (Claspers)	Small (Less than 1m)
59	UCAM	Mustelus spp.	Dogfish, Smooth (Florida)	Brought on Board (Retained/Discarded)	Live - Healthy	Discarded - Live and Healthy (Not Vented)	Male (Claspers)	Small (Less than 1m)
59	UCAM	Mustelus spp.	Dogfish, Smooth (Florida)	Brought on Board (Retained/Discarded)	Live - Healthy	Discarded - Live and Healthy (Not Vented)	Male (Claspers)	Small (Less than 1m)
59	UCAM	Mustelus spp.	Dogfish, Smooth (Florida)	Brought on Board (Retained/Discarded)	Live - Healthy	Discarded - Live and Healthy (Not Vented)	Female	Small (Less than 1m)
59	UCAM	Mustelus spp.	Dogfish, Smooth (Florida)	Brought on Board (Retained/Discarded)	Live - Healthy	Discarded - Live and Healthy (Not Vented)	Male (Claspers)	Small (Less than 1m)
59	UCAM	Mustelus spp.	Dogfish, Smooth (Florida)	Brought on Board (Retained/Discarded)	Live - Healthy	Discarded - Live and Healthy (Not Vented)	Male (Claspers)	Medium (Between 1m and 2m)
59	UCAM	Mustelus spp.	Dogfish, Smooth (Florida)	Brought on Board (Retained/Discarded)	Live - Healthy	Discarded - Live and Healthy (Not Vented)	Male (Claspers)	Small (Less than 1m)
59	UCAM	Mustelus spp.	Dogfish, Smooth (Florida)	Brought on Board (Retained/Discarded)	Live - Healthy	Discarded - Live and Healthy (Not Vented)	Female	Small (Less than 1m)
59	UCAM	Mustelus spp.	Dogfish, Smooth (Florida)	Brought on Board (Retained/Discarded)	Live - Healthy	Discarded - Live and Healthy (Not Vented)	Female	Small (Less than 1m)
59	UCAM	Mustelus spp.	Dogfish, Smooth (Florida)	Brought on Board (Retained/Discarded)	Live - Healthy	Discarded - Live and Healthy (Not Vented)	Male (Claspers)	Small (Less than 1m)
59	UCAM	Mustelus spp.	Dogfish, Smooth (Florida)	Brought on Board (Retained/Discarded)	Live - Healthy	Discarded - Live and Healthy (Not Vented)	Male (Claspers)	Small (Less than 1m)
59	UCAM	Mustelus spp.	Dogfish, Smooth (Florida)	Brought on Board (Retained/Discarded)	Live - Healthy	Discarded - Live and Healthy (Not Vented)	Male (Claspers)	Small (Less than 1m)
59	UCAM	Mustelus spp.	Dogfish, Smooth (Florida)	Brought on Board (Retained/Discarded)	Live - Healthy	Discarded - Live and Healthy (Not Vented)	Female	Small (Less than 1m)
59	UCAM	Mustelus spp.	Dogfish, Smooth (Florida)	Brought on Board (Retained/Discarded)	Live - Healthy	Discarded - Live and Healthy (Not Vented)	Male (Claspers)	Small (Less than 1m)
59	UCAM	Mustelus spp.	Dogfish, Smooth (Florida)	Brought on Board (Retained/Discarded)	Live - Healthy	Discarded - Live and Healthy (Not Vented)	Male (Claspers)	Small (Less than 1m)
59	UCAM	Mustelus spp.	Dogfish, Smooth (Florida)	Brought on Board (Retained/Discarded)	Live - Healthy	Discarded - Live and Healthy (Not Vented)	Male (Claspers)	Small (Less than 1m)
59	UCAM	Mustelus spp.	Dogfish, Smooth (Florida)	Brought on Board (Retained/Discarded)	Live - Healthy	Discarded - Live and Healthy (Not Vented)	Female	Small (Less than 1m)
59	UCAM	Mustelus spp.	Dogfish, Smooth (Florida)	Brought on Board (Retained/Discarded)	Live - Healthy	Discarded - Live and Healthy (Not Vented)	Male (Claspers)	Small (Less than 1m)
59	UCAM	Sphyrna lewini	Scalloped Hammerhead	Cut-off at Rail (No Entanglement)	Dead on Arrival - Undar	Discarded - Dead	Female	Medium (Between 1m and 2m)
60	NOCAM	Hexanchus spp.	Sixgill Shark (all)	Brought on Board (Retained/Discarded)	Live - Healthy	Discarded - Live and Healthy (Not Vented)	Female	Small (Less than 1m)
60	NOCAM	Squalidae spp.	Dogfish, Spiny (Cuban)	Brought on Board (Retained/Discarded)	Live - Healthy	Discarded - Live and Healthy (Not Vented)	Unknown Maturity and/or Sex	Small (Less than 1m)
60	NOCAM	Squalidae spp.	Dogfish, Spiny (Cuban)	Brought on Board (Retained/Discarded)	Live - Healthy	Discarded - Live and Healthy (Not Vented)	Unknown Maturity and/or Sex	Small (Less than 1m)
60	NOCAM	Squalidae spp.	Dogfish, Spiny (Cuban)	Brought on Board (Retained/Discarded)	Live - Healthy	Discarded - Live and Healthy (Not Vented)	Unknown Maturity and/or Sex	Small (Less than 1m)
60	NOCAM	Squalidae spp.	Dogfish, Spiny (Cuban)	Brought on Board (Retained/Discarded)	Live - Healthy	Discarded - Live and Healthy (Not Vented)	Female	Small (Less than 1m)
60	NOCAM	Squalidae spp.	Dogfish, Spiny (Cuban)	Brought on Board (Retained/Discarded)	Live - Healthy	Discarded - Live and Healthy (Not Vented)	Unknown Maturity and/or Sex	Small (Less than 1m)
60	UCAM	Hexanchus spp.	Sixgill Shark (all)	Brought on Board (Retained/Discarded)	Live - Healthy	Discarded - Live and Healthy (Not Vented)	Female	Small (Less than 1m)
60	UCAM	Squalidae spp.	Dogfish, Spiny (Cuban)	Brought on Board (Retained/Discarded)	Live - Healthy	Discarded - Live and Healthy (Not Vented)	Unknown Maturity and/or Sex	Small (Less than 1m)
60	UCAM	Squalidae spp.	Dogfish, Spiny (Cuban)	Brought on Board (Retained/Discarded)	Live - Healthy	Discarded - Live and Healthy (Not Vented)	Female	Small (Less than 1m)
60	UCAM	Squalidae spp.	Dogfish, Spiny (Cuban)	Brought on Board (Retained/Discarded)	Live - Healthy	Discarded - Live and Healthy (Not Vented)	Unknown Maturity and/or Sex	Small (Less than 1m)
60	UCAM	Squalidae spp.	Dogfish, Spiny (Cuban)	Brought on Board (Retained/Discarded)	Live - Healthy	Discarded - Live and Healthy (Not Vented)	Unknown Maturity and/or Sex	Small (Less than 1m)
61	NOCAM	Squalidae spp.	Dogfish, Spiny (Cuban)	Brought on Board (Retained/Discarded)	Live - Healthy	Discarded - Live and Healthy (Not Vented)	Unknown Maturity and/or Sex	Small (Less than 1m)
61	NOCAM	Squalidae spp.	Dogfish, Spiny (Cuban)	Brought on Board (Retained/Discarded)	Live - Healthy	Discarded - Live and Healthy (Not Vented)	Unknown Maturity and/or Sex	Small (Less than 1m)
61	NOCAM	Squalidae spp.	Dogfish, Spiny (Cuban)	Brought on Board (Retained/Discarded)	Live - Healthy	Discarded - Live and Healthy (Not Vented)	Unknown Maturity and/or Sex	Small (Less than 1m)
61	NOCAM	Squalidae spp.	Dogfish, Spiny (Cuban)	Brought on Board (Retained/Discarded)	Live - Healthy	Discarded - Live and Healthy (Not Vented)	Unknown Maturity and/or Sex	Small (Less than 1m)
61	NOCAM	Squalidae spp.	Dogfish, Spiny (Cuban)	Brought on Board (Retained/Discarded)	Live - Healthy	Discarded - Live and Healthy (Not Vented)	Unknown Maturity and/or Sex	Small (Less than 1m)

61	NOCAM	Squalidae spp.	Dogfish, Spiny (Cuban)	Brought on Board (Retained/Discarded)	Live - Healthy	Discarded - Live and Healthy (Not Vented)	Unknown Maturity and/or Sex	Small (Less than 1m)
61	NOCAM	Squalidae spp.	Dogfish, Spiny (Cuban)	Cut-off at Rail (No Entanglement)	Live - Healthy	Discarded - Live and Healthy (Not Vented)	Unknown Maturity and/or Sex	Small (Less than 1m)
61	UCAM	Squalidae spp.	Dogfish, Spiny (Cuban)	Cut-off at Rail (No Entanglement)	Live - Healthy	Discarded - Live and Healthy (Not Vented)	Unknown Maturity and/or Sex	Small (Less than 1m)
61	UCAM	Squalidae spp.	Dogfish, Spiny (Cuban)	Brought on Board (Retained/Discarded)	Live - Healthy	Discarded - Live and Healthy (Not Vented)	Unknown Maturity and/or Sex	Small (Less than 1m)
61	UCAM	Squalidae spp.	Dogfish, Spiny (Cuban)	Brought on Board (Retained/Discarded)	Live - Healthy	Discarded - Live and Healthy (Not Vented)	Unknown Maturity and/or Sex	Small (Less than 1m)
61	UCAM	Squalidae spp.	Dogfish, Spiny (Cuban)	Brought on Board (Retained/Discarded)	Live - Healthy	Discarded - Live and Healthy (Not Vented)	Unknown Maturity and/or Sex	Small (Less than 1m)
61	UCAM	Squalidae spp.	Dogfish, Spiny (Cuban)	Brought on Board (Retained/Discarded)	Live - Healthy	Discarded - Live and Healthy (Not Vented)	Unknown Maturity and/or Sex	Small (Less than 1m)
62	NOCAM	Mustelus spp.	Dogfish, Smooth (Florida)	Cut-off at Rail (No Entanglement)	Live - Healthy	Discarded - Live and Healthy (Not Vented)	Unknown Maturity and/or Sex	Medium (Between 1m and 2m)
62	UCAM	Mustelus spp.	Dogfish, Smooth (Florida)	Cut-off at Rail (No Entanglement)	Live - Healthy	Discarded - Live and Healthy (Not Vented)	Unknown Maturity and/or Sex	Medium (Between 1m and 2m)
63	NOCAM	Carcharhinus falciformis	Silky Shark	Cut-off at Rail (No Entanglement)	Live - Healthy	Discarded - Live and Healthy (Not Vented)	Unknown Maturity and/or Sex	Medium (Between 1m and 2m)
63	NOCAM	Mustelus spp.	Dogfish, Smooth (Florida)	Brought on Board (Retained/Discarded)	Live - Healthy	Discarded - Live and Healthy (Not Vented)	Female	Small (Less than 1m)
63	NOCAM	Mustelus spp.	Dogfish, Smooth (Florida)	Brought on Board (Retained/Discarded)	Live - Healthy	Discarded - Live and Healthy (Not Vented)	Male (Claspers)	Small (Less than 1m)
63	NOCAM	Mustelus spp.	Dogfish, Smooth (Florida)	Brought on Board (Retained/Discarded)	Live - Healthy	Discarded - Live and Healthy (Not Vented)	Male (Claspers)	Small (Less than 1m)
63	NOCAM	Mustelus spp.	Dogfish, Smooth (Florida)	Brought on Board (Retained/Discarded)	Live - Healthy	Discarded - Live and Healthy (Not Vented)	Male (Claspers)	Small (Less than 1m)
63	NOCAM	Mustelus spp.	Dogfish, Smooth (Florida)	Brought on Board (Retained/Discarded)	Live - Healthy	Discarded - Live and Healthy (Not Vented)	Female	Small (Less than 1m)
63	NOCAM	Mustelus spp.	Dogfish, Smooth (Florida)	Brought on Board (Retained/Discarded)	Live - Healthy	Discarded - Live and Healthy (Not Vented)	Male (Claspers)	Small (Less than 1m)
63	NOCAM	Mustelus spp.	Dogfish, Smooth (Florida)	Not Handled (Dropped Off)	Live - Healthy	Discarded - Live and Healthy (Not Vented)	Unknown Maturity and/or Sex	Small (Less than 1m)
63	NOCAM	Mustelus spp.	Dogfish, Smooth (Florida)	Brought on Board (Retained/Discarded)	Live - Healthy	Discarded - Live and Healthy (Not Vented)	Male (Claspers)	Medium (Between 1m and 2m)
63	NOCAM	Mustelus spp.	Dogfish, Smooth (Florida)	Brought on Board (Retained/Discarded)	Live - Healthy	Discarded - Live and Healthy (Not Vented)	Unknown Maturity and/or Sex	Small (Less than 1m)
63	NOCAM	Mustelus spp.	Dogfish, Smooth (Florida)	Brought on Board (Retained/Discarded)	Live - Healthy	Discarded - Live and Healthy (Not Vented)	Male (Claspers)	Medium (Between 1m and 2m)
63	NOCAM	Selachimorpha, unidentified	Shark, Unidentified	Cut-off at Rail (No Entanglement)	Dead on Arrival - Undar	Discarded - Dead	Unknown Maturity and/or Sex	Medium (Between 1m and 2m)
63	NOCAM	Sphyrna lewini	Scalloped Hammerhead	Cut-off at Rail (No Entanglement)	Dead on Arrival - Undar	Discarded - Dead	Unknown Maturity and/or Sex	Medium (Between 1m and 2m)
63	NOCAM	Sphyrna lewini	Scalloped Hammerhead	Cut-off at Rail (No Entanglement)	Live - Healthy	Discarded - Live and Healthy (Not Vented)	Unknown Maturity and/or Sex	Medium (Between 1m and 2m)
63	UCAM	Carcharhinus falciformis	Silky Shark	Cut-off at Rail (No Entanglement)	Live - Healthy	Discarded - Live and Healthy (Not Vented)	Female	Medium (Between 1m and 2m)
63	UCAM	Mustelus spp.	Dogfish, Smooth (Florida)	Brought on Board (Retained/Discarded)	Live - Healthy	Discarded - Live and Healthy (Not Vented)	Male (Claspers)	Medium (Between 1m and 2m)
63	UCAM	Mustelus spp.	Dogfish, Smooth (Florida)	Not Handled (Dropped Off)	Live - Healthy	Discarded - Live and Healthy (Not Vented)	Female	Small (Less than 1m)
63	UCAM	Mustelus spp.	Dogfish, Smooth (Florida)	Brought on Board (Retained/Discarded)	Live - Healthy	Discarded - Live and Healthy (Not Vented)	Male (Claspers)	Small (Less than 1m)
63	UCAM	Mustelus spp.	Dogfish, Smooth (Florida)	Brought on Board (Retained/Discarded)	Live - Healthy	Discarded - Live and Healthy (Not Vented)	Female	Small (Less than 1m)
63	UCAM	Mustelus spp.	Dogfish, Smooth (Florida)	Brought on Board (Retained/Discarded)	Live - Healthy	Discarded - Live and Healthy (Not Vented)	Male (Claspers)	Small (Less than 1m)
63	UCAM	Mustelus spp.	Dogfish, Smooth (Florida)	Brought on Board (Retained/Discarded)	Live - Healthy	Discarded - Live and Healthy (Not Vented)	Male (Claspers)	Medium (Between 1m and 2m)
63	UCAM	Mustelus spp.	Dogfish, Smooth (Florida)	Brought on Board (Retained/Discarded)	Live - Healthy	Discarded - Live and Healthy (Not Vented)	Unknown Maturity and/or Sex	Small (Less than 1m)
63	UCAM	Mustelus spp.	Dogfish, Smooth (Florida)	Brought on Board (Retained/Discarded)	Live - Healthy	Discarded - Live and Healthy (Not Vented)	Male (Claspers)	Small (Less than 1m)
63	UCAM	Mustelus spp.	Dogfish, Smooth (Florida)	Brought on Board (Retained/Discarded)	Live - Healthy	Discarded - Live and Healthy (Not Vented)	Male (Claspers)	Small (Less than 1m)
63	UCAM	Mustelus spp.	Dogfish, Smooth (Florida)	Brought on Board (Retained/Discarded)	Live - Healthy	Discarded - Live and Healthy (Not Vented)	Female	Small (Less than 1m)
63	UCAM	Selachimorpha, unidentified	Shark, Unidentified	Brought on Board (Retained/Discarded)	Dead on Arrival - Undar	Discarded - Dead	Unknown Maturity and/or Sex	Medium (Between 1m and 2m)
63	UCAM	Sphyrna lewini	Scalloped Hammerhead	Cut-off at Rail (No Entanglement)	Live - Healthy	Discarded - Live and Healthy (Not Vented)	Female	Medium (Between 1m and 2m)
63	UCAM	Sphyrna lewini	Scalloped Hammerhead	Cut-off at Rail (No Entanglement)	Live - Healthy	Discarded - Live and Healthy (Not Vented)	Female	Medium (Between 1m and 2m)
68	NOCAM	Carcharhinus signatus	Night Shark	Brought on Board (Retained/Discarded)	Live - Healthy	Discarded - Live and Healthy (Not Vented)	Unknown Maturity and/or Sex	Medium (Between 1m and 2m)
68	NOCAM	Carcharhinus signatus	Night Shark	Brought on Board (Retained/Discarded)	Live - Healthy	Discarded - Live and Healthy (Not Vented)	Unknown Maturity and/or Sex	Medium (Between 1m and 2m)
68	NOCAM	Carcharhinus signatus	Night Shark	Brought on Board (Retained/Discarded)	Dead on Arrival - Undar	Discarded - Dead	Female	Medium (Between 1m and 2m)
68	NOCAM	Selachimorpha, unidentified	Shark, Unidentified	Cut-off at Rail (No Entanglement)	Live - Healthy	Discarded - Live and Healthy (Not Vented)	Unknown Maturity and/or Sex	Medium (Between 1m and 2m)
68	NOCAM	Squalidae spp.	Dogfish, Spiny (Cuban)	Brought on Board (Retained/Discarded)	Live - Healthy	Discarded - Live and Healthy (Not Vented)	Unknown Maturity and/or Sex	Small (Less than 1m)
68	NOCAM	Squalidae spp.	Dogfish, Spiny (Cuban)	Brought on Board (Retained/Discarded)	Live - Healthy	Discarded - Live and Healthy (Not Vented)	Unknown Maturity and/or Sex	Small (Less than 1m)
68	NOCAM	Squalidae spp.	Dogfish, Spiny (Cuban)	Brought on Board (Retained/Discarded)	Live - Healthy	Discarded - Live and Healthy (Not Vented)	Unknown Maturity and/or Sex	Small (Less than 1m)
68	NOCAM	Squalidae spp.	Dogfish, Spiny (Cuban)	Brought on Board (Retained/Discarded)	Live - Healthy	Discarded - Live and Healthy (Not Vented)	Female	Small (Less than 1m)
68	NOCAM	Squalidae spp.	Dogfish, Spiny (Cuban)	Brought on Board (Retained/Discarded)	Live - Healthy	Discarded - Live and Healthy (Not Vented)	Unknown Maturity and/or Sex	Small (Less than 1m)
68	NOCAM	Squalidae spp.	Dogfish, Spiny (Cuban)	Brought on Board (Retained/Discarded)	Live - Healthy	Discarded - Live and Healthy (Not Vented)	Unknown Maturity and/or Sex	Small (Less than 1m)
68	NOCAM	Squalidae spp.	Dogfish, Spiny (Cuban)	Brought on Board (Retained/Discarded)	Live - Healthy	Discarded - Live and Healthy (Not Vented)	Unknown Maturity and/or Sex	Small (Less than 1m)
68	UCAM	Carcharhinus signatus	Night Shark	Brought on Board (Retained/Discarded)	Live - Healthy	Discarded - Live and Healthy (Not Vented)	Unknown Maturity and/or Sex	Medium (Between 1m and 2m)
68	UCAM	Carcharhinus signatus	Night Shark	Brought on Board (Retained/Discarded)	Live - Healthy	Discarded - Live and Healthy (Not Vented)	Unknown Maturity and/or Sex	Medium (Between 1m and 2m)
68	UCAM	Carcharhinus signatus	Night Shark	Brought on Board (Retained/Discarded)	Dead on Arrival - Undar	Discarded - Dead	Female	Medium (Between 1m and 2m)
68	UCAM	Selachimorpha, unidentified	Shark, Unidentified	Cut-off at Rail (No Entanglement)	Live - Healthy	Discarded - Live and Healthy (Not Vented)	Unknown Maturity and/or Sex	Medium (Between 1m and 2m)
68	UCAM	Squalidae spp.	Dogfish, Spiny (Cuban)	Brought on Board (Retained/Discarded)	Live - Healthy	Discarded - Live and Healthy (Not Vented)	Female	Small (Less than 1m)
68	UCAM	Squalidae spp.	Dogfish, Spiny (Cuban)	Brought on Board (Retained/Discarded)	Live - Healthy	Discarded - Live and Healthy (Not Vented)	Unknown Maturity and/or Sex	Small (Less than 1m)
68	UCAM	Squalidae spp.	Dogfish, Spiny (Cuban)	Brought on Board (Retained/Discarded)	Live - Healthy	Discarded - Live and Healthy (Not Vented)	Unknown Maturity and/or Sex	Small (Less than 1m)
68	UCAM	Squalidae spp.	Dogfish, Spiny (Cuban)	Brought on Board (Retained/Discarded)	Live - Healthy	Discarded - Live and Healthy (Not Vented)	Unknown Maturity and/or Sex	Small (Less than 1m)
68	UCAM	Squalidae spp.	Dogfish, Spiny (Cuban)	Brought on Board (Retained/Discarded)	Live - Healthy	Discarded - Live and Healthy (Not Vented)	Unknown Maturity and/or Sex	Small (Less than 1m)
68	UCAM	Squalidae spp.	Dogfish, Spiny (Cuban)	Brought on Board (Retained/Discarded)	Live - Healthy	Discarded - Live and Healthy (Not Vented)	Unknown Maturity and/or Sex	Small (Less than 1m)
68	UCAM	Squalidae spp.	Dogfish, Spiny (Cuban)	Brought on Board (Retained/Discarded)	Live - Healthy	Discarded - Live and Healthy (Not Vented)	Unknown Maturity and/or Sex	Small (Less than 1m)
